# The Exosome Component Rrp6 Is Required for RNA Polymerase II Termination at Specific Targets of the Nrd1-Nab3 Pathway

**DOI:** 10.1371/journal.pgen.1004999

**Published:** 2015-02-13

**Authors:** Melanie J. Fox, Hongyu Gao, Whitney R. Smith-Kinnaman, Yunlong Liu, Amber L. Mosley

**Affiliations:** 1 Department of Biochemistry and Molecular Biology, Indiana University School of Medicine, Indianapolis, Indiana, United States of America; 2 Department of Medical and Molecular Genetics, Indiana University School of Medicine, Indianapolis, Indiana, United States of America; 3 Center for Computational Biology and Bioinformatics, Indiana University School of Medicine, Indianapolis, Indiana, United States of America; The Johns Hopkins University School of Medicine, UNITED STATES

## Abstract

The exosome and its nuclear specific subunit Rrp6 form a 3’-5’ exonuclease complex that regulates diverse aspects of RNA biology including 3’ end processing and degradation of a variety of noncoding RNAs (ncRNAs) and unstable transcripts. Known targets of the nuclear exosome include short (<1000 bp) RNAPII transcripts such as small noncoding RNAs (snRNAs), cryptic unstable transcripts (CUTs), and some stable unannotated transcripts (SUTs) that are terminated by an Nrd1, Nab3, and Sen1 (NNS) dependent mechanism. NNS-dependent termination is coupled to RNA 3’ end processing and/or degradation by the Rrp6/exosome in yeast. Recent work suggests Nrd1 is necessary for transcriptome surveillance, regulating promoter directionality and suppressing antisense transcription independently of, or prior to, Rrp6 activity. It remains unclear whether Rrp6 is directly involved in termination; however, Rrp6 has been implicated in the 3’ end processing and degradation of ncRNA transcripts including CUTs. To determine the role of Rrp6 in NNS termination globally, we performed RNA sequencing (RNA-Seq) on total RNA and perform ChIP-exo analysis of RNA Polymerase II (RNAPII) localization. Deletion of *RRP6* promotes hyper-elongation of multiple NNS-dependent transcripts resulting from both improperly processed 3’ RNA ends and faulty transcript termination at specific target genes. The defects in RNAPII termination cause transcriptome-wide changes in mRNA expression through transcription interference and/or antisense repression, similar to previously reported effects of depleting Nrd1 from the nucleus. Elongated transcripts were identified within all classes of known NNS targets with the largest changes in transcription termination occurring at CUTs. Interestingly, the extended transcripts that we have detected in our studies show remarkable similarity to Nrd1-unterminated transcripts at many locations, suggesting that Rrp6 acts with the NNS complex globally to promote transcription termination in addition to 3’ end RNA processing and/or degradation at specific targets.

## Author Summary

RNAPII is responsible for transcription of protein-coding genes and short, regulatory RNAs. In *Saccharomyces cerevisiae*, termination of RNAPII-transcribed RNAs ≤1000 bases requires the NNS complex (comprised of Nrd1, Nab3, and Sen1), processing by the exosome, and the nuclear specific catalytic subunit, Rrp6. It has been shown that Rrp6 interacts directly with Nrd1, but whether or not Rrp6 is required for NNS-dependent termination is unclear. Loss of Rrp6 function may result in extension (or inhibition of termination) of NNS-dependent transcripts, or Rrp6 may only function after the fact to carry out RNA 3’ end processing. Here, we performed in-depth differential expression analyses and compare RNA-sequencing data of transcript length and abundance in cells lacking *RRP6* to ChIP-exo analysis of RNAPII localization. We find many transcripts that were defined as unterminated upon loss of Nrd1 activity are of similar length in *rrp6Δ*, and expression levels of downstream genes are significantly decreased. This suggests a similar transcription interference mechanism occurs in cells lacking either Nrd1 or Rrp6. Indeed we find increased RNAPII located downstream of its termination site at many know Nrd1-regulated transcripts. Overall, our findings clearly demonstrate that Rrp6 activity is required for efficient NNS termination *in vivo*.

## Introduction

The exosome is a 3’-5’ exonuclease complex involved in termination of short RNAs, RNA quality control surveillance, and RNA degradation in both the cytoplasm and the nucleus. The core RNA exosome is a multi-subunit complex that has been proposed to channel the substrate to the catalytic subunit Dis3 (also known as Rrp44), similar in structure to the proteasome [1,2,3,4]. Dis3 has both an endonucleolytic PIN domain and 3’-5’ exonuclease activity and is essential for viability of the yeast *Saccharomyces cerevisiae* [5,6]. In the nucleus, the exosome aquires a second exonuclease subunit, Rrp6. Although Rrp6 is not essential for viability, it has been shown to be required for proper 3’ end trimming of primary small nucleolar/nuclear RNAs (snRNAs) [7,8], degradation of short-lived, non-coding cryptic unstable transcripts (CUTs) [9,10,11,12,13] and improperly terminated RNAs [7,14], as well as regulation of polyA tail length through Nab2 and the non-canonical polyA polymerases Trf4 and Trf5 [15,16]. Recent studies using high-resolution tiling arrays found that Dis3 and Rrp6 have both shared and distinct roles in the degradation of various RNAs [17,18]. For instance, Rrp6 is largely responsible for snRNA processing whereas both Rrp6 and Dis3 seem to be responsible for the degradation of unspliced pre-mRNAs [17,18]. Rrp6 has also been implicated in the termination and 3’ end processing of a variety of noncoding RNAs, most of which are not terminated by traditional polyA-dependent termination mechanisms [12,19].

RNA polymerase II (RNAPII) transcription termination is coupled to 3’ end processing and carried out by two pathways [20]. The recruitment of factors involved in these pathways is orchestrated by post-translational modifications along the repetitive C-terminal domain (CTD) of Rpb1, the largest subunit of RNAPII (reviewed in [21]). Generally, long (>1000 bp) polyadenylated mRNAs are terminated by the Cleavage and Polyadenylation Factor (CPF) and Cleavage Factor (CF) complexes in a polyadenylation dependent mechanism [22,23,24,25,26]. The second pathway predominates on short (<1000 bp) RNAPII transcripts including snRNAs, CUTs, and stable unannotated transcripts (SUTs) that are terminated by an Nrd1, Nab3, and Sen1 (NNS) dependent mechanism [20,27,28,29,30]. Termination by NNS is coupled to RNA 3’ end processing and/or degradation by the Rrp6-containing nuclear exosome [8,31,32]. In the current model of NNS termination, Nrd1 can be recruited to the RNAPII CTD by its CTD interacting domain (CID) [8,33,34,35,36,37]. Nrd1 and Nab3 also contain RNA binding domains that facilitate their recruitment to specific sequences in the nascent RNA [38,39]. The helicase Sen1 participates through unwinding RNA:DNA hybrids formed by the nascent RNA and template strand promoting RNAPII termination in yeast and mammals [40,41,42,43]. RNAPII transcribes beyond the 3’ end of the functional RNA into a “termination zone” before it is terminated by Sen1 [40]. The RNA is then polyadenylated by the TRAMP complex, and the nuclear exosome trims the ends of stable transcripts (such as snRNAs and SUTs) or completely degrades the transcript in the case of CUTs [19,20,29,32,40,44,45,46,47].

Here, we focus on the role of Rrp6 in termination and processing of RNAPII-transcribed noncoding RNAs (ncRNAs) and specific mRNAs that are likely regulated through the NNS pathway. Recent work suggests that Nrd1-dependent termination of noncoding RNAs is necessary for transcriptome surveillance by regulating promoter directionality and suppressing antisense transcription [48]. Nrd1 interacts with the exosome and the Nrd1 CTD-interacting domain (CID) has been shown to be important for coupling of NNS-termination with RNA processing [7,8]. The interaction between Nrd1 and Rrp6 is direct and independent of the Nrd1 CID [49]. It has previously been shown that nuclear depletion of Nrd1 causes defective termination of many NNS pathway targets resulting in the identification of 1,526 extended ncRNAs referred to as NUTs (Nrd1 unterminated transcripts) [48]. The generation of NUTs upon Nrd1 nuclear depletion caused transcriptome-wide changes in gene expression predominately through transcription interference caused by defective termination. However, it was reported that deletion of *RRP6* does not change ncRNA transcription termination and disruption of Nrd1 and Rrp6 activities did not cause differential expression of the same genes, suggesting that Rrp6 is not required for NNS-dependent termination [48]. Interestingly, deletion of *RRP6* has recently been shown to both stabilize and promote elongation of CUT281. CUT281 is an antisense long noncoding RNA that regulates the expression of *PHO84* through antisense transcription past the *PHO84* promoter [50]. The catalytic subunits of the exosome (Rrp6 and Dis3) have also been implicated in termination at snR4 and snR34 [17]. These data suggest that Rrp6 can regulate both degradation and the efficiency of NNS-dependent termination of ncRNA transcripts such as CUTs that are implicated in mRNA regulation through transcription interference. Together, these studies suggest that Rrp6 may be required for NNS termination at some specific targets while dispensable at others. Our primary objective was to determine if Rrp6 is required for NNS termination through analysis of global changes in the transcriptome *via* high throughput RNA-sequencing (RNA-Seq) and analysis of global RNAPII occupancy changes using high-resolution chromatin IP followed by exonuclease treatment (ChIP-exo).

In this study, we show that deletion of *RRP6* disrupts 3’ end processing and/or termination at multiple NNS-dependent transcripts resulting in extended 3’ RNA ends. Many of the *rrp6Δ* RNA extensions cause transcription interference from extended transcription, and closely match the transcripts observed following nuclear depletion of Nrd1 further supporting that they are NNS-dependent transcripts. We find altered transcript lengths all classes of NNS-terminated transcripts including snRNAs, CUTs, SUTs, and a subset of mRNAs resulting in transcriptome-wide changes in gene expression. These data suggest that Rrp6 acts with the NNS complex to promote transcription termination in addition to coupled 3’ end RNA processing (for mRNAs, snRNAs, and select SUTs) and/or degradation (for CUTs).

## Results

### Genome-wide analysis of *RRP6* deletion strains by RNA-Seq

**Fig 1:**
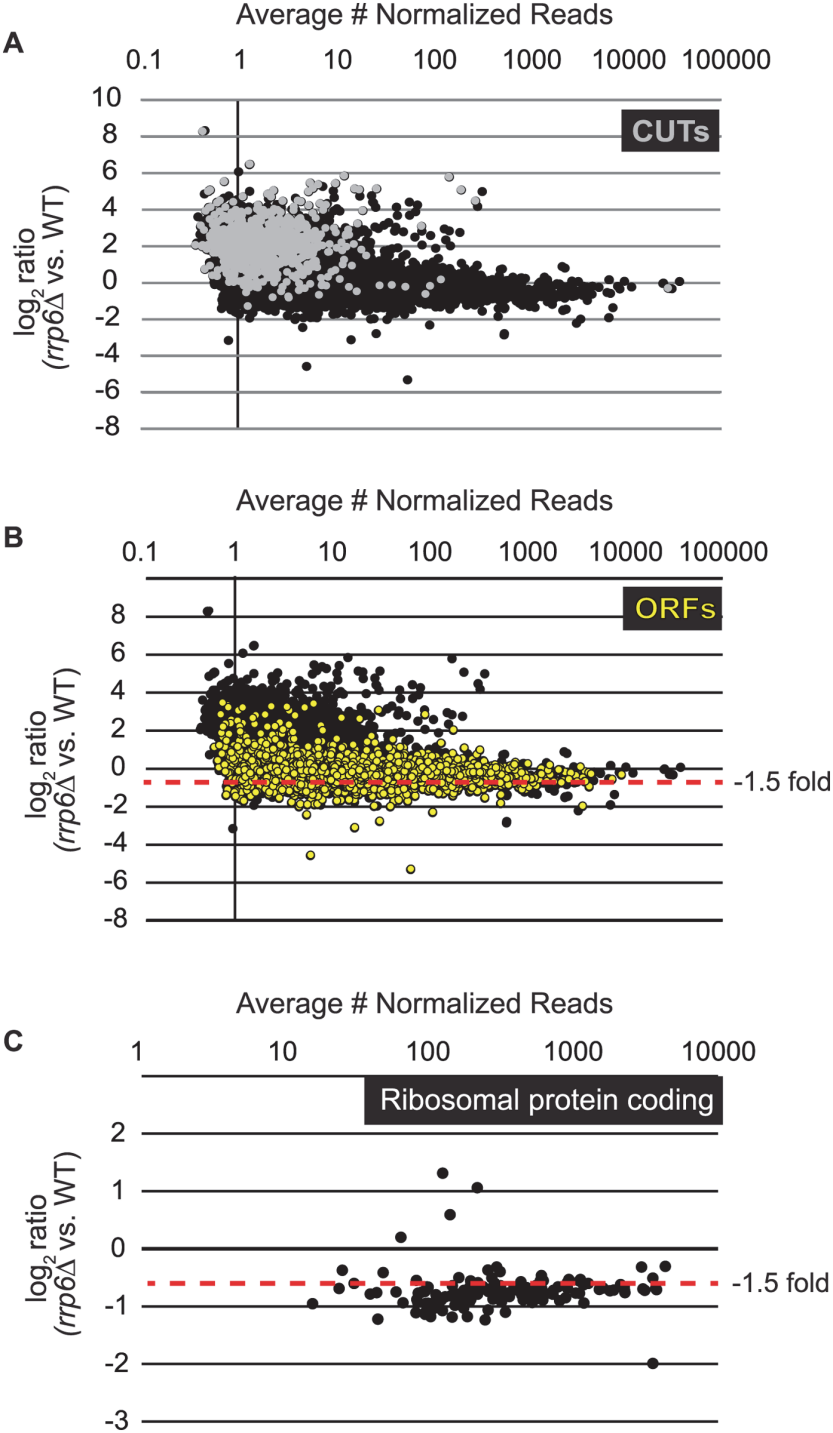
Expression plots for normalized RNA-SEQ data with specific classes of RNA transcripts highlighted. After sequencing reads were aligned to the yeast genome, reads mapped to annotated open reading frame transcripts (ORF-Ts), cryptic unstable transcripts (CUTs), stable unannotated transcripts (SUTs), and Nrd1 unterminated transcripts (NUTs) were used for differential expression analysis using edgeR. Log_2_ of the fold-change values are plotted versus the average number of normalized reads across all biological replicates for all RNA transcripts in cells lacking *RRP6* compared to WT (black dots). (A) RNAs annotated as CUTs, a classification based on the dependence of *rrp6Δ* for detection are shown as gray dots while all other transcripts are shown as black dots. (B) RNAs annotated as ORF-Ts, most of which are protein coding messenger RNAs are shown in yellow. All other transcripts are shown in black. (C) Messenger RNA expression values for ribosomal protein coding genes, shown as black dots.

To identify classes of transcripts affected by the loss of Rrp6, we utilized previously published annotations for yeast transcripts and performed differential expression analysis using normalized sequencing reads through EdgeR (S1 Table). To accurately represent the entire transcribed region for mRNAs including the coding region, we employed annotations that include both the 5’ and 3’ untranslated region (UTR) for the majority of the yeast transcriptome [51]. In summary, the annotations used for our study include 5792 open reading frames (ORF-Ts), 658 CUTs, 648 SUTs, 1215 NUTs, 844 Rrp6-regulated antisense transcripts, 80 snRNAs, and 78 snRNA extended transcripts (ETs, manually annotated in this study). In *rrp6Δ* cells, 136 up-regulated and 734 down-regulated open reading frame transcripts were identified (ORF-Ts, fold change cut-off = +/- 1.5, p-value < 0.05, FDR ≤ 0.1, Fig. 1A-B, S1 Table). We also identified 622 up-regulated CUTs out of 733, in agreement with previous reports that CUT expression and stability is increased in the absence of Rrp6 ([9,11,13], Fig. 1A, S1 Table). Importantly, using deep sequencing technology many CUTs were detected that have not previously been detected in WT cells using microarrays (S1 Table) [9,10,11,13]. Analysis of the NUTs revealed that the majority of that class of transcripts, 887/1215, were also up-regulated in *rrp6Δ* (S1 Table, S1A Fig.). Additionally, our analysis found that 223/648 SUTs were significantly up-regulated in *rrp6Δ* uncovering the specific SUT transcripts that are likely terminated by the NNS pathway (S1 Table). In *rrp6Δ* cells, a total of 54 sn/snoRNAs showed significant transcript extension (S1 Table, annotated as ETs—extended transcripts).

Considering that Rrp6 is an exonuclease, it is expected that the majority of RNA expression changes in *rrp6Δ* cells would be due to accumulation. However, we were surprised to see 734 mRNAs significantly decreased ≤ 1.5-fold in *rrp6Δ* (Fig. 1B, ORFs in yellow, S1 Table). To better understand the classes of mRNAs down-regulated in *rrp6Δ*, we performed GO-term enrichment analysis to determine if any cellular pathways showed significant enrichment within the set of decreased mRNAs. Surprisingly, the most enriched GOterm was GO:0005830 for the cytosolic ribosome with a p-value of 6.43E^-82^ (S2 Table). In total, nine of the 30 statistically significant enriched GO-terms related to ribosomal protein coding genes with p-values less than or equal to 7.85E^-19^ (S2 Table). Differential expression analysis determined that transcript levels of 109 out of 137 ribosomal protein coding genes were decreased more than 1.5-fold (Fig. 1C, S3 Table). Interestingly, only 3 ribosomal protein-coding genes had more than 1.5-fold increase in expression (Fig. 1C). It is also interesting to note that the average transcript length from the down-regulated ribosomal protein-coding mRNAs is 916 nucleotides putting many of the transcripts within the approximate length limits for the NNS pathway.

**Fig 2:**
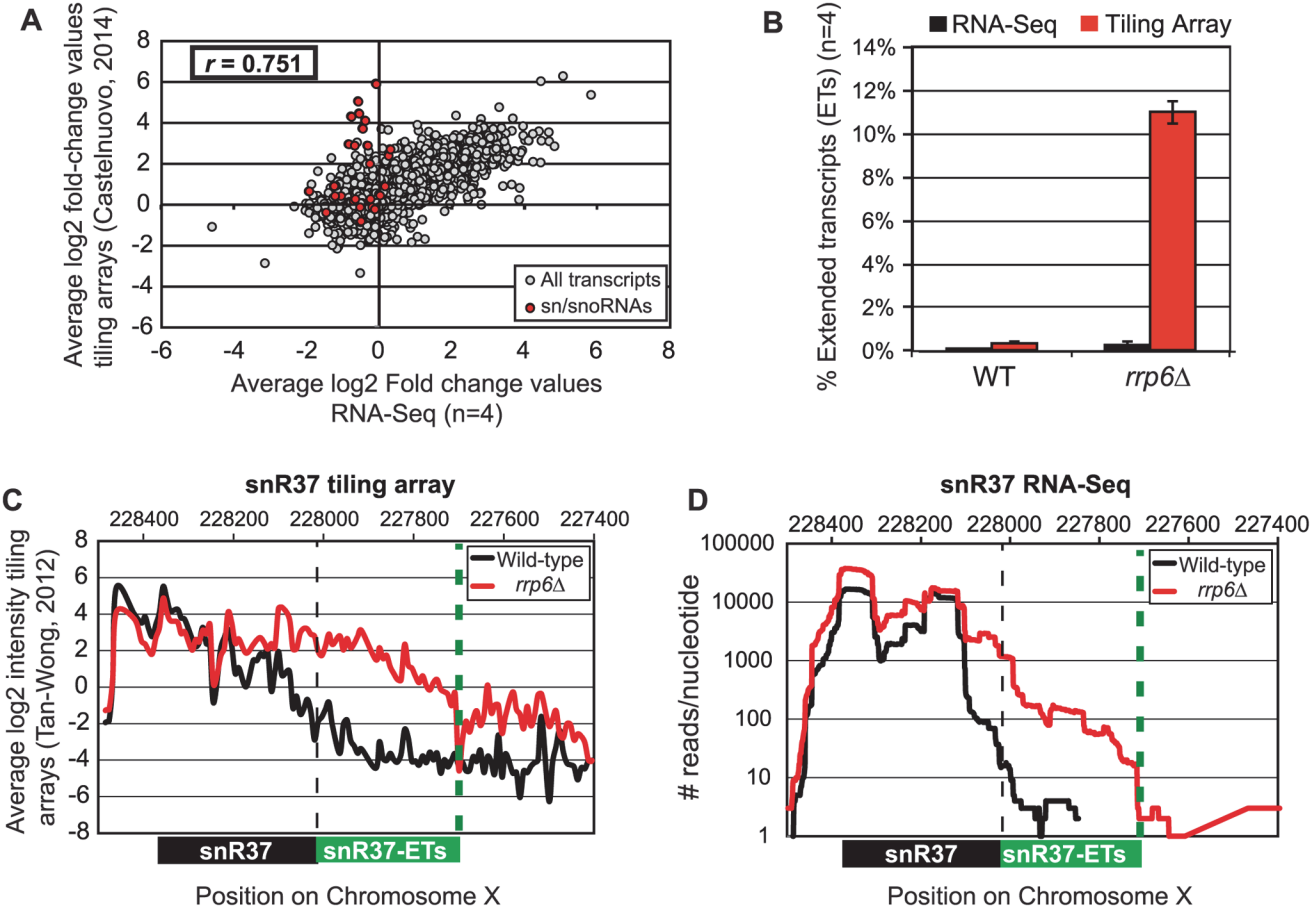
Comparison of highly abundant sn/snoRNA transcripts from *rrp6Δ* and WT strains obtained through tiling array or RNA sequencing. (A) Comparison of a recent *rrp6Δ* tiling array dataset [51] and RNA-sequencing data collected in this study by plotting the average log_2_ ratio values (*rrp6Δ* / WT) for all transcripts. All annotated transcripts included in both data sets are represented by gray circles. All sn/snoRNAs are highlighted in red circles. The Pearson correlation coefficient (*r*) between these datasets = 0.751, indicating a modest positive correlation between the two data sets. (B) Area under the curve calculation for snR37 extended transcripts (labeled as “ET”) from wild-type and *rrp6Δ* strains. The area calculated for snR37-ET annotation was divided by the area calculated for the entire snR37 to snR37-ET annotation to calculate the percentage of the entire transcript area located in the snR37-ET region (see diagram under (C) and (D) for locations of annotations). Values are shown as averages ± standard deviations for sequencing data are in black, tiling array data is in red. (C) Previously published tiling array intensity values [59] at snR37 using probe mid-position (8 nucleotides apart), for comparison with (D) mapped reads from RNA-Seq data at snR37 (single nucleotide resolution). Tiling array data represented in log_2_ scale. RNA-Seq data represented in log_10_ scale. In both graphs, wild-type RNA levels are shown in black, and *rrp6Δ* RNA levels are in red. Locations of annotated snRNA transcript (shown in black) and the extended transcript region annotated in this study (shown in green) are drawn to scale on each plot.

Pearson correlation coefficients were calculated comparing the average log_2_ fold-change values obtained in our RNA-Seq dataset (n = 4) and in a recent tiling array study by Castelnuovo *et al* 2014 [52,53]. The correlation coefficient when comparing these two datasets is 0.751 (Fig. 2A), a value indicating that there is a strong positive correlation between the two datasets similar to the extent of correlation previously found when comparing RNA-Seq to the tiling array platform [54]. Interestingly, the highly abundant sn/snoRNA transcripts had poor correlation across the two platforms, represented as red dots in Fig. 2A (values also given in S4 Table). It has previously been shown that RNA-Seq has a capacity for much greater dynamic range than microarrays, which can be saturated by very abundant transcripts and may not be able to quantitatively detect transcripts expressed at low levels [56]. For this reason, we propose that the differences seen between our dataset and previously published tiling array data are due to the increased dynamic range obtained with deep transcriptome coverage obtained by our RNA-Seq experiments. Considering that sn/snoRNAs are highly abundant and that they are one of the primary cellular targets of Rrp6 activity [14–16], the ability to accurately measure the abundance of snRNAs in this instance is a distinct advantage of using RNA-Seq. To compare the detection and quantification of extended 3’ ends of a selection of snRNAs in WT versus *rrp6Δ* cells, area-under-the-curve values were calculated for snR33 and snR37 using our RNA-Seq data compared to data from previously published tiling array dataset (Fig. 2B-D, [52]). To estimate the degree of extended transcript accumulation in *rrp6Δ*, the ET area was normalized against the area under the curve for the entire transcript resulting in a percent of extended transcript value (Fig. 1B). For snR37 and snR33, which have extended 3’ ends, there are significantly more extended reads in the *RRP6* mutant than in wild-type in both the sequencing and array data sets (Fig. 2). This agrees with the known role of Rrp6 to trim 3’ ends of snRNAs after termination. However, the relative ratios of these extended transcripts to the sense transcript appear to be much higher in the tiling array data than in the sequencing data in RNA isolated from *RRP6* deletion strains. For instance, approximately 11% of snR37 transcripts appeared extended in the tiling arrays dataset as opposed to 0.3% when analyzed by RNA-Seq (Fig. 2B). RNA sequencing read count values in our study span 5 orders of magnitude while the tiling array data only covers 3–4 orders of magnitude, likely causing a loss of accurate detection of highly abundant fully processed sn/snoRNAs (compare Fig. 2C and Fig. 2D). The highly abundant processed snR37 or snR33 peaks are clearly distinguished from extended products using RNA-Seq (Fig. 2). Our data suggests that the relative effects of the loss of *RRP6* on the steady state levels of these extended transcripts is much less dramatic than previously described and that accurate quantitation of steady state levels of the processed snRNA upon loss of *RRP6* was not previously obtained due to limited dynamic range.

### Analysis of snRNA termination in *RRP6* deletion mutants

Loss of *RRP6* results in improper 3’ end processing of multiple sn/snoRNAs. Such 3’ end processing defects could cause instability of snRNAs that leads to changes in their overall expression levels in cells [57,58]. Based on our differential expression analysis, 24 of the 78 sn/snoRNAs show decreased expression of at least 1.5-fold (log_2_ ≥ -0.6) in *rrp6Δ* versus wild-type cells with false discovery rates of ≤ 0.1 (S2 Table). To better understand the role of Rrp6 in global snRNA 3’ end processing, the length and intensity of snRNA extended transcripts was analyzed in *rrp6Δ* cells through manual annotation of the extended transcripts (annotated as “ETs”) observed in our RNA-Seq dataset (S1 Table). The length of snRNA transcript extension was compared to the annotated length of Nrd1-unterminated transcripts (NUTs) and cryptic unstable transcripts (CUTs) [11,48]. Additionally, we performed ChIP-exo according to established protocols to generate high-resolution maps of RNAPII localization throughout the yeast genomes in wild-type and *RRP6* deletion cells with Rpb3-FLAG strains made for this study (S1B Fig.) [55]. It was recently reported that NUT annotations were significantly longer than CUT annotations, suggesting that Rrp6 is required for 3’ end processing of NNS terminated transcripts but is not directly involved in NNS termination [48]. Comparison of our datasets from RNA sequencing and ChIP-exo to NUT annotations as well as direct comparison of specific transcripts in *rrp6Δ* cells to NNS pathway mutants via northern blot analysis allowed us to discover a requirement for Rrp6 in Nrd1-dependent RNAPII termination.

**Fig 3:**
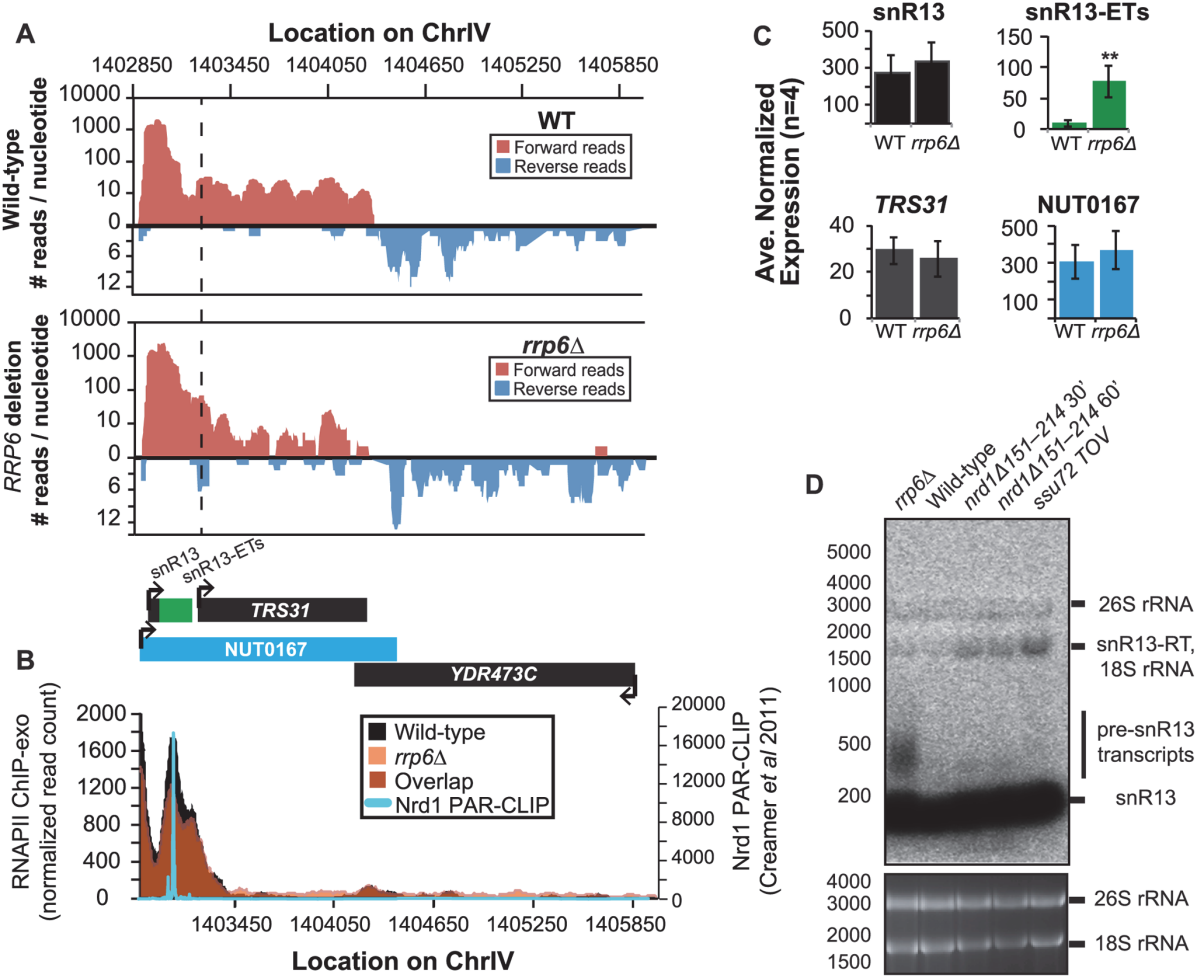
Termination of the C/D box small nucleolar RNA snR13 transcript does not require Rrp6. (A) Graphical representation of strand-specific RNA-seq reads mapped to snR13-*YDR473C* region. Reads mapped to the positive strand are on top in red, while reads mapped to the negative strand are on the bottom in blue. The location and direction of transcription for all analyzed annotations are diagrammed below the graphs to scale. Processed length of snRNAs and mRNAs are in black, snRNA-extended transcripts, including pre-snRNAs and termination read-through products, are in green (labeled “ETs”), NUTs are in aqua, and arrows indicate direction of transcription. The dotted black line marks the transcription start site (TSS) of *TRS31*. (B) Rpb3-FLAG localization as determined by ChIP-exo sequencing reads mapped to the same region and aligned to (A). Wild-type normalized read counts are in black, and *rrp6Δ* are in orange. Nrd1 binding sites as determined by PAR-CLIP from Creamer *et al* [39] are shown for comparison in aqua. (C) Average normalized read counts ± standard deviations for significantly altered transcripts in *rrp6Δ* versus wild-type (n = 4). Two stars represent a p-value of <0.01 as determined by an unpaired, two-tailed student’s t-test. The colors of the bars in each graph correspond to the color representing the related annotation. (D) Strand-specific northern blot analysis using a 5’ end labeled DNA oligonucleotide probe specific to the processed region of snR13 directly comparing *rrp6Δ* to mutants known to be defective in Nrd1-dependent termination. The temperature sensitive *nrd1Δ151–214* strain was grown at 30°C overnight, diluted to an OD_600_ of 0.5, and grown at 37°C for 30 minutes or 60 minutes as indicated. The *ssu72 TOV* strain has been previously shown by Loya et al. [60] to bypass Nrd1-dependent termination at the *IMD2* locus. The 26S and 18S ribosomal RNAs are shown as a loading control (bottom).

It has previously been reported that some snRNAs are significantly longer in Nrd1-depleted cells than in *rrp6Δ*, and our dataset confirms these findings. Previously published data by Kim *et al*. has shown a large extension for snR13 in *SEN1* and *NRD1* mutant strains, with the snR13 transcript extended across the coding region of *TRS31* [20]. This extension at snR13 has also been reported in *SSU72* mutant strains [52,56,57]. NUT0167 was annotated as an Nrd1-dependent unterminated transcript at snR13 following Nrd1 nuclear depletion. Kim *et al*. identified both normal length and an extended snR13 transcript in *rrp6∆* cells, which corresponds to the pre-snRNA transcript that is not correctly processed in the absence of Rrp6 by northern blotting. For direct comparison of our dataset to previous work, we compared our RNA-Seq and ChIP-exo results to northern blot analysis using a strand specific probe against the processed version of snR13 (Fig. 3). The extended transcript detected in Nrd1 nuclear depletion experiments was annotated as NUT0167 and is 1378 nucleotides longer than the pre-snR13 transcript observed in our *rrp6∆* RNA-Seq samples (Fig. 3A). In agreement, we observed a transcript approximately 1500 nucleotides long by northern blotting in the *NRD1-*temperature sensitive *(ts)* mutant (*nrd1Δ151–214* that lacks the Nab3 interaction domain [34,58,59]) following 30 or 60 minute heat shock. Long extended transcripts were also observed in *ssu72 TOV*, a “terminator override” mutant previously shown to be deficient in Nrd1-dependent termination (kindly provided from the Reines lab) [60]. However, no such read-through transcript is observed in the *rrp6∆* cells by RNA-Seq or by northern blotting, only the unprocessed pre-snR13 (Fig. 3A, D). Supporting the RNA transcript data, we see no shift in RNAPII localization in this region as detected by Rpb3-FLAG ChIP-exo sequencing (Fig. 3B). Previous studies to identify Nrd1 RNA binding sites using PAR-CLIP sequencing revealed a strong Nrd1 signal just downstream of the annotation for the mature snR13 transcript supporting the role of the NNS pathway in termination in this region ([39], Fig. 3B). No significant changes were observed in the expression levels of snR13 or *TRS31*, which also suggests that snR13 is correctly terminated in *rrp6∆* (Fig. 2C). Taken together, these data clearly show that Rrp6 is not required for termination through the NNS pathway at snR13 in agreement with previous findings.

**Fig 4:**
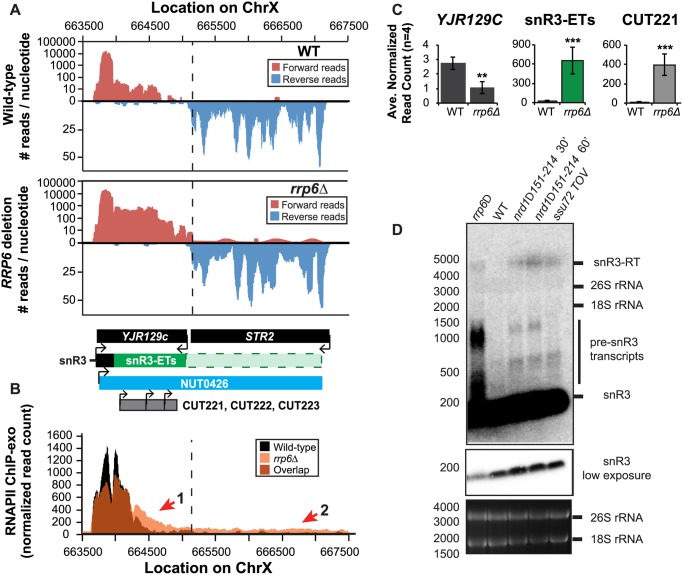
The H/ACA box small nucleolar RNA snR3 requires Rrp6 for efficient termination. (A) Graphical representation of strand-specific RNA-Seq reads mapped to snR3-*STR2* region. Reads mapped to the positive strand are on top in red, while reads mapped to the negative strand are on the bottom in blue. The location and direction of transcription for all analyzed annotations are diagrammed below the graphs to scale. Processed length of snRNAs and mRNAs are in black, snRNA-extended transcripts, including pre-snRNAs and termination read-through products, are in green (labeled “ETs”), an additional region of extended transcript that was not annotated due to its low abundance is shown as a dashed green box, NUTs are in aqua, CUTs are in gray, and arrows indicate direction of transcription. The dotted black line marks the transcription start site (TSS) of *YJR129C*. (B) Rpb3-FLAG localization as determined by ChIP-exo sequencing reads mapped to the same region and aligned to (A). Wild-type normalized read counts are in black, and *rrp6Δ* normalized read counts are in orange. (C) Average normalized read counts ± standard deviations for significantly altered transcripts in *rrp6Δ* versus wild-type (n = 4). Two stars indicate a p-value of <0.01, and three stars indicate a p-value of <0.001 as determined by an unpaired, two-tailed student’s t-test. The colors of the bars in each graph correspond to the color representing the related annotation. (D) Strand-specific northern blot analysis using a 5’ end labeled DNA oligonucleotide probe specific to the processed region of snR3 directly comparing *rrp6Δ* to mutants known to be defective in Nrd1-dependent termination. Transcripts of interest are indicated to the right of the figure. The 26S and 18S ribosomal RNAs are shown as a loading control (bottom).

However, in contrast to previous conclusions that Rrp6 is dispensable for snRNA termination, our data indicates that a subset of snRNA transcripts require Rrp6 for proper RNAPII termination *in vivo* [20,48]. As shown in Fig. 4A, the apparent length of snR3 in *rrp6Δ* cells as revealed by RNA-Seq is 1396 nucleotides whereas the annotation for NUT0426 is 3363 nucleotides long (Fig. 4A, S1 Table). However, the Rrp6-dependent snR3 extension is 246 nucleotides longer than the three tandem CUTs (CUT221, 222, and 223) previously annotated from tiling array data [52]. All three CUTs were significantly up-regulated in *rrp6Δ* as expected (Fig. 4B and S2 Fig.). Interestingly, a comparison of the 4tU-Seq data used to annotated the NUTs with our RNA-Seq data clearly shows a difference in the length of the Nrd1-unterminated transcript NUT0426 resulting from snR3 read-through than that observed upon deletion of *RRP6* (S2 Fig.). However, it is interesting to note that the overall read count for the snR3 read-through product in the 4tU-Seq dataset decreases dramatically just 3’ to the read-through observed in *rrp6Δ*, suggesting that the majority of transcripts are terminated at the location indicated in the *rrp6∆* RNA-Seq (Fig. 4, S2 Fig.). ChIP-exo analysis of RNAPII localization in this region revealed an increase in RNAPII density just downstream of snR3 in the *rrp6Δ* cells when compared to WT (Fig. 4B, arrow 1). This increase in downstream RNAPII corresponds to the increased level of RNA detected downstream of snR3 in Fig. 4A past the pre-snRNA transcript and previously mapped Nrd1 and Nab3 binding sites located ~400bp from the start of the snR3 transcript [39,47]. Although the RNAPII density decreases just before the 3’ end of the snR3-ETs annotation, it remains slightly elevated in *rrp6Δ* compared to wild-type throughout the NUT0426 annotation region, indicated by arrow #2 (Fig. 4B). Differential expression analysis also revealed highly significant down-regulation of the snR3 convergent, “tail-to-tail,” gene *YJR129C* likely as a result of extended snR3 transcripts in *rrp6Δ* cells (Fig. 4C). These data suggest that extension of snR3 causes transcription interference at *YJR129C* as was also reported in Nrd1 nuclear depletion experiments [46]. To definitively determine the length of the snR3 transcripts and compare *rrp6∆* cells directly to an *NRD1* mutant, we also performed northern blot analysis with a probe that recognizes the short, processed snR3. A transcript that is approximately 4000 nucleotides long was detected in *rrp6Δ*, *nrd1-ts* mutants, and the *ssu72 TOV* (Fig. 4D). This transcript is approximately the length expected for NUT0426. We also detected two shorter transcripts approximately 500 and 1000 nucleotides long in the *nrd1-ts* and *rrp6Δ* cells. Together, these data clearly show that Rrp6 is required for proper RNAPII termination at snR3.

### *RRP6* deletion results in extension of snRNA transcripts leading to down-regulation of neighboring genes

**Fig 5:**
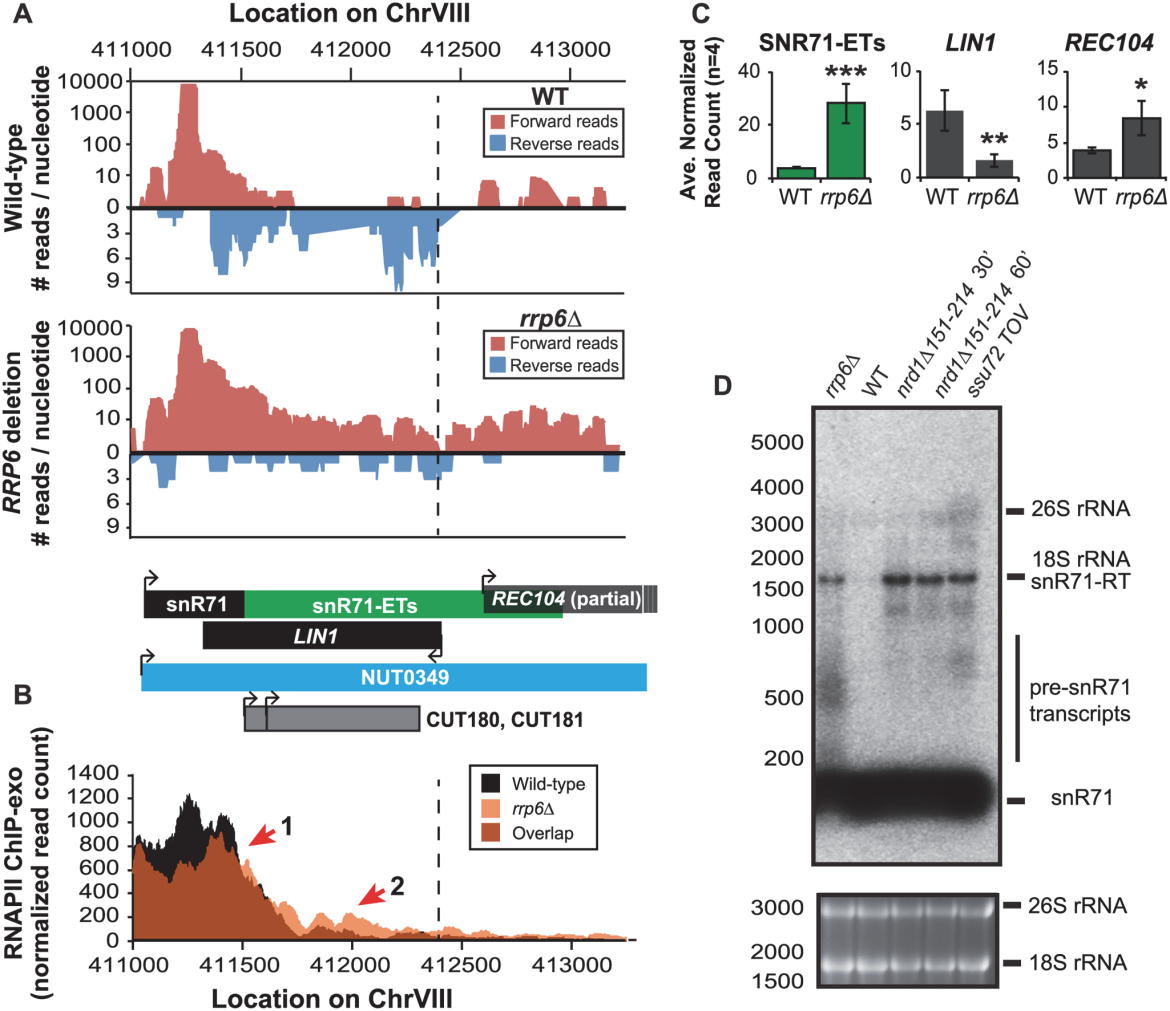
Rrp6 is required for NNS-dependent termination and RNA processing of the snR71 transcript. (A) Graphical representation of strand-specific RNA-seq reads mapped to snR71-extended region. Reads mapped to the positive strand are on top in red, while reads mapped to the negative strand are on the bottom in blue. The location and direction of transcription for all analyzed annotations are diagrammed below the graphs to scale. Processed length of snRNAs and mRNAs are in black, snRNA-extended transcripts, including pre-snRNAs and termination read-through products, are in green (labeled “ETs”), NUTs are in aqua, CUTs are in gray, and bent arrows indicate direction of transcription. The dotted black line marks the transcription start site (TSS) of *LIN1*. (B) Rpb3-FLAG localization as determined by ChIP-exo sequencing reads mapped to the same region and aligned to (A). Wild-type normalized read counts are in black, and *rrp6Δ* are in orange. Red arrows indicate areas of discussion in the text. (C) Average normalized read counts ± standard deviations for significantly altered transcripts in *rrp6Δ* versus wild-type (n = 4). One star indicates a p-value of <0.05, two stars indicate a p-value of <0.01, and three stars indicate a p-value of <0.001 as determined by an unpaired, two-tailed student’s t-test. The colors of the bars in each graph correspond to the color representing the related annotation. (D) Strand-specific northern blot analysis using a 5’ end labeled probe specific to the processed region of snR71 directly comparing *rrp6Δ* to NNS termination mutants. The 26S and 18S ribosomal RNAs are shown as a loading control (bottom).

Several genes downstream of snRNAs transcribed from the opposite DNA strand were found to be significantly down-regulated when transcript extension was high in *rrp6Δ* cells. We focused on snRNAs with “tail-to-tail” or convergent genes because the strand specific sequencing data allows us to distinguish between reads mapped to the two different transcripts. As mentioned in the example of snR3, the expression of neighboring downstream gene *YJR129C* is down almost three-fold (Fig. 4C). The detectable snR3 transcript maps beyond the transcription start site (TSS) for *YJR129C* suggesting that transcription interference is the likely mode of *YJR129C* repression as diagrammed in S3A Fig. Additional examples of snRNA termination defects causing transcription interference at downstream genes are shown in Fig. 5 and S3 Fig. As shown in S3 Fig., snR11 has a downstream gene transcribed on the opposite strand, *CMC4* (S3C Fig.). snR11 has a long region of RNA-seq reads in *rrp6Δ* cells that extends beyond the *CMC4* TSS, and *CMC4* expression is decreased by more than half (S3C Fig. and S1 Table). However, northern blot analysis with a single stranded oligonucleotide probe detecting the short, processed snR11 does not show read-through transcripts >1000 bp in any of the samples, including the mutants known to have defective Nrd1 termination (S3B Fig.). Since there is no detectable read-through snR11 transcript in the *nrd1-ts* mutant, the transcript encoded in the region annotated “snR11-ETs” and “NUT0607” is likely initiated at start site downstream of the snR11 sequence recognized by our probe. The lack of highly extended transcripts, readily distinguishable by northern blot, shows the limitation of using short sequencing reads for transcript annotation (S3 Fig.). Interestingly, Rpb3-FLAG ChIP-exo data shows that the peak of polymerase localization at snR11 is extended farther 3’ in *rrp6Δ* cells than in wild type cells indicated by arrow #1, and this extension does indeed overlap the TSS of *CMC4* (S3D Fig.). Polymerase occupancy quickly decreases just beyond the *CMC4* TSS but remains higher than in wild type cells. The increase in polymerase localization in *rrp6Δ* downstream of the native termination site for snR11 suggests that loss of *rrp6Δ* decreases the efficiency of snR11 termination leading to a small degree of read-through that is sufficient to cause transcription interference at *CMC4*.

A substantial read-through transcript (1442 nucleotides) was detected at snR71, a C/D box small nucleolar RNA gene, in *rrp6Δ* cells that extends well beyond the TSS of downstream gene *LIN1*, which is significantly down-regulated (Fig. 5A, C) [61,62]. There is a 2281 nucleotide long NUT annotated at this locus (NUT0349) that overlaps with a second downstream gene, *REC104*, which is on the same strand as snR71 (Fig. 5A). With strand specific sequencing, it cannot be absolutely determined if reads mapped to the *REC104* locus are from *REC104* transcripts, from a much longer snR71 read-through transcript, or from a combination of both. To answer this question and compare the effects of *RRP6* deletion to mutants with defective Nrd1-termination directly, we performed northern blot analysis with a short probe specific to the processed snR71 transcript (Fig. 5D). We see a striking band at approximately 1700 nucleotides in *rrp6Δ*, *nrd1-ts*, *and Ssu72 TOV* cells. This suggests that a percentage of RNAPIIs transcribing snR71 in all of these mutant strands can escape the Nrd1-termination pathway and are hence terminated much farther downstream (Fig. 5). This is supported by the Rpb3-FLAG ChIP-exo data that shows an increase in polymerase localization downstream of snR71 in *rrp6Δ* cells compared to wild type cells along the full length of the NUT0349 annotation (Fig. 5B). Interestingly, the expression level of the annotated mature snR71 region is also significantly decreased (Fig. 5C), suggesting there may be some instability or other defects caused by improper 3’ processing and/or termination caused by loss of Rrp6.

Overall these data show that multiple transcripts downstream of snRNAs have significant changes in expression following deletion of *RRP6*. ChIP-exo analysis of RNAPII (Rpb3-FLAG) clearly show that RNAPII terminates downstream of its normal stopping point in WT cells at specific snRNAs leading to mislocalization and causing transcription interference at downstream genes (Figs. 4 5, and S3 Fig.). In this model, ineffective termination of the snRNAs results in transcription interference at the downstream gene as previously described following inactivation of Nrd1 (S3A Fig. [46]). These findings, taken together with the similar lengths of Rrp6-dependent snRNA transcripts and Nrd1-dependent transcript annotations (NUTs), support the hypothesis that Rrp6 serves as important regulator of NNS-dependent termination at specific sn/snoRNAs.

### *RRP6* is required for proper RNAPII termination of NNS-dependent regulatory non-coding RNAs

**Fig 6:**
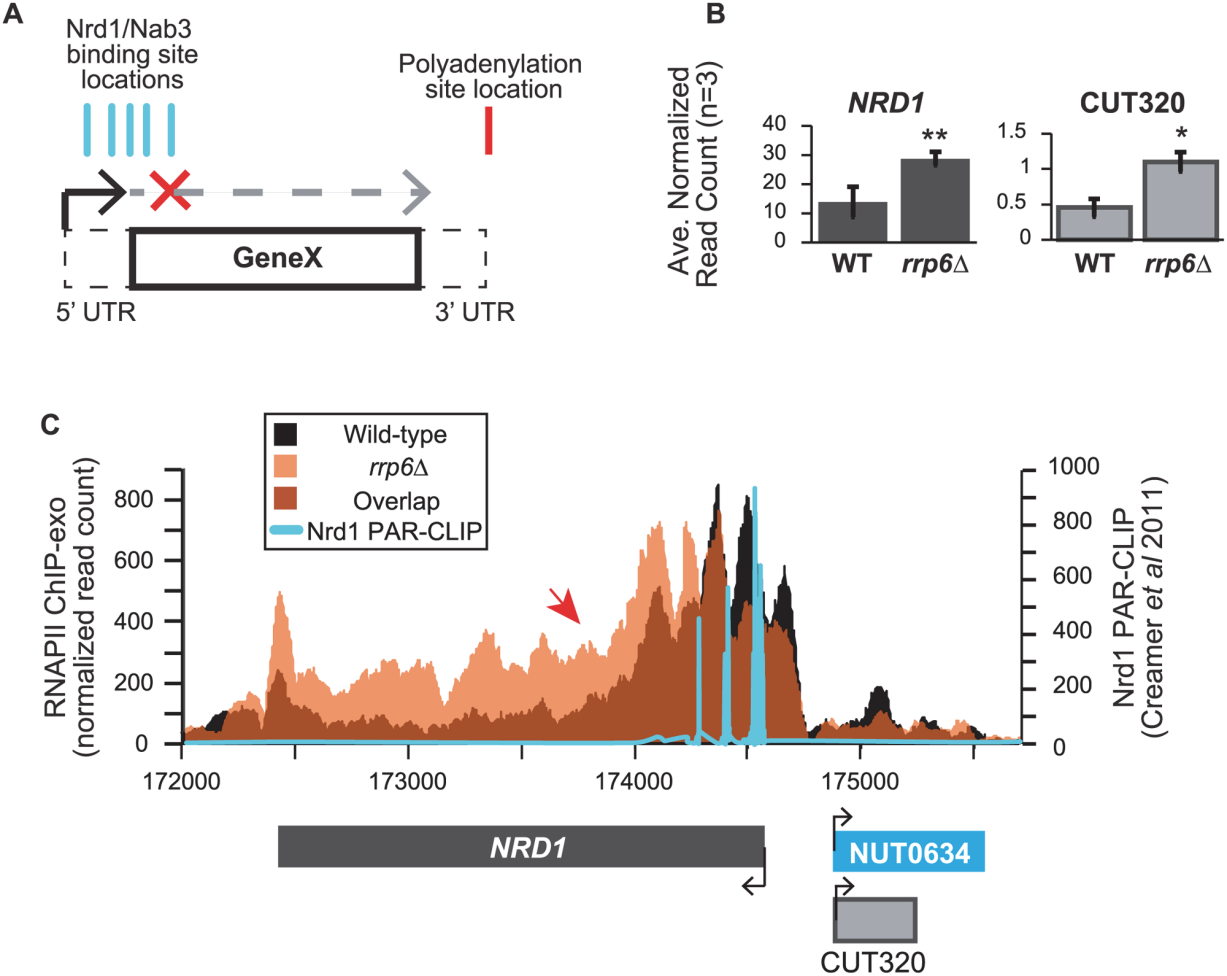
*NRD1* early termination requires Rrp6 for efficient RNAPII termination. (A) Early termination model of NNS-dependent transcription attenuation. A hypothetical gene ‘Gene X’ is shown with the coding region (solid lines) and UTRs (dashed lines) drawn. Locations of Nrd1 or Nab3 binding sites (aqua) or the Polyadenylation site (red) are drawn above the figure. In brief, early termination by the NNS pathway prevents transcription of the full length gene. (B) Average normalized read counts ± standard deviations for *NRD1* and CUT320 transcripts in *rrp6Δ* versus wild-type (n = 3). One star indicates a p-value of <0.05 and two stars indicate a p-value of <0.01. (C) Rpb3-FLAG localization as determined by ChIP-exo sequencing reads mapped to the *NRD1* region. Wild-type reads are in black, and *rrp6Δ* are in orange. The location and direction of transcription for all analyzed annotations are diagrammed below the graphs to scale. Length of mRNAs including untranslated regions are in black, CUTs are in gray, and NUTs are in aqua. Nrd1 binding sites as determined by PAR-CLIP from Creamer *et al* [39] are shown in aqua for comparison. The scale for Nrd1 PAR-CLIP data is shown to the right.

In addition to extension of snRNA transcripts, changes in expression and/or apparent length of other previously described noncoding RNAs, such as CUTs, SUTs, and NUTs, were also detected with corresponding down-regulation of neighboring genes in cells lacking Rrp6 suggesting transcription interference. A number of these transcripts have been well-characterized as extended following depletion or genetic inactivation of NNS components but not the exonuclease *RRP6*. An early and well-characterized example of a gene that is regulated by Nrd1-dependent termination is the *NRD1* gene itself [30,63,64]. There are a cluster of multiple Nrd1 and Nab3 binding sites in the 5’ UTR of the Nrd1 mRNA leading to early termination of the transcript and autoregulation of Nrd1 expression levels through a mechanism that also requires Sen1 [19,38,39,64]. In expression analysis of the RNA-seq data, we see nearly a 2-fold increase in the expression of the *NRD1* transcript in *rrp6Δ*, as well as an increase in CUT320, a noncoding transcript near the *NRD1* promoter on the opposite strand (Fig. 6A). For comparison, Nab3 mRNA levels were not significantly changed by *RRP6* deletion (S1 Table). Since Rrp6 is responsible for the degradation of the short *NRD1* transcript, this increase in expression could be attributed to stabilization of terminated transcript. Alternatively, increased expression of *NRD1* in *rrp6Δ* cells could suggest that the early termination sites are not being utilized by the Nrd1 pathway as often in the *rrp6Δ* cells leading to transcription of the full-length mRNA. Analysis of RNAPII occupancy at the *NRD1* gene in the ChIP-exo dataset can distinguish these two possibilities (Fig. 6B). In wild type cells, there are several peaks of RNAPII at the 5’ end of the *NRD1* gene that quickly decreases to low levels along the *NRD1* coding region in agreement with previous findings in an Nrd1 mutant [64]. In fact, comparison of the ChIP-exo data with Nrd1 RNA binding sites mapped by PAR-CLIP reveal that the majority of RNAPII terminates just downstream of the final Nrd1 binding site in WT cells (Fig. 6B). In *rrp6Δ* cells, the intense peaks of RNAPII localization at the 5’UTR are shifted 3’ and RNAPII localization is higher along the entire length of the gene, including a higher peak at the poly-A dependent termination site of the full-length transcript (Fig. 6B). This supports the hypothesis that the NNS-termination pathway is not terminating the short 5’UTR transcript efficiently in *rrp6Δ* cells and that RNAPII continues down the length of the gene producing the full transcript and increasing overall *NRD1* expression levels (Fig. 6). The requirement for multiple Nrd1-Nab3 RNA binding sites at *NRD1* when compared to RNAPII ChIP-exo data suggest that NNS-dependent termination requires multiple Nrd1 and/or Nab3 binding sites to effectively terminate RNAPII as has been previously proposed (Fig. 6B) [27,39]. The 3’ shift in RNAPII localization observed at *NRD1* supports this hypothesis.

Changes in RNAPII occupancy downstream of known NNS-dependent early termination (also known as attenuated) targets was also observed at *HRP1* and *SRG1-SER3* (S4 Fig. and S5 Fig.). The RNAPII occupancy at *HRP1* shows similar changes as seen at *NRD1* with the majority of RNAPII terminating early in WT cells while showing a 3’ shift (arrow #1) and persistence through the coding region (arrow #2) in *rrp6Δ* (S4A Fig.). RNA-Seq analysis also revealed an increase in downstream *HRP1* RNA levels relative to WT (S4B Fig.). Another well-characterized example of upstream noncoding RNA regulation occurs at the *SRG1-SER3* region. *SRG1* (*SER3* regulatory gene 1) is a known non-coding RNA whose transcription down-regulates expression of *SER3* [65]. *SRG1* RNA is bound by both Nrd1 and Nab3 just prior to the *SER3* transcribed region, which could then terminate *SRG1* transcription to prevent interference with *SER3* [31,47]. SRG1 can also be terminated through a polyA-dependent pathway at a site downstream of the NNS termination site(s) [31]. ChIP-exo revealed that RNAPII occupancy also shifts 3’ in *rrp6Δ* cells with increased RNAPII occupancy in the *SER3* coding region (S5A Fig.). An increase in specific *SRG1* transcripts including a *SRG1*:*SER3* chimeric transcript has previously been observed in Nrd1-depletion *rrp6Δ* cells [31]. Our data suggests that defective termination of *SRG1* can occur in *rrp6Δ* cells even in the absence of Nrd1 disruption (S5 Fig.). These data clearly show that Rrp6 is required for NNS-dependent termination of regulatory non-coding RNAs that participate in gene expression attenuation.

**Fig 7:**
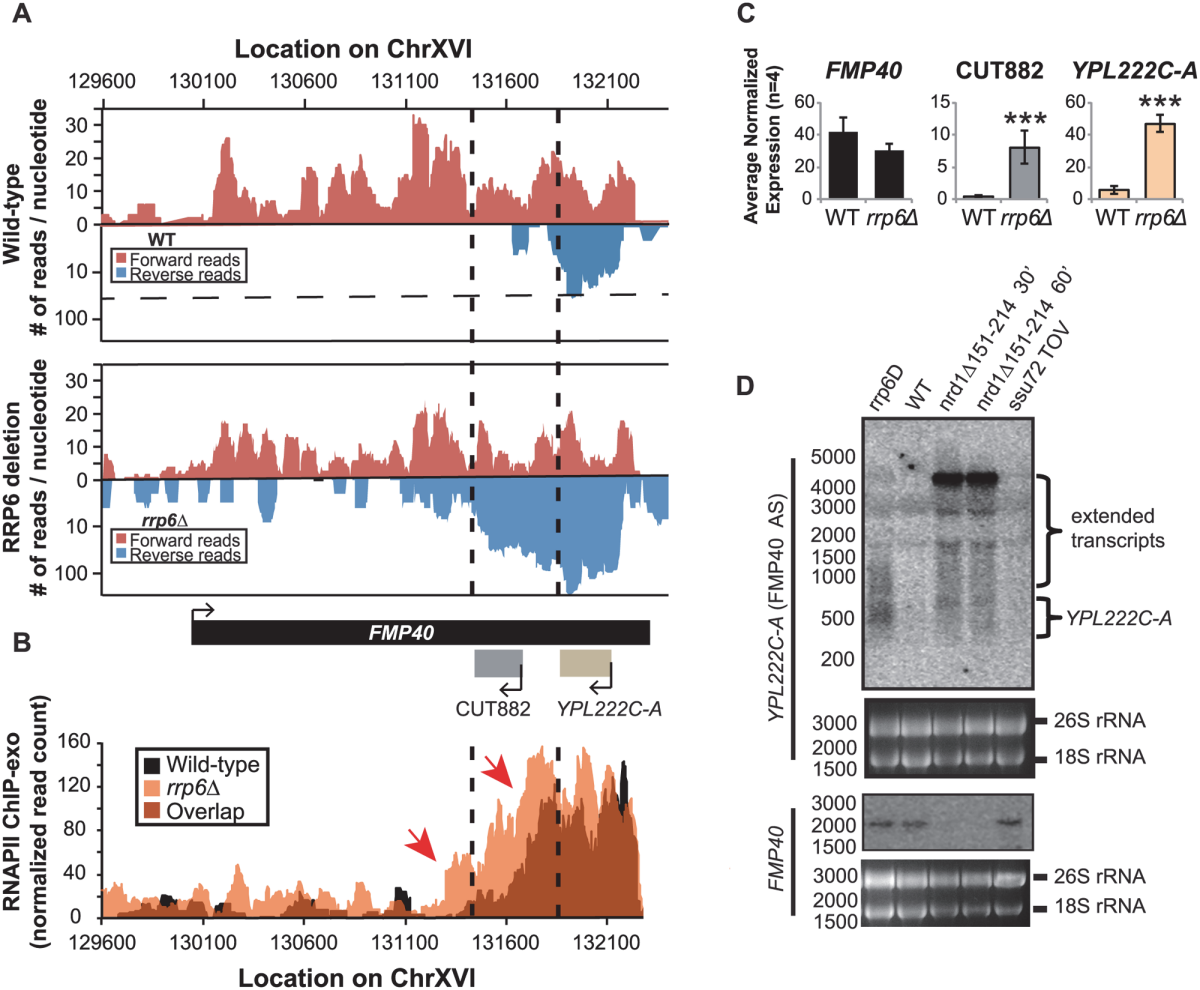
The *FMP40* antisense transcript *YPL222C-A* is extended in *rrp6Δ* deletion cells as a result of inefficient RNAPII termination. (A) Graphical representation of strand-specific RNA-seq reads mapped to *FMP40* region. Reads mapped to the positive strand are on top in red, while reads mapped to the negative strand are on the bottom in blue. The location and direction of transcription for all analyzed annotations are diagrammed below the graphs to scale. Length of mRNAs including untranslated regions are in black, CUTs are in gray, dubious ORFs (an antisense transcript named *YPL222C-A*) are in tan, and bent arrows indicate direction of the TSS. The dotted black lines mark the 3’ end of the annotations for CUT882 and *YPL222C-A*. (B) Rpb3-FLAG localization as determined by ChIP-exo sequencing reads mapped to the same region and aligned to (A). Wild-type reads are in black, and *rrp6Δ* are in orange. Red arrows note areas of interest. (C) Average normalized read counts ± standard deviations for significantly altered transcripts in *rrp6Δ* versus wild-type (n = 4). Three stars indicate a p-value of <0.001 as determined by an unpaired, two-tailed student’s t-test. The colors of the bars in each graph correspond to the color representing the related annotation. (D) Strand-specific northern blot analysis using a 5’ end labeled probes specific to either *FMP40* or *YPL222C-A* directly comparing *rrp6Δ* to mutants known to be defective in NNS termination. The 26S and 18S ribosomal RNAs are shown as a loading control (bottom).

There are multiple published examples of transcription interference by CUTs in which an antisense CUT regulates the expression of the sense gene in *cis* through transcript extension across the sense gene promoter [50,66,67,68,69]. These lncRNA-type transcripts have also been shown to be required for efficient gene activation [70]. Expression of the *FMP40* transcript has been previously shown by several groups to be regulated by an Nrd1-terminated antisense transcript initiating at the 3’ end of *FMP40* [28,39,71]. The *FMP40* antisense transcript (also known as CUT882 and *YPL222C-A)* is readily detectable in our RNA-sequencing data from WT cells and it is significantly upregulated in *rrp6Δ* cells as has been previously reported (Fig. 7A and C) [10,11,72,73]. Mapped sequencing reads suggest that the antisense transcript was significantly longer in *rrp6Δ* than in wild type (specifically in the region of CUT882), supporting the hypothesis that Nrd1-termination is not as efficient in *rrp6Δ* mutants. To determine the length of the antisense transcript in *rrp6Δ* and compare to an *nrd1*-ts mutant, we performed northern blot analysis with strand specific oligonucleotide probes to *FMP40* and its antisense transcript *YPL222C-A* (Fig. 7D). By northern blot, *YPL222C-A* transcripts are detected as smears extending into the region of the CUT882 annotation suggesting that these transcripts terminate at multiple 3’ end locations as has previously been shown for other specific CUTs including *NEL025c* [9]. The *YPL222C-A* antisense transcripts were not detected in WT cells by northern blot even after an extended exposure time (Fig. 7D). In the *nrd1*-ts mutant, there is an accumulation of a strong band ~4500 nucleotides long in the *YPL222C-A* blot, and the *FMP40* transcript is undetectable as a consequence of transcription interference. There is a band of the same size in the *rrp6Δ*, although it is not as abundant as observed in the Nrd1 mutant. There are also shorter transcripts present in *YPL222C-A* blots in the *nrd1-ts* mutant that are also present in the *rrp6Δ* cells (Fig. 7D). RNAPII localization in the *FMP40* region was determined by ChIP-exo and clearly shows that the majority of polymerase was localized at the 5’ end of the antisense *YPL222C-A* transcript, even in wild-type cells (Fig. 7B). The highest peaks of RNAPII localization at *YPL222C-A* are of similar intensity in WT and *rrp6Δ* cells, but polymerase spreads 3’ in *rrp6Δ* and continues to be higher through the CUT882 annotation and past the *FMP40* promoter. These data indicate that the antisense transcript is terminated less efficiently in *rrp6Δ* cells leading to increased RNAPII occupancy downstream of the normal *YPL222C-A*/CUT882 termination site. The RNAPII occupancy data also suggest that CUT882 is an extended transcript of *YPL222C-A* that occurs as a consequence of inefficient RNAPII termination in *rrp6Δ* (Fig. 7B, red arrows). In *nrd1Δ151–214* temperature sensitive mutants, *YPL222C-A* termination rarely occurs resulting in a 4500-nucleotide antisense transcript that is also seen at low levels in *rrp6Δ* (Fig. 7D).

## Discussion

Through comparison of RNA-Seq and RNAPII ChIP-exo datasets, these data clearly show that NNS-terminator read-through occurs at a significant number of NNS-target genes in the absence of the 3–5’ exonuclease Rrp6. These findings support the hypothesis that the NNS pathway requires Rrp6 function for both 3’ end processing and NNS-dependent termination of specific transcripts. Overall, these data show a striking similarity between the annotations for Nrd1 unterminated transcripts (NUTs) at many locations and the transcripts observed following deletion of the nuclear specific 3’-5’ exonuclease subunit Rrp6 genome-wide. The tight coupling of the NNS pathway with exosome function has been previously characterized; however our findings are the first to demonstrate on a large scale that Rrp6 function is required for termination through the NNS pathway at specific transcripts. We have also shown that deletion of *RRP6* causes changes in RNAPII localization that are indicative of termination defects. The comparison of changes in RNA transcript signals to actual changes in RNAPII occupancy allowed us to distinguish between effects of RNAPII termination events versus exonuclease-dependent RNA processing, which was of particular importance for this study. Although it has been proposed that Nrd1-unterminated transcripts (NUTs) aresignificantly longer than CUTs, our data clearly shows that specific NNS-dependent transcripts rely on Rrp6 for proper termination while others do not. For instance, snr71 transcripts show a requirement for Rrp6, Nrd1, and Ssu72 for termination, while snR13 does not require Rrp6 (Figs. 3 and 5). The cryptic transcript CUT882 that is associated with *FMP40/YPL22C-A* also shows a requirement for Rrp6 for termination but it appears that the CUT882 transcript is significantly longer in *nrd1∆151–214* temperature sensitive strains (Fig. 7). Previous studies have found other extended CUT transcripts (eCUTs; specifically CUT060 and CUT095) to be significantly longer in Nrd1 mutants than in *rrp6Δ* as well [48,58]. The mechanisms that underlie the specific requirement for Rrp6 at some transcripts for Nrd1 termination are unknown, but we would speculate that the requirement for Rrp6 in NNS-termination is mediated through its interaction with Nrd1 [7,8,49]. Future studies will also be needed to determine if the requirement for Rrp6 in NNS termination is direct or occurs through an indirect mechanism.

In this study, we have performed multiple biological replicates (n = 4) to provide highly accurate differential expression analysis for all classes of annotated transcripts. In addition, we have included differential expression analysis of 1215 antisense transcripts that were significantly changed in *rrp6Δ*, the majority of which (76%) were upregulated. These data will serve as a valuable resource to determine the diverse roles of the NNS pathway and Rrp6 in transcriptome-wide gene expression regulation. Our dataset also shows that the increased dynamic range and resolution provided by RNA-Seq and ChIP-exo gives a distinct advantage in precise mapping of the transcripts that accumulate following deletion of *RRP6* [10,11,48]. Using RNA-Seq, we have determined that loss of Rrp6 leads to decreased expression of the majority of sn/snoRNA transcripts. Additionally, we have found that the mRNA transcripts from ribosomal protein coding genes show a strong dependence on Rrp6 for control of their steady state transcript levels. Previous ChIP studies on Nrd1 have shown that although Nrd1 localizes to many ribosomal protein-coding genes, it does not directly bind to the mRNA transcripts from those genes [39]. This class of mRNA transcripts may be regulated by Nrd1 and Rrp6 due to their short length and hence high levels of Ser5-phosphorylated RNAPII at their 3’ ends, which could facilitate interaction with Nrd1 and regulation of termination [8,37,74,75]. By comparison of our data to recent 4tU-Seq datasets following Nrd1-depletion from the nucleus, we have found that multiple ribosomal subunit mRNAs display transcript extension following loss of Nrd1 activity and have been annotated as NUTs [48]. These data strongly suggest that ribosomal protein coding mRNAs may require Rrp6 and the NNS pathway for proper 3’ processing and transcript stability.

It has previously been determined that the downstream region of snR13 contains both an Nrd1-dependent terminator and a polyA dependent terminator that is present only 50 nucleotides downstream of the Nrd1 terminator [63]. In *rrp6Δ* cells we observed proper termination for snR13 although termination was defective in other NNS-pathway components (Fig. 2). It is important to note, however, that overall levels of the mature processed snR13 transcript were not significantly changed in any of the mutant backgrounds, which is likely due to the presence of a second polyA-dependent terminator (Fig. 2D). It is likely that the differences in NNS-dependent termination efficiency at specific target genes observed in *rrp6Δ* is reliant on the context of both terminators and other downstream sequence elements that can contribute to proper RNAPII termination downstream. Additional failsafe mechanisms for transcription termination have also been described in yeast that involve the nuclear RNase III enzyme Rnt1 that could contribute to proper snRNA termination in the absence of NNS-termination pathway components including Rrp6 [76,77].

High-resolution RNAPII occupancy maps generated by ChIP-exo provide unique insights into the mechanisms of NNS-dependent termination. At *NRD1*, *HRP1*, and *YPL222C-A*, deletion of *RRP6* lead to a 3’ shift in RNAPII localization indicating that NNS-dependent termination was delayed or less efficient (Figs. 6,7,and S4). This phenomena was also observed to a lesser extent at *SRG1-SER3* (S5 Fig.). Interestingly, we observed distinct accumulation of RNAPII at these regions that occurred just 3’ to Nrd1 and/or Nab3 binding sites that have previously been mapped by RNA-protein crosslinking approaches (Fig. 6) [39,47]. These findings suggest that termination through the NNS-pathway may be an inefficient process requiring clusters of Nrd1/Nab3 binding sites to for facilitate higher order recruitment of multiple Nrd1-Nab3 heterodimers to carry out termination, which has support from other studies [27,59,78]. This hypothesis is further supported by studies that show that NNS-termination occurs in a ‘termination zone’ rather than a specific termination site as proposed for polyA-dependent termination pathways [40]. Through this mechanism, gene expression attenuation at genes such as *NRD1* and *SRG1-SER3* would remain leaky, allowing for transcription of a small percentage of full-length transcripts even in the presence of high Nrd1-Nab3 protein levels, which is what we observe in WT cells at these genes (Figs. 6, 7, S4, and S5). The requirement for the nuclear exosome and Rrp6 for efficient NNS-regulated attenuation adds a layer of complexity to this process and another path for regulation for these tightly controlled genes.

## Methods

### Yeast strains and RNA isolation

All yeast strains used are isogenic to BY4741. *RRP6* deletion strain is from the yeast knockout collection (Open Biosystems) [79]. The Rpb3-FLAG WT strain was produced by yeast transformation with a tagging cassette containing sequences for a 3X-FLAG tag upstream of the URA3 gene from the plasmid pBS1539 (primer sequences available upon request) [80]. *RRP6* deletion Rpb3–3XFLAG strains were produced by amplification of the *RRP6* knockout cassette from the yeast knockout collection *RRP6* deletion strain and transformation into the wild-type Rpb3-FLAG strain. *RRP6* knockout was confirmed by PCR genotyping. RNA was extracted using the hot acid phenol method. Briefly, strains were grown in 100ml YPD medium to an OD_600_ of 0.8. Cells were collected by centrifugation, washed, and resuspended in 10ml AE buffer (50mM sodium acetate at pH 5.2, 10mM EDTA) in a Nalgene phenol-resistant 50ml tube. 800μl 20% SDS and 10ml cold acid phenol were added to each sample and mixed well by vortexing. Samples were incubated at 65°C for 10 minutes with vortexing every minute then cooled on ice for 5 minutes. Samples were centrifuged for 15 minutes at 10,000 rpm. The top phase was transferred to a pre-spun 50ml 5 PRIME Phase Lock Gel tube (Ref # 2302870). 13ml chloroform was added and well mixed before centrifuging for 10 minutes at 3000 rpm. The top phase was poured into a new phenol-resistant tube, and 1/10 volume sodium acetate at pH 5.2 and equal volume room temperature isopropanol was added. The precipitated RNA was collected by centrifuging for 45 minutes at 12,000 rpm. The pellet was washed with 70% ethanol, allowed to dry in a fume hood, and resuspended with molecular biology grade water. The Ambion DNase-turbo kit was used to degrade any contaminating DNA. The quality of the samples was determined with an Agilent Bioanalyzer before preparation of the sequencing libraries.

### ChIP-exo analysis of Rpb3-FLAG localization

Chromatin IP followed by exonuclease treatment was performed using the protocol described by Rhee and Pugh with the following modifications [55]. Rpb3-FLAG WT and *rrp6Δ* strains were grown to an OD_600_ = 0.8–1 prior to crosslinking with formaldehyde (Sigma, catalog # F8775–25ML). Immunoprecipitation was performed with 50uL of anti-FLAG agarose (Sigma). Subsequent sample processing steps including exonuclease treatment and sequencing library preparation were performed as previously described [55].

### Northern blot analysis

30ug of total RNA was loaded per lane on a 1.5% and separated by electrophoresis at 120 volts for 1 hour at 4°C. The RNA was transferred to Bio-Rad Zeta-Probe blotting membranes by capillary overnight. Transfer efficiency was determined by Methylene Blue staining. DNA oligonucleotide probes listed in S5 Table were 5’ end-labeled with gamma ATP-32P by T4 Polynucleotide Kinase. Probes were hybridized overnight to pre-blocked in Roche Life Science DIG Easy Hyb buffer at 37°C. Blots were washed in 6XSSC / 0.1%SDS once at room temperature and twice for 10 minutes at 50°C. Blots were exposed to a phosphorscreen overnight for snRNAs or 7 days for *FMP40* and *YPL22C-A* followed by scanning using a phosphorimager (GE Healthcare).

### SOLiD5500xl sequencing methods

Standard methods were used for RNA-seq library construction, EZBead preparation and Next-Gen sequencing, based on Life Technologies SOLiD5500xl system. Briefly, five microgram of total RNA per sample (RIN equal or higher than 6.0 by Agilent Bioanalyzer) was applied in library preparation. rRNA was first depleted using the standard protocol of RiboMinus Transcriptome Isolation Kit for yeast (Ambion, Cat# K1550–03), and rRNA-depleted RNA was concentrated with the PureLink RNA Micro Kit (Invitrogen, Cat# 12183–016) using 1 volume of Lysis Buffer and 2.5 volumes of 100% ethanol. Following the rRNA depletion, whole transcriptome library was prepared and barcoded per sample using the standard protocol of SOLiD Total RNA-seq Kit (Life Technologies, Cat# 4445374). Each barcoded library was quantified by quantitative PCR using SOLiD Library Taqman qPCR Module (Life Technologies, Cat#A12127), and pooled in equal molarity. Fifty microliters of 500 pM of pooled library was used in subsequent EZBead preparation, which involves bead emulsion, bead library amplification, and bead enrichment using Life Technologies EZ Bead E120 System (Cat# 4465571). Approximately six hundred million enriched beads then were deposited onto each lane of a 6-lane SOLiD5500xl flow chip. And finally sequencing by ligation was carried out using standard single-read, 5’-3’ strand-specific sequencing procedure (75b-read) on SOLiD5500xl Sequencer.

### Sequencing data analysis

The libraries were sequenced on Solid 5500XL. The resulting 75 bp solid reads were mapped to *Saccharomyces cerevisiae* reference genome sacCer3 using in-house mapping pipelines that utilizes bfast-0.7.0a [81]. Briefly, using our RNA-seq pipeline, poor quality and rRNA/tRNAs reads were first discarded. The remaining reads were mapped to reference genome sacCer3 and a splice-junction library, respectively; the genomic and splice-junction library mapping were merged at the end. In a different pipeline, the rRNA/tRNAs were kept and the reads were mapped to the reference genome sacCer3 only based on the facts that in yeast there is some splicing but most genes do not have introns. Read counts were calculated using bamutils from NGSUtils [82]. Differential gene expression was analyzed using edgeR which calculated all normalized read counts, p-values, and FDR values listed in S1 Table [83]. All raw and processed files from the RNA sequencing performed for this study has been deposited to Gene Expression Omnibus [GEO] under the accession number GSE57155.

### Manual annotation of snRNA extension and antisense transcripts

Following data alignment, snRNA transcripts were manually inspected individually using the Integrative Genomics Viewer [84,85]. For all snRNAs in a tail-to-tail orientation with a downstream gene, the snRNA ET annotation started just after the end of the snRNA annotation until continuous reads on the same strand were no longer detected. For all snRNAs in a tail-to-head orientation, the snRNA ET annotation started just after the end of the snRNA annotation and was ended just prior to the 5’ end of the annotation for the downstream gene. Annotations for ET for snRNAs that were encoded within introns were ended just prior to the 5’ end of the exon for the parent transcript. To identify antisense transcripts with significant changes in differential expression, the strand was reversed for all sense annotations for the coding region of each ORF-Ts and the text “AS_” was added in front of the ORF-T name. The annotations for the 5’ and 3’ UTR were not included. These annotations were then used for edgeR analysis and the annotations for antisense transcripts that showed significant changes in *rrp6Δ* were used for subsequent differential expression analysis to generate the final dataset in S1 Table.

### GOStat analysis

GOStat analysis [86] was performed as previously described [87] for all significantly down-regulated ORF-T transcripts from our dataset. In brief, the list of 995 significantly upregulated ORF-Ts was entered into the GOStat web interface (http://gostat.wehi.edu.au/cgi-bin/goStat.pl) to search for the top 30 most over-represented GO terms and to obtain p-values to indicate the significance of enrichment (S3 Table). GO-term enrichment analysis was also performed using DAVID [88] and similar results were obtained.

### Alignment of Nrd1-unterminated transcript data

4tU-seq reads of both wild type and Nrd1 depleted samples were downloaded from ftp.sra.ebi.ac.uk/vol1/ERA242/ERA242535/fastq/. The sequence data were processed according to the description in [48,89,90] with minor changes. Briefly, the reads were quality trimmed and mapped with bowtie-0.12.9 [91] to reference genome sacCer3 (mapping parameters:-q-p 4-S-n 0-e 70-l 28-y-k 1-m 2—best—strata—phred33-quals—norc/—nofw). The output SAM files were then converted to BAM files with SAMtools [92]. The BAM files were further processed to keep the uniquely and perfectly mapped reads using bamutils from NGSUtils [82].

## Supporting Information

S1 Fig Expression plots for normalized RNA-SEQ data for Nrd1-unterminated transcripts (NUTs) and western blot analysis of Rpb3-FLAG strains. (A) RNAs annotated as NUTs, a classification based on the dependence of Nrd1 for appropriate termination are shown as aqua dots while all other transcript annotations are shown as black dots. (B) Western blot analysis of whole cell extracts prepared from Rpb3-FLAG WT and *rrp6Δ* strains using anti-FLAG peroxidase coupled antibodies (Sigma).
(TIF)

S2 Fig Comparison of *rrp6Δ* RNA-Seq reads at snR3 to published 4tU-Seq data for NUTs. (A) Mapped reads obtained from our in-house alignment (see methods section) of 4tU-Seq data from Schulz *et al* 2013 (top two panels) and our RNA-Seq data (bottom two panels) at snR3-*STR2* region, forward strand reads only. The location and transcription direction of all annotations within this region are diagrammed below. Processed length of snRNAs and mRNAs are in black, snRNA-extended regions are in green (labeled “ET”), NUTs are in blue, CUTs are in gray, and arrows indicate annotated transcript state site and direction of transcription. The dotted green line marks the 3’ end of the extended snR3 annotation in *rrp6Δ*. (B) Average normalized read counts ± standard deviations for transcripts in this region that are not significantly changed in *rrp6Δ* versus wild-type (n = 4). The colors of the bars correspond to the color representing the annotation.
(TIF)

S3 Fig Termination of the H/ACA box small nucleolar RNA snR11 shifts 3’ in *rrp6Δ* cells. (A) Diagram showing proposed mechanism where down-regulation of GeneX results from faulty termination of snRNA in *rrp6Δ*. Decreased NNS-termination at select snRNAs results in longer transcribed region, extending over transcription start site of downstream convergent gene, GeneX. The hypothesized resulting increased localization of the transcription machinery interferes with initiation at the TSS of GeneX (indicated with a red ‘X’). (B) Strand-specific northern blot analysis using a 5’ end labeled DNA oligo probe specific to the processed region of snR11 directly comparing *rrp6Δ* to mutants known to be defective in Nrd1-dependent termination. The 26S and 18S ribosomal RNAs are shown as a loading control (bottom). (C) Graphical representation of strand-specific RNA-seq reads mapped to snR11-*ICY1* region. Reads mapped to the positive strand are on top in red, while reads mapped to the negative strand are on the bottom in blue. The location and direction of transcription for all analyzed annotations are diagrammed below the graphs to scale. Processed length of snRNAs and mRNAs are in black, snRNA-extended transcripts, including pre-snRNAs and termination read-through products, are in green (labeled “ETs”), NUTs are in aqua, CUTs are in gray, SUTs are in dark blue, SRTs are in purple, and bent arrows indicate direction of the TSS. The dotted black lines mark the transcription start sites (TSS) of *CMC4*. (D) Rpb3-FLAG localization as determined by ChIP-exo sequencing reads mapped to the same region and aligned to (C). Wild-type normalized read counts are in black, and *rrp6Δ* are in orange. Arrows indicate areas of interest.
(TIF)

S4 Fig Efficient termination of RNAPII at the *HRP1* 5’UTR requires Rrp6. (A) Rpb3-FLAG localization as determined by ChIP-exo sequencing reads mapped to the *HRP1* region. Wild-type reads are in black, and *rrp6Δ* are in orange. The location and direction of transcription for all analyzed annotations are diagrammed below the graphs to scale. Length of mRNAs including untranslated regions are in black. (B) Graphical representation of strand-specific RNA-seq reads mapped to *HRP1* transcribed region. Reads mapped to the positive strand are shown in red. No reads were mapped to the reverse strand at *HRP1*.
(TIF)

S5 Fig Rrp6 regulates RNAPII localization at *SRG1-SER3* independent of other NNS-pathway disruptions. (A) Rpb3-FLAG localization as determined by ChIP-exo sequencing reads mapped to the *SRG1-SER3* region. Wild-type normalized read counts are in black, and *rrp6Δ* are in orange. The location and direction of transcription for all analyzed annotations are diagrammed below the graphs to scale. Length of mRNAs including untranslated regions are in black, CUTs are in gray, and SRTs (Ssu72 regulated transcripts) are in purple. Note that *SRG1* and *SER3* annotations are overlapping. (B) Graphical representation of strand-specific RNA-seq reads mapped to *SRG1-SER3* transcribed region. Reads mapped to the positive strand are shown in red while reverse reads are shown in blue.
(TIF)

S1 Table Complete differential expression dataset for the *RRP6* deletion RNA-Seq. Table includes differential expression data expressed in log_2_ *rrp6Δ*/WT ratio (i.e. fold change), as well as p-values and false discovery rate (FDR), all calculated from four replicates by the EdgeR program as discussed in the methods. Class abbreviations are ORF-T: open reading frame transcript, AST: antisense transcript, NUT: Nrd1-unterminated transcript, SRT: Ssu72-restricted transcript, SUT: stable unannotated transcript, CUT: cryptic unstable transcript, sn/snoRNA-ET: Extended region of an sn/snoRNA. Transcript name listed is the systematic name where possible. “AS_” preceding the name designates antisense transcripts, NUTs, CUTs, SUTs, and SRTs are listed as the number provided by their original publications [11, 46, 59]. “N.reads” columns are normalized read counts, calculated by EdgeR.
(PDF)

S2 Table Top 30 GO-terms enriched in the down-regulated protein coding gene dataset. Table includes GO identification number (“Best GOs”), number of hits from GOStat analysis of our down-regulated protein coding gene list matching the GO ID (“Count”), total number of genes assigned the GO ID (“Total”), p-value for the GO term, and the descriptor for the GO ID. Information calculated using GOstat, as described in the methods.
(PDF)

S3 Table Differential expression information for the ribosomal protein coding transcripts. Table includes differential expression data expressed in log_2_ *rrp6Δ*/WT ratio (i.e. fold change) as well as p-values and false discovery rate (FDR), all calculated from four replicates by the EdgeR program as discussed in the methods. Table also includes standard gene names (or acronyms) for clarity. Significantly downregulated transcripts are in red-shaded cells whereas upregulated sn/snoRNAs are in green-shaded cells.
(PDF)

S4 Table Differential expression information for the sn/snoRNAs. Table includes differential expression data expressed in log_2_ *rrp6Δ*/WT ratio (i.e. fold change), as well as p-values and false discovery rate (FDR), all calculated from four replicates by the EdgeR program as discussed in the methods. Significantly downregulated sn/snoRNAs are in red-shaded cells whereas upregulated sn/snoRNAs are in green-shaded cells.
(PDF)

S5 Table Sequences for DNA oligonucleotide probes used for northern blotting. Oligonucleotide sequences for each strand specific probe used for end labeling and northern blot analysis.
(PDF)

## References

[1] Bonneau F, Basquin J, Ebert J, Lorentzen E, Conti E (2009) The yeast exosome functions as a macromolecular cage to channel RNA substrates for degradation. Cell 139: 547–559. 10.1016/j.cell.2009.08.042 19879841

[2] Schneider C, Tollervey D (2013) Threading the barrel of the RNA exosome. Trends in biochemical sciences 38: 485–493. 10.1016/j.tibs.2013.06.013 23910895 PMC3838930

[3] Makino DL, Baumgartner M, Conti E (2013) Crystal structure of an RNA-bound 11-subunit eukaryotic exosome complex. Nature 495: 70–75. 10.1038/nature11870 23376952

[4] Makino DL, Halbach F, Conti E (2013) The RNA exosome and proteasome: common principles of degradation control. Nature reviews Molecular cell biology 14: 654–660. 10.1038/nrm3657 23989960

[5] Mitchell P, Petfalski E., Shevchenko A., Mann M., Tollervey D. (1997) The exosome: a conserved eukaryotic RNA processing complex containing multiple 3′—>5′ exoribonucleases. Cell 91: 457–466. 9390555 10.1016/s0092-8674(00)80432-8

[6] Lorentzen E, Basquin J, Tomecki R, Dziembowski A, Conti E (2008) Structure of the active subunit of the yeast exosome core, Rrp44: diverse modes of substrate recruitment in the RNase II nuclease family. Molecular cell 29: 717–728. 10.1016/j.molcel.2008.02.018 18374646

[7] Vasiljeva L, Buratowski S (2006) Nrd1 interacts with the nuclear exosome for 3′ processing of RNA polymerase II transcripts. Molecular cell 21: 239–248. 16427013 10.1016/j.molcel.2005.11.028

[8] Heo DH, Yoo I, Kong J, Lidschreiber M, Mayer A, et al (2013) The RNA Polymerase II C-terminal Domain-Interacting Domain of Yeast Nrd1 Contributes to the Choice of Termination Pathway and Couples to RNA Processing by the Nuclear Exosome. The Journal of biological chemistry. 288: 36676–36690. 10.1074/jbc.M113.508267 24196955 PMC3868778

[9] Wyers F, Rougemaille M, Badis G, Rousselle JC, Dufour ME, et al (2005) Cryptic pol II transcripts are degraded by a nuclear quality control pathway involving a new poly(A) polymerase. Cell 121: 725–737. 15935759 10.1016/j.cell.2005.04.030

[10] Neil H, Malabat C, d’Aubenton-Carafa Y, Xu Z, Steinmetz LM, et al (2009) Widespread bidirectional promoters are the major source of cryptic transcripts in yeast. Nature 457: 1038–1042. 10.1038/nature07747 19169244

[11] Xu Z, Wei W, Gagneur J, Perocchi F, Clauder-Munster S, et al (2009) Bidirectional promoters generate pervasive transcription in yeast. Nature 457: 1033–1037. 10.1038/nature07728 19169243 PMC2766638

[12] Porrua O, Hobor F, Boulay J, Kubicek K, D’Aubenton-Carafa Y, et al (2012) In vivo SELEX reveals novel sequence and structural determinants of Nrd1-Nab3-Sen1-dependent transcription termination. The EMBO Journal 31: 3935–3948. 10.1038/emboj.2012.237 23032188 PMC3463846

[13] Davis CA, Ares M Jr., (2006) Accumulation of unstable promoter-associated transcripts upon loss of the nuclear exosome subunit Rrp6p in Saccharomyces cerevisiae. Proceedings of the National Academy of Sciences of the United States of America 103: 3262–3267. 16484372 10.1073/pnas.0507783103 PMC1413877

[14] Dichtl B BD, Ohnacker M, Friedlein A, Roeder D, Langen H, Keller W. (2002) A Role for SSU72 in Balancing RNA Polymerase II Transcription Elongation and Termination. Molecular Cell 10: 1139–1150. 12453421 10.1016/s1097-2765(02)00707-4

[15] Schmid M, Poulsen MB, Olszewski P, Pelechano V, Saguez C, et al (2012) Rrp6p controls mRNA poly(A) tail length and its decoration with poly(A) binding proteins. Molecular cell 47: 267–280. 10.1016/j.molcel.2012.05.005 22683267 PMC3408791

[16] Grenier St-Sauveur V, Soucek S, Corbett AH, Bachand F (2013) Poly(A) tail-mediated gene regulation by opposing roles of Nab2 and Pab2 nuclear poly(A)-binding proteins in pre-mRNA decay. Molecular and cellular biology 33: 4718–4731. 10.1128/MCB.00887-13 24081329 PMC3838004

[17] Gudipati RK, Xu Z, Lebreton A, Seraphin B, Steinmetz LM, et al (2012) Extensive degradation of RNA precursors by the exosome in wild-type cells. Molecular cell 48: 409–421. 10.1016/j.molcel.2012.08.018 23000176 PMC3496076

[18] Schneider C, Kudla G, Wlotzka W, Tuck A, Tollervey D (2012) Transcriptome-wide analysis of exosome targets. Molecular cell 48: 422–433. 10.1016/j.molcel.2012.08.013 23000172 PMC3526797

[19] Steinmetz EJC Nicholas K.; Brow David A.; Corden Jeffry L. (2001) RNA-binding protein Nrd1 directs poly(A)-independent 3′-end formation of RNA polymerase II transcripts. Nature 413: 327–331. 11565036 10.1038/35095090

[20] Kim M, Vasiljeva L, Rando OJ, Zhelkovsky A, Moore C, et al (2006) Distinct pathways for snoRNA and mRNA termination. Molecular cell 24: 723–734. 17157255 10.1016/j.molcel.2006.11.011

[21] Buratowski S (2009) Progression through the RNA polymerase II CTD cycle. Molecular cell 36: 541–546. 10.1016/j.molcel.2009.10.019 19941815 PMC3232742

[22] Kim MK Nevan J.; Vasiljeva Lidia; Rando Oliver J.; Nedea Eduard; Greenblatt Jack F.; Buratowski Stephen (2004) The yeast Rat1 exonuclease promotes transcription termination by RNA polymerase II. Nature 432: 517–522. 15565157 10.1038/nature03041

[23] Nedea E, He X, Kim M, Pootoolal J, Zhong G, et al (2003) Organization and function of APT, a subcomplex of the yeast cleavage and polyadenylation factor involved in the formation of mRNA and small nucleolar RNA 3′-ends. The Journal of biological chemistry 278: 33000–33010. 12819204 10.1074/jbc.M304454200

[24] Zhao JK Marco; Helmling Steffen; O’Connor J. Patrick; Moore Claire (1999) Pta1, a component of yeast CF II, is required for both cleavage and poly(A) addition of mRNA precursor. Molecular and cellular biology 19: 7733–7740. 10523662 10.1128/mcb.19.11.7733 PMC84822

[25] Kyberz AS Martin; Dichtl Bernhard; Keller Walter (2003) The role of the yeast cleavage and polyadenylation factor subunit Ydh1p/Cft2p in pre-mRNA 3′-end formation. Nucleic acids research 31: 3936–3945. 12853609 10.1093/nar/gkg478 PMC167639

[26] Lunde BM, Reichow SL, Kim M, Suh H, Leeper TC, et al (2010) Cooperative interaction of transcription termination factors with the RNA polymerase II C-terminal domain. Nat Struct Mol Biol 17: 1195–1201. 10.1038/nsmb.1893 20818393 PMC2950884

[27] Carroll KL, Ghirlando R, Ames JM, Corden JL (2007) Interaction of yeast RNA-binding proteins Nrd1 and Nab3 with RNA polymerase II terminator elements. RNA 13: 361–373. 17237360 10.1261/rna.338407 PMC1800511

[28] Arigo JT, Eyler DE, Carroll KL, Corden JL (2006) Termination of cryptic unstable transcripts is directed by yeast RNA-binding proteins Nrd1 and Nab3. Mol Cell 23: 841–851. 16973436 10.1016/j.molcel.2006.07.024

[29] Carroll KL, Pradhan DA, Granek JA, Clarke ND, Corden JL (2004) Identification of cis elements directing termination of yeast nonpolyadenylated snoRNA transcripts. Molecular and cellular biology 24: 6241–6252. 15226427 10.1128/MCB.24.14.6241-6252.2004 PMC434237

[30] Steinmetz EJ, Conrad NK, Brow DA, Corden JL (2001) RNA-binding protein Nrd1 directs poly(A)-independent 3′-end formation of RNA polymerase II transcripts. Nature 413: 327–331. 11565036 10.1038/35095090

[31] Thiebaut M, Kisseleva-Romanova E, Rougemaille M, Boulay J, Libri D (2006) Transcription termination and nuclear degradation of cryptic unstable transcripts: a role for the nrd1-nab3 pathway in genome surveillance. Molecular cell 23: 853–864. 16973437 10.1016/j.molcel.2006.07.029

[32] Honorine R, Mosrin-Huaman C, Hervouet-Coste N, Libri D, Rahmouni AR (2011) Nuclear mRNA quality control in yeast is mediated by Nrd1 co-transcriptional recruitment, as revealed by the targeting of Rho-induced aberrant transcripts. Nucleic acids research 39: 2809–2820. 10.1093/nar/gkq1192 21113025 PMC3074134

[33] Meinhart A CP (2004) Recognition of RNA polymerase II carboxy-terminal domain by 3′-RNA-processing factors. Nature 430: 223–226. 15241417 10.1038/nature02679

[34] Vasiljeva L, Kim M, Mutschler H, Buratowski S, Meinhart A (2008) The Nrd1-Nab3-Sen1 termination complex interacts with the Ser5-phosphorylated RNA polymerase II C-terminal domain. Nature structural & molecular biology 15: 795–804. 10.1111/nph.13282 18660819 PMC2597375

[35] Vasiljeva L, Kim M, Terzi N, Soares LM, Buratowski S (2008) Transcription termination and RNA degradation contribute to silencing of RNA polymerase II transcription within heterochromatin. Mol Cell 29: 313–323. 10.1016/j.molcel.2008.01.011 18280237

[36] Conrad NK, Wilson SM, Steinmetz EJ, Patturajan M, Brow DA, et al (2000) A yeast heterogeneous nuclear ribonucleoprotein complex associated with RNA polymerase II. Genetics 154: 557–571. 10655211 10.1093/genetics/154.2.557 PMC1460961

[37] Gudipati RK, Villa T, Boulay J, Libri D (2008) Phosphorylation of the RNA polymerase II C-terminal domain dictates transcription termination choice. Nat Struct Mol Biol 15: 786–794. 10.1038/nsmb.1460 18660821

[38] Jamonnak N, Creamer TJ, Darby MM, Schaughency P, Wheelan SJ, et al (2011) Yeast Nrd1, Nab3, and Sen1 transcriptome-wide binding maps suggest multiple roles in post-transcriptional RNA processing. RNA 17: 2011–2025. 10.1261/rna.2840711 21954178 PMC3198594

[39] Creamer TJ, Darby MM, Jamonnak N, Schaughency P, Hao H, et al (2011) Transcriptome-wide binding sites for components of the Saccharomyces cerevisiae non-poly(A) termination pathway: Nrd1, Nab3, and Sen1. PLoS Genet 7: e1002329 10.1371/journal.pgen.1002329 22028667 PMC3197677

[40] Hazelbaker DZ, Marquardt S, Wlotzka W, Buratowski S (2013) Kinetic Competition between RNA Polymerase II and Sen1-Dependent Transcription Termination. Molecular cell 49: 55–66. 10.1016/j.molcel.2012.10.014 23177741 PMC3545030

[41] Brow DA (2011) Sen-sing RNA terminators. Mol Cell 42: 717–718. 10.1016/j.molcel.2011.06.002 21700218 PMC3136216

[42] Steinmetz EJ, Warren CL, Kuehner JN, Panbehi B, Ansari AZ, et al (2006) Genome-wide distribution of yeast RNA polymerase II and its control by Sen1 helicase. Mol Cell 24: 735–746. 17157256 10.1016/j.molcel.2006.10.023

[43] Skourti-Stathaki K, Proudfoot NJ, Gromak N (2011) Human senataxin resolves RNA/DNA hybrids formed at transcriptional pause sites to promote Xrn2-dependent termination. Mol Cell 42: 794–805. 10.1016/j.molcel.2011.04.026 21700224 PMC3145960

[44] Lykke-Andersen S, Jensen TH (2007) Overlapping pathways dictate termination of RNA polymerase II transcription. Biochimie 89: 1177–1182. 17629387 10.1016/j.biochi.2007.05.007

[45] Grzechnik P, Kufel J (2008) Polyadenylation linked to transcription termination directs the processing of snoRNA precursors in yeast. Mol Cell 32: 247–258. 10.1016/j.molcel.2008.10.003 18951092 PMC2593888

[46] Stuparevic I, Mosrin-Huaman C, Hervouet-Coste N, Remenaric M, Rahmouni AR (2013) Cotranscriptional recruitment of RNA exosome cofactors Rrp47p and Mpp6p and two distinct Trf-Air-Mtr4 polyadenylation (TRAMP) complexes assists the exonuclease Rrp6p in the targeting and degradation of an aberrant messenger ribonucleoprotein particle (mRNP) in yeast. J Biol Chem 288: 31816–31829. 10.1074/jbc.M113.491290 24047896 PMC3814775

[47] Wlotzka W, Kudla G, Granneman S, Tollervey D (2011) The nuclear RNA polymerase II surveillance system targets polymerase III transcripts. EMBO J 30: 1790–1803. 10.1038/emboj.2011.97 21460797 PMC3102002

[48] Schulz D, Schwalb B, Kiesel A, Baejen C, Torkler P, et al (2013) Transcriptome surveillance by selective termination of noncoding RNA synthesis. Cell 155: 1075–1087. 10.1016/j.cell.2013.10.024 24210918

[49] Tudek A, Porrua O, Kabzinski T, Lidschreiber M, Kubicek K, et al (2014) Molecular basis for coordinating transcription termination with noncoding RNA degradation. Mol Cell 55: 467–481. 10.1016/j.molcel.2014.05.031 25066235 PMC4186968

[50] Castelnuovo M, Rahman S, Guffanti E, Infantino V, Stutz F, et al (2013) Bimodal expression of PHO84 is modulated by early termination of antisense transcription. Nat Struct Mol Biol 20: 851–858. 10.1038/nsmb.2598 23770821 PMC4972572

[51] Park D, Morris AR, Battenhouse A, Iyer VR (2014) Simultaneous mapping of transcript ends at single-nucleotide resolution and identification of widespread promoter-associated non-coding RNA governed by TATA elements. Nucleic Acids Res 42: 3736–3749. 10.1093/nar/gkt1366 24413663 PMC3973313

[52] Tan-Wong SM, Zaugg JB, Camblong J, Xu Z, Zhang DW, et al (2012) Gene loops enhance transcriptional directionality. Science 338: 671–675. 10.1126/science.1224350 23019609 PMC3563069

[53] Castelnuovo M, Zaugg JB, Guffanti E, Maffioletti A, Camblong J, et al (2014) Role of histone modifications and early termination in pervasive transcription and antisense-mediated gene silencing in yeast. Nucleic Acids Res 42: 4348–4362. 10.1093/nar/gku100 24497191 PMC3985671

[54] Nagalakshmi U, Wang Z, Waern K, Shou C, Raha D, et al (2008) The transcriptional landscape of the yeast genome defined by RNA sequencing. Science 320: 1344–1349. 10.1126/science.1158441 18451266 PMC2951732

[55] Rhee HS, Pugh BF (2012) ChIP-exo method for identifying genomic location of DNA-binding proteins with near-single-nucleotide accuracy. Curr Protoc Mol Biol Chapter 21: Unit 21 24. 10.1002/0471142727.mb2124s100 PMC3813302 23026909

[56] Ganem C, Devaux F, Torchet C, Jacq C, Quevillon-Cheruel S, et al (2003) Ssu72 is a phosphatase essential for transcription termination of snoRNAs and specific mRNAs in yeast. EMBO J 22: 1588–1598. 12660165 10.1093/emboj/cdg141 PMC152886

[57] Steinmetz EJ, Brow DA (2003) Ssu72 Protein Mediates Both Poly(A)-Coupled and Poly(A)-Independent Termination of RNA Polymerase II Transcription. Molecular and cellular biology 23: 6339–6349. 12944462 10.1128/MCB.23.18.6339-6349.2003 PMC193702

[58] Marquardt S, Hazelbaker DZ, Buratowski S (2011) Distinct RNA degradation pathways and 3′ extensions of yeast non-coding RNA species. Transcription 2: 145–154. 21826286 10.4161/trns.2.3.16298 PMC3149692

[59] Loya TJ, O’Rourke TW, Degtyareva N, Reines D (2013) A network of interdependent molecular interactions describes a higher order Nrd1-Nab3 complex involved in yeast transcription termination. J Biol Chem 288: 34158–34167. 10.1074/jbc.M113.516765 24100036 PMC3837157

[60] Loya TJ, O’Rourke TW, Reines D (2012) A genetic screen for terminator function in yeast identifies a role for a new functional domain in termination factor Nab3. Nucleic Acids Res 40: 7476–7491. 10.1093/nar/gks377 22564898 PMC3424548

[61] Lowe TM, Eddy SR (1999) A computational screen for methylation guide snoRNAs in yeast. Science 283: 1168–1171. 10024243 10.1126/science.283.5405.1168

[62] Samarsky DA, Fournier MJ (1999) A comprehensive database for the small nucleolar RNAs from Saccharomyces cerevisiae. Nucleic Acids Res 27: 161–164. 9847166 10.1093/nar/27.1.161 PMC148121

[63] Steinmetz EJ, Ng SB, Cloute JP, Brow DA (2006) cis- and trans-Acting determinants of transcription termination by yeast RNA polymerase II. Molecular and cellular biology 26: 2688–2696. 16537912 10.1128/MCB.26.7.2688-2696.2006 PMC1430333

[64] Arigo JT, Carroll KL, Ames JM, Corden JL (2006) Regulation of yeast NRD1 expression by premature transcription termination. Mol Cell 21: 641–651. 16507362 10.1016/j.molcel.2006.02.005

[65] Martens JA, Laprade L, Winston F (2004) Intergenic transcription is required to repress the Saccharomyces cerevisiae SER3 gene. Nature 429: 571–574. 15175754 10.1038/nature02538

[66] Pelechano V, Steinmetz LM (2013) Gene regulation by antisense transcription. Nat Rev Genet 14: 880–893. 10.1038/nrg3594 24217315

[67] Camblong J, Beyrouthy N, Guffanti E, Schlaepfer G, Steinmetz LM, et al (2009) Trans-acting antisense RNAs mediate transcriptional gene cosuppression in S. cerevisiae. Genes Dev 23: 1534–1545. 10.1101/gad.522509 19571181 PMC2704465

[68] Houseley J, Rubbi L, Grunstein M, Tollervey D, Vogelauer M (2008) A ncRNA modulates histone modification and mRNA induction in the yeast GAL gene cluster. Mol Cell 32: 685–695. 10.1016/j.molcel.2008.09.027 19061643 PMC7610895

[69] Uhler JP, Hertel C, Svejstrup JQ (2007) A role for noncoding transcription in activation of the yeast PHO5 gene. Proceedings of the National Academy of Sciences of the United States of America 104: 8011–8016. 17470801 10.1073/pnas.0702431104 PMC1859995

[70] Cloutier SC, Wang S, Ma WK, Petell CJ, Tran EJ (2013) Long noncoding RNAs promote transcriptional poising of inducible genes. PLoS Biol 11: e1001715 10.1371/journal.pbio.1001715 24260025 PMC3833879

[71] Terzi N, Churchman LS, Vasiljeva L, Weissman J, Buratowski S (2011) H3K4 trimethylation by Set1 promotes efficient termination by the Nrd1-Nab3-Sen1 pathway. Mol Cell Biol 31: 3569–3583. 10.1128/MCB.05590-11 21709022 PMC3165552

[72] Yassour M, Pfiffner J, Levin JZ, Adiconis X, Gnirke A, et al (2010) Strand-specific RNA sequencing reveals extensive regulated long antisense transcripts that are conserved across yeast species. Genome Biol 11: R87 10.1186/gb-2010-11-8-r87 20796282 PMC2945789

[73] Lenstra TL, Tudek A, Clauder S, Xu Z, Pachis ST, et al (2013) The role of Ctk1 kinase in termination of small non-coding RNAs. PLoS One 8: e80495 10.1371/journal.pone.0080495 24324601 PMC3851182

[74] Mayer A, Lidschreiber M, Siebert M, Leike K, Soding J, et al (2010) Uniform transitions of the general RNA polymerase II transcription complex. Nat Struct Mol Biol 17: 1272–1278. 10.1038/nsmb.1903 20818391

[75] Akhtar MS, Heidemann M, Tietjen JR, Zhang DW, Chapman RD, et al (2009) TFIIH kinase places bivalent marks on the carboxy-terminal domain of RNA polymerase II. Mol Cell 34: 387–393. 10.1016/j.molcel.2009.04.016 19450536 PMC2757088

[76] Rondon AG, Mischo HE, Kawauchi J, Proudfoot NJ (2009) Fail-safe transcriptional termination for protein-coding genes in S. cerevisiae. Molecular cell 36: 88–98. 10.1016/j.molcel.2009.07.028 19818712 PMC2779338

[77] Ghazal G, Gagnon J, Jacques P-E, Landry J-R, Robert F, et al (2009) Yeast RNase III Triggers Polyadenylation-dependent Transcription Termination. Molecular Cell 36: 99–109. 10.1016/j.molcel.2009.07.029 19818713

[78] Loya TJ, O’Rourke TW, Reines D (2013) Yeast Nab3 protein contains a self-assembly domain found in human heterogeneous nuclear ribonucleoprotein-C (hnRNP-C) that is necessary for transcription termination. J Biol Chem 288: 2111–2117. 10.1074/jbc.M112.430678 23192344 PMC3554884

[79] Winzeler EA, Shoemaker DD, Astromoff A, Liang H, Anderson K, et al (1999) Functional characterization of the S. cerevisiae genome by gene deletion and parallel analysis. Science 285: 901–906. 10436161 10.1126/science.285.5429.901

[80] Puig O, Caspary F, Rigaut G, Rutz B, Bouveret E, et al (2001) The tandem affinity purification (TAP) method: a general procedure of protein complex purification. Methods 24: 218–229. 11403571 10.1006/meth.2001.1183

[81] Homer NM Barry; Nelson Stanley F. (2009) BFAST: An Alignment Tool for Large Scale Genome Resequencing. PLOS ONE 4: e7767 10.1371/journal.pone.0007767 19907642 PMC2770639

[82] Breese MR, Liu Y (2013) NGSUtils: a software suite for analyzing and manipulating next-generation sequencing datasets. Bioinformatics 29: 494–496. 10.1093/bioinformatics/bts731 23314324 PMC3570212

[83] Robinson MD, McCarthy DJ, Smyth GK (2010) edgeR: a Bioconductor package for differential expression analysis of digital gene expression data. Bioinformatics 26: 139–140. 10.1093/bioinformatics/btp616 19910308 PMC2796818

[84] Robinson JT, Thorvaldsdottir H, Winckler W, Guttman M, Lander ES, et al (2011) Integrative genomics viewer. Nat Biotechnol 29: 24–26. 10.1038/nbt.1754 21221095 PMC3346182

[85] Thorvaldsdottir H, Robinson JT, Mesirov JP (2013) Integrative Genomics Viewer (IGV): high-performance genomics data visualization and exploration. Brief Bioinform 14: 178–192. 10.1093/bib/bbs017 22517427 PMC3603213

[86] Beissbarth T, Speed TP (2004) GOstat: find statistically overrepresented Gene Ontologies within a group of genes. Bioinformatics 20: 1464–1465. 14962934 10.1093/bioinformatics/bth088

[87] Mosley AL, Sardiu ME, Pattenden SG, Workman JL, Florens L, et al (2011) Highly reproducible label free quantitative proteomic analysis of RNA polymerase complexes. Mol Cell Proteomics 10: M110 000687 10.1074/mcp.M110.000687 21048197 PMC3033667

[88] Huang da W, Sherman BT, Lempicki RA (2009) Systematic and integrative analysis of large gene lists using DAVID bioinformatics resources. Nat Protoc 4: 44–57. 10.1038/nprot.2008.211 19131956

[89] Schwalb B, Schulz D, Sun M, Zacher B, Dumcke S, et al (2012) Measurement of genome-wide RNA synthesis and decay rates with Dynamic Transcriptome Analysis (DTA). Bioinformatics 28: 884–885. 10.1093/bioinformatics/bts052 22285829

[90] Sun M, Schwalb B, Schulz D, Pirkl N, Etzold S, et al (2012) Comparative dynamic transcriptome analysis (cDTA) reveals mutual feedback between mRNA synthesis and degradation. Genome Res 22: 1350–1359. 10.1101/gr.130161.111 22466169 PMC3396375

[91] Langmead B, Trapnell C, Pop M, Salzberg SL (2009) Ultrafast and memory-efficient alignment of short DNA sequences to the human genome. Genome Biol 10: R25 10.1186/gb-2009-10-3-r25 19261174 PMC2690996

[92] Li H, Handsaker B, Wysoker A, Fennell T, Ruan J, et al (2009) The Sequence Alignment/Map format and SAMtools. Bioinformatics 25: 2078–2079. 10.1093/bioinformatics/btp352 19505943 PMC2723002

