# Supplementary figures and images for: The Exosome Component Rrp6 Is Required for RNA Polymerase II Termination at Specific Targets of the Nrd1-Nab3 Pathway
Source: PLoS Genet. 2015 Feb 13;11(2):e1004999. doi: 10.1371/journal.pgen.1004999 (PMC4378619; doi:10.1371/journal.pgen.1004999)

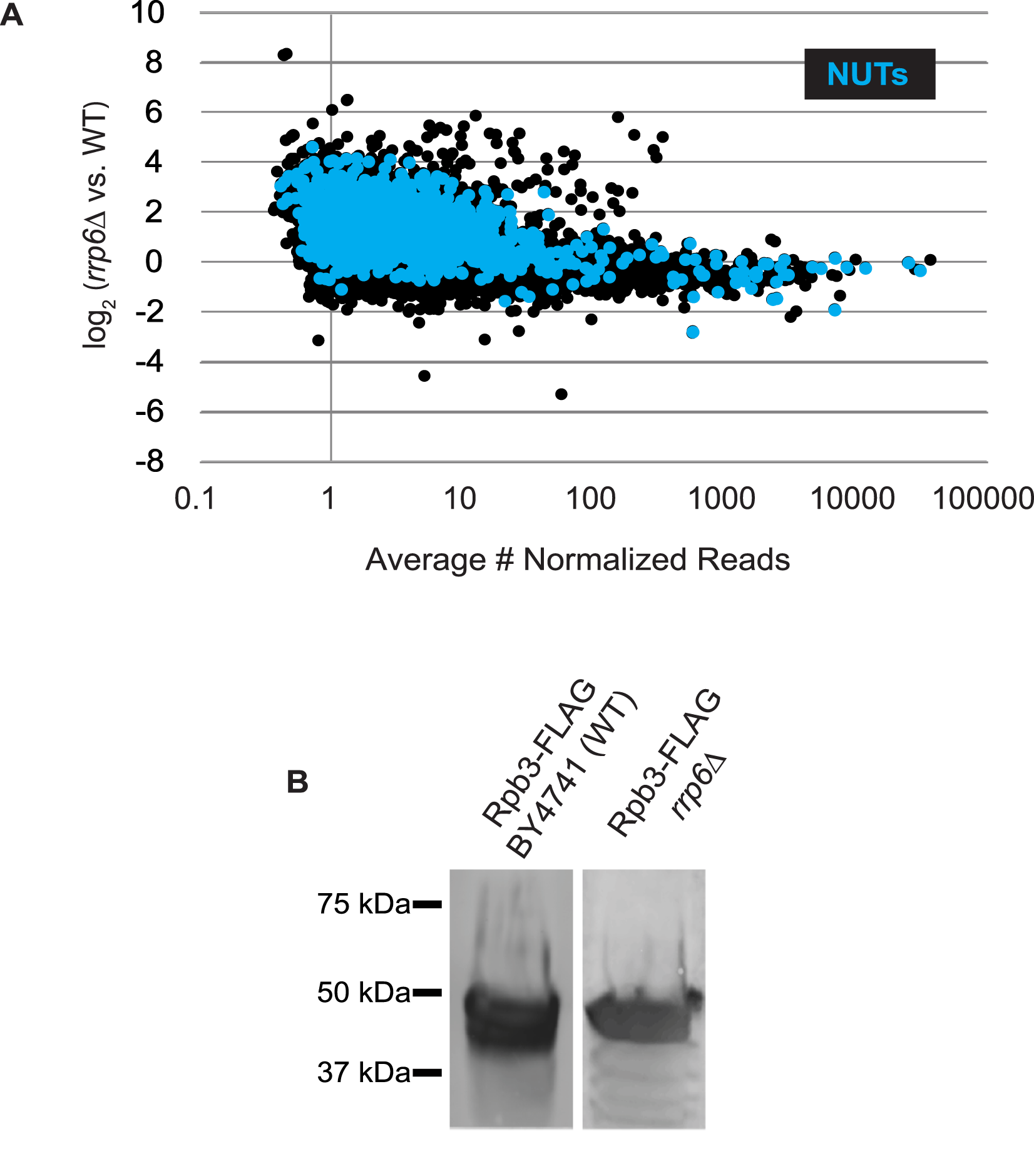

Supplement: S1 Fig — (A) RNAs annotated as NUTs, a classification based on the dependence of Nrd1 for appropriate termination are shown as aqua dots while all other transcript annotations are shown as black dots. (B) Western blot analysis of whole cell extracts prepared from Rpb3-FLAG WT and rrp6Δ strains using anti-FLAG peroxidase coupled antibodies (Sigma). (TIF) [file pgen.1004999.s001.tif]

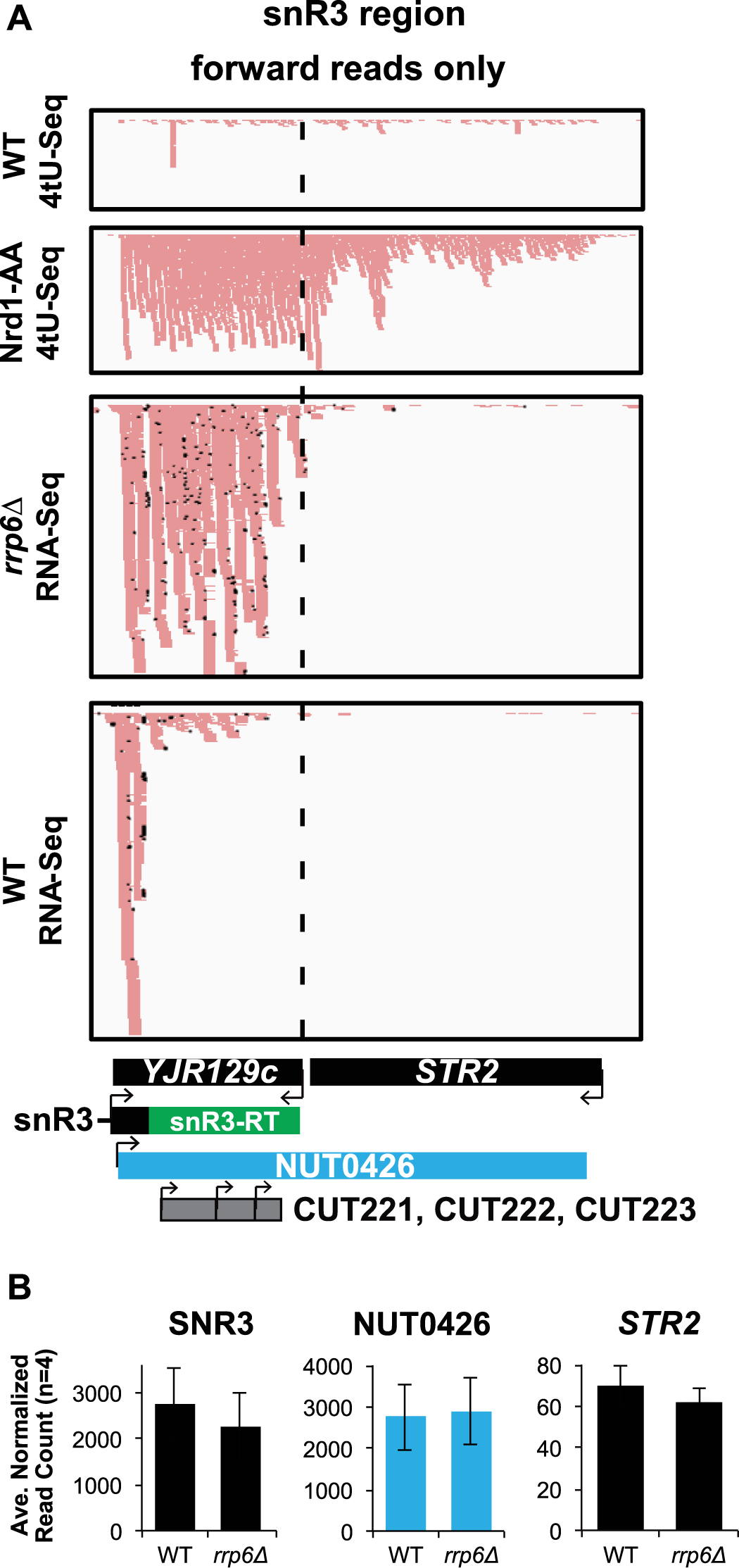

Supplement: S2 Fig — (A) Mapped reads obtained from our in-house alignment (see methods section) of 4tU-Seq data from Schulz et al 2013 (top two panels) and our RNA-Seq data (bottom two panels) at snR3-STR2 region, forward strand reads only. The location and transcription direction of all annotations within this region are diagrammed below. Processed length of snRNAs and mRNAs are in black, snRNA-extended regions are in green (labeled “ET”), NUTs are in blue, CUTs are in gray, and arrows indicate annotated transcript state site and direction of transcription. The dotted green line marks the 3’ end of the extended snR3 annotation in rrp6Δ. (B) Average normalized read counts ± standard deviations for transcripts in this region that are not significantly changed in rrp6Δ versus wild-type (n = 4). The colors of the bars correspond to the color representing the annotation. (TIF) [file pgen.1004999.s002.tif]

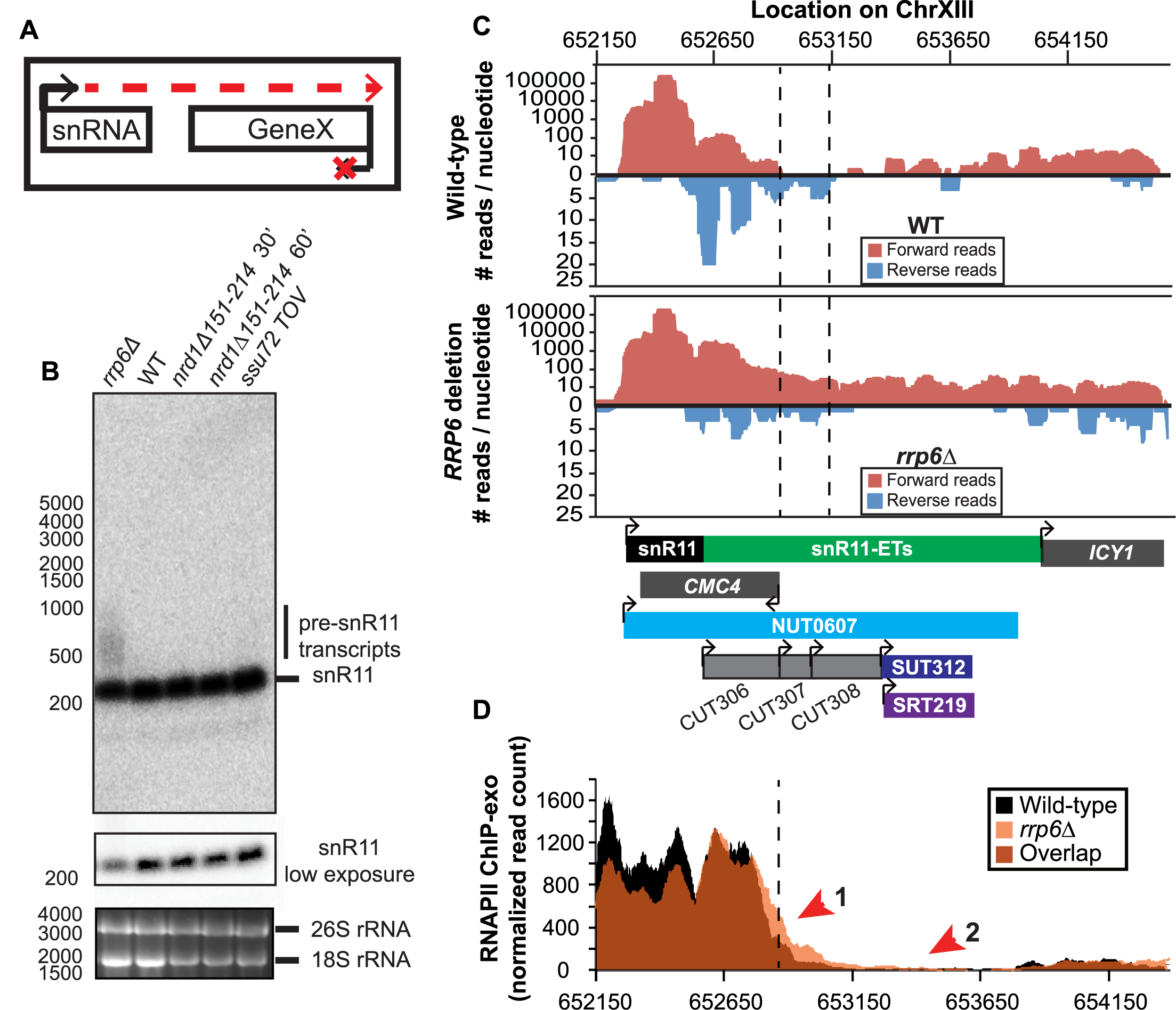

Supplement: S3 Fig — (A) Diagram showing proposed mechanism where down-regulation of GeneX results from faulty termination of snRNA in rrp6Δ. Decreased NNS-termination at select snRNAs results in longer transcribed region, extending over transcription start site of downstream convergent gene, GeneX. The hypothesized resulting increased localization of the transcription machinery interferes with initiation at the TSS of GeneX (indicated with a red ‘X’). (B) Strand-specific northern blot analysis using a 5’ end labeled DNA oligo probe specific to the processed region of snR11 directly comparing rrp6Δ to mutants known to be defective in Nrd1-dependent termination. The 26S and 18S ribosomal RNAs are shown as a loading control (bottom). (C) Graphical representation of strand-specific RNA-seq reads mapped to snR11-ICY1 region. Reads mapped to the positive strand are on top in red, while reads mapped to the negative strand are on the bottom in blue. The location and direction of transcription for all analyzed annotations are diagrammed below the graphs to scale. Processed length of snRNAs and mRNAs are in black, snRNA-extended transcripts, including pre-snRNAs and termination read-through products, are in green (labeled “ETs”), NUTs are in aqua, CUTs are in gray, SUTs are in dark blue, SRTs are in purple, and bent arrows indicate direction of the TSS. The dotted black lines mark the transcription start sites (TSS) of CMC4. (D) Rpb3-FLAG localization as determined by ChIP-exo sequencing reads mapped to the same region and aligned to (C). Wild-type normalized read counts are in black, and rrp6Δ are in orange. Arrows indicate areas of interest. (TIF) [file pgen.1004999.s003.tif]

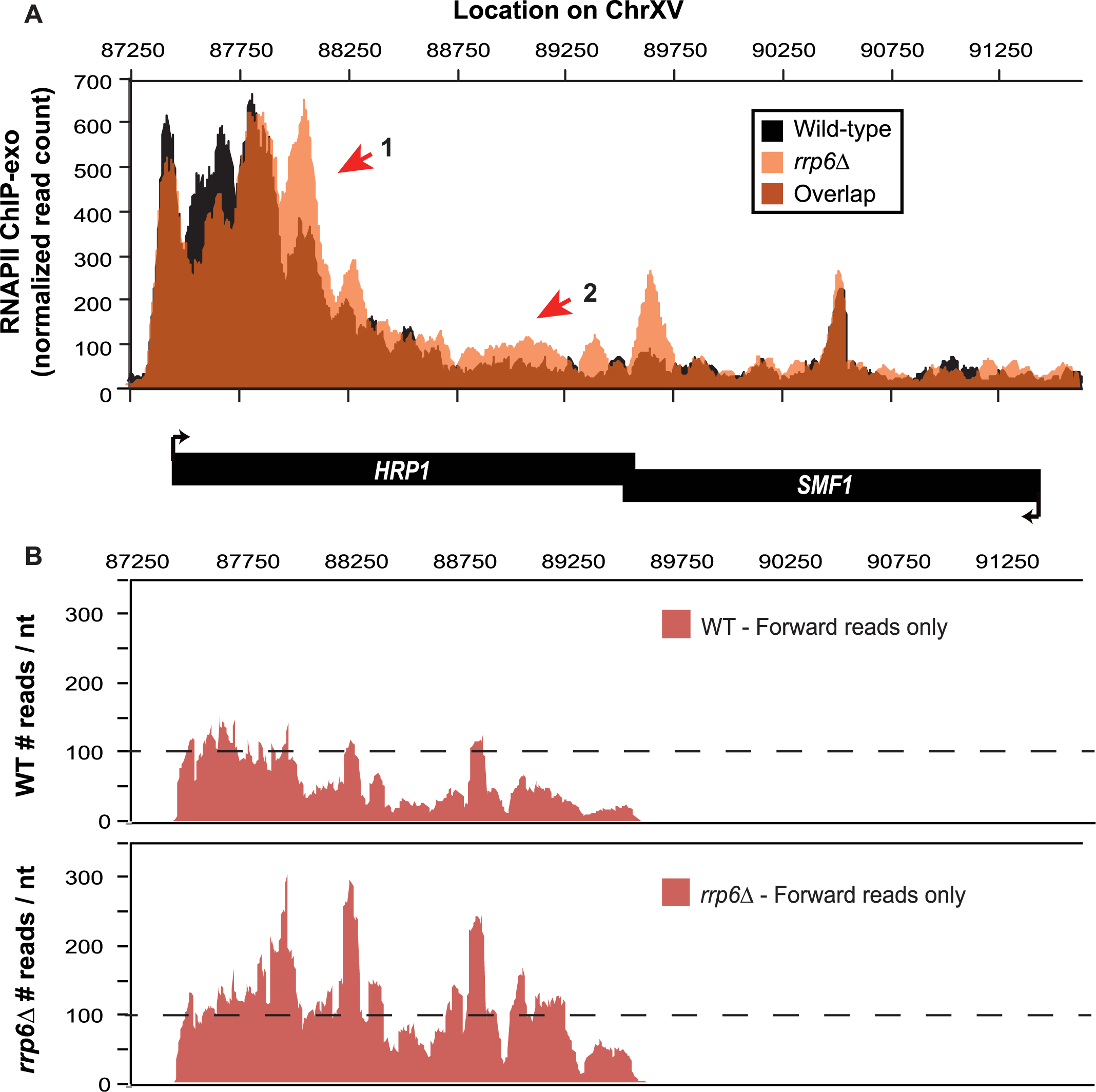

Supplement: S4 Fig — (A) Rpb3-FLAG localization as determined by ChIP-exo sequencing reads mapped to the HRP1 region. Wild-type reads are in black, and rrp6Δ are in orange. The location and direction of transcription for all analyzed annotations are diagrammed below the graphs to scale. Length of mRNAs including untranslated regions are in black. (B) Graphical representation of strand-specific RNA-seq reads mapped to HRP1 transcribed region. Reads mapped to the positive strand are shown in red. No reads were mapped to the reverse strand at HRP1. (TIF) [file pgen.1004999.s004.tif]

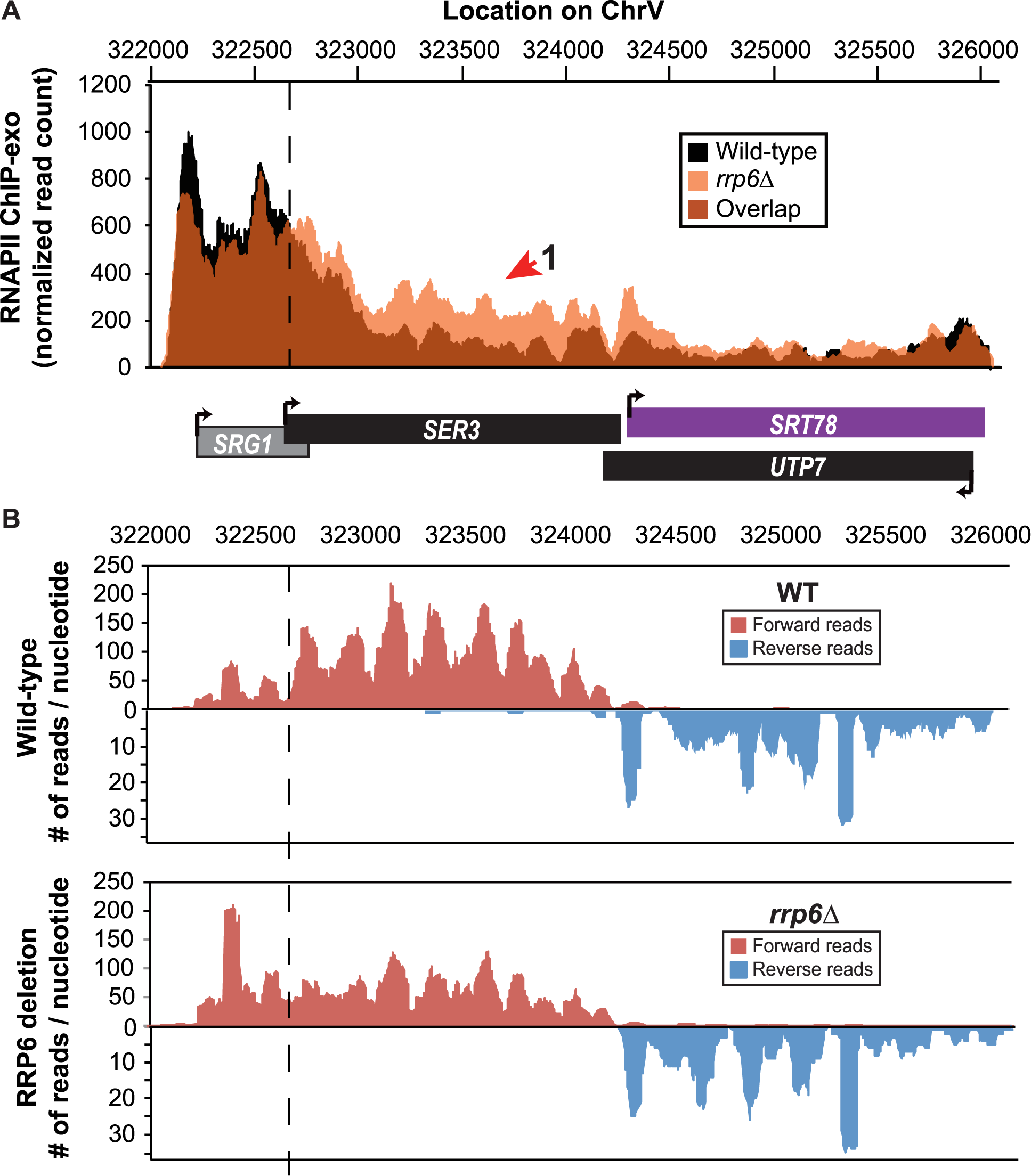

Supplement: S5 Fig — (A) Rpb3-FLAG localization as determined by ChIP-exo sequencing reads mapped to the SRG1-SER3 region. Wild-type normalized read counts are in black, and rrp6Δ are in orange. The location and direction of transcription for all analyzed annotations are diagrammed below the graphs to scale. Length of mRNAs including untranslated regions are in black, CUTs are in gray, and SRTs (Ssu72 regulated transcripts) are in purple. Note that SRG1 and SER3 annotations are overlapping. (B) Graphical representation of strand-specific RNA-seq reads mapped to SRG1-SER3 transcribed region. Reads mapped to the positive strand are shown in red while reverse reads are shown in blue. (TIF) [file pgen.1004999.s005.tif]
