# Supplementary material for: The Exosome Component Rrp6 Is Required for RNA Polymerase II Termination at Specific Targets of the Nrd1-Nab3 Pathway
Source: PLoS Genet. 2015 Feb 13;11(2):e1004999. doi: 10.1371/journal.pgen.1004999 (PMC4378619; doi:10.1371/journal.pgen.1004999)
Supplement: S1 Table — Table includes differential expression data expressed in log2 rrp6Δ/WT ratio (i.e. fold change), as well as p-values and false discovery rate (FDR), all calculated from four replicates by the EdgeR program as discussed in the methods. Class abbreviations are ORF-T: open reading frame transcript, AST: antisense transcript, NUT: Nrd1-unterminated transcript, SRT: Ssu72-restricted transcript, SUT: stable unannotated transcript, CUT: cryptic unstable transcript, sn/snoRNA-ET: Extended region of an sn/snoRNA. Transcript name listed is the systematic name where possible. “AS_” preceding the name designates antisense transcripts, NUTs, CUTs, SUTs, and SRTs are listed as the number provided by their original publications [11, 46, 59]. “N.reads” columns are normalized read counts, calculated by EdgeR. (PDF) [file pgen.1004999.s006.pdf]

TABLE S1: Differential expression data for RRP6 RNA-Seq dataset Page 1

Class	Transcript name	RRP6 KO_vs_WT log2_fold _change	RRP6 KO_vs_WT p-value	RRP6 KO_vs_WT FDR	Ave Norm Reads in WT	Ave Norm Reads in RRP6	Average RAW read counts in WT	Average RAW read counts in RRP6
ORF-T	YOR001W (RRP6)	-5.322832	1.89E-58	1.91E-54	111.33	2.75	1057.75	37.25
ORF-T	YGL251C	-4.591798	2.67E-24	8.17E-22	9.94	0.39	92.50	5.50
AST	AS_YOL013W-B	-3.170474	8.08E-07	9.04E-06	1.44	0.17	13.25	2.00
ORF-T	YPL249C	-3.13285	2.89E-14	2.65E-12	26.66	3.04	232.00	39.50
sn/snoRNA	SNR81	-2.860387	4.85E-14	4.14E-12	995.08	137.05	9074.25	1688.50
ORF-T	YDR034C-A	-2.798024	1.50E-13	1.08E-11	47.24	6.80	413.50	86.75
NUT	NUT0692	-2.791319	7.05E-14	5.74E-12	995.71	143.86	9080.00	1774.75
ORF-T	YAR031W	-2.451657	4.37E-09	9.54E-08	7.99	1.44	70.25	19.25
ORF-T	YER103W	-2.32615	4.03E-06	3.67E-05	160.46	31.99	1279.75	413.00
sn/snoRNA	SNR44	-2.2309	2.77E-07	3.50E-06	5220.43	1112.11	45832.00	13604.25
ORF-T	YHR156C	-2.019987	4.51E-07	5.38E-06	6.27	1.56	54.50	19.25
ORF-T	YBR013C	-2.017959	1.14E-10	3.92E-09	56.14	13.88	499.75	175.00
ORF-T	YDR171W	-2.001422	0.0008857	0.0042168	37.97	9.48	288.25	117.75
ORF-T	YLR367W	-1.992743	1.27E-06	1.34E-05	5592.74	1405.26	49180.50	17254.50
AST	AS_YLR162W-A	-1.970347	0.0002102	0.0011747	40.82	10.43	325.50	124.00
AST	AS_YJL020W-A	-1.932324	0.0004708	0.0023931	2.10	0.56	18.50	6.75
sn/snoRNA	SNR35	-1.928146	2.05E-10	6.41E-09	10831.64	2846.21	96292.00	35870.25
ORF-T	YER011W	-1.922217	0.0092303	0.0324316	7.57	1.97	60.00	28.75
AST	AS_YPL114W	-1.920832	0.0003569	0.0018714	10.48	2.76	92.00	36.25
ORF-T	YPL171C	-1.914988	0.0001876	0.0010604	8.96	2.37	73.50	29.75
NUT	NUT1434	-1.910819	2.81E-10	8.47E-09	10833.07	2880.98	96304.50	36320.00
ORF-T	YKL032C	-1.907229	0.0001095	0.0006639	9.63	2.54	77.25	33.75
ORF-T	YCR089W	-1.897629	0.001805	0.0080009	6.35	1.70	50.00	21.25
ORF-T	YMR120C	-1.850097	4.01E-08	6.58E-07	768.72	213.28	7291.50	2613.25
ORF-T	YPR065W	-1.809528	0.0003084	0.0016396	5.13	1.42	43.50	20.25
AST	AS_YMR119W-A	-1.765623	2.38E-05	0.0001728	22.89	6.78	225.50	82.25
ORF-T	YNL134C	-1.7447	0.0120077	0.0404968	58.38	17.42	459.00	213.50
ORF-T	YOL058W	-1.74129	0.0003437	0.0018111	81.66	24.40	760.75	347.00

TABLE S1: Differential expression data for RRP6 RNA-Seq dataset Page 2

Class	Transcript name	RRP6 KO_vs_WT log2_fold _change	RRP6 KO_vs_WT p-value	RRP6 KO_vs_WT FDR	Ave Norm Reads in WT	Ave Norm Reads in RRP6	Average RAW read counts in WT	Average RAW read counts in RRP6
ORF-T	YBR214W	-1.737246	0.0004136	0.0021354	14.93	4.46	116.50	56.50
ORF-T	YDR403W	-1.732512	0.0002855	0.0015369	1.62	0.47	14.75	6.50
ORF-T	YCR088W	-1.694742	0.0003678	0.0019255	16.34	5.01	136.50	68.50
ORF-T	YPL113C	-1.688753	0.0005479	0.0027398	22.42	6.95	198.50	90.25
ORF-T	YNR044W	-1.685729	0.0063265	0.0237008	8.00	2.48	61.00	30.50
ORF-T	YMR303C	-1.684056	0.0003529	0.0018536	64.86	20.20	644.00	266.75
ORF-T	YOL155C	-1.678131	0.004082	0.0162956	10.43	3.24	86.50	44.25
ORF-T	YGR142W	-1.675193	0.0012067	0.0055479	19.71	6.16	153.25	78.00
ORF-T	YKL102C	-1.650631	0.0024758	0.010503	1.48	0.48	12.50	5.75
AST	AS_YOL037C	-1.648888	0.0033147	0.0135988	2.19	0.68	17.75	9.00
AST	AS_YLR302C	-1.595063	0.0014263	0.0064786	1.42	0.46	12.50	6.00
ORF-T	YNL012W	-1.57915	0.0002548	0.0013944	3.66	1.21	36.25	17.00
ORF-T	YPL240C	-1.563897	0.0016341	0.0073235	501.69	169.68	4187.25	2127.50
NUT	NUT0325	-1.555172	3.80E-05	0.0002609	31.50	10.73	273.50	135.50
ORF-T	YKL187C	-1.539987	0.0006275	0.0030821	8.15	2.80	67.25	34.75
sn/snoRNA	SNR56	-1.523808	6.39E-05	0.0004138	3406.64	1184.72	29931.25	15107.75
AST	AS_YLR057W	-1.517402	0.0009423	0.0044652	3.06	1.10	30.25	13.75
ORF-T	YOR247W	-1.508682	0.0001561	0.0009091	33.55	11.74	282.75	161.25
ORF-T	YBR067C	-1.502809	5.10E-05	0.0003396	69.99	24.72	606.50	304.25
ORF-T	YMR095C	-1.497475	0.0080433	0.0288539	1.25	0.46	12.00	5.75
ORF-T	YDR169C	-1.495477	0.0036037	0.0146294	9.10	3.20	70.75	42.25
NUT	NUT0013	-1.486973	8.06E-05	0.0005055	3408.19	1215.91	29946.25	15520.00
ORF-T	YKL071W	-1.47925	0.0020891	0.0090809	1.82	0.64	17.00	8.75
ORF-T	YDR258C	-1.472432	0.0027535	0.0115355	93.14	33.56	758.00	412.00
ORF-T	YDR528W	-1.463014	0.0088017	0.0310554	5.15	1.88	45.25	23.00
sn/snoRNA	SNR86	-1.462375	8.21E-07	9.17E-06	3620.28	1313.78	31993.75	17022.25
NUT	NUT1320	-1.455211	9.24E-07	1.02E-05	3620.58	1320.43	31996.75	17108.75
ORF-T	YAL005C	-1.451051	0.0032885	0.013502	229.23	83.83	1896.50	1064.75
AST	AS_YOR161C	-1.450695	0.0084866	0.0301548	1.85	0.66	18.25	9.75

TABLE S1: Differential expression data for RRP6 RNA-Seq dataset Page 3

Class	Transcript name	RRP6 KO_vs_WT log2_fold_change	RRP6 KO_vs_WT p-value	RRP6 KO_vs_WT FDR	Ave Norm Reads in WT	Ave Norm Reads in RRP6	Average RAW read counts in WT	Average RAW read counts in RRP6
ORF-T	YGL018C	-1.445919	0.0078969	0.0283985	1.77	0.62	14.75	8.50
sn/snoRNA	SNR65	-1.430942	9.68E-06	7.85E-05	830.70	308.11	7523.00	3934.00
ORF-T	YIL141W	-1.422763	0.0565261	0.140917	1.00	0.36	7.75	4.75
AST	AS_YLR379W	-1.421452	0.000411	0.0021233	7.16	2.65	60.50	34.50
ORF-T	YJL084C	-1.416369	0.0013506	0.0061627	7.90	2.94	65.00	38.00
ORF-T	YIR006C	-1.413925	0.0002266	0.0012591	40.00	14.97	326.75	196.25
ORF-T	YJL045W	-1.409237	0.0130522	0.0435102	7.83	2.94	65.25	37.25
ORF-T	YBR112C	-1.406244	0.0008181	0.0039286	22.17	8.32	182.50	111.50
ORF-T	YJR047C	-1.405293	0.0066492	0.0247056	7.68	2.87	71.00	40.75
ORF-T	YBR296C	-1.404409	0.0465099	0.1212363	717.74	271.16	6675.75	3213.75
AST	AS_YAL004W	-1.40082	0.0045512	0.0178805	67.29	25.47	554.75	324.00
ORF-T	YGR019W	-1.396616	0.006919	0.0255477	74.70	28.36	620.25	358.00
ORF-T	YJR129C	-1.394086	0.0008457	0.0040495	2.73	1.05	24.75	13.25
ORF-T	YER088C	-1.392239	0.0003073	0.001635	23.65	8.99	194.50	115.00
NUT	NUT0082	-1.384104	2.06E-05	0.000152	831.85	318.72	7532.75	4062.50
ORF-T	YKL218C	-1.38288	2.48E-06	2.42E-05	46.41	17.84	429.75	225.25
ORF-T	YGR023W	-1.378708	0.0033454	0.0137022	5.92	2.27	48.25	28.25
ORF-T	YDR216W	-1.377966	0.0010227	0.0047834	51.95	19.97	418.50	252.75
NUT	NUT1203	-1.374523	2.87E-06	2.75E-05	46.48	17.97	430.50	227.25
ORF-T	YLR460C	-1.369008	0.0019686	0.0086308	14.01	5.40	120.00	70.75
ORF-T	YJL159W	-1.367984	0.0001977	0.0011114	222.96	86.37	1882.50	1096.00
ORF-T	YIL123W	-1.367166	1.85E-06	1.87E-05	74.14	28.68	631.00	384.00
sn/snoRNA	SNR78	-1.365953	0.0005821	0.0028889	10863.57	4214.84	93654.50	51692.75
sn/snoRNA	SNR64	-1.364899	2.01E-05	0.0001484	1700.81	660.38	15405.00	8398.25
ORF-T	YKL096W	-1.354703	5.65E-05	0.0003736	42.74	16.72	374.75	210.75
ORF-T	YNL300W	-1.340114	0.0020809	0.0090564	6.62	2.61	57.50	33.25
ORF-T	YPR183W	-1.339491	0.000101	0.0006171	14.55	5.72	123.75	74.50
ORF-T	YAL012W	-1.337111	0.0002974	0.0015929	59.31	23.45	517.25	307.75
ORF-T	YMR194C-B	-1.335755	0.002272	0.00977	4.78	1.89	41.75	24.00

TABLE S1: Differential expression data for RRP6 RNA-Seq dataset Page 4

Class	Transcript name	RRP6 KO_vs_WT log2_fold _change	RRP6 KO_vs_WT p-value	RRP6 KO_vs_WT FDR	Ave Norm Reads in WT	Ave Norm Reads in RRP6	Average RAW read counts in WT	Average RAW read counts in RRP6
AST	AS_YOR041C	-1.334372	9.66E-05	0.0005939	11.57	4.59	105.50	59.25
ORF-T	YLR058C	-1.333002	2.38E-07	3.06E-06	227.58	90.38	2102.75	1144.50
ORF-T	YER091C	-1.330822	0.0206107	0.0629975	177.67	70.63	1499.25	853.00
ORF-T	YJR148W	-1.329996	2.62E-06	2.53E-05	158.68	63.12	1459.50	829.25
SUT	SUT435	-1.322318	0.0070569	0.0259431	2.33	0.92	20.25	12.00
ORF-T	YNL197C	-1.318658	0.0076493	0.0277215	10.65	4.25	85.50	54.25
ORF-T	YPL127C	-1.316763	3.53E-05	0.0002438	16.88	6.74	145.25	89.00
AST	AS_YLR162W	-1.312709	0.0022265	0.0095957	1473.89	593.35	12139.50	7014.50
ORF-T	YIL013C	-1.305602	0.0004271	0.0021928	6.00	2.40	52.00	31.75
ORF-T	YKL164C	-1.305245	0.0027093	0.0113788	44.61	18.02	380.75	238.75
ORF-T	YEL007W	-1.304211	0.0015827	0.0071104	10.50	4.22	87.00	55.75
ORF-T	YER058W	-1.303641	0.0127573	0.042654	1.39	0.56	13.25	7.50
ORF-T	YIL160C	-1.302238	0.0086031	0.0304827	3.01	1.25	29.25	15.25
ORF-T	YDR077W	-1.301417	0.0003822	0.0019918	229.71	93.18	1923.75	1204.25
CUT	CUT289	-1.298512	0.0123576	0.041469	1.85	0.72	16.00	10.25
AST	AS_YGR068W-A	-1.294382	0.0101321	0.0350504	1.13	0.44	10.50	6.25
ORF-T	YJR153W	-1.291662	0.0047273	0.0184696	1.99	0.78	19.00	11.50
ORF-T	YDR096W	-1.287956	0.007582	0.0275421	16.59	6.78	132.50	84.00
AST	AS_YHR201C	-1.286501	0.0162945	0.0521796	1.66	0.67	15.50	9.25
ORF-T	YCL026C-B	-1.286018	0.0468851	0.1218648	1.19	0.47	10.25	6.50
ORF-T	YOR248W	-1.281673	0.0008596	0.0041061	16.79	6.86	142.25	93.50
ORF-T	YBR093C	-1.277853	0.0004455	0.0022756	21.79	8.95	186.75	120.50
ORF-T	YGL205W	-1.276762	0.0044829	0.0176627	4.33	1.77	40.00	24.50
ORF-T	YLR337C	-1.276607	0.0092268	0.0324305	3.32	1.36	27.50	17.25
AST	AS_YPR159C-A	-1.275726	0.0118832	0.0401844	1.83	0.74	17.25	10.50
ORF-T	YML027W	-1.273321	0.0262192	0.0766582	2.73	1.12	23.25	14.25
ORF-T	YFR029W	-1.268847	0.0057956	0.0220456	3.67	1.50	30.75	20.00
ORF-T	YBR212W	-1.262583	0.004029	0.0161289	14.03	5.82	113.75	75.25
ORF-T	YDR207C	-1.25909	0.0052809	0.0203409	5.29	2.19	43.25	28.50

TABLE S1: Differential expression data for RRP6 RNA-Seq dataset Page 5

Class	Transcript name	RRP6 KO_vs_WT log2_fold _change	RRP6 KO_vs_WT p-value	RRP6 KO_vs_WT FDR	Ave Norm Reads in WT	Ave Norm Reads in RRP6	Average RAW read counts in WT	Average RAW read counts in RRP6
ORF-T	YCL044C	-1.256454	0.0039825	0.0159682	4.28	1.76	36.25	23.75
ORF-T	YIL066C	-1.254258	0.0007662	0.0037004	4.54	1.89	40.50	24.75
ORF-T	YJL020C	-1.251916	0.0011221	0.0052048	26.03	10.90	218.75	143.75
ORF-T	YCR061W	-1.251394	0.0366446	0.1006493	4.15	1.74	32.75	21.00
AST	AS_YML090W	-1.250685	0.0253881	0.0746396	4.73	1.99	40.00	24.50
ORF-T	YDL038C	-1.249705	0.0165908	0.0529459	6.57	2.73	52.25	36.75
ORF-T	YNR073C	-1.248886	0.006973	0.0256906	2.04	0.83	18.25	11.50
ORF-T	YBL051C	-1.241246	0.0010667	0.0049747	21.78	9.17	179.25	121.25
ORF-T	YPR171W	-1.237695	0.0013625	0.0062115	6.24	2.62	52.25	34.00
ORF-T	YDR247W	-1.237259	0.0199159	0.0612667	7.55	3.19	59.00	39.25
AST	AS_YHL015W-A	-1.237116	0.015218	0.0493118	1.07	0.43	9.50	6.00
ORF-T	YOR042W	-1.236802	5.68E-05	0.0003747	39.03	16.56	356.00	214.50
sn/snoRNA	SNR84	-1.236072	0.0001251	0.0007484	1229.04	521.78	10752.50	6452.50
ORF-T	YGL189C	-1.234646	0.0004079	0.0021113	327.68	139.20	2746.50	1854.25
ORF-T	YOR383C	-1.234008	0.0021831	0.009424	5.97	2.55	56.00	32.75
ORF-T	YKR091W	-1.232731	0.0006374	0.003123	9.52	4.03	88.25	55.25
ORF-T	YHR143W	-1.227255	0.0041398	0.0164872	29.02	12.36	238.00	162.75
ORF-T	YDR012W	-1.224361	2.50E-05	0.0001799	57.81	24.71	502.75	322.50
ORF-T	YAR015W	-1.223364	0.0002821	0.001521	392.58	168.20	3688.50	2026.75
AST	AS_YBR295W	-1.222247	0.1092485	0.2296086	1.86	0.81	17.50	10.00
ORF-T	YPL085W	-1.221412	0.0005137	0.0025914	36.98	15.83	309.75	204.25
ORF-T	YER032W	-1.220933	0.0063367	0.0237278	4.37	1.84	35.25	24.25
ORF-T	YIL072W	-1.220851	0.0413467	0.1106634	1.21	0.50	11.50	7.25
ORF-T	YBR006W	-1.218242	0.0073217	0.0267507	58.72	25.23	480.75	312.25
ORF-T	YBR072W	-1.217608	0.020616	0.0629975	38.80	16.68	307.25	198.50
sn/snoRNA	SNR33	-1.215933	9.83E-05	0.0006033	766.78	330.10	6650.25	4148.25
ORF-T	YCR102C	-1.215253	0.0015558	0.0070227	4.50	1.94	39.25	24.25
ORF-T	YJL042W	-1.214473	0.0002626	0.0014296	48.63	20.91	402.25	271.00
ORF-T	YCL041C	-1.213838	0.0002769	0.0014971	52.65	22.66	437.00	295.00

TABLE S1: Differential expression data for RRP6 RNA-Seq dataset Page 6

Class	Transcript name	RRP6 KO_vs_WT log2_fold_change	RRP6 KO_vs_WT p-value	RRP6 KO_vs_WT FDR	Ave Norm Reads in WT	Ave Norm Reads in RRP6	Average RAW read counts in WT	Average RAW read counts in RRP6
ORF-T	YDR281C	-1.210906	0.0005968	0.0029474	24.00	10.36	217.50	136.00
ORF-T	YGR204W	-1.206853	2.64E-05	0.0001885	207.69	89.95	1844.25	1170.75
AST	AS_YLR437C-A	-1.206384	0.0117772	0.0398796	4.08	1.73	36.00	24.50
AST	AS_YLR286W-A	-1.198467	0.0055905	0.0213703	25.55	11.08	208.25	150.50
sn/snoRNA	SNR66	-1.19595	9.14E-05	0.0005639	2475.78	1080.65	21606.25	14067.25
ORF-T	YMR181C	-1.195724	0.0086248	0.0305166	4.93	2.13	41.25	27.75
ORF-T	YFL039C	-1.195484	0.0018454	0.0081549	333.29	145.49	2734.50	1876.75
ORF-T	YNL055C	-1.192147	0.0015195	0.0068802	120.99	52.95	1004.00	651.25
ORF-T	YNL327W	-1.190438	0.0147741	0.0480008	49.53	21.67	407.25	285.25
NUT	NUT0947	-1.190062	0.0002112	0.0011791	1230.70	539.42	10767.50	6672.50
ORF-T	YOL040C	-1.187806	0.0001482	0.0008709	190.16	83.45	1619.25	1066.25
ORF-T	YJR095W	-1.187782	0.0513171	0.1305454	10.19	4.48	97.75	57.50
NUT	NUT0034	-1.187691	0.021323	0.0647459	39.34	17.27	312.50	206.25
ORF-T	YKR042W	-1.186616	0.0039802	0.0159682	177.65	78.04	1463.00	972.00
AST	AS_YER060W-A	-1.185689	0.003982	0.0159682	4.09	1.77	34.25	23.00
ORF-T	YOL039W	-1.18084	0.0002859	0.0015386	242.88	107.07	2137.25	1463.50
ORF-T	YKL192C	-1.17875	0.0011191	0.0051956	16.78	7.38	141.25	96.00
ORF-T	YKL180W	-1.178588	0.0002466	0.0013556	134.50	59.38	1125.75	773.25
ORF-T	YGL259W	-1.175283	0.0087962	0.0310467	1.56	0.69	14.50	9.00
ORF-T	YDR309C	-1.172553	0.0015676	0.0070664	10.40	4.59	91.00	60.50
ORF-T	YBR108W	-1.172219	0.0098247	0.0341392	7.12	3.12	58.25	41.50
ORF-T	YJR151C	-1.167519	0.0538294	0.1355685	1.92	0.83	15.00	10.75
ORF-T	YOR085W	-1.167442	0.0033595	0.0137546	18.24	8.09	152.50	105.25
AST	AS_YDR417C	-1.166474	2.56E-05	0.0001838	54.38	24.18	476.25	318.75
ORF-T	YOR163W	-1.16595	0.0078036	0.0281644	8.02	3.52	68.75	49.75
ORF-T	YKL103C	-1.163454	0.0180343	0.0566771	38.31	17.11	326.50	206.50
ORF-T	YDL130W-A	-1.16243	0.0478123	0.1237675	5.72	2.54	45.50	32.00
ORF-T	YOR267C	-1.158291	0.0179862	0.0565906	14.69	6.56	115.75	83.50
ORF-T	YKR093W	-1.156612	0.011015	0.0377165	148.74	66.73	1302.50	823.00

TABLE S1: Differential expression data for RRP6 RNA-Seq dataset Page 7

Class	Transcript name	RRP6 KO_vs_WT log2_fold _change	RRP6 KO_vs_WT p-value	RRP6 KO_vs_WT FDR	Ave Norm Reads in WT	Ave Norm Reads in RRP6	Average RAW read counts in WT	Average RAW read counts in RRP6
ORF-T	YBL066C	-1.155994	0.0041177	0.0164058	8.00	3.57	67.25	46.00
AST	AS_YBR208C	-1.151349	0.0248265	0.0732876	2.31	1.03	22.25	14.25
ORF-T	YLR267W	-1.150696	0.0321542	0.0907735	3.62	1.61	33.25	23.00
ORF-T	YNL040W	-1.148527	2.70E-05	0.0001927	34.49	15.56	324.00	205.00
ORF-T	YIL033C	-1.147961	0.0029012	0.0120892	23.00	10.35	193.00	134.25
SRT	SRT294	-1.147815	0.0001706	0.0009795	14.97	6.78	137.00	86.75
ORF-T	YMR037C	-1.144275	0.0057754	0.0220002	9.91	4.45	82.00	58.75
ORF-T	YMR194C-A	-1.142601	0.0076767	0.0277959	1.86	0.88	18.50	11.00
ORF-T	YLR280C	-1.140836	0.0928148	0.203422	0.97	0.44	8.25	5.25
ORF-T	YLR110C	-1.139826	0.0015795	0.0071041	1231.07	558.62	10475.75	7653.00
ORF-T	YLR185W	-1.138591	7.94E-05	0.0004983	121.76	55.26	1036.75	721.25
ORF-T	YNL202W	-1.135575	0.0010986	0.0051098	9.88	4.51	93.50	59.50
ORF-T	YJR019C	-1.135057	0.0065359	0.0243741	5.17	2.33	43.25	30.50
ORF-T	YKR076W	-1.134468	0.0093656	0.0328155	23.88	10.89	201.75	130.75
ORF-T	YPL248C	-1.132763	0.0088755	0.0312939	6.14	2.77	50.50	36.25
ORF-T	YLR359W	-1.130043	0.0001548	0.0009022	1120.41	511.99	10584.00	6318.25
ORF-T	YLR390W-A	-1.127132	0.0003878	0.0020158	47.47	21.73	404.25	273.75
ORF-T	YIR037W	-1.125182	0.0013446	0.0061381	20.19	9.23	170.25	118.25
ORF-T	YPR129W	-1.124384	0.0019944	0.0087296	16.28	7.43	144.50	100.75
ORF-T	YER175W-A	-1.122636	0.0086489	0.0305804	2.43	1.07	20.50	14.75
SUT	SUT393	-1.122443	0.0002359	0.0013043	14.66	6.76	134.25	86.25
ORF-T	YOR221C	-1.122266	0.013168	0.0437804	3.00	1.35	25.00	17.75
ORF-T	YLR286C	-1.118312	0.0031327	0.0129255	133.49	61.44	1101.25	811.75
ORF-T	YLR216C	-1.116937	0.0031744	0.0130816	79.98	36.84	676.50	483.50
ORF-T	YLL057C	-1.116903	0.0019379	0.0085149	3.98	1.83	37.75	24.75
ORF-T	YAR042W	-1.116331	0.0007799	0.0037612	32.53	14.96	276.50	197.25
ORF-T	YCL043C	-1.115414	0.000331	0.0017513	125.32	57.80	1048.00	749.50
ORF-T	YIL057C	-1.114544	0.026854	0.078197	5.41	2.52	52.00	31.75
ORF-T	YDR418W	-1.112196	1.63E-05	0.0001234	104.22	48.18	913.75	623.50

TABLE S1: Differential expression data for RRP6 RNA-Seq dataset Page 8

Class	Transcript name	RRP6 KO_vs_WT log2_fold _change	RRP6 KO_vs_WT p-value	RRP6 KO_vs_WT FDR	Ave Norm Reads in WT	Ave Norm Reads in RRP6	Average RAW read counts in WT	Average RAW read counts in RRP6
ORF-T	YGL156W	-1.11134	0.0065022	0.0242757	24.85	11.50	216.50	145.50
AST	AS_YNL203C	-1.110934	0.0076565	0.0277327	4.87	2.25	46.50	30.75
AST	AS_YJR038C	-1.108789	0.0168867	0.0537519	1.88	0.86	16.50	11.25
sn/snoRNA	SNR59	-1.107521	0.0142376	0.0466258	22.64	10.49	185.25	133.25
AST	AS_YOR384W	-1.105458	0.0425392	0.1131237	4.74	2.19	37.25	26.50
ORF-T	YDL223C	-1.104963	0.0026905	0.0113094	5.72	2.63	49.00	34.75
ORF-T	YBL092W	-1.103358	0.0002222	0.0012361	442.20	205.78	3784.00	2675.00
ORF-T	YPL092W	-1.101333	0.0001459	0.0008587	65.92	30.70	601.75	411.75
ORF-T	YHL028W	-1.101091	0.0388082	0.1055758	7.03	3.24	57.00	43.50
ORF-T	YPR006C	-1.099644	0.0004532	0.0023095	19.02	8.93	178.25	110.50
SRT	SRT574	-1.099196	0.050238	0.1283181	1.22	0.56	11.25	7.75
ORF-T	YKL185W	-1.098057	0.0026594	0.011202	28.83	13.43	250.50	179.25
ORF-T	YLR378C	-1.097927	0.0017991	0.0079804	34.90	16.28	297.75	209.25
AST	AS_YGR022C	-1.097209	0.0687599	0.1628275	1.60	0.74	13.25	9.25
ORF-T	YGR014W	-1.096664	0.0002056	0.0011516	85.04	39.75	719.00	504.00
ORF-T	YHL033C	-1.096525	2.78E-05	0.0001972	122.06	57.04	1064.25	741.25
ORF-T	YPL089C	-1.092067	0.0010134	0.0047466	11.12	5.18	97.75	69.50
ORF-T	YOR072W-B	-1.090875	0.0147358	0.0479031	336.78	158.09	2934.00	2099.25
NUT	NUT0782	-1.088827	0.000154	0.0008984	66.14	31.06	603.75	417.00
ORF-T	YOR114W	-1.088704	0.0020892	0.0090809	5.12	2.44	48.00	30.75
ORF-T	YGL263W	-1.084478	0.0016945	0.007564	7.25	3.42	65.50	44.00
ORF-T	YLL024C	-1.083716	0.0522571	0.1323024	232.77	109.80	1995.25	1451.75
AST	AS_YHR211W	-1.0822	0.0764288	0.1761215	3.56	1.67	35.25	24.50
NUT	NUT1303	-1.082049	0.0375887	0.1027538	1.62	0.79	15.75	10.00
AST	AS_YOR139C	-1.081853	0.0182086	0.0570587	3.11	1.43	25.75	19.50
ORF-T	YCR091W	-1.0768	0.0583879	0.1441596	5.86	2.76	46.25	34.50
ORF-T	YLR050C	-1.076629	8.44E-05	0.0005255	15.21	7.19	136.75	94.50
ORF-T	YER131W	-1.075197	0.0001519	0.0008904	158.45	75.16	1363.50	982.75
ORF-T	YBR085W	-1.073557	0.0086026	0.0304827	5.09	2.40	48.75	33.00

TABLE S1: Differential expression data for RRP6 RNA-Seq dataset Page 9

Class	Transcript name	RRP6 KO_vs_WT log2_fold _change	RRP6 KO_vs_WT p-value	RRP6 KO_vs_WT FDR	Ave Norm Reads in WT	Ave Norm Reads in RRP6	Average RAW read counts in WT	Average RAW read counts in RRP6
ORF-T	YML054C	-1.071871	0.0158985	0.0511063	8.42	4.04	75.00	48.00
ORF-T	YOR023C	-1.071062	0.0045517	0.0178805	6.89	3.25	57.50	42.00
ORF-T	YCR084C	-1.070003	0.0012071	0.0055479	31.19	14.81	266.25	195.25
ORF-T	YJR112W	-1.068629	0.0019984	0.0087426	14.43	6.84	135.00	95.50
ORF-T	YCR031C	-1.067605	0.0002575	0.0014058	132.77	63.30	1119.00	819.00
ORF-T	YHR145C	-1.06533	0.0019359	0.0085099	19.13	9.10	166.00	121.75
ORF-T	YJL083W	-1.063104	0.0010464	0.004885	9.30	4.40	79.50	58.25
ORF-T	YJR117W	-1.062478	0.0078633	0.0283294	22.09	10.56	182.50	133.25
ORF-T	YLR070C	-1.062336	0.0020796	0.0090548	7.47	3.63	70.50	44.75
ORF-T	YOL127W	-1.061722	0.0006462	0.00316	330.46	158.27	2812.00	2056.00
ORF-T	YIL006W	-1.060455	0.0161908	0.0518971	4.74	2.22	38.75	30.50
ORF-T	YOR313C	-1.060311	0.0091605	0.0322087	3.82	1.83	37.00	25.25
ORF-T	YLR439W	-1.058268	0.0144669	0.047227	2.52	1.17	21.75	16.00
ORF-T	YPL014W	-1.05545	0.0889754	0.1969332	5.05	2.42	39.25	30.00
ORF-T	YHL034C	-1.054428	0.006144	0.0231353	177.58	85.49	1583.25	1110.00
ORF-T	YBR078W	-1.054384	0.0011122	0.0051706	222.50	107.09	1850.00	1382.25
ORF-T	YER057C	-1.054007	0.0039219	0.0157688	13.96	6.73	119.50	82.25
ORF-T	YFR047C	-1.053369	0.000241	0.0013289	37.47	18.05	335.75	233.00
ORF-T	YNL176C	-1.053267	0.0030498	0.0126351	14.02	6.74	118.25	85.50
NUT	NUT0435	-1.052524	0.0002315	0.001284	2147.48	1035.36	19443.75	13232.00
AST	AS_YPL250W-A	-1.05176	0.0007134	0.0034651	132.20	63.73	1145.50	835.00
ORF-T	YKL141W	-1.051542	0.0211078	0.0642279	16.16	7.78	133.00	99.25
ORF-T	YCL050C	-1.050036	0.0024616	0.0104471	18.70	9.00	159.00	116.50
ORF-T	YNL068C	-1.04989	0.0036863	0.0149107	9.19	4.41	78.00	56.75
ORF-T	YKL204W	-1.048988	0.0125764	0.042147	8.16	3.90	66.75	51.50
sn/snoRNA	SNR189	-1.048743	0.0019309	0.0084991	1057.47	511.18	9349.00	6379.00
ORF-T	YDL181W	-1.048436	0.042331	0.1127187	5.90	2.82	46.50	36.50
ORF-T	YLR131C	-1.045534	0.0116548	0.0395582	10.39	4.99	86.75	67.00
AST	AS_YHL034W-A	-1.044577	0.0060922	0.0229745	108.56	52.62	961.50	681.50

TABLE S1: Differential expression data for RRP6 RNA-Seq dataset Page 10

Class	Transcript name	RRP6 KO_vs_WT log2_fold _change	RRP6 KO_vs_WT p-value	RRP6 KO_vs_WT FDR	Ave Norm Reads in WT	Ave Norm Reads in RRP6	Average RAW read counts in WT	Average RAW read counts in RRP6
ORF-T	YEL068C	-1.044563	0.0057165	0.0218024	4.58	2.16	39.00	29.75
ORF-T	YJR045C	-1.044234	0.0054374	0.0208562	242.87	117.74	1994.00	1480.75
ORF-T	YMR173W-A	-1.043889	0.0770148	0.1771885	21.89	10.60	176.25	135.25
ORF-T	YMR279C	-1.042176	0.0363438	0.100052	1.34	0.65	12.75	8.75
SUT	SUT171	-1.037268	0.0156459	0.050423	1.56	0.76	13.50	9.50
ORF-T	YGL128C	-1.036785	0.0369789	0.1014404	1.72	0.81	14.75	11.00
AST	AS_YPR130C	-1.036701	0.0074168	0.0270492	5.11	2.47	44.25	32.25
ORF-T	YMR242C	-1.035553	0.0010762	0.0050125	150.64	73.44	1291.75	972.75
ORF-T	YPR002W	-1.034836	0.0002991	0.0015976	15.50	7.58	146.25	100.25
AST	AS_YLR217W	-1.034411	0.0099422	0.0344643	27.03	13.16	226.00	173.25
ORF-T	YOR081C	-1.033427	0.0204355	0.0625407	7.22	3.51	60.25	44.75
ORF-T	YML073C	-1.032985	0.000519	0.002613	221.52	108.24	1923.50	1386.25
ORF-T	YMR306W	-1.032703	0.001122	0.0052048	11.69	5.67	106.25	78.25
SUT	SUT453	-1.032386	0.1867021	0.3355978	3.19	1.58	29.75	18.75
ORF-T	YOR184W	-1.031898	0.0004093	0.0021176	334.78	163.76	3089.75	2067.75
ORF-T	YFR036W	-1.028825	0.0235334	0.0700031	1.64	0.79	14.75	10.50
ORF-T	YFR051C	-1.028292	0.0013078	0.0059781	23.20	11.33	200.50	150.50
ORF-T	YLR356W	-1.028055	0.0433252	0.1147599	2.33	1.13	19.25	14.25
ORF-T	YFL022C	-1.026805	0.0035855	0.0145673	11.93	5.85	105.25	75.25
ORF-T	YOR020C	-1.026458	0.0042757	0.0169549	38.54	18.89	325.00	242.50
ORF-T	YBL101C	-1.026233	0.0098785	0.0342671	17.66	8.67	147.50	107.00
ORF-T	YJR044C	-1.025704	0.0097935	0.0340645	8.53	4.15	70.75	54.50
ORF-T	YDR533C	-1.024266	0.0051295	0.0198648	20.39	10.00	171.75	128.00
ORF-T	YMR296C	-1.020639	0.0009431	0.0044671	42.23	20.76	353.50	271.25
ORF-T	YDR539W	-1.015567	0.0004917	0.0024904	45.77	22.65	417.50	289.00
ORF-T	YPR181C	-1.011772	0.0033676	0.013782	65.89	32.62	554.25	436.00
ORF-T	YGL234W	-1.008386	0.0013062	0.0059736	1014.11	504.16	9622.00	6439.75
SRT	SRT555	-1.008308	0.0185798	0.0579878	1.79	0.86	16.25	12.00
ORF-T	YGR061C	-1.007903	0.0002208	0.001229	1296.01	644.51	12317.75	8325.75

TABLE S1: Differential expression data for RRP6 RNA-Seq dataset Page 11

Class	Transcript name	RRP6 KO_vs_WT log2_fold_change	RRP6 KO_vs_WT p-value	RRP6 KO_vs_WT FDR	Ave Norm Reads in WT	Ave Norm Reads in RRP6	Average RAW read counts in WT	Average RAW read counts in RRP6
ORF-T	YPR047W	-1.005643	0.0067986	0.0251769	16.56	8.23	150.75	108.50
ORF-T	YLR042C	-1.005392	0.0624858	0.1516137	2.39	1.17	22.00	16.25
AST	AS_YER067C-A	-1.003976	0.0702213	0.1654466	24.31	12.10	195.25	152.25
sn/snoRNA ET	SNR44-ET-RPS22B	-1.003942	0.0023839	0.0101818	504.50	251.58	4363.00	3087.50
ORF-T	YMR016C	-1.00347	0.0208167	0.0634423	11.93	5.92	95.75	75.50
ORF-T	YOR183W	-1.002316	0.0005179	0.0026086	205.17	102.46	1905.25	1292.75
ORF-T	YMR179W	-1.000063	0.0007837	0.0037759	26.61	13.29	241.50	173.75
ORF-T	YKR009C	-0.995739	0.0031501	0.0129921	10.39	5.20	91.75	67.25
ORF-T	YDL140C	-0.990939	0.002437	0.0103735	297.61	149.70	2502.25	1936.00
ORF-T	YER067W	-0.989837	0.0592891	0.145671	31.94	16.07	258.00	202.50
AST	AS_YLL044W	-0.989318	0.0003881	0.0020163	60.76	30.56	530.25	400.00
ORF-T	YKL217W	-0.988914	0.0107484	0.0369668	69.48	35.02	646.25	457.00
ORF-T	YDR510W	-0.98885	0.0032768	0.0134596	15.15	7.60	129.00	97.75
ORF-T	YGR221C	-0.988703	0.033614	0.0939477	2.49	1.22	21.50	16.75
ORF-T	YMR274C	-0.985809	0.0076506	0.0277215	3.18	1.59	28.75	21.00
ORF-T	YPL249C-A	-0.983926	0.0006543	0.0031965	190.18	96.12	1652.75	1250.75
ORF-T	YNL117W	-0.981055	0.0607687	0.1483918	15.57	7.90	152.50	105.50
ORF-T	YAL034C	-0.980186	0.016319	0.0522248	7.32	3.69	60.75	46.75
ORF-T	YNL072W	-0.979803	0.0044501	0.0175575	5.38	2.71	48.75	36.00
ORF-T	YLR438W	-0.979713	0.0079125	0.0284355	24.59	12.41	215.00	172.50
ORF-T	YOR382W	-0.975818	0.0321098	0.0906734	8.85	4.51	85.00	58.75
ORF-T	YMR116C	-0.974223	0.0129478	0.0432193	5438.38	2768.20	47410.00	35393.50
ORF-T	YNL042W-B	-0.973902	0.005764	0.0219669	104.63	53.27	924.75	679.50
ORF-T	YDL224C	-0.973774	0.012263	0.0412201	16.02	8.11	132.00	108.25
ORF-T	YHR008C	-0.973186	0.0250102	0.0737436	23.44	11.93	196.50	148.00
ORF-T	YMR305C	-0.971307	0.0008303	0.0039814	172.37	87.94	1540.75	1113.75
ORF-T	YOR177C	-0.969895	0.0411896	0.11042	1.65	0.82	15.25	11.50
ORF-T	YNL309W	-0.96951	0.0237541	0.0705557	3.83	1.93	33.00	25.25
ORF-T	YMR251W	-0.968673	0.0898511	0.1981902	2.01	1.05	19.00	13.25

TABLE S1: Differential expression data for RRP6 RNA-Seq dataset Page 12

Class	Transcript name	RRP6 KO_vs_WT log2_fold _change	RRP6 KO_vs_WT p-value	RRP6 KO_vs_WT FDR	Ave Norm Reads in WT	Ave Norm Reads in RRP6	Average RAW read counts in WT	Average RAW read counts in RRP6
ORF-T	YPL163C	-0.967698	0.004577	0.0179659	17.57	9.01	161.50	114.25
ORF-T	YLR187W	-0.966155	0.0105879	0.0364894	6.95	3.52	59.75	46.75
ORF-T	YPL079W	-0.964466	0.0001339	0.0007949	132.80	68.01	1167.50	894.00
ORF-T	YER120W	-0.964091	0.0019271	0.008486	139.64	71.49	1182.00	987.25
ORF-T	YNL066W	-0.963885	0.0007609	0.0036764	66.96	34.26	578.50	458.50
ORF-T	YML091C	-0.961694	0.0074445	0.0271208	83.04	42.61	694.00	540.00
ORF-T	YPR001W	-0.960662	0.0080518	0.0288742	7.29	3.74	72.00	52.25
AST	AS_YJL187C	-0.960163	0.0243203	0.0719617	2.68	1.38	26.25	19.00
ORF-T	YMR173W	-0.959309	0.0870093	0.1938153	27.41	14.08	221.00	177.00
ORF-T	YBR188C	-0.958386	0.0119555	0.0403613	2.78	1.42	26.00	19.25
ORF-T	YBR162C	-0.958012	0.0003806	0.0019843	131.49	67.65	1147.00	886.75
ORF-T	YGR097W	-0.956686	0.0067285	0.0249724	24.56	12.62	203.00	162.50
ORF-T	YFL034C-A	-0.955394	0.0012336	0.0056621	18.99	9.74	169.75	131.50
AST	AS_YKL192C	-0.95493	0.1078844	0.2276908	1.58	0.81	15.75	11.50
ORF-T	YKR102W	-0.953398	0.0756813	0.174999	1.52	0.74	12.50	10.50
ORF-T	YOL111C	-0.95312	0.0073561	0.0268474	9.35	4.78	80.50	63.75
AST	AS_YIR036W-A	-0.95131	0.0084177	0.0299414	12.12	6.26	109.50	81.25
ORF-T	YAL044C	-0.950936	0.0001716	0.0009837	94.71	49.02	891.25	632.75
ORF-T	YOL013W-A	-0.949886	0.008782	0.0310184	28.27	14.61	241.25	188.75
ORF-T	YDR025W	-0.949811	0.0016071	0.0072154	118.02	61.07	1014.50	783.50
ORF-T	YJL078C	-0.949211	0.0468398	0.1218337	7.85	4.02	63.25	54.00
ORF-T	YGL242C	-0.948783	0.0017673	0.0078578	20.35	10.50	184.50	141.75
ORF-T	YIL052C	-0.948519	0.0009384	0.0044511	122.08	63.22	1060.75	826.00
ORF-T	YJR073C	-0.946753	0.0017969	0.0079788	23.89	12.36	222.25	169.50
AST	AS_YBL113W-A	-0.945884	0.0590576	0.1453205	1.17	0.59	10.50	8.00
AST	AS_YAR035W	-0.94442	0.0935718	0.2047251	2.05	1.07	18.75	13.25
AST	AS_YNL057W	-0.944323	0.0620342	0.1507357	2.66	1.32	22.00	19.25
ORF-T	YOR074C	-0.944117	0.0055114	0.0211159	6.93	3.59	62.50	47.00
ORF-T	YIR038C	-0.943631	0.0058069	0.0220718	15.60	8.09	132.50	102.75

TABLE S1: Differential expression data for RRP6 RNA-Seq dataset Page 13

Class	Transcript name	RRP6 KO_vs_WT log2_fold _change	RRP6 KO_vs_WT p-value	RRP6 KO_vs_WT FDR	Ave Norm Reads in WT	Ave Norm Reads in RRP6	Average RAW read counts in WT	Average RAW read counts in RRP6
ORF-T	YOL120C	-0.943612	0.0004191	0.0021613	1510.91	785.55	13500.25	10209.00
ORF-T	YLL045C	-0.943453	0.000723	0.0035101	132.67	68.95	1151.25	890.75
ORF-T	YHR025W	-0.94339	0.0005959	0.0029474	34.53	17.92	303.00	235.25
ORF-T	YLR234W	-0.943369	0.0015255	0.0069013	13.29	6.91	123.00	91.75
ORF-T	YEL040W	-0.943272	0.01979	0.0609722	144.78	75.24	1260.75	1033.75
ORF-T	YCR065W	-0.943262	0.0006732	0.0032812	22.27	11.53	196.00	153.00
ORF-T	YBR248C	-0.943091	0.0001357	0.0008047	145.39	75.63	1366.25	997.50
ORF-T	YKL109W	-0.94172	0.0401442	0.1082681	46.57	24.23	392.25	304.50
ORF-T	YER043C	-0.939265	0.0006942	0.0033753	344.68	179.71	3074.00	2384.75
ORF-T	YNL096C	-0.938209	0.0005994	0.0029573	81.29	42.41	702.00	539.25
ORF-T	YIR028W	-0.937632	0.0215181	0.0652205	2.24	1.15	20.25	15.50
ORF-T	YOR140W	-0.936137	0.0133078	0.0441435	10.00	5.18	82.00	68.25
ORF-T	YNL302C	-0.93574	0.0013151	0.0060089	653.52	341.65	5784.00	4308.00
AST	AS_YDR320W-B	-0.934989	0.0542837	0.13644	0.90	0.48	8.50	6.25
ORF-T	YDL105W	-0.933662	0.0098453	0.0341874	5.81	2.99	51.25	40.75
ORF-T	YGL215W	-0.931833	0.0029133	0.0121345	35.28	18.44	296.50	239.50
AST	AS_YML057C-A	-0.931568	0.041999	0.1120257	2.84	1.46	24.25	19.50
AST	AS_YER119C-A	-0.930966	0.0034723	0.0141644	12.54	6.53	110.50	88.25
ORF-T	YER056C-A	-0.930519	0.0009908	0.0046557	102.92	53.97	878.00	687.50
ORF-T	YER102W	-0.928462	0.0006585	0.0032155	237.27	124.63	2119.00	1639.00
AST	AS_YNL144C	-0.928441	0.0273991	0.0796003	1.74	0.90	16.50	12.50
ORF-T	YOR027W	-0.928308	0.0066157	0.0246057	39.09	20.50	345.25	274.75
ORF-T	YDR540C	-0.928187	0.0044369	0.0175319	6.99	3.65	62.75	48.75
AST	AS_YML034C-A	-0.928002	0.0165432	0.0528417	5.41	2.81	45.50	36.50
ORF-T	YJL213W	-0.926674	0.0014132	0.0064249	23.05	12.10	208.50	160.75
ORF-T	YDL037C	-0.92587	0.0520511	0.1318472	4.92	2.55	40.50	34.00
ORF-T	YKL006W	-0.92515	0.0015766	0.0070944	398.59	209.87	3510.50	2755.75
ORF-T	YLR151C	-0.924444	0.0103396	0.0356705	12.00	6.35	106.25	77.25
ORF-T	YER087C-A	-0.923882	0.0433551	0.1148091	2.52	1.31	23.00	18.00

Class	Transcript name	RRP6 KO_vs_WT log2_fold _change	RRP6 KO_vs_WT p-value	RRP6 KO_vs_WT FDR	Ave Norm Reads in WT	Ave Norm Reads in RRP6	Average RAW read counts in WT	Average RAW read counts in RRP6
ORF-T	YLR180W	-0.922879	0.0512617	0.1304457	480.00	253.19	4355.00	3121.00
ORF-T	YGL083W	-0.922233	0.0136731	0.0451178	6.45	3.37	56.00	45.00
ORF-T	YER072W	-0.921665	0.0044353	0.0175319	61.68	32.50	539.00	439.75
ORF-T	YDR233C	-0.919924	0.0038318	0.0154684	54.01	28.49	449.75	375.00
ORF-T	YFR007W	-0.919391	0.0066409	0.0246836	7.34	3.87	67.25	50.75
ORF-T	YPR035W	-0.919091	0.0008477	0.004057	516.86	273.30	4725.50	3680.00
ORF-T	YGR056W	-0.918443	0.0154602	0.0499201	7.21	3.78	60.75	50.00
ORF-T	YNL322C	-0.918295	0.0002982	0.0015954	48.63	25.68	428.75	339.25
ORF-T	YCR093W	-0.918027	0.0039415	0.0158415	98.18	51.89	839.25	701.50
ORF-T	YPL253C	-0.916452	0.0227702	0.0681553	4.29	2.23	36.75	30.25
ORF-T	YDR506C	-0.916395	0.007457	0.0271468	16.38	8.66	143.75	111.75
ORF-T	YDL211C	-0.915713	0.0075539	0.0274501	5.78	3.07	51.75	39.25
AST	AS_YPL248C	-0.915229	0.0028359	0.0118413	19.81	10.54	181.75	132.50
AST	AS_YPR044C	-0.915014	0.0027163	0.0113986	179.89	95.35	1557.00	1258.00
ORF-T	YNL321W	-0.914178	0.010632	0.036604	15.07	7.96	125.75	103.00
ORF-T	YBL042C	-0.914077	0.0059801	0.022628	114.76	60.96	1046.25	736.50
ORF-T	YLR037C	-0.913421	0.0704173	0.1658309	1.34	0.73	13.50	9.50
ORF-T	YDL057W	-0.913396	0.0030844	0.0127448	9.91	5.29	94.25	68.75
AST	AS_YGL074C	-0.913242	0.0070112	0.0258032	3.66	1.96	32.75	24.50
AST	AS_YJL202C	-0.912989	0.0375298	0.1026447	2.02	1.04	18.25	14.50
ORF-T	YFR045W	-0.91212	0.081938	0.1855936	2.54	1.33	20.75	17.00
ORF-T	YIL034C	-0.911256	0.0100031	0.03464	19.69	10.43	167.25	136.75
ORF-T	YBR011C	-0.910945	0.0185021	0.0577991	119.97	63.76	1020.50	845.75
AST	AS_YNR072W	-0.910716	0.0746669	0.1730501	2.87	1.48	22.75	20.50
ORF-T	YGL127C	-0.910612	0.0353644	0.0979442	2.86	1.51	24.75	19.25
sn/snoRNA	SNR39B	-0.909028	0.0390399	0.1060842	106.53	56.68	844.75	750.75
ORF-T	YMR292W	-0.908367	0.0016938	0.007564	17.41	9.28	155.25	117.50
ORF-T	YBL085W	-0.908109	0.0022619	0.0097308	22.96	12.18	198.75	161.25
ORF-T	YMR199W	-0.906596	0.0040945	0.0163328	14.61	7.77	130.50	102.75

Class	Transcript name	RRP6 KO_vs_WT log2_fold _change	RRP6 KO_vs_WT p-value	RRP6 KO_vs_WT FDR	Ave Norm Reads in WT	Ave Norm Reads in RRP6	Average RAW read counts in WT	Average RAW read counts in RRP6
ORF-T	YIL140W	-0.90643	0.002444	0.0103888	24.58	13.11	213.50	165.75
ORF-T	YFR044C	-0.905908	0.0202667	0.062156	33.96	18.08	297.25	247.00
ORF-T	YPL271W	-0.905666	0.0328471	0.0924449	12.12	6.46	101.75	79.25
SUT	SUT613	-0.905658	0.0808908	0.1837718	1.14	0.61	10.50	8.00
SRT	SRT445	-0.905658	0.080915	0.1837718	1.14	0.61	10.50	8.00
ORF-T	YNL278W	-0.905493	0.0040606	0.0162426	8.86	4.71	77.50	61.00
ORF-T	YGR249W	-0.904208	0.0452144	0.1186423	5.86	3.09	47.50	40.50
ORF-T	YFR021W	-0.903226	0.0077087	0.027892	11.27	5.98	97.50	79.75
ORF-T	YNL178W	-0.901668	0.0025041	0.0106052	1012.69	542.03	9113.75	7157.75
ORF-T	YGR208W	-0.899085	0.0005319	0.0026699	52.88	28.39	484.75	361.50
sn/snoRNA	SNR191	-0.896795	0.0204168	0.0625026	2389.58	1283.41	20630.25	15784.75
AST	AS_YOR329W-A	-0.896624	0.0328614	0.0924594	2.04	1.08	17.00	13.50
AST	AS_YLR376C	-0.896226	0.0844793	0.1897303	1.59	0.85	14.00	11.00
ORF-T	YBL087C	-0.892906	0.002011	0.0087862	769.75	414.56	7141.75	5264.75
ORF-T	YAR035W	-0.892905	0.0709975	0.1668855	4.84	2.59	44.25	35.50
ORF-T	YIL056W	-0.892845	0.0467133	0.1216572	7.05	3.77	58.00	48.75
AST	AS_YDL159C-B	-0.892724	0.0340554	0.0949089	1.61	0.85	14.75	11.50
ORF-T	YDL210W	-0.891116	0.0452103	0.1186423	5.86	3.17	55.00	41.75
ORF-T	YAL007C	-0.890447	0.0020737	0.0090409	20.21	10.88	176.00	139.25
ORF-T	YNR017W	-0.890255	0.0028839	0.012022	19.55	10.55	174.75	135.25
ORF-T	YGL031C	-0.88995	0.0016174	0.0072585	149.34	80.57	1308.25	1032.75
SRT	SRT27	-0.889301	0.0828537	0.1871633	1.79	0.98	16.75	12.50
ORF-T	YJL080C	-0.889079	0.0010738	0.0050038	249.59	134.72	2232.25	1786.25
SUT	SUT212	-0.887926	0.0772235	0.1773854	1.62	0.86	14.25	11.50
ORF-T	YAL024C	-0.886129	0.0072351	0.0264821	8.90	4.76	75.25	63.25
ORF-T	YER070W	-0.886011	0.0031038	0.012817	103.57	56.05	938.25	712.50
ORF-T	YLR048W	-0.885436	0.0023533	0.010064	559.95	303.09	4859.25	3908.25
ORF-T	YIL133C	-0.885136	0.0004631	0.0023563	100.61	54.44	885.75	708.75
ORF-T	YPR043W	-0.884017	0.002664	0.0112166	198.89	107.72	1729.75	1418.50

TABLE S1: Differential expression data for RRP6 RNA-Seq dataset Page 16

Class	Transcript name	RRP6 KO_vs_WT log2_fold _change	RRP6 KO_vs_WT p-value	RRP6 KO_vs_WT FDR	Ave Norm Reads in WT	Ave Norm Reads in RRP6	Average RAW read counts in WT	Average RAW read counts in RRP6
AST	AS_YPL197C	-0.883663	0.0024098	0.0102708	15.55	8.39	135.00	110.00
ORF-T	YPL119C-A	-0.883567	0.0315107	0.0892547	5.31	2.90	47.00	35.25
ORF-T	YGL071W	-0.883367	0.0060496	0.0228478	10.17	5.46	87.25	72.50
ORF-T	YOL121C	-0.883042	0.0010284	0.0048079	239.76	129.98	2109.25	1669.25
ORF-T	YMR304C-A	-0.882236	0.011838	0.0400721	37.31	20.28	341.75	253.00
ORF-T	YPR158W	-0.881348	0.0223952	0.0672509	13.26	7.18	112.25	91.25
ORF-T	YOR185C	-0.881321	0.0218551	0.0660238	9.63	5.20	80.00	65.50
ORF-T	YDL227C	-0.881298	0.0633204	0.1530862	38.55	20.91	361.75	284.75
ORF-T	YJR120W	-0.881105	0.0491156	0.1262186	9.41	5.07	78.25	66.75
ORF-T	YKL096W-A	-0.88107	0.0040253	0.0161205	420.87	228.46	3689.00	3083.25
ORF-T	YIL016W	-0.880626	0.0266679	0.0777448	9.31	5.01	83.50	69.75
ORF-T	YGR241C	-0.880441	0.0075277	0.0273646	13.78	7.44	118.00	98.50
ORF-T	YKL042W	-0.879397	0.0118628	0.0401423	4.52	2.42	39.50	32.25
ORF-T	YNL145W	-0.877814	0.0085501	0.030328	64.32	34.98	550.25	447.00
ORF-T	YDR113C	-0.87776	0.0085085	0.0302117	5.80	3.14	53.75	42.00
ORF-T	YLR194C	-0.877034	0.0013546	0.0061779	36.35	19.77	342.50	268.75
AST	AS_YGL091C	-0.875788	0.0709202	0.166766	3.20	1.75	32.00	23.50
ORF-T	YER074W	-0.87574	0.0058453	0.0221935	342.81	186.80	2991.50	2403.75
NUT	NUT0103	-0.87491	0.0017399	0.0077463	89.91	48.99	786.00	640.00
AST	AS_YPL251W	-0.874182	0.0477031	0.1235966	1.98	1.06	17.75	14.25
AST	AS_YDR510C-A	-0.874157	0.0280981	0.0812331	5.96	3.23	51.50	42.00
ORF-T	YKL034W	-0.873917	0.0420984	0.1121881	7.29	3.93	58.50	51.50
ORF-T	YGR157W	-0.873089	0.0411636	0.1103972	278.41	152.00	2526.75	1957.50
ORF-T	YKR039W	-0.873043	0.0811892	0.1841431	52.00	28.41	474.50	355.50
ORF-T	YKL043W	-0.873012	0.0109295	0.0374619	13.71	7.44	120.00	99.25
ORF-T	YGL032C	-0.872983	0.0671901	0.1600595	8.95	4.86	74.25	63.50
ORF-T	YDL198C	-0.872211	0.0009729	0.0045867	31.69	17.25	278.00	229.25
ORF-T	YGR109C	-0.871965	0.1881883	0.3371875	2.74	1.51	23.25	17.50
ORF-T	YER012W	-0.871457	0.0505142	0.1288277	21.95	11.96	183.50	159.50

Class	Transcript name	RRP6 KO_vs_WT log2_fold _change	RRP6 KO_vs_WT p-value	RRP6 KO_vs_WT FDR	Ave Norm Reads in WT	Ave Norm Reads in RRP6	Average RAW read counts in WT	Average RAW read counts in RRP6
ORF-T	YBR265W	-0.870888	0.0003741	0.0019545	40.58	22.13	366.00	297.50
ORF-T	YOR375C	-0.87015	0.0016565	0.0074141	558.55	305.62	5091.25	3806.25
ORF-T	YBR189W	-0.869864	0.0024119	0.0102754	689.93	377.49	6196.00	5024.25
ORF-T	YLR183C	-0.867976	0.0034881	0.014223	26.26	14.42	240.25	180.75
AST	AS_YML101C	-0.867053	0.0771007	0.1772649	1.86	1.03	18.25	13.75
ORF-T	YOR352W	-0.866902	0.0067941	0.0251758	15.17	8.31	135.00	106.25
ORF-T	YHR205W	-0.866814	0.0140115	0.0460233	21.58	11.80	185.75	156.00
ORF-T	YDR500C	-0.866716	0.0017226	0.0076793	203.10	111.34	1771.00	1446.25
ORF-T	YIL026C	-0.86589	0.0003477	0.0018296	47.08	25.85	432.25	333.00
ORF-T	YHR193C	-0.865738	0.0043434	0.0171962	117.17	64.24	1008.50	848.00
ORF-T	YGL170C	-0.863238	0.1088784	0.2290646	1.82	1.01	17.50	13.25
ORF-T	YGL226C-A	-0.863174	0.0136288	0.0450011	13.06	7.12	108.00	93.75
ORF-T	YJL019W	-0.862717	0.0005624	0.0027993	31.53	17.30	286.50	231.75
ORF-T	YJL219W	-0.862601	0.0595955	0.1461837	1.83	1.00	15.75	12.50
ORF-T	YPL119C	-0.86112	0.0203739	0.062428	30.12	16.61	269.75	203.50
ORF-T	YOR181W	-0.861105	0.0168344	0.0536022	7.57	4.14	64.25	53.25
SUT	SUT514	-0.861041	0.0058942	0.0223364	10.74	5.90	93.00	74.75
ORF-T	YCR030C	-0.860497	0.0399035	0.1078329	16.43	9.02	135.00	116.00
ORF-T	YNL015W	-0.86001	0.0146197	0.0476334	8.38	4.62	73.50	57.50
ORF-T	YNL252C	-0.859603	0.0039511	0.0158675	15.05	8.27	131.00	106.75
ORF-T	YAL028W	-0.85931	0.0824315	0.1865439	4.10	2.24	35.25	29.50
ORF-T	YER063W	-0.857778	0.004688	0.0183303	64.50	35.58	576.00	455.75
ORF-T	YOR084W	-0.857408	0.0023265	0.0099787	31.19	17.24	285.75	222.50
ORF-T	YPR040W	-0.856284	0.0190887	0.0592096	5.54	3.01	47.50	40.25
ORF-T	YNL307C	-0.855387	0.0146777	0.047776	35.11	19.37	302.00	252.50
AST	AS_YGL218W	-0.852613	0.0721701	0.1685027	4.99	2.73	39.75	35.00
SUT	SUT201	-0.852376	0.0190801	0.0592096	3.93	2.18	38.00	29.50
ORF-T	YFR031C-A	-0.851989	0.005231	0.0201796	1099.63	609.22	9819.75	7737.00
ORF-T	YPR145W	-0.851252	0.0044503	0.0175575	562.66	311.85	5197.25	4234.50

TABLE S1: Differential expression data for RRP6 RNA-Seq dataset Page 18

Class	Transcript name	RRP6 KO_vs_WT log2_fold _change	RRP6 KO_vs_WT p-value	RRP6 KO_vs_WT FDR	Ave Norm Reads in WT	Ave Norm Reads in RRP6	Average RAW read counts in WT	Average RAW read counts in RRP6
ORF-T	YMR152W	-0.850094	0.0122722	0.0412372	14.94	8.24	128.50	108.75
ORF-T	YJR074W	-0.849834	0.00274	0.0114837	21.79	12.09	196.75	154.50
sn/snoRNA	snR61	-0.849755	0.0135041	0.0446477	3076.56	1707.15	27911.75	21263.75
ORF-T	YMR101C	-0.848051	0.1423829	0.2784878	1.73	0.98	16.75	12.50
AST	AS_YLR062C	-0.848029	0.0063271	0.0237008	511.37	284.07	4478.75	3651.25
ORF-T	YNL030W	-0.847753	0.0023766	0.0101592	216.59	120.29	1946.25	1629.50
ORF-T	YGR026W	-0.846454	0.0301673	0.0861532	17.69	9.79	147.75	130.00
ORF-T	YLR218C	-0.845895	0.0479829	0.1240985	4.73	2.65	40.75	31.25
ORF-T	YOR198C	-0.845681	0.0023069	0.009899	83.71	46.54	728.50	603.75
ORF-T	YDL195W	-0.844724	0.0066882	0.024841	64.28	35.75	544.25	460.00
ORF-T	YPL131W	-0.843837	0.0045064	0.0177372	386.37	215.23	3476.00	2886.25
AST	AS_YKL051W	-0.843275	0.0224014	0.0672509	11.81	6.53	105.25	91.25
ORF-T	YMR076C	-0.842961	0.0046862	0.0183302	17.71	9.85	158.50	129.00
ORF-T	YJL158C	-0.842513	0.0030794	0.0127368	141.59	78.89	1239.25	1066.25
ORF-T	YGR025W	-0.842385	0.0438975	0.115941	11.75	6.51	98.00	86.75
AST	AS_YLR214W	-0.84104	0.1305517	0.2614665	1.20	0.69	12.00	9.00
ORF-T	YPL256C	-0.840779	0.0082456	0.0294125	22.19	12.35	196.75	163.00
ORF-T	YER104W	-0.840685	0.0044426	0.0175476	10.71	5.97	93.75	75.75
ORF-T	YGR066C	-0.840161	0.0878535	0.1951945	1.56	0.86	14.50	11.50
CUT	CUT787	-0.839879	0.0213734	0.0648597	4.38	2.43	37.50	31.25
AST	AS_YOR073W-A	-0.839357	0.0665764	0.1589762	1.24	0.69	11.25	9.00
ORF-T	YFR027W	-0.839348	0.0303434	0.0866071	5.39	3.02	47.75	38.25
ORF-T	YJL068C	-0.838174	0.0099562	0.0345012	27.51	15.37	249.00	202.00
ORF-T	YLR380W	-0.837689	0.0244491	0.0722794	6.43	3.56	54.50	46.50
ORF-T	YKR090W	-0.837376	0.0006159	0.0030295	90.08	50.46	853.75	654.25
ORF-T	YNL289W	-0.83673	0.0112567	0.0384788	35.53	19.91	322.25	253.75
AST	AS_YFL029C	-0.836385	0.0470472	0.1221483	2.32	1.28	21.75	17.75
ORF-T	YNL058C	-0.835564	0.0785182	0.1794193	7.91	4.38	68.00	63.00
AST	AS_YML094C-A	-0.835415	0.0239039	0.0708963	3.10	1.69	26.75	22.75

TABLE S1: Differential expression data for RRP6 RNA-Seq dataset Page 19

Class	Transcript name	RRP6 KO_vs_WT log2_fold _change	RRP6 KO_vs_WT p-value	RRP6 KO_vs_WT FDR	Ave Norm Reads in WT	Ave Norm Reads in RRP6	Average RAW read counts in WT	Average RAW read counts in RRP6
ORF-T	YOR182C	-0.835264	0.0078704	0.0283448	156.61	87.76	1351.75	1112.25
AST	AS_YLR092W	-0.835188	0.0203816	0.0624323	6.01	3.34	53.50	45.25
ORF-T	YLR245C	-0.834727	0.0190582	0.0591698	4.96	2.78	45.00	35.75
ORF-T	YJL225C	-0.834715	0.0396466	0.1073855	2.55	1.42	22.00	18.00
ORF-T	YDL083C	-0.834289	0.0044297	0.0175175	236.33	132.52	2037.00	1695.25
SUT	SUT802	-0.833357	0.0727763	0.1695257	6.94	3.87	55.75	48.50
sn/snoRNA	SNR34	-0.833187	0.0143367	0.0468789	1540.44	864.63	13249.50	10805.25
NUT	NUT1166	-0.833167	0.0027707	0.0116028	520.74	292.30	4657.00	3736.75
ORF-T	YJL130C	-0.833109	0.0027766	0.0116226	520.70	292.28	4656.50	3736.50
ORF-T	YBL081W	-0.831892	0.01586	0.0510136	5.23	2.91	46.25	38.25
ORF-T	YML131W	-0.831639	0.0456162	0.1194542	28.31	15.90	238.75	200.75
ORF-T	YBL072C	-0.831343	0.0019911	0.008726	441.44	248.08	4011.75	3243.75
ORF-T	YBR106W	-0.831162	0.0044691	0.0176179	605.04	340.12	5473.75	4216.25
ORF-T	YNL310C	-0.830967	0.0252944	0.0744191	4.83	2.69	41.00	34.50
ORF-T	YBR083W	-0.830905	0.0447468	0.1175377	7.71	4.31	65.75	56.00
AST	AS_YDR524W-C	-0.830715	0.0013728	0.0062556	87.86	49.37	769.00	642.75
ORF-T	YPR154W	-0.829151	0.0206635	0.0630662	11.28	6.31	95.75	82.75
ORF-T	YGL175C	-0.828931	0.0040054	0.0160469	9.92	5.56	91.25	74.75
ORF-T	YDR224C	-0.82841	0.0025983	0.0109722	60.86	34.20	535.75	460.75
ORF-T	YIR042C	-0.828235	0.0157281	0.0506391	6.63	3.69	57.50	49.25
ORF-T	YJL143W	-0.827779	0.0076107	0.0276365	28.46	16.01	250.50	208.25
ORF-T	YGL219C	-0.825799	0.0315188	0.0892547	12.29	6.88	101.00	91.00
ORF-T	YJL185C	-0.825796	0.0364101	0.1000707	4.01	2.24	37.00	30.25
ORF-T	YCL008C	-0.825102	0.045865	0.1199346	3.72	2.07	33.25	28.00
ORF-T	YNL213C	-0.824937	0.0409065	0.1099187	4.41	2.47	38.25	31.75
AST	AS_YGR038W	-0.823501	0.1010301	0.2170191	1.73	0.97	16.00	13.00
SUT	SUT694	-0.822931	0.0216065	0.0653902	6.04	3.39	53.75	45.75
SUT	SUT102	-0.822413	0.0558315	0.1395648	2.71	1.52	25.25	20.75
ORF-T	YJR091C	-0.82233	0.0453272	0.1188763	35.69	20.15	289.00	255.75

TABLE S1: Differential expression data for RRP6 RNA-Seq dataset Page 20

Class	Transcript name	RRP6 KO_vs_WT log2_fold _change	RRP6 KO_vs_WT p-value	RRP6 KO_vs_WT FDR	Ave Norm Reads in WT	Ave Norm Reads in RRP6	Average RAW read counts in WT	Average RAW read counts in RRP6
ORF-T	YGL178W	-0.822133	0.0358287	0.0989852	14.46	8.14	118.00	104.50
ORF-T	YKR077W	-0.821133	0.0728978	0.1697694	2.20	1.24	20.00	16.25
AST	AS_YAR050W	-0.821063	0.0242804	0.0718647	19.71	11.15	184.00	150.50
NUT	NUT0550	-0.820046	0.0156881	0.0505265	1545.37	875.34	13292.50	10947.00
ORF-T	YLR333C	-0.819168	0.0034659	0.014144	170.83	96.82	1516.50	1229.00
ORF-T	YDR436W	-0.818533	0.0487265	0.1255269	10.26	5.78	85.25	75.25
ORF-T	YPL055C	-0.818238	0.0042666	0.0169253	12.15	6.86	107.50	90.25
ORF-T	YOR236W	-0.817552	0.0410145	0.1101687	2.15	1.18	19.00	16.00
ORF-T	YBR016W	-0.817281	0.0196312	0.0605943	10.73	6.07	91.75	77.75
AST	AS_YBR113W	-0.816632	0.094535	0.2064293	1.39	0.77	12.50	10.50
ORF-T	YER014W	-0.816407	0.0097964	0.0340645	35.99	20.44	340.75	270.75
CUT	CUT828	-0.815861	0.0570303	0.1415966	2.97	1.66	28.50	23.75
AST	AS_YFL053W	-0.815236	0.0697268	0.1645893	2.82	1.57	23.75	21.00
ORF-T	YBR029C	-0.815208	0.0015652	0.007059	36.67	20.85	326.25	264.25
ORF-T	YCR052W	-0.81507	0.0024057	0.0102614	31.17	17.67	284.25	238.75
AST	AS_YKR102W	-0.81486	0.1285959	0.2587829	3.93	2.24	38.75	30.75
ORF-T	YNL167C	-0.814123	0.0405507	0.1091601	8.33	4.71	70.25	60.25
ORF-T	YHR068W	-0.813829	0.0016543	0.0074076	75.65	42.96	665.50	575.50
ORF-T	YOL081W	-0.813802	0.0040401	0.0161666	124.56	70.81	1060.50	920.75
AST	AS_YDL173W	-0.81376	0.058751	0.1448521	1.28	0.71	11.75	9.75
ORF-T	YOR066W	-0.813453	0.0277721	0.0804984	5.94	3.38	51.75	42.50
ORF-T	YML120C	-0.813442	0.1128386	0.2343697	24.52	13.94	197.50	168.75
ORF-T	YFR032C-B	-0.813006	0.0165886	0.0529459	256.51	146.01	2382.25	1900.50
ORF-T	YPL252C	-0.812897	0.0372976	0.1020648	2.96	1.66	26.75	22.25
ORF-T	YCR010C	-0.811462	0.1262785	0.2548896	9.56	5.47	93.00	70.00
ORF-T	YOL019W-A	-0.811311	0.0478246	0.1237675	10.93	6.19	91.50	81.50
ORF-T	YDR502C	-0.811015	0.0521395	0.1320378	18.02	10.27	156.75	127.00
ORF-T	YPL274W	-0.808529	0.0887299	0.1966268	75.14	42.91	665.00	524.00
ORF-T	YBR031W	-0.808244	0.0048695	0.018981	178.50	101.89	1580.00	1350.25

Class	Transcript name	RRP6 KO_vs_WT log2_fold _change	RRP6 KO_vs_WT p-value	RRP6 KO_vs_WT FDR	Ave Norm Reads in WT	Ave Norm Reads in RRP6	Average RAW read counts in WT	Average RAW read counts in RRP6
ORF-T	YKL001C	-0.807474	0.0237954	0.0706317	16.45	9.37	142.50	121.75
ORF-T	YHR180W	-0.807382	0.0570374	0.1415966	2.82	1.56	23.75	21.50
ORF-T	YOR312C	-0.806282	0.006965	0.0256709	338.87	193.76	2936.00	2476.75
ORF-T	YKL148C	-0.805819	0.0499075	0.1277328	62.89	35.95	530.00	458.00
ORF-T	YLR124W	-0.805263	0.1639962	0.3070437	1.02	0.56	10.00	8.50
ORF-T	YAL013W	-0.804732	0.0098576	0.0342181	16.66	9.48	149.50	130.00
ORF-T	YGR279C	-0.803861	0.0103768	0.0357865	250.74	143.58	2119.75	1870.75
ORF-T	YER052C	-0.803734	0.0456654	0.1195147	26.72	15.24	224.50	211.75
ORF-T	YLR061W	-0.803565	0.0086104	0.0304978	535.91	307.02	4700.25	3953.50
ORF-T	YPR133W-A	-0.802427	0.0691873	0.1635839	3.93	2.22	32.50	29.25
SUT	SUT600	-0.801542	0.1722632	0.3172704	2.49	1.42	24.00	20.25
ORF-T	YHL004W	-0.801375	0.0060573	0.02286	14.35	8.19	129.25	110.75
ORF-T	YCL037C	-0.801039	0.0131062	0.0436327	20.35	11.62	174.00	154.75
ORF-T	YKR011C	-0.799838	0.0345289	0.0959993	19.08	10.95	160.25	136.00
ORF-T	YPL032C	-0.798973	0.01739	0.0550409	36.97	21.19	305.75	275.25
ORF-T	YDL061C	-0.798657	0.0044449	0.0175501	133.68	76.83	1152.00	977.50
ORF-T	YLR448W	-0.798618	0.0047792	0.0186579	687.82	395.42	6176.50	5082.50
ORF-T	YGR110W	-0.798366	0.0339213	0.0945968	11.43	6.60	105.00	83.25
ORF-T	YOL060C	-0.797686	0.0131311	0.0437009	27.00	15.49	227.00	199.75
ORF-T	YGR153W	-0.797329	0.0206603	0.0630662	6.30	3.65	58.25	46.25
AST	AS_YCL007C	-0.797001	0.0238756	0.070833	13.86	7.95	120.75	103.25
ORF-T	YER178W	-0.796356	0.028358	0.0818907	125.44	72.18	1084.25	974.50
ORF-T	YHR047C	-0.79621	0.0695201	0.1641856	160.70	92.52	1469.25	1241.75
ORF-T	YNR006W	-0.794767	0.0543901	0.1366731	5.57	3.16	47.00	42.50
ORF-T	YPL241C	-0.794115	0.0123316	0.0414093	7.87	4.51	68.25	58.25
AST	AS_YGL152C	-0.793666	0.0084599	0.0300703	13.60	7.84	123.25	102.25
ORF-T	YJL121C	-0.792776	0.0010169	0.0047608	51.85	29.91	479.00	397.00
ORF-T	YDR064W	-0.791903	0.0052173	0.0201343	261.08	150.74	2247.50	1967.00
ORF-T	YJL172W	-0.791896	0.025064	0.0738374	69.24	39.94	588.00	526.25

TABLE S1: Differential expression data for RRP6 RNA-Seq dataset Page 22

Class	Transcript name	RRP6 KO_vs_WT log2_fold _change	RRP6 KO_vs_WT p-value	RRP6 KO_vs_WT FDR	Ave Norm Reads in WT	Ave Norm Reads in RRP6	Average RAW read counts in WT	Average RAW read counts in RRP6
AST	AS_YGL132W	-0.791852	0.0568827	0.1414562	2.16	1.22	20.25	17.25
ORF-T	YGL125W	-0.790689	0.0871132	0.1939183	7.29	4.19	60.25	53.50
ORF-T	YLL026W	-0.790504	0.1362126	0.2702194	151.51	87.57	1208.75	1081.25
ORF-T	YOR014W	-0.789976	0.007164	0.0262602	34.35	19.81	296.00	260.75
ORF-T	YPL246C	-0.789971	0.0046419	0.0181869	56.07	32.41	512.75	427.00
ORF-T	YER145C	-0.789824	0.0716481	0.1677501	332.81	192.52	3102.25	2482.00
ORF-T	YNL069C	-0.789254	0.0137802	0.0453671	883.28	511.08	7667.75	6529.50
ORF-T	YGR284C	-0.788155	0.0243596	0.072057	56.19	32.50	499.25	432.00
ORF-T	YER154W	-0.78799	0.0052149	0.0201343	14.55	8.44	130.25	107.00
NUT	NUT1269	-0.787949	0.0218638	0.06603	3120.68	1807.42	28288.25	22475.25
ORF-T	YCL005W-A	-0.787888	0.0221654	0.0667412	20.45	11.82	180.25	153.25
ORF-T	YBL007C	-0.787579	0.0038647	0.0155886	109.85	63.58	962.75	840.50
ORF-T	YIL069C	-0.787525	0.0060321	0.0227904	46.13	26.68	398.50	348.25
AST	AS_YKL037W	-0.787265	0.0400229	0.1080905	3.95	2.25	33.75	29.50
AST	AS_YDL009C	-0.78707	0.033087	0.092861	3.58	2.06	31.25	26.75
AST	AS_YIR017W-A	-0.787011	0.160644	0.302829	1.78	1.01	14.75	13.25
ORF-T	YJL177W	-0.786743	0.0076389	0.0277088	189.66	109.92	1658.50	1403.50
ORF-T	YOR348C	-0.786331	0.086001	0.1920784	24.67	14.33	222.25	176.50
ORF-T	YBL027W	-0.786163	0.0135939	0.044915	615.87	357.12	5431.00	4565.50
ORF-T	YNL239W	-0.785844	0.0129228	0.0431501	39.94	23.14	349.00	298.50
ORF-T	YFL017W-A	-0.78424	0.0433681	0.1148132	5.38	3.07	45.50	41.25
ORF-T	YBL013W	-0.783655	0.0215771	0.0653394	5.73	3.30	49.00	42.50
ORF-T	YNL168C	-0.7835	0.0098344	0.0341613	44.01	25.55	405.00	338.75
ORF-T	YDR018C	-0.782918	0.063309	0.1530862	2.52	1.48	23.00	18.75
ORF-T	YNR068C	-0.781477	0.1092235	0.2296038	2.85	1.63	24.50	22.00
ORF-T	YIL109C	-0.780894	0.0021141	0.0091656	82.23	47.79	721.50	635.00
ORF-T	YHR042W	-0.780707	0.0033899	0.0138676	48.04	27.93	414.75	358.25
AST	AS_YDR431W	-0.78062	0.0385336	0.1049446	4.36	2.53	38.50	32.50
AST	AS_YLR339C	-0.780292	0.008211	0.0292993	536.43	312.32	4819.25	4079.50

Class	Transcript name	RRP6 KO_vs_WT log2_fold _change	RRP6 KO_vs_WT p-value	RRP6 KO_vs_WT FDR	Ave Norm Reads in WT	Ave Norm Reads in RRP6	Average RAW read counts in WT	Average RAW read counts in RRP6
ORF-T	YDR242W	-0.780028	0.0109014	0.0374165	14.56	8.47	130.75	110.25
ORF-T	YER018C	-0.779843	0.0093761	0.0328325	8.62	5.00	76.50	65.75
AST	AS_YBR264C	-0.778459	0.014337	0.0468789	7.79	4.51	69.00	59.25
ORF-T	YER117W	-0.778262	0.0095956	0.03347	856.76	499.56	7619.75	6281.00
ORF-T	YCR051W	-0.778189	0.0015323	0.0069289	50.63	29.51	461.75	389.75
ORF-T	YKL198C	-0.777588	0.0094661	0.0330757	7.33	4.24	64.75	55.75
ORF-T	YHR116W	-0.777419	0.0127085	0.042519	9.11	5.29	83.25	70.75
ORF-T	YBR234C	-0.777378	0.0434124	0.1149003	31.64	18.41	269.00	244.75
ORF-T	YOL126C	-0.777269	0.0116213	0.0394709	79.90	46.62	688.50	588.75
ORF-T	YBR007C	-0.776824	0.0107374	0.0369417	8.45	4.92	77.00	64.75
AST	AS_YDR387C	-0.776493	0.1627996	0.3055969	1.20	0.70	12.00	9.75
ORF-T	YDL003W	-0.77616	0.0038905	0.01568	55.00	32.16	507.50	406.00
ORF-T	YGR189C	-0.775614	0.0174013	0.0550597	151.41	88.46	1361.25	1123.00
ORF-T	YOR224C	-0.775332	0.0031681	0.0130611	36.36	21.22	321.50	274.50
ORF-T	YDR076W	-0.775086	0.0485487	0.1252084	4.51	2.61	42.50	36.25
ORF-T	YOR083W	-0.77501	0.0043068	0.0170648	18.58	10.89	167.25	137.25
ORF-T	YGR027C	-0.774556	0.0114579	0.0390381	1350.04	789.18	12195.75	10283.50
ORF-T	YIL124W	-0.773532	0.0710695	0.1670153	64.14	37.50	536.25	475.00
ORF-T	YNR053C	-0.77333	0.0260407	0.0763133	2655.35	1553.57	23237.25	19360.75
ORF-T	YCL035C	-0.773237	0.0332394	0.0931258	20.82	12.13	177.00	161.50
ORF-T	YOR193W	-0.773197	0.0255278	0.0750284	3.81	2.22	34.00	29.00
ORF-T	YMR136W	-0.773135	0.0487382	0.1255269	8.98	5.25	76.50	65.50
ORF-T	YGL030W	-0.772552	0.0080671	0.0289084	422.22	247.14	3685.50	3166.25
AST	AS_YOL099C	-0.77228	0.0241337	0.0715154	3.82	2.21	33.00	28.75
ORF-T	YLR332W	-0.771722	0.0037896	0.0153041	24.37	14.22	215.25	189.00
ORF-T	YOR128C	-0.771044	0.0168957	0.0537634	376.73	220.83	3529.75	2679.50
ORF-T	YIL011W	-0.770725	0.1767792	0.3231712	1.38	0.78	11.25	10.50
ORF-T	YDL136W	-0.770191	0.0040663	0.0162586	99.66	58.42	889.00	756.75
ORF-T	YLR340W	-0.77013	0.0110787	0.0379091	1271.68	745.65	11501.25	9762.75

TABLE S1: Differential expression data for RRP6 RNA-Seq dataset Page 24

Class	Transcript name	RRP6 KO_vs_WT log2_fold_change	RRP6 KO_vs_WT p-value	RRP6 KO_vs_WT FDR	Ave Norm Reads in WT	Ave Norm Reads in RRP6	Average RAW read counts in WT	Average RAW read counts in RRP6
AST	AS_YDR209C	-0.76969	0.0180655	0.0567502	12.76	7.41	111.25	102.50
ORF-T	YER174C	-0.769139	0.0026709	0.0112361	60.66	35.63	551.00	446.00
ORF-T	YJR146W	-0.768639	0.0515033	0.1308539	3.34	1.96	30.00	25.00
ORF-T	YBR109C	-0.768513	0.0220857	0.0665608	15.43	8.99	131.25	121.50
ORF-T	YOL135C	-0.767601	0.0842154	0.189433	2.23	1.31	19.75	16.50
ORF-T	YMR010W	-0.766874	0.001875	0.0082781	92.42	54.27	851.50	735.75
ORF-T	YER030W	-0.766859	0.0109168	0.0374312	26.79	15.73	245.50	207.00
AST	AS_YCR081C-A	-0.766826	0.067198	0.1600595	3.00	1.73	25.75	23.00
ORF-T	YCR034W	-0.766809	0.0069469	0.0256228	46.58	27.39	420.00	347.50
ORF-T	YPL141C	-0.766291	0.0932826	0.2041809	14.31	8.37	125.00	116.00
ORF-T	YPL124W	-0.766049	0.0122446	0.0411718	9.12	5.38	85.75	69.50
sn/snoRNA	SNR71	-0.765951	0.0154011	0.0497612	1744.22	1025.71	15222.50	12997.75
ORF-T	YML126C	-0.765891	0.0060191	0.0227624	130.36	76.60	1178.00	1042.50
CUT	CUT404	-0.765173	0.0377738	0.103144	4.27	2.48	38.50	34.00
ORF-T	YPL262W	-0.764953	0.0117311	0.0397501	134.12	78.87	1199.75	1060.00
ORF-T	YJL060W	-0.764906	0.0986018	0.2131862	6.24	3.66	54.25	46.25
ORF-T	YPR187W	-0.764747	0.0029251	0.0121785	37.58	22.10	337.25	286.75
ORF-T	YOR096W	-0.764428	0.0204919	0.0626942	2803.44	1650.35	24908.25	20915.00
ORF-T	YJL105W	-0.764386	0.0561805	0.1403329	2.24	1.32	20.25	17.00
ORF-T	YOL110W	-0.764317	0.0039798	0.0159682	26.65	15.73	246.00	200.50
ORF-T	YNL250W	-0.764128	0.0033345	0.0136689	34.77	20.43	311.00	270.25
ORF-T	YOR358W	-0.764057	0.0130811	0.0435633	7.84	4.62	70.50	59.25
ORF-T	YDL232W	-0.763919	0.0240893	0.071425	6.52	3.84	57.00	48.50
ORF-T	YGR282C	-0.763437	0.0056379	0.0215349	230.50	135.78	2013.00	1721.00
ORF-T	YJR021C	-0.763343	0.0165915	0.0529459	7.72	4.53	67.00	58.00
AST	AS_YFL030W	-0.763204	0.0677995	0.1610956	3.02	1.79	28.75	23.25
ORF-T	YOR372C	-0.763174	0.0296178	0.0849243	12.24	7.16	104.25	95.00
ORF-T	YHR215W	-0.762465	0.0782873	0.1791353	14.87	8.79	133.25	105.75
AST	AS_YGL192W	-0.762219	0.0413726	0.1106634	10.93	6.39	88.50	83.00

Class	Transcript name	RRP6 KO_vs_WT log2_fold _change	RRP6 KO_vs_WT p-value	RRP6 KO_vs_WT FDR	Ave Norm Reads in WT	Ave Norm Reads in RRP6	Average RAW read counts in WT	Average RAW read counts in RRP6
ORF-T	YMR276W	-0.762011	0.0022555	0.0097073	47.94	28.23	424.25	368.75
ORF-T	YLR388W	-0.761878	0.0042301	0.0168072	50.89	29.97	441.00	387.00
ORF-T	YGL081W	-0.76155	0.0175846	0.0555524	6.99	4.13	66.50	55.50
ORF-T	YBR086C	-0.761517	0.0319498	0.0903378	92.47	54.48	770.75	727.50
ORF-T	YLR019W	-0.761437	0.0014399	0.0065346	89.15	52.55	825.25	710.75
AST	AS_YKL030W	-0.760985	0.1041988	0.222285	28.42	16.81	273.00	208.00
ORF-T	YPL115C	-0.760627	0.0094283	0.0329779	33.48	19.71	300.25	267.25
ORF-T	YHL021C	-0.760553	0.1248563	0.2528216	47.42	27.97	384.00	355.50
NUT	NUT1036	-0.760399	0.079427	0.1811267	111.04	65.50	883.00	862.50
ORF-T	YDL014W	-0.759716	0.0032741	0.0134539	44.01	25.97	391.50	338.50
ORF-T	YLR325C	-0.759297	0.0085087	0.0302117	755.76	446.48	6791.00	5784.00
ORF-T	YHR183W	-0.758897	0.0293476	0.0843379	525.77	310.71	4706.75	3917.75
ORF-T	YPL063W	-0.758576	0.0034492	0.0140912	46.95	27.76	438.25	362.50
SRT	SRT82	-0.758155	0.1130635	0.2347265	3.95	2.33	39.00	32.50
ORF-T	YFR019W	-0.757951	0.0201757	0.0619146	26.85	15.83	241.50	215.75
ORF-T	YNL074C	-0.757872	0.061806	0.1503622	5.98	3.51	49.75	45.00
ORF-T	YHR038W	-0.757585	0.0637343	0.153861	2.83	1.65	23.50	21.00
ORF-T	YDR210W	-0.757551	0.0194843	0.0602145	12.82	7.51	111.75	103.75
ORF-T	RPL15B	-0.757317	0.0062375	0.0234327	20.13	11.88	175.50	153.25
ORF-T	YNL183C	-0.756607	0.0288898	0.0832119	14.54	8.56	124.25	113.25
ORF-T	YEL065W	-0.75591	0.090161	0.1986853	205.91	121.96	1906.75	1541.25
AST	AS_YGL102C	-0.755809	0.0119782	0.0404245	3621.97	2145.00	32106.00	26771.00
ORF-T	YOR367W	-0.755602	0.0303816	0.0866671	6.64	3.91	58.50	51.50
ORF-T	YCL048W	-0.755503	0.1163746	0.2396898	1.81	1.08	16.25	13.50
ORF-T	YDL075W	-0.755111	0.0065852	0.0245218	105.01	62.18	918.25	806.25
AST	AS_YDR355C	-0.755077	0.0436938	0.1154935	3.61	2.12	32.25	27.75
ORF-T	YML028W	-0.754571	0.0970361	0.2104328	542.34	321.45	4574.75	4000.25
ORF-T	YFR032C-A	-0.754373	0.0444649	0.1169799	559.69	331.77	4986.00	4315.00
ORF-T	YML021C	-0.753882	0.020107	0.0617226	7.81	4.61	68.25	59.25

TABLE S1: Differential expression data for RRP6 RNA-Seq dataset Page 26

Class	Transcript name	RRP6 KO_vs_WT log2_fold _change	RRP6 KO_vs_WT p-value	RRP6 KO_vs_WT FDR	Ave Norm Reads in WT	Ave Norm Reads in RRP6	Average RAW read counts in WT	Average RAW read counts in RRP6
ORF-T	YLR264W	-0.753698	0.0357407	0.0988235	219.63	130.22	1887.75	1710.50
ORF-T	YML081C-A	-0.752002	0.0411106	0.1103431	9.10	5.38	76.75	68.25
AST	AS_YER023C-A	-0.751883	0.018221	0.0570801	23.37	13.87	214.75	181.50
ORF-T	YDL130W	-0.751444	0.0019945	0.0087296	69.33	41.16	612.50	531.50
ORF-T	YPL057C	-0.751024	0.0092752	0.0325553	61.66	36.59	560.50	497.50
SUT	SUT097	-0.750943	0.1155149	0.2386992	3.93	2.33	38.75	32.50
ORF-T	YLR109W	-0.750275	0.0471857	0.1224764	230.61	137.08	1986.25	1739.25
ORF-T	YLR203C	-0.749471	0.0062393	0.0234327	34.52	20.48	307.25	271.50
AST	AS_YJL007C	-0.749052	0.0918654	0.2017792	6.32	3.75	53.00	46.75
ORF-T	YER087C-B	-0.747703	0.0143575	0.0469308	12.27	7.27	107.00	95.50
ORF-T	YAL040C	-0.747199	0.0667554	0.1592527	13.78	8.18	115.00	106.00
ORF-T	YNL054W	-0.746558	0.0285552	0.0823659	15.00	8.95	129.50	109.75
ORF-T	YDL135C	-0.746416	0.00651	0.0242956	26.70	15.91	244.75	208.25
ORF-T	YEL060C	-0.74625	0.0180362	0.0566771	90.65	54.02	786.25	693.25
ORF-T	YBL002W	-0.745835	0.0122217	0.0411314	37.63	22.37	327.00	302.25
SRT	SRT86	-0.745823	0.2108777	0.3638136	1.14	0.66	10.75	9.75
ORF-T	YHR179W	-0.745717	0.0153209	0.0495657	388.77	231.81	3540.50	3146.50
ORF-T	YFR006W	-0.745433	0.0062295	0.0234134	205.93	122.85	1893.25	1585.50
AST	AS_YGL123C-A	-0.745424	0.0078994	0.0283985	1247.30	744.02	11547.75	9572.25
ORF-T	YAL046C	-0.745353	0.0042339	0.0168155	18.27	10.89	168.25	143.25
ORF-T	YBL047C	-0.744508	0.0211794	0.0644214	113.00	67.42	950.00	847.50
ORF-T	YLR064W	-0.744406	0.0617195	0.1501882	5.01	2.94	42.50	39.25
ORF-T	YHR153C	-0.744163	0.0715477	0.1676466	2.00	1.20	18.50	15.50
NUT	NUT0349	-0.74393	0.0183549	0.0573926	1749.25	1044.49	15268.00	13233.25
ORF-T	YGR207C	-0.743708	0.0040692	0.0162639	26.07	15.55	233.00	202.50
ORF-T	YGR010W	-0.743473	0.0025003	0.0105935	34.36	20.52	314.00	268.50
AST	AS_YDR512C	-0.743332	0.1359177	0.2697952	7.48	4.44	62.75	57.50
ORF-T	YGL153W	-0.743069	0.01033	0.0356496	18.05	10.77	163.25	141.25
ORF-T	YIL154C	-0.742926	0.0209219	0.06372	9.48	5.66	88.00	75.50

TABLE S1: Differential expression data for RRP6 RNA-Seq dataset Page 27

Class	Transcript name	RRP6 KO_vs_WT log2_fold _change	RRP6 KO_vs_WT p-value	RRP6 KO_vs_WT FDR	Ave Norm Reads in WT	Ave Norm Reads in RRP6	Average RAW read counts in WT	Average RAW read counts in RRP6
ORF-T	YGR214W	-0.742393	0.010866	0.0373205	307.17	183.57	2701.50	2397.25
ORF-T	YOR293W	-0.74227	0.014457	0.0472099	366.12	218.88	3240.75	2745.75
ORF-T	YGR085C	-0.74147	0.0085608	0.0303543	110.37	65.96	959.25	867.00
ORF-T	YDR384C	-0.741248	0.016338	0.052269	40.81	24.41	376.75	326.50
ORF-T	YOR113W	-0.74089	0.0324325	0.0913802	14.55	8.66	124.00	113.75
ORF-T	YKL193C	-0.740247	0.0423782	0.1127849	12.24	7.27	102.50	97.00
ORF-T	YMR078C	-0.739759	0.0057771	0.0220002	22.57	13.48	206.75	181.75
ORF-T	YKL029C	-0.739168	0.1070168	0.2266184	106.13	63.61	993.50	777.00
ORF-T	YIR036C	-0.737692	0.0176306	0.0556803	49.84	29.90	455.00	380.25
ORF-T	YGL117W	-0.737599	0.0063938	0.023915	20.75	12.39	186.25	167.25
ORF-T	YCR024C	-0.736657	0.0263534	0.077006	7.24	4.27	63.50	59.50
ORF-T	YLR063W	-0.7363	0.1222379	0.2487731	2.50	1.46	21.75	20.25
ORF-T	YIL073C	-0.736086	0.0560004	0.1399177	5.87	3.56	58.75	46.75
ORF-T	YMR244W	-0.735375	0.1956664	0.3456727	3.10	1.89	33.00	24.75
ORF-T	YOR132W	-0.734883	0.0051881	0.0200447	45.39	27.24	419.00	365.00
ORF-T	YEL066W	-0.734542	0.0046495	0.0182079	44.16	26.52	399.50	348.25
ORF-T	YJR121W	-0.733752	0.0672047	0.1600595	345.40	207.67	2882.75	2643.50
ORF-T	YIL130W	-0.73339	0.0136955	0.0451767	16.23	9.70	138.50	127.00
AST	AS_YGL231C	-0.73277	0.1247343	0.2528177	1.53	0.91	14.50	12.50
ORF-T	YDR308C	-0.732284	0.033876	0.0945228	16.27	9.78	149.25	128.75
ORF-T	YBR071W	-0.732116	0.0197092	0.060779	12.48	7.50	113.50	98.50
ORF-T	YPR172W	-0.731728	0.1122549	0.2336972	5.61	3.34	46.75	44.50
ORF-T	YFL034C-B	-0.731309	0.0138531	0.0455476	8.37	4.99	73.25	66.25
ORF-T	YLR029C	-0.731306	0.0227316	0.0680827	2181.72	1314.13	19249.50	17638.00
ORF-T	YCR046C	-0.730111	0.0092705	0.03255	18.29	10.99	163.75	144.50
ORF-T	YJL092W	-0.730046	0.0059019	0.022357	42.51	25.59	385.25	343.50
ORF-T	YBR111C	-0.729749	0.0591756	0.1454366	21.57	12.97	180.75	165.25
ORF-T	YOR234C	-0.728371	0.0232336	0.0692543	318.64	192.33	2824.50	2417.75
AST	AS_YPL142C	-0.727654	0.0117155	0.0397106	196.35	118.55	1715.00	1521.75

Class	Transcript name	RRP6 KO_vs_WT log2_fold _change	RRP6 KO_vs_WT p-value	RRP6 KO_vs_WT FDR	Ave Norm Reads in WT	Ave Norm Reads in RRP6	Average RAW read counts in WT	Average RAW read counts in RRP6
ORF-T	YDR450W	-0.727014	0.0146413	0.0476882	497.55	300.58	4448.00	3870.25
ORF-T	YDL193W	-0.726895	0.0053552	0.0205957	30.21	18.25	268.25	232.75
ORF-T	YLL013C	-0.726411	0.0233606	0.0695711	24.23	14.61	204.25	187.50
NUT	NUT0093	-0.726055	0.1081058	0.2278718	5.89	3.57	55.25	47.00
ORF-T	YDR086C	-0.725798	0.0113594	0.0388035	29.23	17.65	257.50	227.75
AST	AS_YDR223W	-0.725648	0.1472136	0.2843874	1.33	0.79	11.75	10.50
ORF-T	YGL197W	-0.725482	0.0193826	0.0599922	27.55	16.62	234.50	215.75
ORF-T	YJR123W	-0.725396	0.0133131	0.0441463	576.94	348.92	5056.00	4509.75
SUT	SUT138	-0.725244	0.0167938	0.0535236	19.96	12.10	187.75	156.25
ORF-T	YKR080W	-0.725147	0.0206991	0.0631557	219.51	132.84	2026.00	1648.25
ORF-T	YOL143C	-0.725101	0.0051878	0.0200447	34.68	20.92	302.25	275.00
AST	AS_YLR236C	-0.725047	0.0327397	0.092194	4.62	2.78	41.25	36.25
ORF-T	YPL143W	-0.724666	0.0117044	0.0396997	206.04	124.66	1800.00	1598.25
ORF-T	YKR072C	-0.724636	0.0041835	0.0166286	42.66	25.81	394.00	342.50
ORF-T	YHR142W	-0.724171	0.005055	0.0196282	101.83	61.68	960.50	803.75
ORF-T	YMR142C	-0.724028	0.0118811	0.0401844	874.36	529.33	8098.25	6993.25
ORF-T	YCL030C	-0.723628	0.0187806	0.0585059	336.36	203.67	3067.00	2687.50
ORF-T	YMR055C	-0.723381	0.062953	0.1525271	3.27	1.95	29.00	26.25
ORF-T	YBR098W	-0.722824	0.0233375	0.0695229	6.27	3.79	57.75	50.00
ORF-T	YMR215W	-0.722721	0.008297	0.0295644	74.20	44.89	648.75	602.75
ORF-T	YOR304C-A	-0.722522	0.0641263	0.1544713	2.60	1.57	22.75	20.00
ORF-T	YGL103W	-0.721152	0.0154361	0.0498583	3817.20	2315.58	33899.00	28976.00
ORF-T	YDR488C	-0.720898	0.0044134	0.0174665	31.92	19.36	295.00	255.50
ORF-T	YPR182W	-0.720835	0.0291636	0.0839287	7.14	4.32	63.00	55.50
SUT	SUT688	-0.720799	0.0842624	0.1894964	2.80	1.68	24.25	21.75
ORF-T	YER055C	-0.719273	0.0097332	0.0338966	218.55	132.72	2005.25	1781.00
ORF-T	YIL018W	-0.719007	0.0215832	0.0653394	2747.71	1669.27	24449.50	21419.00
ORF-T	YJL062W	-0.718185	0.0169305	0.053857	17.52	10.59	153.75	142.25
ORF-T	YJL178C	-0.718051	0.0062907	0.0235906	24.21	14.67	214.50	194.25

Class	Transcript name	RRP6 KO_vs_WT log2_fold _change	RRP6 KO_vs_WT p-value	RRP6 KO_vs_WT FDR	Ave Norm Reads in WT	Ave Norm Reads in RRP6	Average RAW read counts in WT	Average RAW read counts in RRP6
ORF-T	YHR203C	-0.717286	0.009648	0.0336297	2312.34	1406.48	20899.75	17806.75
ORF-T	YJL003W	-0.71711	0.0158462	0.0509869	9.04	5.48	79.75	71.00
ORF-T	YMR300C	-0.717021	0.0392745	0.106578	483.66	294.30	4597.50	3617.75
ORF-T	YCR075W-A	-0.716894	0.0516151	0.1310394	3.77	2.25	32.25	30.00
SUT	SUT353	-0.716765	0.3172576	0.4826834	0.91	0.51	7.25	7.75
SUT	SUT770	-0.716634	0.0178495	0.0562483	6.98	4.22	62.25	55.75
ORF-T	YCR028C-A	-0.715921	0.048612	0.1253075	7.96	4.80	67.50	63.00
ORF-T	YLR219W	-0.715387	0.0177215	0.0559148	32.34	19.65	271.50	252.00
NUT	NUT0098	-0.715317	0.033246	0.0931258	7.60	4.56	66.25	62.50
SUT	SUT389	-0.715243	0.1159505	0.2391094	1.09	0.66	9.75	8.50
ORF-T	YKL167C	-0.715116	0.0469529	0.1219662	3.38	2.03	29.75	26.75
ORF-T	YBR185C	-0.714983	0.0358977	0.0990946	6.54	3.95	56.50	51.75
ORF-T	YHR083W	-0.714867	0.0053457	0.020567	36.39	22.16	325.25	284.75
ORF-T	YHR099W	-0.714763	0.0082645	0.0294692	179.60	109.36	1594.25	1481.25
ORF-T	YOL071W	-0.714577	0.0412127	0.1104408	8.01	4.84	68.50	63.75
ORF-T	YMR058W	-0.714136	0.0516286	0.1310403	637.26	388.46	5839.50	5040.25
ORF-T	YKR094C	-0.713796	0.0177864	0.056102	245.68	149.75	2167.75	1985.25
sn/snoRNA	SNR31	-0.713728	0.037316	0.1020875	576.61	351.59	5056.50	4374.50
AST	AS_YLR264C-A	-0.713528	0.0427072	0.1133842	68.60	41.81	615.50	556.50
ORF-T	YJL115W	-0.713445	0.0074383	0.0271079	24.07	14.73	224.50	186.25
ORF-T	YLR370C	-0.713402	0.0572847	0.1420357	14.90	9.06	127.25	116.75
AST	AS_YBR089W	-0.713329	0.022952	0.0686179	35.89	21.91	318.00	271.25
ORF-T	YML133C	-0.713228	0.1262408	0.2548896	2.70	1.68	24.50	19.75
ORF-T	YNL016W	-0.713202	0.0173412	0.0549199	62.05	37.81	537.75	491.25
ORF-T	YKL066W	-0.713194	0.0371696	0.1018527	75.91	46.29	650.50	579.25
ORF-T	YOL107W	-0.712766	0.0968992	0.2102094	1.57	0.96	14.50	12.50
ORF-T	YLR293C	-0.712607	0.0200465	0.0615931	117.12	71.42	1013.00	932.25
ORF-T	YDR099W	-0.712024	0.03206	0.0905677	58.27	35.52	489.00	462.75
ORF-T	YER059W	-0.711844	0.1025068	0.2196513	6.51	3.98	57.00	48.75

TABLE S1: Differential expression data for RRP6 RNA-Seq dataset Page 30

Class	Transcript name	RRP6 KO_vs_WT log2_fold _change	RRP6 KO_vs_WT p-value	RRP6 KO_vs_WT FDR	Ave Norm Reads in WT	Ave Norm Reads in RRP6	Average RAW read counts in WT	Average RAW read counts in RRP6
ORF-T	YBR088C	-0.711763	0.0206509	0.0630661	49.06	29.98	436.25	371.25
ORF-T	YOR063W	-0.711676	0.0116834	0.0396417	4609.12	2814.41	42154.25	35445.25
AST	AS_YMR307C-A	-0.711241	0.0119425	0.040331	95.51	58.27	845.50	783.25
ORF-T	YOR011W	-0.710637	0.0085504	0.030328	38.36	23.38	348.75	316.50
ORF-T	YDR524C-B	-0.710311	0.0078845	0.0283798	518.31	316.76	4571.25	4109.00
ORF-T	YLL023C	-0.709524	0.0419025	0.111814	10.33	6.29	86.25	78.75
ORF-T	YHR110W	-0.709299	0.0081185	0.0290309	28.18	17.24	252.75	218.25
ORF-T	YFR003C	-0.709226	0.0757976	0.1752158	8.26	5.04	70.00	62.75
ORF-T	YGL147C	-0.708731	0.0198308	0.061042	1830.97	1120.28	16296.50	14363.00
ORF-T	YDR253C	-0.708641	0.1998943	0.3509242	1.16	0.70	10.25	9.00
AST	AS_YJL200C	-0.708199	0.1651359	0.3084331	0.95	0.57	9.00	8.00
ORF-T	YMR298W	-0.708104	0.0251524	0.0740544	15.31	9.31	132.25	124.50
ORF-T	YBR187W	-0.708009	0.0060202	0.0227624	172.92	105.88	1609.50	1377.50
AST	AS_YKR105C	-0.707843	0.0930696	0.2037591	2.27	1.36	20.75	19.00
ORF-T	YHR029C	-0.707053	0.0185538	0.0579424	30.19	18.42	272.75	256.75
ORF-T	YCR067C	-0.70609	0.0748395	0.1733306	28.91	17.69	238.50	224.75
ORF-T	YLR335W	-0.705588	0.0170546	0.0541437	24.76	15.15	218.50	199.75
ORF-T	YLR413W	-0.705104	0.0347098	0.0964225	33.11	20.29	282.75	258.00
ORF-T	YLR373C	-0.704833	0.0339195	0.0945968	10.03	6.11	86.00	80.00
AST	AS_YGL109W	-0.704488	0.057021	0.1415966	3.49	2.16	32.25	27.25
ORF-T	YLR395C	-0.704351	0.0579101	0.143199	23.41	14.32	196.50	186.25
ORF-T	YGL258W-A	-0.704208	0.1946089	0.3445172	1.15	0.70	10.25	9.00
ORF-T	YNL233W	-0.704184	0.0189697	0.0589492	17.93	10.94	157.00	147.00
NUT	NUT1078	-0.703684	0.1662	0.3097321	1.22	0.72	10.50	9.75
AST	AS_YEL053W-A	-0.703055	0.0055225	0.0211424	243.86	149.81	2269.75	1965.25
ORF-T	YLR402W	-0.703009	0.1667924	0.3103391	1.20	0.72	10.25	9.25
ORF-T	YLR423C	-0.702239	0.0213554	0.0648246	11.77	7.23	102.75	91.25
ORF-T	YNR069C	-0.701766	0.1096018	0.2299677	3.99	2.41	34.25	32.50
ORF-T	YNL162W	-0.701371	0.0140184	0.0460311	309.15	190.10	2725.50	2451.75

Class	Transcript name	RRP6 KO_vs_WT log2_fold _change	RRP6 KO_vs_WT p-value	RRP6 KO_vs_WT FDR	Ave Norm Reads in WT	Ave Norm Reads in RRP6	Average RAW read counts in WT	Average RAW read counts in RRP6
ORF-T	YEL061C	-0.701316	0.002579	0.0108998	64.75	39.83	596.25	519.25
AST	AS_YDL096C	-0.700925	0.0036785	0.014891	35.01	21.51	313.25	282.50
ORF-T	YPL237W	-0.700807	0.005733	0.0218568	170.91	105.12	1493.75	1353.00
ORF-T	YCR075C	-0.700638	0.0164821	0.0527134	44.15	27.16	400.25	352.50
ORF-T	YCL004W	-0.70036	0.0537808	0.1354798	7.19	4.36	62.50	59.75
ORF-T	YPR135W	-0.699732	0.0040901	0.0163214	45.79	28.23	429.50	363.25
ORF-T	YBR024W	-0.699481	0.0408719	0.1099073	16.21	10.00	149.00	127.75
ORF-T	YDR359C	-0.699468	0.0058515	0.0221995	22.12	13.62	202.75	178.25
ORF-T	YGL123W	-0.699459	0.015224	0.0493154	3690.23	2272.47	33895.00	29257.50
ORF-T	YIR014W	-0.699418	0.0652844	0.156521	7.07	4.35	64.00	56.50
ORF-T	YFL037W	-0.698172	0.0082689	0.0294745	164.35	101.27	1482.00	1327.75
ORF-T	YIL148W	-0.69744	0.0256972	0.0754465	669.95	413.10	5909.50	5382.25
ORF-T	YML079W	-0.697412	0.0257025	0.0754465	21.64	13.32	192.25	173.75
ORF-T	YPL134C	-0.697382	0.0184024	0.0575231	40.66	25.08	375.25	324.00
ORF-T	YNL121C	-0.697066	0.043734	0.1155696	170.94	105.42	1561.50	1406.25
ORF-T	YML114C	-0.696162	0.0821404	0.1860102	5.80	3.53	51.75	49.25
ORF-T	YER146W	-0.69581	0.0092852	0.032579	52.68	32.55	476.50	411.00
ORF-T	YLL062C	-0.695575	0.1900513	0.3391995	15.79	9.75	138.75	121.25
ORF-T	YER109C	-0.695389	0.0123058	0.0413362	28.02	17.26	255.75	233.75
ORF-T	YDR133C	-0.695081	0.0309668	0.0879879	116.09	71.67	996.25	914.50
ORF-T	YER061C	-0.695022	0.0080641	0.028908	15.53	9.62	141.75	122.50
ORF-T	YAR019C	-0.694053	0.0725749	0.1691736	8.85	5.46	76.25	69.50
ORF-T	YJR015W	-0.693822	0.0114523	0.0390381	217.48	134.47	2006.25	1730.75
AST	AS_YDR360W	-0.69375	0.0436845	0.1154935	3.13	1.93	29.25	25.75
sn/snoRNA	SNR80	-0.693627	0.0740974	0.1721258	1145.04	707.97	10083.75	8934.25
AST	AS_YAL061W	-0.693566	0.0956951	0.208376	2.25	1.37	19.50	17.75
ORF-T	YHR136C	-0.693121	0.1473615	0.2846185	7.95	4.90	68.75	64.75
ORF-T	YDL081C	-0.692989	0.0138284	0.0455004	490.86	303.59	4348.50	4014.50
SUT	SUT227	-0.692955	0.1330689	0.2653176	2.29	1.38	21.25	20.00

Class	Transcript name	RRP6 KO_vs_WT log2_fold _change	RRP6 KO_vs_WT p-value	RRP6 KO_vs_WT FDR	Ave Norm Reads in WT	Ave Norm Reads in RRP6	Average RAW read counts in WT	Average RAW read counts in RRP6
ORF-T	YOR208W	-0.692934	0.0180763	0.0567502	22.84	14.13	202.50	180.50
ORF-T	YPL090C	-0.692649	0.0128554	0.0429534	718.35	444.48	6496.00	5564.25
ORF-T	YFL018C	-0.6926	0.034833	0.0967114	144.40	89.31	1248.25	1148.25
AST	AS_YOR225W	-0.692509	0.0344819	0.0959216	6.75	4.17	60.25	54.50
ORF-T	YLR167W	-0.692286	0.017123	0.054322	4523.90	2799.73	40770.25	35442.50
ORF-T	YMR030W	-0.691947	0.0651694	0.1563689	3.73	2.28	33.00	30.50
NUT	NUT0119	-0.691402	0.3795508	0.5408853	17.06	10.59	157.75	119.00
ORF-T	YIL152W	-0.691336	0.0972781	0.2108494	2.77	1.70	24.50	22.25
ORF-T	YMR072W	-0.690969	0.0644569	0.1550902	27.29	16.87	234.25	221.50
ORF-T	YMR194W	-0.690894	0.0145699	0.0475016	27.29	16.87	240.25	220.00
ORF-T	YLR150W	-0.690155	0.0122244	0.0411314	1295.42	802.92	12203.75	10327.25
ORF-T	YDL144C	-0.690077	0.0058455	0.0221935	41.41	25.70	387.00	333.50
ORF-T	YCR002C	-0.689941	0.0057115	0.0217914	58.32	36.09	521.00	481.50
ORF-T	YBL035C	-0.689834	0.0221237	0.0666353	21.98	13.63	197.50	174.25
ORF-T	YGR011W	-0.68908	0.0149642	0.0485986	10.40	6.47	97.25	84.50
ORF-T	YJR086W	-0.689017	0.0514833	0.1308362	7.49	4.63	65.50	59.75
ORF-T	YBL003C	-0.687764	0.0223462	0.0671578	356.56	221.31	3120.50	2920.75
ORF-T	YLR420W	-0.687246	0.0059682	0.0226	69.00	42.90	630.50	542.25
AST	AS_YLR235C	-0.686779	0.0388321	0.105605	4.89	3.02	43.75	39.75
ORF-T	YBR156C	-0.686527	0.1277375	0.257518	2.98	1.79	25.00	25.00
ORF-T	YJL173C	-0.686426	0.0260932	0.0764452	10.58	6.54	95.00	87.50
ORF-T	YLL061W	-0.685783	0.1693171	0.3135134	85.56	53.19	771.25	655.75
ORF-T	YML024W	-0.685614	0.028975	0.0834333	1087.60	676.22	9703.25	8466.25
CUT	CUT484	-0.685529	0.0349672	0.0969772	8.61	5.32	77.75	72.00
ORF-T	YNR067C	-0.685371	0.064599	0.1553338	86.90	54.02	797.50	721.75
AST	AS_YHR182C-A	-0.685268	0.044667	0.1174197	146.14	90.89	1302.50	1141.75
NUT	NUT1446	-0.685172	0.044015	0.1161599	576.95	358.83	5059.50	4469.25
AST	AS_YDR269C	-0.684922	0.0541605	0.1361982	4.46	2.73	40.00	37.50
ORF-T	YJL074C	-0.684811	0.0053001	0.0204072	48.69	30.32	440.25	382.00

TABLE S1: Differential expression data for RRP6 RNA-Seq dataset Page 33

Class	Transcript name	RRP6 KO_vs_WT log2_fold _change	RRP6 KO_vs_WT p-value	RRP6 KO_vs_WT FDR	Ave Norm Reads in WT	Ave Norm Reads in RRP6	Average RAW read counts in WT	Average RAW read counts in RRP6
ORF-T	YER042W	-0.68429	0.2258856	0.3826081	20.38	12.68	172.50	154.75
ORF-T	YCR018C-A	-0.68414	0.104421	0.2226176	2.46	1.55	22.50	19.50
ORF-T	YBR145W	-0.683573	0.0133365	0.0441806	32.23	20.05	279.50	255.00
ORF-T	YGL108C	-0.683513	0.0424652	0.1129567	4.89	3.07	45.25	39.00
ORF-T	YER152C	-0.683485	0.0078626	0.0283294	67.32	41.95	627.25	539.75
ORF-T	YHR050W-A	-0.683435	0.1048403	0.2231341	7.33	4.56	65.50	57.25
ORF-T	YDR071C	-0.682712	0.013474	0.0445775	53.58	33.33	469.00	434.50
ORF-T	YOR374W	-0.682664	0.0664373	0.1587569	198.45	123.69	1799.50	1478.00
ORF-T	YDR074W	-0.682529	0.1366442	0.2707644	185.69	115.68	1497.75	1425.25
ORF-T	YLR441C	-0.682298	0.01404	0.0460872	1927.80	1201.34	17384.25	15538.75
ORF-T	YPR052C	-0.681808	0.0275742	0.0800628	28.87	17.94	250.50	239.00
ORF-T	YOL051W	-0.680437	0.097291	0.2108494	13.80	8.56	111.25	111.00
ORF-T	YML034W	-0.680106	0.0316127	0.0894955	36.16	22.51	305.25	296.00
ORF-T	YEL043W	-0.679427	0.0364672	0.1002002	21.59	13.41	183.50	181.50
ORF-T	YLR377C	-0.67862	0.1470342	0.2842891	37.03	23.16	363.00	307.00
AST	AS_YER014C-A	-0.678531	0.0511055	0.1301056	11.00	6.87	102.00	90.75
ORF-T	YJL149W	-0.678122	0.1053107	0.2238551	4.68	2.86	38.50	39.50
ORF-T	YGL249W	-0.677741	0.1175828	0.2412912	1.61	1.01	14.75	13.00
AST	AS_YKL118W	-0.677087	0.0884565	0.1961292	3.17	1.95	28.25	26.50
ORF-T	YPR063C	-0.676627	0.0193376	0.0598712	15.86	9.88	141.75	131.25
sn/snoRNA	SNR87*	-0.675374	0.0166876	0.0532357	667.41	417.91	5776.25	5247.00
ORF-T	YGL237C	-0.675364	0.043654	0.115449	6.25	3.90	54.75	49.75
ORF-T	YMR170C	-0.675267	0.0223458	0.0671578	18.56	11.59	166.25	153.25
ORF-T	YDR424C	-0.675108	0.0172656	0.0547332	22.64	14.19	208.75	182.00
ORF-T	YHR141C	-0.675091	0.0171414	0.0543566	558.73	349.93	5112.00	4513.00
AST	AS_YDL025W-A	-0.674666	0.0590841	0.1453205	3.17	1.98	28.50	25.75
ORF-T	YKL020C	-0.674642	0.0194059	0.0600418	33.36	20.83	285.25	274.50
ORF-T	YIL117C	-0.674488	0.0117097	0.0397043	51.13	32.07	477.50	412.75
SUT	SUT218	-0.674428	0.0622632	0.1511464	5.33	3.36	49.25	42.50

Class	Transcript name	RRP6 KO_vs_WT log2_fold _change	RRP6 KO_vs_WT p-value	RRP6 KO_vs_WT FDR	Ave Norm Reads in WT	Ave Norm Reads in RRP6	Average RAW read counts in WT	Average RAW read counts in RRP6
AST	AS_YLR331C	-0.674217	0.0633411	0.1530862	3.75	2.32	33.00	30.50
ORF-T	YPL037C	-0.674084	0.0128804	0.0430229	174.13	109.08	1528.25	1425.75
ORF-T	YLR350W	-0.673488	0.0376114	0.1027841	36.59	22.92	305.75	286.25
AST	AS_YOR378W	-0.673367	0.0662432	0.158387	6.50	4.03	57.75	55.00
ORF-T	YDR019C	-0.673077	0.0174393	0.0551625	58.90	37.02	564.50	462.75
ORF-T	YBL091C	-0.672905	0.0234537	0.0698072	121.47	76.17	1118.25	1009.00
ORF-T	YOR288C	-0.672823	0.0173391	0.0549199	13.22	8.28	118.00	106.75
ORF-T	YEL054C	-0.672709	0.0089589	0.0315658	456.26	286.23	4212.75	3739.50
ORF-T	YKR065C	-0.672556	0.0429933	0.1139406	15.24	9.52	134.50	127.50
ORF-T	YAL009W	-0.671914	0.0116202	0.0394709	20.44	12.78	183.50	170.50
ORF-T	YKL183W	-0.671105	0.0109588	0.0375497	42.63	26.79	401.50	350.75
ORF-T	YAR071W	-0.67109	0.1618893	0.3042289	6.91	4.37	63.50	52.50
AST	AS_YJR018W	-0.670719	0.0294506	0.0845374	9.41	5.87	84.50	79.25
ORF-T	YBL032W	-0.670702	0.0321734	0.0908023	108.10	67.86	1002.75	938.25
ORF-T	YNL079C	-0.670658	0.0500346	0.1278959	317.78	199.64	2880.25	2545.75
ORF-T	YER132C	-0.670383	0.0202907	0.0622108	17.75	11.15	160.75	145.25
ORF-T	YGR152C	-0.670257	0.0097462	0.033925	43.62	27.48	415.25	350.25
ORF-T	YKR058W	-0.670117	0.038088	0.1038895	353.69	222.30	3143.00	2766.75
ORF-T	YDR408C	-0.66942	0.0114392	0.03901	105.56	66.42	977.50	842.50
ORF-T	YPR113W	-0.669226	0.0178303	0.0562054	219.99	138.33	1963.25	1773.75
ORF-T	YKL067W	-0.669053	0.0494104	0.1267159	85.85	53.98	737.00	672.00
ORF-T	YEL005C	-0.668972	0.1010496	0.2170191	3.40	2.08	28.75	28.50
ORF-T	YGR151C	-0.668915	0.0198615	0.0611179	25.13	15.86	240.00	202.75
ORF-T	YKL160W	-0.667184	0.0267524	0.0779461	38.46	24.15	340.50	327.25
ORF-T	YML063W	-0.666632	0.0194106	0.0600418	2624.26	1653.23	23669.00	21098.50
NUT	NUT0953	-0.66656	0.0393383	0.1067226	1602.78	1009.77	14311.00	12880.75
ORF-T	YLR406C	-0.666172	0.0144954	0.0472894	114.12	71.90	1018.50	926.25
ORF-T	YMR307W	-0.665674	0.0260333	0.0763133	913.88	576.06	8196.25	7722.00
ORF-T	YMR053C	-0.665406	0.0813828	0.1844604	29.86	18.84	267.00	237.00

Class	Transcript name	RRP6 KO_vs_WT log2_fold _change	RRP6 KO_vs_WT p-value	RRP6 KO_vs_WT FDR	Ave Norm Reads in WT	Ave Norm Reads in RRP6	Average RAW read counts in WT	Average RAW read counts in RRP6
ORF-T	YER023W	-0.664731	0.0307272	0.0875207	103.78	65.44	941.25	854.75
ORF-T	YBR181C	-0.664573	0.0167511	0.0534043	927.72	585.30	8437.50	7372.00
ORF-T	YML078W	-0.664379	0.0212846	0.0646682	40.06	25.22	350.75	334.00
ORF-T	YHR152W	-0.664043	0.1412019	0.2770733	7.41	4.64	67.00	65.25
ORF-T	YLL022C	-0.663426	0.0137755	0.0453663	38.14	24.13	359.00	310.50
ORF-T	YDR034W-B	-0.663099	0.378442	0.5400041	17.38	11.00	160.75	124.50
ORF-T	YCR053W	-0.662819	0.0246106	0.0727354	99.11	62.53	859.00	837.75
SRT	SRT561	-0.662711	0.1850268	0.3332994	1.02	0.67	10.25	8.75
ORF-T	YGL146C	-0.662674	0.0319345	0.0903378	16.52	10.47	149.00	129.25
ORF-T	YMR146C	-0.662481	0.0055432	0.0212055	113.25	71.50	1021.75	954.00
ORF-T	YLL010C	-0.662412	0.0683135	0.1619355	10.52	6.58	90.25	89.75
ORF-T	YOR188W	-0.662265	0.0195044	0.060258	14.84	9.35	133.75	123.50
ORF-T	YDL191W	-0.662184	0.0153436	0.0496231	111.88	70.65	993.75	937.00
ORF-T	YJL190C	-0.661559	0.0261498	0.0765664	223.58	141.33	1992.75	1826.50
ORF-T	YDL104C	-0.661518	0.0562864	0.1404032	11.05	6.96	100.50	92.25
ORF-T	YDR513W	-0.661288	0.1154856	0.2386877	28.25	17.82	234.00	231.50
sn/snoRNA	SNR46	-0.661274	0.0284455	0.0820963	807.04	510.32	7120.00	6381.50
ORF-T	YJR070C	-0.660888	0.0094083	0.0329308	49.29	31.13	433.75	407.25
ORF-T	YNL131W	-0.660757	0.0231289	0.0690445	50.20	31.70	438.00	420.00
ORF-T	YIR023W	-0.660141	0.0499674	0.1278536	7.57	4.75	65.00	62.50
AST	AS_YBL100C	-0.659923	0.1112652	0.2323459	37.30	23.58	310.25	293.50
AST	AS_YJL067W	-0.659717	0.0807815	0.183634	4.27	2.67	37.50	35.50
ORF-T	YDR178W	-0.659541	0.0807786	0.183634	27.91	17.64	232.75	222.00
ORF-T	YLL018C	-0.659244	0.0130719	0.043547	217.78	137.87	2013.50	1856.50
ORF-T	YLR142W	-0.658535	0.0878692	0.1951945	8.62	5.44	76.75	71.75
ORF-T	YHL015W	-0.658424	0.019602	0.0605225	2697.72	1709.21	24799.00	21959.75
ORF-T	YJR014W	-0.657578	0.0137365	0.0452677	27.23	17.22	245.50	230.75
ORF-T	YIL015W	-0.657519	0.1526949	0.2916823	31.59	20.02	270.75	252.75
ORF-T	YLR287C-A	-0.657464	0.017669	0.0557666	275.75	174.79	2437.00	2282.00

Class	Transcript name	RRP6 KO_vs_WT log2_fold _change	RRP6 KO_vs_WT p-value	RRP6 KO_vs_WT FDR	Ave Norm Reads in WT	Ave Norm Reads in RRP6	Average RAW read counts in WT	Average RAW read counts in RRP6
ORF-T	YGL206C	-0.657197	0.0439341	0.1160073	189.63	120.21	1658.25	1575.50
AST	AS_YDL191W	-0.656756	0.1628597	0.3055969	0.98	0.61	9.00	8.25
ORF-T	YJR017C	-0.656531	0.0178792	0.0563242	21.38	13.51	193.25	183.25
NUT	NUT0239	-0.656508	0.1877267	0.3367792	1.10	0.72	10.25	9.00
ORF-T	YHR117W	-0.656338	0.0638339	0.1539954	49.95	31.65	456.75	431.25
AST	AS_YNL043C	-0.656227	0.0364016	0.1000707	9.49	6.03	84.50	76.50
ORF-T	YML085C	-0.656016	0.0200651	0.0616315	199.79	126.77	1832.75	1681.75
ORF-T	YDR225W	-0.655378	0.0099705	0.0345387	147.32	93.48	1328.50	1259.75
SRT	SRT560	-0.654893	0.0784053	0.1792833	14.66	9.34	130.75	116.50
ORF-T	YCL009C	-0.654658	0.0426409	0.1132448	41.10	26.06	350.50	336.75
ORF-T	YGL062W	-0.654391	0.0411263	0.110356	166.82	105.98	1451.50	1344.00
AST	AS_YER145C-A	-0.653824	0.012101	0.0407706	43.38	27.59	392.25	349.25
ORF-T	YER167W	-0.653819	0.0113949	0.0388722	45.12	28.63	394.75	373.75
ORF-T	YOR271C	-0.65373	0.0077989	0.0281577	79.35	50.43	738.00	668.00
ORF-T	YJR077C	-0.653639	0.0079466	0.0285477	87.89	55.88	780.00	703.75
ORF-T	YOR226C	-0.653639	0.0191915	0.059492	16.35	10.36	144.00	135.75
ORF-T	YOL104C	-0.653489	0.1400041	0.2750976	1.78	1.15	16.75	14.75
ORF-T	YPR174C	-0.65344	0.0210438	0.0640526	18.17	11.57	171.50	152.00
ORF-T	YJR082C	-0.653252	0.0525413	0.1328885	4.94	3.12	43.00	40.25
ORF-T	YMR124W	-0.653244	0.0179189	0.0564142	16.36	10.34	145.00	138.00
ORF-T	YHR061C	-0.651907	0.0569629	0.1415808	3.12	1.96	27.75	25.75
ORF-T	YOL038W	-0.651077	0.0214883	0.0651498	95.53	60.80	842.25	790.75
ORF-T	YOL030W	-0.651066	0.0340614	0.0949089	48.16	30.63	406.25	391.00
AST	AS_YOR015W	-0.650924	0.1071264	0.2267099	1.85	1.17	16.75	15.50
AST	AS_YPR126C	-0.650794	0.080897	0.1837718	7.43	4.68	67.00	65.25
ORF-T	YOR323C	-0.650707	0.007326	0.0267566	174.25	110.99	1625.50	1473.75
ORF-T	YJL034W	-0.650578	0.011377	0.0388303	449.45	286.31	4034.75	3696.00
ORF-T	YML012W	-0.650166	0.0133269	0.0441689	314.52	200.44	2858.00	2532.75
ORF-T	YBR048W	-0.65009	0.0199817	0.0614315	232.63	148.22	2097.75	1940.75

TABLE S1: Differential expression data for RRP6 RNA-Seq dataset Page 37

Class	Transcript name	RRP6 KO_vs_WT log2_fold _change	RRP6 KO_vs_WT p-value	RRP6 KO_vs_WT FDR	Ave Norm Reads in WT	Ave Norm Reads in RRP6	Average RAW read counts in WT	Average RAW read counts in RRP6
AST	AS_YPL073C	-0.649444	0.1589156	0.3001507	1.62	0.97	13.75	14.00
ORF-T	YML101C	-0.64903	0.0134162	0.0444239	19.66	12.56	176.00	158.00
ORF-T	YDR423C	-0.648981	0.0154908	0.0500031	46.66	29.73	416.75	389.25
ORF-T	YEL009C	-0.648949	0.02079	0.0633947	226.08	144.12	2021.75	1957.75
ORF-T	YNR062C	-0.647945	0.1827638	0.3309968	4.59	2.90	43.75	42.50
ORF-T	YML057W	-0.647522	0.0323585	0.0912481	21.50	13.68	185.00	178.25
ORF-T	YJL145W	-0.647369	0.0145214	0.047359	36.89	23.54	339.25	307.75
ORF-T	YBR128C	-0.647336	0.0687705	0.1628275	19.96	12.72	183.25	170.25
ORF-T	YHR121W	-0.646289	0.0221847	0.0667792	38.60	24.66	353.00	317.25
NUT	NUT0830	-0.64505	0.029432	0.0845082	122.32	78.20	1125.75	1037.25
ORF-T	YPL255W	-0.644982	0.0226358	0.0678537	14.99	9.55	136.25	128.00
ORF-T	YJL192C	-0.644952	0.0334061	0.093461	53.94	34.44	468.00	454.75
ORF-T	YJL026W	-0.644717	0.0264002	0.077098	204.14	130.53	1820.00	1731.00
ORF-T	YDR471W	-0.644312	0.0258483	0.0757936	167.23	106.98	1496.75	1390.50
ORF-T	YPR166C	-0.643621	0.060998	0.1486477	9.23	5.85	77.00	76.00
ORF-T	YPL105C	-0.643245	0.0262075	0.0766464	14.00	8.91	121.00	116.50
ORF-T	YML025C	-0.643058	0.091584	0.2012493	5.67	3.60	50.50	48.25
AST	AS_YER060W	-0.642641	0.0681281	0.1617241	4.33	2.78	37.75	34.75
ORF-T	YEL070W	-0.642633	0.1371892	0.271258	2.57	1.65	23.50	21.25
ORF-T	YNL052W	-0.642543	0.0355212	0.0982973	64.39	41.23	573.00	529.25
ORF-T	YER003C	-0.642493	0.0134943	0.0446299	110.06	70.46	1001.25	942.00
ORF-T	YKR057W	-0.642271	0.0276957	0.0803462	643.36	412.20	5706.25	5218.25
ORF-T	YGR148C	-0.642236	0.0182273	0.0570821	365.05	233.92	3319.25	2958.00
AST	AS_YJL116C	-0.642166	0.0660601	0.1581554	5.38	3.43	49.50	45.75
ORF-T	YFL033C	-0.64202	0.0329042	0.0925163	24.02	15.37	207.75	196.25
ORF-T	YBL099W	-0.641721	0.0968472	0.2101888	234.91	150.54	1951.50	1883.75
ORF-T	YHR071W	-0.64101	0.0480054	0.1241092	54.58	34.99	502.25	473.75
ORF-T	YHR208W	-0.640683	0.0719979	0.1682564	130.02	83.34	1094.00	1103.00
ORF-T	YMR144W	-0.640575	0.021985	0.0663366	16.27	10.47	154.25	136.50

Class	Transcript name	RRP6 KO_vs_WT log2_fold_change	RRP6 KO_vs_WT p-value	RRP6 KO_vs_WT FDR	Ave Norm Reads in WT	Ave Norm Reads in RRP6	Average RAW read counts in WT	Average RAW read counts in RRP6
ORF-T	YLR045C	-0.640289	0.0068297	0.0252642	51.64	33.12	471.25	437.75
ORF-T	YDL161W	-0.640246	0.053201	0.1340861	18.98	12.14	169.00	162.25
ORF-T	YHL001W	-0.639806	0.0232776	0.0693652	248.39	159.41	2243.75	2059.50
ORF-T	YDL169C	-0.638835	0.2851664	0.4486896	2.81	1.78	22.75	23.00
ORF-T	YFR018C	-0.638799	0.0102171	0.0353202	38.39	24.70	365.00	321.75
CUT	CUT153	-0.638756	0.0391949	0.1064481	107.14	68.74	922.50	939.50
ORF-T	YER165W	-0.638734	0.0130678	0.043547	347.60	223.25	3123.25	2881.25
ORF-T	YIL042C	-0.638451	0.0180755	0.0567502	16.04	10.27	146.50	137.75
AST	AS_YJL026C-A	-0.638243	0.0223504	0.0671578	65.51	42.05	588.75	556.50
AST	AS_YAL037C-B	-0.637078	0.0473	0.1226784	5572.90	3583.40	48690.25	47464.00
AST	AS_YOR082C	-0.636986	0.0996348	0.2149128	2.64	1.71	24.50	22.00
ORF-T	YPL153C	-0.636925	0.0381041	0.1039053	40.88	26.35	376.75	324.00
ORF-T	YMR222C	-0.636922	0.0223111	0.0671397	11.84	7.61	105.00	97.00
AST	AS_YLR149C-A	-0.635945	0.0170509	0.0541437	48.85	31.51	472.25	404.75
ORF-T	YGL022W	-0.635643	0.0214154	0.0649677	182.82	117.66	1618.25	1502.50
ORF-T	YDL220C	-0.635224	0.048481	0.1250744	10.28	6.57	92.50	89.50
sn/snoRNA	SNR36	-0.635111	0.0589828	0.145246	790.30	508.88	7061.00	6473.75
ORF-T	YMR203W	-0.634932	0.0400338	0.1080905	57.49	36.97	508.75	494.50
ORF-T	YDL082W	-0.634606	0.0175214	0.0553873	258.76	166.65	2347.25	2206.50
ORF-T	YPR036W-A	-0.634599	0.0706521	0.1662287	33.31	21.39	283.75	290.75
ORF-T	YOR373W	-0.634539	0.0197526	0.0608755	16.20	10.39	142.50	136.50
ORF-T	YLR259C	-0.633878	0.0427633	0.1134734	382.68	246.61	3449.75	3171.25
ORF-T	YDL051W	-0.633794	0.0151286	0.0490695	158.26	102.02	1474.00	1329.25
ORF-T	YGL254W	-0.63354	0.0237064	0.0704553	14.98	9.61	134.25	128.00
NUT	NUT0597	-0.633496	0.2511569	0.4123025	0.99	0.66	10.00	8.75
ORF-T	YOR328W	-0.633426	0.026461	0.0772309	39.52	25.46	377.00	350.25
ORF-T	YDL237W	-0.632878	0.0214356	0.0650094	52.54	33.85	470.75	441.50
ORF-T	YBR121C-A	-0.632762	0.0077331	0.02795	64.86	41.84	600.50	546.75
ORF-T	YOR332W	-0.632555	0.0155856	0.0502608	70.98	45.75	635.50	597.75

Class	Transcript name	RRP6 KO_vs_WT log2_fold _change	RRP6 KO_vs_WT p-value	RRP6 KO_vs_WT FDR	Ave Norm Reads in WT	Ave Norm Reads in RRP6	Average RAW read counts in WT	Average RAW read counts in RRP6
ORF-T	YKL162C-A	-0.631922	0.3043455	0.4699158	1.22	0.80	11.25	9.75
ORF-T	YHR123W	-0.631312	0.0252983	0.0744191	29.97	19.31	269.50	257.25
ORF-T	YBR038W	-0.631181	0.1133402	0.2351693	31.14	20.05	273.50	277.75
SUT	SUT608	-0.630708	0.1707268	0.3153131	4.35	2.79	36.25	35.75
ORF-T	YLR403W	-0.630602	0.0620067	0.1507051	16.07	10.34	136.25	134.25
ORF-T	YBL067C	-0.630431	0.0093182	0.0326832	43.69	28.21	397.25	369.75
ORF-T	YLL018C-A	-0.630367	0.0991456	0.2140408	5.29	3.36	45.00	45.50
ORF-T	YGL105W	-0.630057	0.0329615	0.0926375	269.13	173.86	2433.75	2304.50
ORF-T	YBL031W	-0.6297	0.0500125	0.1278717	6.17	3.98	56.75	52.50
AST	AS_YDL222C	-0.628573	0.1661362	0.3097321	2.37	1.54	20.75	19.25
ORF-T	YFR046C	-0.628493	0.0442195	0.1164705	6.56	4.21	59.75	56.75
ORF-T	YGL101W	-0.628439	0.0095874	0.0334531	61.37	39.69	567.75	526.75
sn/snoRNA	SNR50	-0.628211	0.0296266	0.0849243	477.75	309.07	4178.00	3984.00
ORF-T	YLR442C	-0.627787	0.0180888	0.0567716	28.50	18.43	264.00	246.75
ORF-T	YDR432W	-0.627469	0.027717	0.0803618	45.29	29.22	387.25	394.25
ORF-T	YMR301C	-0.627437	0.0251024	0.0739289	71.19	46.09	694.75	628.50
ORF-T	YER040W	-0.626998	0.0454241	0.1190686	12.89	8.32	111.50	106.75
AST	AS_YPR027C	-0.626875	0.0718165	0.1680273	12.26	7.93	108.00	101.25
AST	AS_YPL238C	-0.626861	0.0115176	0.0391978	48.64	31.47	424.75	404.25
ORF-T	YMR004W	-0.62686	0.0138935	0.0456657	37.00	23.91	334.25	321.50
AST	AS_YHR115C	-0.626562	0.138683	0.2733006	2.05	1.30	18.75	18.00
SUT	SUT524	-0.626121	0.1314982	0.262788	3.58	2.34	34.50	30.25
ORF-T	YDR447C	-0.626113	0.029302	0.0842416	1383.26	896.26	12507.50	11297.25
ORF-T	YGR149W	-0.625737	0.0598009	0.1465183	7.25	4.69	63.75	59.75
ORF-T	YPL250C	-0.625363	0.1177863	0.2416595	5.37	3.43	45.25	45.50
ORF-T	YLR200W	-0.625181	0.0414913	0.1109341	10.05	6.49	90.25	85.00
ORF-T	YOR369C	-0.624058	0.0206402	0.0630524	2516.75	1632.99	22795.00	20782.75
ORF-T	YAR033W	-0.623875	0.0860638	0.1921023	7.86	5.11	71.50	64.50
AST	AS_YCR049C	-0.623528	0.0527707	0.1333348	9.00	5.78	80.75	80.00

Class	Transcript name	RRP6 KO_vs_WT log2_fold _change	RRP6 KO_vs_WT p-value	RRP6 KO_vs_WT FDR	Ave Norm Reads in WT	Ave Norm Reads in RRP6	Average RAW read counts in WT	Average RAW read counts in RRP6
ORF-T	YHR076W	-0.623184	0.0144485	0.0471974	48.21	31.31	447.25	409.00
ORF-T	YLR011W	-0.622979	0.1490923	0.2869722	3.66	2.36	32.50	30.50
ORF-T	YMR189W	-0.622861	0.0272401	0.0792068	41.38	26.87	380.25	348.25
ORF-T	YBR191W	-0.622635	0.0416636	0.1112943	685.91	445.46	5989.25	5774.75
ORF-T	YBR289W	-0.622575	0.0329092	0.0925163	13.45	8.69	117.25	114.25
ORF-T	YLR330W	-0.622484	0.0308751	0.0878512	41.24	26.74	354.00	347.50
ORF-T	YDR454C	-0.622386	0.0185666	0.0579646	135.32	87.86	1196.75	1146.25
AST	AS_YOR331C	-0.62201	0.0223794	0.0672249	50.64	32.87	451.00	430.25
ORF-T	YDR320C-A	-0.621861	0.0630497	0.152656	20.20	13.13	182.50	166.25
ORF-T	YDR192C	-0.621578	0.0503526	0.1284807	9.88	6.38	88.25	85.75
ORF-T	YER118C	-0.621358	0.0366506	0.1006493	10.67	6.92	99.75	93.25
ORF-T	YBR039W	-0.621358	0.1375649	0.2717345	240.51	156.33	2088.50	1985.50
ORF-T	YPL234C	-0.621348	0.0299472	0.0856217	41.21	26.74	363.25	351.75
ORF-T	YDR480W	-0.620893	0.0825398	0.186747	4.59	2.94	38.75	38.00
ORF-T	YIR022W	-0.62056	0.0257359	0.075508	22.93	14.86	201.50	196.00
ORF-T	YOR351C	-0.620153	0.0989202	0.2137341	1.81	1.16	16.25	15.25
AST	AS_YDL026W	-0.620009	0.15603	0.2964218	2.32	1.49	20.00	19.00
ORF-T	YOR162C	-0.619835	0.0719461	0.1682564	28.89	18.75	248.25	249.75
AST	AS_YCR041W	-0.619382	0.1017318	0.218176	2.81	1.82	25.50	23.75
ORF-T	YNR034W	-0.619183	0.1035962	0.221327	13.76	8.93	120.75	117.50
ORF-T	YCL039W	-0.61904	0.0504679	0.1287422	15.94	10.37	144.25	135.00
ORF-T	YDR170C	-0.618939	0.0253297	0.0744896	173.58	112.99	1552.50	1484.25
ORF-T	YMR122W-A	-0.618313	0.0124494	0.0417491	74.09	48.21	660.75	641.00
AST	AS_YBR245C	-0.618064	0.1644409	0.307648	1.68	1.12	16.00	14.25
ORF-T	YLR201C	-0.617775	0.0896657	0.1980264	8.74	5.67	78.50	75.25
ORF-T	YNL243W	-0.617701	0.0388109	0.1055758	101.21	65.95	928.50	864.25
ORF-T	YBR059C	-0.617509	0.0582366	0.1438653	39.84	25.92	337.75	337.50
ORF-T	YBR130C	-0.617219	0.0762874	0.1759267	10.85	7.02	94.25	93.75
AST	AS_YGR121C	-0.616918	0.1262321	0.2548896	4.15	2.66	34.50	34.75

Class	Transcript name	RRP6 KO_vs_WT log2_fold _change	RRP6 KO_vs_WT p-value	RRP6 KO_vs_WT FDR	Ave Norm Reads in WT	Ave Norm Reads in RRP6	Average RAW read counts in WT	Average RAW read counts in RRP6
ORF-T	YCR063W	-0.616358	0.1929307	0.3424876	1.16	0.76	10.75	10.00
AST	AS_YDL050C	-0.616203	0.0320633	0.0905677	31.58	20.60	291.25	272.75
ORF-T	YKL113C	-0.615866	0.020369	0.062428	87.03	56.86	815.25	713.00
ORF-T	YDR428C	-0.615639	0.0673562	0.1603069	6.98	4.53	61.50	59.25
ORF-T	YDL076C	-0.615408	0.0331565	0.0930042	31.94	20.82	290.75	278.00
ORF-T	YLR265C	-0.61422	0.0416203	0.111208	13.79	8.99	123.50	117.25
ORF-T	YNL246W	-0.614013	0.0673132	0.16028	22.29	14.56	209.75	194.50
ORF-T	YLR121C	-0.613515	0.0393707	0.1067818	17.73	11.57	167.00	157.75
ORF-T	YNL184C	-0.613295	0.0733821	0.1706609	6.74	4.41	59.75	55.25
AST	AS_YAR018C	-0.613285	0.1123644	0.2336972	14.83	9.74	135.75	118.75
ORF-T	YDL216C	-0.612789	0.0492502	0.1264955	16.01	10.47	143.50	133.75
ORF-T	YMR104C	-0.612729	0.0456186	0.1194542	141.32	92.41	1291.25	1223.25
ORF-T	YBR191W-A	-0.612703	0.0357123	0.0987721	25.89	16.93	229.00	216.00
ORF-T	YDL046W	-0.612401	0.0332703	0.093168	28.80	18.82	247.50	236.50
ORF-T	YCR100C	-0.611801	0.0531373	0.1339926	10.02	6.52	90.00	87.25
ORF-T	YDR293C	-0.611549	0.0361179	0.099539	81.53	53.29	701.75	705.25
ORF-T	YLR449W	-0.611458	0.0097252	0.0338869	58.96	38.57	545.50	516.25
ORF-T	YGR244C	-0.611341	0.0502276	0.1283181	173.40	113.57	1587.50	1389.00
ORF-T	YNL078W	-0.61117	0.148129	0.2857726	6.28	4.05	53.75	56.25
ORF-T	YJR088C	-0.611119	0.0122294	0.0411344	55.26	36.15	502.00	476.25
AST	AS_YNL266W	-0.611024	0.1297645	0.2603552	3.31	2.13	28.75	28.50
ORF-T	YFL016C	-0.611006	0.034179	0.09519	77.16	50.53	685.75	630.25
ORF-T	YJR053W	-0.610573	0.0278431	0.0806062	13.07	8.54	120.25	114.50
ORF-T	YML109W	-0.61051	0.0141052	0.0462708	36.73	24.06	341.50	317.25
ORF-T	YGR034W	-0.61042	0.0178033	0.0561376	242.60	158.88	2157.00	2057.50
ORF-T	YLR278C	-0.610414	0.0354872	0.0982302	19.92	13.00	170.75	168.50
ORF-T	YOR070C	-0.610408	0.0700744	0.1652066	12.77	8.31	109.25	109.75
ORF-T	YDR181C	-0.610328	0.0250545	0.0738309	13.08	8.54	119.25	114.25
ORF-T	YPL162C	-0.610119	0.0420152	0.1120257	10.89	7.10	98.00	94.25

TABLE S1: Differential expression data for RRP6 RNA-Seq dataset Page 42

Class	Transcript name	RRP6 KO_vs_WT log2_fold_change	RRP6 KO_vs_WT p-value	RRP6 KO_vs_WT FDR	Ave Norm Reads in WT	Ave Norm Reads in RRP6	Average RAW read counts in WT	Average RAW read counts in RRP6
ORF-T	YOR195W	-0.609769	0.0141746	0.0464381	35.13	23.06	321.25	291.75
ORF-T	YBR121C	-0.609698	0.0155348	0.050129	371.58	243.51	3442.25	3194.25
ORF-T	YDR024W	-0.609635	0.0336388	0.093991	19.81	13.00	178.75	165.25
AST	AS_YNL114C	-0.609443	0.0585791	0.1445344	5.67	3.68	49.75	48.50
SUT	SUT593	-0.609363	0.2214614	0.377743	1.27	0.82	11.00	10.50
ORF-T	YLR307W	-0.609197	0.2397493	0.3990809	4.46	2.91	42.75	41.25
ORF-T	YJR067C	-0.609173	0.0409087	0.1099187	9.35	6.15	85.50	77.75
ORF-T	YPR102C	-0.608627	0.0229386	0.0685981	693.34	454.70	6258.50	5854.25
ORF-T	YHR018C	-0.608529	0.0645654	0.1553143	79.28	51.93	712.75	726.25
ORF-T	YGR032W	-0.608453	0.0960428	0.2089526	354.35	232.44	3294.00	3029.00
ORF-T	YAL041W	-0.608438	0.0193099	0.0598221	25.39	16.63	224.25	214.25
ORF-T	YML009C	-0.608351	0.062767	0.1522227	8.68	5.70	76.00	71.00
ORF-T	YAL032C	-0.60785	0.0460145	0.120241	13.28	8.66	119.25	119.00
ORF-T	YKL138C	-0.607337	0.0720548	0.1682726	6.60	4.29	57.00	56.00
ORF-T	YLL032C	-0.607103	0.0442067	0.1164705	27.43	17.96	244.25	239.75
AST	AS_YPR194C	-0.606927	0.081627	0.1849308	8.78	5.71	80.00	80.50
ORF-T	YAL054C	-0.606912	0.2835363	0.4472361	1.06	0.66	8.75	8.75
AST	AS_YMR319C	-0.606585	0.2089078	0.3614043	2.78	1.83	24.50	22.75
ORF-T	YOL158C	-0.606363	0.033084	0.092861	75.41	49.50	698.25	669.75
ORF-T	YMR312W	-0.606139	0.0948081	0.206772	4.83	3.16	43.50	41.25
ORF-T	YDR268W	-0.606066	0.0308661	0.0878504	29.51	19.40	269.00	248.00
AST	AS_YBR178W	-0.605892	0.1940033	0.3437707	2.13	1.38	19.50	19.00
ORF-T	YJL147C	-0.605187	0.0212978	0.0646889	14.67	9.67	133.25	122.50
ORF-T	YGR227W	-0.605111	0.0674671	0.160495	9.68	6.30	82.50	83.50
AST	AS_YDR243C	-0.604977	0.1809333	0.3287445	1.86	1.24	16.75	15.50
ORF-T	YCL061C	-0.604746	0.0227366	0.0680827	32.36	21.29	299.25	277.25
ORF-T	YNL152W	-0.603978	0.1578681	0.2988439	3.65	2.37	32.00	31.50
ORF-T	YML094W	-0.603509	0.0518401	0.1314458	7.63	4.99	66.25	65.00
ORF-T	YLR344W	-0.603486	0.0192758	0.0597348	33.86	22.24	299.50	292.50

TABLE S1: Differential expression data for RRP6 RNA-Seq dataset Page 43

Class	Transcript name	RRP6 KO_vs_WT log2_fold _change	RRP6 KO_vs_WT p-value	RRP6 KO_vs_WT FDR	Ave Norm Reads in WT	Ave Norm Reads in RRP6	Average RAW read counts in WT	Average RAW read counts in RRP6
AST	AS_YEL055C	-0.603122	0.1645873	0.3077506	1.73	1.15	16.50	15.25
ORF-T	YLR381W	-0.602743	0.0473395	0.1227493	8.93	5.84	80.25	77.75
ORF-T	YMR011W	-0.602624	0.0523568	0.1324883	1233.91	812.58	11453.50	10948.25
AST	AS_YCL041C	-0.602549	0.2396501	0.3990809	1.05	0.66	9.50	9.50
ORF-T	YGL250W	-0.602514	0.1771982	0.3237076	13.24	8.71	119.00	111.50
ORF-T	YGL150C	-0.60246	0.0139551	0.0458532	105.63	69.55	935.75	896.00
AST	AS_YMR193C-A	-0.602436	0.079539	0.1812589	6.40	4.21	57.75	55.25
ORF-T	YLR126C	-0.602309	0.0339456	0.0946385	19.65	12.96	179.25	167.50
ORF-T	YPL046C	-0.60221	0.0496087	0.1270647	7.34	4.83	65.50	62.00
ORF-T	YLR136C	-0.602022	0.0870346	0.1938289	6.32	4.16	57.50	55.50
ORF-T	YDL218W	-0.601984	0.1694341	0.3135847	2.81	1.82	26.00	25.25
ORF-T	YIL100W	-0.601609	0.2198633	0.3758623	1.13	0.77	11.00	9.75
ORF-T	YDL055C	-0.60144	0.0361615	0.0996046	3029.85	1996.97	27290.75	25302.75
ORF-T	YAL021C	-0.601331	0.0494664	0.1267646	19.94	13.07	168.75	174.00
ORF-T	YHR010W	-0.601308	0.0493884	0.1266937	1486.51	979.84	13215.50	12368.25
ORF-T	YEL026W	-0.600819	0.0413256	0.1106634	87.02	57.35	758.75	743.25
ORF-T	YDL229W	-0.6007	0.022334	0.0671578	1956.54	1290.21	17798.75	16763.00
ORF-T	YKL161C	-0.600634	0.0309509	0.0879879	21.40	14.13	205.00	189.50
AST	AS_YDR149C	-0.600293	0.0464293	0.1211052	15.88	10.43	137.75	137.25
ORF-T	YMR280C	-0.600056	0.1873789	0.3363947	38.99	25.71	334.00	324.50
ORF-T	YDL101C	-0.599922	0.0273803	0.0795686	18.57	12.25	165.75	156.50
ORF-T	YDR476C	-0.599748	0.0384056	0.1046707	15.03	9.87	129.75	129.50
ORF-T	YKL036C	-0.599714	0.0967597	0.2101496	5.06	3.31	43.25	42.50
ORF-T	YGR030C	-0.599649	0.0822296	0.1861706	6.11	4.00	54.00	53.00
AST	AS_YGR138C	-0.599536	0.3715614	0.5341207	4.43	2.92	44.75	42.50
ORF-T	YMR143W	-0.599466	0.0346025	0.0961774	326.69	215.63	2945.75	2721.25
ORF-T	YGR054W	-0.599124	0.0276035	0.0801248	33.81	22.27	300.75	294.25
ORF-T	YNL113W	-0.599121	0.035816	0.0989773	10.20	6.68	90.75	89.50
ORF-T	YML072C	-0.599038	0.1013325	0.2175046	218.56	144.24	1910.75	1935.75

Class	Transcript name	RRP6 KO_vs_WT log2_fold _change	RRP6 KO_vs_WT p-value	RRP6 KO_vs_WT FDR	Ave Norm Reads in WT	Ave Norm Reads in RRP6	Average RAW read counts in WT	Average RAW read counts in RRP6
ORF-T	YFL017C	-0.597761	0.0175594	0.0554901	20.14	13.30	183.00	173.75
ORF-T	YLR040C	-0.597529	0.1676723	0.3114972	2.03	1.32	18.00	17.50
ORF-T	YJL041W	-0.597135	0.0460852	0.1203633	24.26	15.98	206.00	208.00
ORF-T	YJL073W	-0.596987	0.0414958	0.1109341	16.72	11.03	149.00	143.75
ORF-T	YDR356W	-0.596436	0.0147005	0.0478193	41.06	27.11	364.50	354.75
SUT	SUT206	-0.596398	0.0782417	0.1790716	5.90	3.89	54.00	51.50
ORF-T	YPR034W	-0.596167	0.0312866	0.088705	55.73	36.84	501.50	484.25
ORF-T	YMR083W	-0.595535	0.0479504	0.1240462	519.58	343.85	4721.25	4432.50
ORF-T	YBL030C	-0.595533	0.0831381	0.1876797	174.29	115.31	1521.75	1492.50
SUT	SUT406	-0.595253	0.297108	0.4618526	4.88	3.21	46.50	47.50
ORF-T	YGR068C	-0.594382	0.0639629	0.1541961	8.25	5.40	71.75	73.50
ORF-T	YKL159C	-0.594097	0.0383413	0.1045237	8.88	5.88	82.00	77.25
ORF-T	YOR013W	-0.59368	0.1442913	0.281003	3.50	2.28	31.50	31.25
AST	AS_YDL228C	-0.593572	0.0220701	0.0665534	1216.98	806.50	11106.75	10457.75
NUT	NUT0653	-0.593442	0.0968611	0.2101888	18.69	12.37	169.50	162.50
SUT	SUT810	-0.593008	0.3121756	0.4774774	1.48	0.95	12.75	13.25
ORF-T	YIL162W	-0.592846	0.2169611	0.3719544	75.09	49.76	659.25	666.25
ORF-T	YJL137C	-0.592754	0.1848414	0.3330817	2.95	1.93	26.50	26.00
ORF-T	YDL208W	-0.592659	0.0290095	0.0835088	145.90	96.77	1317.50	1222.00
ORF-T	YDL137W	-0.592615	0.0182755	0.0571975	112.55	74.64	1004.50	949.50
ORF-T	YNR028W	-0.592266	0.0248174	0.0732823	20.90	13.80	185.50	184.75
ORF-T	YAL038W	-0.591818	0.0641384	0.1544713	8522.13	5654.44	74892.75	74792.75
AST	AS_YDR029W	-0.591615	0.085703	0.191625	5.80	3.84	51.50	49.25
sn/snoRNA	SNR40	-0.591588	0.0529964	0.1336708	2077.15	1378.42	18793.50	17729.25
ORF-T	YIR003W	-0.591535	0.081031	0.1838697	17.81	11.77	155.00	156.75
ORF-T	YPR004C	-0.591326	0.0077711	0.0280774	61.98	41.12	572.75	543.25
ORF-T	YLR304C	-0.591189	0.167186	0.3108229	478.68	317.73	4224.50	4036.50
SRT	SRT421	-0.591069	0.143859	0.2804462	4.64	3.10	43.50	39.50
SUT	SUT565	-0.591067	0.1438941	0.2804462	4.64	3.10	43.50	39.50

Class	Transcript name	RRP6 KO_vs_WT log2_fold _change	RRP6 KO_vs_WT p-value	RRP6 KO_vs_WT FDR	Ave Norm Reads in WT	Ave Norm Reads in RRP6	Average RAW read counts in WT	Average RAW read counts in RRP6
ORF-T	YGR086C	-0.590621	0.0375901	0.1027538	254.45	168.93	2283.50	2227.50
ORF-T	YPL120W	-0.590466	0.0790196	0.1803608	6.85	4.51	59.75	59.75
ORF-T	YKR073C	-0.590295	0.051451	0.130787	11.74	7.80	108.00	102.25
SUT	SUT421	-0.59007	0.0895419	0.197883	12.90	8.55	113.25	109.50
ORF-T	YDR469W	-0.589622	0.0409089	0.1099187	10.89	7.23	100.00	95.00
ORF-T	YDR382W	-0.589555	0.0261842	0.0766004	249.95	166.04	2215.00	2224.50
ORF-T	YHR135C	-0.589465	0.0313552	0.0888661	32.09	21.27	284.00	282.75
ORF-T	YJL144W	-0.58878	0.1660949	0.3097321	16.50	10.95	137.00	136.50
NUT	NUT0574	-0.588538	0.1084752	0.2284579	3.76	2.47	33.25	33.00
NUT	NUT1304	-0.588472	0.0567461	0.1413258	158.29	105.26	1446.75	1393.75
NUT	NUT1459	-0.588298	0.1402145	0.2754037	5.37	3.53	45.25	46.50
ORF-T	YNR040W	-0.588136	0.0697595	0.1646279	11.10	7.35	99.00	96.50
ORF-T	YMR315W-A	-0.588018	0.3165755	0.4821548	0.94	0.63	9.00	8.50
ORF-T	YJL095W	-0.587956	0.0305873	0.0872044	28.94	19.20	252.25	252.00
ORF-T	YHR064C	-0.587619	0.0266483	0.0777101	664.96	442.48	6005.25	5741.50
ORF-T	YMR070W	-0.587417	0.065003	0.156069	10.14	6.74	89.00	86.00
AST	AS_YNL160W	-0.587366	0.1358315	0.2696839	4.00	2.65	34.75	33.50
ORF-T	YML015C	-0.58709	0.0458572	0.1199346	12.55	8.34	115.75	110.25
AST	AS_YGL079W	-0.586881	0.1505127	0.2887032	4.46	2.98	42.00	39.25
ORF-T	YDL059C	-0.586707	0.0567865	0.1413736	8.96	5.98	80.25	75.25
ORF-T	YIL040W	-0.586706	0.095247	0.20749	4.96	3.28	44.00	42.75
ORF-T	YMR096W	-0.586655	0.0680855	0.1616609	12.18	8.07	111.25	111.25
AST	AS_YDR193W	-0.58649	0.1542762	0.293865	2.12	1.38	19.25	19.00
ORF-T	YGL198W	-0.586173	0.0297475	0.0850988	91.28	60.80	826.75	782.00
AST	AS_YNL174W	-0.586023	0.0465041	0.1212363	10.72	7.10	95.75	94.50
ORF-T	YJL025W	-0.585532	0.1269255	0.2559321	4.73	3.09	41.25	42.75
ORF-T	YJR065C	-0.585492	0.0569333	0.1415472	55.77	37.11	482.50	488.50
ORF-T	YNR045W	-0.585236	0.0768901	0.1769823	28.50	19.01	268.75	248.00
ORF-T	YBR120C	-0.585005	0.0950509	0.2071522	5.34	3.55	46.75	45.00

Class	Transcript name	RRP6 KO_vs_WT log2_fold _change	RRP6 KO_vs_WT p-value	RRP6 KO_vs_WT FDR	Ave Norm Reads in WT	Ave Norm Reads in RRP6	Average RAW read counts in WT	Average RAW read counts in RRP6
AST	AS_YHR200W	-0.584948	0.1394439	0.2742642	1.58	1.07	14.50	13.50
ORF-T	YCR033W	-0.584896	0.0292725	0.0841939	33.90	22.53	297.25	300.00
ORF-T	YIL131C	-0.584505	0.0385015	0.1049039	21.91	14.56	198.25	196.50
ORF-T	YLR404W	-0.584323	0.0407789	0.1097086	13.20	8.76	118.00	116.00
ORF-T	YGL011C	-0.583889	0.0573984	0.1422189	48.36	32.20	414.75	425.50
ORF-T	YPR132W	-0.583809	0.0406487	0.1093946	1056.29	704.78	9496.25	8844.25
ORF-T	YGL014W	-0.583597	0.0341812	0.09519	24.84	16.54	214.75	212.25
ORF-T	YIL076W	-0.583359	0.0132953	0.0441163	123.32	82.30	1122.75	1073.00
ORF-T	YML100W-A	-0.583035	0.2058422	0.3578199	1.89	1.27	16.75	15.50
ORF-T	YNL165W	-0.582991	0.0344975	0.0959385	14.79	9.86	133.25	127.25
SUT	SUT195	-0.582726	0.1492593	0.2871291	3.50	2.29	29.75	31.00
ORF-T	YHL007C	-0.582357	0.0242118	0.071704	27.71	18.48	243.50	238.75
ORF-T	YBR146W	-0.582198	0.0641123	0.1544713	22.67	15.10	198.75	197.50
ORF-T	YIL135C	-0.582148	0.1281305	0.2580523	4.23	2.78	36.00	37.00
NUT	NUT0628	-0.58188	0.0570883	0.1416879	2082.55	1391.34	18842.75	17890.50
ORF-T	YDL085C-A	-0.581848	0.0445874	0.1172716	11.61	7.74	105.00	100.50
ORF-T	YKL016C	-0.581605	0.0994808	0.2146724	80.20	53.55	689.50	691.50
ORF-T	YCL024W	-0.581508	0.0716382	0.1677501	29.32	19.61	271.00	250.50
ORF-T	YBR223C	-0.581119	0.0890856	0.19708	9.54	6.32	82.25	84.00
ORF-T	YBR140C	-0.581057	0.0296915	0.085011	95.04	63.48	844.50	841.25
ORF-T	YPR096C	-0.581014	0.2284717	0.3855269	1.72	1.11	14.00	14.50
ORF-T	YOR305W	-0.580881	0.0743491	0.1725119	6.01	3.98	53.50	53.25
ORF-T	YBR009C	-0.580842	0.0360684	0.0994297	172.19	115.06	1543.00	1560.25
ORF-T	YLR239C	-0.580786	0.1536896	0.2933016	5.82	3.84	52.25	54.25
ORF-T	YJR144W	-0.580415	0.0165076	0.0527783	127.52	85.31	1167.75	1090.50
ORF-T	YBR132C	-0.580298	0.0906581	0.1996933	34.13	22.78	289.50	296.75
ORF-T	YGR060W	-0.580115	0.0267035	0.077826	105.29	70.37	923.75	924.75
ORF-T	YML058W	-0.580075	0.0420106	0.1120257	29.64	19.77	260.00	264.50
ORF-T	YNL283C	-0.58004	0.0646043	0.1553338	12.72	8.44	110.75	114.50

Class	Transcript name	RRP6 KO_vs_WT log2_fold _change	RRP6 KO_vs_WT p-value	RRP6 KO_vs_WT FDR	Ave Norm Reads in WT	Ave Norm Reads in RRP6	Average RAW read counts in WT	Average RAW read counts in RRP6
ORF-T	YNL080C	-0.579644	0.0608398	0.1484723	8.77	5.82	77.75	78.00
ORF-T	YDR150W	-0.57927	0.0562685	0.1404032	129.08	86.32	1118.50	1157.25
ORF-T	YDR116C	-0.579007	0.0696511	0.164449	11.61	7.73	102.50	102.00
ORF-T	YJL170C	-0.578851	0.2618601	0.4242605	2.83	1.89	25.75	24.75
ORF-T	YDR285W	-0.578773	0.0884857	0.1961507	5.89	3.92	52.25	52.00
AST	AS_YER152W-A	-0.578448	0.0349629	0.0969772	14.81	9.95	138.25	128.25
ORF-T	YMR117C	-0.577986	0.048024	0.1241092	12.77	8.54	120.00	115.50
ORF-T	YDR063W	-0.577802	0.1359411	0.2697952	19.13	12.77	164.25	167.50
ORF-T	YHR178W	-0.577703	0.0299466	0.0856217	16.90	11.31	155.00	148.25
ORF-T	YKL182W	-0.577477	0.0652788	0.156521	998.02	668.82	9166.75	8683.75
AST	AS_YCL046W	-0.57727	0.2068519	0.3592035	5.36	3.55	45.00	47.50
ORF-T	YER015W	-0.577214	0.0758701	0.1752448	14.11	9.44	125.00	123.50
ORF-T	YDR453C	-0.57719	0.1507264	0.2889043	6.36	4.24	53.50	53.75
ORF-T	YLR294C	-0.577152	0.1710516	0.3156816	7.57	5.04	64.75	65.50
ORF-T	YDR466W	-0.577116	0.1097508	0.2300326	14.48	9.69	126.00	124.25
ORF-T	YOR098C	-0.577077	0.0199268	0.0612813	111.02	74.36	1000.00	994.25
ORF-T	YOL100W	-0.57668	0.0472175	0.1225274	14.30	9.51	121.50	126.25
ORF-T	YPL218W	-0.576586	0.033412	0.093461	79.72	53.39	680.75	697.75
ORF-T	YCR048W	-0.576546	0.0294214	0.0845016	34.03	22.75	310.00	312.25
ORF-T	YPL190C	-0.576341	0.0615444	0.1497982	33.32	22.28	284.75	294.50
ORF-T	YMR184W	-0.576337	0.0138297	0.0455004	48.99	32.84	449.25	433.00
ORF-T	YOR167C	-0.576323	0.0495282	0.1268908	496.44	332.93	4401.00	4286.75
ORF-T	YJL066C	-0.576011	0.0945073	0.2064135	9.30	6.20	82.00	82.00
ORF-T	YHR082C	-0.575098	0.0212422	0.0645589	92.09	61.76	817.00	816.25
ORF-T	YOR301W	-0.57489	0.0459442	0.1200885	21.34	14.29	191.75	189.25
ORF-T	YLR116W	-0.574865	0.0737338	0.1713996	7.27	4.87	66.00	64.00
sn/snoRNA	SNR128	-0.574196	0.1188599	0.2433177	1525.27	1024.43	12754.00	12936.25
ORF-T	YLR263W	-0.574056	0.1060341	0.22501	79.92	53.64	685.75	699.50
ORF-T	YMR149W	-0.574038	0.0518405	0.1314458	55.32	37.11	480.00	485.25

Class	Transcript name	RRP6 KO_vs_WT log2_fold _change	RRP6 KO_vs_WT p-value	RRP6 KO_vs_WT FDR	Ave Norm Reads in WT	Ave Norm Reads in RRP6	Average RAW read counts in WT	Average RAW read counts in RRP6
ORF-T	YJR078W	-0.573778	0.1733267	0.3189464	20.80	14.00	195.50	179.75
ORF-T	YDR462W	-0.573166	0.1286298	0.2587994	3.61	2.40	31.50	31.25
ORF-T	YKL127W	-0.573121	0.2296732	0.3872951	159.55	107.25	1503.25	1392.00
AST	AS_YJR153W	-0.572784	0.1820586	0.3301347	3.08	2.01	25.75	27.25
NUT	NUT0693	-0.572655	0.047667	0.1235347	483.15	324.84	4227.25	4180.75
ORF-T	YDR320C	-0.572426	0.1507726	0.2889378	5.66	3.75	49.00	51.75
AST	AS_YGL199C	-0.572329	0.0346408	0.0962574	66.68	44.84	605.00	578.50
ORF-T	YBL086C	-0.572102	0.105245	0.2238066	16.98	11.41	149.00	145.00
ORF-T	YAR018C	-0.572064	0.1003162	0.2159207	29.26	19.64	272.00	275.75
ORF-T	YDL241W	-0.571056	0.0730577	0.170024	13.52	9.07	125.25	121.75
ORF-T	YNL305C	-0.569769	0.1200406	0.245237	11.79	7.93	100.00	99.25
ORF-T	YLR181C	-0.569633	0.042412	0.1128451	18.26	12.27	161.25	159.50
ORF-T	YOL012C	-0.569422	0.068311	0.1619355	9.60	6.42	82.75	84.75
ORF-T	YAL055W	-0.569299	0.0662748	0.1584059	7.56	5.05	65.75	66.75
NUT	NUT0290	-0.568928	0.1118565	0.2332908	6.97	4.73	65.00	60.00
ORF-T	YDR044W	-0.568431	0.1146754	0.2375975	37.70	25.38	325.50	333.50
ORF-T	YEL038W	-0.568342	0.0540281	0.1359669	41.89	28.23	387.25	375.25
ORF-T	YBR184W	-0.568103	0.1280369	0.257967	3.16	2.12	28.50	27.75
ORF-T	YLR204W	-0.567983	0.1150519	0.2380352	6.03	4.02	52.50	53.75
ORF-T	YDR278C	-0.567795	0.0857302	0.1916435	130.96	88.30	1087.75	1128.00
ORF-T	YLR202C	-0.567663	0.0854319	0.19129	4.31	2.91	40.00	38.00
ORF-T	YNL190W	-0.567408	0.0788068	0.1799157	83.93	56.57	703.75	742.75
ORF-T	YHR198C	-0.567265	0.1651022	0.3084331	4.71	3.15	39.50	40.00
ORF-T	YLR179C	-0.567189	0.1397377	0.2747349	70.80	47.77	628.75	613.25
AST	AS_YJR087W	-0.567053	0.0360262	0.0993404	20.57	13.87	186.50	181.50
ORF-T	YFR049W	-0.56698	0.1954279	0.3454934	21.79	14.67	183.00	189.00
AST	AS_YGR221C	-0.566269	0.0657865	0.1575752	20.46	13.86	193.25	178.50
ORF-T	YPL219W	-0.566193	0.1666734	0.3102703	7.56	5.09	65.50	64.75
ORF-T	YDR343C	-0.565817	0.36482	0.5279658	18.72	12.64	149.25	149.00

Class	Transcript name	RRP6 KO_vs_WT log2_fold _change	RRP6 KO_vs_WT p-value	RRP6 KO_vs_WT FDR	Ave Norm Reads in WT	Ave Norm Reads in RRP6	Average RAW read counts in WT	Average RAW read counts in RRP6
ORF-T	YNL007C	-0.56569	0.0963166	0.2093226	139.83	94.46	1206.50	1184.50
ORF-T	YBL090W	-0.565643	0.0608172	0.1484581	18.60	12.59	171.25	160.75
ORF-T	YDL095W	-0.56562	0.0252296	0.0742601	253.47	171.26	2312.25	2227.00
AST	AS_YBL057C	-0.565611	0.0832827	0.1878156	5.81	3.95	53.50	50.50
ORF-T	YDR146C	-0.565134	0.1781004	0.3250026	12.74	8.56	111.00	120.50
ORF-T	YHR182W	-0.564858	0.1858093	0.3345297	2.29	1.57	21.25	19.75
ORF-T	YMR086W	-0.564834	0.0487469	0.1255269	29.77	20.06	253.00	261.00
ORF-T	YNL071W	-0.564668	0.0872612	0.1941619	118.53	80.11	1042.00	1034.25
ORF-T	YEL033W	-0.564521	0.0330325	0.0927597	60.93	41.22	537.75	516.00
ORF-T	YBR230W-A	-0.564309	0.1701722	0.3145191	2.56	1.73	22.25	21.50
ORF-T	YIR021W-A	-0.564017	0.093042	0.2037429	4.40	2.97	40.75	39.25
ORF-T	YDL054C	-0.563894	0.0484843	0.1250744	12.10	8.20	107.50	102.75
ORF-T	YHR206W	-0.563366	0.0691499	0.1635336	25.12	16.96	223.50	226.00
ORF-T	YGL106W	-0.562621	0.0397558	0.1076235	44.03	29.77	400.75	395.50
ORF-T	YGL020C	-0.562366	0.0200834	0.0616691	57.94	39.21	515.00	506.50
ORF-T	YGL079W	-0.562221	0.1328731	0.2650697	4.48	2.98	37.75	39.25
ORF-T	YPL061W	-0.56193	0.0768546	0.1769412	1216.88	824.31	11363.50	10997.75
ORF-T	YMR241W	-0.561706	0.0266429	0.0777101	176.62	119.68	1653.75	1568.00
ORF-T	YLR454W	-0.561646	0.1336738	0.266344	120.15	81.37	1072.00	1084.25
SUT	SUT609	-0.561619	0.2123821	0.3661178	1.19	0.80	10.25	10.00
AST	AS_YMR304C-A	-0.561484	0.2258079	0.3826081	1.77	1.18	16.00	15.75
ORF-T	YOR015W	-0.56068	0.1183917	0.242507	4.35	2.92	37.75	37.75
ORF-T	YPR100W	-0.560545	0.0606586	0.1482358	11.09	7.53	98.50	94.25
ORF-T	YER111C	-0.560523	0.060763	0.1483918	15.18	10.27	131.75	131.00
ORF-T	YHR021C	-0.560382	0.0628014	0.1522329	219.54	148.86	1934.75	1908.75
ORF-T	YIL044C	-0.560221	0.0575993	0.1425704	20.75	14.00	186.75	192.50
ORF-T	YIR020C	-0.560096	0.1902547	0.3394423	3.03	2.05	27.50	26.50
AST	AS_YIR027C	-0.560082	0.1673299	0.311033	3.40	2.27	29.25	30.50
ORF-T	YDR519W	-0.559997	0.0270888	0.078835	50.10	34.02	462.50	434.00

Class	Transcript name	RRP6 KO_vs_WT log2_fold _change	RRP6 KO_vs_WT p-value	RRP6 KO_vs_WT FDR	Ave Norm Reads in WT	Ave Norm Reads in RRP6	Average RAW read counts in WT	Average RAW read counts in RRP6
ORF-T	YHR159W	-0.559773	0.0731469	0.1701922	13.26	8.95	118.25	119.75
ORF-T	YNL087W	-0.559769	0.0389084	0.1057839	282.22	191.47	2640.25	2535.00
ORF-T	YJR048W	-0.559574	0.2090604	0.3615444	35.93	24.36	305.00	301.75
ORF-T	YHR011W	-0.559358	0.0572795	0.1420357	14.29	9.72	133.00	125.25
ORF-T	YER007C-A	-0.559339	0.0184377	0.0576156	40.68	27.56	368.00	367.25
ORF-T	YGR118W	-0.559219	0.042936	0.1138487	1267.75	860.42	11664.75	10896.25
ORF-T	YGL065C	-0.559081	0.0669661	0.1596422	9.86	6.65	86.75	87.50
ORF-T	YPL166W	-0.55887	0.1747107	0.3206157	2.65	1.80	24.00	23.25
ORF-T	YDR433W	-0.55883	0.0827234	0.1869747	16.00	10.79	137.25	146.75
ORF-T	YOR252W	-0.558641	0.0277108	0.0803618	17.59	11.96	162.50	155.00
ORF-T	YNL031C	-0.558424	0.0527615	0.1333348	524.01	355.78	4728.25	4738.00
ORF-T	YOR073W	-0.558383	0.0468415	0.1218337	17.97	12.17	165.50	163.75
ORF-T	YDR511W	-0.558312	0.1661502	0.3097321	3.47	2.33	29.75	30.00
ORF-T	YML026C	-0.557373	0.059988	0.1468963	676.14	459.47	6060.00	5821.25
ORF-T	YJL104W	-0.557319	0.0311618	0.0884424	30.18	20.51	269.00	261.25
ORF-T	YPL068C	-0.557314	0.1844329	0.3326842	1.71	1.13	15.25	15.50
ORF-T	YDR507C	-0.557168	0.0217173	0.0656664	85.27	57.99	796.25	748.00
ORF-T	YGR181W	-0.557026	0.1084971	0.2284579	5.47	3.69	48.25	48.00
ORF-T	YIL074C	-0.556891	0.0331521	0.0930042	115.87	78.78	1043.50	1001.00
ORF-T	YDL156W	-0.556506	0.0621655	0.1509948	11.33	7.69	100.75	99.00
ORF-T	YGL025C	-0.556478	0.0758745	0.1752448	8.71	5.88	78.25	79.00
ORF-T	YDL197C	-0.556421	0.0322293	0.0909091	23.23	15.75	205.00	204.75
ORF-T	YFR026C	-0.555807	0.0684768	0.1622083	7.57	5.08	66.00	69.25
ORF-T	YGL135W	-0.55551	0.0363786	0.1000707	708.19	481.86	6397.50	6252.25
ORF-T	YPL208W	-0.555268	0.0232177	0.0692274	74.68	50.86	677.50	641.00
ORF-T	YKR043C	-0.554703	0.0377075	0.1030188	86.56	58.90	808.75	798.00
ORF-T	YNL242W	-0.554308	0.0609624	0.1485967	59.16	40.28	538.50	523.75
ORF-T	YDR055W	-0.554217	0.0441585	0.1164472	57.52	39.16	521.50	515.00
ORF-T	YLL021W	-0.554216	0.0169864	0.0540008	51.46	35.06	468.25	449.50

Class	Transcript name	RRP6 KO_vs_WT log2_fold _change	RRP6 KO_vs_WT p-value	RRP6 KO_vs_WT FDR	Ave Norm Reads in WT	Ave Norm Reads in RRP6	Average RAW read counts in WT	Average RAW read counts in RRP6
ORF-T	YDR085C	-0.55396	0.1683866	0.3124789	27.27	18.56	254.00	253.00
ORF-T	YPR008W	-0.553872	0.0793228	0.1809709	17.45	11.83	148.00	154.00
ORF-T	YDL103C	-0.553597	0.0160924	0.0516966	69.06	47.05	633.00	615.25
ORF-T	YLR161W	-0.553591	0.1045419	0.2228283	873.62	595.24	7655.25	7229.75
ORF-T	YNR014W	-0.553088	0.2549176	0.4166271	5.29	3.60	43.75	43.50
ORF-T	YHR059W	-0.553059	0.1622561	0.3046909	3.14	2.11	27.00	27.50
SRT	SRT283	-0.552877	0.2380211	0.397566	1.22	0.82	12.00	11.75
AST	AS_YCR050C	-0.552668	0.0603258	0.1476518	10.35	7.05	97.00	94.25
ORF-T	YBL021C	-0.55264	0.0851421	0.1909213	10.27	6.99	92.25	90.50
ORF-T	YPL183W-A	-0.552611	0.0688125	0.1628799	8.20	5.56	70.50	70.75
ORF-T	YOL147C	-0.552517	0.0948149	0.206772	6.54	4.43	57.75	58.50
SUT	SUT394	-0.552273	0.1412674	0.2771479	3.99	2.75	36.25	34.25
ORF-T	YGL019W	-0.552202	0.0215305	0.0652385	80.52	54.90	727.25	712.25
ORF-T	YHR168W	-0.551872	0.032015	0.0904818	26.87	18.38	251.25	234.25
ORF-T	YKL105C	-0.551079	0.0684442	0.1621689	21.68	14.77	186.50	186.75
ORF-T	YGR188C	-0.551067	0.1179089	0.2418024	8.71	5.98	80.75	74.75
ORF-T	YFL008W	-0.550811	0.0285061	0.0822478	32.19	21.96	284.00	279.00
ORF-T	YNL044W	-0.550545	0.0407871	0.1097086	21.32	14.56	191.25	187.00
ORF-T	YOR040W	-0.550532	0.0412316	0.1104621	15.34	10.47	138.00	135.50
ORF-T	YNL039W	-0.55034	0.0181183	0.0568405	36.10	24.63	330.25	325.00
ORF-T	YLR295C	-0.550079	0.1666641	0.3102703	16.82	11.44	140.50	148.50
ORF-T	YPL269W	-0.549904	0.0637476	0.153861	8.44	5.73	73.00	73.75
SUT	SUT626	-0.54987	0.2481988	0.4090271	1.97	1.36	18.50	17.25
ORF-T	YJL136C	-0.549716	0.0446804	0.1174244	91.27	62.33	802.00	797.00
ORF-T	YKL025C	-0.549422	0.0979689	0.2120451	8.00	5.44	70.25	70.50
sn/snoRNA	SNR42*	-0.549369	0.0680673	0.1616609	3064.50	2094.03	26570.25	26216.25
ORF-T	YKR045C	-0.549294	0.1388475	0.2733721	4.67	3.15	43.25	44.50
NUT	NUT0486	-0.549242	0.1539601	0.2935905	4.50	3.07	40.25	39.75
SUT	SUT443	-0.54921	0.2986565	0.4634737	0.92	0.62	8.25	8.00

Class	Transcript name	RRP6 KO_vs_WT log2_fold _change	RRP6 KO_vs_WT p-value	RRP6 KO_vs_WT FDR	Ave Norm Reads in WT	Ave Norm Reads in RRP6	Average RAW read counts in WT	Average RAW read counts in RRP6
ORF-T	YDR262W	-0.54897	0.0330049	0.092708	27.88	19.04	254.75	253.25
CUT	CUT552	-0.548934	0.1975058	0.3479469	1.47	1.00	13.75	13.50
ORF-T	YOR286W	-0.548407	0.1022822	0.2192631	10.08	6.82	85.75	91.00
ORF-T	YLR425W	-0.547377	0.0420497	0.112088	20.56	14.02	182.00	185.75
ORF-T	YBR282W	-0.54671	0.0541529	0.1361982	13.66	9.33	122.00	121.00
ORF-T	YGR195W	-0.546453	0.0633036	0.1530862	14.81	10.10	131.25	133.00
ORF-T	YDR084C	-0.546251	0.1153798	0.2385667	10.53	7.17	92.00	95.00
ORF-T	YPR042C	-0.545983	0.1122405	0.2336972	27.88	19.05	231.00	243.00
ORF-T	YNL102W	-0.545605	0.0349843	0.0969981	50.50	34.64	462.00	433.25
ORF-T	YDR036C	-0.545435	0.1015643	0.2179557	10.40	7.07	91.25	95.25
AST	AS_YJR023C	-0.544368	0.2347802	0.3935379	2.65	1.80	22.00	22.25
ORF-T	YPL106C	-0.543954	0.0499972	0.1278651	732.82	502.63	6477.25	6375.00
ORF-T	YKL157W	-0.543867	0.0744282	0.1726555	302.55	207.54	2759.50	2668.00
ORF-T	YIL113W	-0.543712	0.1916433	0.341075	4.02	2.75	35.00	35.00
ORF-T	YDR122W	-0.543582	0.0710859	0.1670153	93.87	64.39	828.00	824.25
ORF-T	YNL237W	-0.543175	0.1165309	0.2398649	7.85	5.39	69.50	68.50
ORF-T	YGL035C	-0.542867	0.0568262	0.1413736	26.26	18.00	234.00	234.25
ORF-T	YOR215C	-0.542682	0.1615165	0.3038187	30.24	20.75	266.75	262.25
ORF-T	YFR041C	-0.542429	0.0230147	0.0687239	47.73	32.78	433.25	421.50
ORF-T	YBR286W	-0.542341	0.0858909	0.191875	268.11	184.04	2317.25	2419.00
ORF-T	YDL189W	-0.5422	0.0487811	0.125551	27.01	18.51	245.00	247.25
AST	AS_YMR272W-A	-0.542029	0.1150824	0.2380495	9.23	6.27	77.00	82.50
ORF-T	YNL265C	-0.541804	0.1222403	0.2487731	6.63	4.49	56.00	60.25
ORF-T	YER020W	-0.541801	0.0362942	0.0999428	37.04	25.39	328.75	337.50
ORF-T	YMR031C	-0.541689	0.0700867	0.1652066	61.73	42.39	532.00	537.50
ORF-T	YHR184W	-0.54154	0.1583594	0.2994367	6.16	4.24	56.75	55.75
ORF-T	YPL091W	-0.541435	0.0348213	0.0967057	243.66	167.46	2259.75	2142.25
ORF-T	YJL106W	-0.541072	0.0489934	0.126001	21.89	15.01	201.75	203.50
ORF-T	YOR232W	-0.541025	0.0648652	0.1558124	83.62	57.47	770.00	747.25

Class	Transcript name	RRP6 KO_vs_WT log2_fold _change	RRP6 KO_vs_WT p-value	RRP6 KO_vs_WT FDR	Ave Norm Reads in WT	Ave Norm Reads in RRP6	Average RAW read counts in WT	Average RAW read counts in RRP6
ORF-T	YDR440W	-0.540756	0.0598602	0.146619	34.03	23.39	323.25	312.75
ORF-T	YKR096W	-0.540646	0.0349323	0.0969604	40.50	27.76	361.50	375.75
ORF-T	YKR051W	-0.540029	0.0198	0.0609844	45.13	31.06	418.25	404.75
AST	AS_YER133W-A	-0.539949	0.1581994	0.2992463	8.75	5.97	74.00	78.50
ORF-T	YDL025C	-0.539446	0.046683	0.1216099	54.99	37.80	475.50	483.00
ORF-T	YPL215W	-0.539239	0.1051111	0.2236162	8.27	5.65	70.75	73.25
ORF-T	YEL051W	-0.538684	0.0411414	0.1103671	219.02	150.75	1997.75	1986.25
ORF-T	YPR153W	-0.538603	0.2032129	0.3546554	1.59	1.11	14.75	14.25
ORF-T	YDR246W	-0.53857	0.1007025	0.2164749	15.81	10.84	142.00	145.50
ORF-T	YGR021W	-0.538483	0.0506627	0.1291739	27.20	18.74	246.00	236.50
ORF-T	YDL200C	-0.538448	0.0465156	0.1212363	14.02	9.66	128.25	125.25
ORF-T	YBL039W-B	-0.538084	0.1678727	0.3117547	5.18	3.52	43.75	46.50
ORF-T	YMR114C	-0.538047	0.1197602	0.2448128	7.44	5.09	64.75	67.00
ORF-T	YJL138C	-0.5378	0.0360119	0.0993281	85.31	58.79	762.25	737.00
ORF-T	YDR316W	-0.53768	0.0758147	0.1752158	15.14	10.41	136.25	136.25
sn/snoRNA	SNR52	-0.537527	0.1202454	0.2456055	7784.48	5363.15	69400.25	66704.25
ORF-T	YKL114C	-0.537224	0.0359996	0.099325	22.69	15.64	207.25	203.00
ORF-T	YCL033C	-0.537213	0.0851205	0.1909152	16.81	11.61	159.75	150.75
NUT	NUT0972	-0.53713	0.0333114	0.093257	27.38	18.87	245.25	240.75
ORF-T	YGL148W	-0.53708	0.0453576	0.1189252	330.76	227.96	3020.00	2936.75
ORF-T	YNR021W	-0.537024	0.0593222	0.1456904	119.27	82.14	1046.00	1088.00
AST	AS_YDL172C	-0.536828	0.0418005	0.1116304	15.03	10.34	133.25	133.00
ORF-T	YPL111W	-0.536757	0.0665569	0.1589673	157.21	108.39	1471.25	1428.00
ORF-T	YAL060W	-0.536558	0.1374405	0.2715419	106.89	73.72	955.00	890.25
AST	AS_YHR069C-A	-0.536405	0.1183407	0.242501	10.72	7.35	101.00	103.50
ORF-T	YDL107W	-0.536215	0.1090737	0.2293368	8.59	5.92	76.25	75.50
ORF-T	YJR094W-A	-0.536115	0.0754008	0.1744304	1065.98	735.14	9670.00	9400.50
ORF-T	YML107C	-0.535995	0.0950081	0.2071036	11.05	7.59	101.00	101.75
ORF-T	YOL007C	-0.535973	0.0971777	0.2106945	21.05	14.53	191.75	186.25

Class	Transcript name	RRP6 KO_vs_WT log2_fold _change	RRP6 KO_vs_WT p-value	RRP6 KO_vs_WT FDR	Ave Norm Reads in WT	Ave Norm Reads in RRP6	Average RAW read counts in WT	Average RAW read counts in RRP6
ORF-T	YMR132C	-0.535806	0.0801064	0.182346	10.91	7.55	103.50	99.00
SRT	SRT320	-0.534674	0.1157371	0.2390237	10.12	6.93	91.75	97.25
ORF-T	YDR385W	-0.533875	0.0880368	0.1954134	376.09	259.73	3250.50	3338.00
ORF-T	YMR226C	-0.533746	0.0438636	0.1158817	132.07	91.20	1178.00	1185.00
ORF-T	YER086W	-0.533616	0.0562596	0.1404032	30.45	20.98	266.75	277.25
ORF-T	YMR238W	-0.533572	0.0883584	0.1959548	202.25	139.79	1913.25	1760.75
AST	AS_YJR086W	-0.533215	0.084641	0.1900089	11.16	7.67	101.75	105.50
ORF-T	YJR145C	-0.533175	0.0835771	0.1883167	1285.22	888.13	11694.75	11435.00
ORF-T	YML020W	-0.532847	0.0674314	0.1604479	19.24	13.28	175.00	174.00
ORF-T	YNL081C	-0.532358	0.1139521	0.2361958	10.87	7.48	92.50	95.25
sn/snoRNA	SNR8	-0.532345	0.1421594	0.2784878	3397.54	2349.14	29023.25	29544.00
ORF-T	YJL180C	-0.532035	0.1033713	0.2209403	21.62	14.91	191.00	197.00
AST	AS_YIL021C-A	-0.531904	0.045031	0.1182225	28.86	19.98	267.00	256.75
ORF-T	YBR027C	-0.531455	0.161186	0.30342	35.67	24.67	317.50	312.00
ORF-T	YDL199C	-0.531363	0.129054	0.2593943	5.68	3.90	49.25	50.50
ORF-T	YGL078C	-0.531314	0.0248579	0.0733589	81.89	56.69	766.50	738.25
ORF-T	YML108W	-0.531228	0.0835884	0.1883167	25.00	17.29	226.00	227.25
ORF-T	YMR261C	-0.531172	0.1450035	0.2817371	81.23	56.18	706.25	717.75
ORF-T	YJR096W	-0.531058	0.165703	0.3093777	28.08	19.43	246.25	241.25
ORF-T	YHR098C	-0.530978	0.0449545	0.1180524	87.26	60.31	762.00	803.50
ORF-T	YDR470C	-0.530948	0.0843394	0.1896273	21.05	14.54	188.25	192.25
AST	AS_YKL096C-B	-0.530775	0.18868	0.3377309	3.06	2.08	27.00	28.25
SUT	SUT428	-0.530744	0.1188955	0.2433412	10.12	6.95	91.75	97.50
ORF-T	YPR141C	-0.530724	0.0400622	0.1081046	23.68	16.42	218.75	211.00
ORF-T	YLR229C	-0.53063	0.0370781	0.1016341	37.35	25.82	330.75	336.00
ORF-T	YGR192C	-0.530451	0.1824895	0.3306783	1309.39	906.49	11212.25	12112.75
SUT	SUT545	-0.529689	0.1779928	0.3248677	5.08	3.53	47.00	46.00
ORF-T	YBR070C	-0.529483	0.1080032	0.2277507	27.71	19.24	251.25	238.75
ORF-T	YDL192W	-0.529029	0.0463791	0.1210057	598.62	414.87	5421.25	5313.00

Class	Transcript name	RRP6 KO_vs_WT log2_fold _change	RRP6 KO_vs_WT p-value	RRP6 KO_vs_WT FDR	Ave Norm Reads in WT	Ave Norm Reads in RRP6	Average RAW read counts in WT	Average RAW read counts in RRP6
ORF-T	YGL004C	-0.528383	0.049333	0.1266158	24.82	17.22	229.75	222.00
AST	AS_YHR063W-A	-0.528367	0.0250177	0.073744	80.57	55.87	733.50	722.50
ORF-T	YJL062W-A	-0.528233	0.1212695	0.2473466	12.02	8.27	101.25	109.25
ORF-T	YJL181W	-0.528109	0.0463424	0.1209413	33.70	23.42	315.00	298.50
ORF-T	YBR208C	-0.52783	0.0766925	0.1766486	123.01	85.36	1153.50	1098.50
ORF-T	YLR192C	-0.527795	0.0247635	0.0731443	75.40	52.28	698.75	696.25
sn/snoRNA	SNR79	-0.527744	0.1497372	0.2877739	7.19	4.97	64.00	65.25
ORF-T	YHR051W	-0.527676	0.1659252	0.3096206	60.11	41.68	517.00	521.75
ORF-T	YJL079C	-0.527484	0.1345244	0.267495	27.34	18.95	228.50	236.50
ORF-T	YBL059C-A	-0.527298	0.1477439	0.2852479	5.60	3.86	47.00	48.75
AST	AS_YOL035C	-0.525984	0.1463199	0.2835299	4.18	2.91	36.75	36.75
ORF-T	YPR139C	-0.525918	0.0629314	0.1525115	27.75	19.25	246.50	246.50
ORF-T	YLR206W	-0.525799	0.0920419	0.2019914	45.29	31.39	391.25	417.75
ORF-T	YPL020C	-0.525639	0.0569756	0.1415808	25.98	18.01	233.00	238.00
ORF-T	YCR044C	-0.525347	0.0526715	0.1331509	41.28	28.68	367.25	362.25
ORF-T	YHL020C	-0.524974	0.0307329	0.0875207	80.61	56.09	756.25	712.75
ORF-T	YKR001C	-0.524243	0.046157	0.1205198	196.76	136.80	1779.50	1770.00
ORF-T	YBR026C	-0.524238	0.1767395	0.3231626	47.35	32.92	421.00	419.00
ORF-T	YGL186C	-0.524093	0.0391116	0.1062503	146.94	102.23	1361.25	1299.75
ORF-T	YNL247W	-0.523903	0.03106	0.0882032	184.18	128.10	1719.25	1691.75
ORF-T	YMR214W	-0.523791	0.0358654	0.0990597	82.47	57.40	763.00	737.25
ORF-T	YLR079W	-0.52364	0.1191252	0.2437619	35.76	24.84	326.75	334.00
ORF-T	YGL073W	-0.52342	0.051417	0.1307335	18.01	12.49	158.00	161.75
ORF-T	YER048C	-0.523068	0.0403561	0.1087231	72.90	50.73	656.25	648.25
ORF-T	YIL156W	-0.522633	0.0319551	0.0903378	43.16	30.01	381.50	388.00
ORF-T	YBR053C	-0.522498	0.2600324	0.422249	28.17	19.60	250.25	251.25
ORF-T	YDR497C	-0.522121	0.0562922	0.1404032	715.56	498.36	6838.25	6249.00
ORF-T	YKL063C	-0.52185	0.105464	0.2240579	7.10	4.92	64.50	65.75
ORF-T	YGL222C	-0.521826	0.1215966	0.2479636	5.54	3.82	48.25	50.25

TABLE S1: Differential expression data for RRP6 RNA-Seq dataset Page 56

Class	Transcript name	RRP6 KO_vs_WT log2_fold _change	RRP6 KO_vs_WT p-value	RRP6 KO_vs_WT FDR	Ave Norm Reads in WT	Ave Norm Reads in RRP6	Average RAW read counts in WT	Average RAW read counts in RRP6
ORF-T	YLR396C	-0.521665	0.0588502	0.1450256	20.72	14.37	188.25	197.25
ORF-T	YNL138W	-0.52139	0.137	0.2710601	131.02	91.27	1173.00	1181.25
ORF-T	YPL072W	-0.521365	0.1278933	0.257729	6.13	4.21	53.50	57.25
ORF-T	YDR040C	-0.520968	0.2404248	0.4000955	63.87	44.55	590.00	549.75
ORF-T	YPR195C	-0.520879	0.2407677	0.4005116	7.44	5.17	70.50	72.00
ORF-T	YOR020W-A	-0.520854	0.1126255	0.2341199	5.88	4.07	51.25	52.50
ORF-T	YHR019C	-0.520842	0.0577825	0.1429537	309.77	215.86	2753.00	2833.25
ORF-T	YDR201W	-0.520812	0.0745961	0.1729655	13.31	9.28	119.50	118.50
ORF-T	YMR237W	-0.520774	0.0773196	0.1774946	28.91	20.10	258.75	269.50
AST	AS_YPR099C	-0.520696	0.0921603	0.2022073	8.55	5.98	76.25	74.50
ORF-T	YDR144C	-0.520653	0.0509497	0.1297415	20.92	14.56	188.75	191.50
ORF-T	YLR361C-A	-0.520598	0.1077929	0.2276865	5.66	3.92	49.75	50.50
AST	AS_YLR258W	-0.520103	0.3033466	0.4693076	1.70	1.15	15.00	16.25
ORF-T	YNL264C	-0.51991	0.1160399	0.2392448	13.73	9.55	128.50	130.75
AST	AS_YJR071W	-0.519816	0.0891735	0.1971985	10.17	7.06	90.00	91.75
sn/snoRNA	SNR18	-0.519382	0.0851665	0.1909335	182.86	127.51	1531.50	1659.75
ORF-T	YDR388W	-0.519268	0.1088161	0.2289862	97.01	67.66	884.50	903.25
ORF-T	YNL175C	-0.518832	0.025586	0.0751774	47.62	33.24	432.25	429.50
ORF-T	YPL267W	-0.518612	0.2763408	0.440016	2.55	1.78	22.75	22.25
ORF-T	YHR086W	-0.518411	0.0732384	0.170366	18.76	13.08	165.50	167.50
NUT	NUT1428	-0.518277	0.1209106	0.2467643	792.33	553.22	7079.00	7032.75
ORF-T	YLR432W	-0.518028	0.0378437	0.103307	109.83	76.68	973.50	986.75
ORF-T	YNL129W	-0.517709	0.1433263	0.2797605	11.14	7.75	101.50	105.00
AST	AS_YHL002C-A	-0.51764	0.2041121	0.3556265	2.90	2.01	24.25	25.00
ORF-T	YBR021W	-0.517556	0.1266128	0.2554549	10.11	7.08	91.75	88.25
ORF-T	YOR329C	-0.517462	0.0916838	0.2014248	19.50	13.60	166.25	171.00
ORF-T	YLR009W	-0.517403	0.0717065	0.1678088	24.59	17.17	227.25	229.50
NUT	NUT0270	-0.51732	0.0848014	0.1902841	813.86	568.63	7182.50	7117.75
ORF-T	YKL013C	-0.517302	0.0724713	0.1690494	46.65	32.56	409.25	419.25

Class	Transcript name	RRP6 KO_vs_WT log2_fold _change	RRP6 KO_vs_WT p-value	RRP6 KO_vs_WT FDR	Ave Norm Reads in WT	Ave Norm Reads in RRP6	Average RAW read counts in WT	Average RAW read counts in RRP6
sn/snoRNA	SNR47	-0.517254	0.1439372	0.280476	1937.50	1353.74	17165.00	16935.00
ORF-T	YFR030W	-0.517085	0.1481172	0.2857726	15.90	11.07	135.75	143.75
ORF-T	YIL037C	-0.516739	0.2481766	0.4090271	2.12	1.48	19.50	19.25
ORF-T	YOR202W	-0.516682	0.1198202	0.2448859	5.44	3.74	47.50	50.75
ORF-T	YOR307C	-0.51667	0.0482723	0.1246871	33.26	23.23	308.25	309.25
ORF-T	YHR112C	-0.516566	0.0380685	0.1038643	57.53	40.23	521.00	513.75
ORF-T	YHR140W	-0.51656	0.1116412	0.23289	636.14	444.67	5478.25	5583.25
ORF-T	YCR073C	-0.516364	0.0941677	0.2058503	75.35	52.66	729.25	735.25
AST	AS_YPL265W	-0.516038	0.2673245	0.4302793	5.07	3.51	48.25	51.50
ORF-T	YER008C	-0.515605	0.0633507	0.1530862	37.43	26.10	319.50	343.50
ORF-T	YDL106C	-0.515461	0.1462865	0.2835197	11.86	8.22	100.00	111.00
ORF-T	YOR144C	-0.515382	0.0873123	0.1942328	11.68	8.21	109.00	103.75
ORF-T	YKL054C	-0.515252	0.0794052	0.1811179	101.97	71.28	886.50	947.75
ORF-T	YOR043W	-0.515097	0.0377665	0.103144	107.87	75.45	1004.50	1024.75
ORF-T	YER114C	-0.515076	0.0268312	0.078153	48.53	33.92	442.25	452.75
ORF-T	YBR060C	-0.514786	0.0765449	0.1763489	13.29	9.26	119.50	123.75
ORF-T	YEL034W	-0.514786	0.0597195	0.146381	340.42	238.26	2982.50	2988.50
ORF-T	YMR164C	-0.514785	0.1000981	0.2156354	8.66	6.03	76.00	78.00
ORF-T	YDR256C	-0.514744	0.1433238	0.2797605	12.92	9.09	123.25	115.75
ORF-T	YOL092W	-0.514641	0.0413628	0.1106634	33.64	23.49	296.75	309.50
AST	AS_YMR075C-A	-0.514622	0.2125994	0.3661657	2.80	1.97	25.75	25.75
ORF-T	YMR286W	-0.514503	0.1100186	0.2303151	15.99	11.14	137.50	145.75
ORF-T	YJR016C	-0.514394	0.0562382	0.1404032	743.74	520.64	6670.00	6903.50
ORF-T	YDL064W	-0.5141	0.0508556	0.1295674	25.07	17.55	227.25	229.75
ORF-T	YGR102C	-0.513852	0.102539	0.2196734	8.37	5.87	77.00	76.50
ORF-T	YBR155W	-0.513777	0.0609035	0.1485609	22.79	15.95	211.75	213.50
ORF-T	YIL114C	-0.513548	0.1012901	0.2174599	46.01	32.22	418.50	417.00
ORF-T	YML123C	-0.513397	0.1612136	0.30342	209.85	147.01	1890.50	1919.00
AST	AS_YJL188C	-0.513272	0.0804296	0.1829579	294.27	206.14	2582.25	2674.75

Class	Transcript name	RRP6 KO_vs_WT log2_fold _change	RRP6 KO_vs_WT p-value	RRP6 KO_vs_WT FDR	Ave Norm Reads in WT	Ave Norm Reads in RRP6	Average RAW read counts in WT	Average RAW read counts in RRP6
ORF-T	YKR095W	-0.512948	0.080879	0.1837718	90.11	63.11	794.00	822.75
ORF-T	YJR122W	-0.512896	0.2087014	0.3611093	12.26	8.55	106.75	114.25
AST	AS_YNL019C	-0.512799	0.2604552	0.4226634	1.54	1.07	13.75	14.25
ORF-T	YDL066W	-0.512446	0.1533357	0.2926817	252.97	177.32	2306.00	2343.25
ORF-T	YCR071C	-0.512257	0.1506314	0.288832	4.24	2.94	38.50	40.00
AST	AS_YLR076C	-0.512226	0.0604909	0.1478914	3006.90	2108.26	27627.25	27367.00
ORF-T	YLR075W	-0.512194	0.0608524	0.1484723	4104.94	2878.21	37685.75	37372.50
ORF-T	YPL277C	-0.512168	0.1945461	0.3445172	1.84	1.26	16.25	17.00
AST	AS_YOR277C	-0.511963	0.0310087	0.0880823	98.58	69.14	892.25	888.00
ORF-T	YLR094C	-0.511898	0.1028711	0.2202447	7.88	5.49	69.25	71.75
ORF-T	YMR318C	-0.511817	0.0613531	0.1494047	397.74	278.99	3633.75	3505.50
AST	AS_YEL034C-A	-0.511656	0.0596341	0.1462071	321.95	225.82	2817.50	2836.50
AST	AS_YLR434C	-0.511619	0.1264285	0.255134	3.10	2.19	29.25	29.00
ORF-T	YJR022W	-0.511597	0.239655	0.3990809	2.76	1.92	23.00	23.75
ORF-T	YOR239W	-0.511556	0.0967193	0.2101071	74.98	52.57	723.75	737.75
ORF-T	YNL225C	-0.511446	0.0423259	0.1127187	34.93	24.53	325.50	318.25
ORF-T	YGR055W	-0.511377	0.2727692	0.435981	126.32	88.62	1106.00	1084.25
ORF-T	YEL001C	-0.511345	0.0413635	0.1106634	141.46	99.24	1294.25	1288.75
ORF-T	YPR018W	-0.511031	0.0705784	0.1660939	16.51	11.62	157.00	151.00
ORF-T	YJL123C	-0.510842	0.0529794	0.1336613	75.98	53.35	695.75	674.25
ORF-T	YMR140W	-0.51033	0.1293729	0.2599833	24.98	17.49	211.00	227.25
ORF-T	YOR021C	-0.510114	0.0293926	0.0844431	73.29	51.50	678.00	665.50
AST	AS_YCR034W	-0.509915	0.3486663	0.5134175	1.14	0.80	10.50	10.50
ORF-T	YLR085C	-0.509885	0.0425557	0.1131377	30.10	21.12	276.75	280.50
AST	AS_YAL016C-B	-0.509722	0.1948624	0.3447356	8.18	5.75	72.00	72.25
ORF-T	YOL032W	-0.509681	0.1759511	0.3222786	14.20	9.94	123.00	128.75
ORF-T	YMR183C	-0.509634	0.1095448	0.2299677	39.13	27.43	346.50	364.75
AST	AS_YKL156C-A	-0.509622	0.1053122	0.2238551	19.24	13.45	170.25	181.00
ORF-T	YBL009W	-0.509566	0.0595212	0.1460724	20.94	14.67	191.25	196.25

Class	Transcript name	RRP6 KO_vs_WT log2_fold _change	RRP6 KO_vs_WT p-value	RRP6 KO_vs_WT FDR	Ave Norm Reads in WT	Ave Norm Reads in RRP6	Average RAW read counts in WT	Average RAW read counts in RRP6
ORF-T	YKL162C	-0.509542	0.3034508	0.4693248	2.69	1.87	23.25	24.50
ORF-T	YGR238C	-0.50949	0.0897839	0.1981574	7.97	5.56	71.50	74.25
ORF-T	YHR013C	-0.509353	0.0579101	0.143199	20.23	14.16	178.25	186.25
ORF-T	YLR285C-A	-0.509326	0.310372	0.4757228	4.14	2.87	34.00	37.00
ORF-T	YLR044C	-0.509003	0.1634116	0.3061766	3169.87	2227.45	26933.50	28831.25
ORF-T	YPL199C	-0.508738	0.09894	0.2137341	15.00	10.48	133.25	141.00
ORF-T	YJR013W	-0.508577	0.0398038	0.1076666	27.99	19.65	254.50	260.25
ORF-T	YJL063C	-0.508318	0.0518199	0.1314458	31.67	22.26	283.75	284.75
ORF-T	YPR053C	-0.507883	0.1071274	0.2267099	20.68	14.48	180.75	194.00
ORF-T	YEL057C	-0.507786	0.1958964	0.3458513	2.18	1.52	20.00	20.25
ORF-T	YDL040C	-0.507642	0.0659752	0.1579895	24.70	17.34	224.75	231.25
AST	AS_YKR012C	-0.507332	0.1561685	0.296573	6.81	4.73	58.75	64.00
ORF-T	YLL002W	-0.507024	0.0809344	0.1837744	14.54	10.23	138.25	137.75
ORF-T	YCR086W	-0.507017	0.2758687	0.4396113	1.63	1.13	14.75	15.25
ORF-T	YER089C	-0.506822	0.033222	0.0931101	87.93	61.82	778.25	818.75
ORF-T	YBR221C	-0.506804	0.110504	0.2311872	91.61	64.42	807.25	855.75
ORF-T	YHR087W	-0.506785	0.4042533	0.5631122	10.22	7.17	79.50	88.25
ORF-T	YLR031W	-0.506739	0.1991591	0.3498816	2.34	1.65	21.50	21.50
ORF-T	YOR111W	-0.506733	0.0462246	0.1206651	18.54	13.05	169.75	169.75
ORF-T	YLR059C	-0.506613	0.1448847	0.2816148	5.42	3.79	47.00	49.00
ORF-T	YEL031W	-0.506594	0.0430752	0.1141278	196.29	138.13	1737.00	1793.50
ORF-T	YDR318W	-0.50652	0.0606673	0.1482358	13.94	9.78	126.75	130.50
ORF-T	YGR089W	-0.50619	0.0662511	0.158387	20.32	14.26	180.00	188.50
ORF-T	YNL173C	-0.506152	0.1813433	0.3293706	32.14	22.62	282.50	287.00
ORF-T	YNR047W	-0.50601	0.044733	0.1175318	85.37	60.05	751.25	792.25
ORF-T	YJL176C	-0.505947	0.0389775	0.1059431	57.53	40.47	527.25	541.50
ORF-T	YGR185C	-0.505896	0.0385373	0.1049446	50.47	35.49	444.00	463.50
sn/snoRNA	SNR83	-0.505713	0.0957942	0.2085468	1478.39	1041.27	13381.75	13181.75
ORF-T	YDL240W	-0.505319	0.0571818	0.1418851	49.33	34.70	444.75	464.75

TABLE S1: Differential expression data for RRP6 RNA-Seq dataset Page 60

Class	Transcript name	RRP6 KO_vs_WT log2_fold_change	RRP6 KO_vs_WT p-value	RRP6 KO_vs_WT FDR	Ave Norm Reads in WT	Ave Norm Reads in RRP6	Average RAW read counts in WT	Average RAW read counts in RRP6
ORF-T	YPR030W	-0.505317	0.3425137	0.5071818	17.74	12.48	145.75	157.75
ORF-T	YPR062W	-0.505235	0.0379801	0.1036514	48.56	34.15	433.00	454.25
ORF-T	YDR069C	-0.505199	0.1224872	0.2491751	14.11	9.93	120.75	123.50
ORF-T	YKL084W	-0.505195	0.2515675	0.412558	2.02	1.41	17.25	17.75
ORF-T	YJL189W	-0.505149	0.0831211	0.1876797	304.47	214.49	2673.25	2783.75
ORF-T	YEL064C	-0.505008	0.06613	0.1582407	44.77	31.59	411.75	396.75
ORF-T	YNL091W	-0.504767	0.0490254	0.1260511	61.81	43.49	550.25	583.50
ORF-T	YER016W	-0.504674	0.0372667	0.102008	56.27	39.69	524.50	510.50
ORF-T	YPR019W	-0.504559	0.0621858	0.1509948	46.16	32.47	417.50	442.00
ORF-T	YHR204W	-0.504321	0.0566619	0.1411509	28.47	20.02	255.75	268.50
ORF-T	YLR141W	-0.504202	0.0713135	0.1671995	17.15	12.11	162.50	158.75
NUT	NUT0737	-0.504172	0.1524403	0.2913586	3560.76	2510.55	30607.75	31673.00
AST	AS_YJL182C	-0.50405	0.1462395	0.2834831	3.80	2.68	35.50	35.75
ORF-T	YFR015C	-0.50392	0.4083279	0.5663587	207.30	146.17	1659.00	1753.75
ORF-T	YPR084W	-0.503871	0.095447	0.2078808	13.78	9.63	118.25	130.00
AST	AS_YJL175W	-0.503861	0.0888621	0.1967689	11.48	8.06	103.50	107.50
ORF-T	YLR089C	-0.503807	0.0947143	0.2066866	28.90	20.33	253.50	271.25
ORF-T	YOL004W	-0.503657	0.0410347	0.1101687	131.28	92.58	1185.25	1207.00
SUT	SUT322	-0.50359	0.2566435	0.4181881	4.34	3.06	38.00	38.25
ORF-T	YMR230W	-0.503579	0.0968439	0.2101888	178.99	126.24	1569.75	1598.75
ORF-T	YJR099W	-0.503517	0.1439922	0.2805139	14.74	10.36	134.75	140.25
NUT	NUT0493	-0.503404	0.2358084	0.3947363	2.08	1.46	19.00	19.25
ORF-T	YIL159W	-0.503348	0.0911733	0.2005872	15.18	10.72	139.25	138.75
ORF-T	YKR013W	-0.503132	0.0528524	0.133441	82.92	58.45	734.00	776.25
AST	AS_YLR198C	-0.502876	0.0558548	0.1395886	113.54	80.18	1030.75	1004.25
ORF-T	YMR163C	-0.50275	0.049261	0.1264955	17.58	12.39	160.25	163.00
NUT	NUT1138	-0.50271	0.2127994	0.3663768	5.38	3.77	45.00	47.75
ORF-T	YOL149W	-0.502576	0.0522722	0.1323075	31.03	21.87	281.50	289.75
ORF-T	YML070W	-0.501636	0.1217935	0.2482648	140.25	99.07	1263.50	1251.75

Class	Transcript name	RRP6 KO_vs_WT log2_fold _change	RRP6 KO_vs_WT p-value	RRP6 KO_vs_WT FDR	Ave Norm Reads in WT	Ave Norm Reads in RRP6	Average RAW read counts in WT	Average RAW read counts in RRP6
ORF-T	YHR163W	-0.501568	0.1616117	0.3038204	45.89	32.37	413.00	439.75
ORF-T	YIL116W	-0.501358	0.0484214	0.1249762	64.01	45.22	601.25	602.00
ORF-T	YPR061C	-0.501334	0.1710514	0.3156816	4.03	2.84	34.25	35.25
ORF-T	YDR307W	-0.501326	0.0656529	0.1573298	23.75	16.74	209.50	217.00
AST	AS_YNR042W	-0.501293	0.0423548	0.1127523	33.74	23.87	317.25	313.25
ORF-T	YNL273W	-0.50122	0.0569889	0.1415808	48.15	34.03	436.25	434.00
ORF-T	YGL193C	-0.500704	0.2383069	0.3977962	2.53	1.78	21.25	21.75
ORF-T	YHR154W	-0.500679	0.0506828	0.1291924	66.66	47.17	620.25	596.00
ORF-T	YOL073C	-0.500244	0.0440967	0.1163452	42.85	30.30	390.75	390.75
ORF-T	YKR092C	-0.500112	0.0863808	0.1927133	18.70	13.20	170.50	174.50
ORF-T	YDL173W	-0.500098	0.0468087	0.1218115	29.70	20.95	261.75	273.00
ORF-T	YDR400W	-0.500049	0.0648211	0.1557436	18.04	12.76	161.50	163.75
ORF-T	YIL010W	-0.500017	0.1147048	0.2376095	12.63	8.89	112.00	118.00
ORF-T	YPL169C	-0.499825	0.0864277	0.1927752	24.31	17.12	207.00	223.75
AST	AS_YLR230W	-0.499779	0.0666376	0.159047	20.65	14.56	181.75	190.50
ORF-T	YGL049C	-0.499742	0.0397519	0.1076235	106.34	75.19	975.50	997.25
ORF-T	YDR165W	-0.499471	0.0714166	0.1674023	23.01	16.21	202.00	216.00
ORF-T	YBR222C	-0.499167	0.1621199	0.3046055	29.58	20.92	256.50	263.50
ORF-T	YER068W	-0.499118	0.1281268	0.2580523	11.86	8.33	101.00	110.25
ORF-T	YDR475C	-0.498934	0.075135	0.1739751	56.18	39.80	513.00	494.25
ORF-T	YER080W	-0.49886	0.1242938	0.2520873	38.49	27.19	337.50	359.50
ORF-T	YMR304W	-0.49822	0.1346386	0.2676317	81.44	57.63	728.75	755.50
ORF-T	YGR041W	-0.498053	0.1257881	0.2542488	9.60	6.76	86.75	91.50
ORF-T	YBR261C	-0.497951	0.0351872	0.0975339	36.14	25.58	326.50	334.00
ORF-T	YLR450W	-0.497837	0.0370797	0.1016341	72.19	51.10	642.00	662.75
ORF-T	YNR037C	-0.497789	0.0784882	0.1793913	16.09	11.42	145.00	142.75
ORF-T	YCL045C	-0.497312	0.0946892	0.2066767	45.74	32.34	394.25	425.25
ORF-T	YOR347C	-0.497297	0.2010826	0.3520954	45.51	32.23	397.25	410.00
AST	AS_YDR402C	-0.497086	0.2091406	0.361621	3.16	2.22	29.00	30.00

TABLE S1: Differential expression data for RRP6 RNA-Seq dataset Page 62

Class	Transcript name	RRP6 KO_vs_WT log2_fold_change	RRP6 KO_vs_WT p-value	RRP6 KO_vs_WT FDR	Ave Norm Reads in WT	Ave Norm Reads in RRP6	Average RAW read counts in WT	Average RAW read counts in RRP6
ORF-T	YJL208C	-0.497077	0.0773842	0.1775522	22.04	15.58	202.25	210.75
CUT	CUT544	-0.496941	0.0803629	0.1828474	20.10	14.27	186.00	184.00
ORF-T	YLR324W	-0.49653	0.1029891	0.2203865	41.43	29.35	361.75	373.25
ORF-T	YIL048W	-0.496455	0.0507044	0.1292147	44.07	31.17	386.75	414.25
ORF-T	YOR156C	-0.496294	0.0700124	0.1651087	14.18	10.00	126.75	133.50
ORF-T	YNL135C	-0.496266	0.0518665	0.1314536	84.12	59.62	759.50	771.50
AST	AS_YKL171W	-0.495996	0.1785535	0.3255938	7.40	5.24	72.00	73.50
ORF-T	YLR438C-A	-0.495907	0.0637331	0.153861	33.69	23.89	307.75	308.50
ORF-T	YCR045C	-0.49544	0.1894008	0.3385246	3.50	2.45	30.50	32.25
NUT	NUT1237	-0.495382	0.1006504	0.2164552	3298.23	2339.67	28619.25	29276.25
ORF-T	YNL160W	-0.495323	0.4114975	0.5686639	14.10	9.97	107.00	126.25
ORF-T	YGL258W	-0.495216	0.2792673	0.4432116	1.60	1.12	13.75	14.25
ORF-T	YJL187C	-0.494945	0.0647566	0.1556453	24.96	17.67	221.25	232.50
ORF-T	YGR285C	-0.49489	0.1082468	0.2280261	82.72	58.63	755.00	824.00
ORF-T	YKR059W	-0.49481	0.0695233	0.1641856	398.12	282.54	3611.25	3594.00
ORF-T	YMR315W	-0.494491	0.2918836	0.4560511	22.35	15.85	186.25	195.75
ORF-T	YCL014W	-0.493337	0.0797494	0.1816154	51.40	36.43	454.75	499.75
ORF-T	YNL095C	-0.493095	0.1060086	0.2250032	21.87	15.51	202.75	210.75
ORF-T	YEL042W	-0.492762	0.0397951	0.1076666	98.48	69.99	884.50	897.25
ORF-T	YMR205C	-0.492733	0.1030029	0.2203865	310.75	220.78	2685.25	2908.75
ORF-T	YNL304W	-0.492376	0.1306826	0.2614909	5.91	4.17	51.00	53.50
ORF-T	YNL024C-A	-0.492241	0.066083	0.1581726	54.56	38.79	488.00	494.75
NUT	NUT0605	-0.492064	0.1045792	0.2228606	1480.76	1052.86	13403.00	13335.00
ORF-T	YGR082W	-0.491849	0.0873637	0.1943043	22.58	15.99	195.50	211.75
ORF-T	YDL016C	-0.491751	0.1971339	0.3475347	26.92	19.10	234.00	256.50
AST	AS_YCL022C	-0.491143	0.1887261	0.3377309	6.58	4.71	60.50	59.00
ORF-T	YDR353W	-0.491111	0.1179278	0.2418024	221.92	157.89	1982.50	1990.00
ORF-T	YIL045W	-0.490789	0.1448135	0.2815307	15.47	10.98	132.50	140.25
SUT	SUT338	-0.490748	0.104305	0.2224173	11.71	8.33	105.25	106.75

Class	Transcript name	RRP6 KO_vs_WT log2_fold _change	RRP6 KO_vs_WT p-value	RRP6 KO_vs_WT FDR	Ave Norm Reads in WT	Ave Norm Reads in RRP6	Average RAW read counts in WT	Average RAW read counts in RRP6
ORF-T	YHR005C-A	-0.490728	0.1295763	0.2601003	61.37	43.66	528.75	544.25
ORF-T	YGR231C	-0.490466	0.0858562	0.1918401	74.15	52.78	671.50	678.25
AST	AS_YLR285C-A	-0.490414	0.1089317	0.2290859	7.86	5.57	71.75	75.00
ORF-T	YDR376W	-0.49027	0.0729844	0.1699096	60.97	43.43	582.25	577.25
AST	AS_YPL089C	-0.49016	0.3866901	0.5476662	5.31	3.75	51.00	55.75
ORF-T	YLR193C	-0.48999	0.142371	0.2784878	7.14	5.04	62.50	67.25
ORF-T	YHR102W	-0.489837	0.0490974	0.126204	37.07	26.35	335.75	354.50
CUT	CUT413	-0.489551	0.233164	0.3915447	3.33	2.35	29.50	31.00
ORF-T	YKR067W	-0.489421	0.1423922	0.2784878	35.09	25.00	302.50	305.75
ORF-T	YOL105C	-0.489412	0.095107	0.2072296	12.75	9.04	111.50	119.50
ORF-T	YDR377W	-0.488929	0.1436982	0.2803351	104.16	74.21	900.25	920.50
ORF-T	YNL285W	-0.488748	0.2332425	0.3916112	2.10	1.52	19.00	19.00
ORF-T	YPL066W	-0.488684	0.1928929	0.3424876	6.03	4.25	50.25	55.25
ORF-T	YDL072C	-0.488653	0.1311515	0.262303	35.21	25.07	304.75	319.00
ORF-T	YGL126W	-0.488491	0.0529335	0.1335788	91.35	65.16	828.75	816.00
ORF-T	YNR010W	-0.488438	0.2231799	0.3793907	1.98	1.43	18.00	17.75
ORF-T	YDR127W	-0.488319	0.0651744	0.1563689	337.85	240.80	3121.00	3255.75
ORF-T	YDR107C	-0.488253	0.0607753	0.1483918	55.68	39.68	501.00	514.50
ORF-T	YLR385C	-0.487698	0.1161673	0.2394095	6.15	4.38	56.00	57.25
AST	AS_YPR136C	-0.487525	0.1926588	0.3423989	6.36	4.52	59.75	61.50
ORF-T	YPL128C	-0.487492	0.0855382	0.1913925	20.22	14.39	183.75	192.50
ORF-T	YJR133W	-0.487456	0.0441571	0.1164472	35.22	25.12	324.50	328.50
ORF-T	YNL036W	-0.487454	0.2006894	0.3517733	101.08	72.10	909.75	935.25
ORF-T	YGL028C	-0.487344	0.2083516	0.3606541	37.22	26.51	328.50	353.25
sn/snoRNA	SNR60	-0.487155	0.2186275	0.3744293	180.95	129.03	1458.75	1688.25
ORF-T	YDR518W	-0.487023	0.0894926	0.1978175	22.78	16.20	198.00	211.50
ORF-T	YNR018W	-0.486905	0.0694257	0.164032	34.15	24.32	305.75	322.75
ORF-T	YHR158C	-0.486893	0.0961331	0.2090138	36.00	25.63	315.75	339.50
ORF-T	YNR016C	-0.486448	0.1622143	0.3046692	457.78	326.77	4138.00	4139.50

Class	Transcript name	RRP6 KO_vs_WT log2_fold _change	RRP6 KO_vs_WT p-value	RRP6 KO_vs_WT FDR	Ave Norm Reads in WT	Ave Norm Reads in RRP6	Average RAW read counts in WT	Average RAW read counts in RRP6
ORF-T	YER010C	-0.486316	0.0919931	0.2019283	18.95	13.47	161.50	174.50
ORF-T	YPL222W	-0.485938	0.0682127	0.1618105	41.76	29.85	391.00	388.00
ORF-T	YDL067C	-0.48583	0.0748007	0.1732807	48.67	34.74	440.75	449.50
ORF-T	YGR234W	-0.485786	0.3586077	0.522558	486.82	347.62	4374.25	4694.00
ORF-T	YKR004C	-0.485751	0.1959412	0.3458513	3.28	2.29	28.25	30.75
ORF-T	YPL006W	-0.4856	0.0588784	0.1450597	66.47	47.46	592.75	609.75
ORF-T	YAR008W	-0.485511	0.1078382	0.2276865	17.11	12.26	159.75	156.50
ORF-T	YOR276W	-0.485507	0.0487191	0.1255269	131.83	94.17	1196.00	1203.25
ORF-T	YAL030W	-0.485128	0.220037	0.3759494	4.97	3.48	41.75	48.25
ORF-T	YFR017C	-0.48463	0.1918668	0.3413523	8.68	6.16	72.75	79.00
ORF-T	YHR009C	-0.484082	0.0426043	0.1132074	92.69	66.26	837.25	856.25
ORF-T	YMR043W	-0.484032	0.1268307	0.2557921	21.72	15.44	186.50	209.25
ORF-T	YDR503C	-0.484016	0.1046506	0.2229658	39.40	28.21	354.00	347.75
ORF-T	YNL106C	-0.483942	0.0834988	0.1882411	37.62	26.87	341.25	358.00
ORF-T	YPR125W	-0.483875	0.1106508	0.2313984	22.72	16.21	207.00	218.25
ORF-T	YMR236W	-0.483742	0.0517085	0.13121	42.35	30.26	385.50	400.50
ORF-T	YNR046W	-0.483456	0.0864919	0.1928044	12.68	9.03	115.75	121.75
ORF-T	YGR267C	-0.483398	0.0442251	0.1164705	124.95	89.39	1145.25	1155.50
ORF-T	YOR124C	-0.483255	0.1498791	0.2879369	144.79	103.55	1311.25	1376.75
ORF-T	YGR094W	-0.483219	0.0583986	0.1441596	822.42	588.33	7545.50	7746.25
ORF-T	YIL149C	-0.482941	0.0581312	0.14364	89.49	64.00	800.50	836.50
ORF-T	YLR244C	-0.482918	0.0763201	0.1759515	26.44	18.90	235.25	246.50
sn/snoRNA ET	SNR52-ET*	-0.482894	0.1789033	0.3259368	4946.56	3539.48	43694.00	44165.50
ORF-T	YDR037W	-0.482709	0.0745563	0.1729129	459.62	328.89	4198.00	4383.75
ORF-T	YLR364W	-0.481829	0.2071187	0.3594209	4.17	2.97	38.50	39.50
ORF-T	YLR383W	-0.481656	0.0896537	0.1980264	22.50	16.16	208.00	204.25
ORF-T	YBR139W	-0.481278	0.2239906	0.3805764	39.41	28.18	334.50	375.00
ORF-T	YOL086C	-0.481264	0.1078598	0.2276865	2723.94	1951.29	24403.75	24877.50
ORF-T	YBR233W-A	-0.481069	0.084771	0.1902583	12.02	8.65	110.50	109.00

Class	Transcript name	RRP6 KO_vs_WT log2_fold _change	RRP6 KO_vs_WT p-value	RRP6 KO_vs_WT FDR	Ave Norm Reads in WT	Ave Norm Reads in RRP6	Average RAW read counts in WT	Average RAW read counts in RRP6
ORF-T	YIL126W	-0.480961	0.0796063	0.1813714	67.48	48.34	617.50	638.25
ORF-T	YNL262W	-0.480756	0.0394805	0.1070219	76.25	54.64	704.75	719.75
ORF-T	YPR114W	-0.480404	0.1738353	0.3194154	10.44	7.43	88.50	98.25
ORF-T	YOR194C	-0.480288	0.0361427	0.0995802	43.89	31.47	404.25	409.50
ORF-T	YPL031C	-0.479671	0.0609574	0.1485967	46.39	33.24	418.25	432.25
ORF-T	YLR210W	-0.479334	0.0858539	0.1918401	12.62	9.08	115.75	116.25
ORF-T	YLR196W	-0.47909	0.0584455	0.1442401	155.19	111.36	1470.50	1481.00
ORF-T	YDR070C	-0.47885	0.2546522	0.4163282	3.54	2.51	28.75	31.50
ORF-T	YIL070C	-0.478519	0.1121003	0.2335577	23.20	16.60	203.75	218.50
ORF-T	YLR351C	-0.478184	0.1109631	0.2319071	128.58	92.30	1164.50	1187.00
ORF-T	YLR228C	-0.477877	0.0525319	0.1328885	39.54	28.37	360.00	372.25
ORF-T	YKL101W	-0.477742	0.0474139	0.1229105	93.69	67.27	856.00	886.25
ORF-T	YHL031C	-0.477465	0.166551	0.3102143	6.19	4.41	53.25	57.50
ORF-T	YGR167W	-0.477456	0.1433481	0.2797605	42.49	30.49	374.00	393.25
ORF-T	YOR091W	-0.477278	0.0480125	0.1241092	43.96	31.52	399.50	424.50
ORF-T	YNL272C	-0.477198	0.0918852	0.2017792	17.77	12.74	158.00	164.75
ORF-T	YNL084C	-0.477198	0.0709301	0.166766	43.49	31.22	393.00	409.75
ORF-T	YKL165C	-0.477185	0.0763768	0.176042	87.10	62.60	797.50	794.75
ORF-T	YOL014W	-0.476928	0.2275525	0.3844261	11.78	8.43	103.75	113.50
ORF-T	YJR050W	-0.476512	0.071107	0.1670261	14.86	10.67	134.00	138.00
AST	AS_YER147C-A	-0.476492	0.0468032	0.1218115	59.48	42.80	558.00	552.50
ORF-T	YAR007C	-0.476248	0.0716651	0.167751	91.71	65.97	833.75	826.75
AST	AS_YFR036W-A	-0.476243	0.0635909	0.1535929	51.64	37.14	486.00	491.75
ORF-T	YHR091C	-0.476104	0.0630473	0.152656	37.78	27.18	355.75	358.25
ORF-T	YMR003W	-0.475746	0.1343174	0.2672566	12.63	9.11	113.00	113.25
ORF-T	YLL025W	-0.475524	0.1539195	0.2935737	3.27	2.34	29.50	30.75
ORF-T	YNL290W	-0.475491	0.0426305	0.1132448	64.18	46.18	599.75	604.50
ORF-T	YEL002C	-0.475249	0.0621813	0.1509948	100.80	72.48	898.25	938.75
ORF-T	YMR012W	-0.4752	0.1446842	0.2814421	225.47	162.15	1982.50	2119.50

Class	Transcript name	RRP6 KO_vs_WT log2_fold _change	RRP6 KO_vs_WT p-value	RRP6 KO_vs_WT FDR	Ave Norm Reads in WT	Ave Norm Reads in RRP6	Average RAW read counts in WT	Average RAW read counts in RRP6
ORF-T	YHL002W	-0.475106	0.1622047	0.3046692	7.22	5.19	63.50	65.00
ORF-T	YLR080W	-0.474899	0.1974581	0.3479237	7.15	5.15	63.00	64.00
ORF-T	YGL051W	-0.47473	0.2995491	0.4643587	1.24	0.92	12.50	12.25
ORF-T	YMR260C	-0.474593	0.0503256	0.1284443	92.31	66.38	834.00	885.25
AST	AS_YAL034C-B	-0.474449	0.0902492	0.1988362	12.82	9.26	121.00	121.00
ORF-T	YMR148W	-0.474136	0.1371483	0.2712303	8.86	6.33	75.25	82.00
ORF-T	YDR368W	-0.474103	0.2270891	0.3837719	40.02	28.77	349.50	381.50
ORF-T	YPL013C	-0.473576	0.0915545	0.2012284	15.79	11.36	141.25	146.00
ORF-T	YEL052W	-0.473473	0.0575354	0.1424471	67.67	48.75	627.50	633.75
ORF-T	YHR089C	-0.473453	0.07359	0.1711047	295.92	213.18	2741.75	2706.50
ORF-T	YIL008W	-0.47343	0.109458	0.2299051	22.11	15.95	203.75	203.00
AST	AS_YEL018C-A	-0.473415	0.1179228	0.2418024	9.26	6.64	85.00	89.50
ORF-T	YOR327C	-0.47335	0.1003083	0.2159207	23.99	17.27	209.00	217.75
ORF-T	YER161C	-0.473098	0.0603078	0.1476436	23.59	16.96	210.75	223.25
ORF-T	YKL039W	-0.472753	0.0668542	0.1594131	115.76	83.38	1034.00	1090.00
ORF-T	YMR197C	-0.472697	0.1168511	0.2402297	16.68	11.97	144.75	156.25
ORF-T	YJR106W	-0.472687	0.0829087	0.1872456	16.53	11.87	147.00	157.00
ORF-T	YGL187C	-0.472261	0.2654604	0.4283729	121.37	87.48	1028.00	1076.75
ORF-T	YLR069C	-0.472171	0.1295859	0.2601003	120.65	87.00	1131.50	1116.25
ORF-T	YIL063C	-0.472159	0.0611472	0.1489751	49.34	35.57	451.00	458.00
ORF-T	YDR226W	-0.472035	0.0941087	0.2057659	422.71	304.74	3863.50	3973.00
NUT	NUT1080	-0.471749	0.2512051	0.4123025	3.96	2.86	38.00	39.25
ORF-T	YFR031C	-0.471445	0.0590851	0.1453205	48.49	34.96	451.50	467.50
AST	AS_YKR040C	-0.471415	0.284509	0.4480147	1.40	1.01	12.75	13.25
ORF-T	YEL017W	-0.471407	0.1187397	0.2431211	10.15	7.28	91.25	97.00
ORF-T	YLR248W	-0.47129	0.0747306	0.1731579	405.92	292.84	3691.50	3674.50
ORF-T	YDR517W	-0.471233	0.0715707	0.1676466	119.45	86.18	1081.75	1094.25
ORF-T	YHR175W	-0.471195	0.0977137	0.2115837	22.33	16.06	193.25	208.00
ORF-T	YGR263C	-0.471194	0.0603887	0.1477339	19.77	14.27	183.50	186.50

Class	Transcript name	RRP6 KO_vs_WT log2_fold _change	RRP6 KO_vs_WT p-value	RRP6 KO_vs_WT FDR	Ave Norm Reads in WT	Ave Norm Reads in RRP6	Average RAW read counts in WT	Average RAW read counts in RRP6
ORF-T	YBL034C	-0.471069	0.1232757	0.2504255	10.76	7.73	94.00	100.25
ORF-T	YLR052W	-0.470955	0.1006985	0.2164749	11.13	8.01	103.00	107.25
ORF-T	YML001W	-0.47083	0.07416	0.1722315	265.45	191.56	2411.50	2430.50
ORF-T	YLR412W	-0.470829	0.0762272	0.1758579	27.47	19.79	256.75	268.50
ORF-T	YJR049C	-0.4703	0.050325	0.1284443	75.08	54.24	696.50	696.00
ORF-T	YER088C-A	-0.469798	0.1390184	0.2735873	6.74	4.85	61.25	64.25
ORF-T	YGR213C	-0.46977	0.255808	0.4176761	3.50	2.53	34.00	35.00
ORF-T	YMR272C	-0.469189	0.1095826	0.2299677	62.52	45.09	539.00	594.00
ORF-T	YMR032W	-0.468195	0.204122	0.3556265	40.34	29.13	367.00	401.75
ORF-T	YJL203W	-0.468134	0.1426428	0.2788156	5.25	3.75	47.00	51.00
ORF-T	YPL220W	-0.468115	0.0691459	0.1635336	671.77	485.62	6043.00	6294.50
SUT	SUT309	-0.468114	0.1645504	0.3077506	7.23	5.24	69.25	70.50
ORF-T	YDR117C	-0.468097	0.0962207	0.2091592	19.99	14.40	185.75	198.75
ORF-T	YOR009W	-0.467991	0.3187532	0.483791	2.55	1.79	21.50	24.75
AST	AS_YOR102W	-0.467816	0.068825	0.1628799	106.65	77.10	974.50	1007.50
ORF-T	YLR190W	-0.467752	0.1999614	0.3509242	94.98	68.65	894.50	968.25
ORF-T	YMR048W	-0.46775	0.1229936	0.2500136	13.54	9.81	127.00	129.00
ORF-T	YHR132C	-0.46746	0.1066236	0.2261182	24.78	17.89	221.00	234.00
ORF-T	YHR188C	-0.467312	0.0609273	0.148583	46.20	33.37	403.75	431.50
AST	AS_YGR115C	-0.467305	0.0854373	0.19129	40.92	29.58	379.75	394.25
ORF-T	YBR127C	-0.467272	0.1779943	0.3248677	366.26	264.90	3333.25	3528.50
ORF-T	YGL191W	-0.466957	0.1242237	0.2520451	65.31	47.23	574.25	601.75
ORF-T	YKL041W	-0.466705	0.0878291	0.1951945	17.61	12.75	162.00	166.00
ORF-T	YER083C	-0.466557	0.0712599	0.1671517	17.81	12.86	162.25	170.25
ORF-T	YJR118C	-0.466015	0.088243	0.1957528	23.17	16.73	207.25	221.25
ORF-T	YDL100C	-0.46601	0.0812063	0.1841431	298.19	215.88	2713.25	2783.75
ORF-T	YJR043C	-0.465921	0.1306313	0.2614909	9.73	7.02	90.25	96.00
ORF-T	YBR227C	-0.465715	0.1070902	0.2267099	16.15	11.67	146.25	153.75
ORF-T	YGR193C	-0.465625	0.1077329	0.2276865	31.58	22.84	287.00	301.00

Class	Transcript name	RRP6 KO_vs_WT log2_fold _change	RRP6 KO_vs_WT p-value	RRP6 KO_vs_WT FDR	Ave Norm Reads in WT	Ave Norm Reads in RRP6	Average RAW read counts in WT	Average RAW read counts in RRP6
ORF-T	YNL082W	-0.465519	0.0929421	0.2035682	46.85	33.98	434.75	427.00
ORF-T	YDR155C	-0.465363	0.2383906	0.39787	407.18	294.86	3395.00	3830.75
ORF-T	YDR297W	-0.464429	0.0831678	0.1877047	71.69	51.91	623.25	671.00
ORF-T	YML100W	-0.463833	0.3785252	0.5400464	139.98	101.47	1101.75	1226.00
ORF-T	YMR257C	-0.463754	0.0573802	0.1422189	34.69	25.11	316.75	336.50
ORF-T	YER124C	-0.46374	0.1806541	0.3284146	163.57	118.56	1466.00	1596.00
ORF-T	YKR007W	-0.463657	0.0837534	0.18852	17.52	12.69	158.00	164.00
ORF-T	YDR374W-A	-0.463429	0.1077611	0.2276865	18.40	13.38	168.75	169.75
ORF-T	YLR355C	-0.463181	0.1088988	0.2290646	1124.05	815.36	10119.25	10517.50
ORF-T	YMR038C	-0.463071	0.0542626	0.1364208	48.52	35.18	445.00	466.00
ORF-T	YDL157C	-0.462529	0.0501362	0.1281229	57.02	41.43	521.75	522.50
ORF-T	YJR012C	-0.462285	0.1386486	0.2732863	5.10	3.66	44.75	48.50
ORF-T	YBR101C	-0.46226	0.0618401	0.1503727	76.00	55.17	692.50	708.00
AST	AS_YHR173C	-0.462166	0.1163148	0.2396312	6.91	5.04	65.50	66.50
ORF-T	YIL002W-A	-0.462033	0.1253613	0.2536404	10.56	7.66	92.50	97.25
ORF-T	YGR211W	-0.461931	0.0711727	0.1670706	163.19	118.44	1457.75	1553.25
ORF-T	YGL087C	-0.461874	0.1615871	0.3038204	7.33	5.32	63.50	66.50
SUT	SUT243	-0.46171	0.169503	0.3136271	4.40	3.15	39.00	42.50
ORF-T	YDL090C	-0.461686	0.1300912	0.2608031	50.61	36.75	457.00	470.75
ORF-T	YKL048C	-0.461445	0.0712088	0.1670706	24.62	17.87	227.00	237.25
ORF-T	YDR457W	-0.461414	0.0835711	0.1883167	136.90	99.38	1224.00	1325.00
ORF-T	YPR202W	-0.46117	0.2351089	0.3938924	3.11	2.25	28.00	29.50
ORF-T	YJR032W	-0.461118	0.1111634	0.2321813	11.25	8.14	103.25	109.25
ORF-T	YJL036W	-0.460975	0.0959219	0.2087796	45.31	32.90	406.00	425.00
ORF-T	YIR010W	-0.460939	0.1098806	0.2301294	15.02	10.86	136.50	148.50
ORF-T	YPL147W	-0.460844	0.1887612	0.3377338	19.85	14.45	184.50	186.50
ORF-T	YLR284C	-0.460751	0.1133118	0.2351587	14.13	10.28	128.75	132.00
ORF-T	YOL109W	-0.460443	0.1130803	0.2347265	489.74	355.93	4520.75	4634.25
AST	AS_YGR242W	-0.460351	0.2021299	0.3533769	3.97	2.85	34.25	37.00

Class	Transcript name	RRP6 KO_vs_WT log2_fold _change	RRP6 KO_vs_WT p-value	RRP6 KO_vs_WT FDR	Ave Norm Reads in WT	Ave Norm Reads in RRP6	Average RAW read counts in WT	Average RAW read counts in RRP6
SUT	SUT659	-0.46002	0.2131497	0.3667294	7.40	5.37	72.00	75.50
ORF-T	YKR008W	-0.46	0.1526035	0.2916153	11.86	8.58	102.75	111.00
ORF-T	YNR039C	-0.459986	0.1582791	0.299341	9.91	7.18	85.25	91.75
ORF-T	YDR128W	-0.459871	0.0693805	0.1640022	84.74	61.61	777.25	800.25
ORF-T	YCR014C	-0.459847	0.0871357	0.1939256	23.27	16.96	221.75	223.00
ORF-T	YDR217C	-0.459652	0.0796436	0.1814154	18.66	13.58	171.25	176.00
ORF-T	YBR182C	-0.459581	0.1755015	0.3216571	8.11	5.95	81.50	79.00
ORF-T	YDR092W	-0.459478	0.0722679	0.1686805	28.94	20.99	256.00	277.75
ORF-T	YFR052C-A	-0.459219	0.4209128	0.5762606	61.88	45.00	499.50	549.25
ORF-T	YBL071W-A	-0.458989	0.3107611	0.4761761	5.22	3.78	50.25	53.50
ORF-T	YBR129C	-0.458957	0.0642154	0.1545829	58.66	42.71	531.00	537.25
AST	AS_YBL089W	-0.458954	0.4194591	0.5752517	1.31	0.95	13.25	13.75
NUT	NUT0903	-0.458835	0.1932409	0.3428291	1949.05	1418.09	17271.75	17754.00
AST	AS_YKL036C	-0.45883	0.1687626	0.312862	48.72	35.45	430.75	446.25
ORF-T	YDR373W	-0.458819	0.0598046	0.1465183	74.23	54.01	660.50	686.75
ORF-T	YPL194W	-0.458741	0.1098291	0.2301095	14.49	10.55	138.25	141.00
ORF-T	YLR154C	-0.458063	0.2082358	0.3606467	3.26	2.38	29.75	30.25
ORF-T	YNL263C	-0.458042	0.0795308	0.1812589	64.67	47.01	584.75	637.75
ORF-T	YAL023C	-0.457972	0.1127708	0.234277	502.63	365.95	4562.00	4596.75
ORF-T	YIL144W	-0.45796	0.0798092	0.1817106	14.15	10.29	126.75	133.00
ORF-T	YPR081C	-0.457949	0.1679115	0.3117694	21.03	15.30	189.50	199.00
ORF-T	YLR314C	-0.457599	0.0651299	0.1563365	106.70	77.74	980.25	981.50
ORF-T	YFL024C	-0.457582	0.0518697	0.1314536	37.71	27.46	345.50	359.00
ORF-T	YGR100W	-0.457467	0.1175483	0.2412696	105.78	77.04	972.00	993.25
ORF-T	YIL041W	-0.457385	0.1028145	0.2201701	188.95	137.61	1730.50	1792.50
ORF-T	YIL039W	-0.457195	0.0591696	0.1454366	113.63	82.74	1031.75	1090.50
ORF-T	YLR025W	-0.456803	0.1420694	0.2784505	29.02	21.13	266.25	280.25
ORF-T	YLR437C	-0.456783	0.1388548	0.2733721	8.46	6.12	75.50	82.25
AST	AS_YPR106W	-0.456437	0.2168057	0.3717512	4.01	2.91	35.00	37.25

TABLE S1: Differential expression data for RRP6 RNA-Seq dataset Page 70

Class	Transcript name	RRP6 KO_vs_WT log2_fold _change	RRP6 KO_vs_WT p-value	RRP6 KO_vs_WT FDR	Ave Norm Reads in WT	Ave Norm Reads in RRP6	Average RAW read counts in WT	Average RAW read counts in RRP6
ORF-T	YGR194C	-0.456223	0.306296	0.4719879	37.82	27.56	325.25	340.75
ORF-T	YPL054W	-0.45612	0.5368418	0.671735	2.48	1.80	19.25	21.00
ORF-T	YBR275C	-0.45603	0.0585988	0.1445475	50.38	36.72	457.25	477.50
ORF-T	YDR499W	-0.455889	0.0677122	0.1609261	30.32	22.14	279.00	283.00
ORF-T	YCL057C-A	-0.455785	0.2215566	0.3777775	4.07	2.94	34.75	37.25
ORF-T	YOL151W	-0.455655	0.2856313	0.4488999	35.35	25.76	301.50	324.25
ORF-T	YAL002W	-0.455287	0.0900284	0.1984365	22.54	16.40	201.75	217.00
ORF-T	YDR414C	-0.455089	0.1872644	0.3363397	8.33	6.03	71.75	79.50
AST	AS_YAL056C-A	-0.455081	0.1161167	0.2393542	10.36	7.56	94.00	97.50
ORF-T	YOR103C	-0.455028	0.0758272	0.1752158	111.63	81.43	1023.00	1064.00
ORF-T	YLR084C	-0.454941	0.1094972	0.2299396	66.07	48.13	589.00	654.25
sn/snoRNA ET	SNR190-ET	-0.454893	0.2694534	0.4326012	1099.63	802.25	9545.75	10157.00
ORF-T	YNL312W	-0.454869	0.0580822	0.1435542	74.34	54.26	681.25	696.50
ORF-T	YNL077W	-0.454832	0.109676	0.2300276	43.15	31.50	392.25	398.00
ORF-T	YEL036C	-0.454763	0.0591251	0.1453835	88.25	64.40	820.50	853.75
ORF-T	YFR016C	-0.454725	0.1282622	0.2582356	34.55	25.20	304.50	318.75
ORF-T	YDR317W	-0.454357	0.2143991	0.3685019	9.03	6.62	86.75	87.50
ORF-T	YML110C	-0.45432	0.1827023	0.3309448	108.19	78.95	960.50	1008.25
ORF-T	YGR078C	-0.454297	0.1471068	0.2842891	24.11	17.55	222.75	242.75
ORF-T	YGR203W	-0.454241	0.1607251	0.302829	10.50	7.60	93.75	104.25
ORF-T	YPR149W	-0.454146	0.365658	0.5285144	103.29	75.35	845.00	989.25
ORF-T	YER064C	-0.453943	0.0810897	0.1839615	32.77	23.87	297.75	324.25
ORF-T	YKL018C-A	-0.453464	0.1040085	0.2219729	11.76	8.61	108.50	111.00
ORF-T	YOL005C	-0.45341	0.1146495	0.2375926	11.14	8.12	98.25	104.00
ORF-T	YER112W	-0.453128	0.1221554	0.2487593	30.11	21.97	271.00	288.50
AST	AS_YNL171C	-0.453013	0.1791995	0.3262407	4.58	3.34	43.50	45.00
sn/snoRNA	SNR9	-0.452972	0.1988836	0.3497022	1371.11	1001.64	11849.25	12422.00
AST	AS_YNL119W	-0.452838	0.2226607	0.3787636	2.73	1.96	23.25	25.25
ORF-T	YDL160C	-0.452788	0.1443504	0.2810639	40.88	29.81	350.50	393.50

Class	Transcript name	RRP6 KO_vs_WT log2_fold _change	RRP6 KO_vs_WT p-value	RRP6 KO_vs_WT FDR	Ave Norm Reads in WT	Ave Norm Reads in RRP6	Average RAW read counts in WT	Average RAW read counts in RRP6
ORF-T	YER082C	-0.452772	0.0705427	0.1660874	26.58	19.45	246.25	251.00
ORF-T	YGL067W	-0.4525	0.0631855	0.1528701	64.59	47.24	608.25	616.75
AST	AS_YKL006C-A	-0.452355	0.1844543	0.3326842	6.60	4.82	59.00	61.50
ORF-T	YER148W	-0.452286	0.0652492	0.1565112	110.11	80.53	1024.00	1032.00
ORF-T	YLR004C	-0.452269	0.2785672	0.4422944	3.15	2.29	29.25	31.50
ORF-T	YKL007W	-0.45216	0.1422747	0.2784878	65.12	47.58	586.00	619.00
AST	AS_YDL185C-A	-0.451858	0.1166975	0.2400607	44.78	32.77	426.75	426.25
ORF-T	YGL208W	-0.451763	0.1454712	0.2823958	24.20	17.69	223.00	233.50
ORF-T	YMR109W	-0.451509	0.0895921	0.1979507	113.90	83.28	1043.25	1104.00
AST	AS_YPR170C	-0.451431	0.1429457	0.279252	7.44	5.41	65.50	70.25
ORF-T	YIL053W	-0.4511	0.1243471	0.2521447	56.83	41.53	506.75	550.00
ORF-T	YJR030C	-0.450837	0.1607209	0.302829	10.87	7.97	96.75	99.75
ORF-T	YAL043C	-0.450498	0.1562425	0.2966018	18.58	13.53	162.75	182.25
ORF-T	YKL045W	-0.450454	0.0639243	0.1541397	65.55	48.04	614.00	609.75
ORF-T	YJL122W	-0.45009	0.0938515	0.2052479	66.68	48.81	626.00	656.75
ORF-T	YBR003W	-0.450073	0.2123948	0.3661178	23.51	17.18	203.50	222.50
ORF-T	YJR154W	-0.44953	0.1886344	0.3377309	10.17	7.46	94.25	97.75
ORF-T	YJR052W	-0.449262	0.083208	0.1877112	24.98	18.29	223.50	234.75
ORF-T	YOL026C	-0.449154	0.1365988	0.2707276	10.43	7.60	91.00	98.75
ORF-T	YDR468C	-0.449086	0.0982104	0.2124312	23.72	17.39	217.75	225.25
SUT	SUT454	-0.448964	0.4747954	0.6220736	0.85	0.63	8.25	8.50
NUT	NUT1210	-0.448826	0.2212249	0.3774673	7.40	5.41	72.00	76.00
ORF-T	YLR300W	-0.448683	0.1235741	0.2509307	390.88	286.39	3655.50	3831.25
ORF-T	YBR269C	-0.448496	0.2124731	0.3661423	18.09	13.22	154.75	169.25
ORF-T	YDL170W	-0.448364	0.1460234	0.2831186	20.21	14.76	188.75	207.25
ORF-T	YNL306W	-0.448227	0.086944	0.1937126	41.14	30.18	369.75	377.50
ORF-T	YGR180C	-0.447815	0.1975591	0.34798	358.46	262.75	3250.50	3625.00
ORF-T	YOR126C	-0.447696	0.1047185	0.2230632	15.01	10.98	137.00	145.50
ORF-T	YGL045W	-0.447575	0.284838	0.4484475	6.16	4.50	52.25	56.00

Class	Transcript name	RRP6 KO_vs_WT log2_fold _change	RRP6 KO_vs_WT p-value	RRP6 KO_vs_WT FDR	Ave Norm Reads in WT	Ave Norm Reads in RRP6	Average RAW read counts in WT	Average RAW read counts in RRP6
ORF-T	YHR104W	-0.447489	0.2342384	0.3928903	13.39	9.77	110.50	126.25
ORF-T	YMR273C	-0.447417	0.11667	0.2400531	19.12	14.02	173.75	181.25
ORF-T	YOR187W	-0.446617	0.1604624	0.3026189	260.15	190.89	2341.00	2401.50
AST	AS_YKL169C	-0.446596	0.2051105	0.3569787	14.91	10.93	129.50	137.00
ORF-T	YBR206W	-0.446323	0.0875469	0.1946687	41.20	30.26	374.50	383.00
ORF-T	YGR012W	-0.446144	0.0771619	0.1773246	19.72	14.47	181.75	191.50
ORF-T	YGR124W	-0.446117	0.0898647	0.1981902	729.06	535.15	6724.25	6947.75
ORF-T	YDR524C-A	-0.446041	0.1203264	0.245685	12.81	9.39	118.50	125.25
ORF-T	YLR066W	-0.445895	0.1048241	0.2231341	29.12	21.34	262.00	282.50
ORF-T	YKL104C	-0.445825	0.0878801	0.1951945	477.36	350.51	4495.50	4556.00
ORF-T	YDR330W	-0.445467	0.1717288	0.3165854	27.00	19.79	238.75	261.25
ORF-T	YBR162W-A	-0.445405	0.1138098	0.235998	62.33	45.80	566.00	581.75
ORF-T	YPR074C	-0.445364	0.0832915	0.1878156	694.15	509.75	6322.25	6754.75
ORF-T	YHR199C	-0.444791	0.0681458	0.161728	48.28	35.45	448.00	477.25
ORF-T	YHR143W-A	-0.44471	0.1149639	0.2380007	52.61	38.68	478.75	489.75
ORF-T	YML051W	-0.444635	0.0661669	0.1582607	39.79	29.19	352.75	380.00
ORF-T	YPL132W	-0.444448	0.1507189	0.2889043	31.03	22.80	275.50	287.50
ORF-T	YLR043C	-0.444413	0.1954867	0.3455369	93.94	69.01	838.25	900.50
sn/snoRNA	SNR77	-0.444249	0.1494594	0.2874043	3851.44	2830.72	35310.75	35703.75
ORF-T	YDR003W-A	-0.44422	0.1738873	0.3194154	7.05	5.18	63.00	66.25
ORF-T	YAL035W	-0.444057	0.0986374	0.2132176	473.53	348.10	4439.75	4550.75
ORF-T	YML116W	-0.443995	0.0737575	0.1714153	38.63	28.39	352.50	374.50
ORF-T	YNL125C	-0.443797	0.1897526	0.3388463	12.40	9.03	103.75	121.25
ORF-T	YMR275C	-0.443669	0.0981402	0.2123247	38.48	28.26	339.75	369.25
ORF-T	YCR020C-A	-0.443625	0.1388535	0.2733721	14.95	11.00	136.25	142.75
NUT	NUT0654	-0.443558	0.1832896	0.3315918	11.51	8.42	104.75	113.75
ORF-T	YOL027C	-0.443471	0.078122	0.1789195	45.41	33.34	407.50	442.50
ORF-T	YDR270W	-0.443297	0.0863514	0.1926904	45.66	33.61	414.75	427.25
ORF-T	YIL155C	-0.443262	0.200276	0.351293	59.28	43.58	518.25	561.00

Class	Transcript name	RRP6 KO_vs_WT log2_fold _change	RRP6 KO_vs_WT p-value	RRP6 KO_vs_WT FDR	Ave Norm Reads in WT	Ave Norm Reads in RRP6	Average RAW read counts in WT	Average RAW read counts in RRP6
ORF-T	YLR285W	-0.443232	0.058076	0.1435542	55.76	41.05	524.25	536.50
ORF-T	YFL011W	-0.443182	0.2003523	0.3513045	3.75	2.80	36.00	36.00
AST	AS_YDR271C	-0.443172	0.1791608	0.3262407	6.72	4.96	60.75	62.50
NUT	NUT1416	-0.443113	0.2078853	0.3602536	1372.52	1009.55	11861.50	12526.50
ORF-T	YGL023C	-0.443044	0.0891003	0.19708	54.31	39.97	514.25	527.50
ORF-T	YJL174W	-0.44252	0.1097492	0.2300326	85.41	62.80	754.75	827.00
ORF-T	YGR155W	-0.442402	0.1195091	0.2444483	192.44	141.58	1682.25	1814.00
ORF-T	YAL053W	-0.44235	0.0887472	0.1966268	89.42	65.79	825.00	884.50
ORF-T	YOR160W	-0.442178	0.1047482	0.2230794	18.97	13.93	175.75	189.50
ORF-T	YOR016C	-0.441858	0.0720451	0.1682726	39.04	28.73	352.75	373.25
AST	AS_YDL034W	-0.441849	0.1025969	0.219751	12.79	9.41	116.75	122.25
ORF-T	YBL041W	-0.441773	0.0881661	0.1956573	107.70	79.26	978.00	1044.25
ORF-T	YJR139C	-0.441697	0.1313676	0.2625789	477.18	351.31	4271.50	4556.25
ORF-T	YDL201W	-0.441688	0.1127176	0.2342148	15.06	11.08	134.75	143.25
ORF-T	YIR001C	-0.441349	0.2864631	0.4497232	3.66	2.63	30.75	36.25
ORF-T	YMR186W	-0.44124	0.2798675	0.4435348	1316.14	969.33	11773.75	12532.00
ORF-T	YLR068W	-0.441209	0.1540156	0.2935905	12.83	9.49	123.25	124.50
ORF-T	YLR256W	-0.440635	0.1449323	0.2816529	106.86	78.76	941.00	978.75
AST	AS_YGR290W	-0.440573	0.1863908	0.3352175	8.68	6.39	79.75	84.50
ORF-T	YML060W	-0.440357	0.1637312	0.3067184	31.22	23.07	294.50	288.75
ORF-T	YDR123C	-0.440276	0.1287609	0.2589084	8.57	6.28	77.50	83.50
ORF-T	YPR089W	-0.440161	0.1712906	0.3158915	40.56	29.84	365.50	406.25
ORF-T	YMR029C	-0.439944	0.0908561	0.2000421	68.19	50.27	613.00	638.50
ORF-T	YOR051C	-0.439769	0.0705676	0.1660939	105.22	77.58	984.25	1024.25
SUT	SUT699	-0.439697	0.303572	0.4693262	1.66	1.21	14.50	15.50
ORF-T	YLR015W	-0.439423	0.0787028	0.1797597	35.30	26.06	331.50	341.50
AST	AS_YLR322W	-0.43941	0.172055	0.3170078	5.59	4.09	50.00	54.00
ORF-T	YOL129W	-0.439403	0.1340824	0.2669853	108.49	80.00	983.25	1033.75
ORF-T	YEL053C	-0.439397	0.1066498	0.2261262	23.32	17.14	203.50	224.25

Class	Transcript name	RRP6 KO_vs_WT log2_fold _change	RRP6 KO_vs_WT p-value	RRP6 KO_vs_WT FDR	Ave Norm Reads in WT	Ave Norm Reads in RRP6	Average RAW read counts in WT	Average RAW read counts in RRP6
ORF-T	YBR243C	-0.439113	0.0742211	0.1722941	30.37	22.38	274.75	293.50
ORF-T	YGL200C	-0.439072	0.088248	0.1957528	143.84	106.08	1291.00	1375.00
ORF-T	YHR129C	-0.439069	0.116655	0.2400531	10.29	7.59	95.25	100.50
ORF-T	YER134C	-0.438817	0.2016741	0.3527964	15.68	11.51	134.50	151.25
ORF-T	YMR089C	-0.438736	0.1118852	0.2333025	88.03	64.96	816.00	845.50
ORF-T	YDR023W	-0.438664	0.1006083	0.2164309	424.29	313.07	3845.00	3973.00
ORF-T	YPR170W-A	-0.438565	0.164219	0.3073468	7.10	5.17	60.75	68.25
ORF-T	YIL156W-B	-0.4383	0.1557322	0.2960232	11.95	8.81	105.50	111.00
ORF-T	YLR393W	-0.438273	0.1973043	0.3477387	8.02	5.92	71.25	75.25
ORF-T	YLR032W	-0.438215	0.1032559	0.2207404	71.70	52.98	671.75	670.25
ORF-T	YDR420W	-0.438048	0.0943246	0.2060592	40.06	29.55	353.25	377.00
ORF-T	YDL164C	-0.437934	0.0746236	0.1729896	64.60	47.75	604.00	606.50
ORF-T	YPR011C	-0.437906	0.1094108	0.2298539	26.53	19.53	236.50	260.75
ORF-T	YLR292C	-0.437818	0.0772175	0.1773854	35.31	26.03	328.00	354.75
AST	AS_YDL016C	-0.437606	0.2574532	0.4190055	2.91	2.17	26.75	27.50
ORF-T	YCR024C-A	-0.437545	0.1262821	0.2548896	85.04	62.69	739.00	862.50
ORF-T	YOR035C	-0.437526	0.1257763	0.2542488	46.05	33.95	416.75	460.75
ORF-T	YIL005W	-0.437486	0.0775639	0.1779242	29.57	21.78	261.75	286.50
ORF-T	YFR011C	-0.437263	0.279417	0.4432344	10.31	7.58	87.75	97.75
SUT	SUT128	-0.436833	0.2561544	0.417964	4.46	3.24	37.50	42.75
AST	AS_YNR002C	-0.436811	0.1658059	0.3094553	9.42	6.97	84.50	88.50
ORF-T	YGL053W	-0.436587	0.1548193	0.2946211	18.96	13.98	166.00	181.00
ORF-T	YHR174W	-0.436572	0.2537486	0.4151875	3348.76	2474.31	30119.25	33624.75
ORF-T	YLR182W	-0.435974	0.0876354	0.1948227	34.81	25.71	326.00	347.50
ORF-T	YPL048W	-0.435835	0.0879624	0.1952911	238.14	176.06	2167.75	2271.00
ORF-T	YDR525W-A	-0.435829	0.1776159	0.3242946	11.91	8.76	101.00	112.00
ORF-T	YJR143C	-0.435726	0.1122791	0.2336972	503.55	372.28	4532.25	4802.25
NUT	NUT1131	-0.435573	0.2064954	0.3586462	59.58	44.03	521.00	566.75
AST	AS_YLR405W	-0.435524	0.168877	0.3128709	8.99	6.61	80.50	88.25

TABLE S1: Differential expression data for RRP6 RNA-Seq dataset Page 75

Class	Transcript name	RRP6 KO_vs_WT log2_fold _change	RRP6 KO_vs_WT p-value	RRP6 KO_vs_WT FDR	Ave Norm Reads in WT	Ave Norm Reads in RRP6	Average RAW read counts in WT	Average RAW read counts in RRP6
ORF-T	YDL019C	-0.435483	0.2677197	0.4307092	103.30	76.37	922.75	992.25
ORF-T	YNL191W	-0.435201	0.2792708	0.4432116	10.42	7.69	93.00	98.50
ORF-T	YKL128C	-0.435094	0.0682066	0.1618105	97.11	71.83	897.75	941.50
ORF-T	YPR161C	-0.435065	0.0809994	0.1838431	28.02	20.75	260.50	269.25
ORF-T	YBL033C	-0.435064	0.1076832	0.2276472	34.89	25.81	319.00	334.75
ORF-T	YLR149C	-0.435037	0.395755	0.5566737	26.50	19.58	216.50	241.00
ORF-T	YDR389W	-0.434697	0.0780297	0.1787893	32.76	24.19	296.00	324.25
ORF-T	YDL070W	-0.434681	0.0824174	0.1865439	325.46	240.83	3026.25	3123.00
ORF-T	YML065W	-0.434226	0.10296	0.2203865	31.24	23.13	287.75	301.75
ORF-T	YHR084W	-0.433878	0.1168136	0.2402243	17.93	13.22	161.00	177.50
ORF-T	YMR075W	-0.433845	0.1485493	0.2862873	6.65	4.88	58.25	64.00
ORF-T	YBR173C	-0.43381	0.2160713	0.3708072	8.97	6.60	76.75	85.25
AST	AS_YIL071W-A	-0.433783	0.1761589	0.3224516	6.23	4.62	58.75	61.25
ORF-T	YDR189W	-0.433614	0.1158176	0.2390308	45.32	33.49	404.50	450.75
ORF-T	YCR082W	-0.433458	0.120333	0.245685	42.88	31.76	381.50	399.00
ORF-T	YEL021W	-0.433366	0.1541262	0.2936964	8.54	6.35	78.00	80.00
ORF-T	YLR447C	-0.432938	0.1445724	0.2812788	250.68	185.67	2272.50	2450.00
AST	AS_YOL030W	-0.432922	0.4089846	0.5669332	1.20	0.86	10.50	12.00
ORF-T	YHR149C	-0.432759	0.1282727	0.2582356	93.57	69.31	871.25	923.25
ORF-T	YDR100W	-0.432614	0.34156	0.5061412	9.19	6.77	76.25	87.00
AST	AS_YOR335W-A	-0.432319	0.0741967	0.1722771	32.07	23.80	304.00	313.25
ORF-T	YCR079W	-0.432192	0.1306648	0.2614909	38.03	28.20	350.00	365.50
ORF-T	YOR176W	-0.43211	0.1743947	0.3202106	35.49	26.26	313.00	343.50
ORF-T	YFR010W	-0.431672	0.1343085	0.2672566	178.49	132.31	1614.75	1747.50
ORF-T	YLR436C	-0.431631	0.1386037	0.2732512	34.76	25.74	294.75	324.75
ORF-T	YNL111C	-0.431598	0.1738706	0.3194154	14.89	10.98	133.75	150.50
AST	AS_YJR079W	-0.431378	0.1348774	0.2680536	21.11	15.66	195.00	204.00
ORF-T	YDR032C	-0.431338	0.2294064	0.3869746	56.82	42.12	495.25	527.25
CUT	CUT894	-0.431337	0.2976039	0.4623383	4.97	3.70	43.50	44.50

Class	Transcript name	RRP6 KO_vs_WT log2_fold _change	RRP6 KO_vs_WT p-value	RRP6 KO_vs_WT FDR	Ave Norm Reads in WT	Ave Norm Reads in RRP6	Average RAW read counts in WT	Average RAW read counts in RRP6
ORF-T	YPR191W	-0.4313	0.2804942	0.4441746	139.84	103.69	1230.25	1311.75
ORF-T	YLR209C	-0.431292	0.0743277	0.1725019	39.13	29.01	364.75	387.25
ORF-T	YDR251W	-0.431291	0.1322056	0.2639924	55.75	41.30	495.75	543.25
ORF-T	YML077W	-0.431141	0.1174299	0.2410756	22.91	16.96	204.50	220.75
ORF-T	YOR142W	-0.430753	0.2228721	0.3789315	25.00	18.51	220.50	243.00
ORF-T	YER164W	-0.430647	0.0638101	0.1539749	84.24	62.43	759.50	836.25
ORF-T	YCL025C	-0.430616	0.192822	0.3424876	295.68	219.44	2738.25	2697.25
ORF-T	YDL045C	-0.430494	0.1391417	0.2737231	27.45	20.34	240.50	262.00
ORF-T	YPR091C	-0.430252	0.1911757	0.3404832	38.31	28.38	332.50	371.00
ORF-T	YMR204C	-0.43015	0.1545074	0.2941387	7.46	5.50	65.75	72.50
ORF-T	YGR150C	-0.430041	0.0870761	0.1938784	34.88	25.88	313.75	335.25
ORF-T	YLR220W	-0.4299	0.0843709	0.1896558	153.05	113.66	1431.75	1455.50
ORF-T	YDL119C	-0.42987	0.1332465	0.265545	23.93	17.76	222.25	235.00
ORF-T	YJR057W	-0.429847	0.1082037	0.2279829	12.97	9.66	119.75	124.00
ORF-T	YIL021W	-0.429843	0.0794574	0.1811549	115.49	85.78	1070.25	1092.75
ORF-T	YML013W	-0.429672	0.1058984	0.224864	44.89	33.26	404.25	450.50
ORF-T	YML052W	-0.42945	0.219886	0.3758623	18.20	13.45	153.75	179.25
ORF-T	YHR020W	-0.429378	0.0844751	0.1897303	293.76	218.14	2675.75	2839.50
ORF-T	YGL036W	-0.429356	0.1380508	0.2723741	26.67	19.79	232.25	251.00
ORF-T	YDR003W	-0.429351	0.1257042	0.2542322	19.66	14.60	173.00	183.25
AST	AS_YAL037C-A	-0.429257	0.167577	0.3113776	101.84	75.59	888.75	985.25
ORF-T	YLR262C-A	-0.429168	0.1838409	0.3322314	98.14	72.93	886.25	902.75
ORF-T	YLR313C	-0.428998	0.2539543	0.4154567	27.43	20.42	255.75	252.00
ORF-T	YIL125W	-0.428939	0.1688102	0.312862	136.94	101.69	1209.50	1321.75
ORF-T	YLR310C	-0.428888	0.0977419	0.2115993	113.87	84.52	998.25	1115.75
ORF-T	YJR131W	-0.428581	0.0706725	0.1662379	64.45	47.90	594.25	620.50
ORF-T	YML068W	-0.428417	0.1075775	0.2274714	23.26	17.31	217.75	227.00
ORF-T	YCL028W	-0.42832	0.0729917	0.1699096	102.89	76.47	948.75	997.00
ORF-T	YDR204W	-0.428119	0.1834241	0.3316566	36.36	26.99	322.00	351.25

Class	Transcript name	RRP6 KO_vs_WT log2_fold _change	RRP6 KO_vs_WT p-value	RRP6 KO_vs_WT FDR	Ave Norm Reads in WT	Ave Norm Reads in RRP6	Average RAW read counts in WT	Average RAW read counts in RRP6
ORF-T	YOL119C	-0.427944	0.1016928	0.2181387	56.89	42.27	527.00	566.75
ORF-T	YLR301W	-0.427766	0.1123291	0.2336972	114.43	85.04	1043.50	1119.25
ORF-T	YLR133W	-0.427605	0.1677879	0.3116546	26.35	19.56	236.25	257.75
ORF-T	YML038C	-0.42759	0.0810011	0.1838431	40.41	30.07	380.25	396.00
ORF-T	YDL085W	-0.427244	0.0825926	0.1867826	25.58	19.05	234.50	246.25
ORF-T	YCL029C	-0.427113	0.1389886	0.2735822	13.94	10.32	124.50	137.75
ORF-T	YPR037C	-0.426768	0.0815399	0.1847749	32.55	24.22	294.75	310.25
ORF-T	YJR007W	-0.426709	0.0969123	0.2102094	54.02	40.13	472.00	527.25
SUT	SUT674	-0.426676	0.2216559	0.377883	10.60	7.84	95.00	106.75
ORF-T	YER142C	-0.426659	0.2440416	0.4040912	20.62	15.33	179.00	191.25
ORF-T	YGR105W	-0.426435	0.1068433	0.2264024	15.33	11.39	139.50	149.50
ORF-T	YLL031C	-0.426385	0.0928835	0.2034878	52.46	38.95	464.25	523.00
ORF-T	YGL225W	-0.426138	0.1156699	0.2389706	146.21	108.80	1320.50	1416.00
AST	AS_YLL028W	-0.425905	0.3319374	0.4961839	3.08	2.32	31.25	31.50
ORF-T	YOR123C	-0.425675	0.2036106	0.3550423	20.63	15.33	187.50	205.50
ORF-T	YLR408C	-0.425666	0.3500383	0.5144219	1.72	1.24	14.75	16.75
ORF-T	YMR190C	-0.425648	0.0844353	0.1897303	71.85	53.46	660.25	715.25
ORF-T	YIL020C	-0.425582	0.1638537	0.3068339	9.07	6.73	84.25	91.25
ORF-T	YBR160W	-0.425543	0.0712797	0.1671592	96.90	72.16	890.25	940.00
ORF-T	YGL047W	-0.425353	0.2071197	0.3594209	29.28	21.81	262.50	275.00
AST	AS_YPL222C-A	-0.425277	0.189206	0.3384096	7.22	5.40	68.75	70.75
ORF-T	YJL134W	-0.424747	0.093608	0.2047598	64.38	48.00	601.50	615.00
AST	AS_YOL144W	-0.424568	0.2873091	0.45065	2.47	1.79	21.25	24.25
ORF-T	YDR493W	-0.424182	0.1819003	0.3299662	5.22	3.84	46.25	51.50
ORF-T	YLR172C	-0.42378	0.0753804	0.174423	59.01	43.98	540.50	581.75
ORF-T	YIR009W	-0.423723	0.1814312	0.3294709	8.92	6.60	79.25	89.25
sn/snoRNA	snR161	-0.423553	0.1796715	0.326982	387.64	289.02	3399.00	3629.25
ORF-T	YAL039C	-0.423116	0.0911832	0.2005872	46.77	34.91	430.50	450.25
ORF-T	YLR104W	-0.423017	0.1820299	0.3301347	10.01	7.47	89.25	95.00

Class	Transcript name	RRP6 KO_vs_WT log2_fold _change	RRP6 KO_vs_WT p-value	RRP6 KO_vs_WT FDR	Ave Norm Reads in WT	Ave Norm Reads in RRP6	Average RAW read counts in WT	Average RAW read counts in RRP6
ORF-T	YDR174W	-0.422748	0.1079586	0.2277045	46.62	34.72	411.75	456.25
ORF-T	YDR429C	-0.422672	0.091256	0.2007035	249.30	186.04	2320.00	2369.00
ORF-T	YNL229C	-0.422656	0.1492031	0.2870757	24.68	18.36	219.50	244.25
ORF-T	YDR478W	-0.422624	0.2710973	0.4341345	2.25	1.66	20.25	22.00
ORF-T	YNR030W	-0.422618	0.180762	0.3285515	35.57	26.52	323.00	348.50
ORF-T	YHL013C	-0.422522	0.1229986	0.2500136	17.43	13.00	165.00	174.50
ORF-T	YBR204C	-0.422505	0.1064216	0.2257374	46.61	34.81	428.00	440.25
ORF-T	YBR279W	-0.422502	0.1898309	0.3389261	21.23	15.80	192.75	212.50
ORF-T	YDL018C	-0.422388	0.1376663	0.2718763	24.15	18.05	218.25	224.50
ORF-T	YDR009W	-0.422015	0.1906683	0.3399397	6.76	5.04	61.25	66.25
ORF-T	YGL212W	-0.421677	0.125848	0.2543189	14.45	10.78	130.75	140.75
ORF-T	YNL288W	-0.421492	0.2104552	0.3634927	13.75	10.22	119.25	134.75
ORF-T	YJL089W	-0.421273	0.3849131	0.5460697	2.36	1.76	21.75	23.00
ORF-T	YBR263W	-0.421225	0.142184	0.2784878	222.77	166.36	2087.75	2217.00
ORF-T	YMR156C	-0.420872	0.1341016	0.2669853	12.24	9.11	111.75	121.75
ORF-T	YLR108C	-0.420824	0.2250592	0.3816846	16.74	12.47	155.50	171.50
ORF-T	YFL045C	-0.42081	0.1362853	0.2702194	675.88	504.95	6212.25	6254.75
ORF-T	YFL036W	-0.420796	0.1368374	0.2709876	200.81	149.97	1800.50	1978.25
ORF-T	YHL019C	-0.420699	0.2512085	0.4123025	13.30	9.88	113.50	131.75
ORF-T	YNL220W	-0.420497	0.1231925	0.2503069	223.62	167.14	2053.50	2077.00
ORF-T	YDL097C	-0.420265	0.2360919	0.3951453	130.66	97.62	1158.00	1279.25
ORF-T	YER119C	-0.420146	0.2314656	0.389601	9.48	7.08	81.50	87.75
ORF-T	YGL253W	-0.420108	0.2018867	0.3530742	777.26	580.87	7010.75	7719.00
ORF-T	YHL036W	-0.41967	0.3216133	0.4859378	70.35	52.61	606.50	625.75
ORF-T	YDR013W	-0.419337	0.1708125	0.3154135	12.92	9.71	120.00	123.00
ORF-T	YJL097W	-0.419253	0.1741549	0.3198284	9.41	6.97	80.00	92.00
AST	AS_YDL086C-A	-0.419213	0.2194329	0.3753625	20.91	15.60	184.00	204.50
ORF-T	YNL067W-B	-0.418663	0.1242479	0.2520451	21.92	16.39	196.00	210.25
NUT	NUT1486	-0.418607	0.1483557	0.2861004	9.15	6.83	81.00	88.25

TABLE S1: Differential expression data for RRP6 RNA-Seq dataset Page 79

Class	Transcript name	RRP6 KO_vs_WT log2_fold _change	RRP6 KO_vs_WT p-value	RRP6 KO_vs_WT FDR	Ave Norm Reads in WT	Ave Norm Reads in RRP6	Average RAW read counts in WT	Average RAW read counts in RRP6
ORF-T	YLR382C	-0.41855	0.1221577	0.2487593	67.93	50.85	617.00	643.75
ORF-T	YGL099W	-0.418518	0.0991007	0.2139897	80.37	60.16	759.00	800.50
AST	AS_YDL221W	-0.418288	0.2473558	0.4079713	2.30	1.69	21.25	23.25
ORF-T	YLR132C	-0.418264	0.3143759	0.4796918	10.68	7.99	97.75	103.50
ORF-T	YDR098C	-0.418137	0.0854432	0.19129	52.69	39.35	463.75	521.75
ORF-T	YNL298W	-0.418106	0.1512659	0.289718	25.07	18.69	220.75	253.75
ORF-T	YDR182W	-0.418089	0.0838519	0.1886996	88.57	66.29	810.50	864.00
ORF-T	YDR248C	-0.418058	0.2126242	0.3661657	10.51	7.86	94.50	100.25
ORF-T	YGL181W	-0.417901	0.2422575	0.4019291	13.39	9.99	117.00	130.00
ORF-T	YKR097W	-0.417861	0.4795234	0.6254707	3.96	2.99	37.25	36.75
ORF-T	YLR273C	-0.417644	0.1868102	0.3357324	11.02	8.22	100.00	109.75
ORF-T	YHR146W	-0.417544	0.1010632	0.2170191	134.79	100.98	1239.00	1257.25
ORF-T	YLR435W	-0.417447	0.0786526	0.1796856	36.43	27.29	336.25	356.50
ORF-T	YKR053C	-0.417308	0.1928855	0.3424876	14.42	10.80	137.50	148.00
SUT	SUT400	-0.417253	0.250636	0.4123025	6.92	5.20	63.25	65.50
ORF-T	YHR107C	-0.417141	0.0776827	0.178075	89.90	67.37	849.75	880.50
SRT	SRT510	-0.417081	0.3369928	0.5014456	2.75	2.07	26.00	27.50
ORF-T	YIL062C	-0.417027	0.2281529	0.3851595	97.43	72.95	858.75	940.25
ORF-T	YHL023C	-0.416683	0.1158041	0.2390308	27.83	20.80	249.50	275.50
ORF-T	YGR184C	-0.416503	0.1206547	0.2462919	183.37	137.34	1637.75	1818.50
ORF-T	YDL207W	-0.416467	0.1098843	0.2301294	31.94	23.89	288.75	318.25
sn/snoRNA	SNR43	-0.416456	0.2267263	0.3835257	1973.65	1478.76	17429.75	19403.00
ORF-T	YJL051W	-0.416217	0.4078066	0.566026	13.62	10.16	119.25	142.25
ORF-T	YKR002W	-0.416069	0.090003	0.1984237	74.98	56.18	693.25	743.25
ORF-T	YPL273W	-0.415651	0.0888218	0.1967229	63.71	47.75	576.00	621.50
ORF-T	YFR024C-A	-0.415297	0.1664096	0.3100081	136.47	102.33	1231.00	1308.00
ORF-T	YGR161W-C	-0.415151	0.1501674	0.2882163	11.97	8.97	107.25	115.75
ORF-T	YDL202W	-0.415065	0.0999158	0.2153347	27.69	20.77	255.25	271.00
ORF-T	YJR092W	-0.414966	0.1711955	0.3157739	41.53	31.06	363.00	427.50

Class	Transcript name	RRP6 KO_vs_WT log2_fold _change	RRP6 KO_vs_WT p-value	RRP6 KO_vs_WT FDR	Ave Norm Reads in WT	Ave Norm Reads in RRP6	Average RAW read counts in WT	Average RAW read counts in RRP6
ORF-T	YER110C	-0.414397	0.1247765	0.2528177	398.79	299.23	3576.00	3814.50
AST	AS_YBL077W	-0.414392	0.1008039	0.2166006	263.88	198.01	2417.50	2568.00
ORF-T	YJR076C	-0.41437	0.0914842	0.2011177	54.19	40.61	493.00	546.75
ORF-T	YJR080C	-0.414255	0.137375	0.2714657	31.79	23.87	295.00	309.25
ORF-T	YKL156W	-0.41425	0.1248119	0.2528177	51.32	38.44	455.25	515.75
ORF-T	YLR398C	-0.414169	0.1164273	0.2397319	69.89	52.43	644.50	700.75
ORF-T	YML061C	-0.414105	0.1001942	0.2157501	56.84	42.73	541.00	547.50
ORF-T	YDR141C	-0.413704	0.1306936	0.2614909	36.20	27.13	325.25	363.00
ORF-T	YLR105C	-0.413486	0.0995275	0.2147272	26.88	20.25	256.50	261.75
ORF-T	YNL085W	-0.413476	0.1114639	0.2326246	259.88	195.09	2320.00	2536.50
SRT	SRT517	-0.41336	0.4044819	0.5631716	1.73	1.26	15.25	17.75
SUT	SUT315	-0.41329	0.1458311	0.2828545	14.56	10.96	135.25	141.50
AST	AS_YOR334W	-0.413247	0.4462751	0.5985134	1.54	1.17	15.25	15.75
SUT	SUT714	-0.413226	0.337953	0.5021983	2.84	2.14	26.75	28.25
ORF-T	YEL018W	-0.413135	0.1504276	0.2886057	13.07	9.79	120.25	131.50
ORF-T	YKL117W	-0.412852	0.1629168	0.3056328	303.58	228.04	2720.00	2872.00
ORF-T	YKL089W	-0.41272	0.1323553	0.264239	24.43	18.38	225.75	236.00
ORF-T	YHR127W	-0.412504	0.1445251	0.2812411	11.18	8.41	105.00	111.00
ORF-T	YKL108W	-0.412281	0.1463594	0.2835519	11.17	8.41	102.75	108.75
ORF-T	YGL143C	-0.411813	0.1518182	0.2903898	19.87	14.91	178.00	194.75
ORF-T	YML011C	-0.411665	0.1447166	0.2814508	9.74	7.30	90.75	98.25
ORF-T	YJL096W	-0.411626	0.1427619	0.2789944	21.46	16.12	192.25	207.25
ORF-T	YHR202W	-0.411553	0.1701351	0.3145082	27.35	20.59	252.75	264.00
ORF-T	YBR205W	-0.411371	0.1120859	0.2335577	134.59	101.23	1216.50	1272.50
ORF-T	YAL014C	-0.411141	0.0771558	0.1773246	50.03	37.66	460.75	483.25
ORF-T	YDL084W	-0.411004	0.1255865	0.2540449	777.69	584.90	7106.25	7585.50
ORF-T	YOR228C	-0.41097	0.2323474	0.3903685	28.80	21.66	264.00	283.00
ORF-T	YPR033C	-0.410887	0.1092742	0.2296147	208.31	156.70	1871.75	1979.75
ORF-T	YPL019C	-0.41071	0.277491	0.4413612	622.60	468.39	5652.50	5822.00

Class	Transcript name	RRP6 KO_vs_WT log2_fold _change	RRP6 KO_vs_WT p-value	RRP6 KO_vs_WT FDR	Ave Norm Reads in WT	Ave Norm Reads in RRP6	Average RAW read counts in WT	Average RAW read counts in RRP6
ORF-T	YJL184W	-0.410451	0.1456001	0.2825152	12.08	9.10	111.75	118.50
ORF-T	YPL160W	-0.41043	0.1305145	0.2614438	779.69	586.67	7334.75	7602.25
ORF-T	YER056C	-0.410256	0.1437772	0.2803808	476.77	358.80	4418.25	4601.50
ORF-T	YDR154C	-0.410124	0.2734106	0.4366602	210.91	158.66	1758.00	2056.50
ORF-T	YEL047C	-0.410045	0.1815624	0.3295904	83.37	62.70	775.50	870.50
ORF-T	YKL170W	-0.40998	0.2263538	0.3831071	18.55	13.94	160.75	176.00
AST	AS_YHR056W-A	-0.409844	0.2889058	0.4528018	3.76	2.78	32.75	37.50
ORF-T	YNR013C	-0.409804	0.1825518	0.3307317	13.18	9.87	114.75	129.50
ORF-T	YOL017W	-0.409743	0.1880858	0.3370637	18.25	13.76	165.00	173.50
ORF-T	YHR172W	-0.40965	0.0769768	0.1771414	38.31	28.86	355.25	378.50
ORF-T	YMR157C	-0.409533	0.1419797	0.2783289	16.07	12.08	145.25	158.75
ORF-T	YER074W-A	-0.409532	0.0997497	0.2150686	33.33	25.06	300.00	328.75
ORF-T	YFR053C	-0.409519	0.479203	0.6252145	534.21	402.18	4318.75	4928.00
ORF-T	YHR070W	-0.409512	0.1660159	0.3096753	55.32	41.61	522.00	582.75
ORF-T	YGR047C	-0.409402	0.1071703	0.2267532	62.22	46.82	572.75	625.25
AST	AS_YMR141W-A	-0.409254	0.2952204	0.4599108	2.85	2.15	25.25	27.25
ORF-T	YJR033C	-0.408808	0.1410042	0.2767392	59.08	44.47	528.25	581.50
AST	AS_YGR190C	-0.40875	0.2227755	0.3788386	6.75	5.09	62.00	66.50
ORF-T	YMR202W	-0.40853	0.1324117	0.2642992	115.06	86.64	1041.25	1157.25
ORF-T	YNR035C	-0.408474	0.1526964	0.2916823	118.44	89.20	1065.25	1177.00
AST	AS_YGR265W	-0.408423	0.0760735	0.1755435	42.60	32.09	391.75	421.75
ORF-T	YER019C-A	-0.40835	0.1564476	0.2968794	290.91	219.23	2658.25	2749.25
ORF-T	YEL077C	-0.408293	0.2210232	0.3771871	9.94	7.50	92.25	97.75
ORF-T	YPR111W	-0.40802	0.1499267	0.2879734	18.70	14.04	172.50	194.25
ORF-T	YKR016W	-0.407769	0.1896382	0.3388463	25.81	19.42	225.25	252.50
ORF-T	YDR287W	-0.40769	0.1312729	0.2624418	21.96	16.57	200.75	213.25
ORF-T	YOL097C	-0.407684	0.1006176	0.2164309	234.07	176.48	2195.75	2304.50
ORF-T	YDR279W	-0.407645	0.1138082	0.235998	22.23	16.79	207.50	217.50
ORF-T	YML076C	-0.407605	0.1844236	0.3326842	7.81	5.82	66.00	76.25

Class	Transcript name	RRP6 KO_vs_WT log2_fold _change	RRP6 KO_vs_WT p-value	RRP6 KO_vs_WT FDR	Ave Norm Reads in WT	Ave Norm Reads in RRP6	Average RAW read counts in WT	Average RAW read counts in RRP6
ORF-T	YJL135W	-0.407455	0.2543339	0.4159427	3.15	2.40	29.50	30.50
ORF-T	YHR209W	-0.407262	0.2264473	0.3831724	10.15	7.64	93.00	102.25
ORF-T	YNL209W	-0.406945	0.1237863	0.2512604	1399.89	1055.80	12454.50	13718.75
ORF-T	YHR132W-A	-0.406933	0.1042472	0.2223412	45.95	34.66	409.25	438.00
AST	AS_YHR070C-A	-0.406723	0.2259562	0.3826081	13.17	9.91	126.50	139.75
ORF-T	YNL124W	-0.406354	0.2125489	0.3661657	16.01	12.02	146.75	167.50
ORF-T	YDR176W	-0.406225	0.1565346	0.2969682	33.68	25.40	303.25	329.00
ORF-T	YOR356W	-0.405637	0.1501021	0.2881667	106.82	80.67	1032.25	1069.00
ORF-T	YFL038C	-0.405556	0.1388228	0.2733721	325.36	245.68	2999.50	3048.50
ORF-T	YJL146W	-0.405516	0.1496176	0.2875989	17.63	13.27	157.25	175.25
ORF-T	YLR060W	-0.405063	0.1054743	0.2240579	418.94	316.37	3821.00	4132.75
ORF-T	YOL096C	-0.405061	0.3576248	0.5214897	5.29	3.95	43.75	51.75
AST	AS_YOR050C	-0.405022	0.3167115	0.4822843	4.02	3.04	38.00	40.75
ORF-T	YER100W	-0.40495	0.1100741	0.2303835	32.88	24.85	294.75	314.25
NUT	NUT0298	-0.404388	0.5198458	0.6586412	7.01	5.29	60.75	66.75
ORF-T	YLR213C	-0.404287	0.4531233	0.6040047	4.03	3.03	39.75	45.00
ORF-T	YBR244W	-0.404249	0.2411475	0.4008129	47.78	36.10	426.25	457.50
ORF-T	YLR041W	-0.404133	0.3697413	0.5324106	1.37	1.04	12.25	13.00
ORF-T	YMR006C	-0.404095	0.1704613	0.3149957	24.40	18.39	215.25	246.50
ORF-T	YDR430C	-0.403928	0.1370066	0.2710601	88.50	66.89	812.50	870.25
ORF-T	YGR205W	-0.403651	0.3886817	0.5496255	29.58	22.36	254.75	276.50
ORF-T	YPR204W	-0.40365	0.2471047	0.4077193	2.58	1.97	24.50	25.75
ORF-T	YHR201C	-0.403588	0.0805299	0.1831447	76.53	57.90	714.00	746.00
ORF-T	YLR289W	-0.40351	0.1539938	0.2935905	20.07	15.19	182.25	194.00
ORF-T	YGL021W	-0.403493	0.2262382	0.3829757	42.24	31.87	376.25	439.75
NUT	NUT1435	-0.403144	0.4887615	0.6335868	1.26	0.99	14.00	13.50
NUT	NUT0890	-0.402977	0.1626199	0.3053172	11.25	8.50	102.00	110.50
ORF-T	YNL088W	-0.402964	0.0839216	0.1888142	70.91	53.65	670.25	709.00
ORF-T	YOR189W	-0.402816	0.1431232	0.279538	18.86	14.25	169.00	184.25

Class	Transcript name	RRP6 KO_vs_WT log2_fold _change	RRP6 KO_vs_WT p-value	RRP6 KO_vs_WT FDR	Ave Norm Reads in WT	Ave Norm Reads in RRP6	Average RAW read counts in WT	Average RAW read counts in RRP6
ORF-T	YGL056C	-0.402765	0.1830359	0.3312515	21.51	16.21	189.75	216.00
NUT	NUT0859	-0.402565	0.241934	0.4016566	1974.67	1493.84	17438.75	19585.75
ORF-T	YDR501W	-0.402351	0.2820782	0.445703	4.67	3.52	42.50	46.75
ORF-T	YOR133W	-0.401638	0.1760891	0.3224409	246.69	186.71	2161.50	2406.25
ORF-T	YOR354C	-0.401326	0.190939	0.3401818	27.72	20.97	245.00	270.50
ORF-T	YPL059W	-0.401317	0.1940102	0.3437707	18.34	13.86	162.25	179.75
ORF-T	YDR006C	-0.401219	0.1331277	0.2653606	43.40	32.86	380.00	412.50
ORF-T	YHR056C	-0.401154	0.2015339	0.3527018	14.18	10.66	124.00	144.50
ORF-T	YLR087C	-0.400957	0.1157667	0.2390237	95.04	71.95	866.50	959.00
ORF-T	YGR098C	-0.40091	0.1120894	0.2335577	37.04	27.98	332.00	378.50
SUT	SUT099	-0.400879	0.2435472	0.403405	5.11	3.80	43.50	50.75
ORF-T	YMR264W	-0.400451	0.1434305	0.2798671	91.78	69.58	860.00	886.75
SRT	SRT292	-0.40023	0.4095156	0.5670908	0.99	0.76	10.25	10.75
ORF-T	YJL128C	-0.400126	0.1032047	0.2206778	76.59	57.99	697.00	776.75
ORF-T	YCL027W	-0.400109	0.3870539	0.5479946	2.94	2.22	25.50	28.00
ORF-T	YDR337W	-0.400098	0.1685187	0.3125954	18.93	14.30	163.00	184.25
ORF-T	YDR405W	-0.400041	0.1956022	0.34566	7.71	5.84	71.50	77.50
ORF-T	YGL076C	-0.39988	0.1361771	0.2702105	338.30	256.39	2964.75	3282.50
SUT	SUT645	-0.399831	0.1958045	0.3458513	8.69	6.60	79.50	85.50
ORF-T	YNL320W	-0.399554	0.2069238	0.3592665	11.05	8.35	95.00	106.00
ORF-T	YOR359W	-0.398996	0.2467529	0.4073121	8.52	6.42	74.75	86.00
ORF-T	YGL017W	-0.398951	0.1186503	0.2429873	33.94	25.75	316.50	339.25
ORF-T	YOR045W	-0.398944	0.1994289	0.3502335	20.10	15.19	170.50	195.25
ORF-T	YLR257W	-0.398702	0.2033054	0.3547554	159.99	121.34	1414.75	1568.25
NUT	NUT0880	-0.398577	0.0928852	0.2034878	58.58	44.49	535.75	560.25
ORF-T	YMR291W	-0.398472	0.1509717	0.2892095	54.79	41.55	495.75	543.25
ORF-T	YPL228W	-0.398422	0.1643956	0.3076202	13.48	10.19	122.00	136.50
ORF-T	YCR012W	-0.398089	0.3165243	0.4821548	3366.21	2554.46	28979.00	32868.75
ORF-T	YPR022C	-0.397629	0.125268	0.2535024	37.84	28.68	342.50	382.00

TABLE S1: Differential expression data for RRP6 RNA-Seq dataset Page 84

Class	Transcript name	RRP6 KO_vs_WT log2_fold_change	RRP6 KO_vs_WT p-value	RRP6 KO_vs_WT FDR	Ave Norm Reads in WT	Ave Norm Reads in RRP6	Average RAW read counts in WT	Average RAW read counts in RRP6
ORF-T	YCR069W	-0.397521	0.1944018	0.3443436	19.95	15.07	173.50	203.00
ORF-T	YGR171C	-0.397298	0.1554616	0.2956204	25.07	18.98	221.75	251.50
ORF-T	YNR057C	-0.397253	0.2324934	0.3905486	9.55	7.30	95.25	97.25
ORF-T	YBL106C	-0.39714	0.1468393	0.2842087	24.95	18.97	237.00	252.00
ORF-T	YHR006W	-0.397128	0.1123396	0.2336972	70.99	53.94	673.00	714.00
ORF-T	YGR080W	-0.397038	0.291169	0.4551462	7.88	5.93	68.50	79.50
AST	AS_YDL094C	-0.397032	0.2248136	0.3814	6.84	5.23	64.25	67.00
ORF-T	YJL076W	-0.397015	0.1257758	0.2542488	42.73	32.37	368.75	423.25
ORF-T	YGR187C	-0.396983	0.1097696	0.2300326	55.91	42.48	528.00	563.00
NUT	NUT0700	-0.396877	0.4895308	0.633784	1.27	0.92	10.50	13.00
ORF-T	YMR094W	-0.396703	0.1694489	0.3135847	10.42	7.87	96.25	108.25
ORF-T	YMR231W	-0.396643	0.1987828	0.3496469	24.47	18.56	217.00	240.00
ORF-T	YDR310C	-0.396574	0.1544235	0.2940344	24.05	18.21	214.00	242.25
AST	AS_YMR294W-A	-0.396457	0.1362887	0.2702194	151.57	115.23	1412.25	1433.00
ORF-T	YER143W	-0.395673	0.193047	0.3425593	41.93	31.83	372.50	420.00
ORF-T	YDL047W	-0.395547	0.1170554	0.2405025	40.89	31.04	376.25	419.00
ORF-T	YDL045W-A	-0.395443	0.2895064	0.4533907	5.71	4.33	49.50	54.50
SUT	SUT682	-0.395106	0.2426317	0.402219	11.49	8.70	100.00	112.50
ORF-T	YPR137W	-0.395048	0.1828336	0.3310366	26.29	19.97	246.75	270.00
ORF-T	YIL023C	-0.394895	0.1077837	0.2276865	109.06	83.00	1030.00	1084.50
AST	AS_YHL030W-A	-0.394503	0.2951566	0.4598824	4.08	3.06	35.00	40.50
ORF-T	YKL190W	-0.394085	0.0973815	0.2110001	82.19	62.56	760.75	814.75
ORF-T	YAL051W	-0.394055	0.1138397	0.2360114	38.90	29.54	348.50	393.50
ORF-T	YDR066C	-0.393955	0.2098584	0.3626756	4.32	3.28	39.50	43.00
ORF-T	YCR081W	-0.393681	0.1370085	0.2710601	24.25	18.41	214.25	243.50
ORF-T	YPR185W	-0.393676	0.1369828	0.2710601	34.19	25.99	307.50	341.75
ORF-T	YML106W	-0.393618	0.1312078	0.2623636	306.21	233.11	2792.25	2983.25
ORF-T	YGL027C	-0.393595	0.1079343	0.2277045	138.99	105.81	1247.50	1345.00
ORF-T	YPR133C	-0.393522	0.1220692	0.2487263	132.35	100.81	1244.50	1288.00

Class	Transcript name	RRP6 KO_vs_WT log2_fold _change	RRP6 KO_vs_WT p-value	RRP6 KO_vs_WT FDR	Ave Norm Reads in WT	Ave Norm Reads in RRP6	Average RAW read counts in WT	Average RAW read counts in RRP6
NUT	NUT0823	-0.393496	0.0910252	0.2003707	50.30	38.33	462.75	492.00
ORF-T	YDR505C	-0.393379	0.1537781	0.293415	145.25	110.57	1327.25	1442.50
ORF-T	YDR532C	-0.39328	0.1576943	0.2986271	11.09	8.45	99.25	107.00
ORF-T	YMR158W	-0.392966	0.2396721	0.3990809	6.91	5.27	62.00	66.25
AST	AS_YNL097W-A	-0.392719	0.2420825	0.4017709	7.02	5.39	68.00	69.50
ORF-T	YHR181W	-0.392687	0.213947	0.367913	8.88	6.70	76.25	89.25
ORF-T	YFL044C	-0.392249	0.1183721	0.242507	103.67	79.04	938.00	987.00
SUT	SUT430	-0.392193	0.2061638	0.3581937	23.93	18.17	215.75	256.00
AST	AS_YDR464C-A	-0.392159	0.2640856	0.4269744	3.53	2.70	32.50	34.75
ORF-T	YJR142W	-0.391712	0.2385578	0.3980832	27.54	20.99	248.50	268.50
ORF-T	YGL228W	-0.391633	0.1199751	0.2451528	50.99	38.82	442.75	498.00
SUT	SUT241	-0.39159	0.4780209	0.6242376	1.55	1.17	14.75	16.75
ORF-T	YOR315W	-0.391582	0.3880243	0.5489389	16.68	12.66	146.50	182.75
ORF-T	YDR097C	-0.391483	0.1235348	0.2509013	114.03	86.99	1062.25	1101.00
ORF-T	YER166W	-0.391435	0.1040682	0.2220533	182.42	139.05	1647.75	1822.00
ORF-T	YCR004C	-0.391384	0.1517228	0.2902624	214.82	163.82	1973.25	2061.25
ORF-T	YPR123C	-0.391163	0.5237736	0.6620372	1.76	1.39	16.75	15.50
ORF-T	YGL229C	-0.391124	0.3050405	0.4706637	15.74	11.96	134.00	154.25
ORF-T	YOR290C	-0.391066	0.1692105	0.3133735	63.63	48.45	562.25	651.00
ORF-T	YHR119W	-0.39054	0.1164425	0.2397319	53.24	40.59	502.50	554.00
ORF-T	YDR125C	-0.390396	0.2084594	0.3607525	6.44	4.92	58.25	63.00
ORF-T	YJR059W	-0.390215	0.2428492	0.4024472	39.75	30.28	331.00	385.75
ORF-T	YNL076W	-0.390185	0.1376906	0.2718763	18.68	14.23	166.50	183.50
ORF-T	YHR207C	-0.390176	0.1404185	0.2756971	29.70	22.67	281.00	304.50
ORF-T	YJL012C	-0.389768	0.1666358	0.3102703	416.25	317.74	3832.75	4086.50
ORF-T	YPL158C	-0.389741	0.3063964	0.4720705	14.13	10.73	125.75	147.00
ORF-T	YBL001C	-0.389665	0.1593752	0.3008497	25.81	19.71	228.25	244.75
ORF-T	YPR167C	-0.389404	0.2025704	0.3539064	15.73	11.99	140.75	155.50
ORF-T	YDR451C	-0.389292	0.2553423	0.41713	8.33	6.32	74.00	84.00

TABLE S1: Differential expression data for RRP6 RNA-Seq dataset Page 86

Class	Transcript name	RRP6 KO_vs_WT log2_fold _change	RRP6 KO_vs_WT p-value	RRP6 KO_vs_WT FDR	Ave Norm Reads in WT	Ave Norm Reads in RRP6	Average RAW read counts in WT	Average RAW read counts in RRP6
ORF-T	YGR209C	-0.389201	0.3768694	0.5384458	177.77	135.73	1523.50	1689.00
ORF-T	YER044C-A	-0.389186	0.4547101	0.6052182	1.40	1.05	12.00	13.75
AST	AS_YJL211C	-0.389083	0.1736123	0.3192344	24.16	18.45	219.00	235.25
ORF-T	YJL167W	-0.38898	0.1648454	0.3080617	194.23	148.33	1782.25	1921.00
ORF-T	YDL213C	-0.388893	0.1395445	0.2744084	19.82	15.11	179.25	200.75
AST	AS_YNL105W	-0.388467	0.2583961	0.4202688	4.76	3.64	43.50	47.00
ORF-T	YDR383C	-0.388415	0.1999502	0.3509242	7.76	5.96	71.75	76.00
ORF-T	YAL042W	-0.388359	0.1384196	0.272995	362.49	276.98	3375.75	3561.00
ORF-T	YDR062W	-0.38833	0.156198	0.2965733	494.01	377.41	4505.50	4938.25
ORF-T	YLR186W	-0.388243	0.1353008	0.2687891	28.96	22.08	260.50	295.25
NUT	NUT0988	-0.38819	0.4128574	0.5699314	2.64	2.00	23.75	27.00
ORF-T	YIL088C	-0.388129	0.1775365	0.3242083	127.67	97.56	1151.00	1231.50
AST	AS_YJR004C	-0.388004	0.3844965	0.5458232	7.51	5.72	70.75	80.50
ORF-T	YKL207W	-0.38787	0.1081993	0.2279829	127.88	97.77	1170.75	1242.00
ORF-T	YHR028C	-0.387852	0.1515327	0.2900637	176.90	135.22	1583.75	1692.50
ORF-T	YOR130C	-0.387634	0.1210682	0.247036	21.99	16.83	201.25	215.50
ORF-T	YOR036W	-0.387629	0.1892898	0.3384994	29.98	22.92	267.50	292.00
ORF-T	YOL034W	-0.387505	0.1114768	0.2326246	29.07	22.24	266.25	288.50
ORF-T	YGL137W	-0.387401	0.1839061	0.3322317	562.83	430.29	5250.00	5645.50
ORF-T	YER156C	-0.387367	0.1159193	0.239094	138.97	106.27	1271.00	1361.25
ORF-T	YIL157C	-0.387343	0.3401091	0.5045844	3.11	2.39	28.75	30.25
ORF-T	YDL124W	-0.3873	0.4305762	0.5850082	48.03	36.71	393.50	446.25
ORF-T	YKR006C	-0.387251	0.1789817	0.3260209	29.15	22.26	259.25	288.50
ORF-T	YER026C	-0.387223	0.1828514	0.3310366	241.66	184.82	2306.50	2383.75
ORF-T	YBL036C	-0.387069	0.0943106	0.2060592	43.27	33.09	398.25	435.00
ORF-T	YPL110C	-0.386659	0.1052265	0.2238066	79.32	60.69	738.75	800.50
ORF-T	YBL037W	-0.386585	0.1304886	0.2614438	41.67	31.83	375.00	420.50
ORF-T	YHR147C	-0.386451	0.2023159	0.3536407	38.18	29.21	340.25	363.75
ORF-T	YJL054W	-0.386321	0.1407862	0.2763652	40.33	30.80	351.25	400.00

Class	Transcript name	RRP6 KO_vs_WT log2_fold _change	RRP6 KO_vs_WT p-value	RRP6 KO_vs_WT FDR	Ave Norm Reads in WT	Ave Norm Reads in RRP6	Average RAW read counts in WT	Average RAW read counts in RRP6
AST	AS_YEL020C-B	-0.385873	0.178878	0.3259368	12.78	9.81	117.50	124.50
ORF-T	YHR175W-A	-0.385725	0.3972585	0.5581656	2.13	1.59	18.25	21.50
ORF-T	YML029W	-0.385355	0.2616493	0.4240553	160.12	122.57	1394.50	1557.25
ORF-T	YGR224W	-0.385252	0.5478727	0.6800528	6.42	4.91	55.50	61.50
ORF-T	YPL096W	-0.384779	0.175088	0.3210743	14.50	11.09	132.00	145.75
ORF-T	YOR012W	-0.384768	0.3458261	0.5107367	4.15	3.15	38.00	42.75
ORF-T	YPR169W-A	-0.384432	0.205597	0.3575168	9.26	7.04	80.25	92.00
ORF-T	YHL038C	-0.384105	0.2029005	0.3542941	24.05	18.41	219.75	244.50
ORF-T	YER147C	-0.383881	0.1797664	0.3270956	14.67	11.28	139.75	148.50
ORF-T	YMR219W	-0.38386	0.1369665	0.2710601	65.76	50.38	587.50	649.75
sn/snoRNA	SNR32	-0.383846	0.2510801	0.4123025	1552.58	1189.88	13373.50	14940.75
ORF-T	YLR027C	-0.383809	0.1872816	0.3363397	444.78	340.83	4090.75	4664.00
ORF-T	YOR335C	-0.38359	0.1403431	0.2756026	949.20	727.62	8974.75	9540.25
SUT	SUT651	-0.383527	0.2785343	0.4422944	4.70	3.54	40.25	47.50
ORF-T	YMR138W	-0.383466	0.2242833	0.3809453	7.79	5.94	71.50	80.25
ORF-T	YBR118W	-0.383388	0.2193823	0.3753492	2869.33	2199.73	26055.75	28315.75
ORF-T	YOR350C	-0.383163	0.1284928	0.2586269	26.78	20.51	244.75	273.50
AST	AS_YGR213C	-0.383106	0.3626911	0.5264685	2.05	1.55	18.75	21.00
AST	AS_YPR059C	-0.38307	0.1344838	0.2674822	25.48	19.57	240.75	257.00
AST	AS_YKL183C-A	-0.382729	0.2721447	0.435234	3.41	2.65	32.50	34.25
ORF-T	YDL022W	-0.382455	0.3036379	0.4693262	42.02	32.16	343.00	420.75
ORF-T	YOL102C	-0.382286	0.1451142	0.2818978	17.97	13.77	164.25	180.75
ORF-T	YPR115W	-0.382243	0.2668491	0.4296854	91.25	70.00	802.25	883.00
ORF-T	YJL044C	-0.382017	0.0926448	0.2031377	74.67	57.33	699.25	748.75
ORF-T	YNL244C	-0.381963	0.1399384	0.2750758	42.76	32.76	386.75	441.50
ORF-T	YOL113W	-0.381689	0.14229	0.2784878	84.48	64.83	787.75	873.50
AST	AS_YMR095C	-0.381678	0.2456933	0.4062259	6.24	4.79	59.50	64.75
ORF-T	YHR001W-A	-0.38165	0.3675179	0.52989	16.45	12.60	139.75	157.75
ORF-T	YHR200W	-0.381524	0.1224503	0.2491503	102.34	78.54	922.75	1023.50

Class	Transcript name	RRP6 KO_vs_WT log2_fold _change	RRP6 KO_vs_WT p-value	RRP6 KO_vs_WT FDR	Ave Norm Reads in WT	Ave Norm Reads in RRP6	Average RAW read counts in WT	Average RAW read counts in RRP6
ORF-T	YDL004W	-0.381294	0.3162504	0.481878	72.04	55.27	615.00	704.75
ORF-T	YLR207W	-0.381254	0.1157475	0.2390237	82.30	63.19	753.25	817.50
ORF-T	YMR125W	-0.381029	0.1588906	0.3001507	298.33	229.09	2756.00	3002.50
ORF-T	YDR434W	-0.380857	0.1342937	0.2672566	157.85	121.26	1468.25	1562.75
ORF-T	YGR264C	-0.380827	0.1259877	0.2545501	367.31	282.09	3351.75	3689.50
ORF-T	YLR177W	-0.380819	0.3103226	0.4757228	72.61	55.75	617.50	698.25
ORF-T	YBR154C	-0.380505	0.0960279	0.2089526	100.44	77.15	920.25	1009.75
ORF-T	YPR165W	-0.380447	0.1373667	0.2714657	302.03	232.06	2770.25	2951.50
ORF-T	YDR375C	-0.38041	0.2072402	0.3595063	28.17	21.63	254.25	279.00
ORF-T	YMR088C	-0.380253	0.1149644	0.2380007	107.84	82.90	989.75	1053.00
ORF-T	YOL036W	-0.38015	0.1956249	0.34566	22.48	17.22	194.00	224.00
ORF-T	YPL015C	-0.380126	0.1458924	0.2829191	40.66	31.18	352.00	402.00
ORF-T	YHL018W	-0.38	0.2834105	0.4471776	3.27	2.54	31.25	33.25
ORF-T	YGL077C	-0.379883	0.16908	0.3131894	151.61	116.55	1439.00	1524.75
NUT	NUT1201	-0.379847	0.2777571	0.4415944	4.96	3.75	42.75	50.25
ORF-T	YKR062W	-0.379727	0.1294067	0.2599851	28.60	22.00	261.50	283.00
ORF-T	YDL078C	-0.379549	0.150333	0.2884791	125.29	96.33	1161.00	1241.75
ORF-T	YMR174C	-0.379536	0.3261249	0.4907448	2.30	1.77	20.25	22.00
ORF-T	YDR205W	-0.379289	0.2465517	0.4071777	11.73	8.96	100.75	117.25
ORF-T	YDR379W	-0.379191	0.1377185	0.2718781	29.02	22.30	263.75	289.75
ORF-T	YJR006W	-0.379	0.1016835	0.2181387	39.21	30.16	365.75	399.25
ORF-T	YDL133W	-0.378812	0.1068164	0.2264024	50.54	38.90	467.00	502.00
ORF-T	YDL099W	-0.378735	0.1862284	0.3349852	20.79	15.98	185.50	205.50
ORF-T	YJR134C	-0.378676	0.1444246	0.2810997	22.89	17.58	209.25	234.00
ORF-T	YBR245C	-0.378653	0.0946328	0.2065981	76.22	58.62	703.00	771.50
ORF-T	YLR268W	-0.37863	0.2594952	0.4216483	10.37	7.94	93.50	106.50
ORF-T	YOL146W	-0.378521	0.1422004	0.2784878	29.87	23.02	274.00	291.50
ORF-T	YDR266C	-0.378364	0.164722	0.3079453	19.58	14.99	173.25	200.75
ORF-T	YHR219W	-0.37827	0.3463074	0.5112229	2.47	1.92	23.25	24.75

Class	Transcript name	RRP6 KO_vs_WT log2_fold _change	RRP6 KO_vs_WT p-value	RRP6 KO_vs_WT FDR	Ave Norm Reads in WT	Ave Norm Reads in RRP6	Average RAW read counts in WT	Average RAW read counts in RRP6
ORF-T	YBL113C	-0.378255	0.236243	0.3953326	11.33	8.76	107.25	113.00
AST	AS_YKR089C	-0.37825	0.4897016	0.6339043	1.50	1.14	14.25	16.25
ORF-T	YDR208W	-0.378127	0.1237496	0.2512364	44.85	34.45	395.25	450.50
ORF-T	YDR312W	-0.378029	0.1081028	0.2278718	41.03	31.61	386.25	411.75
ORF-T	YOL139C	-0.377977	0.1668026	0.3103391	357.69	275.26	3209.25	3464.50
ORF-T	YOR368W	-0.377973	0.1597049	0.3014157	25.19	19.41	229.50	246.25
ORF-T	YGR116W	-0.377915	0.1697074	0.3138611	279.06	214.74	2579.25	2834.50
ORF-T	YPR148C	-0.377817	0.1683834	0.3124789	54.22	41.71	495.50	551.25
ORF-T	YLR225C	-0.377435	0.1744455	0.3202455	30.92	23.77	282.50	320.50
ORF-T	YJR147W	-0.377386	0.2056972	0.3576294	13.99	10.72	124.00	142.25
ORF-T	YDR121W	-0.377343	0.1922288	0.3418154	6.85	5.23	61.25	70.00
ORF-T	YGR020C	-0.377168	0.1722901	0.3172704	124.37	95.79	1149.25	1214.75
ORF-T	YMR150C	-0.377131	0.1509339	0.289192	23.45	18.08	216.50	232.75
SUT	SUT426	-0.376852	0.3479339	0.5129481	3.05	2.35	27.25	29.50
ORF-T	YPR069C	-0.376762	0.1490291	0.2869051	348.45	268.39	3256.75	3493.25
ORF-T	YFR040W	-0.376639	0.1731461	0.3186722	103.49	79.69	915.25	1023.00
ORF-T	YHR062C	-0.376622	0.1612654	0.3034522	26.28	20.26	249.00	267.75
ORF-T	YER162C	-0.376397	0.1671678	0.3108229	31.01	23.92	280.50	300.75
ORF-T	YBL102W	-0.376369	0.1267753	0.2557316	57.47	44.27	520.00	568.50
ORF-T	YML069W	-0.376209	0.1172672	0.2408887	111.00	85.57	1032.50	1096.75
ORF-T	YGL093W	-0.376196	0.1538929	0.2935737	62.46	48.17	574.50	608.00
ORF-T	YPR098C	-0.376095	0.1934148	0.3429568	22.43	17.23	197.50	227.75
SRT	SRT246	-0.376064	0.3679128	0.530237	1.72	1.34	16.00	17.25
ORF-T	YMR200W	-0.375649	0.1371061	0.2712001	197.26	152.07	1824.75	1962.75
ORF-T	YPR140W	-0.375569	0.1984845	0.3491831	31.06	23.98	284.25	300.25
ORF-T	YOR026W	-0.375567	0.1521704	0.290898	29.60	22.79	273.00	307.00
ORF-T	YER151C	-0.375479	0.1388151	0.2733721	40.25	30.96	362.00	415.25
ORF-T	YBR010W	-0.374948	0.1873635	0.3363947	798.51	615.74	7247.00	8068.00
AST	AS_YML009C-A	-0.374893	0.1858514	0.3345457	19.90	15.40	183.75	192.75

Class	Transcript name	RRP6 KO_vs_WT log2_fold _change	RRP6 KO_vs_WT p-value	RRP6 KO_vs_WT FDR	Ave Norm Reads in WT	Ave Norm Reads in RRP6	Average RAW read counts in WT	Average RAW read counts in RRP6
ORF-T	YBR015C	-0.374622	0.1330798	0.2653176	147.45	113.74	1343.00	1459.25
AST	AS_YLR387C	-0.37461	0.3327456	0.4970237	2.06	1.61	19.00	20.50
ORF-T	YHR108W	-0.374358	0.1541295	0.2936964	64.62	49.81	584.75	660.50
ORF-T	YPL007C	-0.374342	0.129557	0.2601003	47.44	36.58	435.25	480.75
sn/snoRNA	SNR55	-0.3743	0.296847	0.4615555	951.65	734.16	8163.25	9338.00
ORF-T	YDR276C	-0.374227	0.1996341	0.3505328	48.88	37.67	423.00	484.50
ORF-T	YDR272W	-0.374225	0.1532611	0.2925946	62.61	48.34	564.50	604.00
AST	AS_YNL245C	-0.374118	0.2818805	0.4454604	6.77	5.24	64.75	69.50
AST	AS_YOR055W	-0.373977	0.2903513	0.4542201	12.14	9.38	108.75	117.75
ORF-T	YGR095C	-0.373922	0.2126405	0.3661657	10.43	8.01	92.25	105.00
ORF-T	YNL314W	-0.373918	0.2377493	0.3973258	5.91	4.51	52.25	60.50
ORF-T	YPL227C	-0.373756	0.2143083	0.3684087	8.65	6.69	78.75	85.50
ORF-T	YHR039C-A	-0.373551	0.208285	0.3606467	43.24	33.28	364.50	442.75
ORF-T	YDR348C	-0.373404	0.10511	0.2236162	43.48	33.54	387.00	431.50
ORF-T	YNR043W	-0.373368	0.1023539	0.2193702	66.21	51.08	591.75	662.75
ORF-T	YLR208W	-0.373367	0.1484773	0.2862254	238.53	184.18	2202.00	2345.00
ORF-T	YJR105W	-0.373054	0.1927365	0.3424766	453.22	350.00	4173.75	4366.75
ORF-T	YHR161C	-0.372746	0.2340374	0.392619	82.75	63.93	763.50	813.75
ORF-T	YFR037C	-0.372668	0.1612182	0.30342	142.67	110.23	1348.25	1435.50
ORF-T	YIL122W	-0.372653	0.2424183	0.4019974	8.37	6.42	74.50	86.50
ORF-T	YKR100C	-0.372447	0.1697228	0.3138611	19.11	14.69	168.25	197.00
SUT	SUT415	-0.372438	0.3469633	0.5119097	1.86	1.46	17.00	18.50
ORF-T	YJL154C	-0.372425	0.1505356	0.2887032	71.73	55.38	642.00	723.75
NUT	NUT0232	-0.372149	0.3210301	0.4856387	4.65	3.58	42.25	46.75
ORF-T	YPL202C	-0.372144	0.3199668	0.4846877	4.81	3.68	43.50	50.00
ORF-T	YDR238C	-0.371915	0.157962	0.2989095	402.43	310.96	3696.50	4109.50
SUT	SUT413	-0.371886	0.4638519	0.6132794	1.04	0.81	10.25	11.25
ORF-T	YHR050W	-0.371631	0.1221998	0.2487731	162.09	125.32	1488.50	1597.75
ORF-T	YNR022C	-0.371608	0.2699422	0.4329723	9.57	7.35	84.00	97.00

TABLE S1: Differential expression data for RRP6 RNA-Seq dataset Page 91

Class	Transcript name	RRP6 KO_vs_WT log2_fold _change	RRP6 KO_vs_WT p-value	RRP6 KO_vs_WT FDR	Ave Norm Reads in WT	Ave Norm Reads in RRP6	Average RAW read counts in WT	Average RAW read counts in RRP6
ORF-T	YBR073W	-0.371529	0.1843771	0.3326842	194.85	150.68	1815.50	1894.50
ORF-T	YNL067W	-0.371443	0.1648438	0.3080617	274.93	212.51	2440.75	2726.25
ORF-T	YGL248W	-0.371335	0.1833852	0.3316566	34.12	26.38	307.25	338.25
ORF-T	YBR035C	-0.371237	0.1876991	0.3367792	58.06	44.84	502.50	580.50
ORF-T	YOR017W	-0.371207	0.1078396	0.2276865	44.75	34.64	418.50	447.00
ORF-T	YJL111W	-0.370744	0.1252118	0.2534677	189.43	146.51	1740.75	1913.00
ORF-T	YOL070C	-0.370698	0.184409	0.3326842	56.20	43.45	525.50	582.50
ORF-T	YGR130C	-0.370575	0.2182479	0.3738426	53.42	41.28	457.00	525.50
ORF-T	YOR294W	-0.370492	0.1633752	0.3061653	57.70	44.69	556.75	590.25
ORF-T	YDR103W	-0.370296	0.1337294	0.2664021	26.49	20.47	235.75	266.25
NUT	NUT0348	-0.370261	0.2678944	0.4307734	1553.00	1201.46	13377.50	15091.50
ORF-T	YMR027W	-0.370078	0.1765654	0.3230785	101.75	78.73	934.00	1019.00
ORF-T	YJR066W	-0.36995	0.1572423	0.298051	80.94	62.59	721.50	820.25
ORF-T	YPL184C	-0.369866	0.1225292	0.2492103	71.23	55.11	657.75	730.25
ORF-T	YDR120C	-0.369611	0.1304546	0.2614277	127.13	98.44	1205.00	1287.00
AST	AS_YNL089C	-0.369608	0.1592561	0.3006812	40.84	31.63	392.50	423.00
ORF-T	YLL050C	-0.369527	0.2981534	0.4628354	143.21	110.85	1280.75	1403.00
AST	AS_YNL303W	-0.369205	0.2892218	0.4531562	8.60	6.62	77.75	90.50
ORF-T	YHL024W	-0.369028	0.3891776	0.5498762	16.47	12.76	143.00	157.75
ORF-T	YPL226W	-0.368858	0.1667942	0.3103391	275.01	212.95	2550.25	2860.50
ORF-T	YLR342W	-0.368713	0.1783171	0.3252214	1820.86	1410.20	16675.00	18626.00
ORF-T	YLR266C	-0.368399	0.1195677	0.2444689	46.55	36.09	429.25	467.50
ORF-T	YPR170W-B	-0.368378	0.1774517	0.324112	18.28	14.10	157.50	183.00
ORF-T	YLR078C	-0.368366	0.1453311	0.2822104	32.18	24.98	295.25	315.00
ORF-T	YPR095C	-0.367598	0.1036212	0.2213337	71.43	55.35	647.25	722.25
ORF-T	YIL035C	-0.367559	0.1097416	0.2300326	54.90	42.58	510.25	555.50
ORF-T	YIL128W	-0.367479	0.1385494	0.2731975	38.85	30.04	340.00	398.00
ORF-T	YHL025W	-0.367469	0.1123655	0.2336972	61.38	47.64	576.00	610.50
ORF-T	YEL027W	-0.367265	0.1432844	0.2797605	120.12	93.06	1063.25	1233.25

Class	Transcript name	RRP6 KO_vs_WT log2_fold _change	RRP6 KO_vs_WT p-value	RRP6 KO_vs_WT FDR	Ave Norm Reads in WT	Ave Norm Reads in RRP6	Average RAW read counts in WT	Average RAW read counts in RRP6
AST	AS_YHR165W-A	-0.367026	0.3157992	0.4813361	6.87	5.26	59.75	71.75
ORF-T	YAL029C	-0.366965	0.1399922	0.2750976	144.15	111.76	1329.75	1478.50
ORF-T	YLL029W	-0.366862	0.1605165	0.3026643	149.28	115.80	1382.25	1483.50
ORF-T	YLR021W	-0.366759	0.2511787	0.4123025	8.61	6.63	77.25	90.25
ORF-T	YKL143W	-0.366734	0.1474401	0.2847159	85.20	66.14	815.50	865.25
ORF-T	YMR001C	-0.366691	0.2209806	0.377178	64.33	49.84	586.00	686.75
ORF-T	YPR155C	-0.366607	0.2511849	0.4123025	35.94	27.87	326.00	357.50
ORF-T	YDR504C	-0.366339	0.2113167	0.3645086	8.02	6.21	71.50	80.50
ORF-T	YJL212C	-0.366093	0.4932135	0.6369833	36.09	27.99	311.25	353.75
ORF-T	YPR058W	-0.366045	0.1075702	0.2274714	55.88	43.36	519.25	573.00
ORF-T	YAL010C	-0.366016	0.2496388	0.4110643	12.22	9.45	111.00	126.25
ORF-T	YGR289C	-0.365846	0.4349376	0.5891083	2.02	1.58	19.25	21.00
ORF-T	YML118W	-0.36568	0.3096015	0.4752659	6.53	5.06	58.75	65.50
ORF-T	YPR023C	-0.365661	0.1422599	0.2784878	50.94	39.56	470.50	511.25
ORF-T	YBR276C	-0.365613	0.1610816	0.3033776	185.60	144.11	1732.50	1832.75
ORF-T	YKR025W	-0.365557	0.1735714	0.3192219	55.03	42.77	529.25	560.00
ORF-T	YIL061C	-0.365402	0.3195253	0.4845481	2.72	2.10	24.50	27.25
ORF-T	YDL209C	-0.365272	0.2513546	0.4124081	10.76	8.38	105.75	113.75
ORF-T	YML009W-B	-0.365034	0.1973186	0.3477387	25.16	19.59	231.00	244.25
ORF-T	YBL088C	-0.364868	0.1328766	0.2650697	73.51	57.02	667.00	770.25
ORF-T	YGR015C	-0.364852	0.2225837	0.3786966	8.06	6.27	75.25	82.25
ORF-T	YOR235W	-0.364682	0.3480682	0.5129961	4.04	3.14	36.25	40.25
ORF-T	YDL121C	-0.36468	0.1663721	0.3099956	61.07	47.46	549.25	593.50
ORF-T	YGL059W	-0.364643	0.3852624	0.5461811	17.12	13.27	149.75	176.00
ORF-T	YDL233W	-0.364564	0.1788508	0.3259368	15.36	11.85	133.50	157.50
SUT	SUT555	-0.364562	0.3789032	0.540263	2.33	1.83	23.00	24.75
ORF-T	YGR275W	-0.364533	0.2139912	0.3679262	17.44	13.48	155.25	182.50
ORF-T	YMR178W	-0.364528	0.134462	0.2674822	48.00	37.27	436.25	485.25
ORF-T	YJR062C	-0.364494	0.240676	0.4004251	32.37	25.11	285.25	325.00

Class	Transcript name	RRP6 KO_vs_WT log2_fold _change	RRP6 KO_vs_WT p-value	RRP6 KO_vs_WT FDR	Ave Norm Reads in WT	Ave Norm Reads in RRP6	Average RAW read counts in WT	Average RAW read counts in RRP6
ORF-T	YDL029W	-0.364428	0.2987552	0.4635086	193.18	150.04	1741.50	1958.75
ORF-T	YKR095W-A	-0.364392	0.256233	0.417964	8.06	6.27	70.00	76.50
ORF-T	YPL189C-A	-0.364391	0.2137841	0.3677583	72.58	56.38	641.75	703.75
ORF-T	YDR056C	-0.36429	0.1546719	0.2943963	42.19	32.68	368.00	437.50
ORF-T	YBL056W	-0.364221	0.1682338	0.3123103	200.16	155.52	1812.25	1977.25
ORF-T	YKL134C	-0.364201	0.2642398	0.4270634	42.45	32.96	397.50	450.50
ORF-T	YDL056W	-0.364197	0.1840703	0.3324078	29.34	22.73	264.25	307.75
ORF-T	YDR296W	-0.364132	0.1586782	0.2999833	32.47	25.20	290.25	326.50
ORF-T	YHR097C	-0.363361	0.2682611	0.4311381	19.07	14.79	166.50	193.75
ORF-T	YER141W	-0.363242	0.2104682	0.3634927	111.66	86.80	1010.75	1121.25
ORF-T	YDR443C	-0.363161	0.183633	0.3319748	28.64	22.25	253.25	283.25
ORF-T	YGL207W	-0.362698	0.1494567	0.2874043	312.88	243.34	2913.75	3192.50
ORF-T	YPL203W	-0.362545	0.3109474	0.4763891	67.41	52.42	589.25	664.00
ORF-T	YPR020W	-0.362307	0.2752211	0.4388313	40.35	31.35	344.25	396.00
ORF-T	YMR299C	-0.36218	0.1770355	0.3235278	20.73	16.07	181.75	212.00
ORF-T	YNL005C	-0.361654	0.1513471	0.2897635	65.38	50.90	584.25	642.75
ORF-T	YMR112C	-0.361556	0.2544645	0.4160889	4.92	3.84	44.50	49.00
ORF-T	YLL040C	-0.361369	0.3576579	0.5214897	162.31	126.32	1443.50	1680.00
ORF-T	YNL192W	-0.361277	0.1912226	0.3405066	444.53	346.10	4334.50	4624.25
ORF-T	YGL185C	-0.361038	0.1857241	0.334436	16.08	12.53	146.50	160.50
ORF-T	YML113W	-0.361022	0.2387094	0.3982043	21.42	16.71	209.00	223.50
ORF-T	YJL201W	-0.360875	0.2466183	0.407221	10.35	7.99	88.00	104.75
ORF-T	YPR072W	-0.360839	0.1425884	0.2787633	41.20	32.02	371.00	428.25
ORF-T	YDR378C	-0.360805	0.1698498	0.3140384	100.17	78.02	906.25	987.25
ORF-T	YDR284C	-0.360803	0.2155444	0.370155	58.10	45.22	524.25	588.00
ORF-T	YJR109C	-0.36077	0.3568087	0.5207031	192.20	149.63	1807.50	2232.50
ORF-T	YDL027C	-0.360717	0.2721723	0.435234	14.08	10.93	122.50	140.00
SUT	SUT747	-0.360679	0.2376021	0.3972114	10.50	8.14	90.25	103.00
ORF-T	YPR180W	-0.360553	0.1489727	0.2869051	32.81	25.57	300.50	329.25

Class	Transcript name	RRP6 KO_vs_WT log2_fold _change	RRP6 KO_vs_WT p-value	RRP6 KO_vs_WT FDR	Ave Norm Reads in WT	Ave Norm Reads in RRP6	Average RAW read counts in WT	Average RAW read counts in RRP6
ORF-T	YKL126W	-0.360547	0.1922725	0.3418328	275.27	214.44	2550.50	2732.25
ORF-T	YGR286C	-0.36023	0.2641917	0.4270634	96.60	75.25	851.75	948.00
ORF-T	YKR081C	-0.360185	0.1795382	0.3267985	73.19	57.03	675.75	750.00
ORF-T	YLR371W	-0.359808	0.1372282	0.2712818	83.22	64.81	760.25	866.00
ORF-T	YPL023C	-0.359805	0.1768084	0.3231712	176.85	137.89	1681.75	1755.50
ORF-T	YKL129C	-0.359754	0.2701192	0.4331874	60.68	47.24	543.50	636.00
ORF-T	YDR088C	-0.359724	0.184223	0.332624	13.26	10.36	123.75	135.00
NUT	NUT0476	-0.359676	0.5120676	0.6524753	1.50	1.15	14.25	16.50
ORF-T	YMR195W	-0.359651	0.2948547	0.459483	12.38	9.60	103.75	122.75
SUT	SUT742	-0.359611	0.4898318	0.6339148	1.49	1.14	13.50	16.00
ORF-T	YLR290C	-0.359395	0.2560551	0.4179443	21.58	16.79	194.00	220.00
ORF-T	YFL005W	-0.359275	0.1660134	0.3096753	268.92	209.67	2448.75	2656.00
ORF-T	YML105C	-0.359273	0.1110021	0.2319406	40.47	31.55	370.75	411.00
ORF-T	YDL093W	-0.359137	0.124062	0.2517693	48.10	37.48	441.25	497.25
ORF-T	YLR199C	-0.358966	0.2306224	0.3885708	11.83	9.18	105.00	122.75
ORF-T	YOL067C	-0.358854	0.1278021	0.2575967	66.92	52.22	627.00	684.75
ORF-T	YNL151C	-0.358799	0.1211036	0.2470582	46.99	36.71	443.00	471.75
ORF-T	YDL226C	-0.358772	0.1299729	0.2606177	87.53	68.25	809.25	902.25
AST	AS_YML101C-A	-0.358674	0.1947933	0.3447342	21.75	17.00	199.75	216.75
ORF-T	YMR160W	-0.358616	0.3924884	0.5534685	7.22	5.61	62.75	72.25
ORF-T	YOR110W	-0.358514	0.1616579	0.3038506	22.39	17.51	211.00	226.25
ORF-T	YDR052C	-0.358193	0.1611054	0.3033776	18.79	14.67	173.50	191.75
ORF-T	YDR464W	-0.35814	0.1471101	0.2842891	24.35	18.99	223.25	249.25
ORF-T	YAL058W	-0.357873	0.1379816	0.2722909	51.57	40.26	479.75	525.25
ORF-T	YOR046C	-0.357812	0.1471345	0.2842891	200.70	156.65	1856.50	2004.75
ORF-T	YLR118C	-0.357786	0.2000634	0.3510421	20.21	15.73	181.25	207.25
ORF-T	YBL058W	-0.357731	0.2929126	0.4573047	108.38	84.57	967.00	1084.50
ORF-T	YJR039W	-0.357593	0.3073949	0.4731029	30.56	23.83	272.75	312.75
ORF-T	YGR135W	-0.357396	0.1383482	0.2729075	115.15	89.87	1044.00	1170.25

TABLE S1: Differential expression data for RRP6 RNA-Seq dataset Page 95

Class	Transcript name	RRP6 KO_vs_WT log2_fold _change	RRP6 KO_vs_WT p-value	RRP6 KO_vs_WT FDR	Ave Norm Reads in WT	Ave Norm Reads in RRP6	Average RAW read counts in WT	Average RAW read counts in RRP6
ORF-T	YLR392C	-0.357215	0.2188773	0.3747298	37.82	29.56	343.25	370.50
ORF-T	YLR067C	-0.357108	0.1129863	0.2346281	49.32	38.53	459.75	504.25
AST	AS_YML107C	-0.356918	0.2682856	0.4311381	17.14	13.36	154.25	177.75
ORF-T	YGL190C	-0.356837	0.1932899	0.3428558	96.55	75.39	900.75	998.25
ORF-T	YNL188W	-0.356788	0.2971753	0.4618859	7.29	5.64	64.75	76.25
ORF-T	YPL010W	-0.356762	0.156656	0.2971072	89.64	69.98	811.75	915.75
ORF-T	YKL044W	-0.356456	0.435514	0.5893348	1.85	1.38	15.50	19.25
ORF-T	YDL219W	-0.356291	0.1719395	0.3168564	35.40	27.67	320.75	353.00
SUT	SUT485	-0.356114	0.2999484	0.4647634	15.70	12.29	143.00	151.75
ORF-T	YNL059C	-0.356073	0.2312159	0.3892455	37.66	29.40	347.50	393.00
ORF-T	YOR302W	-0.356062	0.4186125	0.5744813	2.31	1.77	19.25	22.75
ORF-T	YJR125C	-0.35594	0.2318608	0.3897175	106.83	83.49	985.50	1070.25
ORF-T	YOR253W	-0.355814	0.1561046	0.2965075	75.96	59.34	696.50	782.75
ORF-T	YER004W	-0.355665	0.151325	0.2897635	69.20	54.07	629.25	700.00
ORF-T	YNL014W	-0.35566	0.2601858	0.4224302	16.32	12.77	150.75	167.00
NUT	NUT1231	-0.355506	0.2053024	0.3571893	718.14	561.29	6251.25	7005.50
ORF-T	YKL144C	-0.355468	0.2392131	0.398649	16.25	12.67	152.25	175.25
sn/snoRNA	SNR38*	-0.355406	0.3403987	0.5049397	1137.75	889.32	9946.75	11110.00
ORF-T	YCR083W	-0.355157	0.2036598	0.3550667	17.52	13.71	156.75	173.25
ORF-T	YEL044W	-0.355104	0.1294511	0.2599851	81.43	63.72	766.50	824.00
AST	AS_YIL020C-A	-0.354876	0.1566931	0.2971218	34.32	26.91	326.75	342.50
ORF-T	YBL105C	-0.354812	0.1479752	0.2856397	114.49	89.48	1024.00	1176.00
AST	AS_YMR087W	-0.354792	0.2979051	0.4625924	3.48	2.74	32.00	35.00
ORF-T	YNL313C	-0.354596	0.1615506	0.3038187	62.74	49.12	604.00	649.75
ORF-T	YKL057C	-0.354536	0.1327494	0.2649208	61.18	47.78	543.50	634.00
ORF-T	YPL244C	-0.354516	0.1421661	0.2784878	84.04	65.75	770.25	845.75
ORF-T	YOL136C	-0.35447	0.3191881	0.4842323	17.72	13.78	156.75	195.00
ORF-T	YDR456W	-0.354384	0.1902029	0.33941	66.84	52.21	585.50	692.00
AST	AS_YLR358C	-0.354326	0.2511824	0.4123025	10.85	8.42	94.25	112.00

Class	Transcript name	RRP6 KO_vs_WT log2_fold _change	RRP6 KO_vs_WT p-value	RRP6 KO_vs_WT FDR	Ave Norm Reads in WT	Ave Norm Reads in RRP6	Average RAW read counts in WT	Average RAW read counts in RRP6
AST	AS_YMR279C	-0.354319	0.3487614	0.5134175	5.41	4.15	46.00	57.50
ORF-T	YJL156C	-0.354085	0.1489078	0.2868908	32.91	25.77	303.50	335.75
ORF-T	YOR355W	-0.353918	0.1898899	0.3389714	34.31	26.78	299.50	349.75
ORF-T	YOR254C	-0.353871	0.1788747	0.3259368	440.52	344.74	4091.50	4400.50
ORF-T	YDR140W	-0.353858	0.1471171	0.2842891	25.22	19.73	231.50	259.75
ORF-T	YHR052W	-0.35367	0.1489414	0.2869008	42.75	33.40	388.25	451.25
ORF-T	YDR449C	-0.353563	0.2108174	0.3637718	27.99	21.88	255.75	292.25
AST	AS_YER148W-A	-0.353516	0.2061605	0.3581937	16.30	12.73	146.25	166.00
ORF-T	YOR322C	-0.353337	0.1734075	0.319037	24.50	19.14	223.50	256.00
ORF-T	YCL057W	-0.353227	0.2423729	0.4019882	88.83	69.51	794.75	906.50
ORF-T	YJR127C	-0.353217	0.2227799	0.3788386	38.85	30.34	333.00	394.50
ORF-T	YDL142C	-0.352724	0.3212516	0.485725	23.39	18.28	206.75	238.50
AST	AS_YEL050W-A	-0.352662	0.2499121	0.411437	11.61	9.12	107.50	117.25
ORF-T	YAR002C-A	-0.352396	0.1711112	0.3157101	205.49	160.96	1877.25	2080.25
AST	AS_YLR428C	-0.352178	0.2814885	0.4451941	16.47	12.93	150.00	161.25
ORF-T	YNL287W	-0.352091	0.1817906	0.3298266	393.95	308.63	3604.25	4038.50
ORF-T	YMR198W	-0.352088	0.1467526	0.2840954	33.59	26.29	310.75	354.25
ORF-T	YOR158W	-0.352058	0.2707275	0.4338249	21.32	16.69	186.25	210.50
ORF-T	YDR011W	-0.35198	0.2431101	0.4028134	349.96	274.17	3034.75	3479.75
ORF-T	YKR037C	-0.351977	0.1843047	0.3326842	22.52	17.65	202.25	225.75
ORF-T	YMR295C	-0.351956	0.2019383	0.3531032	308.05	241.44	2873.25	2994.25
ORF-T	YER139C	-0.351606	0.3082911	0.4740481	15.95	12.49	143.25	163.75
AST	AS_YDR455C	-0.351506	0.2963692	0.4610594	6.97	5.38	58.25	72.00
SUT	SUT696	-0.351494	0.49921	0.6417108	2.89	2.27	27.25	30.25
ORF-T	YMR079W	-0.351319	0.1417266	0.2779947	110.24	86.40	1004.25	1141.75
ORF-T	YMR252C	-0.351254	0.2315376	0.3896145	12.62	9.88	111.25	126.00
AST	AS_YMR290W-A	-0.350968	0.1615463	0.3038187	17.42	13.68	161.25	178.50
ORF-T	YEL019C	-0.350926	0.2513313	0.4124081	6.53	5.12	61.25	68.25
ORF-T	YNL172W	-0.350906	0.1799571	0.3273836	46.64	36.53	434.75	500.75

Class	Transcript name	RRP6 KO_vs_WT log2_fold _change	RRP6 KO_vs_WT p-value	RRP6 KO_vs_WT FDR	Ave Norm Reads in WT	Ave Norm Reads in RRP6	Average RAW read counts in WT	Average RAW read counts in RRP6
ORF-T	YER177W	-0.350638	0.2391894	0.398649	559.41	438.71	5064.75	5622.75
ORF-T	YER149C	-0.350553	0.2035463	0.3550397	24.67	19.33	221.50	250.75
ORF-T	YGR070W	-0.350478	0.2510284	0.4123025	42.52	33.29	363.75	434.00
ORF-T	YJL209W	-0.350446	0.160754	0.302829	25.47	19.96	233.50	264.00
ORF-T	YGL180W	-0.350381	0.2578693	0.4195473	45.27	35.50	402.00	455.75
ORF-T	YLR249W	-0.350148	0.1965798	0.3466791	9960.67	7814.22	92862.75	101114.75
ORF-T	YGL245W	-0.349839	0.2509889	0.4123025	682.85	535.80	6343.00	7130.00
ORF-T	YDR452W	-0.349497	0.1893966	0.3385246	125.41	98.45	1152.00	1268.00
ORF-T	YAL049C	-0.349353	0.2815341	0.4451941	32.44	25.43	284.25	330.75
ORF-T	YOR150W	-0.34905	0.2815354	0.4451941	8.68	6.77	76.00	88.25
ORF-T	YJL196C	-0.349007	0.222215	0.3784667	137.26	107.79	1240.00	1380.75
AST	AS_YNL146C-A	-0.348777	0.375827	0.5377007	1.30	1.01	11.75	13.25
ORF-T	YPL175W	-0.348637	0.1989234	0.3497113	23.70	18.57	219.00	254.50
ORF-T	YPL096C-A	-0.348258	0.4890735	0.6336653	1.04	0.79	8.75	10.25
ORF-T	YGR252W	-0.348231	0.1909053	0.3401818	35.98	28.25	336.00	381.25
SUT	SUT284	-0.348177	0.3677359	0.5301284	2.93	2.30	27.25	30.75
AST	AS_YKL147C	-0.347924	0.1866031	0.3354797	18.29	14.34	162.25	185.00
ORF-T	YGL210W	-0.347745	0.180562	0.3283064	20.03	15.69	178.50	208.50
ORF-T	YDL058W	-0.347465	0.2338855	0.3924295	87.31	68.62	797.25	892.75
ORF-T	YGR186W	-0.347454	0.219692	0.3756782	101.10	79.44	927.50	1057.75
ORF-T	YAL017W	-0.347418	0.2571668	0.4186745	87.21	68.51	761.00	889.25
AST	AS_YNR062C	-0.347392	0.3296837	0.494207	3.34	2.58	28.75	34.25
ORF-T	YLR221C	-0.347271	0.1738051	0.3194154	31.28	24.61	291.75	322.50
AST	AS_YAL026C-A	-0.347239	0.2935016	0.4580451	3.72	2.91	34.00	39.00
AST	AS_YHR073C-B	-0.34717	0.2627338	0.4254027	6.13	4.81	56.25	63.50
ORF-T	YLR283W	-0.34704	0.28526	0.4487616	8.30	6.52	74.75	83.75
ORF-T	YGL080W	-0.346955	0.1946203	0.3445172	28.14	22.15	258.50	283.25
ORF-T	YKL152C	-0.346899	0.2494626	0.4108412	1795.15	1411.43	15934.00	18565.00
ORF-T	YER090W	-0.34664	0.1962518	0.3462217	81.09	63.71	725.50	850.75

Class	Transcript name	RRP6 KO_vs_WT log2_fold _change	RRP6 KO_vs_WT p-value	RRP6 KO_vs_WT FDR	Ave Norm Reads in WT	Ave Norm Reads in RRP6	Average RAW read counts in WT	Average RAW read counts in RRP6
SUT	SUT610	-0.346236	0.4150118	0.5718454	2.13	1.62	18.75	23.00
AST	AS_YGR114C	-0.34597	0.3052499	0.4706637	8.57	6.73	78.75	89.75
ORF-T	YCL059C	-0.345927	0.2156276	0.3701717	141.62	111.49	1322.75	1409.75
ORF-T	YBL076C	-0.34589	0.1839069	0.3322317	1340.58	1054.80	12277.50	13733.50
ORF-T	YIL078W	-0.345648	0.1903723	0.3395921	1561.99	1229.27	14640.25	15661.25
ORF-T	YBL026W	-0.345553	0.1887111	0.3377309	33.16	26.07	295.25	338.25
ORF-T	YMR225C	-0.34552	0.4271253	0.5822808	8.89	6.96	73.50	89.00
AST	AS_YNL150W	-0.345477	0.1575588	0.2984266	117.25	92.28	1080.50	1209.50
ORF-T	YLR446W	-0.345298	0.5473022	0.6794282	1.51	1.18	12.50	14.75
SUT	SUT214	-0.345111	0.4412364	0.5942336	1.66	1.26	14.75	18.00
AST	AS_YNL109W	-0.345006	0.1910612	0.3403393	19.40	15.26	180.75	203.00
ORF-T	YJR054W	-0.344734	0.1551565	0.2950959	62.17	49.01	587.75	634.50
ORF-T	YPL109C	-0.344701	0.3231604	0.4872532	30.86	24.27	272.00	316.00
ORF-T	YKL065C	-0.344688	0.2464874	0.4071382	85.27	67.17	754.75	829.75
ORF-T	YBR233W	-0.344603	0.3198157	0.484674	3.40	2.63	29.50	34.75
AST	AS_YGR018C	-0.344527	0.281088	0.4449053	17.79	13.99	166.50	189.50
ORF-T	YLR272C	-0.344499	0.175892	0.3222557	112.29	88.42	1059.75	1198.50
ORF-T	YBR057C	-0.344405	0.1991252	0.3498816	16.09	12.64	146.75	169.00
ORF-T	YIL138C	-0.344047	0.1894046	0.3385246	61.09	48.13	570.00	638.00
ORF-T	YKL155C	-0.343885	0.1632074	0.3060215	48.81	38.47	443.75	495.25
ORF-T	YKL087C	-0.343837	0.3536459	0.5181874	9.32	7.33	80.00	91.25
ORF-T	YGL221C	-0.343616	0.1734647	0.3190839	106.73	84.12	995.50	1102.50
ORF-T	YOL025W	-0.343536	0.3443152	0.5087289	5.71	4.44	49.25	59.50
ORF-T	YDR406W	-0.343381	0.283815	0.4474656	74.61	58.80	663.75	747.25
ORF-T	YJL133W	-0.343331	0.2424685	0.4020146	10.31	8.11	90.75	103.50
AST	AS_YOR293C-A	-0.343265	0.3332669	0.4973211	5.96	4.72	54.00	59.50
ORF-T	YER095W	-0.343142	0.2926472	0.4571025	130.24	102.72	1188.25	1261.50
ORF-T	YLR175W	-0.34305	0.1982393	0.3489345	645.85	509.25	6083.25	6444.25
ORF-T	YGL094C	-0.342959	0.1454826	0.2823958	45.84	36.11	405.50	462.75

TABLE S1: Differential expression data for RRP6 RNA-Seq dataset Page 99

Class	Transcript name	RRP6 KO_vs_WT log2_fold _change	RRP6 KO_vs_WT p-value	RRP6 KO_vs_WT FDR	Ave Norm Reads in WT	Ave Norm Reads in RRP6	Average RAW read counts in WT	Average RAW read counts in RRP6
ORF-T	YHR016C	-0.342926	0.3561443	0.5200515	42.69	33.65	381.50	432.00
ORF-T	YOR129C	-0.342849	0.2844383	0.4480147	6.93	5.42	61.00	72.50
SRT	SRT5	-0.342393	0.4517196	0.6030051	5.00	3.96	52.00	57.25
ORF-T	YDL015C	-0.342347	0.2967258	0.4614718	162.82	128.38	1458.50	1708.25
AST	AS_YPL102C	-0.342089	0.3440776	0.5084523	4.24	3.38	41.00	43.75
ORF-T	YDR391C	-0.342046	0.2946155	0.4592522	10.54	8.26	89.25	105.75
ORF-T	YBR052C	-0.341896	0.2894305	0.4533423	75.49	59.55	664.75	755.75
ORF-T	YFL042C	-0.341777	0.2531862	0.4145364	30.08	23.71	273.25	313.50
ORF-T	YHR023W	-0.341603	0.2656754	0.4286338	133.87	105.61	1223.50	1440.00
ORF-T	YLR386W	-0.341454	0.1345433	0.267495	51.94	41.02	487.75	537.50
ORF-T	YLR291C	-0.341287	0.1530528	0.2922524	39.46	31.10	355.75	411.50
ORF-T	YNL206C	-0.341078	0.1480989	0.2857726	56.28	44.48	527.00	570.50
ORF-T	YER027C	-0.341074	0.2258061	0.3826081	44.93	35.44	404.25	462.25
ORF-T	YLR197W	-0.340306	0.2389195	0.3983572	572.98	452.62	5156.25	5665.50
ORF-T	YOL041C	-0.340234	0.2005856	0.3516524	23.69	18.72	219.25	245.25
ORF-T	YGL155W	-0.340202	0.1846111	0.3327927	46.76	36.91	436.50	498.75
ORF-T	YLR357W	-0.340069	0.1848729	0.3330817	34.13	26.88	302.00	359.75
ORF-T	YOR275C	-0.340046	0.1861062	0.3348251	27.73	21.87	248.00	287.00
ORF-T	YDR527W	-0.340032	0.2305436	0.3885029	61.48	48.57	596.00	668.25
ORF-T	YDR180W	-0.339984	0.157045	0.297733	64.96	51.30	596.25	683.00
ORF-T	YBR215W	-0.339961	0.3586512	0.522558	4.63	3.58	39.50	49.00
ORF-T	YBR079C	-0.339897	0.2347294	0.3935181	181.34	143.21	1617.25	1944.00
ORF-T	YCR036W	-0.33973	0.2083668	0.3606541	69.34	54.79	630.00	712.00
ORF-T	YMR135C	-0.339579	0.180399	0.3281283	58.88	46.56	531.50	588.50
ORF-T	YMR266W	-0.339472	0.2016783	0.3527964	192.99	152.50	1799.75	2089.00
ORF-T	YGR170W	-0.339356	0.1970672	0.3474778	36.70	28.97	337.00	389.75
ORF-T	YDL230W	-0.339141	0.147023	0.2842891	38.33	30.35	356.00	389.00
ORF-T	YFR028C	-0.339113	0.1685818	0.312611	79.30	62.72	753.50	832.00
ORF-T	YML102W	-0.339112	0.2010383	0.3520788	70.17	55.52	652.50	708.00

TABLE S1: Differential expression data for RRP6 RNA-Seq dataset Page 100

Class	Transcript name	RRP6 KO_vs_WT log2_fold_change	RRP6 KO_vs_WT p-value	RRP6 KO_vs_WT FDR	Ave Norm Reads in WT	Ave Norm Reads in RRP6	Average RAW read counts in WT	Average RAW read counts in RRP6
ORF-T	YKR049C	-0.338942	0.2857003	0.4488999	13.64	10.76	119.50	138.75
ORF-T	YGR079W	-0.33894	0.3440414	0.5084523	25.37	20.06	245.50	274.75
ORF-T	YHR106W	-0.33888	0.1736415	0.3192344	37.92	29.98	346.50	389.00
ORF-T	YPR116W	-0.338858	0.3104181	0.4757228	6.04	4.76	57.25	65.00
ORF-T	YIL102C-A	-0.338843	0.3094704	0.4752659	2.80	2.20	25.25	28.75
ORF-T	YLR323C	-0.338777	0.312405	0.477534	4.41	3.47	41.00	47.25
ORF-T	YBR123C	-0.338751	0.2441636	0.4042006	32.04	25.30	295.75	342.75
ORF-T	YMR297W	-0.338656	0.3724028	0.5345636	210.31	166.29	1891.50	2155.25
AST	AS_YER090C-A	-0.338586	0.2853458	0.4488252	6.82	5.36	61.25	70.75
ORF-T	YBL010C	-0.338419	0.2626641	0.4253581	8.69	6.86	78.00	89.25
ORF-T	YPL077C	-0.338362	0.3087565	0.4745466	4.90	3.87	43.75	49.25
ORF-T	YLR410W	-0.338275	0.1720845	0.3170078	70.13	55.52	640.50	700.50
ORF-T	YPL239W	-0.337981	0.1924371	0.3420652	37.11	29.27	329.25	396.00
ORF-T	YLR065C	-0.337876	0.1726619	0.3178917	29.55	23.35	265.75	306.50
SUT	SUT072	-0.33785	0.4767685	0.6237998	1.71	1.35	14.75	16.75
ORF-T	YNL047C	-0.337717	0.242194	0.4018897	10.99	8.69	99.00	111.50
AST	AS_YPR014C	-0.337684	0.2968712	0.4615555	6.85	5.46	65.25	71.00
ORF-T	YDR255C	-0.337676	0.3553358	0.5195827	18.98	14.99	161.75	191.25
SUT	SUT621	-0.337552	0.4035067	0.5623997	2.35	1.85	21.50	24.50
ORF-T	YOR175C	-0.337297	0.1631429	0.3059575	180.32	142.75	1675.25	1860.25
ORF-T	YEL050C	-0.337239	0.1782843	0.3252204	62.98	49.91	585.25	632.25
ORF-T	YDR286C	-0.337225	0.3627827	0.5265257	2.51	1.95	22.50	26.25
ORF-T	YPR078C	-0.337144	0.5446833	0.677261	2.04	1.64	19.00	20.50
ORF-T	YIL079C	-0.337078	0.2874689	0.4507597	29.17	23.10	280.75	311.50
SRT	SRT528	-0.336809	0.3679846	0.5302597	5.77	4.61	57.00	61.25
AST	AS_YKL153W	-0.336756	0.2682061	0.4311381	1380.95	1093.42	12290.75	14357.50
ORF-T	YLL051C	-0.336364	0.186037	0.3348023	33.15	26.24	299.00	341.25
ORF-T	YOR131C	-0.336348	0.2014633	0.3526395	65.35	51.77	601.75	664.25
ORF-T	YHL009C	-0.336331	0.1875224	0.3365863	19.19	15.19	174.50	198.75

Class	Transcript name	RRP6 KO_vs_WT log2_fold _change	RRP6 KO_vs_WT p-value	RRP6 KO_vs_WT FDR	Ave Norm Reads in WT	Ave Norm Reads in RRP6	Average RAW read counts in WT	Average RAW read counts in RRP6
ORF-T	YML008C	-0.33612	0.1897308	0.3388463	340.43	269.73	3203.50	3470.50
ORF-T	YDR495C	-0.335995	0.2506267	0.4123025	18.61	14.71	167.50	193.50
ORF-T	YMR039C	-0.335985	0.2120781	0.3656969	22.42	17.73	204.25	234.50
ORF-T	YCR024C-B	-0.335892	0.2016929	0.3527964	73.54	58.15	636.50	791.50
ORF-T	YER034W	-0.335839	0.3411473	0.5057526	4.37	3.43	39.75	46.75
ORF-T	YKL085W	-0.335833	0.5079829	0.6494041	315.36	249.86	2711.00	3042.75
ORF-T	YOL019W	-0.335793	0.2089457	0.3614079	40.30	31.89	363.25	424.75
ORF-T	YMR022W	-0.335649	0.3040873	0.4698049	8.35	6.62	74.50	83.25
ORF-T	YAL034W-A	-0.335551	0.2981088	0.4628354	5.14	4.06	46.00	53.00
SUT	SUT805	-0.335182	0.4771543	0.6239139	0.96	0.77	9.50	10.50
ORF-T	YIR016W	-0.335041	0.3121785	0.4774774	62.28	49.40	557.50	614.50
SUT	SUT344	-0.335012	0.2657381	0.4286338	6.77	5.32	60.25	71.00
ORF-T	YMR302C	-0.334872	0.1988657	0.3497022	102.16	81.03	944.50	1041.00
ORF-T	YNL094W	-0.334851	0.1759684	0.3222786	43.75	34.64	396.50	464.25
ORF-T	YML067C	-0.33482	0.1499638	0.2879898	83.59	66.30	758.75	846.75
ORF-T	YIL002C	-0.334631	0.2217266	0.3779396	41.83	33.14	386.50	448.75
ORF-T	YLR128W	-0.334598	0.257694	0.4193297	9.26	7.31	84.00	98.00
SUT	SUT310	-0.334503	0.52273	0.6610147	0.85	0.70	8.75	9.50
ORF-T	YLL041C	-0.334501	0.3635481	0.5272572	54.30	43.03	455.00	539.75
AST	AS_YPL205C	-0.334387	0.307207	0.473023	6.23	4.96	56.25	62.75
ORF-T	YDR393W	-0.333751	0.1719283	0.3168564	38.28	30.39	348.50	390.00
ORF-T	YJL072C	-0.333613	0.160701	0.302829	35.53	28.19	327.50	373.25
ORF-T	YDR245W	-0.333542	0.185351	0.3338238	187.68	148.97	1738.25	1929.00
NUT	NUT1162	-0.333453	0.349531	0.5140252	34154.27	27106.00	296724.50	345495.50
ORF-T	YKL003C	-0.333343	0.46538	0.6148968	2.20	1.71	17.75	21.25
ORF-T	YKL092C	-0.333191	0.1458252	0.2828545	61.94	49.18	566.25	636.50
ORF-T	YKL195W	-0.332832	0.2499467	0.411437	78.47	62.30	702.75	792.50
ORF-T	YDR326C	-0.332747	0.1990018	0.3497881	111.47	88.49	994.50	1144.50
ORF-T	YCR059C	-0.332734	0.2388967	0.3983572	54.20	43.10	504.00	537.75

Class	Transcript name	RRP6 KO_vs_WT log2_fold _change	RRP6 KO_vs_WT p-value	RRP6 KO_vs_WT FDR	Ave Norm Reads in WT	Ave Norm Reads in RRP6	Average RAW read counts in WT	Average RAW read counts in RRP6
ORF-T	YBL068W-A	-0.332706	0.303115	0.4690932	7.09	5.66	67.50	74.75
ORF-T	YHR103W	-0.332647	0.1684288	0.3124997	187.25	148.72	1749.25	1948.00
AST	AS_YAL016C-A	-0.332609	0.2181477	0.3737345	49.14	39.02	448.00	505.75
ORF-T	YNL010W	-0.332273	0.1904577	0.3396843	158.56	125.95	1417.75	1598.75
SUT	SUT681	-0.332026	0.2060106	0.3580508	30.40	24.16	286.25	320.25
AST	AS_YJL195C	-0.331881	0.3587595	0.522558	3.58	2.83	33.25	38.25
ORF-T	YDL160C-A	-0.331839	0.2896321	0.453517	8.90	7.05	79.50	92.50
ORF-T	YLR049C	-0.33179	0.2130472	0.3666783	39.80	31.62	363.25	413.50
ORF-T	YKL179C	-0.331701	0.1754421	0.3216066	59.54	47.33	553.50	616.00
ORF-T	YPL123C	-0.331696	0.4717837	0.6201078	19.41	15.41	166.00	194.75
ORF-T	YBR037C	-0.331323	0.2609791	0.4233092	34.65	27.52	315.50	359.75
ORF-T	YHR133C	-0.331236	0.1808743	0.3286965	200.14	159.10	1871.00	2079.00
ORF-T	YDR200C	-0.331163	0.3202829	0.4849448	8.91	7.06	79.50	92.75
ORF-T	YJR023C	-0.331153	0.4495838	0.6010345	5.39	4.27	45.75	54.00
AST	AS_YDR442W	-0.331097	0.3102277	0.4757228	6.19	4.94	56.00	62.25
AST	AS_YDL152W	-0.330855	0.2521644	0.4131989	11.11	8.87	105.50	115.75
ORF-T	YPR120C	-0.330751	0.30689	0.4727588	22.21	17.71	207.50	220.50
ORF-T	YBL098W	-0.330677	0.2997006	0.4645221	129.92	103.32	1229.50	1386.25
AST	AS_YOR049C	-0.330596	0.2516364	0.4126018	15.10	11.97	134.75	157.25
AST	AS_YLR169W	-0.330437	0.2520724	0.4131662	11.72	9.32	106.00	120.25
ORF-T	YBR082C	-0.330375	0.2076481	0.3599662	55.47	44.08	484.25	560.75
ORF-T	YBR177C	-0.330369	0.3573194	0.5212976	22.57	17.91	202.50	239.25
ORF-T	YOL090W	-0.329976	0.219388	0.3753492	147.45	117.39	1387.75	1461.50
ORF-T	YNL169C	-0.329855	0.1490146	0.2869051	51.14	40.72	483.00	538.50
ORF-T	YCR045W-A	-0.329737	0.3720896	0.5343551	4.81	3.80	42.50	49.75
ORF-T	YGR132C	-0.329539	0.2380507	0.397566	136.28	108.46	1228.50	1365.50
ORF-T	YGR065C	-0.329276	0.1824539	0.3306731	50.55	40.23	462.50	527.25
ORF-T	YAL026C-A	-0.329169	0.2081783	0.3606373	14.15	11.22	128.25	150.00
AST	AS_YPR038W	-0.329135	0.2279675	0.3849869	12.73	10.17	118.50	130.25

TABLE S1: Differential expression data for RRP6 RNA-Seq dataset Page 103

Class	Transcript name	RRP6 KO_vs_WT log2_fold _change	RRP6 KO_vs_WT p-value	RRP6 KO_vs_WT FDR	Ave Norm Reads in WT	Ave Norm Reads in RRP6	Average RAW read counts in WT	Average RAW read counts in RRP6
ORF-T	YPL206C	-0.328911	0.1959218	0.3458513	35.22	28.00	312.50	365.25
ORF-T	YEL055C	-0.328899	0.2222235	0.3784667	160.01	127.38	1512.75	1728.25
ORF-T	YJL001W	-0.328541	0.2355485	0.3943668	105.75	84.19	946.75	1084.50
ORF-T	YKL081W	-0.328443	0.3254916	0.4901086	1454.26	1158.16	12706.50	14509.50
ORF-T	YIL127C	-0.328279	0.263871	0.4268327	26.00	20.72	243.75	272.75
AST	AS_YIL142C-A	-0.328216	0.1943844	0.3443436	104.22	83.06	958.75	1043.50
ORF-T	YOL023W	-0.328192	0.2028329	0.3542941	15.10	12.01	136.50	157.00
ORF-T	YKL035W	-0.328142	0.3073811	0.4731029	428.77	341.53	3763.00	4312.75
ORF-T	YGL202W	-0.328109	0.2064807	0.3586462	244.06	194.42	2300.00	2607.00
ORF-T	YMR290C	-0.328044	0.1748619	0.320718	113.34	90.31	1062.75	1189.00
ORF-T	YDR139C	-0.328033	0.215123	0.36962	28.54	22.74	255.75	289.75
ORF-T	YOR125C	-0.327859	0.2702774	0.4333228	31.63	25.22	288.50	318.25
ORF-T	YDL044C	-0.32777	0.2710418	0.4341145	8.19	6.53	74.00	83.00
ORF-T	YGR230W	-0.327745	0.3841086	0.5453888	6.44	5.06	55.75	69.25
ORF-T	YLR026C	-0.327741	0.2567274	0.4181881	17.99	14.29	164.25	195.00
ORF-T	YBR119W	-0.32773	0.2907684	0.454802	8.29	6.55	73.00	88.00
AST	AS_YLR279W	-0.327647	0.3313878	0.4956563	4.76	3.81	43.00	47.75
ORF-T	YDR043C	-0.32757	0.3989904	0.5595077	8.44	6.75	76.50	85.25
ORF-T	YBR280C	-0.327396	0.3206411	0.4852687	29.27	23.34	263.50	294.00
ORF-T	YGL244W	-0.327183	0.2223202	0.3785037	44.11	35.14	408.25	472.75
ORF-T	YLR103C	-0.326895	0.3212886	0.485725	23.62	18.90	220.75	231.50
ORF-T	YNL257C	-0.326796	0.2523237	0.4133905	51.03	40.67	468.25	536.75
ORF-T	YNR009W	-0.326792	0.39074	0.5517746	4.26	3.38	38.00	44.00
AST	AS_YOR325W	-0.326606	0.3079733	0.473772	15.87	12.69	152.50	168.25
ORF-T	YML071C	-0.326504	0.2078635	0.3602536	40.99	32.70	387.75	435.00
ORF-T	YJR083C	-0.326481	0.4341009	0.5886075	2.23	1.76	20.50	23.75
ORF-T	YLR001C	-0.326411	0.2445502	0.4046012	23.37	18.61	203.75	239.25
ORF-T	YDR090C	-0.326341	0.2049414	0.3568075	24.87	19.85	228.25	257.25
ORF-T	YLR170C	-0.326294	0.2248157	0.3814	20.35	16.23	184.50	209.25

Class	Transcript name	RRP6 KO_vs_WT log2_fold _change	RRP6 KO_vs_WT p-value	RRP6 KO_vs_WT FDR	Ave Norm Reads in WT	Ave Norm Reads in RRP6	Average RAW read counts in WT	Average RAW read counts in RRP6
AST	AS_YIL115W-A	-0.325979	0.2760844	0.4397466	10.07	8.03	92.50	105.25
ORF-T	YBL082C	-0.325892	0.2003467	0.3513045	42.98	34.26	394.25	456.75
ORF-T	YLR139C	-0.325886	0.4960373	0.6390751	1.49	1.16	12.75	15.00
ORF-T	YLR195C	-0.325771	0.1494987	0.2874252	87.48	69.82	805.25	902.50
ORF-T	YGR112W	-0.325761	0.3662233	0.5288028	8.29	6.61	76.00	86.75
ORF-T	YLR363W-A	-0.325342	0.4090082	0.5669332	4.22	3.37	39.25	45.00
ORF-T	YNL271C	-0.325332	0.2050564	0.3569461	110.97	88.54	997.25	1161.75
ORF-T	YDL115C	-0.325034	0.2554563	0.4171695	16.32	12.99	143.50	169.25
sn/snoRNA	SNR190	-0.324843	0.3617471	0.525784	32624.51	26046.91	283929.75	332118.25
CUT	CUT727	-0.324794	0.2802458	0.4439903	5.53	4.43	51.75	58.75
ORF-T	YPL209C	-0.324669	0.2849174	0.4485025	6.87	5.51	64.00	72.25
ORF-T	YDR463W	-0.324503	0.1829712	0.3311939	33.66	26.81	303.25	362.75
ORF-T	YBL050W	-0.324327	0.1940032	0.3437707	79.75	63.69	719.50	818.75
ORF-T	YKR074W	-0.324309	0.2075695	0.3599124	46.04	36.83	423.50	460.25
ORF-T	YBR049C	-0.324266	0.2567851	0.4181881	49.29	39.32	456.00	535.25
ORF-T	YKR078W	-0.324148	0.2318827	0.3897175	19.41	15.47	173.75	203.75
ORF-T	YDL053C	-0.324084	0.1633224	0.3061653	63.94	51.09	600.75	674.00
ORF-T	YER180C-A	-0.323841	0.3920823	0.5531279	3.12	2.47	28.25	33.25
ORF-T	YDR409W	-0.323722	0.2443686	0.4043686	12.43	9.95	112.50	126.25
ORF-T	YOR019W	-0.323643	0.4463329	0.5985134	9.44	7.55	82.50	92.50
ORF-T	YKL024C	-0.323629	0.28565	0.4488999	31.18	24.92	276.75	314.00
ORF-T	YGR106C	-0.323371	0.2336251	0.3920578	183.95	147.00	1667.25	1911.75
ORF-T	YHR164C	-0.323146	0.2025731	0.3539064	36.38	29.08	333.75	379.75
ORF-T	YDR461W	-0.323126	0.5486152	0.6803044	23.54	18.81	193.50	222.00
AST	AS_YGL042C	-0.323021	0.2390634	0.3985312	15.92	12.71	146.25	167.75
ORF-T	YGR268C	-0.322931	0.2222637	0.3784713	14.32	11.44	129.50	148.50
AST	AS_YHL006W-A	-0.322767	0.3619368	0.5259057	2.60	2.08	23.25	26.50
ORF-T	YGR161C	-0.322755	0.3975831	0.5583105	10.28	8.18	93.50	114.50
ORF-T	YOR105W	-0.322628	0.2842581	0.4479542	11.26	8.97	102.00	122.00

Class	Transcript name	RRP6 KO_vs_WT log2_fold _change	RRP6 KO_vs_WT p-value	RRP6 KO_vs_WT FDR	Ave Norm Reads in WT	Ave Norm Reads in RRP6	Average RAW read counts in WT	Average RAW read counts in RRP6
SUT	SUT592	-0.322379	0.3980052	0.5587476	2.41	1.90	21.75	25.50
ORF-T	YKL210W	-0.322193	0.3112228	0.4765938	441.57	353.18	3992.25	4559.25
ORF-T	YNL136W	-0.3218	0.32267	0.4868709	12.05	9.64	113.25	128.25
ORF-T	YOL077W-A	-0.321509	0.4297903	0.584334	8.63	6.86	72.25	89.00
ORF-T	YGL129C	-0.321311	0.2346326	0.3934212	33.95	27.18	309.50	348.25
ORF-T	YNL149C	-0.321261	0.1905656	0.3398167	127.49	102.03	1174.75	1336.75
ORF-T	YDR461C-A	-0.321235	0.2459255	0.4064764	22.72	18.13	198.75	236.00
ORF-T	YIR002C	-0.321116	0.1767207	0.3231626	50.56	40.48	471.00	535.25
ORF-T	YJL055W	-0.320982	0.1834098	0.3316566	49.00	39.23	452.25	515.75
ORF-T	YMR139W	-0.320968	0.3174345	0.4828069	24.16	19.30	213.50	253.75
ORF-T	YOR309C	-0.320773	0.2286005	0.3856797	31.62	25.30	290.25	337.25
ORF-T	YBR161W	-0.320636	0.2675198	0.4305249	16.32	13.04	148.00	172.00
ORF-T	YOR364W	-0.320549	0.5411077	0.6744312	1.13	0.87	9.25	11.50
ORF-T	YKL112W	-0.320363	0.1847551	0.3329885	56.06	44.86	513.75	600.00
ORF-T	YNL099C	-0.32036	0.2484636	0.4093966	13.44	10.75	119.75	137.75
ORF-T	YFL059W	-0.320347	0.404928	0.5636016	2.82	2.27	25.25	28.00
ORF-T	YAL008W	-0.320262	0.2694963	0.4326012	10.92	8.74	99.50	113.00
AST	AS_YAL042C-A	-0.319961	0.1865907	0.3354797	77.16	61.90	740.50	798.00
ORF-T	YIL031W	-0.319961	0.2034194	0.3548929	25.05	20.03	223.75	260.00
ORF-T	YMR270C	-0.319957	0.1920412	0.3416016	27.81	22.26	257.75	299.25
ORF-T	YGR258C	-0.319929	0.253615	0.4150364	36.63	29.35	334.75	380.75
ORF-T	YNL267W	-0.319898	0.2247455	0.3814	55.45	44.38	505.00	589.75
ORF-T	YDR194C	-0.319884	0.2259524	0.3826081	165.38	132.50	1529.25	1715.00
ORF-T	YPL004C	-0.319773	0.2812723	0.4450573	175.02	140.18	1517.50	1805.75
AST	AS_YMR006C	-0.319655	0.473082	0.6208801	3.92	3.15	38.25	43.50
ORF-T	YDL133C-A	-0.319501	0.2893062	0.4532179	3232.25	2590.14	28471.00	33359.75
ORF-T	YIL115C	-0.319434	0.2667882	0.4296854	93.00	74.50	851.00	998.50
ORF-T	YMR028W	-0.319306	0.318422	0.4835066	11.13	8.88	101.00	119.50
ORF-T	YGR049W	-0.319245	0.4373435	0.5907799	5.20	4.12	44.25	54.50

Class	Transcript name	RRP6 KO_vs_WT log2_fold _change	RRP6 KO_vs_WT p-value	RRP6 KO_vs_WT FDR	Ave Norm Reads in WT	Ave Norm Reads in RRP6	Average RAW read counts in WT	Average RAW read counts in RRP6
ORF-T	YIL003W	-0.319212	0.2267533	0.3835257	20.18	16.19	188.50	213.50
ORF-T	YBL059W	-0.319198	0.2764943	0.4401212	18.78	15.04	167.25	192.75
ORF-T	YGL116W	-0.319169	0.4371606	0.5907702	47.48	38.04	453.50	532.00
ORF-T	YIL153W	-0.319016	0.285009	0.4485766	19.57	15.67	168.75	196.50
ORF-T	YKL142W	-0.318769	0.317066	0.4824648	72.77	58.33	620.25	720.75
ORF-T	YDL143W	-0.318743	0.1930548	0.3425593	271.88	217.99	2503.75	2842.00
sn/snoRNA	SNR49	-0.318736	0.3138812	0.4792094	220.35	176.67	1913.25	2203.50
AST	AS_YLR171W	-0.318713	0.2547666	0.4164478	9.92	7.97	91.00	102.50
ORF-T	YLL015W	-0.318552	0.2131021	0.3667102	62.17	49.84	554.25	638.25
ORF-T	YDR277C	-0.318424	0.3963282	0.5570793	34.67	27.75	300.25	366.50
CUT	CUT677	-0.318372	0.3716175	0.5341207	31219.75	25037.42	271764.50	319367.00
ORF-T	YCL017C	-0.318357	0.1766648	0.3231626	67.32	53.97	618.75	713.00
ORF-T	YLR102C	-0.318235	0.2873667	0.4506697	11.62	9.28	103.25	122.75
ORF-T	YBL063W	-0.318061	0.2105737	0.363538	30.04	24.08	269.25	311.75
ORF-T	YGR255C	-0.317995	0.3283926	0.4927843	53.71	43.06	487.25	564.00
ORF-T	YDL089W	-0.317694	0.4016876	0.5608572	21.80	17.48	201.00	230.50
ORF-T	YLR073C	-0.317288	0.3578308	0.521519	12.71	10.17	116.00	138.75
ORF-T	YBR084C-A	-0.317091	0.2983566	0.4630795	311.02	249.63	2720.00	3165.00
ORF-T	YMR024W	-0.316734	0.2333802	0.3917772	35.94	28.86	338.25	384.25
ORF-T	YKL213C	-0.316588	0.2926968	0.4571092	115.35	92.62	1056.50	1207.75
ORF-T	YGL026C	-0.316313	0.2857175	0.4488999	376.78	302.60	3528.75	4022.75
ORF-T	YLR345W	-0.316309	0.4779813	0.6242376	26.44	21.22	221.00	263.00
ORF-T	YBR080C	-0.31582	0.2024802	0.3538666	61.25	49.14	553.25	663.75
ORF-T	YJR004C	-0.315663	0.3484418	0.5131718	4.80	3.84	42.25	48.75
ORF-T	YBL080C	-0.31553	0.2382979	0.3977962	22.86	18.39	209.50	235.00
ORF-T	YDR196C	-0.315432	0.3282163	0.4927843	31.22	25.08	286.00	325.50
ORF-T	YGL173C	-0.315346	0.307182	0.473023	262.34	210.80	2333.00	2745.00
ORF-T	YOR151C	-0.315285	0.2351624	0.3939166	446.79	359.13	4177.25	4618.75
ORF-T	YGL084C	-0.314974	0.3219892	0.4862871	51.82	41.63	465.25	545.50

Class	Transcript name	RRP6 KO_vs_WT log2_fold _change	RRP6 KO_vs_WT p-value	RRP6 KO_vs_WT FDR	Ave Norm Reads in WT	Ave Norm Reads in RRP6	Average RAW read counts in WT	Average RAW read counts in RRP6
ORF-T	YMR217W	-0.314972	0.2615232	0.423919	758.02	609.40	7161.25	7854.75
ORF-T	YPR060C	-0.314907	0.2378968	0.3975064	65.60	52.76	595.00	672.75
ORF-T	YCR026C	-0.314644	0.2259481	0.3826081	147.71	118.81	1356.75	1505.25
ORF-T	YMR001C-A	-0.314607	0.3607626	0.5247267	8.53	6.84	79.00	91.75
ORF-T	YNR041C	-0.314588	0.1753272	0.3214545	82.52	66.40	775.75	863.00
ORF-T	YHR171W	-0.314535	0.4052814	0.5639379	12.28	9.85	105.25	124.75
ORF-T	YNR060W	-0.314498	0.5367054	0.6716583	6.13	4.94	56.50	65.00
ORF-T	YJR036C	-0.314402	0.4785823	0.6246472	10.61	8.53	92.75	106.00
AST	AS_YDL118W	-0.314253	0.3532949	0.5178236	7.97	6.42	75.50	84.75
ORF-T	YGR220C	-0.314185	0.2658142	0.4286338	23.32	18.73	203.50	238.75
ORF-T	YHR072W-A	-0.314089	0.2990401	0.4637113	240.22	193.26	2196.50	2423.50
ORF-T	YGL231C	-0.313992	0.2553262	0.41713	13.35	10.69	120.00	142.50
ORF-T	YIL087C	-0.313825	0.5107401	0.65136	1.91	1.49	16.25	20.00
ORF-T	YER128W	-0.313493	0.5296695	0.6667316	1.64	1.29	13.75	17.00
ORF-T	YIL022W	-0.313464	0.2794153	0.4432344	51.17	41.16	481.25	562.00
ORF-T	YKR014C	-0.313454	0.3205445	0.4852535	15.23	12.23	133.00	155.75
ORF-T	YPL104W	-0.313244	0.266566	0.4294012	36.38	29.26	333.75	386.75
ORF-T	YOR168W	-0.313159	0.229562	0.3871723	582.75	469.11	5508.00	5986.50
ORF-T	YLR176C	-0.313148	0.4205604	0.5761351	4.21	3.37	35.75	41.75
SUT	SUT316	-0.31302	0.4778308	0.6242376	1.98	1.58	18.25	21.25
SRT	SRT225	-0.31302	0.4778073	0.6242376	1.98	1.58	18.25	21.25
ORF-T	YAL003W	-0.312933	0.2434933	0.403382	704.44	567.05	6235.00	7322.00
ORF-T	YHR048W	-0.312915	0.2832824	0.4470454	27.97	22.56	263.25	291.25
ORF-T	YBR034C	-0.312082	0.1845106	0.3327261	44.55	35.86	405.50	471.50
ORF-T	YLR091W	-0.311831	0.295614	0.4602895	9.79	7.86	90.00	105.75
ORF-T	YER065C	-0.311627	0.3423107	0.5070604	44.13	35.55	400.75	464.75
ORF-T	YLR093C	-0.311557	0.2128811	0.3664549	82.90	66.84	751.75	842.50
ORF-T	YLR017W	-0.31149	0.2094784	0.362143	189.43	152.69	1749.50	1947.75
AST	AS_YPL245W	-0.311372	0.436282	0.5900127	3.32	2.68	30.25	34.75

Class	Transcript name	RRP6 KO_vs_WT log2_fold _change	RRP6 KO_vs_WT p-value	RRP6 KO_vs_WT FDR	Ave Norm Reads in WT	Ave Norm Reads in RRP6	Average RAW read counts in WT	Average RAW read counts in RRP6
ORF-T	YFR034C	-0.311336	0.1984694	0.3491831	35.04	28.22	317.50	368.25
ORF-T	YLL053C	-0.311209	0.4304767	0.5849517	4.51	3.69	45.25	48.25
ORF-T	YOL069W	-0.311188	0.3494308	0.5140252	21.62	17.39	199.50	238.50
ORF-T	YBR257W	-0.3111	0.2804701	0.4441746	11.39	9.15	104.25	122.75
ORF-T	YOL033W	-0.31081	0.2350073	0.3937875	34.34	27.73	317.50	350.25
ORF-T	YDL149W	-0.310715	0.3188977	0.4839375	18.38	14.80	166.50	193.50
ORF-T	YKR005C	-0.310674	0.4281464	0.5829213	5.60	4.54	54.50	60.00
ORF-T	YNL064C	-0.310658	0.2319569	0.3897772	318.26	256.61	2895.00	3292.75
ORF-T	YML092C	-0.310644	0.226937	0.3835829	75.24	60.60	655.50	789.50
ORF-T	YIL093C	-0.310409	0.2696542	0.4327168	34.73	28.03	319.00	354.75
AST	AS_YPR076W	-0.310341	0.2008118	0.3518656	33.57	27.11	305.25	343.25
AST	AS_YOR345C	-0.310136	0.2658771	0.4286338	15.30	12.35	147.25	168.50
ORF-T	YLR353W	-0.310025	0.2956038	0.4602895	18.69	15.02	171.50	208.00
SUT	SUT673	-0.309975	0.309356	0.4752507	48.38	39.05	428.50	477.50
ORF-T	YBL005W	-0.309746	0.2281776	0.3851595	18.32	14.74	164.00	194.50
ORF-T	YFR042W	-0.30965	0.2691047	0.432179	30.34	24.47	272.25	315.75
ORF-T	YDR386W	-0.309611	0.2613806	0.4237948	11.98	9.67	109.00	125.75
ORF-T	YER163C	-0.309433	0.2521035	0.4131662	37.03	29.90	341.75	385.25
ORF-T	YKL191W	-0.309351	0.220018	0.3759494	166.68	134.56	1582.25	1754.50
ORF-T	YDL035C	-0.309218	0.1907637	0.3400497	111.68	90.14	1019.50	1169.75
ORF-T	YDR483W	-0.309137	0.2196125	0.375606	90.04	72.64	791.50	938.50
ORF-T	YFR052W	-0.309136	0.2961411	0.4607756	38.02	30.65	336.00	399.25
ORF-T	YBR179C	-0.308994	0.236454	0.3956201	33.80	27.25	307.75	361.75
ORF-T	YER179W	-0.308733	0.4882591	0.6331748	1.94	1.56	17.25	20.00
ORF-T	YIL147C	-0.308648	0.2051704	0.3570213	106.56	86.05	1005.50	1146.25
ORF-T	YLR135W	-0.308516	0.2414425	0.4011048	15.68	12.70	149.00	166.75
ORF-T	YLR095C	-0.308233	0.222444	0.3785865	58.52	47.21	538.25	640.25
ORF-T	YKL137W	-0.308163	0.2745591	0.4378704	29.48	23.83	261.50	294.50
AST	AS_YNL226W	-0.307886	0.3737789	0.5357764	4.04	3.28	38.25	43.75

Class	Transcript name	RRP6 KO_vs_WT log2_fold_change	RRP6 KO_vs_WT p-value	RRP6 KO_vs_WT FDR	Ave Norm Reads in WT	Ave Norm Reads in RRP6	Average RAW read counts in WT	Average RAW read counts in RRP6
ORF-T	YGL092W	-0.307838	0.2653134	0.4283414	183.82	148.48	1713.00	2001.00
ORF-T	YPL053C	-0.307606	0.2199115	0.3758623	204.27	165.09	1886.00	2102.00
ORF-T	YJL164C	-0.307572	0.494914	0.6380354	50.33	40.65	427.25	501.75
SUT	SUT200	-0.307545	0.4991883	0.6417108	1.49	1.19	13.25	15.50
ORF-T	YDL184C	-0.307528	0.3224259	0.4867975	4734.67	3825.72	42369.75	49028.00
ORF-T	YKR020W	-0.307495	0.31387	0.4792094	11.18	9.02	100.00	116.75
ORF-T	YBR246W	-0.307443	0.2511416	0.4123025	38.29	30.87	348.50	425.50
ORF-T	YMR111C	-0.306924	0.2394473	0.3989733	12.59	10.17	114.00	131.50
ORF-T	YER190W	-0.306792	0.5379414	0.6724434	3.83	3.13	36.00	38.00
ORF-T	YPR175W	-0.306706	0.2718702	0.4351172	52.25	42.30	493.00	539.50
ORF-T	YOR061W	-0.306417	0.1880085	0.3370449	84.47	68.35	789.25	889.75
ORF-T	YBR004C	-0.306304	0.2743047	0.437603	15.92	12.87	146.25	169.00
ORF-T	YKR029C	-0.30594	0.2561923	0.417964	20.48	16.54	185.25	218.75
ORF-T	YKL088W	-0.30563	0.1965671	0.3466791	50.80	41.11	464.50	533.75
ORF-T	YLR240W	-0.305494	0.3699089	0.5324998	33.87	27.37	306.25	368.75
AST	AS_YGR064W	-0.305486	0.3137368	0.479134	10.33	8.34	93.25	108.75
ORF-T	YML014W	-0.305467	0.2663265	0.429221	36.61	29.68	352.75	387.50
ORF-T	YFL021W	-0.305264	0.5109527	0.6515487	1.83	1.46	15.25	18.25
ORF-T	YJL071W	-0.305232	0.2976765	0.4623799	22.24	18.02	212.50	239.50
ORF-T	YFR025C	-0.304973	0.2415211	0.4011693	33.07	26.71	298.75	359.50
ORF-T	YKL196C	-0.304747	0.2080597	0.3604938	130.60	105.77	1198.00	1347.00
ORF-T	YOL159C	-0.304734	0.2619676	0.4243664	13.92	11.32	130.00	145.75
ORF-T	YMR054W	-0.304562	0.2243893	0.3810613	122.08	98.87	1111.25	1268.50
ORF-T	YHR151C	-0.3043	0.2187845	0.3746345	29.85	24.13	268.75	319.75
ORF-T	YIL120W	-0.304282	0.2202695	0.3762829	28.38	22.98	255.50	297.75
ORF-T	YFR050C	-0.304281	0.2437256	0.4036343	62.64	50.69	550.75	655.25
ORF-T	YKL214C	-0.304271	0.2519757	0.4130911	20.47	16.53	186.25	224.00
ORF-T	YOR259C	-0.304004	0.2773825	0.4413261	198.09	160.47	1806.25	2046.25
ORF-T	YDR186C	-0.30393	0.1980191	0.3486685	42.04	34.00	371.25	445.00

TABLE S1: Differential expression data for RRP6 RNA-Seq dataset Page 110

Class	Transcript name	RRP6 KO_vs_WT log2_fold _change	RRP6 KO_vs_WT p-value	RRP6 KO_vs_WT FDR	Ave Norm Reads in WT	Ave Norm Reads in RRP6	Average RAW read counts in WT	Average RAW read counts in RRP6
NUT	NUT1212	-0.303875	0.3492794	0.5138801	24.66	19.96	222.25	259.50
ORF-T	YHL003C	-0.303753	0.2505784	0.4123025	165.41	134.03	1508.50	1697.25
ORF-T	YKL049C	-0.303711	0.2470924	0.4077193	14.43	11.71	132.50	150.75
ORF-T	YGL050W	-0.303558	0.2404382	0.4000955	18.28	14.82	171.00	196.50
SUT	SUT439	-0.303557	0.4446256	0.5967795	2.84	2.26	24.75	30.50
ORF-T	YOR136W	-0.303333	0.3911743	0.5520695	35.47	28.70	301.00	365.75
ORF-T	YNL230C	-0.303191	0.3331397	0.4973177	8.89	7.20	81.00	93.50
AST	AS_YMR135W-A	-0.303067	0.3295431	0.4940697	8.65	7.03	78.75	89.25
ORF-T	YDR302W	-0.303024	0.2308813	0.3888716	48.16	39.05	448.50	508.25
ORF-T	YNL255C	-0.302988	0.216411	0.3712638	247.78	200.82	2230.50	2617.00
ORF-T	YGL097W	-0.302976	0.2046069	0.3562945	94.54	76.67	887.25	1000.00
ORF-T	YLL052C	-0.302922	0.4420821	0.5948701	3.53	2.87	33.25	37.75
SUT	SUT101	-0.302857	0.5183078	0.6574125	1.37	1.08	11.25	13.75
NUT	NUT1354	-0.302655	0.2593725	0.4215169	19.47	15.75	176.00	209.75
ORF-T	YMR244C-A	-0.302516	0.2409977	0.4006299	17.49	14.21	161.50	184.25
ORF-T	YDL028C	-0.302472	0.2658482	0.4286338	18.66	15.08	168.00	203.25
ORF-T	YIL065C	-0.302387	0.3034361	0.4693248	38.47	31.24	348.00	381.75
ORF-T	YIL142W	-0.30234	0.239735	0.3990809	365.03	296.05	3361.25	3777.50
ORF-T	YDR017C	-0.302301	0.1933339	0.3428736	35.97	29.18	335.50	385.00
ORF-T	YML117W	-0.302267	0.2781863	0.4419774	185.73	150.63	1712.00	1960.25
AST	AS_YLR415C	-0.302051	0.5416847	0.6748639	0.86	0.70	8.00	9.25
ORF-T	YNL170W	-0.301867	0.4545231	0.6051506	2.44	1.98	23.00	26.25
ORF-T	YPR173C	-0.301739	0.187939	0.3370298	93.68	76.03	865.75	981.25
ORF-T	YBR138C	-0.301734	0.4715073	0.6199061	6.68	5.38	59.00	72.75
ORF-T	YCR016W	-0.301708	0.2318559	0.3897175	30.22	24.53	286.00	328.50
ORF-T	YDR334W	-0.301631	0.2690669	0.432179	235.70	191.26	2145.75	2426.25
ORF-T	YPL146C	-0.301478	0.2095938	0.3622804	50.67	41.16	471.75	531.25
ORF-T	YFR033C	-0.301318	0.3500554	0.5144219	57.63	46.74	505.00	592.25
ORF-T	YLL058W	-0.300968	0.3327971	0.4970269	35.04	28.43	320.25	376.25

Class	Transcript name	RRP6 KO_vs_WT log2_fold_change	RRP6 KO_vs_WT p-value	RRP6 KO_vs_WT FDR	Ave Norm Reads in WT	Ave Norm Reads in RRP6	Average RAW read counts in WT	Average RAW read counts in RRP6
ORF-T	YJL118W	-0.300929	0.4721266	0.6203033	3.31	2.64	28.25	35.50
ORF-T	YKL011C	-0.300494	0.3085149	0.4742476	10.83	8.75	97.00	117.00
AST	AS_YIL066W-A	-0.300436	0.2934561	0.4580451	10.59	8.60	97.00	112.00
NUT	NUT0279	-0.30042	0.3146979	0.4800928	43.79	35.50	375.50	463.25
SUT	SUT060	-0.300409	0.3111301	0.4765243	12.56	10.16	109.00	131.00
ORF-T	YLR211C	-0.300361	0.3944174	0.5554817	3.86	3.10	33.75	41.00
ORF-T	YHR007C	-0.30036	0.2753052	0.438852	283.80	230.41	2509.00	3030.25
ORF-T	YLR287C	-0.300334	0.221909	0.3781228	75.79	61.58	721.50	812.75
ORF-T	YNL208W	-0.299944	0.3644021	0.527928	173.79	141.25	1620.50	1696.75
ORF-T	YDL215C	-0.299898	0.3473617	0.5121878	345.77	280.87	3237.50	3772.00
ORF-T	YDR227W	-0.29981	0.2309166	0.3888716	38.08	30.89	341.50	406.75
ORF-T	YGR191W	-0.299776	0.2423309	0.4019847	79.01	64.20	705.00	809.00
ORF-T	YER035W	-0.299754	0.2812508	0.4450573	19.43	15.73	171.00	208.00
ORF-T	YGR243W	-0.299581	0.4353682	0.5892984	124.72	101.36	1173.25	1288.25
ORF-T	YMR250W	-0.299184	0.4697232	0.6182052	61.34	49.84	516.75	614.50
ORF-T	YGR017W	-0.299152	0.2916058	0.4556877	53.21	43.22	489.50	573.50
AST	AS_YGL214W	-0.299115	0.248045	0.4089073	19.89	16.18	187.25	215.00
ORF-T	YJL206C	-0.298987	0.2273687	0.38418	23.52	19.11	217.25	252.75
ORF-T	YEL071W	-0.298944	0.324689	0.4891922	252.87	205.56	2271.75	2597.25
ORF-T	YKR048C	-0.298745	0.2697684	0.4327795	267.52	217.52	2507.50	2825.00
ORF-T	YPR118W	-0.298703	0.2757151	0.439436	242.29	196.99	2249.50	2566.00
ORF-T	YMR281W	-0.298464	0.364124	0.527817	17.57	14.28	153.25	177.75
ORF-T	YBR056W	-0.29841	0.4157215	0.5723833	78.44	63.77	698.00	811.75
ORF-T	YDL185W	-0.29826	0.3211024	0.4856751	1450.06	1179.26	13608.00	15333.75
ORF-T	YBR025C	-0.298194	0.2909115	0.4548847	203.96	165.82	1802.00	2175.50
SUT	SUT265	-0.298113	0.3110929	0.4765243	15.36	12.51	136.00	156.50
ORF-T	YDR479C	-0.297885	0.2566384	0.4181881	37.47	30.52	341.50	385.00
ORF-T	YDR328C	-0.29781	0.2664364	0.4293297	247.78	201.61	2328.00	2590.25
ORF-T	YOR065W	-0.297664	0.5037314	0.6459344	10.78	8.75	89.25	107.00

Class	Transcript name	RRP6 KO_vs_WT log2_fold _change	RRP6 KO_vs_WT p-value	RRP6 KO_vs_WT FDR	Ave Norm Reads in WT	Ave Norm Reads in RRP6	Average RAW read counts in WT	Average RAW read counts in RRP6
ORF-T	YBL084C	-0.297616	0.3318477	0.4961234	12.60	10.17	106.50	133.75
ORF-T	YJR072C	-0.297303	0.2409566	0.4006276	263.10	214.15	2464.25	2751.50
ORF-T	YKL055C	-0.297283	0.3053885	0.4708053	16.02	13.06	151.25	171.25
ORF-T	YJR093C	-0.297146	0.4069529	0.5651531	4.41	3.57	39.75	46.75
ORF-T	YDR229W	-0.297062	0.3337787	0.4977557	27.63	22.49	250.00	291.00
ORF-T	YMR106C	-0.296993	0.3378935	0.5021983	11.52	9.35	105.75	124.75
ORF-T	YPL082C	-0.296905	0.2604223	0.4226634	185.89	151.33	1745.75	2015.00
ORF-T	YIR024C	-0.296857	0.4348701	0.5891083	4.08	3.28	34.75	42.75
ORF-T	YJL141C	-0.296804	0.3646362	0.5279324	44.86	36.51	385.25	452.50
sn/snoRNA	SNR54	-0.296668	0.33884	0.5034423	926.69	754.46	8242.25	9437.50
ORF-T	YJL014W	-0.29662	0.2284563	0.3855269	237.89	193.70	2175.50	2497.50
ORF-T	YDR494W	-0.296592	0.2598863	0.4220799	47.19	38.44	437.75	496.00
ORF-T	YNR054C	-0.296533	0.2808573	0.4446099	15.75	12.81	143.75	169.25
ORF-T	YML056C	-0.295967	0.3399691	0.5045333	927.03	755.10	8245.50	9445.50
ORF-T	YKL186C	-0.295946	0.2461245	0.406672	32.23	26.18	288.75	353.50
ORF-T	YDL010W	-0.295698	0.3197994	0.484674	9.81	7.99	86.50	101.25
ORF-T	YJL148W	-0.295463	0.2592674	0.421414	105.00	85.60	980.75	1104.25
ORF-T	YPL086C	-0.295439	0.2420047	0.4017078	82.29	67.10	777.75	874.75
ORF-T	YDR002W	-0.295366	0.2677703	0.430722	457.87	373.14	4162.25	4721.75
ORF-T	YDR050C	-0.295287	0.3177501	0.4829955	968.02	788.80	8531.50	10308.25
ORF-T	YBR042C	-0.295279	0.2369048	0.3961771	55.85	45.52	507.75	585.50
AST	AS_YFL010W-A	-0.295229	0.1946365	0.3445172	44.85	36.57	418.50	483.50
ORF-T	YNR001C	-0.295165	0.5312998	0.6679501	177.70	144.80	1497.50	1791.00
ORF-T	YGR199W	-0.29513	0.2054155	0.3572628	69.40	56.58	641.25	732.75
ORF-T	YKL008C	-0.295038	0.258979	0.421081	62.90	51.24	568.25	679.00
AST	AS_YDL151C	-0.294959	0.2937044	0.4582571	23.97	19.58	229.00	256.50
ORF-T	YNL181W	-0.294737	0.2247368	0.3814	44.55	36.32	404.50	469.00
ORF-T	YCL021W-A	-0.294699	0.3963748	0.5570793	10.52	8.60	98.75	112.00
ORF-T	YNL045W	-0.294642	0.4132004	0.5700523	44.49	36.25	399.50	477.25

Class	Transcript name	RRP6 KO_vs_WT log2_fold _change	RRP6 KO_vs_WT p-value	RRP6 KO_vs_WT FDR	Ave Norm Reads in WT	Ave Norm Reads in RRP6	Average RAW read counts in WT	Average RAW read counts in RRP6
ORF-T	YGR053C	-0.294569	0.4503759	0.601854	5.09	4.16	44.50	50.75
ORF-T	YHR030C	-0.294516	0.3046013	0.4701667	295.66	241.10	2866.25	3249.25
ORF-T	YDR512C	-0.294451	0.4456305	0.59781	4.80	3.88	41.75	50.00
ORF-T	YML124C	-0.294282	0.3186317	0.4837522	142.35	116.10	1343.00	1513.75
ORF-T	YDR166C	-0.29421	0.227999	0.3849869	48.48	39.47	433.00	522.75
ORF-T	YOR076C	-0.294145	0.2821541	0.445753	17.62	14.34	155.75	185.50
ORF-T	YPL266W	-0.294105	0.2739222	0.4372695	155.86	127.19	1472.50	1613.75
ORF-T	YLR212C	-0.294062	0.3264786	0.491031	18.74	15.24	171.25	208.25
ORF-T	YBL107C	-0.293779	0.3238939	0.4881402	9.58	7.81	86.00	100.75
SUT	SUT704	-0.29375	0.6209386	0.7380416	0.97	0.80	9.25	10.50
ORF-T	YBL052C	-0.293726	0.2532672	0.4146017	76.26	62.21	721.50	836.75
ORF-T	YKL146W	-0.293665	0.223263	0.3794681	98.25	80.13	874.75	1029.25
AST	AS_YGL041C	-0.293589	0.4257567	0.5808862	3.77	3.03	32.00	39.25
ORF-T	YGL068W	-0.293545	0.2854747	0.4488893	99.70	81.37	914.25	1027.00
ORF-T	YAL059W	-0.293492	0.2816865	0.4453633	83.90	68.50	791.00	888.50
ORF-T	YMR278W	-0.293368	0.2780236	0.4418582	34.34	27.98	302.75	363.25
ORF-T	YOR157C	-0.293244	0.2480012	0.408902	131.66	107.40	1199.00	1426.25
ORF-T	YKL107W	-0.293089	0.440958	0.5939914	1.99	1.65	19.00	21.25
ORF-T	YJL029C	-0.293051	0.2740953	0.4374373	31.62	25.77	279.50	335.00
ORF-T	YNL270C	-0.293017	0.4323169	0.5868197	6.48	5.31	63.00	71.50
ORF-T	YEL030W	-0.292767	0.2990732	0.4637113	13.57	11.08	127.50	147.50
ORF-T	YPL268W	-0.29276	0.1959733	0.3458513	48.57	39.67	450.25	518.50
ORF-T	YKL033W-A	-0.292645	0.2975691	0.4623383	139.21	113.66	1266.50	1454.00
ORF-T	YIR015W	-0.29253	0.3556433	0.5196793	8.02	6.52	74.25	89.00
ORF-T	YIL118W	-0.292515	0.2107016	0.3636343	86.86	70.99	819.50	910.50
AST	AS_YKR046C	-0.29249	0.23955	0.3990786	54.65	44.64	515.25	591.00
ORF-T	YGR237C	-0.292399	0.4161974	0.5728822	22.96	18.71	199.50	242.00
ORF-T	YNL037C	-0.292388	0.3781701	0.5397022	22.73	18.53	197.75	235.75
ORF-T	YPL038W	-0.292385	0.2873095	0.45065	10.22	8.36	94.50	109.25

Class	Transcript name	RRP6 KO_vs_WT log2_fold _change	RRP6 KO_vs_WT p-value	RRP6 KO_vs_WT FDR	Ave Norm Reads in WT	Ave Norm Reads in RRP6	Average RAW read counts in WT	Average RAW read counts in RRP6
SUT	SUT629	-0.292302	0.4087501	0.5667312	5.16	4.23	50.25	57.50
ORF-T	YOR264W	-0.29224	0.3851095	0.5461178	53.42	43.59	490.50	593.00
ORF-T	YHR027C	-0.292216	0.3491384	0.5138225	560.97	458.14	5160.50	5854.75
AST	AS_YHR028W-A	-0.29218	0.3352291	0.4994018	7.39	6.06	67.25	77.00
ORF-T	YEL067C	-0.292093	0.5655671	0.6946625	1.03	0.84	9.50	11.00
ORF-T	YMR159C	-0.291992	0.4029011	0.5618662	3.83	3.11	34.25	40.50
ORF-T	YNR031C	-0.291798	0.2658724	0.4286338	230.52	188.33	2124.50	2427.00
ORF-T	YBR058C-A	-0.291705	0.2388315	0.3983421	76.13	62.26	702.25	780.00
AST	AS_YNL285W	-0.291625	0.3737393	0.5357764	459.26	375.20	4004.25	4629.25
ORF-T	YIL009W	-0.291562	0.2739157	0.4372695	91.32	74.59	842.50	991.50
ORF-T	YDR315C	-0.291531	0.321328	0.485725	7.62	6.21	68.25	80.75
ORF-T	YGR103W	-0.291463	0.3577577	0.521519	570.41	466.14	5387.00	5822.25
ORF-T	YKR056W	-0.291418	0.3024216	0.4681639	30.67	25.00	280.50	343.75
ORF-T	YER116C	-0.291314	0.2463262	0.4069386	27.61	22.59	256.00	292.25
ORF-T	YOL013C	-0.291189	0.245577	0.4061	28.60	23.34	253.00	300.50
ORF-T	YLR074C	-0.290755	0.3052007	0.4706637	34.76	28.39	325.25	388.75
ORF-T	YMR311C	-0.290575	0.3629244	0.5265798	27.82	22.73	246.25	287.50
ORF-T	YEL058W	-0.29054	0.2553494	0.41713	224.31	183.41	2091.75	2404.50
ORF-T	YCR101C	-0.290513	0.4281731	0.5829213	4.71	3.80	41.25	51.25
NUT	NUT1436	-0.29049	0.3726279	0.5348106	30.57	24.98	279.75	329.00
ORF-T	YMR188C	-0.290109	0.2720455	0.435234	36.71	30.09	343.50	376.25
ORF-T	YLR071C	-0.290091	0.203803	0.3551934	55.81	45.61	501.25	600.25
ORF-T	YJL057C	-0.290073	0.3648881	0.5279855	43.61	35.68	389.00	443.25
CUT	CUT847	-0.290071	0.6236282	0.7400172	1.79	1.48	16.00	18.25
SUT	SUT1	-0.289977	0.4241164	0.5793339	11.44	9.36	98.25	114.50
ORF-T	YMR210W	-0.289963	0.4068014	0.5650394	32.20	26.34	289.25	333.50
ORF-T	YNL090W	-0.28994	0.2467542	0.4073121	64.88	53.10	619.00	705.00
ORF-T	YGL160W	-0.289936	0.2900105	0.4538277	20.63	16.82	182.75	221.75
ORF-T	YIR007W	-0.289785	0.4556796	0.6060503	7.55	6.15	65.50	78.75

Class	Transcript name	RRP6 KO_vs_WT log2_fold _change	RRP6 KO_vs_WT p-value	RRP6 KO_vs_WT FDR	Ave Norm Reads in WT	Ave Norm Reads in RRP6	Average RAW read counts in WT	Average RAW read counts in RRP6
ORF-T	YBR158W	-0.289751	0.3767567	0.5384266	175.05	143.16	1619.75	1952.25
ORF-T	YKL172W	-0.289559	0.2104754	0.3634927	66.18	54.18	619.25	708.50
ORF-T	YNL216W	-0.289505	0.2175799	0.3728884	49.42	40.43	464.00	544.50
ORF-T	YPR009W	-0.289411	0.3036328	0.4693262	12.09	9.96	116.50	128.50
ORF-T	YOL028C	-0.289298	0.3434987	0.5078946	10.14	8.30	97.00	113.25
ORF-T	YNL187W	-0.289274	0.2818419	0.4454604	12.03	9.82	107.50	128.00
ORF-T	YNL279W	-0.289139	0.4870247	0.6322293	8.91	7.26	79.50	98.50
ORF-T	YGL136C	-0.288752	0.3592203	0.5230115	6.88	5.58	60.75	74.75
ORF-T	YDR153C	-0.288677	0.260525	0.4227086	26.14	21.38	239.25	283.50
CUT	CUT588	-0.288627	0.4718571	0.6201234	1.47	1.21	13.75	16.00
ORF-T	YDR091C	-0.28849	0.2382282	0.3977962	53.67	43.94	478.00	564.00
ORF-T	YNL116W	-0.2879	0.3807241	0.5419792	10.10	8.21	90.50	113.00
ORF-T	YDR179C	-0.28789	0.3735064	0.5357034	5.85	4.83	55.00	61.75
ORF-T	YMR092C	-0.287415	0.3542938	0.5188351	279.83	229.31	2603.25	2944.50
ORF-T	YPL065W	-0.287123	0.4872267	0.6323286	2.84	2.30	24.75	30.00
ORF-T	YDR202C	-0.287089	0.3607182	0.5247267	12.04	9.79	105.50	133.25
ORF-T	YBR225W	-0.286807	0.2733268	0.4366602	15.86	13.00	142.75	165.50
ORF-T	YML022W	-0.286728	0.3309736	0.4951836	301.10	246.88	2765.25	3083.00
ORF-T	YDR028C	-0.286694	0.2826202	0.4464195	117.05	95.94	1049.25	1238.25
ORF-T	YML127W	-0.286655	0.2317183	0.3897009	134.61	110.37	1237.75	1425.75
ORF-T	YDR168W	-0.286509	0.2783083	0.442032	57.06	46.75	506.50	603.75
ORF-T	YPL180W	-0.286441	0.2761834	0.4398348	19.97	16.36	182.50	215.00
ORF-T	YPL225W	-0.285942	0.2455068	0.4060506	112.68	92.42	1018.50	1186.00
ORF-T	YAL020C	-0.285712	0.3223464	0.4867537	21.93	18.05	210.75	234.75
ORF-T	YHR176W	-0.285505	0.382316	0.5436089	10.05	8.28	93.25	104.25
ORF-T	YKL121W	-0.285438	0.276942	0.4407644	24.63	20.18	219.75	261.50
ORF-T	YHL008C	-0.285294	0.2958562	0.4604034	42.46	34.78	373.00	459.50
ORF-T	YGR233C	-0.285164	0.226913	0.3835829	66.47	54.56	609.25	710.50
SUT	SUT306	-0.284944	0.552963	0.6840136	0.92	0.77	8.50	9.75

Class	Transcript name	RRP6 KO_vs_WT log2_fold _change	RRP6 KO_vs_WT p-value	RRP6 KO_vs_WT FDR	Ave Norm Reads in WT	Ave Norm Reads in RRP6	Average RAW read counts in WT	Average RAW read counts in RRP6
ORF-T	YKR003W	-0.284928	0.3254067	0.4901086	55.38	45.48	514.25	583.00
ORF-T	YJR104C	-0.284612	0.460493	0.6101653	396.02	325.10	3461.50	4118.00
ORF-T	YNL122C	-0.284251	0.4156418	0.5723518	11.70	9.60	102.75	120.25
ORF-T	YLR260W	-0.283944	0.2365539	0.3957216	45.95	37.77	424.50	490.50
ORF-T	YHR079C	-0.283936	0.2166334	0.3715189	53.28	43.74	480.50	569.75
ORF-T	YPL118W	-0.283758	0.2899893	0.4538277	97.34	79.97	880.75	1012.00
ORF-T	YBR213W	-0.283633	0.4279825	0.5829213	2.87	2.33	25.00	30.25
ORF-T	YBR196C	-0.283556	0.319161	0.4842323	1394.34	1145.57	12738.75	14477.75
ORF-T	YPL071C	-0.28351	0.3588052	0.522558	6.66	5.48	62.50	73.25
ORF-T	YNL177C	-0.283508	0.2664909	0.4293488	48.55	39.87	439.75	519.00
ORF-T	YBR122C	-0.283466	0.2304699	0.3884437	51.18	42.09	476.50	542.00
AST	AS_YDL041W	-0.283313	0.4464227	0.5985542	2.42	1.96	22.00	26.50
ORF-T	YLR188W	-0.283261	0.2460129	0.4065543	106.05	87.20	1006.00	1132.50
ORF-T	YMR131C	-0.283236	0.2637798	0.4267848	249.58	205.14	2325.50	2608.00
AST	AS_YGL069C	-0.283218	0.3143874	0.4796918	76.85	63.18	706.75	793.25
ORF-T	YKL171W	-0.283121	0.2719168	0.4351172	31.95	26.25	288.25	339.00
ORF-T	YOR256C	-0.282981	0.2164702	0.3713021	87.61	72.02	810.25	942.00
AST	AS_YNL235C	-0.282874	0.3735686	0.5357034	5.37	4.43	48.50	55.75
ORF-T	YPR188C	-0.282707	0.3003128	0.4652564	16.92	13.88	155.50	187.25
ORF-T	YDR212W	-0.282633	0.2554282	0.4171695	481.82	396.13	4440.00	5084.25
ORF-T	YLR008C	-0.282236	0.2329429	0.3912386	38.57	31.69	352.00	417.75
ORF-T	YDR260C	-0.282182	0.2802998	0.4440062	18.20	15.02	170.50	191.75
ORF-T	YGR083C	-0.281978	0.2632062	0.4260991	32.45	26.69	301.50	352.50
ORF-T	YNL219C	-0.281945	0.2623978	0.4249951	126.81	104.34	1169.00	1332.25
SUT	SUT697	-0.281759	0.4622735	0.6119415	2.38	1.96	22.00	25.75
ORF-T	YLR106C	-0.281756	0.3196079	0.4845481	344.40	283.28	3125.50	3774.75
ORF-T	YBR172C	-0.28148	0.2408911	0.4005848	82.97	68.24	773.25	917.75
ORF-T	YOL112W	-0.281405	0.2878191	0.4512388	56.54	46.58	533.75	599.50
ORF-T	YPL078C	-0.281175	0.3429875	0.5074363	164.71	135.53	1481.25	1734.75

TABLE S1: Differential expression data for RRP6 RNA-Seq dataset Page 117

Class	Transcript name	RRP6 KO_vs_WT log2_fold_change	RRP6 KO_vs_WT p-value	RRP6 KO_vs_WT FDR	Ave Norm Reads in WT	Ave Norm Reads in RRP6	Average RAW read counts in WT	Average RAW read counts in RRP6
AST	AS_YAL059C-A	-0.280726	0.3123843	0.477534	62.40	51.40	585.50	665.75
SUT	SUT379	-0.280656	0.497535	0.6403501	2.01	1.62	17.25	21.25
ORF-T	YIL137C	-0.280568	0.3173245	0.4827124	43.53	35.77	385.25	477.75
ORF-T	YDR435C	-0.280554	0.3747327	0.5366095	17.06	14.04	151.75	178.50
NUT	NUT1427	-0.280465	0.3151411	0.4806235	119.27	98.19	1067.25	1260.00
ORF-T	YOL057W	-0.280191	0.3194449	0.4845481	115.45	95.04	1065.25	1267.75
ORF-T	YOL117W	-0.280031	0.3951438	0.5560466	11.18	9.14	98.50	125.00
ORF-T	YOR008C	-0.279903	0.2566972	0.4181881	141.21	116.36	1322.50	1490.50
AST	AS_YMR158W-B	-0.279829	0.428014	0.5829213	3.88	3.18	34.50	41.25
ORF-T	YOR141C	-0.279594	0.2688378	0.4318879	59.35	48.87	545.25	651.50
ORF-T	YDL194W	-0.279557	0.3554269	0.5195827	53.07	43.72	489.75	571.00
ORF-T	YBR198C	-0.279475	0.2379687	0.3975606	95.62	78.80	899.50	1041.50
ORF-T	YNL236W	-0.279431	0.318092	0.4832239	16.31	13.38	141.50	174.25
ORF-T	YMR243C	-0.279372	0.3180365	0.4832122	401.00	330.46	3735.00	4162.25
ORF-T	YJL179W	-0.27936	0.2989864	0.4637113	18.55	15.26	170.00	202.00
AST	AS_YCR045C	-0.279157	0.38258	0.543831	11.41	9.33	99.00	125.00
ORF-T	YOR216C	-0.279107	0.456738	0.606899	8.72	7.16	78.25	95.25
ORF-T	YNL198C	-0.279095	0.3115077	0.476772	19.06	15.71	178.00	208.50
ORF-T	YOR223W	-0.279026	0.3922687	0.5533135	9.08	7.50	83.50	95.25
SRT	SRT139	-0.279015	0.417725	0.5743521	2.67	2.21	24.75	29.00
ORF-T	YKR068C	-0.278648	0.2225699	0.3786966	48.96	40.35	452.75	537.25
ORF-T	YGR121C	-0.278622	0.5383849	0.672581	25.69	21.21	233.00	258.00
SUT	SUT513	-0.278589	0.4244928	0.5796325	4.05	3.36	39.00	44.50
SRT	SRT388	-0.278499	0.4078485	0.566026	5.76	4.76	53.75	62.50
ORF-T	YLR056W	-0.278491	0.3155468	0.481024	267.73	220.70	2393.00	2872.75
AST	AS_YER088W-B	-0.278395	0.3548852	0.5193995	8.98	7.37	81.25	98.25
SUT	SUT013	-0.27838	0.3814085	0.5427778	5.44	4.48	49.25	57.75
ORF-T	YOL103W	-0.278072	0.2510597	0.4123025	145.35	119.89	1319.00	1531.50
ORF-T	YLR401C	-0.277833	0.2679305	0.4307734	68.33	56.40	644.00	740.00

Class	Transcript name	RRP6 KO_vs_WT log2_fold _change	RRP6 KO_vs_WT p-value	RRP6 KO_vs_WT FDR	Ave Norm Reads in WT	Ave Norm Reads in RRP6	Average RAW read counts in WT	Average RAW read counts in RRP6
ORF-T	YPL242C	-0.277813	0.3720312	0.5343551	110.43	91.03	1008.25	1256.25
ORF-T	YER189W	-0.277726	0.672008	0.7762347	1.32	1.11	12.25	13.25
ORF-T	YBR077C	-0.277675	0.254196	0.4157847	28.85	23.83	265.00	306.75
ORF-T	YDR298C	-0.277461	0.3657777	0.5285144	160.23	132.17	1413.75	1701.00
ORF-T	YGL220W	-0.277434	0.2596523	0.4218356	26.15	21.55	237.75	282.75
ORF-T	YER047C	-0.277407	0.2643594	0.4271432	22.24	18.34	204.00	241.50
ORF-T	YIR008C	-0.27734	0.2349567	0.3937682	77.15	63.72	723.50	821.50
ORF-T	YBR283C	-0.277244	0.301523	0.4668511	589.74	486.66	5419.25	6222.00
AST	AS_YJL127W-A	-0.2772	0.44025	0.5933881	3.56	2.92	32.25	38.50
ORF-T	YCL001W	-0.277142	0.2797958	0.4435348	66.93	55.24	602.50	701.50
ORF-T	YGL176C	-0.277127	0.263799	0.4267848	26.78	22.11	254.25	296.00
ORF-T	YDL102W	-0.277108	0.237591	0.3972114	58.52	48.34	545.50	620.00
ORF-T	YPL231W	-0.276964	0.4108528	0.5682016	1789.26	1476.79	17433.00	19059.00
ORF-T	YBL023C	-0.276942	0.2759621	0.4396402	124.62	102.92	1182.00	1328.00
ORF-T	YNR023W	-0.276646	0.2899404	0.4538277	14.14	11.68	129.25	151.00
ORF-T	YNL041C	-0.276535	0.2752487	0.4388313	42.09	34.71	383.00	460.75
ORF-T	YMR246W	-0.276477	0.3225597	0.4868571	209.88	173.28	1956.25	2328.50
AST	AS_YCL063W	-0.276414	0.4063276	0.5647301	6.13	5.03	55.00	67.00
ORF-T	YBR202W	-0.276331	0.2618517	0.4242605	53.45	44.12	499.75	597.00
ORF-T	YOR340C	-0.276315	0.2836942	0.4474151	163.18	134.78	1569.25	1783.75
SUT	SUT686	-0.276177	0.3433489	0.507822	29.69	24.53	269.75	312.00
ORF-T	YBL008W-A	-0.275854	0.5806	0.7069271	0.80	0.64	7.25	8.75
ORF-T	YLR390W	-0.27581	0.4751471	0.6224193	3.05	2.50	26.25	31.75
ORF-T	YBR062C	-0.275596	0.3178978	0.4830921	31.54	26.06	283.00	331.75
ORF-T	YPL224C	-0.275562	0.2453327	0.4058292	57.62	47.58	521.00	620.50
ORF-T	YDR363W-A	-0.27531	0.2530973	0.4144581	43.89	36.26	400.50	472.00
SUT	SUT047	-0.275077	0.482538	0.6285043	1.82	1.51	16.00	18.75
ORF-T	YHR109W	-0.275004	0.4428671	0.5953606	5.84	4.80	52.25	62.75
ORF-T	YIL150C	-0.274904	0.3368245	0.5014081	14.01	11.57	130.75	154.25

TABLE S1: Differential expression data for RRP6 RNA-Seq dataset Page 119

Class	Transcript name	RRP6 KO_vs_WT log2_fold _change	RRP6 KO_vs_WT p-value	RRP6 KO_vs_WT FDR	Ave Norm Reads in WT	Ave Norm Reads in RRP6	Average RAW read counts in WT	Average RAW read counts in RRP6
ORF-T	YNL224C	-0.274806	0.2639786	0.4269383	31.50	26.01	288.50	345.25
AST	AS_YNL170W	-0.274723	0.3647603	0.5279658	6.45	5.33	61.25	72.50
AST	AS_YNL008C	-0.274278	0.4296583	0.5842333	2.96	2.47	27.00	31.25
ORF-T	YDR486C	-0.273871	0.3321499	0.4963389	13.18	10.82	115.25	144.50
ORF-T	YFL034W	-0.273858	0.2515022	0.412558	72.52	60.02	683.25	782.50
ORF-T	YBR262C	-0.273644	0.3727408	0.5348965	10.80	8.90	95.50	115.75
ORF-T	YHR043C	-0.273564	0.3846852	0.5458232	39.22	32.45	355.50	411.50
ORF-T	YOL084W	-0.27353	0.5386262	0.6726323	9.54	7.92	83.75	93.75
ORF-T	YER170W	-0.273458	0.3265425	0.491031	27.91	23.10	253.00	292.50
ORF-T	YPL005W	-0.273389	0.3587513	0.522558	22.59	18.71	205.25	239.00
ORF-T	YOR311C	-0.27336	0.2774921	0.4413612	38.80	32.06	351.50	429.25
ORF-T	YLR119W	-0.273287	0.4602781	0.6101653	5.29	4.34	46.25	56.75
ORF-T	YML088W	-0.273195	0.2515687	0.412558	38.79	32.09	358.00	422.00
ORF-T	YGR245C	-0.273027	0.3510244	0.5156949	225.28	186.49	2145.50	2416.25
ORF-T	YFL010C	-0.273024	0.2646722	0.4274431	129.32	107.06	1230.25	1414.50
ORF-T	YIR039C	-0.272869	0.3571166	0.5210771	24.09	19.94	214.75	252.25
ORF-T	YFL004W	-0.272697	0.2759741	0.4396402	149.58	123.83	1367.25	1596.25
ORF-T	YBL018C	-0.272565	0.3423652	0.5070604	26.44	21.90	235.00	275.50
ORF-T	YDR068W	-0.272538	0.3554436	0.5195827	16.00	13.23	145.75	173.75
ORF-T	YIL019W	-0.272535	0.3126221	0.4777935	23.31	19.28	218.25	262.75
ORF-T	YDR392W	-0.272502	0.3496266	0.5140907	15.28	12.64	136.00	161.75
ORF-T	YBL029C-A	-0.272441	0.4010807	0.5606457	8.74	7.23	78.25	91.75
ORF-T	YBR268W	-0.272405	0.3012884	0.4666246	24.48	20.31	225.25	256.75
ORF-T	YOR320C	-0.272332	0.2719267	0.4351172	98.80	81.84	900.50	1040.00
ORF-T	YPL178W	-0.2723	0.2386854	0.3982043	85.42	70.77	796.50	912.25
ORF-T	YIL071C	-0.272251	0.3139626	0.4792611	16.31	13.49	149.75	178.25
ORF-T	YDR041W	-0.272167	0.3051197	0.4706637	49.51	41.02	442.25	510.50
ORF-T	YOR283W	-0.272056	0.2777701	0.4415944	59.77	49.56	556.25	629.00
ORF-T	YNR066C	-0.271601	0.3578332	0.521519	13.99	11.54	130.25	161.25

Class	Transcript name	RRP6 KO_vs_WT log2_fold _change	RRP6 KO_vs_WT p-value	RRP6 KO_vs_WT FDR	Ave Norm Reads in WT	Ave Norm Reads in RRP6	Average RAW read counts in WT	Average RAW read counts in RRP6
ORF-T	YNL221C	-0.271591	0.2646032	0.4274001	81.69	67.71	782.00	903.00
ORF-T	YJR034W	-0.271493	0.4429781	0.5953606	9.11	7.48	77.25	99.25
ORF-T	YAL026C	-0.271489	0.2685697	0.431526	204.05	169.05	1881.25	2222.50
ORF-T	YBL019W	-0.271332	0.3439374	0.5083941	18.20	15.10	167.25	194.25
ORF-T	YKL140W	-0.271225	0.3123908	0.477534	82.60	68.42	744.00	894.50
ORF-T	YAL031C	-0.270995	0.3280442	0.492766	15.90	13.12	140.75	174.25
SUT	SUT692	-0.270825	0.5254417	0.6633973	2.11	1.73	19.25	23.50
ORF-T	YHR122W	-0.270747	0.2637191	0.4267848	42.09	34.88	391.00	463.75
ORF-T	YKL023W	-0.270653	0.3983512	0.5591556	10.23	8.46	93.00	112.00
ORF-T	YOR094W	-0.27061	0.3298929	0.4943736	15.53	12.86	142.00	168.75
AST	AS_YMR153C-A	-0.270599	0.3370478	0.5014456	8.86	7.37	82.75	96.25
ORF-T	YLR328W	-0.270458	0.2726234	0.4358171	39.92	33.06	359.75	432.75
ORF-T	YBL045C	-0.270436	0.4528382	0.6037842	102.72	85.13	895.25	1087.75
ORF-T	YGL100W	-0.270329	0.2472712	0.4078984	88.35	73.28	812.00	940.00
ORF-T	YKL094W	-0.270082	0.2733623	0.4366602	121.21	100.56	1113.75	1279.25
ORF-T	YNL161W	-0.270037	0.2733296	0.4366602	29.54	24.53	269.25	312.50
sn/snoRNA	SNR3	-0.269967	0.4525107	0.6035811	2733.83	2267.27	23825.50	28090.50
ORF-T	YKL023C-A	-0.269913	0.3855914	0.5465707	20.21	16.77	180.50	210.50
ORF-T	YLR321C	-0.269626	0.3213851	0.4857385	25.91	21.47	238.50	285.50
ORF-T	YBR142W	-0.269325	0.2725595	0.435784	91.88	76.24	869.75	1020.25
ORF-T	YLR115W	-0.26929	0.3344111	0.4984778	26.61	22.03	239.00	294.50
ORF-T	YOR101W	-0.26904	0.2741141	0.4374373	50.55	41.94	465.50	551.00
ORF-T	YBR193C	-0.269	0.3939507	0.5550648	7.49	6.15	65.75	82.25
ORF-T	YNL002C	-0.268987	0.3227137	0.4868709	170.43	141.52	1635.50	1829.75
ORF-T	YCR073W-A	-0.268861	0.3662395	0.5288028	59.14	49.06	540.75	654.00
ORF-T	YGR262C	-0.268732	0.2244535	0.3811061	43.45	36.06	397.25	470.25
ORF-T	YGL226W	-0.268726	0.3961775	0.5570349	6.58	5.41	57.25	70.25
ORF-T	YJL183W	-0.268639	0.2953585	0.4600549	180.50	149.85	1665.75	1939.25
NUT	NUT1507	-0.268541	0.2560256	0.4179443	98.17	81.55	901.50	1038.50

Class	Transcript name	RRP6 KO_vs_WT log2_fold _change	RRP6 KO_vs_WT p-value	RRP6 KO_vs_WT FDR	Ave Norm Reads in WT	Ave Norm Reads in RRP6	Average RAW read counts in WT	Average RAW read counts in RRP6
ORF-T	YBR102C	-0.268461	0.2946052	0.4592522	29.54	24.46	266.75	329.75
ORF-T	YAL016W	-0.268232	0.3286643	0.4931139	189.10	157.01	1727.25	2047.50
ORF-T	YER121W	-0.26822	0.5772572	0.7035366	7.72	6.44	68.25	74.50
ORF-T	YOR079C	-0.268057	0.256962	0.4184087	34.76	28.87	321.00	380.25
ORF-T	YLR098C	-0.267955	0.2862623	0.4495668	17.37	14.38	155.00	189.00
ORF-T	YIL017C	-0.267867	0.3469105	0.5119097	70.36	58.44	639.75	749.50
ORF-T	YBR041W	-0.267862	0.2845187	0.4480147	145.02	120.47	1329.25	1543.00
AST	AS_YOR169C	-0.267518	0.2640521	0.4269744	86.02	71.55	810.00	904.00
ORF-T	YNL092W	-0.267151	0.4973303	0.64025	3.62	3.01	32.50	37.75
ORF-T	YBR236C	-0.267117	0.250926	0.4123025	48.34	40.20	447.00	523.25
ORF-T	YDR319C	-0.266999	0.3407282	0.5053542	27.57	22.92	248.00	294.00
ORF-T	YKL099C	-0.266985	0.3207395	0.4853447	45.68	37.99	434.00	503.00
ORF-T	YBR103W	-0.266944	0.2692471	0.4323388	51.66	42.96	481.00	559.50
ORF-T	YOR025W	-0.266643	0.4000466	0.5600189	32.10	26.61	287.25	370.25
ORF-T	YGR063C	-0.26653	0.3264058	0.491031	16.12	13.40	146.00	173.00
ORF-T	YDL159W	-0.266503	0.3693854	0.5320802	13.01	10.78	119.75	146.00
ORF-T	YLR046C	-0.266413	0.4189809	0.5747522	20.69	17.20	187.00	222.00
ORF-T	YDL091C	-0.266274	0.4133036	0.5700523	13.24	10.95	114.00	144.00
AST	AS_YKL053W	-0.266046	0.3520951	0.5168161	36.97	30.83	346.00	384.50
ORF-T	YOR297C	-0.265964	0.4000049	0.5600189	5.46	4.49	48.25	60.00
ORF-T	YNR055C	-0.265953	0.271403	0.4344861	117.27	97.49	1078.50	1308.25
ORF-T	YNL315C	-0.265946	0.3308778	0.495176	43.44	36.14	393.25	457.50
ORF-T	YKL046C	-0.265863	0.3136922	0.479134	286.22	238.10	2631.75	2983.50
ORF-T	YNL128W	-0.265757	0.5206381	0.6589	2.71	2.25	26.00	30.75
ORF-T	YIL067C	-0.265751	0.2857486	0.4488999	74.40	61.90	696.00	815.25
ORF-T	YEL006W	-0.265738	0.2457557	0.4062625	33.55	27.91	308.00	365.00
ORF-T	YDR182W-A	-0.265717	0.4237465	0.5790014	3.23	2.72	30.50	35.25
ORF-T	YDL007W	-0.265632	0.3484194	0.5131718	223.25	185.73	2020.25	2338.50
ORF-T	YOR310C	-0.265603	0.2919631	0.4561047	230.77	191.98	2112.00	2499.25

Class	Transcript name	RRP6 KO_vs_WT log2_fold _change	RRP6 KO_vs_WT p-value	RRP6 KO_vs_WT FDR	Ave Norm Reads in WT	Ave Norm Reads in RRP6	Average RAW read counts in WT	Average RAW read counts in RRP6
ORF-T	YNL093W	-0.265569	0.5606801	0.6905947	6.81	5.69	61.25	68.75
ORF-T	YML111W	-0.265417	0.2523633	0.4133905	35.47	29.47	318.50	386.75
ORF-T	YPL067C	-0.265279	0.4093936	0.5670908	9.01	7.47	81.50	100.00
ORF-T	YNR002C	-0.265023	0.5523117	0.6833756	2.90	2.41	25.00	29.75
ORF-T	YMR277W	-0.264808	0.2911257	0.4551462	51.21	42.59	471.00	574.50
sn/snoRNA	SNR70	-0.264719	0.3942244	0.5553728	2381.34	1982.13	20989.00	24918.50
ORF-T	YML086C	-0.264433	0.3115204	0.476772	91.50	76.19	852.25	998.25
ORF-T	YIL139C	-0.264221	0.3345887	0.4986245	28.73	23.94	272.25	316.75
ORF-T	YHR057C	-0.264219	0.2654225	0.4283729	48.31	40.22	437.50	521.50
ORF-T	YNL154C	-0.264213	0.309902	0.4756548	117.80	98.06	1088.75	1314.50
ORF-T	YPL012W	-0.264207	0.3283686	0.4927843	169.50	141.14	1614.50	1906.25
ORF-T	YNL126W	-0.264004	0.3076539	0.4734293	26.98	22.48	245.00	290.25
ORF-T	YER096W	-0.263961	0.5551706	0.685735	8.42	7.05	78.50	87.75
ORF-T	YDL131W	-0.263936	0.3042351	0.4698892	25.35	21.06	230.50	282.75
ORF-T	YNL004W	-0.26378	0.2535857	0.4150364	73.35	61.09	665.50	794.25
ORF-T	YLR191W	-0.263622	0.2744979	0.437842	29.47	24.60	273.00	314.25
ORF-T	YDL043C	-0.263594	0.3062235	0.4719483	14.93	12.39	135.50	166.00
ORF-T	YNL199C	-0.263456	0.3095172	0.4752659	32.56	27.12	302.50	360.50
ORF-T	YGL172W	-0.263346	0.2798123	0.4435348	159.61	133.02	1489.25	1721.25
SUT	SUT760	-0.263291	0.5249394	0.6630048	3.00	2.45	25.00	32.00
ORF-T	YPR146C	-0.263079	0.3307178	0.4950212	11.29	9.42	104.25	122.50
ORF-T	YDL039C	-0.263039	0.6442779	0.7564642	1.37	1.11	11.75	15.00
ORF-T	YJL013C	-0.263018	0.314025	0.4792838	17.73	14.77	162.75	194.50
ORF-T	YNL212W	-0.262793	0.3256701	0.4903041	57.04	47.56	530.25	617.25
AST	AS_YOL046C	-0.262596	0.3739664	0.5358927	14.27	11.88	131.50	157.75
ORF-T	YGL066W	-0.262553	0.3705138	0.5331424	15.59	12.97	142.00	172.25
ORF-T	YGR092W	-0.262501	0.3524299	0.5172323	80.65	67.22	762.50	918.75
ORF-T	YLR309C	-0.262478	0.2870564	0.4505333	56.62	47.22	525.25	614.25
ORF-T	YPR075C	-0.262426	0.2668703	0.4296854	101.30	84.50	929.50	1076.00

Class	Transcript name	RRP6 KO_vs_WT log2_fold _change	RRP6 KO_vs_WT p-value	RRP6 KO_vs_WT FDR	Ave Norm Reads in WT	Ave Norm Reads in RRP6	Average RAW read counts in WT	Average RAW read counts in RRP6
ORF-T	YNL159C	-0.262352	0.3491294	0.5138225	32.80	27.34	293.75	349.25
ORF-T	YPR028W	-0.262337	0.4766642	0.6237998	172.92	144.12	1480.75	1879.00
AST	AS_YGR228W	-0.262217	0.2562235	0.417964	38.57	32.20	356.50	413.00
ORF-T	YNL110C	-0.262078	0.3136556	0.479134	34.79	29.01	324.50	386.25
ORF-T	YOL108C	-0.262066	0.3316494	0.495974	13.16	10.98	120.25	142.25
ORF-T	YML018C	-0.261876	0.3187036	0.4837886	82.85	69.12	768.25	902.75
SUT	SUT366	-0.261776	0.3289103	0.4932677	26.90	22.40	242.50	295.75
ORF-T	YLR144C	-0.261679	0.2511884	0.4123025	40.81	34.00	364.00	444.75
ORF-T	YDR016C	-0.261649	0.3640569	0.527817	21.09	17.61	189.00	219.75
ORF-T	YIL036W	-0.261486	0.4001296	0.5600189	36.49	30.37	308.75	394.50
sn/snoRNA	SNR63	-0.261438	0.4031922	0.562039	5877.72	4903.51	51504.50	61784.00
ORF-T	YBR264C	-0.261378	0.322866	0.4870278	11.68	9.73	105.50	126.50
ORF-T	YJL002C	-0.261368	0.3555256	0.5195827	212.77	177.52	1916.50	2250.50
ORF-T	YPR079W	-0.261281	0.3641997	0.527817	14.59	12.19	130.25	152.50
ORF-T	YEL056W	-0.261228	0.410065	0.5675399	31.76	26.49	285.50	340.50
ORF-T	YKL028W	-0.260793	0.304321	0.4699158	67.21	56.11	631.50	740.00
ORF-T	YOR308C	-0.260526	0.3773839	0.5388756	14.24	11.84	130.50	160.50
ORF-T	YGL188C	-0.260448	0.5635463	0.6929054	6.11	5.09	52.00	62.75
ORF-T	YFR008W	-0.260359	0.4838596	0.6294982	2.93	2.43	26.50	32.25
ORF-T	YBR104W	-0.260307	0.4476635	0.5994209	36.21	30.21	346.00	417.75
ORF-T	YBR046C	-0.260299	0.4557534	0.6060684	41.93	35.07	391.00	435.25
ORF-T	YJL197W	-0.260133	0.2837465	0.4474276	120.61	100.74	1126.00	1318.00
ORF-T	YKL060C	-0.259954	0.3732313	0.5354481	2866.46	2393.78	25315.50	31157.25
ORF-T	YDR232W	-0.259855	0.2832165	0.4470113	104.72	87.46	956.00	1138.25
ORF-T	YGL095C	-0.259853	0.3508687	0.5155413	53.37	44.55	491.50	599.00
ORF-T	YPL103C	-0.259786	0.3321887	0.4963389	24.30	20.30	229.00	271.00
ORF-T	YOR087W	-0.259754	0.3423815	0.5070604	79.95	66.74	718.00	877.25
ORF-T	YJL218W	-0.259738	0.5603699	0.690493	6.33	5.27	59.75	72.50
ORF-T	YPR107C	-0.259694	0.3623256	0.5261652	17.88	14.94	163.00	191.75

TABLE S1: Differential expression data for RRP6 RNA-Seq dataset Page 124

Class	Transcript name	RRP6 KO_vs_WT log2_fold _change	RRP6 KO_vs_WT p-value	RRP6 KO_vs_WT FDR	Ave Norm Reads in WT	Ave Norm Reads in RRP6	Average RAW read counts in WT	Average RAW read counts in RRP6
ORF-T	YML053C	-0.259328	0.4236662	0.5789741	10.71	8.86	94.75	125.00
ORF-T	YDR135C	-0.259165	0.3645388	0.5279324	326.18	272.54	2963.00	3517.75
ORF-T	YHR031C	-0.259141	0.4060089	0.5644683	43.23	36.09	412.50	511.00
ORF-T	YHR113W	-0.258936	0.2798703	0.4435348	83.62	69.92	768.75	892.25
SUT	SUT776	-0.258865	0.5688574	0.69726	1.63	1.29	13.50	18.00
ORF-T	YGR174C	-0.258856	0.4082696	0.5663587	26.76	22.38	240.50	278.75
ORF-T	YJR079W	-0.258819	0.6181177	0.7360757	2.73	2.32	25.75	28.25
ORF-T	YFR039C	-0.258809	0.2927766	0.4571631	60.24	50.31	543.75	666.50
ORF-T	YNR052C	-0.258757	0.2702896	0.4333228	82.15	68.68	770.25	905.75
sn/snoRNA	snR53	-0.258692	0.4779018	0.6242376	128.48	107.39	1122.25	1352.00
ORF-T	YML119W	-0.25864	0.4196855	0.5753275	16.30	13.55	145.50	188.50
ORF-T	YDL221W	-0.2586	0.5383141	0.672581	1.65	1.36	14.25	17.50
ORF-T	YLR288C	-0.258497	0.3254516	0.4901086	21.38	17.91	200.00	231.75
ORF-T	YGL038C	-0.258342	0.2857136	0.4488999	79.09	66.09	714.75	865.50
ORF-T	YKR044W	-0.25831	0.3553593	0.5195827	37.33	31.20	352.75	428.25
ORF-T	YDR129C	-0.25816	0.40197	0.5608572	350.70	293.28	3270.50	3710.00
NUT	NUT0891	-0.257998	0.4093139	0.5670908	5879.53	4916.73	51520.25	61959.25
ORF-T	YPL051W	-0.257905	0.3593289	0.5230941	11.23	9.38	102.25	123.50
ORF-T	YLR020C	-0.257799	0.2711434	0.4341394	68.03	56.94	628.50	733.25
CUT	CUT266	-0.257775	0.6256378	0.7417037	1.71	1.41	14.00	17.75
ORF-T	YPL154C	-0.257728	0.4041745	0.5630969	357.82	299.31	3202.50	3717.00
SUT	SUT821	-0.257725	0.6212774	0.7381833	1.02	0.87	10.00	11.50
ORF-T	YOR284W	-0.257662	0.3590176	0.5227919	9.94	8.31	91.50	108.75
AST	AS_YDL071C	-0.25751	0.3654119	0.5283644	9.75	8.21	90.75	104.25
CUT	CUT061	-0.257298	0.541973	0.6750563	3.18	2.69	29.75	33.75
ORF-T	YJL117W	-0.257278	0.3575833	0.5214897	138.19	115.65	1245.00	1450.50
ORF-T	YJR075W	-0.257158	0.3072492	0.473023	157.87	132.13	1452.25	1685.50
ORF-T	YJR041C	-0.257125	0.3031712	0.4691082	27.66	23.14	250.00	300.25
NUT	NUT1068	-0.257108	0.5659059	0.6948565	25.71	21.55	233.25	263.25

TABLE S1: Differential expression data for RRP6 RNA-Seq dataset Page 125

Class	Transcript name	RRP6 KO_vs_WT log2_fold _change	RRP6 KO_vs_WT p-value	RRP6 KO_vs_WT FDR	Ave Norm Reads in WT	Ave Norm Reads in RRP6	Average RAW read counts in WT	Average RAW read counts in RRP6
ORF-T	YJL070C	-0.257084	0.3461795	0.5111838	47.09	39.42	422.50	494.25
ORF-T	YMR130W	-0.256876	0.304189	0.4698892	21.25	17.75	193.00	235.00
ORF-T	YFR013W	-0.256769	0.2573471	0.4189003	49.63	41.52	454.25	544.25
ORF-T	YEL011W	-0.256763	0.6492079	0.7598408	269.70	225.72	2224.00	2683.00
NUT	NUT1497	-0.256681	0.4604758	0.6101653	5.84	4.88	54.00	64.75
AST	AS_YDL056W	-0.256662	0.6024907	0.7247425	0.76	0.66	7.50	8.75
SUT	SUT646	-0.256615	0.4133501	0.5700523	15.53	12.96	133.25	166.00
ORF-T	YOR005C	-0.256477	0.3471433	0.512039	21.40	17.91	195.50	235.25
ORF-T	YMR235C	-0.256429	0.3265363	0.491031	205.58	172.08	1879.00	2295.50
ORF-T	YDL052C	-0.256238	0.2913132	0.455301	32.10	26.82	280.75	346.50
ORF-T	YLL043W	-0.256182	0.2998938	0.4647501	158.97	133.15	1492.25	1737.75
ORF-T	YDL139C	-0.255914	0.5190397	0.6580662	2.22	1.83	19.50	23.75
ORF-T	YHR041C	-0.255773	0.2873043	0.45065	48.38	40.51	440.25	524.75
ORF-T	YKL216W	-0.25575	0.5355506	0.6713689	882.21	738.96	8140.25	8712.75
SRT	SRT33	-0.255742	0.5401833	0.6737425	3.04	2.54	28.75	35.25
ORF-T	YNL009W	-0.255735	0.4248416	0.5799717	8.93	7.49	80.50	95.50
ORF-T	YNL245C	-0.255691	0.3795959	0.5408853	9.02	7.53	83.00	101.25
ORF-T	YKL058W	-0.255676	0.270891	0.434011	76.17	63.83	693.00	810.75
ORF-T	YLR088W	-0.255668	0.2947766	0.4594324	118.66	99.38	1087.00	1308.00
NUT	NUT1171	-0.255461	0.5547771	0.6854707	1.92	1.64	19.50	22.50
ORF-T	YOL068C	-0.255404	0.2779582	0.4418239	58.24	48.82	543.50	639.75
ORF-T	YOL048C	-0.255201	0.532035	0.6684945	14.86	12.44	124.75	151.25
ORF-T	YPL084W	-0.254892	0.2781547	0.4419774	47.62	39.91	429.25	511.00
ORF-T	YGL012W	-0.25485	0.3550016	0.5194944	469.09	393.18	4291.00	4940.00
ORF-T	YNL098C	-0.254844	0.5152683	0.6550638	39.47	33.08	349.75	413.00
ORF-T	YML097C	-0.254839	0.3683208	0.5306682	17.87	14.98	167.00	198.25
ORF-T	YIL004C	-0.254792	0.3099945	0.4757228	21.52	18.05	197.00	234.00
ORF-T	YMR161W	-0.25476	0.3152223	0.480667	21.39	17.91	191.25	231.50
ORF-T	YOR209C	-0.254627	0.2839596	0.4476237	159.51	133.69	1461.25	1763.25

Class	Transcript name	RRP6 KO_vs_WT log2_fold_change	RRP6 KO_vs_WT p-value	RRP6 KO_vs_WT FDR	Ave Norm Reads in WT	Ave Norm Reads in RRP6	Average RAW read counts in WT	Average RAW read counts in RRP6
ORF-T	YLR003C	-0.254589	0.3273069	0.4918139	28.82	24.13	260.00	316.75
ORF-T	YGR001C	-0.254542	0.3803223	0.5417294	19.44	16.27	170.00	207.00
ORF-T	YJL053W	-0.254528	0.4014716	0.5608572	17.18	14.36	149.50	183.25
ORF-T	YPL028W	-0.254482	0.3278458	0.4925502	422.21	353.96	3914.75	4554.75
ORF-T	YOR330C	-0.254422	0.334945	0.4990522	95.62	80.14	880.00	1066.75
ORF-T	YOR326W	-0.254421	0.3373886	0.5017293	371.69	311.63	3461.25	4013.50
ORF-T	YNL297C	-0.25442	0.317502	0.482831	69.94	58.59	626.50	773.25
ORF-T	YGR091W	-0.2543	0.3202097	0.4849068	16.65	13.98	155.75	183.50
ORF-T	YKR035W-A	-0.254121	0.3959019	0.5568026	8.43	7.08	77.00	90.50
ORF-T	YPL008W	-0.254097	0.2614045	0.4237948	52.90	44.39	492.00	578.50
ORF-T	YPL174C	-0.254044	0.351391	0.516008	26.86	22.54	245.50	291.50
AST	AS_YGR219W	-0.253997	0.4099868	0.5675234	13.06	10.93	113.50	138.25
SUT	SUT471	-0.253988	0.4542969	0.6050092	2.97	2.48	26.75	32.25
ORF-T	YPL093W	-0.253864	0.3554141	0.5195827	315.54	264.65	2973.25	3499.50
ORF-T	YJR085C	-0.25376	0.3992284	0.5595387	91.70	76.88	815.50	994.25
ORF-T	YDL127W	-0.253677	0.4640688	0.6134054	4.66	3.88	41.25	51.25
ORF-T	YNR056C	-0.25357	0.5536484	0.6845868	3.07	2.56	28.50	35.50
ORF-T	YDL150W	-0.253528	0.3414108	0.5059945	53.06	44.58	510.25	581.25
ORF-T	YPR067W	-0.253366	0.4284642	0.5829443	10.33	8.61	90.00	114.25
ORF-T	YOR054C	-0.253191	0.4209646	0.5762606	44.20	37.12	404.50	464.00
ORF-T	YGR172C	-0.253177	0.3427761	0.5073469	43.34	36.35	388.50	472.25
ORF-T	YMR193W	-0.253101	0.4335677	0.5880426	14.98	12.57	133.75	158.50
SUT	SUT745	-0.252983	0.5337072	0.6697241	2.43	2.00	20.75	26.25
ORF-T	YBL040C	-0.252785	0.3484111	0.5131718	125.80	105.60	1131.50	1321.00
ORF-T	YDR162C	-0.252752	0.2710029	0.4341145	37.78	31.72	350.00	413.75
ORF-T	YFL013C	-0.252616	0.5070234	0.6485886	8.33	6.95	71.75	91.25
ORF-T	YKL154W	-0.252542	0.2958558	0.4604034	48.63	40.85	445.50	522.50
ORF-T	YKL100C	-0.252212	0.3184208	0.4835066	67.54	56.64	595.00	747.75
ORF-T	YKL052C	-0.252189	0.3574421	0.5214011	85.57	71.93	797.25	898.25

Class	Transcript name	RRP6 KO_vs_WT log2_fold _change	RRP6 KO_vs_WT p-value	RRP6 KO_vs_WT FDR	Ave Norm Reads in WT	Ave Norm Reads in RRP6	Average RAW read counts in WT	Average RAW read counts in RRP6
ORF-T	YPL232W	-0.251731	0.3027747	0.4686385	38.44	32.28	349.75	421.00
ORF-T	YPR105C	-0.251421	0.3511263	0.5157469	90.27	75.82	837.75	1008.75
ORF-T	YNL215W	-0.251188	0.3552165	0.5195827	19.71	16.53	182.00	222.50
ORF-T	YOR257W	-0.250965	0.4046977	0.5633589	8.15	6.79	72.25	91.00
ORF-T	YDL188C	-0.250929	0.3938313	0.5550648	35.69	29.96	322.25	395.50
ORF-T	YDR489W	-0.25081	0.4094523	0.5670908	13.90	11.60	120.75	156.50
ORF-T	YMR084W	-0.250767	0.4589056	0.6088332	6.55	5.51	60.25	71.75
ORF-T	YJL081C	-0.250612	0.3379487	0.5021983	57.21	48.09	529.75	628.50
AST	AS_YNR003W-A	-0.250548	0.4042972	0.5631122	8.02	6.76	74.00	87.25
CUT	CUT090	-0.250509	0.5036192	0.6458725	15.42	13.01	144.75	163.50
ORF-T	YMR049C	-0.250501	0.3101968	0.4757228	91.69	77.11	861.25	1010.25
ORF-T	YNL104C	-0.250349	0.3271261	0.4916295	135.37	113.73	1215.00	1524.25
ORF-T	YDR221W	-0.250228	0.3294885	0.4940613	66.34	55.73	608.50	752.75
ORF-T	YDR109C	-0.250105	0.4020054	0.5608572	18.40	15.52	180.50	208.00
ORF-T	YKR021W	-0.24995	0.3977749	0.5585021	14.14	11.82	121.50	154.00
ORF-T	YMR103C	-0.249944	0.3949798	0.5558934	35.53	29.87	324.00	389.50
ORF-T	YHR114W	-0.249933	0.3196364	0.4845481	63.57	53.49	600.25	701.25
ORF-T	YLR274W	-0.249838	0.2974868	0.4622987	150.58	126.66	1412.25	1664.50
ORF-T	YGL061C	-0.249692	0.4668625	0.6158872	5.95	4.99	55.75	67.50
ORF-T	YDR367W	-0.249674	0.3357627	0.5001228	35.50	29.83	312.75	380.25
ORF-T	YML050W	-0.24945	0.4152049	0.5719064	8.53	7.14	77.00	96.00
ORF-T	YMR098C	-0.249429	0.3414055	0.5059945	33.74	28.40	306.50	359.75
ORF-T	YBL022C	-0.249182	0.4207066	0.5761896	141.06	118.67	1262.00	1530.25
ORF-T	YLR327C	-0.249113	0.5847883	0.7099694	153.26	128.96	1340.00	1568.50
ORF-T	YBR230C	-0.249036	0.4665205	0.6155971	49.72	41.83	426.25	515.00
ORF-T	YGL252C	-0.24879	0.3230654	0.4871828	67.14	56.48	604.25	735.50
ORF-T	YOR122C	-0.24843	0.4273508	0.5825095	253.79	213.62	2266.00	2767.25
ORF-T	YER136W	-0.248115	0.3604036	0.5243558	92.49	77.88	839.50	999.75
SUT	SUT305	-0.247779	0.548413	0.6801453	3.34	2.75	28.50	37.75

Class	Transcript name	RRP6 KO_vs_WT log2_fold _change	RRP6 KO_vs_WT p-value	RRP6 KO_vs_WT FDR	Ave Norm Reads in WT	Ave Norm Reads in RRP6	Average RAW read counts in WT	Average RAW read counts in RRP6
ORF-T	YIL043C	-0.247486	0.3663792	0.528853	369.93	311.61	3361.00	4030.00
AST	AS_YLR299C-A	-0.247301	0.4895422	0.633784	2.29	1.94	21.50	25.50
ORF-T	YGR120C	-0.247133	0.4414339	0.5943944	7.65	6.36	66.50	87.50
ORF-T	YHL011C	-0.247083	0.3400247	0.5045333	175.98	148.32	1638.00	1915.50
ORF-T	YHL032C	-0.24708	0.3176062	0.4828496	78.62	66.26	712.50	843.50
ORF-T	YJR058C	-0.247007	0.3095309	0.4752659	29.07	24.46	259.00	316.75
ORF-T	YEL020W-A	-0.247005	0.3182703	0.4834218	31.01	26.17	286.00	334.25
ORF-T	YGR217W	-0.246678	0.2797689	0.4435348	91.61	77.25	854.00	1010.50
ORF-T	YBL065W	-0.246501	0.6980339	0.7954566	0.81	0.69	8.25	9.75
ORF-T	YLR168C	-0.246466	0.3259705	0.49061	24.97	21.05	229.50	274.50
ORF-T	YNL330C	-0.246403	0.2871084	0.4505448	56.32	47.51	525.50	618.75
ORF-T	YER024W	-0.246266	0.3911488	0.5520695	21.99	18.52	208.75	256.00
ORF-T	YDL138W	-0.246066	0.3393235	0.5038877	19.96	16.82	179.50	218.25
ORF-T	YER176W	-0.245852	0.3230068	0.4871673	62.04	52.29	579.00	711.25
ORF-T	YPR080W	-0.245536	0.3482748	0.5131506	4362.83	3680.09	40736.00	47946.25
AST	AS_YOR170W	-0.245423	0.3819255	0.5432068	11.22	9.51	103.25	120.00
ORF-T	YMR107W	-0.245418	0.6030912	0.7252053	2.32	1.98	20.50	23.50
ORF-T	YDR441C	-0.245398	0.320583	0.4852535	20.12	16.94	182.00	222.75
ORF-T	YMR134W	-0.245338	0.3342434	0.498375	15.33	12.94	143.25	171.25
ORF-T	YMR119W	-0.245303	0.3361333	0.5004531	32.90	27.72	291.25	358.75
ORF-T	YKL068W-A	-0.245238	0.5211529	0.6593446	2.65	2.24	24.25	28.75
ORF-T	YLL019C	-0.24508	0.3679164	0.530237	46.62	39.28	403.75	510.25
ORF-T	YLR086W	-0.244912	0.3312512	0.4955255	93.66	79.04	861.00	1031.75
sn/snoRNA ET	SNR70-ET	-0.244898	0.4180291	0.5743521	2639.70	2227.57	23272.00	28185.75
ORF-T	YOR191W	-0.244808	0.316959	0.4823747	81.56	68.84	748.00	896.75
ORF-T	YLL006W	-0.244701	0.3803495	0.5417294	21.16	17.82	189.50	233.50
ORF-T	YMR268C	-0.24443	0.4299788	0.5844327	11.33	9.53	106.00	132.00
NUT	NUT1415	-0.244403	0.5847107	0.7099606	1.67	1.34	14.00	18.75
sn/snoRNA	SNR30	-0.244304	0.4343221	0.5888282	12734.35	10750.60	108682.00	136496.50

Class	Transcript name	RRP6 KO_vs_WT log2_fold _change	RRP6 KO_vs_WT p-value	RRP6 KO_vs_WT FDR	Ave Norm Reads in WT	Ave Norm Reads in RRP6	Average RAW read counts in WT	Average RAW read counts in RRP6
ORF-T	YOL080C	-0.244249	0.3717794	0.5342773	18.01	15.22	168.50	201.00
AST	AS_YKR047W	-0.244172	0.3956987	0.5566721	28.28	23.90	260.75	309.25
ORF-T	YDR267C	-0.244124	0.3975405	0.5583105	22.09	18.63	200.25	243.00
ORF-T	YOL082W	-0.244004	0.387335	0.5481717	30.14	25.39	261.00	330.25
ORF-T	YJR068W	-0.243891	0.2902205	0.4540859	90.49	76.40	825.25	1000.50
NUT	NUT0490	-0.243774	0.4353702	0.5892984	12734.59	10754.75	108684.00	136557.00
NUT	NUT1135	-0.243625	0.4933944	0.6371227	2.88	2.40	25.50	32.00
ORF-T	YJL155C	-0.243594	0.3833534	0.5446232	100.36	84.79	920.75	1090.75
ORF-T	YGL161C	-0.243425	0.3953764	0.5562963	90.42	76.38	827.25	981.00
ORF-T	YLL039C	-0.243211	0.4487099	0.6001845	229.88	194.21	1979.25	2413.50
ORF-T	YLR316C	-0.243181	0.3650892	0.5281249	11.27	9.52	102.25	124.00
ORF-T	YPL009C	-0.242968	0.3309194	0.495176	92.28	77.99	855.00	1014.50
ORF-T	YPL050C	-0.242968	0.3772834	0.5388083	232.73	196.72	2155.25	2449.00
ORF-T	YBR152W	-0.242777	0.4011959	0.5607289	11.14	9.46	104.75	121.75
ORF-T	YPL083C	-0.242742	0.4019097	0.5608572	14.34	12.09	125.50	154.75
ORF-T	YBR235W	-0.242507	0.3175461	0.482831	131.32	111.00	1211.25	1459.50
ORF-T	YAL015C	-0.242497	0.4858907	0.6312447	18.87	15.92	175.75	220.75
ORF-T	YFL049W	-0.242235	0.323625	0.4878079	50.12	42.39	471.00	559.50
ORF-T	YDR322C-A	-0.242191	0.3393289	0.5038877	55.19	46.67	492.25	585.75
ORF-T	YDR531W	-0.241859	0.4030826	0.562039	96.79	81.86	893.25	1064.25
SUT	SUT333	-0.241835	0.5436393	0.6762964	2.48	2.08	21.75	26.75
ORF-T	YML010W	-0.241813	0.3664603	0.5288944	152.76	129.23	1397.50	1628.50
ORF-T	YLR055C	-0.241646	0.3596515	0.5234126	36.54	30.92	343.25	409.50
ORF-T	YKL194C	-0.241529	0.4104162	0.56787	21.04	17.84	201.75	231.50
ORF-T	YML049C	-0.241456	0.3617502	0.525784	63.00	53.22	569.50	725.25
ORF-T	YGL131C	-0.241445	0.3398941	0.5045333	31.99	27.01	285.50	357.00
ORF-T	YAL019W	-0.241365	0.3306869	0.4950212	116.06	98.22	1090.25	1284.50
AST	AS_YER165C-A	-0.24134	0.4056775	0.5640999	10.63	8.96	95.00	117.25
ORF-T	YBR288C	-0.241317	0.3059647	0.4716215	61.52	52.06	565.25	670.75

Class	Transcript name	RRP6 KO_vs_WT log2_fold _change	RRP6 KO_vs_WT p-value	RRP6 KO_vs_WT FDR	Ave Norm Reads in WT	Ave Norm Reads in RRP6	Average RAW read counts in WT	Average RAW read counts in RRP6
AST	AS_YKR025W	-0.241259	0.461507	0.6110604	5.79	4.93	55.75	65.50
ORF-T	YER002W	-0.241176	0.309598	0.4752659	39.49	33.46	374.00	438.50
ORF-T	YKR070W	-0.241134	0.3530505	0.5176911	73.80	62.45	684.75	819.00
ORF-T	YCR003W	-0.241128	0.4006669	0.5604557	32.83	27.80	300.50	351.25
ORF-T	YDR164C	-0.240909	0.3525377	0.5173152	38.94	32.93	361.25	442.00
ORF-T	YMR267W	-0.240728	0.3236022	0.4878079	47.54	40.25	434.25	513.00
ORF-T	YOR243C	-0.240706	0.352881	0.5176236	223.69	189.38	2080.75	2413.75
ORF-T	YKL116C	-0.240631	0.4343887	0.5888393	19.82	16.71	179.00	228.00
ORF-T	YBR110W	-0.240525	0.3045277	0.4701252	89.50	75.80	828.75	971.50
ORF-T	YPL076W	-0.240408	0.5411803	0.6744312	2.79	2.30	24.00	31.25
ORF-T	YNL253W	-0.240335	0.3436085	0.5079825	19.63	16.62	185.00	223.50
ORF-T	YHR105W	-0.240302	0.4009286	0.5605521	10.66	9.02	98.75	119.25
ORF-T	YPL155C	-0.240188	0.400149	0.5600189	39.89	33.74	371.00	463.50
AST	AS_YAL019W-A	-0.240064	0.4399512	0.5933881	6.00	5.09	56.50	67.75
ORF-T	YPL176C	-0.239812	0.3215382	0.485897	53.88	45.68	494.75	579.50
ORF-T	YPR057W	-0.23978	0.3253947	0.4901086	24.63	20.86	223.75	270.50
ORF-T	YOL065C	-0.239539	0.5646461	0.6937847	19.35	16.38	179.25	218.75
ORF-T	YJL210W	-0.239359	0.3546736	0.5192629	53.93	45.71	493.75	578.75
ORF-T	YPL094C	-0.238464	0.362923	0.5265798	99.80	84.59	930.75	1124.75
ORF-T	YDR485C	-0.238463	0.428449	0.5829443	27.48	23.25	252.75	315.25
ORF-T	YAL025C	-0.238419	0.3885199	0.5495629	38.40	32.57	370.50	442.00
ORF-T	YDR407C	-0.238227	0.3838792	0.5451399	45.29	38.37	404.50	498.25
ORF-T	YNR011C	-0.23798	0.3870851	0.5479946	22.75	19.31	217.25	259.25
ORF-T	YLR258W	-0.237865	0.6688908	0.7739638	252.72	214.29	2051.25	2601.75
ORF-T	YKL133C	-0.237712	0.4606992	0.6101653	13.50	11.46	119.50	142.50
ORF-T	YIR012W	-0.237543	0.4430539	0.5953832	240.43	204.02	2308.25	2567.25
ORF-T	YPR082C	-0.2375	0.4360546	0.5899583	8.85	7.48	79.50	98.00
ORF-T	YER180C	-0.237317	0.4187856	0.5745624	12.10	10.25	114.25	141.25
ORF-T	YJL006C	-0.237309	0.4079674	0.566113	9.85	8.33	87.75	109.50

Class	Transcript name	RRP6 KO_vs_WT log2_fold _change	RRP6 KO_vs_WT p-value	RRP6 KO_vs_WT FDR	Ave Norm Reads in WT	Ave Norm Reads in RRP6	Average RAW read counts in WT	Average RAW read counts in RRP6
ORF-T	YOR164C	-0.237291	0.3746753	0.5366036	62.67	53.13	571.75	709.00
ORF-T	YDL225W	-0.237238	0.3329847	0.497183	44.81	38.02	419.75	505.25
ORF-T	YDL086W	-0.237189	0.4673698	0.616265	29.11	24.65	258.00	325.25
ORF-T	YLL001W	-0.237169	0.387779	0.5486689	118.92	100.91	1077.75	1274.50
ORF-T	YOR287C	-0.23709	0.4469409	0.5990897	8.78	7.48	84.50	99.50
ORF-T	YOL133W	-0.236952	0.3165674	0.4821548	38.50	32.63	347.50	430.50
ORF-T	YDR342C	-0.236928	0.6152441	0.7335325	233.01	197.70	1951.75	2434.00
ORF-T	YLR342W-A	-0.236603	0.5143396	0.654368	16.55	14.08	153.75	180.25
ORF-T	YHR111W	-0.236593	0.3271354	0.4916295	30.74	26.05	279.50	347.25
ORF-T	YMR162C	-0.236467	0.3102938	0.4757228	41.52	35.22	374.25	457.50
ORF-T	YJR005W	-0.236324	0.3281346	0.492766	59.53	50.51	530.25	650.50
ORF-T	YAR002W	-0.236268	0.3992345	0.5595387	17.58	14.85	155.75	198.25
ORF-T	YLR281C	-0.236181	0.4587701	0.6088332	5.87	5.00	52.25	62.00
ORF-T	YLR414C	-0.236115	0.448079	0.599818	322.85	274.17	3055.25	3512.50
ORF-T	YNL132W	-0.235737	0.3893276	0.5500111	93.70	79.58	884.00	1070.25
ORF-T	YBL057C	-0.235673	0.3084603	0.4742359	61.36	52.11	563.75	682.25
ORF-T	YGL048C	-0.23542	0.3815602	0.5428146	155.49	132.08	1403.50	1695.00
CUT	CUT717	-0.23539	0.3561559	0.5200515	58.96	50.15	541.00	625.75
ORF-T	YPL172C	-0.235325	0.3781769	0.5397022	39.95	33.94	365.50	439.25
ORF-T	YHL040C	-0.235267	0.3722275	0.5344309	31.99	27.14	288.25	362.00
ORF-T	YOR058C	-0.235154	0.4593391	0.6091679	14.70	12.43	134.25	172.75
AST	AS_YML031C-A	-0.235125	0.4198872	0.5755257	8.91	7.59	82.25	97.50
ORF-T	YKL019W	-0.235037	0.3371582	0.5015346	47.74	40.53	427.00	526.25
ORF-T	YGR248W	-0.234982	0.6744462	0.7779817	4.12	3.48	33.00	41.75
ORF-T	YJR107W	-0.234956	0.4069955	0.5651531	12.56	10.65	114.25	141.50
ORF-T	YLR028C	-0.234955	0.3658773	0.5285826	273.86	232.74	2543.25	3005.50
ORF-T	YPL129W	-0.234871	0.349529	0.5140252	29.74	25.24	268.25	332.25
AST	AS_YBR266C	-0.234547	0.4419051	0.5947906	25.09	21.34	236.25	282.50
ORF-T	YGR206W	-0.234504	0.482722	0.6285859	10.63	9.01	95.50	118.75

Class	Transcript name	RRP6 KO_vs_WT log2_fold _change	RRP6 KO_vs_WT p-value	RRP6 KO_vs_WT FDR	Ave Norm Reads in WT	Ave Norm Reads in RRP6	Average RAW read counts in WT	Average RAW read counts in RRP6
ORF-T	YDR159W	-0.234444	0.3199688	0.4846877	39.88	33.90	359.25	434.50
ORF-T	YJR002W	-0.234143	0.3852355	0.5461811	336.16	285.83	3153.00	3713.50
ORF-T	YLR163C	-0.234041	0.404369	0.5631345	74.30	63.18	688.50	821.75
ORF-T	YOL116W	-0.233881	0.4108236	0.5682016	19.65	16.70	172.75	212.50
ORF-T	YLR305C	-0.233666	0.3425755	0.5071988	106.54	90.55	968.25	1217.50
ORF-T	YJR138W	-0.233536	0.4000854	0.5600189	42.96	36.50	400.00	503.75
ORF-T	YGR235C	-0.233287	0.3449788	0.5096346	112.62	95.85	1031.50	1209.25
ORF-T	YOR370C	-0.233257	0.3597991	0.5235518	224.92	191.36	2133.75	2554.50
ORF-T	YOR127W	-0.233179	0.3080646	0.473772	66.76	56.83	621.50	740.00
ORF-T	YDR422C	-0.233116	0.38048	0.5417622	29.85	25.31	263.25	339.50
ORF-T	YBR281C	-0.233043	0.4195411	0.575286	81.51	69.38	756.75	886.50
ORF-T	YGR137W	-0.232978	0.3534912	0.5180359	31.75	26.95	282.50	359.00
ORF-T	YKL098W	-0.232918	0.3663769	0.528853	19.74	16.74	176.50	223.75
ORF-T	YMR153W	-0.232874	0.3118812	0.4772209	45.78	39.00	429.50	507.25
ORF-T	YPR073C	-0.232794	0.3500558	0.5144219	40.22	34.26	374.50	440.25
AST	AS_YIL060W	-0.232783	0.4954862	0.6385282	6.16	5.16	53.25	71.00
ORF-T	YBL061C	-0.232734	0.3346085	0.4986245	71.36	60.79	678.50	795.00
ORF-T	YBR092C	-0.232581	0.6107501	0.7298891	98.85	84.09	904.00	1198.50
AST	AS_YNL228W	-0.232392	0.4251621	0.5802321	11.89	10.13	112.25	135.50
ORF-T	YBR137W	-0.232265	0.4399351	0.5933881	45.86	39.05	414.25	488.75
ORF-T	YFR005C	-0.231938	0.3318235	0.4961234	36.06	30.72	332.25	397.75
ORF-T	YMR284W	-0.231743	0.4205246	0.5761351	35.47	30.24	323.25	379.25
ORF-T	YDR172W	-0.231606	0.3670395	0.5295788	472.11	402.13	4424.50	5226.25
ORF-T	YBR076W	-0.231319	0.6946159	0.7931726	1.03	0.85	9.50	12.50
ORF-T	YAL001C	-0.231175	0.3639835	0.5278127	60.66	51.68	541.50	656.75
ORF-T	YBR169C	-0.231042	0.4691831	0.6176556	49.36	42.04	434.00	529.50
ORF-T	YBR081C	-0.231021	0.3931875	0.554351	68.03	57.94	623.25	768.75
ORF-T	YDR158W	-0.230843	0.4286753	0.5830539	458.71	390.86	4214.75	5231.50
ORF-T	YKL201C	-0.230828	0.4531183	0.6040047	15.82	13.41	136.50	177.75

TABLE S1: Differential expression data for RRP6 RNA-Seq dataset Page 133

Class	Transcript name	RRP6 KO_vs_WT log2_fold _change	RRP6 KO_vs_WT p-value	RRP6 KO_vs_WT FDR	Ave Norm Reads in WT	Ave Norm Reads in RRP6	Average RAW read counts in WT	Average RAW read counts in RRP6
ORF-T	YLR099W-A	-0.230763	0.4702594	0.6188302	6.29	5.34	56.25	70.25
ORF-T	YFL001W	-0.230712	0.3419847	0.506696	43.94	37.47	416.50	501.00
ORF-T	YMR071C	-0.230346	0.4517696	0.6030051	26.19	22.27	227.00	288.25
ORF-T	YDR404C	-0.230185	0.4401357	0.5933881	12.55	10.66	111.75	140.50
ORF-T	YDR311W	-0.230107	0.3219055	0.4862336	75.57	64.44	706.75	849.50
AST	AS_YFL031C-A	-0.229932	0.378911	0.540263	222.95	190.19	2141.75	2447.75
ORF-T	YPL049C	-0.229788	0.4127395	0.5699314	20.00	17.01	180.25	227.50
ORF-T	YBL038W	-0.229592	0.3760036	0.5378185	30.80	26.27	280.00	339.50
AST	AS_YOL079W	-0.229571	0.4772522	0.623961	5.37	4.59	50.25	61.00
sn/snoRNA	SNR76	-0.229566	0.418324	0.5743891	2517.09	2146.78	22464.50	28119.00
ORF-T	YOR265W	-0.22937	0.513628	0.6537746	13.18	11.21	120.75	152.50
ORF-T	YLR271W	-0.229331	0.4417146	0.5946269	10.21	8.66	88.75	112.25
ORF-T	YIL136W	-0.229276	0.5760628	0.7026616	44.73	38.16	381.00	455.25
ORF-T	YJR040W	-0.229273	0.3537059	0.5182	55.98	47.77	518.75	620.25
ORF-T	YDR395W	-0.228906	0.362065	0.5259382	184.42	157.39	1721.75	2039.00
ORF-T	YBL046W	-0.228684	0.3943524	0.5554756	24.15	20.58	217.00	272.25
AST	AS_YGR212W	-0.228634	0.6504426	0.7604714	0.91	0.77	8.25	10.25
ORF-T	YCR006C	-0.228605	0.425062	0.580174	15.09	12.88	135.50	164.25
ORF-T	YJL008C	-0.228512	0.3753207	0.5371464	437.37	373.33	4014.25	4782.25
ORF-T	YOR106W	-0.228493	0.3752221	0.5370815	16.05	13.67	148.25	183.25
ORF-T	YDR369C	-0.228316	0.3562394	0.5200781	29.32	25.06	273.25	325.25
ORF-T	YPR104C	-0.228216	0.351162	0.5157469	84.89	72.51	811.25	962.50
ORF-T	YML103C	-0.228172	0.3269511	0.491499	97.25	82.98	871.50	1094.00
ORF-T	YNL027W	-0.228133	0.3671198	0.5296189	32.61	27.80	298.00	372.50
ORF-T	YOR258W	-0.228122	0.411709	0.5688783	8.07	6.87	72.00	88.75
ORF-T	YCR042C	-0.228069	0.3562772	0.5200781	84.86	72.42	772.00	957.75
ORF-T	YCL005W	-0.227913	0.3898572	0.550605	18.41	15.66	163.50	207.75
ORF-T	YBL083C	-0.227896	0.4390081	0.5924842	14.57	12.42	134.75	165.75
ORF-T	YDR344C	-0.227875	0.5934544	0.7175533	2.34	1.99	22.75	28.50

Class	Transcript name	RRP6 KO_vs_WT log2_fold _change	RRP6 KO_vs_WT p-value	RRP6 KO_vs_WT FDR	Ave Norm Reads in WT	Ave Norm Reads in RRP6	Average RAW read counts in WT	Average RAW read counts in RRP6
ORF-T	YFL048C	-0.227802	0.389689	0.5504446	208.92	178.43	1879.25	2229.50
AST	AS_YJL009W	-0.227638	0.3427761	0.5073469	95.30	81.40	866.75	1042.25
ORF-T	YLR415C	-0.227632	0.463064	0.6125591	274.46	234.46	2593.00	3000.00
ORF-T	YBR153W	-0.227586	0.3743531	0.5362183	20.40	17.47	191.50	225.50
AST	AS_YJL075C	-0.227556	0.4760265	0.6233283	6.53	5.52	56.25	72.00
ORF-T	YML104C	-0.227482	0.3297918	0.4942956	58.51	49.95	535.25	662.25
ORF-T	YNL118C	-0.227433	0.3532034	0.5178236	93.80	80.07	842.75	1057.25
ORF-T	YOR115C	-0.227411	0.330309	0.4948501	60.41	51.58	552.50	682.25
ORF-T	YOR317W	-0.227327	0.5221106	0.6604319	338.89	289.49	3087.00	3671.00
ORF-T	YBR260C	-0.227177	0.36307	0.5266396	49.36	42.13	454.50	565.75
ORF-T	YHR063C	-0.226928	0.3327251	0.4970237	104.09	88.99	969.50	1144.50
ORF-T	YKL064W	-0.226713	0.3830616	0.5442854	80.01	68.37	714.75	872.00
ORF-T	YKR071C	-0.226472	0.3673496	0.5297989	32.74	27.93	289.75	364.50
ORF-T	YNR065C	-0.226141	0.4473539	0.5993646	23.75	20.26	223.75	286.75
ORF-T	YBR171W	-0.226059	0.4036972	0.5625874	14.33	12.23	131.50	163.25
ORF-T	YOL045W	-0.22603	0.3305126	0.4949346	68.59	58.65	643.00	782.50
ORF-T	YOL098C	-0.225797	0.3825104	0.5438086	346.35	296.18	3233.50	3920.50
ORF-T	YER033C	-0.225604	0.5354753	0.671358	9.10	7.73	77.00	99.50
AST	AS_YOL134C	-0.225562	0.4182971	0.5743891	16.77	14.30	152.50	191.75
ORF-T	YNR051C	-0.225526	0.3455667	0.5104284	70.51	60.29	644.25	787.50
ORF-T	YLR455W	-0.225477	0.3720985	0.5343551	24.89	21.30	225.00	271.50
AST	AS_YLR317W	-0.225416	0.4505866	0.601859	5.28	4.50	47.25	58.25
ORF-T	YML080W	-0.225267	0.4077387	0.566026	24.31	20.83	232.00	276.50
ORF-T	YLR253W	-0.225239	0.3624323	0.5262443	35.52	30.41	333.75	402.00
ORF-T	YHR120W	-0.225013	0.4015195	0.5608572	33.58	28.78	324.00	383.50
ORF-T	YDL042C	-0.224914	0.418404	0.5743891	12.06	10.30	110.25	136.75
ORF-T	YJL005W	-0.224902	0.4483856	0.5999833	121.27	103.70	1045.50	1344.00
NUT	NUT0554	-0.224851	0.3718619	0.5343196	47.42	40.56	422.50	520.25
ORF-T	YJL030W	-0.224738	0.4137163	0.5703684	18.25	15.63	169.75	203.25

TABLE S1: Differential expression data for RRP6 RNA-Seq dataset Page 135

Class	Transcript name	RRP6 KO_vs_WT log2_fold _change	RRP6 KO_vs_WT p-value	RRP6 KO_vs_WT FDR	Ave Norm Reads in WT	Ave Norm Reads in RRP6	Average RAW read counts in WT	Average RAW read counts in RRP6
ORF-T	YJR025C	-0.224564	0.5191901	0.6581412	16.25	13.85	137.25	177.25
ORF-T	YDR157W	-0.224558	0.4449988	0.5972009	422.63	361.68	3886.00	4841.75
ORF-T	YBR087W	-0.224335	0.3520373	0.5168066	61.03	52.23	559.25	687.00
ORF-T	YOL003C	-0.224034	0.4474332	0.5993646	19.21	16.43	170.00	210.00
ORF-T	YLR362W	-0.223997	0.4041592	0.5630969	51.33	43.96	476.75	571.75
ORF-T	YGL082W	-0.223837	0.4467619	0.5989294	41.32	35.37	367.50	452.00
AST	AS_YOR071C	-0.223825	0.5676658	0.6962225	2.72	2.34	26.75	32.25
ORF-T	YPL095C	-0.223745	0.3974098	0.5583004	28.55	24.45	261.50	317.25
ORF-T	YMR208W	-0.223659	0.3532921	0.5178236	57.92	49.60	541.75	660.75
ORF-T	YGR009C	-0.22344	0.3768025	0.5384266	28.99	24.82	265.50	324.00
ORF-T	YIL158W	-0.223408	0.6475932	0.7586349	28.51	24.38	258.00	352.75
ORF-T	YER092W	-0.223213	0.3789449	0.540263	33.35	28.59	298.75	361.00
ORF-T	YJL103C	-0.223198	0.6171597	0.7351727	9.38	8.02	80.00	100.00
sn/snoRNA ET	SNR54-ET	-0.222398	0.3807398	0.5419792	165.86	142.18	1544.00	1862.75
ORF-T	YKR054C	-0.222112	0.4350708	0.5891438	159.27	136.51	1469.25	1857.00
ORF-T	YAL027W	-0.221887	0.4019464	0.5608572	19.02	16.31	174.75	211.75
ORF-T	YOR346W	-0.221836	0.3975438	0.5583105	141.69	121.51	1342.50	1625.25
ORF-T	YKR089C	-0.22175	0.4248563	0.5799717	66.08	56.66	598.00	737.75
ORF-T	YNL147W	-0.221671	0.4770953	0.6239139	9.25	7.93	83.00	101.50
ORF-T	YMR115W	-0.221628	0.3641541	0.527817	53.01	45.49	485.75	581.25
ORF-T	YGL043W	-0.221564	0.3984661	0.5591643	46.29	39.69	427.00	525.25
ORF-T	YDL145C	-0.221416	0.4242168	0.5793339	506.20	434.20	4726.50	5667.25
AST	AS_YJR140W-A	-0.221225	0.4803833	0.6264303	4.90	4.21	45.00	54.75
ORF-T	YGR178C	-0.221211	0.3939033	0.5550648	330.51	283.56	3120.00	3739.25
AST	AS_YDR526C	-0.22109	0.4645267	0.6139302	18.07	15.52	174.00	210.50
ORF-T	YDL134C	-0.221078	0.3909594	0.5519298	181.48	155.73	1673.00	1983.50
ORF-T	YKL056C	-0.221059	0.4285369	0.5829443	2469.93	2119.07	22597.50	26941.25
ORF-T	YFR038W	-0.220898	0.3465581	0.5115181	77.03	66.14	729.75	869.00
ORF-T	YDR214W	-0.220834	0.4951594	0.6382701	39.61	33.93	353.25	458.75

Class	Transcript name	RRP6 KO_vs_WT log2_fold_change	RRP6 KO_vs_WT p-value	RRP6 KO_vs_WT FDR	Ave Norm Reads in WT	Ave Norm Reads in RRP6	Average RAW read counts in WT	Average RAW read counts in RRP6
ORF-T	YKL069W	-0.220503	0.4544397	0.6051194	24.85	21.28	220.00	282.25
ORF-T	YMR309C	-0.220356	0.3813054	0.5427076	497.40	426.94	4537.25	5561.75
ORF-T	YPR163C	-0.220293	0.4109683	0.5682441	479.11	411.34	4482.75	5146.00
ORF-T	YER036C	-0.220227	0.3996788	0.5599278	450.98	387.16	4181.25	5049.25
ORF-T	YLL056C	-0.220152	0.4868629	0.6321007	10.64	9.10	93.75	118.75
ORF-T	YOR337W	-0.220144	0.4910387	0.6348732	19.48	16.68	184.75	236.75
ORF-T	YNL186W	-0.219919	0.4001814	0.5600189	16.46	14.12	148.00	183.50
AST	AS_YPL136W	-0.219909	0.4444581	0.5967038	16.55	14.16	149.25	190.25
ORF-T	YKL119C	-0.219861	0.4404874	0.5934368	21.45	18.37	192.50	243.50
AST	AS_YDR396W	-0.219646	0.3991599	0.5595387	18.55	15.86	163.50	210.75
SUT	SUT606	-0.219514	0.519771	0.6586412	3.20	2.70	28.00	36.00
ORF-T	YML064C	-0.219363	0.4854587	0.6309274	29.99	25.77	283.50	343.50
ORF-T	YJR035W	-0.219266	0.4443581	0.5967038	19.27	16.53	175.00	218.75
ORF-T	YGL120C	-0.219234	0.3743293	0.5362183	83.24	71.48	762.75	955.00
sn/snoRNA	SNR24	-0.219193	0.5038838	0.6459655	52.13	44.74	435.25	562.25
ORF-T	YMR168C	-0.219089	0.3993451	0.5596158	25.85	22.14	234.00	301.50
ORF-T	YCR019W	-0.219041	0.4280802	0.5829213	11.61	9.97	104.50	128.75
ORF-T	YHR199C-A	-0.218982	0.4999136	0.6422638	4.70	4.01	41.50	52.50
ORF-T	YIL097W	-0.218926	0.5840094	0.7094508	7.85	6.69	65.25	86.25
ORF-T	YDR179W-A	-0.21887	0.4333591	0.5879549	24.31	20.96	235.75	275.25
ORF-T	YNR032C-A	-0.218855	0.4623724	0.6119415	16.76	14.35	144.50	183.75
ORF-T	YOR245C	-0.218829	0.3528549	0.5176236	35.27	30.31	321.25	392.50
ORF-T	YGL003C	-0.218441	0.3675084	0.52989	33.29	28.61	301.00	370.25
NUT	NUT0852	-0.218428	0.5051988	0.6470755	8.32	7.22	83.25	95.00
ORF-T	YLR117C	-0.218412	0.4327681	0.5873394	16.88	14.45	151.75	197.00
AST	AS_YPR087W	-0.218354	0.363056	0.5266396	43.85	37.74	407.50	485.50
ORF-T	YJR110W	-0.218349	0.4259789	0.5811109	21.58	18.57	202.25	243.00
ORF-T	YLR429W	-0.218238	0.4392199	0.5926795	209.77	180.37	1944.75	2272.50
ORF-T	YIR029W	-0.21823	0.5771342	0.7035313	3.73	3.18	34.00	43.25

Class	Transcript name	RRP6 KO_vs_WT log2_fold _change	RRP6 KO_vs_WT p-value	RRP6 KO_vs_WT FDR	Ave Norm Reads in WT	Ave Norm Reads in RRP6	Average RAW read counts in WT	Average RAW read counts in RRP6
ORF-T	YBR047W	-0.218022	0.6663962	0.7725844	1.58	1.34	13.50	17.50
ORF-T	YGL113W	-0.218022	0.4223388	0.5777081	11.77	10.09	108.75	135.75
ORF-T	YPL045W	-0.217972	0.3651915	0.5281972	43.58	37.47	401.75	489.75
ORF-T	YBL029W	-0.217971	0.5397964	0.6734265	3.74	3.21	33.50	41.25
ORF-T	YLR250W	-0.217794	0.3431105	0.5075438	91.17	78.41	834.75	1019.00
AST	AS_YMR245W	-0.217773	0.4166297	0.5731643	13.97	12.04	130.25	156.75
ORF-T	YNL153C	-0.217638	0.3822792	0.5436089	37.99	32.66	345.00	423.00
ORF-T	YOR007C	-0.217324	0.4175732	0.5743055	466.41	401.18	4147.25	5107.25
ORF-T	YDR322W	-0.21712	0.4843857	0.6299389	9.19	7.87	82.25	104.25
ORF-T	YDR358W	-0.217046	0.6443148	0.7564642	30.50	26.23	266.25	330.00
ORF-T	YOR371C	-0.217006	0.4024863	0.5613654	50.59	43.52	475.75	588.00
AST	AS_YDR124W	-0.216916	0.4998268	0.6422341	3.85	3.27	34.75	44.00
ORF-T	YOR281C	-0.216886	0.3888799	0.5496285	25.05	21.57	232.75	279.75
ORF-T	YJR100C	-0.216763	0.3694064	0.5320802	31.72	27.30	288.50	351.25
ORF-T	YOR270C	-0.216238	0.4158394	0.5724675	607.52	522.95	5637.50	6948.50
ORF-T	YOR165W	-0.216202	0.3653696	0.5283644	61.67	53.07	558.25	686.75
ORF-T	YAL036C	-0.216072	0.3714537	0.5341137	102.61	88.38	955.75	1141.50
ORF-T	YJL127C-B	-0.215989	0.3873732	0.5481717	36.31	31.30	326.25	391.25
ORF-T	YNL180C	-0.21588	0.5270085	0.6644251	8.84	7.53	75.25	101.25
NUT	NUT0422	-0.215794	0.3614767	0.5256137	93.43	80.47	861.75	1042.25
ORF-T	YOL157C	-0.215654	0.538232	0.672581	3.44	3.03	34.25	39.25
ORF-T	YJR103W	-0.215473	0.3622055	0.5260665	93.43	80.49	861.75	1042.50
ORF-T	YML095C	-0.215294	0.5418883	0.6750342	3.30	2.80	29.25	37.50
ORF-T	YNL227C	-0.215236	0.417567	0.5743055	21.44	18.46	200.50	247.25
ORF-T	YBL015W	-0.215174	0.5560146	0.6866093	86.50	74.52	751.00	910.25
SUT	SUT765	-0.215045	0.5502665	0.6816816	4.44	3.84	44.25	53.75
ORF-T	YOR295W	-0.215031	0.458853	0.6088332	7.45	6.44	71.00	85.50
ORF-T	YHR045W	-0.215026	0.40555	0.5640781	121.70	104.88	1107.75	1326.75
ORF-T	YMR005W	-0.214862	0.3646236	0.5279324	33.15	28.59	311.75	375.25

Class	Transcript name	RRP6 KO_vs_WT log2_fold _change	RRP6 KO_vs_WT p-value	RRP6 KO_vs_WT FDR	Ave Norm Reads in WT	Ave Norm Reads in RRP6	Average RAW read counts in WT	Average RAW read counts in RRP6
ORF-T	YPL159C	-0.214669	0.4118379	0.5689784	21.92	18.82	197.00	254.00
ORF-T	YER125W	-0.214192	0.454897	0.6053288	366.27	315.71	3391.75	4246.50
ORF-T	YDR148C	-0.214055	0.5877705	0.7125605	90.97	78.40	766.25	970.50
AST	AS_YBL021C	-0.213895	0.6189422	0.7364499	3.07	2.64	28.50	36.00
ORF-T	YPL233W	-0.213842	0.4227633	0.5780536	14.95	12.90	137.25	168.00
ORF-T	YJL085W	-0.213823	0.4130644	0.5699701	21.84	18.82	195.25	241.50
ORF-T	YPL212C	-0.213796	0.4014437	0.5608572	109.43	94.36	1021.25	1262.50
ORF-T	YOL031C	-0.213343	0.3789214	0.540263	29.19	25.16	263.75	327.50
ORF-T	YML016C	-0.213279	0.4018603	0.5608572	43.61	37.60	388.00	483.25
ORF-T	YHR186C	-0.213108	0.3665377	0.5289304	43.48	37.44	388.50	499.50
ORF-T	YIL106W	-0.213049	0.3845694	0.5458232	58.39	50.36	546.50	686.25
ORF-T	YNL083W	-0.213019	0.4031409	0.562039	24.33	21.03	227.25	272.50
ORF-T	YOL056W	-0.212981	0.4351657	0.5891798	18.06	15.55	163.00	204.00
ORF-T	YKR018C	-0.212964	0.5136708	0.6537746	122.45	105.63	1097.75	1367.00
ORF-T	YMR090W	-0.212804	0.4894153	0.633784	10.55	9.10	94.50	116.25
ORF-T	YJR155W	-0.212748	0.4854171	0.6309274	20.00	17.30	193.75	230.25
ORF-T	YIL046W	-0.212598	0.4066027	0.5649104	113.63	98.11	1074.00	1270.75
ORF-T	YGL139W	-0.212557	0.3462399	0.5111981	70.03	60.40	629.75	793.25
ORF-T	YOR376W-A	-0.21253	0.5140863	0.6541383	14.98	12.92	129.75	161.50
ORF-T	YJL069C	-0.212356	0.4707066	0.6191722	66.55	57.44	639.75	793.25
ORF-T	YDL132W	-0.21183	0.5145416	0.6543874	90.14	77.82	821.25	1023.25
ORF-T	YBL024W	-0.211817	0.4113634	0.5686345	130.47	112.65	1201.25	1491.00
ORF-T	YGR099W	-0.211788	0.3846428	0.5458232	32.67	28.20	294.75	367.25
ORF-T	YOR086C	-0.211653	0.5366909	0.6716583	312.64	269.96	2830.50	3528.50
NUT	NUT0887	-0.211643	0.4347696	0.5891083	25.47	21.99	235.50	289.25
ORF-T	YDR520C	-0.211495	0.400188	0.5600189	23.41	20.19	213.50	269.00
ORF-T	YHR037W	-0.211449	0.4861995	0.6315646	59.45	51.30	534.50	687.25
ORF-T	YBR164C	-0.211404	0.3400082	0.5045333	72.46	62.60	667.25	817.75
ORF-T	YDR439W	-0.211319	0.4722196	0.6203033	9.65	8.30	89.50	113.50

Class	Transcript name	RRP6 KO_vs_WT log2_fold _change	RRP6 KO_vs_WT p-value	RRP6 KO_vs_WT FDR	Ave Norm Reads in WT	Ave Norm Reads in RRP6	Average RAW read counts in WT	Average RAW read counts in RRP6
AST	AS_YML066C	-0.211295	0.527271	0.6645421	5.25	4.50	47.50	60.75
ORF-T	YOL006C	-0.211288	0.3838672	0.5451399	62.86	54.32	579.50	705.25
SUT	SUT217	-0.211086	0.5201841	0.6586702	3.99	3.40	35.75	45.75
sn/snoRNA ET	SNR51-ET	-0.21095	0.4676589	0.6163733	41.71	35.98	361.75	464.75
ORF-T	YOL052C	-0.210893	0.4883809	0.6331748	12.48	10.73	109.50	142.25
ORF-T	YGR162W	-0.210749	0.4045073	0.5631716	244.44	211.26	2299.25	2746.50
ORF-T	YPL145C	-0.210696	0.4771125	0.6239139	120.40	104.01	1093.75	1377.25
ORF-T	YDR498C	-0.210674	0.4328162	0.5873394	24.07	20.79	220.75	274.25
ORF-T	YBR229C	-0.210485	0.5118656	0.6523004	153.54	132.68	1407.00	1762.25
ORF-T	YKR023W	-0.210366	0.4420215	0.5948678	15.26	13.19	141.50	174.25
ORF-T	YGR090W	-0.210339	0.4017883	0.5608572	129.46	111.91	1200.25	1468.75
AST	AS_YDR327W	-0.210229	0.355415	0.5195827	76.72	66.35	710.25	854.50
ORF-T	YLR421C	-0.210061	0.3648221	0.5279658	59.48	51.38	534.50	670.50
ORF-T	YLR262C	-0.209915	0.3554804	0.5195827	42.54	36.79	389.25	474.25
ORF-T	YOR048C	-0.209742	0.3735545	0.5357034	116.07	100.37	1087.25	1338.75
ORF-T	YPL058C	-0.209525	0.4674545	0.616265	46.58	40.22	412.75	533.75
ORF-T	YDL212W	-0.209488	0.4276382	0.5827292	188.11	162.73	1728.75	2069.00
ORF-T	YER025W	-0.209013	0.4003641	0.5601875	239.37	207.10	2212.25	2682.25
ORF-T	YIL111W	-0.208947	0.5172268	0.6563426	33.57	29.02	287.00	362.50
ORF-T	YGR165W	-0.208855	0.3887276	0.5496255	66.18	57.30	607.25	726.25
ORF-T	YER093C	-0.208772	0.3559577	0.5199127	73.21	63.33	671.00	835.25
ORF-T	YJL207C	-0.208695	0.3719578	0.5343551	117.69	101.83	1081.25	1344.25
ORF-T	YJL171C	-0.20852	0.4633362	0.6128388	493.68	427.29	4587.00	5426.00
ORF-T	YBR170C	-0.208485	0.4299198	0.5844313	31.09	26.92	286.50	352.00
ORF-T	YKR036C	-0.20836	0.3985588	0.559197	42.48	36.81	401.50	484.25
ORF-T	YOL144W	-0.208351	0.4494014	0.6008702	29.65	25.68	286.50	349.75
ORF-T	YFL009W	-0.208204	0.3722573	0.5344309	64.81	56.09	596.00	742.25
ORF-T	YGL058W	-0.208152	0.3829356	0.5442598	66.85	57.88	618.00	755.75
ORF-T	YKL197C	-0.207984	0.5032535	0.6455678	39.47	34.16	366.50	457.00

Class	Transcript name	RRP6 KO_vs_WT log2_fold _change	RRP6 KO_vs_WT p-value	RRP6 KO_vs_WT FDR	Ave Norm Reads in WT	Ave Norm Reads in RRP6	Average RAW read counts in WT	Average RAW read counts in RRP6
ORF-T	YDR292C	-0.207895	0.4199771	0.5755707	137.15	118.74	1265.25	1564.25
ORF-T	YDR081C	-0.207878	0.3748588	0.5367139	48.49	41.99	456.75	561.25
ORF-T	YBR091C	-0.207847	0.4805533	0.6265709	9.86	8.52	88.00	109.50
ORF-T	YLR016C	-0.207814	0.4099968	0.5675234	22.75	19.67	204.75	258.50
ORF-T	YDR411C	-0.20776	0.5008791	0.6432584	36.56	31.63	328.25	417.00
ORF-T	YKL095W	-0.207739	0.5035944	0.6458725	5.72	4.91	51.25	66.00
ORF-T	YGL162W	-0.2075	0.5618471	0.6913564	11.76	10.19	100.75	125.50
ORF-T	YDL235C	-0.20748	0.3737625	0.5357764	58.37	50.60	533.75	643.25
ORF-T	YOR119C	-0.207269	0.3988565	0.5593977	47.25	40.93	442.75	547.75
ORF-T	YOL029C	-0.207014	0.5498645	0.6813509	5.68	4.94	55.00	67.25
ORF-T	YDR425W	-0.206869	0.6783203	0.7804863	2.46	2.08	19.75	27.50
ORF-T	YPL196W	-0.20682	0.5318559	0.6684825	29.10	25.21	264.25	322.25
ORF-T	YDL217C	-0.20678	0.4294401	0.5840153	21.71	18.81	203.25	251.75
ORF-T	YJL129C	-0.20673	0.3657343	0.5285144	64.56	55.93	579.00	719.25
ORF-T	YPR110C	-0.206699	0.4705119	0.6190011	296.94	257.35	2750.00	3271.25
ORF-T	YPL173W	-0.206553	0.4985441	0.6412529	17.75	15.38	159.75	196.50
ORF-T	YLR114C	-0.206515	0.4135878	0.5703022	122.95	106.58	1155.25	1400.75
AST	AS_YFL034C-A	-0.20643	0.6949082	0.7933266	1.21	1.03	10.50	13.75
ORF-T	YBL017C	-0.206396	0.4402112	0.5933881	259.38	224.80	2378.75	2949.75
ORF-T	YKR019C	-0.206388	0.4233588	0.5787894	20.30	17.60	190.50	235.00
ORF-T	YGR141W	-0.206297	0.4923704	0.6361388	26.68	23.14	239.50	289.25
ORF-T	YEL059C-A	-0.206211	0.6084993	0.7286699	2.33	2.00	21.75	27.50
ORF-T	YMR119W-A	-0.206151	0.5068678	0.6484718	5.51	4.72	48.00	61.75
SUT	SUT843	-0.206134	0.6539441	0.7634142	1.22	1.06	11.00	13.75
AST	AS_YML002W	-0.206093	0.6676229	0.7732146	1.93	1.63	17.00	23.00
AST	AS_YKL111C	-0.205806	0.4719252	0.6201321	12.80	11.09	119.00	148.75
ORF-T	YDL002C	-0.205769	0.384648	0.5458232	30.04	26.05	274.50	338.00
ORF-T	YGR175C	-0.205735	0.4439888	0.5963216	608.63	527.79	5725.50	6808.50
ORF-T	YDR460W	-0.205729	0.3770274	0.5385191	42.39	36.78	396.25	486.25

Class	Transcript name	RRP6 KO_vs_WT log2_fold _change	RRP6 KO_vs_WT p-value	RRP6 KO_vs_WT FDR	Ave Norm Reads in WT	Ave Norm Reads in RRP6	Average RAW read counts in WT	Average RAW read counts in RRP6
ORF-T	YNR029C	-0.205485	0.3801904	0.5416558	57.83	50.19	542.75	655.50
ORF-T	YJR069C	-0.2053	0.4944101	0.6377769	11.44	9.87	103.25	132.75
ORF-T	YBR143C	-0.205264	0.4202721	0.5758967	381.56	330.98	3537.75	4293.75
ORF-T	YBR005W	-0.205244	0.4738236	0.6212702	91.34	79.28	854.75	1021.00
ORF-T	YNL254C	-0.205198	0.4671132	0.6161372	31.63	27.48	303.75	367.75
ORF-T	YMR067C	-0.205032	0.4753945	0.6226625	14.51	12.52	125.25	163.50
ORF-T	YMR064W	-0.204964	0.3850103	0.5460887	38.56	33.40	348.25	443.75
ORF-T	YLR035C	-0.204799	0.4364403	0.5901132	16.75	14.50	154.25	195.25
AST	AS_YPL200W	-0.204787	0.6749571	0.7782149	1.25	1.07	11.00	14.00
AST	AS_YER046W-A	-0.20449	0.5257612	0.6635653	4.39	3.81	40.00	49.50
ORF-T	YPL100W	-0.204287	0.539538	0.6733344	7.70	6.62	66.50	88.00
ORF-T	YKR066C	-0.204209	0.5235055	0.6617813	81.06	70.36	740.75	912.75
ORF-T	YDR331W	-0.204172	0.423801	0.5790014	48.89	42.43	440.50	546.75
ORF-T	YPL195W	-0.204138	0.4846373	0.6301847	146.57	127.25	1376.50	1658.00
ORF-T	YDR027C	-0.204007	0.4183101	0.5743891	69.40	60.26	631.25	774.00
ORF-T	YCR077C	-0.203885	0.4910761	0.6348732	39.20	33.97	348.75	459.50
ORF-T	YPR127W	-0.203702	0.4372908	0.5907799	30.37	26.31	276.75	357.50
ORF-T	YGR183C	-0.203646	0.5438517	0.676464	59.02	51.23	506.00	633.50
ORF-T	YDL077C	-0.203602	0.5361776	0.6715989	13.66	11.83	124.50	158.50
ORF-T	YIL064W	-0.203523	0.3888837	0.5496285	51.30	44.64	479.00	562.75
ORF-T	YGR040W	-0.203504	0.4688883	0.6174378	40.93	35.56	399.50	495.00
ORF-T	YJR089W	-0.203458	0.3938796	0.5550648	54.98	47.82	520.75	619.25
ORF-T	YPL040C	-0.203373	0.4213212	0.5766291	27.71	24.02	251.25	320.25
ORF-T	YNL323W	-0.20336	0.3657196	0.5285144	73.25	63.60	659.75	822.75
ORF-T	YNL026W	-0.203319	0.4656616	0.6149465	53.37	46.37	504.50	612.75
ORF-T	YJL049W	-0.203313	0.5537658	0.6845868	7.39	6.39	66.00	84.00
ORF-T	YMR251W-A	-0.203308	0.5607772	0.6905969	212.55	184.61	1840.25	2282.50
ORF-T	YHR134W	-0.203082	0.4743097	0.6217261	11.51	10.01	104.75	129.00
ORF-T	YGR119C	-0.203069	0.4083589	0.5663587	161.78	140.60	1536.75	1825.00

Class	Transcript name	RRP6 KO_vs_WT log2_fold _change	RRP6 KO_vs_WT p-value	RRP6 KO_vs_WT FDR	Ave Norm Reads in WT	Ave Norm Reads in RRP6	Average RAW read counts in WT	Average RAW read counts in RRP6
ORF-T	YLR376C	-0.202823	0.5468897	0.679167	5.64	4.90	51.75	65.00
ORF-T	YMR033W	-0.20277	0.4192348	0.5750223	112.82	98.02	1038.00	1288.50
ORF-T	YMR212C	-0.202379	0.462747	0.61222	264.64	230.02	2462.75	2998.00
ORF-T	YER099C	-0.202254	0.3830135	0.5442854	53.04	46.15	493.50	595.75
ORF-T	YKL211C	-0.202239	0.4462495	0.5985134	168.74	146.67	1577.50	1952.25
ORF-T	YLR319C	-0.202161	0.40545	0.5640168	49.76	43.31	474.50	566.75
ORF-T	YGR196C	-0.202152	0.5077314	0.6492472	11.04	9.60	100.75	124.00
ORF-T	YJL204C	-0.202085	0.4876423	0.6327052	13.10	11.36	115.50	146.75
ORF-T	YDR304C	-0.201896	0.4948974	0.6380354	172.12	149.65	1576.50	1914.25
ORF-T	YBR044C	-0.201797	0.5025715	0.644857	22.45	19.48	202.50	259.25
ORF-T	YNL046W	-0.201595	0.5385237	0.6725877	18.91	16.40	172.50	224.25
ORF-T	YNL061W	-0.201312	0.458361	0.6084929	317.77	276.46	2994.25	3492.50
ORF-T	YPL029W	-0.201251	0.4235924	0.5789542	32.73	28.48	308.50	377.50
ORF-T	YJL093C	-0.201251	0.3945736	0.5554817	94.38	82.13	883.00	1075.25
ORF-T	YOL077C	-0.201243	0.3758679	0.5377007	71.50	62.20	658.25	814.25
AST	AS_YOR282W	-0.201166	0.5102282	0.6512666	4.77	4.15	44.00	54.00
ORF-T	YNL056W	-0.20108	0.4093074	0.5670908	31.01	26.96	279.25	349.50
NUT	NUT0638	-0.20102	0.4828702	0.6286165	21.98	19.12	196.50	243.25
ORF-T	YNL051W	-0.200973	0.532435	0.6688768	13.59	11.77	124.00	163.00
ORF-T	YDR294C	-0.200942	0.4422684	0.5949296	153.65	133.67	1393.50	1721.75
ORF-T	YKL130C	-0.200929	0.5076606	0.649239	8.69	7.51	77.00	100.25
ORF-T	YGL157W	-0.200611	0.4818459	0.6278504	23.81	20.68	215.50	273.25
ORF-T	YIL166C	-0.200558	0.5081531	0.6495294	8.06	7.03	76.00	93.50
ORF-T	YMR229C	-0.200386	0.4623697	0.6119415	357.35	311.07	3318.75	3938.75
ORF-T	YJR064W	-0.200339	0.4510668	0.6022984	671.13	584.18	6235.25	7379.25
ORF-T	YEL013W	-0.200284	0.4576887	0.6076805	162.91	141.80	1532.50	1883.75
ORF-T	YGR071C	-0.200192	0.4155913	0.5723518	26.09	22.69	237.25	298.50
ORF-T	YGR136W	-0.19985	0.3862282	0.5471671	53.61	46.60	481.00	623.25
ORF-T	YDR341C	-0.199769	0.4417245	0.5946269	690.58	601.32	6405.25	7744.50

Class	Transcript name	RRP6 KO_vs_WT log2_fold _change	RRP6 KO_vs_WT p-value	RRP6 KO_vs_WT FDR	Ave Norm Reads in WT	Ave Norm Reads in RRP6	Average RAW read counts in WT	Average RAW read counts in RRP6
ORF-T	YBL097W	-0.199491	0.4401808	0.5933881	30.27	26.34	274.50	348.00
ORF-T	YGL002W	-0.199464	0.4891484	0.6336784	38.95	33.96	357.00	425.00
ORF-T	YBL078C	-0.199393	0.5152482	0.6550638	18.49	16.13	165.75	200.75
ORF-T	YCL052C	-0.199177	0.4525661	0.6035811	61.58	53.64	563.00	695.75
ORF-T	YPR083W	-0.19917	0.470512	0.6190011	26.12	22.72	232.25	294.00
ORF-T	YDL108W	-0.199157	0.3945026	0.5554817	32.91	28.69	306.00	377.25
ORF-T	YDR314C	-0.199139	0.4484405	0.5999833	22.91	19.92	202.50	259.00
ORF-T	YLR246W	-0.19911	0.4868594	0.6321007	16.32	14.20	147.75	185.75
ORF-T	YDR427W	-0.198975	0.4313601	0.5859154	130.79	113.94	1182.25	1468.25
SRT	SRT210	-0.19889	0.604578	0.7261272	3.80	3.33	36.50	45.00
ORF-T	YGR140W	-0.198818	0.4675796	0.6163494	14.53	12.62	132.75	169.75
ORF-T	YER006W	-0.198734	0.4568743	0.6069988	168.39	146.78	1560.00	1857.75
ORF-T	YMR191W	-0.198717	0.4087496	0.5667312	108.75	94.80	1014.75	1228.75
ORF-T	YHR115C	-0.198602	0.4685209	0.6173481	16.59	14.41	150.75	194.00
ORF-T	YOL087C	-0.198518	0.473336	0.6209341	125.35	109.22	1125.75	1397.75
ORF-T	YNL108C	-0.198319	0.411472	0.5686639	33.94	29.56	312.00	393.00
ORF-T	YGL188C-A	-0.19828	0.6092014	0.7290291	5.79	5.07	52.75	64.00
ORF-T	YLR010C	-0.198276	0.5813744	0.7076137	10.81	9.39	95.00	124.75
NUT	NUT0661	-0.198239	0.5172506	0.6563426	9025.24	7866.52	79507.00	100021.75
ORF-T	YDL234C	-0.198235	0.5200108	0.6586702	210.39	183.42	1964.00	2351.25
sn/snoRNA	SNR37	-0.198167	0.5331928	0.6691938	4940.19	4306.14	43037.75	54843.25
ORF-T	YOL062C	-0.197991	0.3959669	0.5568163	119.77	104.44	1099.75	1344.00
ORF-T	YDR477W	-0.197904	0.3991694	0.5595387	62.46	54.42	560.50	710.50
SUT	SUT026	-0.197836	0.6357964	0.7498679	1.80	1.56	16.25	20.50
ORF-T	YOR378W	-0.197711	0.6348071	0.7491392	1.97	1.74	18.50	22.50
ORF-T	YPR108W	-0.197686	0.4870923	0.6322356	238.15	207.67	2179.25	2638.75
ORF-T	YDR188W	-0.197665	0.4660931	0.6152747	427.97	373.24	3979.75	4692.50
ORF-T	YOR336W	-0.197635	0.3986472	0.559197	116.05	101.18	1062.00	1342.25
ORF-T	YDR472W	-0.197606	0.510591	0.6513346	29.21	25.45	274.25	348.50

Class	Transcript name	RRP6 KO_vs_WT log2_fold _change	RRP6 KO_vs_WT p-value	RRP6 KO_vs_WT FDR	Ave Norm Reads in WT	Ave Norm Reads in RRP6	Average RAW read counts in WT	Average RAW read counts in RRP6
ORF-T	YDL088C	-0.197429	0.4403465	0.5934056	32.78	28.50	289.50	380.75
ORF-T	YMR224C	-0.197368	0.5242065	0.6625014	8.08	7.00	70.25	90.75
SRT	SRT56	-0.197305	0.631403	0.7465198	1.31	1.11	11.50	14.75
ORF-T	YKL073W	-0.197118	0.4390165	0.5924842	126.86	110.69	1158.50	1410.00
ORF-T	YDL168W	-0.197016	0.4066526	0.5649104	70.39	61.44	650.75	793.25
NUT	NUT1510	-0.196982	0.5163109	0.6558105	2829.17	2468.09	24956.50	31268.75
ORF-T	YLR405W	-0.196854	0.439353	0.5927798	37.95	33.14	363.75	443.50
ORF-T	YLR243W	-0.196731	0.4060545	0.5644683	41.64	36.34	387.00	479.00
ORF-T	YOL059W	-0.196512	0.4508559	0.6020965	39.61	34.48	339.75	448.00
ORF-T	YOL009C	-0.196438	0.5425952	0.675581	6.29	5.46	57.00	73.50
ORF-T	YJL050W	-0.19633	0.4661596	0.6152818	360.47	314.68	3438.50	4047.75
ORF-T	YDL012C	-0.196256	0.5406574	0.6740837	5.39	4.73	49.00	59.75
ORF-T	YPL116W	-0.196091	0.4063548	0.5647301	52.83	46.12	478.25	594.50
ORF-T	YEL003W	-0.195988	0.4264529	0.5816001	49.69	43.40	449.75	550.50
ORF-T	YKR026C	-0.195931	0.400922	0.5605521	86.94	75.94	815.50	991.00
ORF-T	YOR047C	-0.195815	0.4931151	0.6369377	17.38	15.15	160.50	201.75
ORF-T	YJL124C	-0.195757	0.4783189	0.6243842	29.52	25.72	262.50	340.75
ORF-T	YGR043C	-0.195702	0.6509209	0.7608539	21.90	19.13	188.50	230.00
ORF-T	YDL065C	-0.195667	0.4894779	0.633784	9.61	8.36	88.50	112.75
AST	AS_YCL048W	-0.195581	0.6934478	0.7923771	1.41	1.23	13.50	17.00
ORF-T	YDR415C	-0.195241	0.4166283	0.5731643	42.34	36.98	386.75	480.50
NUT	NUT1169	-0.195064	0.539445	0.6733214	4940.33	4315.54	43039.25	54973.25
ORF-T	YPL126W	-0.195055	0.4006084	0.5604517	109.59	95.76	1009.50	1239.25
ORF-T	YMR128W	-0.194974	0.3924409	0.5534685	44.33	38.76	411.50	501.25
ORF-T	YJL031C	-0.194617	0.4267981	0.5819135	42.29	36.94	382.00	479.25
ORF-T	YOL053W	-0.194463	0.5430945	0.6758689	28.95	25.32	265.50	319.75
SUT	SUT292	-0.194312	0.5619589	0.6914097	5.25	4.55	47.50	61.50
ORF-T	YNL133C	-0.194217	0.5548207	0.6854707	4.26	3.71	38.75	48.50
ORF-T	YJL131C	-0.194095	0.5586358	0.6890866	13.18	11.51	115.00	142.75

Class	Transcript name	RRP6 KO_vs_WT log2_fold _change	RRP6 KO_vs_WT p-value	RRP6 KO_vs_WT FDR	Ave Norm Reads in WT	Ave Norm Reads in RRP6	Average RAW read counts in WT	Average RAW read counts in RRP6
ORF-T	YMR310C	-0.194031	0.6186351	0.7362208	4.05	3.51	36.00	46.75
ORF-T	YMR167W	-0.193931	0.4505469	0.601859	31.41	27.42	290.50	373.50
ORF-T	YNL097C	-0.193796	0.4809721	0.6270359	29.06	25.45	273.25	325.75
ORF-T	YOR285W	-0.193581	0.4627353	0.61222	113.01	98.85	1010.50	1220.00
ORF-T	YJL186W	-0.19344	0.4903293	0.6343921	293.59	256.81	2697.75	3198.25
ORF-T	YLR006C	-0.193386	0.5172135	0.6563426	25.77	22.50	232.00	296.00
ORF-T	YHR166C	-0.193232	0.4438256	0.5963216	34.85	30.51	323.00	396.25
ORF-T	YHR034C	-0.193229	0.4595922	0.6094053	25.55	22.36	238.75	295.25
AST	AS_YJL156W-A	-0.193183	0.5667708	0.6956309	3.84	3.35	34.75	43.75
ORF-T	YML125C	-0.19295	0.4315182	0.5859723	59.06	51.67	525.75	652.25
ORF-T	YNL200C	-0.192793	0.6309309	0.7460491	28.61	25.03	246.25	304.50
ORF-T	YHR026W	-0.192735	0.4816433	0.6276675	303.81	265.80	2770.75	3499.75
ORF-T	YKR084C	-0.192592	0.466309	0.6153984	41.81	36.57	381.75	478.00
AST	AS_YBL066C	-0.192585	0.6338021	0.7484614	4.98	4.39	47.25	57.50
ORF-T	YGR072W	-0.192585	0.4839254	0.6295026	11.60	10.13	108.25	137.25
ORF-T	YLR054C	-0.192557	0.5951034	0.7190298	5.92	5.17	53.00	67.25
ORF-T	YDR065W	-0.192351	0.4541957	0.6050092	21.47	18.78	199.75	252.00
ORF-T	YJR001W	-0.19226	0.451954	0.6031635	199.94	175.04	1843.50	2223.00
ORF-T	YNL137C	-0.192214	0.4561289	0.6064078	82.34	72.06	739.75	925.25
ORF-T	YLR261C	-0.191804	0.4350807	0.5891438	25.93	22.73	240.25	294.50
ORF-T	YJL142C	-0.191684	0.5958608	0.7197256	11.27	9.87	98.50	121.25
ORF-T	YML042W	-0.191433	0.5585852	0.6890866	5.62	4.89	51.50	66.50
ORF-T	YJR060W	-0.191275	0.4095116	0.5670908	57.23	50.16	525.75	645.00
ORF-T	YCL031C	-0.191091	0.4113109	0.5686345	68.94	60.41	650.75	806.00
ORF-T	YML098W	-0.191017	0.4423162	0.5949296	21.09	18.48	192.50	240.00
ORF-T	YBR022W	-0.190966	0.5305603	0.6674364	11.64	10.20	105.50	129.00
ORF-T	YBR267W	-0.190591	0.5123988	0.6524851	47.72	41.81	448.25	563.00
ORF-T	YNR036C	-0.190559	0.4589192	0.6088332	41.98	36.84	388.75	466.25
ORF-T	YCR023C	-0.190559	0.4387965	0.5923459	39.17	34.26	351.00	455.25

Class	Transcript name	RRP6 KO_vs_WT log2_fold _change	RRP6 KO_vs_WT p-value	RRP6 KO_vs_WT FDR	Ave Norm Reads in WT	Ave Norm Reads in RRP6	Average RAW read counts in WT	Average RAW read counts in RRP6
ORF-T	YIL077C	-0.190536	0.4916973	0.6354146	38.98	34.16	347.00	433.50
ORF-T	YIL090W	-0.190386	0.4534326	0.6042572	48.19	42.19	445.50	579.50
ORF-T	YPL211W	-0.190384	0.4729972	0.6208801	143.77	126.03	1306.25	1597.75
ORF-T	YDR397C	-0.190349	0.467383	0.616265	19.63	17.13	173.50	227.75
ORF-T	YDR083W	-0.190316	0.4517754	0.6030051	62.75	55.03	578.75	710.00
ORF-T	YNL223W	-0.189942	0.5056274	0.6473777	22.03	19.31	197.25	245.75
ORF-T	YER013W	-0.189913	0.4688057	0.6174378	18.33	16.06	165.00	208.25
ORF-T	YPR201W	-0.189825	0.5672969	0.6958547	4.24	3.71	39.00	49.75
CUT	CUT015	-0.189511	0.485874	0.6312447	104.15	91.33	908.50	1140.25
ORF-T	YLR315W	-0.189221	0.7250529	0.8164639	0.72	0.62	6.75	8.50
ORF-T	YER001W	-0.189081	0.5329733	0.6691363	161.23	141.42	1442.25	1786.50
ORF-T	YDR394W	-0.189006	0.4866741	0.6320183	231.45	203.02	2123.75	2659.00
ORF-T	YER183C	-0.188927	0.4196007	0.5752894	36.71	32.20	335.50	421.75
ORF-T	YNL189W	-0.188865	0.4777858	0.6242376	135.94	119.25	1261.25	1580.25
ORF-T	YGL008C	-0.188785	0.5183545	0.6574125	3951.45	3466.73	36685.75	48007.25
ORF-T	YDR061W	-0.188653	0.4575508	0.6075773	68.66	60.28	632.75	770.25
SUT	SUT509	-0.18864	0.5268471	0.6644251	9.61	8.43	85.50	106.75
ORF-T	YCL046W	-0.188415	0.6431627	0.7559153	1.89	1.68	18.25	22.50
ORF-T	YDR014W	-0.1883	0.4847313	0.6302257	16.40	14.38	147.50	186.50
ORF-T	YLR394W	-0.188091	0.5920753	0.7164871	7.69	6.72	69.25	89.75
ORF-T	YFL054C	-0.187925	0.5834025	0.7091174	87.37	76.73	802.00	955.25
ORF-T	YMR255W	-0.187812	0.4768814	0.6237998	15.88	13.95	145.50	183.00
ORF-T	YIL014C-A	-0.187801	0.5902503	0.7146217	5.39	4.72	47.25	60.25
ORF-T	YCL034W	-0.187624	0.4653387	0.6148968	77.64	68.20	713.00	866.50
ORF-T	YNL123W	-0.18737	0.4520812	0.6032535	65.11	57.21	618.75	762.75
ORF-T	YAL044W-A	-0.187315	0.6494039	0.7598693	1.66	1.41	14.25	18.75
ORF-T	YLR039C	-0.187306	0.5290254	0.6661704	35.30	31.02	336.50	410.00
ORF-T	YDR329C	-0.187247	0.456293	0.6065148	43.49	38.19	396.00	497.50
ORF-T	YCR038C	-0.187081	0.6015676	0.7242368	4.38	3.79	36.75	49.25

Class	Transcript name	RRP6 KO_vs_WT log2_fold _change	RRP6 KO_vs_WT p-value	RRP6 KO_vs_WT FDR	Ave Norm Reads in WT	Ave Norm Reads in RRP6	Average RAW read counts in WT	Average RAW read counts in RRP6
ORF-T	YGL227W	-0.187	0.5490668	0.680697	57.13	50.20	509.25	623.50
ORF-T	YIL101C	-0.186887	0.7034657	0.7997933	8.65	7.60	73.25	90.75
NUT	NUT1140	-0.186887	0.7035072	0.7997933	8.65	7.60	73.25	90.75
ORF-T	YNL158W	-0.186758	0.4655858	0.6149465	16.97	14.87	155.50	201.25
ORF-T	YCR008W	-0.186644	0.4083691	0.5663587	100.12	87.97	914.00	1141.25
ORF-T	YNL006W	-0.186639	0.4832752	0.6289813	71.58	62.84	626.75	811.25
ORF-T	YJR101W	-0.186588	0.4689863	0.6174771	25.23	22.15	227.75	287.25
ORF-T	YKR082W	-0.186526	0.4255687	0.5807084	98.11	86.22	914.75	1141.00
ORF-T	YNL032W	-0.186403	0.4220595	0.5774044	44.90	39.49	416.25	508.00
ORF-T	YOR197W	-0.186393	0.4547539	0.6052182	46.50	40.88	436.75	536.50
ORF-T	YOR112W	-0.186337	0.4145946	0.5713779	48.78	42.88	448.00	557.00
ORF-T	YGR223C	-0.18625	0.4903894	0.6343921	84.64	74.41	777.25	958.50
ORF-T	YCR094W	-0.185709	0.4563296	0.6065148	30.05	26.40	274.50	347.25
ORF-T	YKR069W	-0.185592	0.4939312	0.6373387	31.40	27.63	290.50	356.00
ORF-T	YOL145C	-0.185539	0.4748179	0.6220736	172.64	151.82	1584.50	1970.50
ORF-T	YDL153C	-0.185427	0.4658689	0.6151397	94.22	82.93	893.50	1067.25
ORF-T	YJL157C	-0.18516	0.567184	0.6958547	160.11	140.82	1509.75	1901.25
ORF-T	YMR026C	-0.185079	0.4695664	0.6180796	25.74	22.64	230.75	288.75
ORF-T	YCR043C	-0.184983	0.4856184	0.6310535	21.05	18.55	194.75	238.00
ORF-T	YDL148C	-0.184577	0.4684228	0.6172995	164.81	145.07	1554.75	1877.25
ORF-T	YDR237W	-0.184476	0.4810686	0.6270807	43.02	37.89	396.75	482.25
ORF-T	YGL216W	-0.18438	0.4361502	0.5899583	52.22	45.95	492.00	623.25
ORF-T	YGL104C	-0.184301	0.5900984	0.7145235	15.92	13.97	140.75	184.75
SUT	SUT458	-0.184274	0.6079346	0.7284235	12.73	11.20	120.50	153.50
ORF-T	YMR289W	-0.18406	0.4308712	0.5853301	44.77	39.42	416.25	518.00
ORF-T	YDR299W	-0.184013	0.5211856	0.6593446	43.17	38.01	408.75	513.25
ORF-T	YMR294W	-0.183965	0.5342579	0.6700817	5.28	4.66	49.75	62.00
ORF-T	YKL002W	-0.183947	0.4506184	0.601859	47.49	41.83	428.50	527.25
ORF-T	YDR347W	-0.18394	0.4455208	0.5977423	70.18	61.81	639.00	782.75

Class	Transcript name	RRP6 KO_vs_WT log2_fold _change	RRP6 KO_vs_WT p-value	RRP6 KO_vs_WT FDR	Ave Norm Reads in WT	Ave Norm Reads in RRP6	Average RAW read counts in WT	Average RAW read counts in RRP6
ORF-T	YOR227W	-0.183729	0.5436331	0.6762964	21.30	18.74	185.00	234.75
ORF-T	YJR090C	-0.183658	0.4150473	0.5718454	52.67	46.33	475.00	609.50
ORF-T	YCL010C	-0.183617	0.4470417	0.5991452	51.29	45.21	479.50	587.50
ORF-T	YPR016C	-0.183449	0.4939254	0.6373387	31.49	27.65	276.50	370.75
SUT	SUT677	-0.183444	0.4607099	0.6101653	26.42	23.23	236.00	303.25
ORF-T	YGL213C	-0.183432	0.4361476	0.5899583	59.16	52.09	552.75	699.50
ORF-T	YNL166C	-0.183351	0.4335469	0.5880426	45.30	39.92	423.00	525.50
ORF-T	YIL068C	-0.182882	0.5687421	0.6972034	48.27	42.51	441.00	554.25
ORF-T	YBR255W	-0.182783	0.5358618	0.6714253	20.02	17.60	180.25	234.25
ORF-T	YPL101W	-0.182673	0.4637479	0.6132223	54.33	47.95	518.50	620.25
ORF-T	YOL044W	-0.18266	0.4574597	0.6075363	28.80	25.40	266.25	327.75
ORF-T	YOR090C	-0.182625	0.4820264	0.6279232	50.55	44.53	461.00	582.00
CUT	CUT234	-0.182338	0.5202606	0.6586702	8.81	7.76	81.25	102.00
ORF-T	YGR037C	-0.182106	0.4779846	0.6242376	33.14	29.17	295.50	384.50
ORF-T	YHL014C	-0.182064	0.5087858	0.6500185	12.45	11.01	118.00	144.25
ORF-T	YMR047C	-0.181869	0.4941837	0.6375829	93.80	82.66	861.75	1113.25
ORF-T	YMR166C	-0.181706	0.545895	0.678433	11.14	9.78	98.25	128.75
AST	AS_YKR035C	-0.181671	0.5482784	0.6801453	7.20	6.34	65.00	81.25
ORF-T	YGR058W	-0.181599	0.635167	0.7494762	2.71	2.35	23.50	31.00
ORF-T	YPR134W	-0.181498	0.5286514	0.6657825	10.16	8.96	93.25	117.00
ORF-T	YJL163C	-0.181455	0.5953652	0.7192599	18.32	16.16	162.75	202.75
ORF-T	YKL151C	-0.181371	0.6780941	0.7804451	41.73	36.79	352.75	441.50
ORF-T	YKL027W	-0.181364	0.4725371	0.6206127	29.56	26.13	279.50	340.75
ORF-T	YOR261C	-0.181008	0.5102833	0.6512666	364.30	321.38	3321.00	4034.50
ORF-T	YDR303C	-0.180965	0.433655	0.5880818	51.61	45.52	465.00	587.00
ORF-T	YGR274C	-0.180795	0.4372981	0.5907799	93.60	82.59	870.75	1089.00
ORF-T	YMR314W	-0.180574	0.5130635	0.6531666	19.61	17.29	179.75	228.25
ORF-T	YIL007C	-0.180386	0.5104547	0.6512666	13.67	12.02	120.50	156.00
ORF-T	YGR168C	-0.180344	0.4819888	0.6279232	25.50	22.51	238.50	302.75

TABLE S1: Differential expression data for RRP6 RNA-Seq dataset Page 149

Class	Transcript name	RRP6 KO_vs_WT log2_fold_change	RRP6 KO_vs_WT p-value	RRP6 KO_vs_WT FDR	Ave Norm Reads in WT	Ave Norm Reads in RRP6	Average RAW read counts in WT	Average RAW read counts in RRP6
ORF-T	YDR087C	-0.180324	0.4836703	0.629333	92.61	81.81	889.50	1067.00
ORF-T	YJR024C	-0.180314	0.4471848	0.5992573	90.03	79.49	828.00	1017.75
SUT	SUT329	-0.18022	0.5810476	0.7073867	7.38	6.50	64.75	83.00
ORF-T	YIL129C	-0.180102	0.5093179	0.6503688	189.90	167.60	1743.00	2232.75
ORF-T	YMR240C	-0.179755	0.4972283	0.6402005	16.80	14.85	154.25	193.25
ORF-T	YBR090C	-0.179592	0.447562	0.5993646	53.49	47.32	500.50	597.00
ORF-T	YJR112W-A	-0.179484	0.5092701	0.6503688	11.84	10.46	110.25	137.50
ORF-T	YGR093W	-0.179343	0.4401185	0.5933881	38.60	34.09	359.25	452.75
ORF-T	YOR363C	-0.179335	0.4964639	0.6394612	20.21	17.81	183.00	236.25
ORF-T	YDR280W	-0.17929	0.4488269	0.6002119	56.36	49.81	530.50	655.50
ORF-T	YOR037W	-0.179272	0.5064759	0.6481349	24.90	22.01	228.00	281.50
ORF-T	YDR313C	-0.179077	0.4606441	0.6101653	27.16	24.00	247.25	308.25
ORF-T	YLR368W	-0.17903	0.4726741	0.6207117	30.73	27.15	289.75	365.00
AST	AS_YBL062W	-0.178957	0.54587	0.678433	11.87	10.55	115.75	138.50
ORF-T	YBR252W	-0.178954	0.4888328	0.6335977	51.83	45.78	481.00	606.75
SUT	SUT475	-0.17885	0.6292988	0.7448481	4.36	3.82	37.50	49.25
ORF-T	YJR008W	-0.178824	0.5482487	0.6801453	34.08	30.13	303.75	372.50
ORF-T	YER022W	-0.178762	0.4444967	0.5967038	57.40	50.74	536.00	661.50
ORF-T	YPR101W	-0.178739	0.522768	0.6610147	10.28	9.04	91.75	119.75
sn/snoRNA ET	SNR10-ET	-0.178707	0.6693673	0.7744219	2.25	1.99	20.75	26.25
ORF-T	YDL126C	-0.178583	0.6007571	0.7238419	542.15	479.04	4972.25	6138.75
ORF-T	YLR459W	-0.178524	0.591003	0.71542	8.66	7.60	74.50	99.25
ORF-T	YJL140W	-0.178487	0.4761928	0.6233842	44.95	39.75	418.50	516.25
ORF-T	YLR443W	-0.178456	0.4674234	0.616265	99.56	88.01	923.75	1143.25
ORF-T	YOR117W	-0.178298	0.4862729	0.6315785	215.80	190.75	2017.50	2471.25
ORF-T	YNL182C	-0.178245	0.5159646	0.6556184	128.01	113.19	1239.25	1499.00
ORF-T	YDL176W	-0.178109	0.5092825	0.6503688	23.59	20.88	223.50	278.50
ORF-T	YPR032W	-0.177793	0.5668385	0.6956309	17.96	15.84	166.50	216.00
ORF-T	YGR292W	-0.177558	0.7644179	0.8456819	2.49	2.19	21.75	28.50

Class	Transcript name	RRP6 KO_vs_WT log2_fold _change	RRP6 KO_vs_WT p-value	RRP6 KO_vs_WT FDR	Ave Norm Reads in WT	Ave Norm Reads in RRP6	Average RAW read counts in WT	Average RAW read counts in RRP6
ORF-T	YMR265C	-0.177461	0.4453565	0.5976014	50.53	44.71	472.50	586.75
ORF-T	YKR050W	-0.177231	0.5410669	0.6744312	21.12	18.61	189.25	252.00
ORF-T	YKR047W	-0.177064	0.494701	0.6379239	19.88	17.63	188.00	231.00
CUT	CUT032	-0.176996	0.7089707	0.8034682	1.28	1.09	10.75	14.50
ORF-T	YLR127C	-0.176906	0.473118	0.6208801	28.70	25.41	266.25	332.75
CUT	CUT077	-0.176885	0.7290978	0.8193717	1.23	1.11	11.50	14.00
ORF-T	YOL066C	-0.176694	0.4782988	0.6243842	31.62	27.99	297.50	370.50
ORF-T	YBR058C	-0.1766	0.4535178	0.6042909	86.49	76.52	799.50	1012.50
ORF-T	YOR060C	-0.176508	0.4873347	0.6323875	30.63	27.08	274.75	353.00
ORF-T	YER017C	-0.176419	0.4422846	0.5949296	78.11	69.12	715.25	899.00
ORF-T	YOR030W	-0.176255	0.5020061	0.6442955	17.32	15.34	159.75	200.25
ORF-T	YGL112C	-0.176226	0.4526392	0.6035987	106.40	94.17	978.25	1227.25
ORF-T	YOR279C	-0.176208	0.5295603	0.6667316	11.44	10.13	108.75	137.25
ORF-T	YLR427W	-0.176038	0.4499374	0.6014275	64.33	56.98	596.00	737.00
ORF-T	YEL020C	-0.175945	0.5374679	0.6721849	39.95	35.42	372.50	451.75
ORF-T	YGR266W	-0.175869	0.4892093	0.6336784	88.66	78.52	836.25	1025.75
ORF-T	YOR362C	-0.175694	0.5123838	0.6524851	221.56	196.18	2029.00	2517.25
ORF-T	YBL093C	-0.175528	0.4551211	0.605547	36.90	32.73	339.75	415.25
ORF-T	YMR041C	-0.175471	0.4878235	0.6327775	17.70	15.65	159.25	204.25
ORF-T	YJL165C	-0.17547	0.5043161	0.6462489	129.33	114.53	1182.75	1482.00
ORF-T	YGR270W	-0.175356	0.5205059	0.6588153	112.96	100.01	1012.25	1305.25
ORF-T	YPL204W	-0.175107	0.476823	0.6237998	127.88	113.25	1171.00	1483.50
ORF-T	YCL051W	-0.175086	0.5463542	0.6786691	9.94	8.80	89.00	112.25
ORF-T	YDR219C	-0.175073	0.5082098	0.6495294	20.12	17.78	177.00	230.00
ORF-T	YNR003C	-0.174739	0.4929481	0.6368036	49.27	43.70	458.75	563.75
ORF-T	YPL247C	-0.174719	0.5859983	0.7109248	36.21	32.05	318.75	410.25
ORF-T	YGR240C	-0.174689	0.5332182	0.6691938	711.25	630.12	6437.00	8193.25
ORF-T	YDR301W	-0.174677	0.491563	0.6354146	85.86	76.08	812.50	1016.25
ORF-T	YDR352W	-0.174644	0.5090572	0.6502829	31.45	27.80	284.50	377.25

Class	Transcript name	RRP6 KO_vs_WT log2_fold _change	RRP6 KO_vs_WT p-value	RRP6 KO_vs_WT FDR	Ave Norm Reads in WT	Ave Norm Reads in RRP6	Average RAW read counts in WT	Average RAW read counts in RRP6
ORF-T	YBR238C	-0.174495	0.607821	0.728374	13.18	11.64	115.00	150.50
ORF-T	YJL098W	-0.173843	0.5094458	0.6504497	59.82	53.06	566.25	705.25
ORF-T	YDR152W	-0.17376	0.438489	0.59201	57.01	50.56	527.75	660.25
ORF-T	YNR059W	-0.173579	0.5264986	0.6642327	15.65	13.87	139.00	178.25
ORF-T	YLR077W	-0.173573	0.4774208	0.6241005	45.37	40.27	425.75	520.00
ORF-T	YNL062C	-0.173443	0.4992925	0.6417112	91.91	81.57	890.75	1075.25
ORF-T	YLR125W	-0.173271	0.6375201	0.7513104	3.37	3.02	32.50	40.00
ORF-T	YLR241W	-0.173217	0.5338663	0.6698405	138.99	123.29	1257.25	1556.50
ORF-T	YOR298C-A	-0.173076	0.5884621	0.713056	80.56	71.38	685.25	933.50
ORF-T	YGR257C	-0.173074	0.4729847	0.6208801	106.22	94.27	978.00	1187.00
ORF-T	YMR123W	-0.172824	0.5794454	0.7056917	14.75	13.05	133.50	173.00
ORF-T	YJR130C	-0.172492	0.4656387	0.6149465	69.79	61.94	649.50	816.75
ORF-T	YLR138W	-0.17248	0.5360855	0.6715989	332.38	294.99	3128.75	3751.75
ORF-T	YOL086W-A	-0.172468	0.5227375	0.6610147	17.09	15.17	154.25	193.00
ORF-T	YLR238W	-0.172282	0.5593324	0.689727	13.62	12.09	125.50	157.50
ORF-T	YGR007W	-0.172211	0.4522977	0.6034626	58.32	51.79	545.75	683.00
ORF-T	YOR099W	-0.172062	0.5402697	0.673767	463.49	411.41	4216.50	5171.00
ORF-T	YFR002W	-0.171854	0.5279396	0.6653015	141.34	125.46	1313.00	1667.00
ORF-T	YNL217W	-0.171601	0.4916344	0.6354146	82.34	73.10	756.25	960.25
ORF-T	YHR197W	-0.171586	0.5258773	0.663615	162.66	144.47	1574.75	1924.75
ORF-T	YJL101C	-0.171384	0.5370126	0.6718654	124.93	110.93	1134.75	1434.50
ORF-T	YOR324C	-0.171307	0.5412036	0.6744312	56.76	50.45	536.00	661.75
ORF-T	YHR032W-A	-0.171015	0.5752353	0.7020907	44.59	39.64	414.75	513.75
ORF-T	YGR280C	-0.17096	0.5256058	0.6635213	36.99	32.86	350.00	446.00
ORF-T	YMR207C	-0.170564	0.5162792	0.6558105	65.66	58.36	627.25	781.75
ORF-T	YLR024C	-0.170499	0.4983463	0.6412305	80.63	71.62	722.75	932.00
NUT	NUT0814	-0.170466	0.6831413	0.7840653	55.95	49.70	469.00	611.25
ORF-T	YPL259C	-0.170343	0.5203267	0.6586712	67.41	59.90	636.50	804.75
ORF-T	YHR100C	-0.170299	0.5520148	0.6831759	15.08	13.42	137.50	171.00

Class	Transcript name	RRP6 KO_vs_WT log2_fold _change	RRP6 KO_vs_WT p-value	RRP6 KO_vs_WT FDR	Ave Norm Reads in WT	Ave Norm Reads in RRP6	Average RAW read counts in WT	Average RAW read counts in RRP6
ORF-T	YOR159C	-0.170253	0.4908333	0.6348035	20.76	18.49	192.75	237.75
ORF-T	YBL069W	-0.170117	0.4910161	0.6348732	29.87	26.61	283.25	345.50
ORF-T	YCL055W	-0.169909	0.5272598	0.6645421	57.65	51.30	531.50	641.75
ORF-T	YJL166W	-0.169894	0.6378611	0.7515999	116.97	103.96	1019.00	1286.50
ORF-T	YGL255W	-0.169559	0.7225213	0.8145226	54.21	48.23	547.50	653.75
ORF-T	YPR088C	-0.169536	0.4981908	0.6411122	171.84	152.83	1600.50	1971.50
ORF-T	YBR107C	-0.169436	0.5556368	0.6862267	11.07	9.82	99.25	128.50
ORF-T	YPL230W	-0.169296	0.7439269	0.8305756	7.41	6.59	61.50	76.25
ORF-T	YBR168W	-0.169286	0.505855	0.6475032	28.01	24.94	255.50	318.00
ORF-T	YOR196C	-0.169199	0.4707649	0.6191722	53.44	47.56	503.00	624.25
ORF-T	YLR140W	-0.169134	0.7091165	0.8035432	1.44	1.26	13.25	17.50
ORF-T	YPL130W	-0.169024	0.6549587	0.7644217	2.13	1.86	19.25	25.50
ORF-T	YKL068W	-0.169018	0.5047104	0.646532	55.99	49.75	512.00	670.50
ORF-T	YKL004W	-0.168941	0.4829372	0.6286225	80.93	71.96	717.00	924.25
ORF-T	YER073W	-0.168908	0.5483837	0.6801453	286.03	254.47	2735.25	3370.50
ORF-T	YOR097C	-0.168861	0.5612676	0.6909807	14.32	12.75	128.25	160.50
ORF-T	YDR119W	-0.168832	0.5188183	0.657918	159.64	142.05	1466.75	1805.00
ORF-T	YGL115W	-0.168618	0.4883635	0.6331748	75.44	67.13	695.25	865.25
CUT	CUT235	-0.16835	0.5912695	0.7155979	7.85	7.08	76.50	89.75
ORF-T	YOR143C	-0.168057	0.5593601	0.689727	16.03	14.27	150.75	189.75
ORF-T	YNR032W	-0.167841	0.5281434	0.6654752	52.58	46.81	476.50	597.25
ORF-T	YLL027W	-0.167633	0.5396691	0.6733511	15.71	13.96	142.50	185.50
ORF-T	YNR008W	-0.167589	0.4906919	0.634702	32.41	28.91	302.75	369.75
ORF-T	YMR282C	-0.167404	0.4992286	0.6417108	60.41	53.85	568.00	690.50
ORF-T	YOL008W	-0.167183	0.5738038	0.7013622	18.41	16.35	166.50	219.75
ORF-T	YDR033W	-0.167106	0.6497392	0.759913	327.40	291.53	2834.50	3925.75
ORF-T	YLR096W	-0.1671	0.448249	0.5998862	49.63	44.18	453.00	583.25
ORF-T	YPL156C	-0.167082	0.6074641	0.7281194	7.10	6.30	65.00	84.00
SUT	SUT842	-0.166974	0.6116473	0.7307014	4.57	4.08	41.25	52.00

Class	Transcript name	RRP6 KO_vs_WT log2_fold _change	RRP6 KO_vs_WT p-value	RRP6 KO_vs_WT FDR	Ave Norm Reads in WT	Ave Norm Reads in RRP6	Average RAW read counts in WT	Average RAW read counts in RRP6
ORF-T	YML031W	-0.166862	0.477697	0.6242376	78.22	69.66	713.50	911.50
ORF-T	YMR155W	-0.166737	0.4953967	0.6384944	38.81	34.61	355.00	442.75
ORF-T	YBL008W	-0.166657	0.517028	0.6563426	45.38	40.50	441.25	536.25
ORF-T	YPL260W	-0.166385	0.5144615	0.654368	161.43	143.87	1484.25	1843.75
CUT	CUT628	-0.166152	0.6083169	0.7286219	31.20	27.73	267.00	369.75
ORF-T	YOR321W	-0.166095	0.5057123	0.6474043	103.61	92.36	945.75	1177.25
ORF-T	YML048W	-0.166037	0.5845163	0.7099316	341.38	304.26	3094.00	3962.00
ORF-T	YDR390C	-0.165959	0.5733407	0.700966	16.89	14.98	150.00	203.25
ORF-T	YDL111C	-0.165866	0.5377661	0.6723909	72.37	64.49	662.50	851.00
ORF-T	YPL064C	-0.165853	0.4970886	0.6401841	24.78	22.09	223.50	284.50
ORF-T	YML004C	-0.16579	0.5799279	0.7061941	278.56	248.32	2517.50	3170.75
SRT	SRT499	-0.165722	0.7863063	0.8622323	0.80	0.71	7.50	9.50
ORF-T	YBR023C	-0.165377	0.6444896	0.7565673	427.26	381.05	4054.00	4740.50
ORF-T	YBR036C	-0.165255	0.5146706	0.6544689	203.99	181.95	1864.25	2306.50
ORF-T	YNL156C	-0.165237	0.6068569	0.7277375	13.60	12.17	123.25	149.75
ORF-T	YJL125C	-0.165204	0.56987	0.6980769	15.78	14.05	147.25	190.75
ORF-T	YNL205C	-0.165191	0.6599584	0.768212	2.19	1.97	20.75	26.25
ORF-T	YDR335W	-0.165189	0.5563715	0.6869659	304.79	271.82	2797.00	3514.00
ORF-T	YHR195W	-0.165136	0.5507694	0.6822207	79.79	71.19	725.50	896.50
ORF-T	YMR165C	-0.165016	0.5248808	0.6630048	87.80	78.35	821.50	1017.25
ORF-T	YPR131C	-0.16501	0.6174015	0.7353108	12.73	11.32	114.25	149.75
ORF-T	YGL133W	-0.164922	0.4994818	0.6418727	52.61	46.96	503.50	631.75
ORF-T	YNL142W	-0.164872	0.6611014	0.7689212	25.37	22.64	234.50	297.25
ORF-T	YJR011C	-0.164763	0.5247015	0.6628777	14.23	12.75	133.25	162.00
ORF-T	YLR270W	-0.164754	0.6630537	0.7702146	40.75	36.33	355.75	460.00
ORF-T	YDR340W	-0.164548	0.7154685	0.8083791	1.96	1.72	16.75	22.50
ORF-T	YOR389W	-0.164447	0.6303109	0.7456658	4.77	4.31	44.75	53.75
ORF-T	YLR369W	-0.164397	0.5542348	0.6849986	39.42	35.19	362.25	453.00
ORF-T	YML096W	-0.164133	0.504328	0.6462489	25.97	23.18	239.50	307.00

Class	Transcript name	RRP6 KO_vs_WT log2_fold _change	RRP6 KO_vs_WT p-value	RRP6 KO_vs_WT FDR	Ave Norm Reads in WT	Ave Norm Reads in RRP6	Average RAW read counts in WT	Average RAW read counts in RRP6
ORF-T	YDL092W	-0.164058	0.4571277	0.6071753	46.72	41.69	425.50	542.25
ORF-T	YNR033W	-0.16373	0.5472213	0.6794114	87.15	77.81	801.75	1010.25
ORF-T	YKL188C	-0.163703	0.6352598	0.749498	18.06	16.12	166.25	211.25
ORF-T	YNL317W	-0.163701	0.5101592	0.6512666	37.13	33.11	343.00	446.50
ORF-T	YOR342C	-0.163691	0.5749063	0.7020123	11.34	10.11	102.00	131.25
ORF-T	YJR063W	-0.163669	0.6149254	0.7334013	5.95	5.29	53.75	70.25
ORF-T	YLR320W	-0.163611	0.4889723	0.6336157	46.73	41.72	433.25	552.00
ORF-T	YGR146C	-0.163274	0.5945806	0.7185703	11.82	10.53	104.00	134.75
ORF-T	YNL130C	-0.163185	0.5727254	0.7005693	36.44	32.53	333.25	425.75
ORF-T	YKL080W	-0.162981	0.5367139	0.6716583	292.37	261.15	2715.75	3410.50
ORF-T	YPL188W	-0.162892	0.5866632	0.7115602	52.07	46.49	484.00	624.75
ORF-T	YGL072C	-0.162828	0.5838794	0.7094508	8.12	7.27	75.75	95.75
ORF-T	YHR085W	-0.162657	0.6194852	0.7369243	7.20	6.44	67.75	86.25
ORF-T	YDL060W	-0.162581	0.5381215	0.672581	174.43	155.89	1683.50	2066.75
ORF-T	YNL179C	-0.162378	0.6357736	0.7498679	8.69	7.69	74.00	103.50
ORF-T	YLR148W	-0.162103	0.519637	0.6586248	72.46	64.77	663.00	836.75
ORF-T	YGR002C	-0.162062	0.5658898	0.6948565	12.86	11.43	115.75	155.50
ORF-T	YBR150C	-0.161816	0.5303287	0.6672282	69.32	62.00	633.25	791.50
ORF-T	YPL137C	-0.161793	0.4917469	0.6354146	134.46	120.14	1202.75	1580.75
ORF-T	YHR165C	-0.161778	0.5312488	0.6679501	281.58	251.74	2606.75	3270.50
ORF-T	YGR253C	-0.161449	0.5490558	0.680697	223.05	199.43	2004.00	2557.25
ORF-T	YDR365C	-0.161184	0.5616386	0.6911843	315.94	282.60	3004.00	3696.25
ORF-T	YBR207W	-0.161135	0.5430369	0.6758689	118.95	106.45	1097.00	1330.00
ORF-T	YML036W	-0.161049	0.6225341	0.7389729	4.23	3.78	39.25	50.25
ORF-T	YPL170W	-0.161046	0.6061379	0.7272213	26.80	24.02	247.50	300.75
ORF-T	YOR260W	-0.16072	0.5385236	0.6725877	49.98	44.65	441.75	586.25
ORF-T	YLR146W-A	-0.160256	0.6918457	0.791174	3.97	3.55	35.00	45.00
ORF-T	YIL028W	-0.160224	0.603318	0.7253915	8.57	7.68	80.50	103.00
ORF-T	YGR028W	-0.160199	0.5461928	0.6786318	75.05	67.21	681.00	838.50

Class	Transcript name	RRP6 KO_vs_WT log2_fold _change	RRP6 KO_vs_WT p-value	RRP6 KO_vs_WT FDR	Ave Norm Reads in WT	Ave Norm Reads in RRP6	Average RAW read counts in WT	Average RAW read counts in RRP6
ORF-T	YMR008C	-0.160107	0.6347074	0.7491092	250.42	224.12	2223.25	2796.25
ORF-T	YGL001C	-0.160107	0.4984783	0.6412529	126.52	113.26	1176.00	1467.50
ORF-T	YHR192W	-0.159897	0.5666064	0.6955153	16.49	14.71	144.25	191.00
ORF-T	YDR354W	-0.159822	0.6098702	0.7292495	47.31	42.31	438.00	578.50
ORF-T	YDR338C	-0.159814	0.5001091	0.6424332	34.06	30.47	307.75	399.00
ORF-T	YHR080C	-0.159761	0.6019546	0.7244433	56.42	50.51	515.50	648.50
ORF-T	YCL002C	-0.159745	0.5317825	0.6684735	52.11	46.68	475.50	591.00
ORF-T	YER050C	-0.159734	0.5602231	0.690493	17.40	15.59	161.75	204.00
ORF-T	YCR068W	-0.159718	0.5606506	0.6905947	14.32	12.78	129.25	170.75
ORF-T	YHR090C	-0.15964	0.5999004	0.7230066	7.47	6.68	67.75	87.50
ORF-T	YNL025C	-0.159179	0.6092353	0.7290291	7.06	6.31	63.50	81.75
ORF-T	YER075C	-0.159137	0.5653734	0.6945091	13.83	12.31	124.00	167.50
ORF-T	YKL212W	-0.159116	0.5393323	0.673264	334.56	299.66	3089.75	3836.75
ORF-T	YJR137C	-0.159067	0.5268421	0.6644251	75.66	67.72	680.25	891.50
ORF-T	YMR014W	-0.159064	0.5283316	0.6656292	40.79	36.60	390.75	480.50
ORF-T	YKL168C	-0.158668	0.5320643	0.6684945	50.04	44.82	452.00	577.25
ORF-T	YKR088C	-0.15859	0.5042078	0.6462489	44.52	39.84	391.75	513.50
ORF-T	YEL004W	-0.158294	0.5747075	0.7019562	17.51	15.68	158.50	203.25
ORF-T	YDR530C	-0.158164	0.5753932	0.7021983	16.43	14.70	149.25	196.25
ORF-T	YGR287C	-0.158078	0.5984457	0.72203	21.52	19.30	202.75	256.00
ORF-T	YKL145W	-0.157883	0.5726459	0.7005693	332.87	298.38	3003.00	3780.00
ORF-T	YLR072W	-0.157573	0.5363553	0.6716013	34.90	31.31	322.75	406.00
ORF-T	YGL090W	-0.157463	0.5882961	0.7129405	10.43	9.34	95.75	124.25
ORF-T	YLL049W	-0.15746	0.5761909	0.7026616	13.38	11.99	122.25	156.00
ORF-T	YMR036C	-0.157452	0.5044254	0.6462489	33.40	29.97	308.50	392.25
ORF-T	YDL013W	-0.157434	0.5851734	0.7102658	14.26	12.84	135.50	165.50
ORF-T	YPL164C	-0.157257	0.5429243	0.6758689	17.50	15.66	160.50	211.00
ORF-T	YIL112W	-0.157017	0.5443553	0.6769366	73.59	66.02	685.00	856.50
ORF-T	YJL091C	-0.1567	0.5681107	0.6965141	54.15	48.56	483.75	622.00

Class	Transcript name	RRP6 KO_vs_WT log2_fold _change	RRP6 KO_vs_WT p-value	RRP6 KO_vs_WT FDR	Ave Norm Reads in WT	Ave Norm Reads in RRP6	Average RAW read counts in WT	Average RAW read counts in RRP6
ORF-T	YOR269W	-0.156615	0.5706148	0.6986498	25.68	23.06	245.00	308.50
ORF-T	YNL291C	-0.156568	0.5270465	0.6644251	54.96	49.30	499.50	636.75
ORF-T	YOR381W	-0.15648	0.5423861	0.6754641	26.20	23.49	241.25	313.50
ORF-T	YER049W	-0.156368	0.589806	0.7144269	382.88	343.62	3670.25	4394.50
ORF-T	YDR265W	-0.156268	0.5159243	0.6556184	41.98	37.70	382.50	479.25
ORF-T	YPL043W	-0.156263	0.5243506	0.6626004	53.00	47.63	498.50	613.50
ORF-T	YKL026C	-0.156237	0.7152264	0.8083774	7.72	6.91	65.50	85.50
ORF-T	YKR031C	-0.15621	0.5537657	0.6845868	141.79	127.26	1308.75	1657.25
ORF-T	YGL086W	-0.155901	0.5190657	0.6580662	45.83	41.15	423.00	537.25
SUT	SUT423	-0.155708	0.6762329	0.779234	1.71	1.52	15.25	19.75
ORF-T	YGR005C	-0.155619	0.5484195	0.6801453	30.84	27.73	285.25	351.25
SUT	SUT837	-0.155423	0.7592944	0.8419534	4.62	4.18	43.00	50.25
ORF-T	YDR038C	-0.154633	0.5484066	0.6801453	25.03	22.53	233.25	291.25
ORF-T	YMR108W	-0.154409	0.560819	0.6905969	326.47	293.31	2982.25	3873.75
NUT	NUT0734	-0.154213	0.6083066	0.7286219	21.37	19.19	185.50	240.50
ORF-T	YDL190C	-0.154176	0.5439082	0.676464	106.19	95.38	962.50	1273.00
ORF-T	YNL311C	-0.153867	0.6216332	0.738432	9.54	8.54	87.50	116.00
ORF-T	YOL049W	-0.153737	0.6534696	0.7629487	131.04	117.80	1232.75	1574.50
SUT	SUT332	-0.153675	0.7383334	0.8264203	1.84	1.65	16.75	21.75
ORF-T	YBR216C	-0.153549	0.5521972	0.6833177	48.54	43.63	440.75	568.50
ORF-T	YDR213W	-0.153422	0.5378896	0.6724434	59.30	53.34	552.25	700.50
ORF-T	YNL248C	-0.153357	0.535855	0.6714253	56.48	50.81	533.50	676.50
ORF-T	YKL015W	-0.153327	0.5129974	0.6531649	125.94	113.26	1176.75	1497.50
ORF-T	YCR092C	-0.153242	0.5564499	0.6869785	36.73	33.04	348.50	447.00
ORF-T	YDL141W	-0.153187	0.6391163	0.7523755	11.52	10.32	105.00	139.25
ORF-T	YPR070W	-0.153115	0.495866	0.638936	51.15	45.99	468.50	605.50
ORF-T	YOR230W	-0.153094	0.6478026	0.7587922	133.91	120.39	1233.75	1652.50
ORF-T	YDR321W	-0.152799	0.5929098	0.7171528	426.90	384.06	4016.00	4854.25
ORF-T	YCL047C	-0.152691	0.5366289	0.6716583	36.13	32.48	332.25	427.75

Class	Transcript name	RRP6 KO_vs_WT log2_fold _change	RRP6 KO_vs_WT p-value	RRP6 KO_vs_WT FDR	Ave Norm Reads in WT	Ave Norm Reads in RRP6	Average RAW read counts in WT	Average RAW read counts in RRP6
ORF-T	YML030W	-0.152561	0.6380975	0.7516885	19.91	17.88	173.25	227.75
ORF-T	YJL161W	-0.152553	0.7294151	0.8195456	2.56	2.31	22.50	28.75
ORF-T	YGL057C	-0.152543	0.6337715	0.7484614	6.85	6.13	60.75	80.50
ORF-T	YER115C	-0.152534	0.5400406	0.6736478	19.63	17.69	182.75	229.25
ORF-T	YDL017W	-0.152532	0.5900421	0.7145235	20.62	18.63	195.00	234.75
ORF-T	YDR487C	-0.152504	0.558448	0.6890236	157.82	141.98	1474.75	1904.50
ORF-T	YLR034C	-0.152493	0.5451201	0.677637	54.05	48.64	488.25	627.75
ORF-T	YMR213W	-0.152489	0.5707701	0.6987551	14.25	12.81	131.50	171.00
ORF-T	YOR319W	-0.151948	0.5580834	0.6886579	19.86	17.87	182.00	233.25
ORF-T	YML081W	-0.151757	0.5302947	0.6672282	39.90	35.93	357.75	455.50
ORF-T	YPR119W	-0.151749	0.6293242	0.7448481	83.97	75.54	777.50	1058.50
ORF-T	YJL194W	-0.151562	0.6375409	0.7513104	6.97	6.27	64.00	83.00
ORF-T	YLR341W	-0.15142	0.7585828	0.8415345	0.99	0.86	8.75	11.75
ORF-T	YDR108W	-0.15123	0.5703634	0.6985965	17.49	15.72	159.75	209.00
ORF-T	YJR031C	-0.150988	0.5358294	0.6714253	88.60	79.78	826.00	1075.25
ORF-T	YDR243C	-0.150894	0.6100036	0.7292562	13.18	11.86	119.25	154.50
ORF-T	YFL046W	-0.150867	0.651705	0.7614174	5.77	5.14	50.00	67.75
SRT	SRT109	-0.1506	0.7978004	0.8689202	0.76	0.71	7.25	8.75
ORF-T	YJR055W	-0.150507	0.6790706	0.7809817	4.97	4.47	46.00	59.75
ORF-T	YLR166C	-0.150485	0.5511817	0.6824799	66.87	60.22	607.50	792.75
ORF-T	YKL122C	-0.15039	0.6279937	0.7436658	5.66	5.07	50.75	66.75
ORF-T	YKR010C	-0.150331	0.5731366	0.7008561	13.85	12.46	123.75	162.50
ORF-T	YDR145W	-0.150002	0.5347805	0.6706537	36.34	32.75	337.25	434.50
ORF-T	YDR465C	-0.149798	0.5435403	0.6762964	55.40	49.99	527.75	658.75
ORF-T	YDL180W	-0.149754	0.6042016	0.7258644	14.03	12.65	127.25	161.50
ORF-T	YPR168W	-0.149615	0.5991774	0.7226534	8.34	7.49	76.00	100.25
ORF-T	YOL089C	-0.149545	0.582377	0.7085775	15.43	13.86	137.50	182.75
ORF-T	YDR492W	-0.149488	0.6383718	0.7518502	21.51	19.39	199.00	259.25
ORF-T	YER098W	-0.149215	0.6072802	0.728072	36.81	33.18	334.00	428.75

Class	Transcript name	RRP6 KO_vs_WT log2_fold _change	RRP6 KO_vs_WT p-value	RRP6 KO_vs_WT FDR	Ave Norm Reads in WT	Ave Norm Reads in RRP6	Average RAW read counts in WT	Average RAW read counts in RRP6
ORF-T	YOL078W	-0.14913	0.5162883	0.6558105	83.11	74.99	775.25	970.50
ORF-T	YGL005C	-0.149049	0.5670618	0.6958203	19.59	17.66	176.50	226.75
ORF-T	YHR058C	-0.148947	0.5533817	0.6843636	28.68	25.85	266.25	347.00
ORF-T	YPR184W	-0.148902	0.7356407	0.8245194	73.09	65.90	604.25	807.25
ORF-T	YDL155W	-0.148298	0.5107207	0.65136	64.39	58.12	597.75	764.25
ORF-T	YDR167W	-0.147828	0.5390749	0.673026	37.49	33.92	353.75	434.00
ORF-T	YDL069C	-0.147786	0.6439927	0.7563501	6.26	5.66	55.50	70.00
ORF-T	YMR177W	-0.1477	0.5744969	0.7018539	19.69	17.79	184.50	235.50
ORF-T	YDL074C	-0.147235	0.5139696	0.6540724	65.04	58.74	606.50	777.50
ORF-T	YNL001W	-0.147204	0.5429797	0.6758689	67.95	61.44	640.00	787.75
ORF-T	YBR020W	-0.147023	0.6895342	0.7894258	6.46	5.85	58.75	74.25
ORF-T	YFR014C	-0.146997	0.6681118	0.7735143	62.48	56.42	552.00	705.75
ORF-T	YHL017W	-0.146718	0.5268834	0.6644251	91.97	83.10	843.00	1070.00
ORF-T	YOR078W	-0.146458	0.5740029	0.7014456	24.00	21.63	217.75	289.75
ORF-T	YER069W	-0.146294	0.5763823	0.70281	31.86	28.73	294.25	396.25
ORF-T	YIR017C	-0.146243	0.7892831	0.863996	2.00	1.77	16.50	23.25
ORF-T	YOR207C	-0.146193	0.5876338	0.7124803	276.76	250.12	2614.00	3306.75
AST	AS_YKL139W	-0.146183	0.785063	0.8612466	0.93	0.84	8.25	10.50
ORF-T	YNL101W	-0.146079	0.5413354	0.674512	43.99	39.71	400.75	533.25
ORF-T	YJR140C	-0.145832	0.5460364	0.6785251	119.77	108.25	1098.25	1414.00
ORF-T	YBR199W	-0.145822	0.5881184	0.7128965	194.33	175.67	1758.75	2208.75
SRT	SRT408	-0.145759	0.7661786	0.8464225	1.00	0.92	9.25	11.75
ORF-T	YGL257C	-0.14552	0.5155912	0.6553916	71.90	65.03	667.50	844.50
ORF-T	YER172C	-0.145323	0.5362997	0.6716013	71.29	64.49	659.75	840.75
ORF-T	YJL198W	-0.145238	0.5731813	0.7008561	158.65	143.49	1484.00	1885.75
ORF-T	YLL038C	-0.145174	0.5513835	0.6826459	32.45	29.37	302.50	384.25
ORF-T	YIL095W	-0.144797	0.6471302	0.7582688	10.46	9.37	87.50	121.75
ORF-T	YBR159W	-0.144784	0.614529	0.7332754	123.25	111.48	1115.25	1423.25
ORF-T	YIR021W	-0.144478	0.5829787	0.7088937	15.10	13.60	134.50	181.25

TABLE S1: Differential expression data for RRP6 RNA-Seq dataset Page 159

Class	Transcript name	RRP6 KO_vs_WT log2_fold _change	RRP6 KO_vs_WT p-value	RRP6 KO_vs_WT FDR	Ave Norm Reads in WT	Ave Norm Reads in RRP6	Average RAW read counts in WT	Average RAW read counts in RRP6
ORF-T	YLR038C	-0.144335	0.6414546	0.7545565	43.11	39.00	372.25	478.00
SRT	SRT600	-0.144217	0.7771386	0.8548935	4.68	4.27	43.50	51.25
ORF-T	YJL107C	-0.14415	0.7725578	0.8521629	24.19	21.90	254.75	333.00
ORF-T	YLR173W	-0.144075	0.5783327	0.704677	43.16	39.10	402.75	508.50
ORF-T	YCR090C	-0.144074	0.5296434	0.6667316	43.12	39.05	397.00	503.00
ORF-T	YHR012W	-0.143935	0.5308478	0.6676315	55.78	50.47	507.75	658.75
ORF-T	YDR190C	-0.143883	0.5639691	0.6931218	214.65	194.33	2026.50	2512.25
ORF-T	YOR206W	-0.143784	0.5598648	0.6902649	163.56	148.09	1530.25	1923.75
CUT	CUT155	-0.14322	0.5698673	0.6980769	41.38	37.47	370.75	481.00
ORF-T	YBL079W	-0.143141	0.5705943	0.6986498	161.23	145.96	1476.50	1960.00
ORF-T	YNL049C	-0.142717	0.571592	0.6995065	111.27	100.77	1022.75	1327.25
ORF-T	YLL033W	-0.142608	0.7106836	0.8047764	2.87	2.57	25.00	33.50
ORF-T	YBR239C	-0.142532	0.6812149	0.782656	3.51	3.10	30.50	42.50
AST	AS_YKL202W	-0.142418	0.7457029	0.8318784	1.97	1.78	17.50	23.00
ORF-T	YPL069C	-0.142418	0.6052045	0.7266201	10.84	9.77	99.25	132.50
ORF-T	YHR190W	-0.142326	0.6094759	0.7290831	84.93	76.91	758.75	1006.75
ORF-T	YOR057W	-0.14216	0.5868762	0.7116882	30.12	27.28	281.00	365.75
ORF-T	YML129C	-0.142047	0.6703859	0.7748584	7.65	6.89	66.25	89.25
ORF-T	YCR095C	-0.142029	0.5492585	0.6808509	31.58	28.67	300.00	377.50
ORF-T	YDR101C	-0.141974	0.606728	0.7276695	253.53	229.80	2393.75	3030.25
ORF-T	YDR300C	-0.14195	0.5637718	0.6929638	130.07	117.92	1217.50	1528.50
ORF-T	YER105C	-0.141716	0.5636969	0.6929561	152.41	138.11	1384.25	1844.25
ORF-T	YKL222C	-0.141705	0.5826822	0.7087777	17.91	16.23	163.25	211.00
ORF-T	YBR192W	-0.141566	0.5493399	0.6808682	43.67	39.67	416.25	517.00
ORF-T	YGR281W	-0.141371	0.6614004	0.7690915	264.33	239.66	2415.50	3119.00
ORF-T	YLR346C	-0.141311	0.677639	0.7800585	4.35	3.95	39.50	51.00
ORF-T	YLR277C	-0.141294	0.574908	0.7020123	48.96	44.40	462.00	596.00
ORF-T	YOR038C	-0.141199	0.5502381	0.6816816	58.08	52.69	543.75	690.00
AST	AS_YPL261C	-0.140843	0.6639972	0.7708667	5.05	4.63	47.50	58.25

Class	Transcript name	RRP6 KO_vs_WT log2_fold _change	RRP6 KO_vs_WT p-value	RRP6 KO_vs_WT FDR	Ave Norm Reads in WT	Ave Norm Reads in RRP6	Average RAW read counts in WT	Average RAW read counts in RRP6
ORF-T	YOR031W	-0.140507	0.6956826	0.7936716	3.50	3.18	31.75	41.00
ORF-T	YEL025C	-0.140475	0.6008089	0.7238419	93.32	84.65	860.00	1123.00
ORF-T	YOR104W	-0.140447	0.6011635	0.7240096	20.39	18.47	186.25	249.00
ORF-T	YHR144C	-0.140445	0.5821364	0.7083946	24.87	22.56	227.50	294.50
ORF-T	YBR249C	-0.140418	0.6062704	0.7272937	188.32	170.81	1674.25	2243.50
SUT	SUT804	-0.140125	0.7480798	0.8340118	1.92	1.73	18.25	24.50
ORF-T	YPR097W	-0.140068	0.5899007	0.7144557	103.41	93.85	960.75	1229.25
ORF-T	YKR055W	-0.139962	0.6791381	0.7809817	13.29	12.07	119.50	150.75
ORF-T	YPL270W	-0.139938	0.6507813	0.7607789	102.85	93.33	949.00	1226.75
ORF-T	YAR027W	-0.139813	0.6273025	0.7432025	33.76	30.63	299.25	391.25
ORF-T	YGR232W	-0.139765	0.6453914	0.7571006	36.40	33.01	329.50	431.75
ORF-T	YAL022C	-0.139572	0.5672328	0.6958547	111.17	100.92	1014.50	1303.75
ORF-T	YER039C	-0.139471	0.5885995	0.7131368	16.68	15.17	153.25	195.75
ORF-T	YIL059C	-0.139433	0.6763819	0.779234	6.60	5.91	57.25	81.50
ORF-T	YDR080W	-0.139357	0.5729421	0.7007335	42.63	38.72	399.25	513.00
ORF-T	YMR060C	-0.139287	0.5922432	0.7166044	23.61	21.42	212.50	278.25
NUT	NUT1197	-0.139061	0.6388124	0.7521056	15.87	14.35	137.00	186.25
ORF-T	YNL008C	-0.139	0.583973	0.7094508	50.29	45.67	471.25	609.75
SUT	SUT567	-0.138945	0.6960339	0.7938928	3.87	3.47	34.00	46.00
ORF-T	YFL057C	-0.138865	0.68378	0.7843646	7.03	6.39	64.25	82.75
ORF-T	YEL024W	-0.138594	0.7565512	0.8400271	27.82	25.24	231.75	310.25
ORF-T	YNL303W	-0.13847	0.7709692	0.8506898	1.13	0.98	9.25	12.75
ORF-T	YEL073C	-0.138397	0.6749548	0.7782149	5.87	5.31	51.50	68.75
ORF-T	YEL029C	-0.137972	0.6029276	0.7250951	23.93	21.72	222.50	295.50
ORF-T	YNL201C	-0.137831	0.5829884	0.7088937	97.58	88.76	946.00	1176.25
ORF-T	YKL014C	-0.13776	0.5646265	0.6937847	102.11	92.82	947.50	1226.00
ORF-T	YBR069C	-0.137509	0.6226674	0.7389729	531.23	482.96	5013.50	6407.00
ORF-T	YJL011C	-0.137376	0.6467781	0.7581205	34.57	31.46	320.75	404.00
ORF-T	YLR090W	-0.137331	0.6027859	0.7250111	55.19	50.22	520.25	655.75

Class	Transcript name	RRP6 KO_vs_WT log2_fold _change	RRP6 KO_vs_WT p-value	RRP6 KO_vs_WT FDR	Ave Norm Reads in WT	Ave Norm Reads in RRP6	Average RAW read counts in WT	Average RAW read counts in RRP6
ORF-T	YBR254C	-0.137101	0.6054573	0.7267508	21.82	19.85	204.50	264.25
ORF-T	YML055W	-0.137066	0.6247964	0.7411419	12.10	11.00	109.00	142.25
ORF-T	YPR108W-A	-0.136972	0.726815	0.8178088	6.12	5.53	54.00	75.00
ORF-T	YER053C-A	-0.136642	0.7501658	0.8354903	1.98	1.76	17.00	23.00
ORF-T	YDL178W	-0.136569	0.5928568	0.7171528	134.56	122.49	1258.25	1541.50
ORF-T	YER153C	-0.136565	0.7039507	0.8000577	3.89	3.54	36.00	46.75
ORF-T	YDR073W	-0.136367	0.6213545	0.7381879	28.68	26.07	267.25	358.25
ORF-T	YDR264C	-0.136187	0.5964329	0.7200322	297.04	270.33	2753.00	3483.75
ORF-T	YBL049W	-0.136182	0.755568	0.8396769	7.18	6.56	63.50	77.75
ORF-T	YHR066W	-0.135539	0.6338228	0.7484614	93.38	85.01	882.50	1145.00
SUT	SUT530	-0.135342	0.751751	0.8365325	1.00	0.94	9.50	12.00
ORF-T	YPL042C	-0.135302	0.582832	0.7088745	46.70	42.53	435.25	562.50
ORF-T	YHR067W	-0.13494	0.6037939	0.7257042	21.20	19.32	197.00	252.75
ORF-T	YJR126C	-0.134799	0.6054575	0.7267508	176.25	160.55	1609.00	2039.25
ORF-T	YOR266W	-0.134738	0.6059132	0.7271246	22.13	20.15	204.00	265.50
ORF-T	YKL018W	-0.134673	0.6921989	0.7914881	7.56	6.82	65.00	90.00
AST	AS_YOL152W	-0.134553	0.7148303	0.8081898	3.82	3.51	38.50	49.25
ORF-T	YGL064C	-0.134373	0.7038457	0.8000577	3.20	2.93	29.50	37.75
ORF-T	YMR269W	-0.134267	0.6735075	0.7774324	10.30	9.39	97.75	127.50
ORF-T	YOL016C	-0.134176	0.6198502	0.7371824	129.17	117.71	1174.75	1509.25
ORF-T	YPR152C	-0.134151	0.6204346	0.7377904	18.53	16.90	172.00	222.50
ORF-T	YBR133C	-0.134031	0.5723516	0.7003511	86.41	78.73	807.00	1060.25
ORF-T	YGR179C	-0.133983	0.6135325	0.7325277	19.34	17.57	174.25	235.50
ORF-T	YDR054C	-0.133871	0.5734364	0.7009981	40.07	36.49	354.75	468.00
ORF-T	YFL029C	-0.133434	0.6355299	0.7497289	43.08	39.28	407.00	519.50
ORF-T	YGR062C	-0.133141	0.5991111	0.7226534	25.28	23.02	226.75	300.75
ORF-T	YPL177C	-0.133092	0.6649151	0.7716594	9.90	8.97	85.50	118.00
ORF-T	YGR159C	-0.133071	0.6830136	0.7840079	518.98	473.30	4855.25	5983.25
ORF-T	YBL104C	-0.133039	0.5635821	0.6929054	55.55	50.68	520.75	669.75

Class	Transcript name	RRP6 KO_vs_WT log2_fold _change	RRP6 KO_vs_WT p-value	RRP6 KO_vs_WT FDR	Ave Norm Reads in WT	Ave Norm Reads in RRP6	Average RAW read counts in WT	Average RAW read counts in RRP6
ORF-T	YKL079W	-0.133038	0.5751586	0.702082	76.87	70.12	704.25	902.50
ORF-T	YOR212W	-0.1326	0.603869	0.7257078	199.14	181.72	1891.00	2349.50
ORF-T	YDR324C	-0.132486	0.6220881	0.7387114	168.69	153.93	1611.50	2037.25
ORF-T	YKR075C	-0.132446	0.6660054	0.7722201	55.37	50.54	534.50	680.75
ORF-T	YGR076C	-0.132418	0.7556742	0.8396769	3.33	3.01	29.50	39.75
ORF-T	YER122C	-0.13223	0.5946705	0.7185929	171.32	156.37	1622.75	2045.50
ORF-T	YKL059C	-0.132094	0.610141	0.7293341	17.43	15.91	160.25	207.50
ORF-T	YKL082C	-0.132009	0.6226748	0.7389729	41.82	38.22	398.25	499.50
ORF-T	YJL046W	-0.131663	0.6222374	0.7388016	24.06	21.94	216.75	284.50
ORF-T	YCL012C	-0.131609	0.7524803	0.8371269	1.49	1.34	13.25	17.75
SUT	SUT311	-0.131547	0.7855023	0.8616345	1.28	1.15	11.75	15.75
ORF-T	YLR051C	-0.131522	0.6240674	0.7404513	25.14	22.96	232.75	302.00
ORF-T	YOR292C	-0.13152	0.6679669	0.7735143	16.53	15.08	144.50	190.00
ORF-T	YBR226C	-0.131481	0.7905637	0.8644844	1.03	0.94	9.50	12.50
AST	AS_YDL214C	-0.131396	0.7420435	0.8292073	3.25	2.96	30.75	41.00
ORF-T	YER127W	-0.131293	0.6741736	0.777932	18.69	17.05	178.25	239.50
ORF-T	YLR397C	-0.131281	0.6141792	0.7330314	140.07	127.93	1337.25	1707.75
SUT	SUT845	-0.131201	0.6940993	0.792852	4.75	4.37	44.50	56.50
ORF-T	YER048W-A	-0.131055	0.6035429	0.7255754	46.07	42.06	413.50	544.75
ORF-T	YGR212W	-0.131029	0.6990309	0.7962925	5.08	4.60	45.25	60.75
ORF-T	YPL151C	-0.130927	0.6009481	0.7239232	54.81	50.09	512.50	656.75
ORF-T	YDR381W	-0.130916	0.5981492	0.7217585	252.78	230.87	2301.00	2966.25
ORF-T	YEL016C	-0.130667	0.5812412	0.707537	31.80	29.03	286.25	375.75
ORF-T	YFL041W-A	-0.130377	0.7201543	0.8122174	4.65	4.19	39.50	54.75
ORF-T	YOR056C	-0.130213	0.6040988	0.7258644	145.77	133.25	1388.00	1735.75
ORF-T	YGR229C	-0.130053	0.6024558	0.7247425	200.80	183.53	1864.00	2353.00
ORF-T	YLR375W	-0.129989	0.6454575	0.7571006	22.61	20.64	201.00	266.00
SUT	SUT258	-0.129846	0.5869099	0.7116882	51.22	46.81	473.75	622.50
SUT	SUT198	-0.129837	0.7434533	0.8303227	4.56	4.19	43.25	55.75

Class	Transcript name	RRP6 KO_vs_WT log2_fold _change	RRP6 KO_vs_WT p-value	RRP6 KO_vs_WT FDR	Ave Norm Reads in WT	Ave Norm Reads in RRP6	Average RAW read counts in WT	Average RAW read counts in RRP6
ORF-T	YPR024W	-0.129798	0.620646	0.7378677	222.91	203.76	2068.75	2644.50
ORF-T	YOR137C	-0.129785	0.630114	0.7455203	32.06	29.30	294.25	385.00
ORF-T	YDR130C	-0.129641	0.625499	0.7416264	20.86	19.09	190.00	245.00
ORF-T	YER039C-A	-0.129537	0.7142899	0.807681	2.98	2.73	27.75	36.25
ORF-T	YHL035C	-0.129365	0.6018779	0.7244374	45.47	41.51	411.25	561.00
ORF-T	YHR138C	-0.129126	0.7384509	0.8264203	31.89	29.17	274.25	349.25
ORF-T	YER076C	-0.129006	0.6091036	0.7290291	21.54	19.73	200.75	258.00
NUT	NUT0667	-0.128684	0.7928052	0.8660652	5.97	5.47	55.25	72.25
ORF-T	YJR046W	-0.128577	0.5936963	0.7177596	96.01	87.88	907.00	1137.25
ORF-T	YNL103W	-0.128486	0.6206447	0.7378677	22.34	20.36	198.25	270.75
ORF-T	YBL006C	-0.127895	0.61136	0.7304448	87.19	79.86	803.50	997.00
ORF-T	YOR067C	-0.12753	0.6011532	0.7240096	60.81	55.71	555.50	701.50
ORF-T	YJL024C	-0.127506	0.6647324	0.7715425	11.99	10.93	107.75	147.50
ORF-T	YDL116W	-0.127495	0.5928029	0.7171528	144.86	132.64	1356.50	1728.00
ORF-T	YJL102W	-0.127435	0.6474473	0.7585521	61.52	56.31	542.25	713.00
AST	AS_YML052W	-0.127345	0.7632785	0.8447921	1.77	1.60	16.00	21.50
ORF-T	YER094C	-0.126811	0.6410588	0.75431	222.85	204.15	2082.75	2606.00
ORF-T	YBL055C	-0.126572	0.6014603	0.7241941	51.15	46.93	489.00	608.75
ORF-T	YLR387C	-0.126354	0.648433	0.7593541	49.73	45.55	442.75	579.25
ORF-T	YJL037W	-0.12617	0.799936	0.8698032	2.43	2.25	24.00	29.75
ORF-T	YDR459C	-0.126054	0.6590186	0.7674335	38.92	35.69	360.25	466.25
ORF-T	YHL029C	-0.125929	0.656581	0.7653471	9.72	8.89	85.50	112.75
ORF-T	YOL055C	-0.125366	0.6379773	0.7516489	22.01	20.21	204.00	261.25
ORF-T	YDL113C	-0.125301	0.6638685	0.770806	56.56	51.86	511.25	659.00
sn/snoRNA	SNR4	-0.125202	0.7397879	0.8273263	874.57	801.81	7150.00	10298.25
ORF-T	YHR081W	-0.124951	0.6252058	0.7415402	22.41	20.55	207.00	271.75
ORF-T	YMR192W	-0.124855	0.6533171	0.762859	15.65	14.29	137.75	189.25
AST	AS_YMR253C	-0.124768	0.7558258	0.8396769	2.44	2.23	22.75	30.25
ORF-T	YAR028W	-0.124732	0.7650565	0.8458315	15.61	14.29	137.00	185.75

TABLE S1: Differential expression data for RRP6 RNA-Seq dataset Page 164

Class	Transcript name	RRP6 KO_vs_WT log2_fold _change	RRP6 KO_vs_WT p-value	RRP6 KO_vs_WT FDR	Ave Norm Reads in WT	Ave Norm Reads in RRP6	Average RAW read counts in WT	Average RAW read counts in RRP6
ORF-T	YNL194C	-0.124731	0.8367103	0.89452	8.75	8.02	70.25	94.50
ORF-T	YGL075C	-0.124684	0.6959573	0.7938928	11.26	10.32	98.25	129.00
ORF-T	YPR036W	-0.124643	0.6764819	0.7792601	456.62	418.78	4145.50	5673.00
ORF-T	YML130C	-0.124523	0.6427516	0.7558384	223.86	205.39	2045.00	2577.50
ORF-T	YPR055W	-0.124355	0.6433695	0.7559153	31.72	29.08	295.00	391.25
ORF-T	YDR458C	-0.124336	0.6432781	0.7559153	23.63	21.66	212.00	280.25
ORF-T	YPL052W	-0.124275	0.6392104	0.7523984	55.71	51.20	543.50	672.50
ORF-T	YGL060W	-0.124193	0.7064322	0.8020336	28.29	25.91	257.75	352.75
ORF-T	YBR149W	-0.12412	0.7150769	0.808299	67.63	62.03	573.25	769.50
ORF-T	YDR004W	-0.124107	0.6099256	0.7292495	47.25	43.39	436.50	560.00
ORF-T	YLR347C	-0.124032	0.6305062	0.7457219	295.72	271.37	2738.50	3524.25
ORF-T	YDR412W	-0.123724	0.6492723	0.7598408	31.00	28.47	292.25	379.00
ORF-T	YKL139W	-0.12366	0.6048867	0.726325	31.07	28.57	290.75	369.00
ORF-T	YGL110C	-0.123476	0.624446	0.7408134	26.96	24.75	247.25	321.25
ORF-T	YEL037C	-0.123445	0.6615571	0.7691851	15.68	14.41	145.25	189.25
ORF-T	YHR211W	-0.123436	0.6963064	0.7941138	10.33	9.45	90.00	121.00
ORF-T	YPR026W	-0.123374	0.6456822	0.7572761	38.54	35.37	340.50	451.75
ORF-T	YKL040C	-0.123169	0.6085015	0.7286699	48.46	44.47	442.25	586.75
ORF-T	YCL036W	-0.123064	0.6928153	0.791744	49.44	45.43	480.25	610.25
ORF-T	YLR418C	-0.122918	0.6160646	0.7341521	75.26	69.14	703.25	904.50
ORF-T	YFL032W	-0.122801	0.6688003	0.7739638	1589.89	1460.22	15347.25	19242.75
sn/snoRNA	SNR41	-0.122609	0.6652922	0.7716594	268.33	246.40	2367.50	3288.75
ORF-T	YKR022C	-0.122597	0.6521937	0.7618585	25.22	23.18	227.00	294.75
ORF-T	YPL038W-A	-0.122547	0.7354957	0.8244485	1.91	1.79	18.00	23.00
ORF-T	YPL107W	-0.122481	0.6643018	0.7711315	15.27	14.05	138.50	179.00
ORF-T	YLL048C	-0.122232	0.6326491	0.7476425	479.37	440.45	4471.25	5782.75
ORF-T	YMR110C	-0.122005	0.6596758	0.7680603	71.33	65.55	647.00	840.50
ORF-T	YDR104C	-0.121941	0.6438944	0.7563228	26.46	24.34	245.00	314.00
ORF-T	YBR096W	-0.121931	0.6361239	0.7501665	36.71	33.72	329.50	435.50

Class	Transcript name	RRP6 KO_vs_WT log2_fold _change	RRP6 KO_vs_WT p-value	RRP6 KO_vs_WT FDR	Ave Norm Reads in WT	Ave Norm Reads in RRP6	Average RAW read counts in WT	Average RAW read counts in RRP6
ORF-T	YPL235W	-0.121851	0.6186328	0.7362208	83.24	76.57	781.50	982.25
ORF-T	YOL124C	-0.121698	0.6212009	0.7381793	51.65	47.53	497.25	628.25
ORF-T	YOR147W	-0.121609	0.6045649	0.7261272	85.70	78.81	801.75	1027.50
ORF-T	YJR084W	-0.12156	0.6492125	0.7598408	39.23	36.06	362.75	474.00
ORF-T	YFL023W	-0.12146	0.6790319	0.7809817	19.12	17.57	180.50	238.00
ORF-T	YBR295W	-0.121349	0.7280802	0.8186798	86.66	79.71	838.00	1076.75
ORF-T	YKL184W	-0.121339	0.6450968	0.7569094	156.92	144.29	1484.25	1927.75
ORF-T	YJL168C	-0.121261	0.5995936	0.7229445	65.76	60.52	624.25	794.25
ORF-T	YCR087C-A	-0.121002	0.7021825	0.7988277	15.37	14.16	145.50	188.50
ORF-T	YBL075C	-0.120921	0.6695939	0.7744219	55.60	51.15	514.00	669.25
ORF-T	YNL231C	-0.120744	0.6398772	0.7530075	49.49	45.49	445.50	592.00
ORF-T	YDL110C	-0.120633	0.7367863	0.8255038	49.17	45.23	433.00	556.50
ORF-T	YLR057W	-0.120598	0.6088268	0.7288863	59.28	54.51	551.50	732.00
SRT	SRT172	-0.120442	0.6676298	0.7732146	13.06	12.04	122.25	157.00
ORF-T	YGL029W	-0.120417	0.7323861	0.8218764	24.18	22.30	228.25	279.25
ORF-T	YLR422W	-0.120072	0.6638228	0.770806	99.95	91.94	903.25	1207.00
SUT	SUT376	-0.119977	0.8270436	0.8889474	0.84	0.81	8.25	10.00
ORF-T	YOR304W	-0.119968	0.6138238	0.7327807	65.07	59.89	609.75	798.75
ORF-T	YPL236C	-0.119906	0.6298413	0.7453726	25.67	23.59	230.50	308.25
ORF-T	YIL083C	-0.119806	0.6559297	0.7651122	28.36	26.06	257.50	348.50
ORF-T	YLR222C-A	-0.119672	0.7306685	0.8208624	7.07	6.55	65.50	82.25
ORF-T	YNL157W	-0.119652	0.6433976	0.7559153	21.51	19.80	197.00	261.00
ORF-T	YIR004W	-0.119521	0.6080548	0.728481	36.19	33.31	330.00	435.25
ORF-T	YMR085W	-0.119461	0.7487703	0.8342282	8.47	7.84	81.50	102.50
ORF-T	YKL010C	-0.119162	0.6495452	0.7598693	85.97	79.16	783.50	1022.50
ORF-T	YGR084C	-0.119016	0.6850061	0.785401	11.37	10.44	100.75	135.50
ORF-T	YLR018C	-0.118994	0.6442481	0.7564642	32.52	29.95	293.75	382.50
ORF-T	YNL331C	-0.118977	0.8032809	0.8714668	1.29	1.19	11.75	15.50
ORF-T	YPR003C	-0.118968	0.6070694	0.7279058	57.01	52.52	521.50	678.00

Class	Transcript name	RRP6 KO_vs_WT log2_fold _change	RRP6 KO_vs_WT p-value	RRP6 KO_vs_WT FDR	Ave Norm Reads in WT	Ave Norm Reads in RRP6	Average RAW read counts in WT	Average RAW read counts in RRP6
ORF-T	YIL051C	-0.118533	0.776636	0.8547316	168.83	155.45	1418.75	2060.75
ORF-T	YLL020C	-0.118517	0.7544507	0.8388886	4.41	4.08	38.75	50.00
ORF-T	YDR079W	-0.11814	0.7507012	0.8359177	4.30	3.95	38.75	51.25
ORF-T	YLL014W	-0.117858	0.66693	0.7729354	66.09	60.93	601.75	776.25
ORF-T	YDR001C	-0.117477	0.6911943	0.7908235	146.99	135.49	1294.00	1700.25
ORF-T	YAL056W	-0.117444	0.6325213	0.7475791	144.41	133.17	1360.50	1734.75
ORF-T	YER101C	-0.117405	0.7334862	0.8226532	6.71	6.17	60.00	80.50
ORF-T	YGR113W	-0.117095	0.6340373	0.7485812	23.45	21.61	213.00	281.00
ORF-T	YER133W	-0.11687	0.6522337	0.7618585	327.40	301.94	2971.00	3838.00
ORF-T	YGR182C	-0.116864	0.7380058	0.8264203	18.34	16.89	157.50	209.50
NUT	NUT0047	-0.116722	0.6416416	0.7546436	29.11	26.88	265.75	344.25
ORF-T	YOR039W	-0.116503	0.6042152	0.7258644	59.36	54.75	536.50	705.75
ORF-T	YLR336C	-0.11644	0.6651996	0.7716594	93.31	86.14	901.00	1134.00
NUT	NUT0838	-0.11632	0.7708147	0.8506898	28.95	26.69	257.00	345.50
ORF-T	YMR091C	-0.116221	0.622075	0.7387114	89.95	83.05	838.75	1060.50
ORF-T	YOR118W	-0.11587	0.6393198	0.7524394	67.70	62.53	633.00	800.50
ORF-T	YCR017C	-0.115856	0.6431449	0.7559153	132.55	122.32	1191.75	1559.75
ORF-T	YBR028C	-0.115838	0.6261	0.7420772	37.03	34.22	347.00	445.00
ORF-T	YIL145C	-0.115797	0.6279792	0.7436658	124.70	115.13	1167.00	1497.25
ORF-T	YGR108W	-0.115484	0.7511578	0.8363338	112.96	104.24	1067.00	1501.00
ORF-T	YGR048W	-0.115228	0.6567382	0.7653471	82.87	76.54	753.25	974.50
ORF-T	YCR035C	-0.115227	0.6168067	0.7349497	61.19	56.50	564.25	743.00
ORF-T	YGL013C	-0.114966	0.611784	0.7307782	44.01	40.60	394.50	530.75
ORF-T	YOR217W	-0.114959	0.6556994	0.7650119	174.17	160.87	1660.25	2126.00
ORF-T	YDR282C	-0.114732	0.6526761	0.762287	23.22	21.46	212.50	274.00
ORF-T	YGR133W	-0.114685	0.7026189	0.7991439	5.72	5.25	51.50	69.75
ORF-T	YKL021C	-0.11468	0.6727968	0.7768789	94.42	87.28	900.75	1124.50
ORF-T	YMR042W	-0.114662	0.7261145	0.8172942	7.00	6.46	65.25	87.00
ORF-T	YLR097C	-0.114495	0.6636869	0.7707727	22.12	20.46	203.25	261.25

Class	Transcript name	RRP6 KO_vs_WT log2_fold_change	RRP6 KO_vs_WT p-value	RRP6 KO_vs_WT FDR	Ave Norm Reads in WT	Ave Norm Reads in RRP6	Average RAW read counts in WT	Average RAW read counts in RRP6
ORF-T	YHR060W	-0.114428	0.6763262	0.779234	11.10	10.24	102.50	135.50
ORF-T	YKL203C	-0.114374	0.6273443	0.7432025	70.37	64.97	636.75	857.50
ORF-T	YPR010C	-0.114062	0.6794947	0.7812136	812.26	750.57	7665.50	9718.50
ORF-T	YPL034W	-0.114021	0.6556146	0.7650101	16.56	15.32	150.75	196.25
ORF-T	YGL010W	-0.113902	0.7080024	0.8029416	14.06	12.94	122.50	170.50
ORF-T	YOR004W	-0.1139	0.6771767	0.7796887	47.32	43.81	448.50	556.00
ORF-T	YBR141C	-0.113869	0.7092873	0.8035601	17.46	16.16	171.00	222.50
SUT	SUT572	-0.113722	0.7251722	0.816507	7.18	6.64	65.25	86.75
ORF-T	YKL181W	-0.113676	0.6450035	0.7569094	86.43	79.95	795.00	1001.25
ORF-T	YBL064C	-0.113646	0.8040157	0.8720002	33.26	30.71	277.75	386.25
ORF-T	YJR087W	-0.113547	0.813077	0.8792567	1.58	1.44	13.75	18.50
ORF-T	YBR201W	-0.113441	0.6801839	0.7816495	29.86	27.61	269.50	351.50
ORF-T	YGR052W	-0.113435	0.8375875	0.8952378	5.62	5.18	44.25	61.00
ORF-T	YHR124W	-0.113324	0.7865234	0.8622323	1.87	1.71	16.75	23.00
ORF-T	YNL259C	-0.113264	0.6881019	0.7884127	30.50	28.22	277.75	355.25
ORF-T	YNR024W	-0.112883	0.6706866	0.775043	13.56	12.57	125.75	162.00
ORF-T	YOR353C	-0.112629	0.6414927	0.7545565	67.09	62.10	633.25	815.25
ORF-T	YOR116C	-0.112584	0.6821599	0.7832954	150.14	138.91	1387.50	1786.25
ORF-T	YPR199C	-0.112437	0.6766937	0.7793376	13.67	12.61	123.25	167.00
ORF-T	YPL217C	-0.11242	0.6944438	0.7930658	109.10	101.01	1041.00	1286.50
ORF-T	YML046W	-0.112385	0.6364666	0.7503951	91.48	84.64	859.25	1128.75
ORF-T	YLR227C	-0.11237	0.6701671	0.7747295	19.86	18.41	184.25	239.50
ORF-T	YOR109W	-0.112287	0.674326	0.777932	179.01	165.66	1673.50	2116.25
ORF-T	YML115C	-0.112174	0.6446934	0.7566443	109.91	101.72	1030.00	1339.50
ORF-T	YLR276C	-0.111967	0.6630494	0.7702146	87.34	80.88	833.25	1049.25
ORF-T	YIL161W	-0.111944	0.6825548	0.7836597	10.08	9.34	94.50	123.75
ORF-T	YDR021W	-0.1118	0.6782591	0.7804863	22.74	21.05	211.75	280.00
ORF-T	YGL154C	-0.111718	0.7270748	0.8179694	5.17	4.72	46.00	64.00
ORF-T	YOL018C	-0.11129	0.6562319	0.7652877	29.49	27.35	277.25	353.75

Class	Transcript name	RRP6 KO_vs_WT log2_fold _change	RRP6 KO_vs_WT p-value	RRP6 KO_vs_WT FDR	Ave Norm Reads in WT	Ave Norm Reads in RRP6	Average RAW read counts in WT	Average RAW read counts in RRP6
ORF-T	YGR057C	-0.110962	0.6912395	0.7908235	15.35	14.23	143.00	188.50
ORF-T	YNL207W	-0.110837	0.6801693	0.7816495	107.30	99.43	1036.25	1311.00
ORF-T	YGR134W	-0.110785	0.6292559	0.7448481	86.87	80.47	800.25	1051.75
ORF-T	YLR389C	-0.110377	0.6788204	0.7808835	136.59	126.49	1256.50	1697.25
ORF-T	YPR164W	-0.110172	0.6281041	0.7436658	66.34	61.45	608.00	807.25
ORF-T	YML006C	-0.109379	0.6464061	0.7577725	35.82	33.19	324.25	428.50
ORF-T	YNL155W	-0.109337	0.6707458	0.775043	74.05	68.67	658.75	853.25
ORF-T	YFR048W	-0.109215	0.6309039	0.7460491	41.82	38.78	386.50	508.25
ORF-T	YFL031W	-0.108934	0.7003261	0.7971654	2082.76	1931.34	19959.50	25327.50
ORF-T	YER087W	-0.108591	0.7323416	0.8218764	17.17	15.95	160.00	205.25
ORF-T	YBR105C	-0.108577	0.7368465	0.8255038	18.29	16.95	161.50	216.50
ORF-T	YKL215C	-0.108478	0.691304	0.7908235	52.88	49.06	491.00	642.75
ORF-T	YOR155C	-0.108383	0.6792607	0.7810336	72.27	67.06	659.50	851.00
SUT	SUT754	-0.108336	0.8205526	0.8844661	2.88	2.67	28.25	37.75
ORF-T	YNL048W	-0.10803	0.6364196	0.7503951	52.29	48.48	471.75	639.00
ORF-T	YNL292W	-0.107851	0.7154154	0.8083791	16.46	15.24	152.50	207.75
ORF-T	YIR033W	-0.107569	0.7133432	0.807063	97.25	90.29	876.00	1135.00
ORF-T	YNL293W	-0.107533	0.6704323	0.7748584	49.64	46.13	462.25	589.50
ORF-T	YOR169C	-0.107275	0.8089672	0.8759396	1.50	1.39	13.50	18.00
ORF-T	YCL032W	-0.107195	0.7077757	0.8029416	25.37	23.54	232.50	310.50
ORF-T	YMR216C	-0.107132	0.6675388	0.7732146	143.96	133.68	1347.50	1757.00
NUT	NUT0429	-0.107006	0.790229	0.8643746	2.56	2.36	24.00	32.50
ORF-T	YOR172W	-0.106839	0.6695389	0.7744219	64.82	60.27	607.25	765.50
ORF-T	YPR077C	-0.106602	0.8506478	0.9040823	1.86	1.74	16.50	21.75
ORF-T	YDR529C	-0.106501	0.7744091	0.8533648	135.75	126.07	1193.75	1591.00
ORF-T	YKL022C	-0.106295	0.6445527	0.7565673	67.23	62.50	636.00	821.75
ORF-T	YNL232W	-0.106274	0.6874017	0.7877894	100.03	93.00	981.00	1236.25
ORF-T	YNR007C	-0.106034	0.688432	0.7887013	20.14	18.75	184.50	238.00
ORF-T	YGR033C	-0.106013	0.6733992	0.7773964	47.75	44.36	433.00	573.25

Class	Transcript name	RRP6 KO_vs_WT log2_fold _change	RRP6 KO_vs_WT p-value	RRP6 KO_vs_WT FDR	Ave Norm Reads in WT	Ave Norm Reads in RRP6	Average RAW read counts in WT	Average RAW read counts in RRP6
ORF-T	YDL236W	-0.105978	0.6671588	0.7729354	154.25	143.35	1426.00	1849.00
ORF-T	YBR089C-A	-0.1056	0.6770416	0.7796377	136.98	127.37	1256.50	1602.50
ORF-T	YJR051W	-0.105452	0.685095	0.7854136	92.59	86.05	834.75	1106.50
ORF-T	YIL009C-A	-0.105274	0.7497122	0.8351853	12.06	11.20	109.50	144.75
ORF-T	YDL087C	-0.105133	0.7084419	0.80323	8.88	8.23	82.25	111.75
AST	AS_YML012W	-0.105057	0.8427903	0.8992614	0.59	0.56	5.75	7.50
ORF-T	YOR380W	-0.104958	0.704685	0.800681	9.61	8.91	87.50	118.75
ORF-T	YPR025C	-0.104926	0.6912527	0.7908235	14.84	13.78	133.00	178.00
ORF-T	YDR005C	-0.104787	0.6743074	0.777932	27.27	25.35	245.50	327.00
ORF-T	YOL054W	-0.104406	0.6885395	0.7887348	31.57	29.37	294.25	389.25
ORF-T	YDR049W	-0.104377	0.684408	0.7848937	25.23	23.47	237.00	314.25
SUT	SUT052	-0.104164	0.8386169	0.8962073	1.23	1.17	11.75	14.75
ORF-T	YDL147W	-0.104107	0.6948267	0.7933234	167.31	155.68	1528.50	1995.50
ORF-T	YPL056C	-0.103789	0.7909323	0.8646119	2.81	2.58	26.00	36.00
ORF-T	YEL022W	-0.103686	0.6617321	0.7692338	63.46	59.08	583.50	762.00
ORF-T	YBR228W	-0.103587	0.720087	0.8122174	7.78	7.23	71.00	95.00
ORF-T	YCL063W	-0.103548	0.763473	0.8449146	16.04	14.88	149.50	211.75
ORF-T	YOR069W	-0.103532	0.6996661	0.7966583	101.72	94.69	947.25	1243.50
ORF-T	YNL021W	-0.103046	0.6650691	0.7716594	64.06	59.65	587.25	779.00
ORF-T	YEL041W	-0.103019	0.7799077	0.8571759	5.08	4.71	44.50	60.25
ORF-T	YER021W	-0.103	0.7079761	0.8029416	488.96	455.28	4541.50	5956.25
ORF-T	YGR278W	-0.102873	0.691746	0.7911497	16.23	15.11	150.00	199.00
ORF-T	YDL005C	-0.102408	0.6907354	0.7905318	24.62	22.92	221.00	294.50
ORF-T	YDR416W	-0.102371	0.6634554	0.7705925	49.10	45.82	466.00	593.00
ORF-T	YOR171C	-0.102359	0.6451444	0.7569094	80.36	74.85	731.75	975.00
ORF-T	YFR043C	-0.102148	0.7862323	0.8622323	3.02	2.77	28.00	39.25
ORF-T	YGR261C	-0.101904	0.7117944	0.8057628	34.26	31.89	312.75	422.25
ORF-T	YHR160C	-0.101893	0.8596298	0.9098426	1.11	1.02	10.25	14.25
ORF-T	YML112W	-0.101846	0.802367	0.8707565	3.65	3.33	31.25	45.75

Class	Transcript name	RRP6 KO_vs_WT log2_fold _change	RRP6 KO_vs_WT p-value	RRP6 KO_vs_WT FDR	Ave Norm Reads in WT	Ave Norm Reads in RRP6	Average RAW read counts in WT	Average RAW read counts in RRP6
ORF-T	YGR006W	-0.10179	0.722234	0.8142898	9.34	8.72	87.50	115.50
ORF-T	YBR231C	-0.101579	0.700704	0.7974156	21.29	19.86	195.75	260.50
ORF-T	YOL022C	-0.101326	0.6967166	0.7942329	153.34	143.02	1453.75	1830.00
ORF-T	YBR084W	-0.10114	0.7158175	0.8086829	435.91	406.43	4165.00	5353.75
ORF-T	YFL007W	-0.101126	0.7033433	0.7997873	248.20	231.39	2300.75	3096.75
ORF-T	YLR440C	-0.10098	0.660161	0.7682704	75.48	70.43	707.75	919.75
ORF-T	YML037C	-0.10097	0.7433657	0.8303167	5.27	4.88	47.50	64.75
ORF-T	YHR039C	-0.100763	0.6800782	0.7816495	86.07	80.23	782.25	1061.75
ORF-T	YPL139C	-0.100736	0.69404	0.792852	23.28	21.71	212.75	284.00
ORF-T	YBR014C	-0.100684	0.6726716	0.7768232	56.82	52.99	510.50	674.00
ORF-T	YGL037C	-0.10032	0.7969517	0.8684311	260.46	242.94	2258.50	3078.75
ORF-T	YDR236C	-0.100084	0.6719117	0.7762347	34.19	31.91	313.00	412.50
ORF-T	YBR259W	-0.099906	0.6974553	0.7949745	27.52	25.70	259.50	341.50
SUT	SUT459	-0.099776	0.8393897	0.8967316	1.08	0.98	9.50	13.25
ORF-T	YLR033W	-0.099663	0.6607447	0.7687723	72.80	67.98	677.50	879.00
ORF-T	YLR224W	-0.099609	0.6757096	0.778917	37.74	35.22	340.00	454.25
ORF-T	YGR210C	-0.09945	0.7056298	0.8014644	125.68	117.39	1186.50	1490.00
ORF-T	YMR113W	-0.099318	0.6826577	0.7836885	39.37	36.73	361.00	487.50
ORF-T	YGR271C-A	-0.099239	0.7072403	0.8025896	31.78	29.72	301.75	386.25
ORF-T	YDR399W	-0.099191	0.7393442	0.8270157	349.93	326.75	3271.75	4080.25
ORF-T	YML093W	-0.099069	0.6837633	0.7843646	47.74	44.59	449.00	590.25
ORF-T	YJR135C	-0.098962	0.7226838	0.8146147	10.94	10.27	106.00	136.00
ORF-T	YDR138W	-0.098912	0.7287288	0.8191396	21.59	20.14	200.75	273.00
ORF-T	YLR399C	-0.098805	0.6952822	0.7933944	242.27	226.27	2247.25	2923.75
ORF-T	YMR145C	-0.09879	0.7050239	0.8009758	320.90	299.68	2952.75	3878.75
ORF-T	YER077C	-0.098789	0.7384542	0.8264203	28.77	26.86	271.75	367.75
ORF-T	YKL145W-A	-0.098727	0.718317	0.8106968	146.28	136.60	1302.50	1726.25
ORF-T	YBR166C	-0.098474	0.6951674	0.7933943	74.47	69.57	704.50	930.00
ORF-T	YDR175C	-0.098335	0.7316854	0.8217121	20.19	18.84	181.00	241.50

Class	Transcript name	RRP6 KO_vs_WT log2_fold _change	RRP6 KO_vs_WT p-value	RRP6 KO_vs_WT FDR	Ave Norm Reads in WT	Ave Norm Reads in RRP6	Average RAW read counts in WT	Average RAW read counts in RRP6
ORF-T	YLL034C	-0.098246	0.7375377	0.8260946	208.57	194.91	2045.50	2592.75
ORF-T	YML075C	-0.097821	0.7591833	0.8419229	81.94	76.52	738.50	1022.00
ORF-T	YNL053W	-0.097747	0.7471004	0.8331041	25.04	23.36	233.75	323.75
ORF-T	YDR346C	-0.097687	0.7258538	0.817092	384.03	358.96	3621.00	4551.75
ORF-T	YNL100W	-0.097668	0.7403955	0.8277326	38.14	35.62	334.50	451.25
ORF-T	YDR110W	-0.097662	0.6820842	0.7832954	38.72	36.24	365.50	474.00
ORF-T	YBR131W	-0.097415	0.7116663	0.8057082	17.80	16.63	167.25	224.25
ORF-T	YCR009C	-0.097326	0.7186425	0.8109658	114.59	107.12	1019.00	1344.50
ORF-T	YPR041W	-0.097255	0.7229115	0.8146893	463.11	433.00	4362.50	5445.25
ORF-T	YBR066C	-0.096913	0.7271233	0.8179694	39.11	36.57	365.25	492.50
ORF-T	YFR020W	-0.096741	0.7153236	0.8083791	18.49	17.29	167.75	222.50
NUT	NUT0677	-0.096653	0.85316	0.9057023	1.29	1.14	10.50	16.00
ORF-T	YBR197C	-0.096632	0.7539764	0.8385461	12.93	12.02	114.50	163.25
ORF-T	YNL144C	-0.096547	0.7874142	0.8626056	68.48	64.04	618.50	818.25
ORF-T	YPR200C	-0.096484	0.842949	0.8992614	1.58	1.51	15.00	18.75
ORF-T	YNL282W	-0.096432	0.717457	0.8099	37.64	35.25	355.50	460.50
ORF-T	YNL035C	-0.096336	0.6997225	0.7966583	31.14	29.17	297.50	389.50
ORF-T	YER159C	-0.096312	0.7236473	0.8152451	22.13	20.69	205.50	278.25
ORF-T	YNR015W	-0.096302	0.7021618	0.7988277	26.88	25.12	244.50	331.25
ORF-T	YMR099C	-0.096196	0.7332288	0.822456	97.21	90.93	890.50	1186.50
ORF-T	YOR100C	-0.095867	0.7751616	0.8538545	7.43	6.96	70.25	94.25
ORF-T	YJR113C	-0.095668	0.7092981	0.8035601	56.48	52.95	534.75	666.50
ORF-T	YIL030C	-0.095617	0.6864264	0.7867642	107.82	100.85	977.25	1347.25
ORF-T	YBR165W	-0.095465	0.6989988	0.7962925	20.49	19.20	189.75	250.00
ORF-T	YOR229W	-0.095174	0.6992782	0.7964221	48.45	45.36	454.75	606.00
ORF-T	YPL011C	-0.095109	0.6927105	0.791744	30.11	28.18	269.25	358.75
ORF-T	YPL210C	-0.095079	0.6928131	0.791744	91.48	85.67	851.25	1121.75
ORF-T	YCL011C	-0.094921	0.7098707	0.8040364	181.56	170.05	1700.25	2206.50
ORF-T	YDR261C	-0.094886	0.7183237	0.8106968	33.76	31.60	315.00	422.50

Class	Transcript name	RRP6 KO_vs_WT log2_fold _change	RRP6 KO_vs_WT p-value	RRP6 KO_vs_WT FDR	Ave Norm Reads in WT	Ave Norm Reads in RRP6	Average RAW read counts in WT	Average RAW read counts in RRP6
ORF-T	YDR177W	-0.09458	0.6988583	0.7962925	85.51	80.10	765.25	1008.75
ORF-T	YGL145W	-0.094579	0.6767037	0.7793376	45.77	42.91	429.00	559.50
SUT	SUT038	-0.094073	0.8094837	0.8764048	1.78	1.64	15.50	21.00
CUT	CUT478	-0.093862	0.8505177	0.9040393	1.25	1.20	12.50	16.00
ORF-T	YOL142W	-0.093856	0.7615394	0.8435153	8.49	7.94	75.25	101.50
ORF-T	YGR273C	-0.093838	0.7985364	0.8692457	4.09	3.89	40.00	49.25
ORF-T	YGR111W	-0.093649	0.7041771	0.8001941	40.62	38.07	365.75	487.00
ORF-T	YMR316C-B	-0.093334	0.7687046	0.8489341	7.16	6.64	62.50	89.75
ORF-T	YER184C	-0.09331	0.739868	0.8273263	45.84	43.03	437.50	562.00
ORF-T	YIL075C	-0.093298	0.7452082	0.8315458	712.41	667.82	6580.75	8597.75
ORF-T	YMR206W	-0.093206	0.8646801	0.912552	2.34	2.15	18.50	27.00
ORF-T	YGL040C	-0.092736	0.7139473	0.8074963	87.90	82.42	809.00	1083.50
ORF-T	YLR424W	-0.092654	0.716874	0.8094231	15.80	14.83	146.50	194.75
ORF-T	YIL091C	-0.09255	0.7391421	0.8268814	68.79	64.54	666.25	881.00
ORF-T	YKL062W	-0.09234	0.8229642	0.8860017	24.42	22.86	201.50	288.00
ORF-T	YDR089W	-0.092216	0.7088348	0.8034046	71.81	67.41	651.75	849.50
ORF-T	YCL054W	-0.092064	0.7374438	0.8260813	127.31	119.51	1219.75	1543.25
ORF-T	YNL038W	-0.091991	0.7025496	0.7991439	25.10	23.55	228.75	305.75
ORF-T	YGL195W	-0.091454	0.7159455	0.808737	167.53	157.21	1536.25	2094.25
ORF-T	YOR338W	-0.091367	0.8031037	0.8713684	6.75	6.36	66.00	87.25
SUT	SUT033	-0.091208	0.840688	0.89758	3.67	3.45	34.00	46.50
NUT	NUT0721	-0.091054	0.8979307	0.935793	1.76	1.67	16.00	20.00
ORF-T	YOR201C	-0.090931	0.7085819	0.8032985	71.35	67.07	666.50	846.00
ORF-T	YGL088W	-0.090826	0.7742737	0.8533088	2025.73	1902.13	17892.50	24078.75
sn/snoRNA	SNR48	-0.090758	0.808693	0.8757367	2158.33	2026.72	18694.25	25621.50
ORF-T	YIL098C	-0.090725	0.8014979	0.8703008	11.85	11.11	104.50	140.50
ORF-T	YLR456W	-0.090654	0.7789918	0.856356	4.86	4.57	44.75	59.50
ORF-T	YML007W	-0.090457	0.7555178	0.8396769	110.19	103.49	1008.50	1343.50
ORF-T	YBL011W	-0.090311	0.7391148	0.8268814	203.03	190.76	1874.75	2425.25

Class	Transcript name	RRP6 KO_vs_WT log2_fold _change	RRP6 KO_vs_WT p-value	RRP6 KO_vs_WT FDR	Ave Norm Reads in WT	Ave Norm Reads in RRP6	Average RAW read counts in WT	Average RAW read counts in RRP6
ORF-T	YML019W	-0.090188	0.7241161	0.8155911	110.31	103.63	996.00	1319.50
CUT	CUT764	-0.090182	0.8128895	0.8792425	7.74	7.30	75.00	98.75
ORF-T	YGL223C	-0.090107	0.7541338	0.8386287	15.65	14.65	143.75	200.25
ORF-T	YJL133C-A	-0.0901	0.8224231	0.8858486	5.57	5.17	44.50	64.75
ORF-T	YFR023W	-0.089728	0.8596768	0.9098426	2.89	2.71	26.50	36.50
ORF-T	YDR482C	-0.089694	0.7502345	0.8354903	18.90	17.80	176.00	229.25
ORF-T	YGR218W	-0.089657	0.7272186	0.8179694	113.60	106.73	1042.25	1427.25
ORF-T	YER044C	-0.089646	0.7933734	0.8664981	13.35	12.50	117.00	163.75
ORF-T	YKR064W	-0.089505	0.743856	0.8305756	20.50	19.29	193.75	254.25
SUT	SUT491	-0.089499	0.8580541	0.9089376	1.50	1.38	13.75	19.50
ORF-T	YGL211W	-0.089405	0.7232993	0.8149442	74.68	70.24	697.00	911.25
ORF-T	YNL281W	-0.089311	0.6943033	0.7929952	52.55	49.43	483.50	635.00
ORF-T	YPR031W	-0.089112	0.7657363	0.8462771	22.80	21.41	214.50	295.50
ORF-T	YBL103C	-0.088751	0.7281574	0.8186798	20.34	19.11	188.75	255.75
ORF-T	YPR190C	-0.088619	0.7161838	0.8089082	67.21	63.26	647.25	842.75
ORF-T	YPL168W	-0.088267	0.7328698	0.8222362	21.94	20.63	198.50	266.25
ORF-T	YOR192C-C	-0.088107	0.8524334	0.9052167	1.62	1.54	14.50	18.75
ORF-T	YEL032W	-0.087994	0.7346073	0.8236476	195.75	184.24	1841.25	2367.00
ORF-T	YOR075W	-0.08792	0.7344226	0.8236119	12.83	12.07	116.50	156.50
ORF-T	YER054C	-0.087877	0.8741363	0.919244	12.07	11.35	100.75	137.25
ORF-T	YOR089C	-0.087735	0.7174285	0.8099	64.46	60.70	594.75	779.75
ORF-T	YCR005C	-0.087717	0.7952095	0.8672824	103.64	97.55	938.75	1245.75
ORF-T	YLR215C	-0.087201	0.7548983	0.8392012	10.62	10.04	101.00	131.00
ORF-T	YBR167C	-0.087095	0.7926322	0.86597	4.07	3.81	38.25	52.75
ORF-T	YLL036C	-0.087091	0.7013166	0.7980226	67.07	63.19	636.50	832.50
ORF-T	YPR071W	-0.087053	0.7650529	0.8458315	13.78	12.92	123.75	173.25
ORF-T	YFL028C	-0.087038	0.7129593	0.8068098	54.63	51.43	509.00	683.25
ORF-T	YMR002W	-0.087022	0.7447076	0.8311354	146.79	138.20	1343.50	1796.75
ORF-T	YPR046W	-0.087011	0.8286387	0.8899202	3.63	3.41	34.25	46.75

Class	Transcript name	RRP6 KO_vs_WT log2_fold _change	RRP6 KO_vs_WT p-value	RRP6 KO_vs_WT FDR	Ave Norm Reads in WT	Ave Norm Reads in RRP6	Average RAW read counts in WT	Average RAW read counts in RRP6
ORF-T	YKL115C	-0.086866	0.8346797	0.8933848	4.25	3.94	37.00	54.25
ORF-T	YNL294C	-0.086801	0.7068074	0.8022789	78.80	74.22	733.25	977.50
ORF-T	YKL074C	-0.086351	0.7290253	0.8193717	39.28	37.04	371.75	488.00
ORF-T	YOR251C	-0.085854	0.698036	0.7954566	67.01	63.17	621.25	824.00
ORF-T	YGL240W	-0.085839	0.7516099	0.8365325	13.47	12.66	119.50	164.50
ORF-T	YJR042W	-0.085831	0.7195555	0.8118145	139.19	131.20	1304.50	1701.25
ORF-T	YOR095C	-0.085777	0.7384577	0.8264203	112.26	105.82	1048.50	1370.50
ORF-T	YDR197W	-0.085677	0.7742683	0.8533088	9.36	8.76	82.50	115.50
ORF-T	YCR076C	-0.085662	0.7311912	0.8213582	24.86	23.45	227.25	300.50
SUT	SUT367	-0.0856	0.8165809	0.8815332	2.98	2.85	28.25	36.25
ORF-T	YMR283C	-0.085517	0.7482268	0.8340835	13.60	12.80	121.50	165.50
ORF-T	YPL016W	-0.08551	0.8056969	0.8732424	9.12	8.56	78.00	108.75
ORF-T	YOR211C	-0.085468	0.7238436	0.8153752	72.53	68.40	679.00	890.25
ORF-T	YNL127W	-0.085467	0.7227958	0.8146499	78.75	74.28	732.50	951.00
ORF-T	YNR048W	-0.085306	0.7162576	0.8089082	41.88	39.54	397.50	515.25
ORF-T	YAL011W	-0.085143	0.7408129	0.8280156	18.79	17.71	172.00	232.25
ORF-T	YJR098C	-0.085102	0.7905866	0.8644844	12.24	11.57	118.75	156.75
ORF-T	YDR031W	-0.085081	0.7899035	0.8642997	19.58	18.45	177.00	235.75
SUT	SUT635	-0.085053	0.8389497	0.896468	1.71	1.63	16.50	21.50
ORF-T	YER107C	-0.084955	0.7396856	0.8273059	123.11	116.13	1179.25	1517.75
ORF-T	YKL135C	-0.084323	0.7367562	0.8255038	57.42	54.13	522.50	716.50
ORF-T	YNL138W-A	-0.084231	0.7485286	0.8342282	15.58	14.68	140.50	190.00
ORF-T	YOL148C	-0.084032	0.7956714	0.8675984	11.58	10.91	100.25	136.00
ORF-T	YDR536W	-0.083991	0.8703856	0.9162479	1.14	1.08	10.25	13.75
ORF-T	YGL039W	-0.083932	0.764837	0.8458315	24.63	23.28	227.00	299.00
SUT	SUT184	-0.083797	0.8195729	0.8839115	2.52	2.38	23.25	31.50
ORF-T	YGR123C	-0.083767	0.755823	0.8396769	197.24	186.18	1853.75	2365.25
ORF-T	YJL160C	-0.083722	0.813826	0.879878	10.04	9.54	97.25	121.75
ORF-T	YAL067C	-0.083581	0.8872655	0.9278425	2.18	2.03	19.00	27.50

Class	Transcript name	RRP6 KO_vs_WT log2_fold _change	RRP6 KO_vs_WT p-value	RRP6 KO_vs_WT FDR	Ave Norm Reads in WT	Ave Norm Reads in RRP6	Average RAW read counts in WT	Average RAW read counts in RRP6
ORF-T	YHR073W	-0.083527	0.7453981	0.8316656	130.23	122.89	1213.00	1648.00
ORF-T	YHL010C	-0.083438	0.7478292	0.8338246	26.59	25.13	246.50	324.00
ORF-T	YPL098C	-0.083279	0.7326982	0.8221352	25.34	23.96	237.50	312.75
ORF-T	YMR056C	-0.083177	0.8004978	0.8699865	21.17	20.02	198.50	257.50
sn/snoRNA	SNR45	-0.083114	0.7954391	0.8674389	1316.56	1242.86	11735.00	16182.50
ORF-T	YBR211C	-0.082728	0.7318701	0.8217121	36.41	34.43	339.25	445.75
CUT	CUT673	-0.082198	0.845212	0.9007779	5.80	5.50	55.25	74.25
ORF-T	YAL047C	-0.082146	0.7646182	0.8457179	11.60	10.98	107.00	140.25
ORF-T	YHR169W	-0.081907	0.8003968	0.8699865	115.30	108.93	1086.00	1481.00
ORF-T	YBR290W	-0.081728	0.7363974	0.8252758	45.35	42.86	415.00	558.00
SUT	SUT326	-0.081584	0.8721766	0.9176783	1.24	1.17	11.50	15.25
ORF-T	YBL091C-A	-0.081484	0.8642035	0.912431	2.22	2.07	18.75	26.00
ORF-T	YBR247C	-0.08142	0.7613969	0.8434501	115.59	109.32	1123.25	1444.50
ORF-T	YDL204W	-0.081242	0.8582091	0.9089376	21.57	20.37	178.75	250.00
ORF-T	YLR047C	-0.081069	0.7669342	0.8471645	15.99	15.09	142.75	197.75
ORF-T	YGR169C-A	-0.081055	0.7321284	0.8218616	31.56	29.83	293.25	396.00
SUT	SUT654	-0.080968	0.8311553	0.8910741	3.01	2.91	29.25	36.75
ORF-T	YHR024C	-0.080936	0.7486515	0.8342282	56.37	53.33	526.50	688.25
NUT	NUT0161	-0.080779	0.8275843	0.8891256	5.49	5.14	47.50	68.75
ORF-T	YOR205C	-0.080387	0.7618658	0.8437842	11.69	11.06	107.75	145.25
ORF-T	YDR156W	-0.080374	0.7897807	0.8642624	5.44	5.10	48.50	67.25
ORF-T	YPL243W	-0.080344	0.7513704	0.8364781	272.93	258.16	2549.00	3413.00
ORF-T	YMR221C	-0.080266	0.7561775	0.8398735	19.01	17.95	171.00	235.00
ORF-T	YPR103W	-0.080162	0.7888203	0.8636768	238.18	225.33	2180.25	2864.00
ORF-T	YDR339C	-0.080024	0.7696591	0.8497742	18.23	17.18	162.00	231.25
ORF-T	YGL041W-A	-0.079743	0.786088	0.8621831	8.12	7.65	72.00	99.50
ORF-T	YML116W-A	-0.079392	0.7628267	0.8445701	31.02	29.40	287.75	375.75
sn/snoRNA	SNR69	-0.079138	0.7826497	0.8592531	210.31	199.00	1815.75	2663.75
ORF-T	YOR272W	-0.078833	0.7698341	0.8498092	162.97	154.37	1582.50	2041.50

Class	Transcript name	RRP6 KO_vs_WT log2_fold _change	RRP6 KO_vs_WT p-value	RRP6 KO_vs_WT FDR	Ave Norm Reads in WT	Ave Norm Reads in RRP6	Average RAW read counts in WT	Average RAW read counts in RRP6
ORF-T	YLR120C	-0.078513	0.7809729	0.8580661	157.95	149.61	1437.00	1913.00
SRT	SRT455	-0.078192	0.887096	0.9278425	0.95	0.90	9.50	13.00
ORF-T	YPL030W	-0.07794	0.7908704	0.8646119	8.36	7.93	78.00	105.00
SUT	SUT275	-0.077775	0.8582808	0.9089376	1.52	1.44	13.50	18.25
SUT	SUT039	-0.077704	0.8747459	0.9194484	1.49	1.40	13.25	18.50
ORF-T	YMR021C	-0.07755	0.7410789	0.8282212	53.17	50.40	490.25	654.50
SRT	SRT155	-0.077468	0.8772839	0.9208617	2.53	2.43	22.75	28.75
ORF-T	YDR325W	-0.077092	0.7525343	0.8371269	70.56	66.96	664.50	866.25
ORF-T	YBL025W	-0.077052	0.7849508	0.8612466	11.42	10.84	106.00	143.00
ORF-T	YOR233W	-0.076993	0.7487267	0.8342282	51.78	49.11	488.50	653.75
ORF-T	YPL207W	-0.0768	0.7782931	0.8558986	209.72	198.91	1985.50	2580.00
ORF-T	YLR178C	-0.076743	0.8419433	0.8987146	27.85	26.38	237.25	330.00
ORF-T	YGR125W	-0.076742	0.7435468	0.8303351	93.60	88.74	863.50	1170.75
ORF-T	YDR541C	-0.076648	0.7986475	0.8692457	6.40	6.02	57.00	80.25
ORF-T	YGL107C	-0.076616	0.7556526	0.8396769	47.64	45.19	439.75	591.25
ORF-T	YHR191C	-0.076232	0.801249	0.8701987	7.79	7.40	72.25	97.25
ORF-T	YBR002C	-0.076135	0.7910462	0.8646119	7.60	7.19	69.00	95.25
ORF-T	YLR222C	-0.07596	0.7727358	0.8521728	165.81	157.39	1568.50	2002.50
ORF-T	YHL042W	-0.075747	0.8468607	0.9019586	3.22	2.99	27.00	39.50
ORF-T	YNL193W	-0.075471	0.7769562	0.8548637	27.63	26.21	248.50	338.75
ORF-T	YPL179W	-0.07533	0.7690161	0.8491852	34.04	32.28	303.50	420.25
ORF-T	YOL162W	-0.075237	0.8545858	0.9064525	3.69	3.49	32.00	44.75
ORF-T	YGR202C	-0.075226	0.76527	0.8459749	37.26	35.35	343.50	469.00
ORF-T	YMR020W	-0.0751	0.7623757	0.8442041	62.78	59.63	578.75	768.00
ORF-T	YNL240C	-0.07509	0.7659848	0.8463012	97.18	92.31	926.25	1207.50
ORF-T	YGR013W	-0.075001	0.7624124	0.8442041	33.15	31.47	306.00	414.25
sn/snoRNA ET	SNR56-ET	-0.074985	0.8154066	0.8809564	415.36	394.31	3598.50	4960.00
ORF-T	YPR176C	-0.074547	0.7457537	0.8318784	42.72	40.60	394.50	522.25
ORF-T	YMR234W	-0.074466	0.7951824	0.8672824	17.69	16.75	158.00	223.25

Class	Transcript name	RRP6 KO_vs_WT log2_fold _change	RRP6 KO_vs_WT p-value	RRP6 KO_vs_WT FDR	Ave Norm Reads in WT	Ave Norm Reads in RRP6	Average RAW read counts in WT	Average RAW read counts in RRP6
ORF-T	YDL177C	-0.074101	0.8001664	0.8699599	7.00	6.65	64.75	87.75
ORF-T	YMR147W	-0.07409	0.8443617	0.9002477	2.46	2.33	21.75	30.00
ORF-T	YGR101W	-0.074	0.8009865	0.8701312	76.97	73.17	716.25	917.25
ORF-T	YNR020C	-0.07395	0.8041304	0.872001	9.52	9.04	89.50	121.75
ORF-T	YGR198W	-0.073771	0.751554	0.8365325	90.55	85.99	827.50	1148.75
NUT	NUT0275	-0.073764	0.8436698	0.8998585	2163.80	2055.93	18743.50	25991.00
ORF-T	YNL238W	-0.073708	0.7546618	0.8390309	95.82	91.09	886.25	1174.25
ORF-T	YBR151W	-0.073708	0.8347035	0.8933848	138.79	131.90	1257.75	1634.00
ORF-T	YPR151C	-0.073642	0.8614841	0.9109012	3.13	3.02	29.00	37.00
ORF-T	YKL091C	-0.073636	0.8608423	0.9105983	24.35	23.14	213.50	281.00
ORF-T	YPL144W	-0.073143	0.7932883	0.8664981	14.83	14.03	130.00	184.75
ORF-T	YKR052C	-0.073061	0.7786791	0.8561056	70.79	67.35	643.00	838.25
ORF-T	YGR247W	-0.073037	0.8376208	0.8952378	26.64	25.32	246.75	333.75
ORF-T	YHR189W	-0.072949	0.7986462	0.8692457	19.70	18.78	184.00	240.00
ORF-T	YGL201C	-0.072877	0.7729371	0.8522083	147.66	140.45	1396.00	1824.25
ORF-T	YOR244W	-0.072716	0.7519332	0.8366429	58.91	56.08	554.50	724.75
ORF-T	YPR117W	-0.072708	0.7818005	0.8586063	92.83	88.21	825.00	1164.50
ORF-T	YOR238W	-0.072645	0.7872432	0.862512	11.36	10.78	102.75	142.50
ORF-T	YGR277C	-0.072538	0.772657	0.8521728	20.50	19.52	189.75	254.25
ORF-T	YLR426W	-0.07237	0.7766665	0.8547316	22.72	21.64	209.75	279.75
ORF-T	YKL110C	-0.072348	0.7869982	0.8624309	25.94	24.61	229.25	321.00
ORF-T	YGL142C	-0.072033	0.7649694	0.8458315	32.38	30.80	293.25	395.50
ORF-T	YBR135W	-0.071464	0.8349379	0.893412	2.60	2.47	24.25	33.00
ORF-T	YMR180C	-0.07125	0.8164197	0.8814536	8.43	7.99	78.00	110.50
ORF-T	YNL097C-B	-0.071204	0.8703886	0.9162479	6.08	5.81	54.75	70.25
ORF-T	YLR384C	-0.070902	0.7865848	0.8622323	413.00	393.26	3889.75	5052.75
ORF-T	YLL008W	-0.070704	0.8189639	0.8835248	233.88	222.77	2280.00	2898.50
ORF-T	YBR258C	-0.070638	0.829222	0.890145	5.49	5.24	51.50	69.00
ORF-T	YGL184C	-0.070598	0.9212649	0.9517504	5.70	5.42	48.50	63.00

Class	Transcript name	RRP6 KO_vs_WT log2_fold _change	RRP6 KO_vs_WT p-value	RRP6 KO_vs_WT FDR	Ave Norm Reads in WT	Ave Norm Reads in RRP6	Average RAW read counts in WT	Average RAW read counts in RRP6
ORF-T	YBR220C	-0.070541	0.7747639	0.8536624	96.92	92.36	906.25	1180.25
ORF-T	YDL128W	-0.070494	0.7979176	0.8689202	130.76	124.51	1178.25	1606.25
ORF-T	YLR189C	-0.070183	0.7805655	0.857712	80.26	76.46	731.00	989.25
ORF-T	YDR421W	-0.069901	0.7948166	0.8672824	43.45	41.38	409.75	562.25
ORF-T	YDR306C	-0.069877	0.7868194	0.8623288	46.23	44.10	426.25	556.25
ORF-T	YMR066W	-0.069794	0.7607099	0.8428743	43.43	41.34	397.00	554.50
ORF-T	YMR052W	-0.069791	0.86149	0.9109012	3.54	3.35	30.25	42.25
ORF-T	YBR095C	-0.06976	0.755999	0.8397768	48.29	46.06	450.25	594.75
SUT	SUT642	-0.069736	0.8555111	0.907243	2.92	2.75	26.75	38.25
SUT	SUT515	-0.069606	0.8809159	0.9235009	2.20	2.06	17.75	25.50
ORF-T	YIL038C	-0.06956	0.7793576	0.8566647	164.78	157.08	1564.75	2050.25
ORF-T	YDL098C	-0.069478	0.83626	0.894352	2.80	2.66	25.50	35.25
ORF-T	YHR001W	-0.069404	0.776288	0.8545948	62.01	59.10	571.00	771.25
ORF-T	YDR059C	-0.069308	0.8551168	0.9069202	7.19	6.86	62.00	83.00
ORF-T	YBR196C-A	-0.069301	0.8040319	0.8720002	65.07	62.06	602.50	783.25
ORF-T	YOL072W	-0.069202	0.7986123	0.8692457	15.22	14.46	137.50	193.00
ORF-T	YDR351W	-0.069152	0.7659063	0.8463012	49.93	47.61	466.00	629.75
ORF-T	YJL065C	-0.069134	0.7989438	0.8693805	11.94	11.31	107.00	154.00
ORF-T	YPL022W	-0.069106	0.799658	0.8696884	177.93	169.66	1651.00	2157.75
ORF-T	YOL061W	-0.06902	0.8102461	0.8769418	437.01	416.68	4170.25	5273.25
ORF-T	YGL164C	-0.068964	0.7978822	0.8689202	24.97	23.79	223.75	305.00
SRT	SRT449	-0.068891	0.8990739	0.9365972	3.28	3.15	29.75	39.25
ORF-T	YJL010C	-0.068821	0.7946474	0.8672824	98.70	94.15	932.75	1241.25
ORF-T	YLR083C	-0.068771	0.8006223	0.8699865	333.52	318.05	3082.75	4018.75
ORF-T	YLR419W	-0.068229	0.7771528	0.8548935	57.26	54.61	532.25	726.25
ORF-T	YBR237W	-0.068109	0.7844956	0.8609047	27.68	26.38	250.00	344.75
ORF-T	YPR162C	-0.067814	0.7949466	0.8672824	17.68	16.82	160.75	226.00
ORF-T	YPL193W	-0.067616	0.7783726	0.8558986	42.98	41.01	401.00	550.00
ORF-T	YLR354C	-0.067492	0.8132565	0.8793565	220.65	210.54	1958.50	2680.00

Class	Transcript name	RRP6 KO_vs_WT log2_fold _change	RRP6 KO_vs_WT p-value	RRP6 KO_vs_WT FDR	Ave Norm Reads in WT	Ave Norm Reads in RRP6	Average RAW read counts in WT	Average RAW read counts in RRP6
ORF-T	YDR481C	-0.067066	0.8043267	0.8720386	179.58	171.47	1640.75	2149.75
ORF-T	YLR226W	-0.067019	0.7728948	0.8522083	46.57	44.53	443.75	580.25
ORF-T	YLR100W	-0.066933	0.8154644	0.8809564	166.81	159.23	1561.75	2176.25
ORF-T	YNL075W	-0.066901	0.7948426	0.8672824	71.90	68.68	687.75	922.25
ORF-T	YJR141W	-0.066612	0.7843874	0.8608797	29.98	28.66	278.50	371.25
ORF-T	YMR233W	-0.066599	0.8067645	0.8743056	11.13	10.62	99.50	135.50
ORF-T	YJL004C	-0.066531	0.7753979	0.8539879	48.46	46.31	443.00	590.50
ORF-T	YBR253W	-0.06643	0.8293249	0.890145	6.56	6.25	60.25	82.50
ORF-T	YDR026C	-0.066382	0.7969476	0.8684311	34.15	32.63	322.50	438.50
ORF-T	YMR176W	-0.066366	0.7756143	0.8540103	52.51	50.13	480.00	661.50
ORF-T	YMR187C	-0.066353	0.8070286	0.8744039	12.28	11.72	114.25	157.00
ORF-T	YBR116C	-0.066311	0.8820948	0.9242564	1.89	1.83	18.25	24.00
ORF-T	YGR163W	-0.066072	0.7850633	0.8612466	30.19	28.85	277.00	372.50
ORF-T	YNL011C	-0.066051	0.8190391	0.8835248	20.64	19.69	188.00	260.00
ORF-T	YMR218C	-0.065991	0.7994679	0.8696332	25.67	24.51	237.75	325.25
ORF-T	YGR166W	-0.065765	0.8107272	0.8772801	39.99	38.20	382.00	528.25
ORF-T	YOL010W	-0.065479	0.799521	0.8696332	60.23	57.63	587.50	767.00
ORF-T	YPL161C	-0.065378	0.8005456	0.8699865	29.51	28.21	275.25	372.50
SUT	SUT532	-0.065277	0.8849267	0.9263182	2.34	2.23	22.00	30.25
ORF-T	YPR144C	-0.064995	0.8156096	0.8809564	22.50	21.45	202.00	288.75
ORF-T	YER126C	-0.064985	0.7837689	0.8602945	93.36	89.29	874.25	1160.50
NUT	NUT1351	-0.064985	0.8475226	0.9024729	73.53	70.30	665.00	898.00
ORF-T	YMR256C	-0.064838	0.8518533	0.9048866	12.58	11.99	106.75	151.50
ORF-T	YIL103W	-0.064694	0.8042056	0.872001	59.92	57.36	578.75	756.50
ORF-T	YBR195C	-0.064549	0.8173758	0.8822969	10.24	9.81	95.75	128.75
ORF-T	YDR349C	-0.064377	0.782597	0.8592531	76.84	73.47	689.50	945.25
ORF-T	YKL138C-A	-0.064245	0.8226882	0.8859662	16.08	15.38	149.00	202.50
NUT	NUT0100	-0.064153	0.8292134	0.890145	7.19	6.85	65.00	90.50
ORF-T	YNL284C	-0.063886	0.7959262	0.8677825	61.01	58.39	553.50	736.25

Class	Transcript name	RRP6 KO_vs_WT log2_fold_change	RRP6 KO_vs_WT p-value	RRP6 KO_vs_WT FDR	Ave Norm Reads in WT	Ave Norm Reads in RRP6	Average RAW read counts in WT	Average RAW read counts in RRP6
ORF-T	YER029C	-0.063569	0.8144207	0.8801687	16.74	16.00	154.25	213.50
ORF-T	YPL167C	-0.063526	0.7967885	0.8684311	54.81	52.46	497.50	672.00
ORF-T	YLR143W	-0.063277	0.7897836	0.8642624	46.79	44.84	445.50	591.50
ORF-T	YNR026C	-0.063264	0.7871184	0.8624689	62.98	60.35	596.00	784.50
ORF-T	YDL117W	-0.062856	0.8054295	0.8730464	86.52	82.82	815.75	1123.50
ORF-T	YCR032W	-0.062824	0.7890932	0.8638818	70.87	67.86	648.50	877.00
SUT	SUT683	-0.062421	0.8361185	0.8942956	17.90	17.19	163.25	211.50
ORF-T	YDR060W	-0.062406	0.8156098	0.8809564	343.59	329.11	3263.00	4245.75
ORF-T	YHR150W	-0.062288	0.8312381	0.8910741	21.10	20.26	192.50	251.00
ORF-T	YOL063C	-0.062234	0.7909623	0.8646119	85.31	81.72	795.50	1090.25
ORF-T	YJL061W	-0.062227	0.7814721	0.8584275	67.37	64.53	623.25	848.25
SUT	SUT397	-0.062187	0.8584895	0.9090631	7.23	6.97	69.75	92.00
ORF-T	YGR147C	-0.062049	0.7935907	0.8665477	60.61	58.13	573.25	750.00
ORF-T	YLR153C	-0.061982	0.8085284	0.8756525	339.56	325.31	3140.75	4175.00
ORF-T	YGL163C	-0.061675	0.8413831	0.8982117	38.92	37.35	355.75	456.25
ORF-T	YGR127W	-0.061279	0.8607765	0.9105983	6.45	6.10	55.25	82.50
ORF-T	YJR056C	-0.06115	0.8239691	0.8866615	11.94	11.47	113.50	153.50
ORF-T	YKR085C	-0.060885	0.8520231	0.9049717	11.04	10.54	94.50	134.50
ORF-T	YHR077C	-0.060656	0.7901303	0.8643604	74.36	71.28	684.25	951.75
ORF-T	YOL093W	-0.060536	0.8160929	0.8813839	18.74	17.97	174.00	239.00
ORF-T	YML082W	-0.060448	0.8295422	0.8902834	57.90	55.58	563.50	742.75
ORF-T	YDR254W	-0.060317	0.8286742	0.8899202	17.96	17.23	163.50	221.75
AST	AS_YJL100W	-0.060053	0.8705446	0.9162479	6.00	5.78	58.75	79.50
ORF-T	YLR152C	-0.059888	0.8402722	0.8974057	14.04	13.44	124.50	173.50
NUT	NUT0206	-0.059848	0.8726825	0.9181148	877.24	841.53	7175.00	10814.75
CUT	CUT496	-0.059782	0.8746208	0.9194484	4.46	4.23	38.00	55.25
ORF-T	YBR074W	-0.059732	0.8230046	0.8860017	176.03	168.92	1638.25	2194.50
ORF-T	YMR154C	-0.059359	0.8330064	0.8924816	10.99	10.51	99.50	139.50
ORF-T	YMR158C-A	-0.059217	0.8756712	0.9198196	2.36	2.30	21.50	28.25

TABLE S1: Differential expression data for RRP6 RNA-Seq dataset Page 181

Class	Transcript name	RRP6 KO_vs_WT log2_fold_change	RRP6 KO_vs_WT p-value	RRP6 KO_vs_WT FDR	Ave Norm Reads in WT	Ave Norm Reads in RRP6	Average RAW read counts in WT	Average RAW read counts in RRP6
ORF-T	YFR001W	-0.059212	0.820188	0.8842912	24.89	23.91	233.25	317.50
ORF-T	YMR093W	-0.059075	0.8355687	0.8938024	76.53	73.49	725.00	977.75
ORF-T	YOL115W	-0.058978	0.8270631	0.8889474	45.56	43.80	441.00	582.00
ORF-T	YPL191C	-0.058733	0.8468067	0.9019586	11.70	11.24	104.25	140.25
ORF-T	YOR002W	-0.058536	0.8216615	0.8854066	73.38	70.41	662.25	931.50
ORF-T	YMR259C	-0.058234	0.802073	0.8705382	45.21	43.43	418.00	573.00
SUT	SUT071	-0.05818	0.8700417	0.9161968	2.56	2.47	24.00	32.75
ORF-T	YPR066W	-0.058088	0.8346286	0.8933848	11.82	11.29	104.25	149.00
ORF-T	YFR004W	-0.057596	0.8252145	0.8875281	110.19	105.89	1002.25	1346.00
ORF-T	YDL031W	-0.057534	0.8232373	0.8860631	59.62	57.37	560.25	730.25
ORF-T	YJR102C	-0.057311	0.86461	0.912552	6.25	5.98	56.75	79.50
ORF-T	YGR044C	-0.056907	0.830715	0.8909732	90.76	87.24	832.00	1145.00
ORF-T	YIL105C	-0.056887	0.8353208	0.893632	79.20	76.16	722.50	970.25
ORF-T	YOR210W	-0.05666	0.8439768	0.8999325	14.32	13.73	127.25	179.25
ORF-T	YDR218C	-0.056446	0.9014777	0.9385196	1.41	1.39	13.75	18.25
ORF-T	YGR128C	-0.056388	0.8099137	0.8767761	43.24	41.53	389.75	554.25
sn/snoRNA	SNR67	-0.056167	0.8561069	0.9076839	280.54	269.84	2550.25	3509.25
ORF-T	YDR516C	-0.056087	0.9232426	0.9531848	164.63	158.33	1361.50	1898.50
ORF-T	YKL075C	-0.056008	0.8174863	0.8823218	41.19	39.61	381.00	526.50
sn/snoRNA	SNR72	-0.05584	0.85936	0.9096983	404.05	388.64	3428.75	5067.50
ORF-T	YOL083W	-0.055646	0.875925	0.9199904	10.04	9.69	90.75	118.25
ORF-T	YDL125C	-0.055552	0.8429488	0.8992614	93.71	90.16	824.50	1135.25
ORF-T	YDL122W	-0.055527	0.8182904	0.8829061	119.92	115.45	1130.50	1497.50
ORF-T	YHR218W	-0.055505	0.8820149	0.9242564	2.38	2.30	21.50	29.00
ORF-T	YGR031W	-0.054953	0.8127162	0.8791493	83.78	80.68	774.00	1037.00
SRT	SRT394	-0.054851	0.9077	0.9423639	2.82	2.68	22.50	32.75
ORF-T	YPR179C	-0.054724	0.8249043	0.8875084	22.09	21.27	204.50	281.25
NUT	NUT0258	-0.054714	0.8623365	0.9113411	2027.74	1952.28	17912.25	24755.75
ORF-T	YPL221W	-0.054172	0.8540365	0.9059772	604.87	582.66	5766.50	7396.50

Class	Transcript name	RRP6 KO_vs_WT log2_fold _change	RRP6 KO_vs_WT p-value	RRP6 KO_vs_WT FDR	Ave Norm Reads in WT	Ave Norm Reads in RRP6	Average RAW read counts in WT	Average RAW read counts in RRP6
ORF-T	YHL030W	-0.05404	0.849795	0.9035568	194.91	187.72	1747.00	2439.00
ORF-T	YGL119W	-0.054028	0.8269235	0.8889474	35.15	33.87	327.00	448.00
SUT	SUT462	-0.053855	0.8708482	0.9164719	5.54	5.32	52.25	74.25
ORF-T	YFL025C	-0.053414	0.8340316	0.8933848	58.68	56.56	544.75	745.25
ORF-T	YMR129W	-0.053344	0.820701	0.8844661	85.45	82.35	770.25	1053.00
ORF-T	YGR145W	-0.053243	0.8222539	0.8858486	46.49	44.84	433.50	587.50
ORF-T	YKL175W	-0.053227	0.8206193	0.8844661	44.95	43.28	400.25	560.50
ORF-T	YPR189W	-0.052875	0.8318993	0.8915796	150.59	145.22	1419.75	1903.25
ORF-T	YOR022C	-0.052672	0.8424831	0.8991956	18.92	18.18	168.75	242.50
ORF-T	YGR239C	-0.052464	0.8747978	0.9194484	4.91	4.68	43.50	63.00
ORF-T	YEL015W	-0.05245	0.8272862	0.8890924	86.89	83.81	811.00	1098.75
ORF-T	YKL053C-A	-0.052066	0.8394167	0.8967316	31.80	30.68	282.00	383.25
ORF-T	YBR294W	-0.052046	0.9120629	0.9455374	1.33	1.28	11.50	16.00
SUT	SUT043	-0.05177	0.861848	0.9111712	7.13	6.85	64.25	90.50
ORF-T	YER123W	-0.051727	0.8364042	0.894377	30.16	29.08	279.00	387.50
ORF-T	YLL004W	-0.051576	0.8364607	0.894377	24.06	23.19	218.75	307.00
ORF-T	YBR256C	-0.051552	0.8483549	0.9026437	94.89	91.50	855.25	1224.75
ORF-T	YHR148W	-0.051502	0.8799771	0.9231886	19.47	18.75	180.75	260.25
ORF-T	YDL167C	-0.051437	0.8480426	0.9026437	156.12	150.68	1487.50	2000.50
sn/snoRNA	SNR11	-0.051427	0.8954834	0.9339607	25232.36	24348.74	215786.50	303862.50
ORF-T	YJR111C	-0.051342	0.8604049	0.9104221	11.32	10.86	103.50	149.75
SUT	SUT662	-0.051209	0.9017276	0.9386829	2.16	2.05	18.50	26.25
ORF-T	YCR047C	-0.051048	0.8749623	0.9194581	35.37	34.20	342.00	447.25
ORF-T	YDR444W	-0.050935	0.8345171	0.8933848	47.41	45.82	450.00	599.75
ORF-T	YKL009W	-0.050745	0.8349304	0.893412	145.49	140.43	1329.75	1872.50
ORF-T	YPL039W	-0.050667	0.8467176	0.9019586	24.33	23.54	228.50	302.75
ORF-T	YDR371W	-0.050472	0.8579188	0.9089376	45.92	44.37	414.25	549.50
ORF-T	YOR250C	-0.05033	0.8569059	0.9081491	43.02	41.53	403.50	565.50
ORF-T	YBR200W	-0.05026	0.8301191	0.890713	71.24	68.83	650.50	879.00

TABLE S1: Differential expression data for RRP6 RNA-Seq dataset Page 183

Class	Transcript name	RRP6 KO_vs_WT log2_fold _change	RRP6 KO_vs_WT p-value	RRP6 KO_vs_WT FDR	Ave Norm Reads in WT	Ave Norm Reads in RRP6	Average RAW read counts in WT	Average RAW read counts in RRP6
ORF-T	YGL238W	-0.050129	0.8406895	0.89758	242.85	234.56	2250.75	3083.50
ORF-T	YNL020C	-0.049882	0.863581	0.9122513	10.15	9.81	90.75	124.00
ORF-T	YDR161W	-0.049776	0.8509021	0.9041784	115.11	111.27	1095.50	1445.50
ORF-T	YER157W	-0.049287	0.8394631	0.8967316	70.99	68.59	653.00	909.50
ORF-T	YMR227C	-0.0491	0.8491791	0.9032829	15.01	14.52	139.25	190.75
ORF-T	YLL035W	-0.048905	0.864152	0.912431	11.77	11.39	110.25	151.25
ORF-T	YDL079C	-0.048534	0.912703	0.9459093	41.28	39.92	364.00	499.50
ORF-T	YLR407W	-0.04836	0.8593085	0.9096983	36.28	35.15	344.50	454.50
ORF-T	YPR138C	-0.048267	0.8449843	0.9007211	64.13	61.99	575.25	810.00
ORF-T	YER079W	-0.048232	0.9267612	0.9556662	2.00	1.90	16.25	24.25
NUT	NUT1464	-0.047929	0.9221729	0.9522982	1.69	1.58	14.50	22.00
ORF-T	YGR271W	-0.047577	0.8690017	0.9154389	42.47	41.02	379.25	557.50
ORF-T	YNL249C	-0.047501	0.8559559	0.9076193	16.03	15.48	145.25	205.25
ORF-T	YHR017W	-0.047399	0.8568426	0.9081491	55.48	53.68	498.25	689.25
SUT	SUT352	-0.04729	0.879147	0.9225097	6.32	6.08	58.75	83.75
ORF-T	YBR278W	-0.046959	0.8563045	0.907798	20.18	19.57	185.00	249.00
ORF-T	YFL003C	-0.046959	0.9007778	0.9379847	3.41	3.30	31.50	43.75
ORF-T	YDR289C	-0.046514	0.8493807	0.9033865	26.72	25.92	251.75	337.50
ORF-T	YLR451W	-0.046267	0.8500759	0.9037602	45.04	43.62	423.00	583.75
ORF-T	YLR174W	-0.046197	0.8910443	0.9307324	18.74	18.19	174.00	232.25
ORF-T	YNL274C	-0.046005	0.9200539	0.9510839	6.09	5.91	53.75	71.50
ORF-T	YLR275W	-0.045762	0.8669804	0.9139275	11.81	11.43	105.50	146.75
ORF-T	YDR514C	-0.045742	0.8626184	0.9114254	25.58	24.83	240.75	323.75
CUT	CUT887	-0.045474	0.9178804	0.9491861	1.37	1.34	12.75	17.25
ORF-T	YPR147C	-0.044991	0.8609398	0.9106059	34.44	33.39	315.25	430.50
ORF-T	YMR293C	-0.044702	0.8690006	0.9154389	19.43	18.85	178.00	243.00
ORF-T	YOL043C	-0.044537	0.8803231	0.9233898	10.08	9.78	91.50	126.25
ORF-T	YML059C	-0.04419	0.8653068	0.9127486	61.53	59.71	556.75	750.75
ORF-T	YPL150W	-0.043941	0.8481654	0.9026437	69.64	67.57	638.50	876.25

Class	Transcript name	RRP6 KO_vs_WT log2_fold _change	RRP6 KO_vs_WT p-value	RRP6 KO_vs_WT FDR	Ave Norm Reads in WT	Ave Norm Reads in RRP6	Average RAW read counts in WT	Average RAW read counts in RRP6
ORF-T	YDR350C	-0.0434	0.8540479	0.9059772	62.65	60.79	589.25	822.75
ORF-T	YKL173W	-0.043122	0.852756	0.9053687	68.48	66.52	644.75	869.50
ORF-T	YGL256W	-0.042958	0.85811	0.9089376	68.17	66.19	646.75	886.00
ORF-T	YGL233W	-0.042778	0.8608122	0.9105983	87.73	85.17	813.25	1119.25
ORF-T	YOR148C	-0.042511	0.8964541	0.9345439	10.30	10.02	94.25	129.25
ORF-T	YPL138C	-0.042505	0.8654787	0.9127486	24.11	23.45	221.50	298.00
ORF-T	YNL295W	-0.042124	0.8770958	0.9208365	43.28	42.06	407.00	553.50
ORF-T	YLR198C	-0.042067	0.9178144	0.9491861	1.84	1.77	16.75	24.00
ORF-T	YDR257C	-0.041692	0.880758	0.9235009	12.62	12.25	115.75	161.75
ORF-T	YDR094W	-0.041665	0.8728164	0.9181599	22.32	21.70	201.50	275.50
SUT	SUT560	-0.041455	0.8908524	0.9307028	5.76	5.57	52.25	74.25
ORF-T	YDR398W	-0.041432	0.8858683	0.926862	184.67	179.50	1812.75	2400.25
NUT	NUT0945	-0.041273	0.9424079	0.9654864	168.33	163.57	1396.00	1966.25
NUT	NUT0505	-0.041217	0.8659417	0.9130235	46.54	45.20	426.75	603.50
SUT	SUT719	-0.041012	0.9203352	0.9511797	2.39	2.30	21.75	31.50
ORF-T	YBR017C	-0.040919	0.8732517	0.918522	183.71	178.58	1716.25	2366.75
ORF-T	YKL072W	-0.040787	0.8839316	0.9257001	24.47	23.79	232.00	324.00
ORF-T	YPL117C	-0.040679	0.8680238	0.9148361	121.55	118.26	1149.25	1515.50
ORF-T	YCR057C	-0.04053	0.8886384	0.9290855	269.61	262.20	2630.50	3499.50
NUT	NUT0736	-0.04	0.8816432	0.9240713	27.05	26.33	257.25	351.75
ORF-T	YNL299W	-0.039982	0.9019659	0.938834	26.92	26.24	263.25	349.25
ORF-T	YOR166C	-0.03976	0.8784931	0.9219195	16.88	16.47	158.50	213.25
ORF-T	YOR145C	-0.0394	0.885562	0.9266376	55.12	53.63	521.00	738.50
ORF-T	YJR010C-A	-0.039359	0.8890604	0.9293339	20.68	20.09	185.50	262.75
ORF-T	YDL112W	-0.039284	0.8842872	0.9258426	163.21	158.85	1566.75	2158.50
ORF-T	YMR065W	-0.038886	0.8843431	0.9258426	12.31	11.97	111.50	156.25
NUT	NUT0110	-0.038579	0.9215154	0.9518141	2.05	1.98	18.00	25.25
ORF-T	YHR210C	-0.038525	0.9143044	0.9467694	10.09	9.87	99.00	130.50
ORF-T	YIR032C	-0.038445	0.9192925	0.9503943	4.03	3.94	37.00	50.75

Class	Transcript name	RRP6 KO_vs_WT log2_fold _change	RRP6 KO_vs_WT p-value	RRP6 KO_vs_WT FDR	Ave Norm Reads in WT	Ave Norm Reads in RRP6	Average RAW read counts in WT	Average RAW read counts in RRP6
sn/snoRNA	SNR7-L, SNR7-S	-0.038192	0.903347	0.9398835	1635.33	1592.62	14544.75	19953.00
NUT	NUT0802	-0.037717	0.9324633	0.9590062	1.21	1.14	10.50	15.50
sn/snoRNA ET	SNR41-ET	-0.037651	0.8933488	0.93256	185.90	181.04	1605.50	2377.75
AST	AS_YJL219W	-0.037264	0.9339692	0.9601534	2.04	1.98	18.75	26.50
ORF-T	YOR296W	-0.037219	0.8700202	0.9161968	46.52	45.34	428.00	597.25
ORF-T	YJL110C	-0.037143	0.8793366	0.9226127	40.60	39.59	378.50	525.00
ORF-T	YGR215W	-0.037098	0.9099285	0.9440623	5.72	5.53	51.00	73.75
ORF-T	YKR086W	-0.036754	0.8821886	0.9242587	42.49	41.47	405.75	548.25
ORF-T	YBR298C	-0.036639	0.924537	0.9541581	48.68	47.47	435.75	594.50
ORF-T	YGR222W	-0.036538	0.8969034	0.934819	10.31	10.00	93.00	134.00
SUT	SUT049	-0.03643	0.9245418	0.9541581	2.06	2.01	18.50	25.75
ORF-T	YBL020W	-0.036303	0.8784135	0.9219195	95.26	92.92	885.00	1206.25
ORF-T	YDR132C	-0.035971	0.9032616	0.9398835	8.48	8.20	73.75	109.00
ORF-T	YOR306C	-0.035767	0.9355247	0.9611647	25.51	24.89	252.75	358.75
ORF-T	YOR138C	-0.03553	0.8862694	0.9271854	127.05	124.04	1200.00	1592.50
ORF-T	YGL246C	-0.035201	0.883518	0.9253631	42.40	41.42	399.75	544.50
ORF-T	YJR115W	-0.035011	0.9281586	0.9564226	6.28	6.10	53.25	77.00
ORF-T	YER113C	-0.034889	0.8968687	0.934819	117.88	115.13	1094.25	1440.00
ORF-T	YDL120W	-0.034602	0.9099847	0.9440623	8.77	8.54	77.75	109.25
ORF-T	YGR045C	-0.034524	0.8993767	0.9368159	34.33	33.50	312.75	438.00
AST	AS_YJL041W	-0.034277	0.9412525	0.9650829	0.95	0.92	9.00	12.75
ORF-T	YJR003C	-0.033905	0.9001112	0.9373873	67.97	66.41	654.00	903.00
ORF-T	YIL084C	-0.03377	0.8852263	0.9264786	94.33	92.17	864.50	1185.75
ORF-T	YML041C	-0.033486	0.9067927	0.9418166	9.84	9.59	86.50	122.00
ORF-T	YNL286W	-0.032879	0.8952149	0.9339279	21.69	21.26	206.50	277.50
ORF-T	YOR064C	-0.032174	0.9066914	0.9418084	11.23	10.95	101.25	144.50
ORF-T	YDR047W	-0.032073	0.9060989	0.9413869	25.14	24.60	228.25	314.00
ORF-T	YJL162C	-0.031901	0.9143611	0.9467694	9.07	8.84	83.00	119.25
ORF-T	YOR341W	-0.031709	0.9086796	0.9429994	1308.86	1280.47	12457.00	16586.00

Class	Transcript name	RRP6 KO_vs_WT log2_fold _change	RRP6 KO_vs_WT p-value	RRP6 KO_vs_WT FDR	Ave Norm Reads in WT	Ave Norm Reads in RRP6	Average RAW read counts in WT	Average RAW read counts in RRP6
ORF-T	YDR147W	-0.031589	0.8960211	0.9341891	32.07	31.39	300.25	418.25
ORF-T	YFL041W	-0.031256	0.9047283	0.940577	205.65	201.31	1924.75	2567.25
ORF-T	YDR035W	-0.031241	0.9051834	0.9406952	137.84	134.82	1257.25	1853.00
ORF-T	YKL206C	-0.031228	0.8942497	0.9332106	37.79	36.94	341.75	488.00
SUT	SUT432	-0.03054	0.9420242	0.9654814	1.36	1.27	12.00	18.00
ORF-T	YNL139C	-0.030294	0.9012748	0.9384053	130.90	128.18	1197.50	1674.75
ORF-T	YOR178C	-0.029572	0.9546291	0.9736456	15.78	15.43	125.75	186.50
ORF-T	YPR029C	-0.029265	0.8996084	0.9369605	78.37	76.79	725.75	1016.00
ORF-T	YDR410C	-0.029138	0.9074783	0.9423346	42.48	41.65	390.75	542.25
ORF-T	YMR100W	-0.029042	0.9029085	0.9396769	102.50	100.50	950.50	1304.50
ORF-T	YGL151W	-0.02889	0.9073491	0.9422974	66.37	65.04	615.50	863.00
NUT	NUT0607	-0.028842	0.9410567	0.9649802	25253.60	24753.72	215972.50	309020.25
ORF-T	YMR288W	-0.028823	0.905281	0.9406952	38.68	37.93	361.75	499.50
ORF-T	YJL077W-B	-0.028589	0.9293196	0.9569345	6.54	6.46	60.25	80.75
ORF-T	YDR034C	-0.028393	0.9043396	0.9404311	61.57	60.35	573.25	818.50
ORF-T	YDR538W	-0.028254	0.939902	0.9643846	5.49	5.34	49.50	74.00
ORF-T	YFL026W	-0.028121	0.9256129	0.954775	149.05	146.14	1319.50	1896.00
ORF-T	YPR178W	-0.027978	0.9205446	0.9512986	17.72	17.37	160.75	226.75
ORF-T	YGL114W	-0.027268	0.9113709	0.9451096	103.59	101.68	951.25	1315.50
ORF-T	YFL050C	-0.027054	0.9128332	0.945947	43.44	42.62	395.00	564.75
NUT	NUT0222	-0.0269	0.9393038	0.9639143	4.77	4.61	40.50	60.75
ORF-T	YGL171W	-0.026896	0.9104342	0.9444314	46.60	45.74	434.75	609.50
ORF-T	YGR276C	-0.026882	0.9120385	0.9455374	103.98	102.13	968.50	1290.25
ORF-T	YBR186W	-0.026504	0.9530072	0.9727831	1.93	1.89	18.50	26.25
ORF-T	YCL040W	-0.026189	0.9431874	0.9656921	403.59	396.32	3428.75	4863.50
AST	AS_YNR022C	-0.026068	0.9500616	0.9709559	1.69	1.64	15.50	22.25
ORF-T	YJL056C	-0.025756	0.9407795	0.9649802	6.57	6.36	54.75	83.75
ORF-T	YNL163C	-0.025536	0.9208898	0.9514604	107.78	105.94	1033.00	1412.75
ORF-T	YMR044W	-0.025218	0.9284761	0.9564654	15.22	14.98	140.00	191.75

Class	Transcript name	RRP6 KO_vs_WT log2_fold _change	RRP6 KO_vs_WT p-value	RRP6 KO_vs_WT FDR	Ave Norm Reads in WT	Ave Norm Reads in RRP6	Average RAW read counts in WT	Average RAW read counts in RRP6
ORF-T	YLR112W	-0.024976	0.9345924	0.9604984	15.94	15.63	149.50	218.75
ORF-T	YDR370C	-0.024621	0.9208189	0.9514604	20.23	19.91	188.00	261.25
ORF-T	YLR233C	-0.024536	0.9233151	0.9531848	17.49	17.21	160.25	224.50
ORF-T	YBR061C	-0.024108	0.9273159	0.9560426	12.36	12.13	111.50	158.25
ORF-T	YML062C	-0.023982	0.9337527	0.9600288	13.35	13.13	124.25	173.25
ORF-T	YPR093C	-0.023951	0.928556	0.9564654	26.59	26.18	241.50	332.50
SUT	SUT190	-0.023887	0.93472	0.9604984	7.37	7.23	68.00	97.25
ORF-T	YLR147C	-0.023785	0.9384146	0.963741	6.17	6.00	53.75	79.50
ORF-T	YEL008W	-0.02352	0.9616165	0.9767265	1.79	1.72	15.75	24.00
ORF-T	YEL039C	-0.023193	0.9706669	0.9826528	6.10	5.97	47.25	73.00
ORF-T	YIL027C	-0.023164	0.9304505	0.9577078	78.24	76.97	705.25	1003.00
ORF-T	YJL112W	-0.022904	0.9244081	0.9541581	52.37	51.55	477.25	666.75
ORF-T	YFL047W	-0.022875	0.9276246	0.9561462	36.69	36.13	341.50	479.00
ORF-T	YKL163W	-0.022612	0.9447757	0.9671219	15.68	15.43	136.25	190.75
ORF-T	YJL082W	-0.022239	0.9409612	0.9649802	65.69	64.67	593.50	830.75
ORF-T	YMR019W	-0.022018	0.9287693	0.9564654	34.91	34.30	317.50	472.25
ORF-T	YBR094W	-0.021993	0.9342781	0.9602753	60.40	59.51	568.00	781.50
ORF-T	YGR088W	-0.021974	0.9596393	0.9764887	24.00	23.61	201.50	285.75
ORF-T	YGL070C	-0.021778	0.9277007	0.9561462	34.59	34.12	328.00	451.75
ORF-T	YPL074W	-0.02176	0.9269372	0.95575	41.97	41.36	388.75	540.75
SUT	SUT840	-0.021072	0.9622066	0.9770307	3.90	3.86	34.75	48.25
ORF-T	YJR152W	-0.020677	0.9458033	0.967864	26.21	25.87	239.00	329.50
ORF-T	YGL054C	-0.020235	0.9407738	0.9649802	90.44	89.14	820.50	1194.25
ORF-T	YPR128C	-0.020194	0.942153	0.9654864	46.80	46.14	439.75	620.00
ORF-T	YMR263W	-0.020174	0.9387619	0.963866	14.95	14.77	141.00	194.50
ORF-T	YCR097W	-0.020076	0.9428359	0.9656263	15.36	15.17	139.25	193.25
ORF-T	YAR050W	-0.019594	0.960696	0.9765045	1.53	1.51	14.25	20.00
ORF-T	YDR126W	-0.019451	0.9526484	0.9725153	4.97	4.82	43.25	66.00
ORF-T	YCR072C	-0.019417	0.9507215	0.9712339	85.62	84.50	834.00	1159.50

TABLE S1: Differential expression data for RRP6 RNA-Seq dataset Page 188

Class	Transcript name	RRP6 KO_vs_WT log2_fold _change	RRP6 KO_vs_WT p-value	RRP6 KO_vs_WT FDR	Ave Norm Reads in WT	Ave Norm Reads in RRP6	Average RAW read counts in WT	Average RAW read counts in RRP6
ORF-T	YMR308C	-0.018774	0.9431118	0.9656921	532.00	525.15	4980.25	6892.50
ORF-T	YGR046W	-0.018625	0.9390977	0.9639136	51.71	51.09	483.75	662.00
ORF-T	YGR169C	-0.018287	0.9472456	0.9690306	16.46	16.30	152.50	207.50
ORF-T	YDR473C	-0.018123	0.9513432	0.9715763	8.62	8.52	79.00	111.50
ORF-T	YIL092W	-0.017954	0.9490921	0.9701481	22.21	21.96	209.75	293.25
NUT	NUT0440	-0.017896	0.9808004	0.9887425	0.87	0.86	8.25	11.50
ORF-T	YOR357C	-0.017852	0.9418441	0.9654424	28.53	28.17	258.75	365.75
ORF-T	YGR024C	-0.01748	0.9384109	0.963741	58.25	57.52	531.00	765.75
ORF-T	YFL030W	-0.017372	0.9508152	0.9712339	28.61	28.32	266.00	359.50
ORF-T	YBR055C	-0.017352	0.9418903	0.9654424	89.60	88.59	844.25	1151.00
ORF-T	YHL039W	-0.017004	0.9458846	0.967864	33.93	33.53	315.00	442.50
ORF-T	YBR242W	-0.016919	0.9510979	0.9714243	26.96	26.68	256.50	355.25
ORF-T	YNL234W	-0.016804	0.9583252	0.9758063	7.90	7.76	69.50	101.75
ORF-T	YPL229W	-0.016777	0.9649949	0.9789748	2.77	2.75	24.50	34.00
ORF-T	YGL194C	-0.016579	0.9489231	0.9701481	19.58	19.35	182.50	259.75
NUT	NUT0672	-0.016494	0.9661363	0.9797384	6.10	6.00	52.75	78.00
ORF-T	YOR386W	-0.016459	0.9704441	0.9826246	21.05	20.80	183.75	257.25
ORF-T	YBL095W	-0.016419	0.9491201	0.9701481	22.54	22.35	210.00	283.00
ORF-T	YNL073W	-0.01608	0.9558908	0.9741154	59.81	59.14	539.75	761.00
ORF-T	YBR030W	-0.015697	0.9502962	0.9709988	67.31	66.63	645.75	888.25
ORF-T	YFL002C	-0.015686	0.947409	0.9690306	103.58	102.50	962.00	1326.25
ORF-T	YGR251W	-0.015398	0.9620622	0.9770266	5.05	5.03	49.00	67.50
NUT	NUT0353	-0.01501	0.9577575	0.9753612	29.29	29.03	271.00	374.75
ORF-T	YMR262W	-0.01498	0.96043	0.9765045	40.73	40.31	365.00	512.25
NUT	NUT0658	-0.014724	0.9595931	0.9764887	59.85	59.23	540.00	762.25
ORF-T	YHR065C	-0.014705	0.9518072	0.9719518	114.77	113.67	1083.75	1478.25
ORF-T	YOR280C	-0.014634	0.9605995	0.9765045	11.04	10.95	102.00	139.50
ORF-T	YDR283C	-0.014105	0.9560049	0.9741154	109.55	108.50	1029.75	1460.00
ORF-T	YHR162W	-0.013944	0.9576204	0.97532	277.58	274.93	2550.25	3571.25

Class	Transcript name	RRP6 KO_vs_WT log2_fold _change	RRP6 KO_vs_WT p-value	RRP6 KO_vs_WT FDR	Ave Norm Reads in WT	Ave Norm Reads in RRP6	Average RAW read counts in WT	Average RAW read counts in RRP6
ORF-T	YLR312W-A	-0.013905	0.9545119	0.9736292	48.65	48.21	448.25	619.75
ORF-T	YPL001W	-0.013821	0.9552718	0.9739161	23.05	22.86	212.50	296.75
ORF-T	YHR167W	-0.013819	0.95881	0.9760388	19.77	19.63	185.25	253.75
SRT	SRT423	-0.013731	0.9543459	0.9736292	26.28	26.06	244.00	342.00
ORF-T	YBR241C	-0.013697	0.9607201	0.9765045	14.07	13.91	125.50	179.25
ORF-T	YHR088W	-0.013667	0.9583881	0.9758063	32.55	32.26	307.00	433.00
ORF-T	YLR318W	-0.013662	0.9621057	0.9770266	12.35	12.22	115.75	166.00
ORF-T	YOR377W	-0.013659	0.9564626	0.9743377	66.35	65.75	612.50	867.00
ORF-T	YNL218W	-0.013537	0.9556451	0.9739969	43.91	43.57	418.25	570.00
ORF-T	YOR006C	-0.013366	0.9614441	0.9767265	23.53	23.31	217.75	311.25
ORF-T	YPL097W	-0.013287	0.9600916	0.9765045	35.42	35.14	327.50	446.25
NUT	NUT0156	-0.013287	0.9714722	0.9832892	6.91	6.84	62.25	89.75
ORF-T	YGR254W	-0.013052	0.9714905	0.9832892	463.44	459.20	3875.25	5863.50
ORF-T	YPR186C	-0.012784	0.9623456	0.9770734	14.74	14.63	139.00	195.25
ORF-T	YOL122C	-0.012646	0.9613545	0.9767265	112.35	111.33	995.75	1433.50
ORF-T	YLL003W	-0.012628	0.9596029	0.9764887	24.67	24.45	224.75	319.75
ORF-T	YJL094C	-0.012029	0.959795	0.9765045	39.31	38.99	357.75	507.00
ORF-T	YIL119C	-0.011783	0.9752792	0.9853437	15.26	15.10	129.50	190.75
ORF-T	YLR023C	-0.011698	0.9605584	0.9765045	62.84	62.28	560.00	816.50
ORF-T	YOR173W	-0.011671	0.980425	0.9885618	31.46	31.19	258.50	373.00
ORF-T	YOR231W	-0.011645	0.9601199	0.9765045	34.49	34.23	314.25	440.50
ORF-T	YKL149C	-0.011535	0.9612944	0.9767265	28.91	28.70	269.25	379.25
ORF-T	YML017W	-0.011451	0.9654773	0.9793655	70.24	69.68	644.00	910.00
ORF-T	YER078C	-0.011448	0.9640487	0.9784229	50.22	49.83	462.25	647.50
ORF-T	YKL012W	-0.011292	0.9644283	0.9784983	43.63	43.35	412.00	557.25
SUT	SUT291	-0.010885	0.9820485	0.9893839	1.21	1.20	10.50	14.75
ORF-T	YBL039C	-0.010798	0.971972	0.983436	321.90	319.56	3008.75	3998.75
ORF-T	YBR251W	-0.010581	0.9675895	0.9807189	36.96	36.66	329.75	475.00
ORF-T	YOR385W	-0.01055	0.9661045	0.9797384	39.56	39.28	360.50	508.75

Class	Transcript name	RRP6 KO_vs_WT log2_fold_change	RRP6 KO_vs_WT p-value	RRP6 KO_vs_WT FDR	Ave Norm Reads in WT	Ave Norm Reads in RRP6	Average RAW read counts in WT	Average RAW read counts in RRP6
CUT	CUT757	-0.01049	0.9755028	0.9854355	2.41	2.42	22.50	31.50
ORF-T	YMR063W	-0.010322	0.9729466	0.9836445	6.94	6.84	62.50	92.50
ORF-T	YOL163W	-0.010318	0.9790804	0.9877106	5.81	5.75	50.50	74.50
ORF-T	YDL123W	-0.009854	0.9799525	0.9881841	2.64	2.56	22.25	34.00
ORF-T	YPR094W	-0.009245	0.973567	0.9839095	14.17	14.09	128.75	181.50
SUT	SUT569	-0.009181	0.9694617	0.9818305	26.20	26.06	243.25	342.00
ORF-T	YDR401W	-0.008804	0.9863272	0.9916374	0.77	0.78	7.75	11.00
ORF-T	YML121W	-0.008711	0.9724894	0.9836445	24.60	24.48	227.00	316.00
ORF-T	YDR137W	-0.008671	0.9694652	0.9818305	67.70	67.32	623.50	869.25
ORF-T	YNL308C	-0.008664	0.9728112	0.9836445	65.16	64.79	615.50	864.75
ORF-T	YBR287W	-0.008612	0.9726553	0.9836445	55.80	55.45	496.25	712.00
ORF-T	YOR361C	-0.008573	0.9732351	0.9836728	381.03	378.79	3564.75	5017.50
ORF-T	YJL139C	-0.00844	0.9730246	0.9836445	29.36	29.19	266.75	379.00
ORF-T	YDR448W	-0.00829	0.9721429	0.983456	30.52	30.39	283.75	392.75
ORF-T	YBR271W	-0.008257	0.9759685	0.9855436	73.70	73.35	710.00	965.75
SRT	SRT46	-0.008067	0.9865633	0.991776	1.26	1.21	11.25	17.00
ORF-T	YGR201C	-0.007932	0.9793206	0.9878432	9.82	9.72	87.75	130.00
ORF-T	YGL141W	-0.007767	0.9790329	0.9877106	82.50	82.07	754.25	1050.00
ORF-T	YBR111W-A	-0.007699	0.9822275	0.9893839	8.87	8.80	75.50	107.75
ORF-T	YGL140C	-0.007643	0.9751811	0.9853433	187.15	186.20	1747.00	2420.25
ORF-T	YLR223C	-0.007507	0.9760635	0.9855436	66.07	65.79	634.00	874.75
ORF-T	YDR235W	-0.007475	0.9761957	0.9855784	24.89	24.79	232.25	325.50
ORF-T	YHR040W	-0.007218	0.9829361	0.989642	21.47	21.42	209.25	283.25
ORF-T	YGL134W	-0.007188	0.9782322	0.987338	26.19	26.12	246.50	336.25
ORF-T	YOR190W	-0.007067	0.9850248	0.9909208	4.16	4.17	39.00	53.25
SUT	SUT638	-0.006362	0.9893267	0.9935392	0.83	0.87	8.50	11.50
ORF-T	YGL243W	-0.006174	0.9798647	0.9881841	28.72	28.65	269.50	373.50
ORF-T	YBR217W	-0.005989	0.9818072	0.9893839	16.88	16.86	154.00	211.25
ORF-T	YKR027W	-0.005946	0.9806552	0.9886949	52.96	52.77	502.00	707.25

TABLE S1: Differential expression data for RRP6 RNA-Seq dataset Page 191

Class	Transcript name	RRP6 KO_vs_WT log2_fold _change	RRP6 KO_vs_WT p-value	RRP6 KO_vs_WT FDR	Ave Norm Reads in WT	Ave Norm Reads in RRP6	Average RAW read counts in WT	Average RAW read counts in RRP6
ORF-T	YPR160W	-0.005872	0.9928096	0.9957066	220.85	219.93	1739.25	2617.75
ORF-T	YHR074W	-0.005665	0.9826452	0.9895141	162.41	161.79	1490.75	2074.00
ORF-T	YBL054W	-0.005541	0.9861539	0.9915621	13.36	13.30	123.00	175.25
AST	AS_YDR107C	-0.005471	0.989622	0.9935966	2.34	2.29	21.00	31.75
ORF-T	YOR249C	-0.005459	0.9824178	0.9893839	25.47	25.40	237.25	331.00
ORF-T	YER081W	-0.005356	0.9870573	0.9921736	119.07	118.63	1092.25	1581.25
AST	AS_YJL005W	-0.005326	0.9927039	0.9957066	0.78	0.79	8.00	11.25
ORF-T	YMR171C	-0.005242	0.9824006	0.9893839	86.96	86.68	800.50	1117.75
ORF-T	YDR515W	-0.005211	0.9855572	0.9912586	22.09	22.03	201.50	278.00
ORF-T	YLR002C	-0.005181	0.983851	0.9900363	86.34	86.10	836.75	1141.50
ORF-T	YHR194W	-0.005032	0.9829685	0.989642	36.00	35.85	329.00	472.25
SUT	SUT492	-0.004317	0.9900372	0.9936972	8.88	8.85	79.25	113.25
AST	AS_YDR270W	-0.004111	0.9941663	0.996439	1.06	1.05	9.25	13.25
ORF-T	YHR170W	-0.003654	0.9900512	0.9936972	333.56	332.77	3113.50	4198.50
ORF-T	YMR172W	-0.003328	0.9891601	0.9935392	42.97	42.96	414.00	560.00
ORF-T	YOL064C	-0.003192	0.9894011	0.9935392	65.04	64.84	589.75	866.25
ORF-T	YGL167C	-0.00237	0.9929418	0.9957066	110.09	109.96	990.00	1356.50
ORF-T	YOR360C	-0.00202	0.993679	0.9960496	157.22	157.02	1472.25	2045.50
ORF-T	YDL001W	-0.001669	0.9948723	0.9969484	14.03	13.99	125.25	179.50
ORF-T	YOR384W	-0.001344	0.9970451	0.9981654	5.64	5.68	54.50	74.75
ORF-T	YLR430W	-0.00128	0.996181	0.9978632	184.71	184.57	1736.00	2445.25
ORF-T	YMR220W	-0.001171	0.9963204	0.9979038	108.14	108.09	1027.50	1427.50
ORF-T	YNR058W	-0.001154	0.9970315	0.9981654	10.37	10.38	100.50	142.00
ORF-T	YBR097W	-0.000928	0.9975214	0.9983775	50.19	50.13	463.25	672.25
ORF-T	YDR291W	-0.000316	0.9988817	0.9994764	61.77	61.76	570.50	812.75
ORF-T	YNL333W	-0.000245	0.9994213	0.9997023	1.96	1.98	18.00	25.25
ORF-T	YJL109C	-0.000242	0.9992637	0.9997023	293.02	292.99	2726.50	3860.00
ORF-T	YAL048C	-0.000143	0.9995706	0.9997023	17.64	17.63	162.50	231.50
ORF-T	YDL030W	-9.80E-05	0.9997023	0.9997023	22.39	22.41	210.50	297.25

Class	Transcript name	RRP6 KO_vs_WT log2_fold _change	RRP6 KO_vs_WT p-value	RRP6 KO_vs_WT FDR	Ave Norm Reads in WT	Ave Norm Reads in RRP6	Average RAW read counts in WT	Average RAW read counts in RRP6
AST	AS_YJL099W	0.0002063	0.99966	0.9997023	1.10	1.10	10.50	15.25
ORF-T	YOL132W	0.0003339	0.9994419	0.9997023	2.14	2.19	20.50	27.00
ORF-T	YCL042W	0.0006663	0.9985358	0.9992294	265.49	265.60	2261.00	3267.50
ORF-T	YDR240C	0.0007214	0.9975855	0.9983775	36.22	36.25	338.00	480.75
ORF-T	YIR026C	0.0009598	0.9970765	0.9981654	84.24	84.38	800.75	1083.00
ORF-T	YGR104C	0.0010481	0.9968304	0.9981654	15.27	15.22	138.25	206.00
ORF-T	YIL099W	0.0012377	0.9970119	0.9981654	18.86	18.94	177.50	236.75
ORF-T	YCR099C	0.0013388	0.9972355	0.9982255	1.67	1.72	16.00	22.00
ORF-T	YOR246C	0.0013607	0.9961535	0.9978632	174.71	174.93	1646.75	2265.50
SUT	SUT280	0.0017423	0.9956287	0.9976073	12.69	12.72	120.00	170.75
ORF-T	YJL033W	0.0017662	0.9946043	0.9967789	97.45	97.64	919.50	1264.50
ORF-T	YEL017C-A	0.0019405	0.9957617	0.9976415	58.14	58.14	514.00	805.50
ORF-T	YGR096W	0.0024286	0.9933716	0.9958616	8.47	8.48	79.00	113.25
ORF-T	YDR295C	0.0024968	0.9927833	0.9957066	30.65	30.70	276.25	391.75
ORF-T	YPL075W	0.0025576	0.9932947	0.9958616	31.72	31.69	270.00	412.50
ORF-T	YHR128W	0.0028113	0.990322	0.9938702	103.75	103.96	945.00	1340.50
ORF-T	YDR211W	0.0029371	0.9904761	0.9939258	210.59	211.06	1954.25	2722.25
NUT	NUT1252	0.0029781	0.9900479	0.9936972	38.96	39.14	366.75	495.25
SUT	SUT183	0.0030003	0.9928442	0.9957066	4.28	4.23	37.75	57.50
ORF-T	YCR027C	0.0034051	0.9890785	0.9935392	30.76	30.85	283.25	402.25
ORF-T	YDR239C	0.0034289	0.9892915	0.9935392	21.60	21.64	200.50	289.25
ORF-T	YDL109C	0.0037725	0.9896554	0.9935966	12.08	12.09	111.75	164.25
ORF-T	YKL178C	0.0038238	0.9933927	0.9958616	2.44	2.40	20.75	32.00
ORF-T	YJL126W	0.0038262	0.9881442	0.9929693	23.52	23.64	223.00	307.25
NUT	NUT0283	0.0039009	0.9928003	0.9957066	24.37	24.41	204.50	295.75
ORF-T	YMR228W	0.0043231	0.9886932	0.9934219	17.05	17.12	161.25	227.00
ORF-T	YDL222C	0.0044453	0.9927463	0.9957066	2.10	2.12	18.75	25.75
ORF-T	YMR209C	0.0044709	0.9861342	0.9915621	48.93	49.07	451.50	651.25
ORF-T	YDL174C	0.0049549	0.9875478	0.9925644	84.04	84.37	748.25	1016.00

Class	Transcript name	RRP6 KO_vs_WT log2_fold_change	RRP6 KO_vs_WT p-value	RRP6 KO_vs_WT FDR	Ave Norm Reads in WT	Ave Norm Reads in RRP6	Average RAW read counts in WT	Average RAW read counts in RRP6
ORF-T	YOR154W	0.0049898	0.9837686	0.9900363	42.11	42.30	391.50	546.25
ORF-T	YLR036C	0.0050239	0.9852657	0.9910643	17.23	17.34	160.50	223.00
ORF-T	YHR049W	0.0051051	0.9845884	0.9906795	327.01	328.21	3015.75	4201.00
ORF-T	YOR059C	0.0056368	0.9819811	0.9893839	46.83	47.00	419.50	609.50
ORF-T	YKR099W	0.0058174	0.9832412	0.989719	43.62	43.79	413.50	599.00
ORF-T	YPL070W	0.0058933	0.9810543	0.9888996	27.61	27.75	254.75	359.50
ORF-T	YNL107W	0.0060146	0.981911	0.9893839	12.98	13.02	119.00	170.75
ORF-T	YGL144C	0.0060824	0.9822175	0.9893839	17.53	17.65	165.00	230.75
ORF-T	YER140W	0.0066997	0.9772152	0.986509	29.52	29.66	273.50	390.50
ORF-T	YPR122W	0.0069683	0.9779219	0.9871236	27.40	27.51	249.75	363.75
SUT	SUT045	0.0070844	0.9876429	0.9925644	1.08	1.06	9.25	13.75
SUT	SUT068	0.0071169	0.9847852	0.9907786	20.52	20.61	180.50	262.50
ORF-T	YDR332W	0.0071622	0.9784813	0.9873917	23.04	23.13	212.50	312.25
ORF-T	YER186C	0.0072889	0.9756632	0.9854355	60.98	61.36	568.75	780.50
ORF-T	YDR142C	0.0074056	0.9755839	0.9854355	33.39	33.63	313.00	430.75
ORF-T	YLR251W	0.0077846	0.9836274	0.9900088	29.53	29.67	256.00	366.75
ORF-T	YLR005W	0.0087912	0.971685	0.9833873	95.04	95.71	893.25	1207.75
AST	AS_YPR201W	0.0087937	0.9784786	0.9873917	2.94	2.97	27.25	39.00
ORF-T	YBR056W-A	0.0093664	0.970559	0.9826423	41.52	41.84	392.25	551.75
sn/snoRNA ET	snR55-ET	0.0095563	0.9799261	0.9881841	32.16	32.36	275.00	402.00
AST	AS_YOR231W	0.0098978	0.9892946	0.9935392	0.92	0.91	9.00	14.00
sn/snoRNA ET	snr18-ET	0.010113	0.974639	0.9848943	66.92	67.29	547.25	860.50
ORF-T	YDR093W	0.0102754	0.9686059	0.9812559	241.88	243.64	2204.50	3111.00
AST	AS_YCR021C	0.0105601	0.9860471	0.9915621	1.06	1.03	9.25	14.75
ORF-T	YPR112C	0.0108116	0.9681123	0.9810516	128.38	129.42	1229.25	1679.00
NUT	NUT0955	0.0109021	0.9812026	0.9889502	1.39	1.43	14.00	19.50
ORF-T	YGR260W	0.0111521	0.9668522	0.9802673	435.63	439.04	4004.50	5573.50
ORF-T	YKR098C	0.0111691	0.9727306	0.9836445	17.65	17.75	156.75	232.75
ORF-T	YOL138C	0.0112132	0.9633129	0.977957	32.19	32.42	296.50	435.00

Class	Transcript name	RRP6 KO_vs_WT log2_fold _change	RRP6 KO_vs_WT p-value	RRP6 KO_vs_WT FDR	Ave Norm Reads in WT	Ave Norm Reads in RRP6	Average RAW read counts in WT	Average RAW read counts in RRP6
ORF-T	YPR068C	0.0113461	0.9690492	0.9816064	7.72	7.74	70.25	105.00
ORF-T	YJR149W	0.0118981	0.9659656	0.9797384	15.44	15.61	149.50	212.00
ORF-T	YDL165W	0.0119851	0.9609148	0.976604	45.20	45.62	419.75	582.50
ORF-T	YBL060W	0.0120255	0.961506	0.9767265	35.16	35.49	331.75	467.50
ORF-T	YKR063C	0.0120684	0.9615778	0.9767265	28.12	28.35	263.75	383.50
ORF-T	YDR252W	0.0121313	0.9720257	0.983436	7.32	7.31	63.75	98.75
ORF-T	YGR042W	0.0127618	0.9641344	0.9784229	8.98	9.07	81.50	116.25
ORF-T	YLR434C	0.0130485	0.9759298	0.9855436	1.03	1.03	9.00	13.00
ORF-T	YCL016C	0.0131359	0.9596318	0.9764887	12.87	12.97	117.50	170.00
ORF-T	YOL094C	0.0135196	0.9663018	0.9798076	5.62	5.66	49.75	72.75
ORF-T	YDL036C	0.013609	0.9536853	0.9732782	26.36	26.65	247.00	348.75
ORF-T	YMR074C	0.0136506	0.9564298	0.9743377	104.79	105.78	949.50	1360.50
ORF-T	YOR093C	0.0137592	0.9535777	0.973267	78.46	79.22	733.50	1057.25
CUT	CUT624	0.0138325	0.9790911	0.9877106	0.89	0.94	8.75	11.75
ORF-T	YBR065C	0.0138466	0.9545026	0.9736292	34.55	34.90	321.75	462.00
ORF-T	YPR169W	0.0140496	0.9507126	0.9712339	57.13	57.66	522.25	764.50
CUT	CUT553	0.0141287	0.9831983	0.989719	0.82	0.83	7.50	10.50
ORF-T	YDR183W	0.0143037	0.9672134	0.9804362	4.74	4.71	40.00	61.50
ORF-T	YOR033C	0.0145461	0.9552761	0.9739161	67.65	68.42	634.50	862.50
ORF-T	YLR014C	0.0147245	0.9571495	0.974939	35.86	36.28	340.00	477.00
ORF-T	YLR306W	0.0152261	0.9555156	0.9739969	12.14	12.24	109.50	160.00
ORF-T	YGR003W	0.0152319	0.9469868	0.9688936	35.44	35.87	330.75	463.00
ORF-T	YDR075W	0.0154188	0.9540332	0.9735348	21.62	21.92	207.25	286.75
NUT	NUT0248	0.0158069	0.9678783	0.9809131	2.91	2.95	25.75	36.75
ORF-T	YOR241W	0.0158218	0.9501901	0.9709888	82.71	83.63	777.00	1118.50
ORF-T	YGL230C	0.0161005	0.9718218	0.9834271	1.79	1.85	17.25	23.50
ORF-T	YPL099C	0.0161769	0.9600609	0.9765045	6.47	6.63	63.25	84.75
ORF-T	YHL027W	0.0164958	0.9473227	0.9690306	157.27	159.15	1480.50	2050.25
SRT	SRT605	0.0167409	0.9731096	0.9836445	1.21	1.20	10.75	16.25

Class	Transcript name	RRP6 KO_vs_WT log2_fold _change	RRP6 KO_vs_WT p-value	RRP6 KO_vs_WT FDR	Ave Norm Reads in WT	Ave Norm Reads in RRP6	Average RAW read counts in WT	Average RAW read counts in RRP6
ORF-T	YOR339C	0.016981	0.9728369	0.9836445	2.92	2.96	28.00	40.25
ORF-T	YOR203W	0.0169826	0.9522803	0.972238	268.75	271.96	2490.25	3530.25
ORF-T	YOR179C	0.0170032	0.9603319	0.9765045	8.14	8.22	75.25	108.75
ORF-T	YEL012W	0.0170964	0.9684593	0.9812559	4.52	4.54	37.75	56.50
ORF-T	YGR143W	0.0175177	0.9451644	0.9674217	154.66	156.56	1424.50	2042.50
ORF-T	YNL050C	0.0177703	0.9542872	0.9736292	9.00	9.10	83.50	123.75
ORF-T	YGL096W	0.0184774	0.9585796	0.9759027	3.00	2.98	26.50	40.75
ORF-T	YNR063W	0.0186309	0.9641599	0.9784229	5.44	5.52	54.00	78.50
CUT	CUT729	0.0186856	0.9636758	0.978227	1.81	1.88	17.75	24.25
ORF-T	YGR008C	0.0187422	0.9670048	0.9803234	62.69	63.50	528.50	756.75
ORF-T	YHR004C	0.0189434	0.9427539	0.9656263	34.86	35.34	317.00	456.00
ORF-T	YNR049C	0.0195262	0.9430151	0.9656921	18.47	18.76	171.25	243.00
ORF-T	YOR291W	0.0196745	0.9323475	0.9590062	77.80	78.87	700.00	1010.00
ORF-T	YNL043C	0.0197051	0.9644139	0.9784983	0.95	0.97	9.00	12.75
ORF-T	YBR285W	0.0197711	0.9700435	0.9823176	1.69	1.71	14.25	20.75
ORF-T	YNL268W	0.0204434	0.9363167	0.9617823	132.46	134.32	1218.50	1801.75
sn/snoRNA	SNR82	0.0204844	0.9489406	0.9701481	163.77	166.03	1379.25	2158.25
ORF-T	YDL020C	0.0205135	0.9295146	0.9570119	66.31	67.23	594.25	877.50
ORF-T	YPL087W	0.0208239	0.9489555	0.9701481	43.37	43.99	380.75	545.25
ORF-T	YML032C	0.0212749	0.9362144	0.9617752	70.69	71.81	664.00	909.25
ORF-T	YGL044C	0.0214618	0.928767	0.9564654	33.69	34.26	318.00	443.75
ORF-T	YDR288W	0.0214663	0.9316257	0.9585627	40.99	41.65	376.50	526.75
ORF-T	YDL073W	0.0215818	0.9264037	0.9553953	48.43	49.19	458.00	658.00
ORF-T	YER005W	0.0219086	0.930088	0.9574323	74.78	75.99	699.25	978.25
ORF-T	YDR364C	0.0230362	0.9249508	0.9543849	35.43	36.01	324.25	469.50
ORF-T	YMR097C	0.0230369	0.9318765	0.9586863	19.41	19.71	172.75	253.25
SUT	SUT274	0.0230719	0.9555638	0.9739969	2.34	2.43	22.00	30.00
AST	AS_YLR278C	0.0231279	0.9414781	0.965216	13.12	13.35	124.50	180.00
ORF-T	YMR126C	0.0232762	0.9275994	0.9561462	19.94	20.27	183.75	265.50

Class	Transcript name	RRP6 KO_vs_WT log2_fold _change	RRP6 KO_vs_WT p-value	RRP6 KO_vs_WT FDR	Ave Norm Reads in WT	Ave Norm Reads in RRP6	Average RAW read counts in WT	Average RAW read counts in RRP6
ORF-T	YGR173W	0.0233556	0.9279443	0.9562994	63.80	64.88	600.00	853.75
NUT	NUT1013	0.0236518	0.9435978	0.9660143	12.95	13.13	112.00	165.00
ORF-T	YDR372C	0.0237203	0.9232897	0.9531848	109.36	111.25	1010.50	1397.00
ORF-T	YJL193W	0.0239092	0.9387571	0.963866	6.90	6.99	62.75	93.25
ORF-T	YKR024C	0.0245596	0.9409636	0.9649802	118.73	120.84	1176.00	1579.00
ORF-T	YDR072C	0.0246011	0.9179357	0.9491861	120.86	122.96	1134.00	1642.25
NUT	NUT0424	0.0247724	0.942412	0.9654864	4.17	4.22	37.50	56.00
ORF-T	YJR116W	0.0247795	0.9423711	0.9654864	4.17	4.22	37.50	56.00
ORF-T	YJR061W	0.0249418	0.931523	0.9585627	17.46	17.84	168.25	233.25
ORF-T	YIL096C	0.0253468	0.9286806	0.9564654	12.71	12.94	115.75	165.75
SUT	SUT141	0.0254135	0.9391562	0.9639136	11.00	11.19	100.50	146.50
ORF-T	YJR161C	0.025637	0.9522056	0.972238	1.53	1.54	13.75	20.25
SUT	SUT847	0.0259032	0.9316614	0.9585627	6.54	6.61	59.25	89.00
ORF-T	YBL068W	0.0264651	0.9111437	0.9450702	71.67	73.03	658.75	942.50
ORF-T	YLR082C	0.0264685	0.9170097	0.9487151	21.21	21.60	194.25	282.50
ORF-T	YHL006C	0.0265219	0.9325679	0.9590062	14.62	14.94	132.25	183.00
ORF-T	YLL012W	0.0266558	0.9409944	0.9649802	80.00	81.53	767.25	1090.75
ORF-T	YNL086W	0.0267038	0.934781	0.9604984	4.33	4.39	40.00	59.75
ORF-T	YMR035W	0.0267409	0.9251663	0.9545096	18.15	18.53	168.00	237.75
ORF-T	YML057C-A	0.027183	0.9685476	0.9812559	0.56	0.59	6.00	8.50
AST	AS_YKL206C	0.0276599	0.9388952	0.963866	3.80	3.91	36.00	50.50
ORF-T	YHR005C	0.0279259	0.9039681	0.9402387	48.82	49.84	456.50	643.25
ORF-T	YMR052C-A	0.0280492	0.9560512	0.9741154	1.89	1.93	16.50	23.50
ORF-T	YLR409C	0.0281365	0.9255201	0.954775	272.49	277.94	2676.00	3633.50
ORF-T	YBR125C	0.0283885	0.9164377	0.9484152	41.02	41.82	378.75	553.00
ORF-T	YKL037W	0.0285668	0.9285462	0.9564654	5.97	6.10	53.25	76.25
ORF-T	YMR077C	0.0286271	0.9152905	0.9474224	11.25	11.50	104.00	150.25
ORF-T	YGL169W	0.0290722	0.914311	0.9467694	19.80	20.25	186.25	265.75
ORF-T	YBR272C	0.0292009	0.898938	0.9365524	39.35	40.17	361.75	523.00

TABLE S1: Differential expression data for RRP6 RNA-Seq dataset Page 197

Class	Transcript name	RRP6 KO_vs_WT log2_fold_change	RRP6 KO_vs_WT p-value	RRP6 KO_vs_WT FDR	Ave Norm Reads in WT	Ave Norm Reads in RRP6	Average RAW read counts in WT	Average RAW read counts in RRP6
NUT	NUT0062	0.0292923	0.9602158	0.9765045	7.70	7.87	78.50	115.00
CUT	CUT046	0.0295214	0.939348	0.9639143	3.22	3.25	28.00	43.50
ORF-T	YBR299W	0.0296799	0.9453581	0.9675216	2.22	2.30	20.75	29.00
ORF-T	YPL157W	0.0299979	0.9148664	0.9471779	17.02	17.42	164.50	233.00
ORF-T	YIL104C	0.030029	0.9132656	0.9462017	29.55	30.22	283.50	401.75
ORF-T	YLR417W	0.0306566	0.9039602	0.9402387	57.22	58.54	545.00	755.50
ORF-T	YKL165C-A	0.0307665	0.9190314	0.9502218	9.46	9.71	89.00	123.75
ORF-T	YHR073W-A	0.0308322	0.9248769	0.9543849	10.70	10.91	99.25	148.00
ORF-T	YPR005C	0.0309242	0.9325343	0.9590062	5.29	5.41	49.75	72.00
ORF-T	YGR074W	0.0313797	0.9132667	0.9462017	11.12	11.33	100.75	150.25
ORF-T	YOR278W	0.0313922	0.9165729	0.9484578	9.93	10.11	88.75	132.75
AST	AS_YDR230W	0.0315202	0.9201667	0.951103	19.34	19.75	170.50	251.75
ORF-T	YBR194W	0.0316195	0.916837	0.9486337	6.63	6.72	57.75	87.25
ORF-T	YDR357C	0.0318611	0.9046432	0.940577	23.02	23.57	211.00	299.00
ORF-T	YML043C	0.0323262	0.9138424	0.9466036	19.59	20.06	186.75	268.25
ORF-T	YDR115W	0.0324756	0.904853	0.940577	24.89	25.50	226.25	317.00
SUT	SUT040	0.0324956	0.9389186	0.963866	1.46	1.56	15.00	20.25
ORF-T	YOR213C	0.0325141	0.8899408	0.9300615	47.79	48.94	448.75	636.00
ORF-T	YLR129W	0.0325368	0.9078803	0.9423639	215.11	220.08	2066.50	2909.00
ORF-T	YDR437W	0.0325974	0.9029621	0.9396769	18.26	18.66	164.50	242.50
ORF-T	YMR196W	0.0326263	0.9336303	0.9600008	102.64	104.99	903.50	1291.25
ORF-T	YLR326W	0.0327543	0.9065148	0.941722	9.72	9.97	89.75	127.75
ORF-T	YBR224W	0.0331432	0.9548633	0.9736921	1.09	1.10	9.00	13.75
ORF-T	YBL089W	0.033202	0.8984517	0.9361856	29.07	29.73	260.75	380.25
SRT	SRT21	0.0333955	0.9547211	0.9736456	7.62	7.81	77.75	114.25
ORF-T	YDR163W	0.033428	0.9136814	0.9465342	4.78	4.92	45.75	65.25
ORF-T	YER188W	0.0335225	0.9173614	0.9488843	4.38	4.44	38.25	57.75
AST	AS_YER064C	0.0335493	0.9482956	0.969839	6.55	6.73	63.00	90.50
ORF-T	YER173W	0.0335833	0.8923442	0.9318971	19.83	20.34	184.00	261.50

Class	Transcript name	RRP6 KO_vs_WT log2_fold _change	RRP6 KO_vs_WT p-value	RRP6 KO_vs_WT FDR	Ave Norm Reads in WT	Ave Norm Reads in RRP6	Average RAW read counts in WT	Average RAW read counts in RRP6
ORF-T	YOR289W	0.0341523	0.9295846	0.9570119	31.30	32.03	266.00	395.50
ORF-T	YLR298C	0.0342601	0.9099835	0.9440623	5.57	5.68	50.25	74.75
ORF-T	YER007W	0.0346601	0.8909236	0.9307028	16.76	17.20	155.25	222.25
ORF-T	YLR007W	0.0347178	0.9125847	0.9458838	37.07	38.03	350.25	484.00
ORF-T	YDR058C	0.035521	0.9143779	0.9467694	9.12	9.36	81.75	118.75
ORF-T	YNL325C	0.035588	0.8849811	0.9263182	36.63	37.62	341.75	477.00
NUT	NUT0805	0.0356459	0.9114628	0.9451096	1322.92	1356.01	11791.00	17637.50
ORF-T	YDR231C	0.0359508	0.9053399	0.9406952	31.93	32.72	283.00	415.25
CUT	CUT158	0.0360676	0.949175	0.9701481	1.57	1.60	15.00	22.25
ORF-T	YLR134W	0.0361035	0.9077159	0.9423639	11.25	11.50	102.25	153.25
ORF-T	YDR045C	0.0361767	0.8949568	0.9337553	35.72	36.62	322.75	472.75
ORF-T	YBR136W	0.0371863	0.8766353	0.9206405	33.14	33.99	302.50	445.00
AST	AS_YCR030C	0.0372776	0.9340918	0.9601817	3.80	3.92	36.00	52.75
ORF-T	YJL047C	0.0375923	0.8754694	0.9197992	25.90	26.59	237.75	346.00
ORF-T	YJL127C	0.0377199	0.8806869	0.9235009	21.23	21.83	196.75	282.50
ORF-T	YOR080W	0.0379183	0.8742116	0.919244	36.47	37.43	338.25	505.50
ORF-T	YLL028W	0.0380259	0.895486	0.9339607	14.92	15.25	130.25	197.50
ORF-T	YLR146C	0.038065	0.8748619	0.9194484	44.71	45.97	415.50	591.25
SUT	SUT132	0.0386361	0.9215107	0.9518141	3.90	3.98	36.00	55.25
ORF-T	YBR218C	0.0387262	0.8824683	0.9244557	531.93	546.38	4893.75	7308.00
AST	AS_YGL219C	0.0388718	0.9309866	0.9581617	1.47	1.51	14.00	20.75
ORF-T	YER158C	0.0389255	0.908519	0.9429298	7.03	7.17	59.50	91.00
ORF-T	YJL039C	0.0394281	0.8747966	0.9194484	260.17	267.41	2427.00	3505.00
ORF-T	YHL005C	0.039541	0.9427701	0.9656263	1.05	1.11	10.00	13.75
ORF-T	YBL014C	0.0400796	0.873422	0.9186053	71.57	73.62	678.00	984.25
ORF-T	YOR052C	0.0401119	0.8872064	0.9278425	132.96	136.73	1206.00	1722.25
ORF-T	YDR160W	0.0405134	0.8804435	0.9233898	64.08	65.96	592.50	838.25
ORF-T	YIL143C	0.0409808	0.8697767	0.916109	100.62	103.57	938.50	1344.00
SUT	SUT085	0.0412491	0.9078752	0.9423639	4.39	4.50	39.00	58.50

Class	Transcript name	RRP6 KO_vs_WT log2_fold _change	RRP6 KO_vs_WT p-value	RRP6 KO_vs_WT FDR	Ave Norm Reads in WT	Ave Norm Reads in RRP6	Average RAW read counts in WT	Average RAW read counts in RRP6
ORF-T	YOL154W	0.0413277	0.8891636	0.9293455	7.59	7.84	72.25	105.25
ORF-T	YNL140C	0.0416633	0.8655	0.9127486	22.02	22.67	201.25	294.75
ORF-T	YBR219C	0.0417439	0.8676256	0.914512	26.27	27.10	245.75	348.50
ORF-T	YGR126W	0.0417599	0.9124505	0.945842	1.99	2.02	18.00	27.25
ORF-T	YBR068C	0.0424228	0.8690497	0.9154389	345.93	356.28	3170.75	4573.75
ORF-T	YPL060W	0.0424446	0.8842894	0.9258426	13.46	13.82	121.00	184.75
ORF-T	YPR143W	0.0425402	0.8611238	0.910705	37.24	38.41	348.50	496.25
ORF-T	YGL111W	0.0426763	0.8619261	0.9111712	68.52	70.66	653.00	922.75
ORF-T	YDL006W	0.0430538	0.8497656	0.9035568	56.84	58.65	536.25	754.75
NUT	NUT0980	0.0430777	0.9258401	0.9549117	2.30	2.33	18.50	28.50
ORF-T	YBR273C	0.0432953	0.8773025	0.9208617	23.84	24.57	217.25	315.75
ORF-T	YGL241W	0.0435837	0.8656995	0.9128635	46.37	47.80	430.00	626.25
ORF-T	YOL164W	0.0438404	0.8900707	0.9301008	8.64	8.92	80.50	116.50
ORF-T	YGL007C-A	0.0438423	0.893786	0.9328233	9.30	9.58	86.25	128.50
ORF-T	YLR452C	0.0443433	0.8803781	0.9233898	45.98	47.37	407.50	617.00
NUT	NUT1443	0.0444149	0.9052303	0.9406952	1.73	1.74	15.25	23.25
SUT	SUT219	0.0446034	0.8957276	0.9339796	5.30	5.49	47.50	68.75
ORF-T	YKR087C	0.0447809	0.8478602	0.9025464	50.21	51.89	479.50	673.75
ORF-T	YOL042W	0.0450935	0.8477999	0.9025464	38.19	39.44	354.25	508.25
ORF-T	YNL329C	0.0454472	0.8623579	0.9113411	32.55	33.56	297.75	447.00
ORF-T	YBL004W	0.045867	0.8643082	0.9124459	260.97	269.42	2428.75	3568.25
ORF-T	YPL183C	0.0473779	0.864113	0.912431	152.67	157.81	1472.25	2123.75
ORF-T	YIR018W	0.0477566	0.8648021	0.9125852	10.35	10.70	95.75	140.75
ORF-T	YMR080C	0.0479467	0.8504567	0.9040393	282.14	291.71	2646.25	3839.75
ORF-T	YOR204W	0.0479882	0.8650835	0.9127486	472.05	488.05	4372.50	6295.75
ORF-T	YOR034C	0.0480466	0.8478445	0.9025464	73.77	76.34	691.75	984.75
ORF-T	YMR223W	0.0480724	0.8489569	0.9031419	47.05	48.62	439.25	660.50
ORF-T	YJL099W	0.0480884	0.8483468	0.9026437	38.96	40.28	366.50	543.25
ORF-T	YJL199C	0.0481087	0.8943512	0.9332199	5.87	6.09	53.75	78.00

Class	Transcript name	RRP6 KO_vs_WT log2_fold _change	RRP6 KO_vs_WT p-value	RRP6 KO_vs_WT FDR	Ave Norm Reads in WT	Ave Norm Reads in RRP6	Average RAW read counts in WT	Average RAW read counts in RRP6
ORF-T	YKL106W	0.0482479	0.8639936	0.912431	57.71	59.68	540.50	799.50
SRT	SRT54	0.0483509	0.9114507	0.9451096	0.97	0.97	8.75	13.25
ORF-T	YDR198C	0.0486254	0.8452163	0.9007779	34.56	35.81	335.75	478.25
ORF-T	YHR032W	0.0489465	0.8639546	0.912431	178.84	185.06	1690.75	2433.50
ORF-T	YPL181W	0.0490669	0.8525976	0.9052958	50.62	52.45	481.25	674.75
SUT	SUT794	0.0490712	0.8903541	0.9303006	2.90	2.98	26.50	40.25
ORF-T	YLR231C	0.0497193	0.8323025	0.8919169	66.71	69.12	623.25	882.50
ORF-T	YKR038C	0.0499281	0.8313165	0.8910741	70.19	72.69	660.50	970.00
ORF-T	YGL052W	0.0500708	0.9172695	0.9488843	1.01	1.06	9.50	13.75
ORF-T	YNL148C	0.0501065	0.8645266	0.912552	11.37	11.78	105.50	153.75
ORF-T	YEL045C	0.0501763	0.8539108	0.9059772	134.43	139.13	1199.25	1851.00
SUT	SUT574	0.0504067	0.8565435	0.9079559	22.71	23.54	214.25	311.25
ORF-T	YOR077W	0.0507602	0.8652776	0.9127486	7.94	8.19	71.75	109.25
ORF-T	YNR027W	0.0507816	0.8351414	0.8935349	47.48	49.24	452.00	645.00
ORF-T	YJL100W	0.0508566	0.8534338	0.9057911	13.74	14.27	126.50	182.50
ORF-T	YMR185W	0.0509788	0.8407024	0.89758	44.06	45.65	404.25	598.25
ORF-T	YFR055W	0.0515533	0.8812658	0.9237718	79.42	82.27	735.25	1140.00
NUT	NUT0951	0.0515622	0.8579881	0.9089376	12.17	12.62	111.50	163.75
SUT	SUT658	0.0523956	0.8929331	0.9322225	3.57	3.67	29.75	45.25
ORF-T	YPR109W	0.0525233	0.8230948	0.8860043	38.72	40.19	354.75	512.25
ORF-T	YGR174W-A	0.0525421	0.8984931	0.9361856	2.19	2.28	19.00	27.25
ORF-T	YJL090C	0.0525714	0.8588097	0.9093067	14.57	15.11	132.00	197.50
SUT	SUT501	0.0526428	0.8928842	0.9322225	3.10	3.22	28.75	42.75
ORF-T	YNL115C	0.0527796	0.8685789	0.9152768	49.06	50.89	443.25	637.25
ORF-T	YHR072W	0.0527964	0.8535132	0.9057911	165.21	171.43	1583.50	2246.00
SUT	SUT823	0.0528402	0.8919053	0.9315353	2.19	2.24	19.50	29.75
ORF-T	YOR299W	0.0529447	0.8194916	0.8839115	79.53	82.55	739.25	1070.00
ORF-T	YDR496C	0.0530138	0.8312834	0.8910741	58.78	61.03	556.50	802.75
ORF-T	YKR030W	0.0532127	0.8300119	0.8906927	25.73	26.68	231.25	342.75

Class	Transcript name	RRP6 KO_vs_WT log2_fold _change	RRP6 KO_vs_WT p-value	RRP6 KO_vs_WT FDR	Ave Norm Reads in WT	Ave Norm Reads in RRP6	Average RAW read counts in WT	Average RAW read counts in RRP6
ORF-T	YAR014C	0.0533066	0.8274168	0.8891256	59.07	61.38	564.00	795.50
ORF-T	YPL122C	0.0537639	0.8305419	0.8909432	21.06	21.88	192.00	280.00
ORF-T	YLR252W	0.054225	0.8935975	0.9327231	13.92	14.44	121.75	179.50
ORF-T	YPR049C	0.0543028	0.8250786	0.8875281	37.22	38.67	352.25	522.25
AST	AS_YCL044C	0.054559	0.8817478	0.9240848	2.30	2.40	21.75	32.25
ORF-T	YNL164C	0.0545969	0.8494557	0.9033865	23.52	24.44	223.75	332.00
ORF-T	YLR242C	0.0550064	0.8532743	0.9057282	15.78	16.43	143.75	208.75
NUT	NUT0102	0.0552474	0.9047976	0.940577	1.08	1.09	9.25	14.25
sn/snoRNA	SNR17A	0.0552548	0.8627529	0.911472	36046.79	37454.15	319586.00	472159.25
SUT	SUT791	0.0557951	0.8890563	0.9293339	4.48	4.68	41.75	61.25
ORF-T	YGL055W	0.0559893	0.904321	0.9404311	3237.39	3365.55	30036.00	40343.50
ORF-T	YOL002C	0.0561238	0.8750571	0.9194619	113.87	118.45	1050.00	1466.25
ORF-T	YOR262W	0.0564818	0.8010143	0.8701312	51.83	53.92	479.25	705.75
ORF-T	YDR363W	0.056522	0.8539972	0.9059772	10.53	10.89	94.75	147.00
ORF-T	YGL203C	0.0569837	0.8229608	0.8860017	84.49	87.95	771.00	1100.50
ORF-T	YDR184C	0.0571127	0.8622198	0.9113411	7.56	7.91	73.00	104.00
ORF-T	YOR003W	0.0571412	0.8346256	0.8933848	9.31	9.68	85.25	126.75
CUT	CUT171	0.0571907	0.8924424	0.9319032	1.02	1.09	9.75	14.25
ORF-T	YMR059W	0.0572183	0.8574378	0.9086173	6.82	7.06	61.25	93.50
ORF-T	YPR056W	0.0573212	0.8073995	0.8747117	60.53	63.05	560.50	801.00
ORF-T	YIR011C	0.0578621	0.8005599	0.8699865	70.77	73.71	667.00	963.00
ORF-T	YPL133C	0.0583036	0.8196948	0.8839485	22.30	23.22	202.00	299.25
ORF-T	YLL060C	0.0585384	0.8347354	0.8933848	20.77	21.64	190.00	281.25
ORF-T	YER019W	0.0590078	0.8037728	0.8719068	77.13	80.38	709.75	1044.00
ORF-T	YEL046C	0.0596898	0.826402	0.8885371	140.50	146.38	1252.50	1944.75
ORF-T	YBR043C	0.0597941	0.8345316	0.8933848	55.79	58.20	518.25	747.25
NUT	NUT0594	0.0603742	0.9157583	0.9478094	1.08	1.11	9.50	14.25
ORF-T	YCR011C	0.0608235	0.8313393	0.8910741	271.85	283.61	2479.50	3541.75
ORF-T	YPL026C	0.0611852	0.8077926	0.8749495	30.75	32.10	287.25	421.00

TABLE S1: Differential expression data for RRP6 RNA-Seq dataset Page 202

Class	Transcript name	RRP6 KO_vs_WT log2_fold _change	RRP6 KO_vs_WT p-value	RRP6 KO_vs_WT FDR	Ave Norm Reads in WT	Ave Norm Reads in RRP6	Average RAW read counts in WT	Average RAW read counts in RRP6
SUT	SUT601	0.0613099	0.8955563	0.9339607	1.14	1.20	10.50	15.50
ORF-T	YML035C	0.0617921	0.798168	0.869099	109.62	114.42	1007.00	1498.75
ORF-T	YBR210W	0.061927	0.8305013	0.8909432	9.42	9.79	83.25	127.75
SRT	SRT47	0.0620213	0.92216	0.9522982	0.78	0.78	6.25	10.25
ORF-T	YGL121C	0.0620455	0.8624782	0.9113728	8.71	9.09	75.50	112.25
ORF-T	YDR057W	0.0628561	0.7951926	0.8672824	21.99	22.99	204.00	299.25
NUT	NUT1300	0.062991	0.8219856	0.8856612	15.47	16.09	134.00	208.75
ORF-T	YPR159W	0.0630946	0.8337647	0.8931991	637.70	666.25	5898.25	8578.25
ORF-T	YHR046C	0.0635069	0.8275106	0.8891256	14.60	15.32	140.00	199.00
ORF-T	YGL232W	0.0637076	0.7905415	0.8644844	46.43	48.49	422.75	645.50
ORF-T	YDR524C	0.0637696	0.7987827	0.869299	22.18	23.13	200.50	309.50
AST	AS_YOL156W	0.0639907	0.8514876	0.9045934	4.44	4.64	40.50	61.00
ORF-T	YIL049W	0.0641741	0.8768217	0.9207404	2.08	2.17	19.25	29.00
ORF-T	YLR361C	0.0642743	0.7960133	0.8677836	52.23	54.66	483.00	697.25
ORF-T	YGR016W	0.0644016	0.8292896	0.890145	6.81	7.15	63.50	93.50
ORF-T	YKL033W	0.064535	0.794647	0.8672824	59.45	62.16	560.75	850.25
SUT	SUT417	0.064655	0.8143054	0.8801687	19.73	20.70	186.50	267.00
NUT	NUT0514	0.0650662	0.8324218	0.89195	11.83	12.34	107.25	164.00
ORF-T	YPR196W	0.0651248	0.8437289	0.8998585	12.46	13.01	116.75	182.50
ORF-T	YPL213W	0.066138	0.825193	0.8875281	6.67	6.99	60.25	89.50
ORF-T	YLL055W	0.0662304	0.8276848	0.8891256	47.56	49.85	435.75	616.00
ORF-T	YGL196W	0.0662696	0.7750721	0.8538545	50.81	53.19	469.75	702.75
ORF-T	YJR132W	0.0665463	0.8019553	0.8705382	172.45	180.63	1643.25	2427.00
NUT	NUT0138	0.0670522	0.8454879	0.9008822	7.64	7.93	66.50	107.00
AST	AS_YJR091C	0.0672613	0.9152774	0.9474224	1.85	1.95	19.00	28.75
ORF-T	YER182W	0.0672684	0.8291217	0.890145	26.72	28.02	243.25	349.00
ORF-T	YLR145W	0.0677335	0.827758	0.8891256	5.26	5.51	49.00	73.75
ORF-T	YGR077C	0.0677935	0.8264171	0.8885371	21.56	22.52	197.25	318.25
ORF-T	YIL055C	0.068129	0.8455388	0.9008822	4.64	4.85	41.00	62.25

Class	Transcript name	RRP6 KO_vs_WT log2_fold _change	RRP6 KO_vs_WT p-value	RRP6 KO_vs_WT FDR	Ave Norm Reads in WT	Ave Norm Reads in RRP6	Average RAW read counts in WT	Average RAW read counts in RRP6
ORF-T	YCR107W	0.0685271	0.8142013	0.8801687	8.82	9.24	82.00	123.75
ORF-T	YGR117C	0.0686511	0.7814429	0.8584275	27.29	28.59	246.50	375.25
ORF-T	YPL002C	0.068819	0.7818052	0.8586063	26.73	28.07	243.50	356.00
ORF-T	YHR078W	0.0689209	0.7657952	0.8462771	78.51	82.37	727.50	1079.50
ORF-T	YOR018W	0.0689457	0.7913131	0.86481	27.67	29.01	252.50	380.00
NUT	NUT0634	0.0691517	0.847089	0.9021064	2.57	2.64	22.25	35.00
ORF-T	YGL130W	0.0694682	0.7601952	0.8423965	41.36	43.42	386.00	574.75
ORF-T	YPR051W	0.069758	0.7735738	0.8528171	34.95	36.69	316.75	469.75
ORF-T	YKL150W	0.0698127	0.8163088	0.8814282	54.37	57.06	485.75	717.25
ORF-T	YNL162W-A	0.0699777	0.8686235	0.9152768	3.32	3.46	30.50	48.50
ORF-T	YMR153C-A	0.0699858	0.8756089	0.9198196	1.01	1.08	9.50	14.00
ORF-T	YPL149W	0.0706036	0.7767927	0.8547771	56.60	59.48	515.75	759.00
NUT	NUT1403	0.0707373	0.8429907	0.8992614	114.36	120.16	1054.50	1487.50
ORF-T	YER051W	0.0707526	0.763269	0.8447921	31.90	33.55	297.50	434.25
NUT	NUT0659	0.0716183	0.7994635	0.8696332	22.68	23.86	208.25	306.25
ORF-T	YML003W	0.0719722	0.7971939	0.8686011	9.42	9.90	85.25	128.25
sn/snoRNA	SNR17B	0.0723524	0.8144441	0.8801687	9657.25	10153.86	85107.25	136035.50
ORF-T	YOR220W	0.0727169	0.778406	0.8558986	53.68	56.46	487.25	728.75
NUT	NUT1041	0.0731483	0.7899952	0.8643063	8.14	8.56	74.50	112.25
ORF-T	YGR200C	0.0734254	0.7882625	0.8633474	221.92	233.61	2138.25	3007.75
ORF-T	YMR253C	0.0736007	0.8027729	0.8711031	9.06	9.49	78.50	121.25
ORF-T	YMR137C	0.0737253	0.7973422	0.8686689	11.45	12.05	104.50	156.75
ORF-T	YDR336W	0.0737645	0.7700032	0.8499028	14.68	15.42	133.50	204.00
ORF-T	YOR071C	0.074016	0.7756724	0.8540103	26.27	27.65	235.25	351.50
SUT	SUT115	0.0741728	0.8455821	0.9008822	2.79	2.92	25.75	39.50
ORF-T	YCL056C	0.0742969	0.8238283	0.8866045	3.69	3.87	33.75	51.75
ORF-T	YHR177W	0.0745784	0.8719736	0.9175605	1.91	1.93	15.25	26.00
ORF-T	YGL174W	0.0747753	0.8514863	0.9045934	1.84	1.96	17.75	26.25
SUT	SUT785	0.0756007	0.8524118	0.9052167	1.54	1.67	14.75	21.25

Class	Transcript name	RRP6 KO_vs_WT log2_fold _change	RRP6 KO_vs_WT p-value	RRP6 KO_vs_WT FDR	Ave Norm Reads in WT	Ave Norm Reads in RRP6	Average RAW read counts in WT	Average RAW read counts in RRP6
ORF-T	YMR062C	0.0766873	0.8020796	0.8705382	88.47	93.28	837.50	1314.75
ORF-T	YPL108W	0.0766978	0.7896792	0.8642624	5.08	5.36	46.75	70.25
ORF-T	YOL038C-A	0.0767602	0.8864852	0.9273151	0.98	1.02	8.50	13.00
ORF-T	YGR158C	0.0769768	0.7949728	0.8672824	10.48	11.06	96.50	144.75
ORF-T	YNL316C	0.0769883	0.8396089	0.8967923	4.16	4.34	37.50	60.00
ORF-T	YDR118W-A	0.0771266	0.83448	0.8933848	1.99	2.18	20.00	28.25
ORF-T	YNL022C	0.0773285	0.7583914	0.8414148	98.88	104.40	942.75	1361.25
ORF-T	YER168C	0.0778727	0.7566408	0.8400271	123.25	130.15	1166.50	1697.50
ORF-T	YKL174C	0.0783606	0.7596687	0.8422364	47.22	49.92	438.25	633.25
CUT	CUT509	0.079307	0.865389	0.9127486	1.14	1.14	9.25	15.00
ORF-T	YGR197C	0.0795161	0.8113034	0.8778094	63.98	67.61	553.75	824.50
ORF-T	YDR131C	0.0799301	0.7501994	0.8354903	17.10	18.08	156.75	235.25
NUT	NUT1077	0.0799506	0.8103276	0.8769418	63.96	67.61	553.50	824.50
ORF-T	YNR012W	0.0800578	0.7537177	0.8383509	40.53	42.92	387.25	562.75
ORF-T	YMR313C	0.0804326	0.744758	0.8311354	112.04	118.52	1034.75	1507.75
SRT	SRT120	0.0807117	0.8249322	0.8875084	2.35	2.52	22.25	32.25
ORF-T	YJR135W-A	0.0809572	0.7922833	0.8656826	10.27	10.91	95.75	139.25
ORF-T	YBL028C	0.0810187	0.7935565	0.8665477	72.99	77.22	688.50	1032.50
ORF-T	YCR106W	0.0811675	0.7600573	0.8423363	26.43	27.94	242.00	373.50
NUT	NUT0426	0.0813053	0.8122712	0.8787623	2751.57	2911.07	23978.50	36628.25
AST	AS_YBR283C	0.0813142	0.8874701	0.9279603	6.40	6.79	64.50	98.25
ORF-T	YJL108C	0.0816751	0.876979	0.9208097	4.03	4.28	41.50	63.00
ORF-T	YNR061C	0.0821266	0.7389441	0.8268433	67.37	71.33	621.75	932.25
ORF-T	YOL015W	0.0829816	0.7992884	0.8696332	6.10	6.40	51.75	82.00
ORF-T	YNR025C	0.0838398	0.8255442	0.887788	2.26	2.40	21.25	32.50
SUT	SUT273	0.0843913	0.8427417	0.8992614	2.07	2.18	19.00	29.75
SUT	SUT004	0.0848877	0.7425099	0.8296366	20.96	22.25	192.50	286.00
ORF-T	YOR303W	0.0851506	0.8130716	0.8792567	22.78	24.16	203.50	308.00
ORF-T	YKL189W	0.0852742	0.7828301	0.8593576	10.26	10.80	86.00	139.25

Class	Transcript name	RRP6 KO_vs_WT log2_fold _change	RRP6 KO_vs_WT p-value	RRP6 KO_vs_WT FDR	Ave Norm Reads in WT	Ave Norm Reads in RRP6	Average RAW read counts in WT	Average RAW read counts in RRP6
ORF-T	YHR035W	0.085419	0.7646021	0.8457179	6.14	6.52	57.00	86.00
ORF-T	YOL137W	0.0859327	0.7636406	0.8450074	9.10	9.62	80.50	124.25
ORF-T	YHL050C	0.0865018	0.7588556	0.841652	15.28	16.24	139.25	207.50
ORF-T	YMR069W	0.08655	0.7998342	0.8697862	5.61	5.95	49.75	74.75
ORF-T	YDL146W	0.0866687	0.7078938	0.8029416	54.25	57.61	488.75	735.75
ORF-T	YIL030W-A	0.0868979	0.8015156	0.8703008	2.94	3.18	28.00	40.00
AST	AS_YDR171W	0.0871764	0.8808767	0.9235009	0.96	1.02	9.50	14.75
ORF-T	YML002W	0.0876797	0.7317898	0.8217121	31.66	33.72	297.00	429.75
ORF-T	YKR101W	0.0877324	0.7576863	0.8408175	10.23	10.91	94.25	139.75
ORF-T	YLR130C	0.0877496	0.7459543	0.8320101	119.81	127.35	1120.00	1672.50
ORF-T	YER093C-A	0.0878603	0.8223786	0.8858486	2.03	2.17	18.75	28.00
SUT	SUT672	0.0878789	0.820536	0.8844661	2.06	2.23	19.50	28.25
ORF-T	YBL074C	0.0878799	0.7433283	0.8303167	12.45	13.26	114.75	171.00
ORF-T	YNL280C	0.088056	0.7283377	0.8187912	143.67	152.75	1333.75	1969.00
ORF-T	YNL328C	0.0886895	0.8017889	0.8705038	4.63	4.86	40.75	66.00
SRT	SRT211	0.0893823	0.88296	0.9248747	0.95	0.99	8.50	14.25
NUT	NUT1431	0.0894025	0.822708	0.8859662	1.58	1.71	15.00	22.00
SUT	SUT481	0.0896392	0.8008763	0.8701312	2.17	2.31	19.75	29.75
ORF-T	YIL169C	0.0900393	0.8956168	0.9339607	1.28	1.37	12.75	20.00
ORF-T	YPL152W	0.0901797	0.7782078	0.8558986	9.91	10.55	88.00	132.50
ORF-T	YGL063W	0.0903867	0.7201189	0.8122174	35.38	37.69	322.75	486.25
SRT	SRT128	0.0906276	0.8704744	0.9162479	0.71	0.74	6.25	9.75
SUT	SUT178	0.0906307	0.8704639	0.9162479	0.71	0.74	6.25	9.75
ORF-T	YLL011W	0.090631	0.7485735	0.8342282	125.05	133.23	1216.00	1762.75
ORF-T	YPL088W	0.0907298	0.7709388	0.8506898	50.90	54.29	495.25	703.00
SUT	SUT308	0.0907473	0.885553	0.9266376	0.97	1.04	8.75	13.00
ORF-T	YDL063C	0.0908243	0.6966321	0.7942329	63.05	67.11	582.00	908.75
ORF-T	YOL125W	0.0909003	0.7430681	0.8301683	81.21	86.56	772.25	1128.50
ORF-T	YGL009C	0.0909793	0.7653919	0.8460169	428.64	456.61	3984.00	5647.50

Class	Transcript name	RRP6 KO_vs_WT log2_fold _change	RRP6 KO_vs_WT p-value	RRP6 KO_vs_WT FDR	Ave Norm Reads in WT	Ave Norm Reads in RRP6	Average RAW read counts in WT	Average RAW read counts in RRP6
ORF-T	YLR457C	0.0915434	0.7468004	0.8328617	13.42	14.34	124.00	183.50
SUT	SUT706	0.0918866	0.8439763	0.8999325	1.98	2.13	19.75	28.75
AST	AS_YHR007C-A	0.0919513	0.8483991	0.9026437	0.86	0.95	8.25	12.00
ORF-T	YKL136W	0.0921106	0.8178603	0.8825364	2.21	2.37	20.00	30.00
ORF-T	YGR038W	0.092172	0.7569043	0.8402271	11.33	12.09	101.75	154.50
sn/snoRNA	SNR58	0.0922257	0.7609478	0.8430452	569.15	606.68	5005.50	7919.50
ORF-T	YDR105C	0.092442	0.7108305	0.8048524	76.08	81.12	687.00	1035.50
sn/snoRNA ET	SNR4-ET	0.0925372	0.8069294	0.8743903	303.48	323.52	2512.50	4155.00
ORF-T	YOR174W	0.0929254	0.683791	0.7843646	50.34	53.77	469.50	686.00
AST	AS_YGR109C	0.0929749	0.8662483	0.9132512	1.29	1.40	12.75	18.50
ORF-T	YBL043W	0.0931527	0.810069	0.8768501	4.93	5.24	44.25	67.75
ORF-T	YJL087C	0.0933377	0.6954116	0.7934522	55.14	58.88	519.75	768.50
ORF-T	YEL062W	0.0933963	0.7599166	0.8422729	12.51	13.29	110.50	175.00
ORF-T	YML023C	0.0942275	0.7293207	0.8195309	18.21	19.42	170.50	266.00
ORF-T	YIL029C	0.0946085	0.7597166	0.8422364	7.89	8.42	70.75	108.50
ORF-T	YIL110W	0.0948995	0.6915125	0.7909724	84.98	90.83	799.75	1171.75
ORF-T	YBR302C	0.0949673	0.8305987	0.8909432	1.39	1.50	12.50	18.25
ORF-T	YKL205W	0.0953241	0.692744	0.791744	129.75	138.62	1210.00	1839.50
ORF-T	YML005W	0.0962538	0.6998244	0.7966842	25.62	27.40	239.75	366.25
SUT	SUT244	0.0967692	0.8162383	0.8814282	1.62	1.78	16.25	23.50
ORF-T	YLL009C	0.0972788	0.782306	0.8590628	9.25	9.87	82.00	126.50
ORF-T	YOL076W	0.097392	0.6940216	0.792852	70.85	75.82	667.25	1004.75
ORF-T	YER129W	0.0976991	0.6688216	0.7739638	70.37	75.30	636.75	971.50
SUT	SUT042	0.0978968	0.7265117	0.81765	11.84	12.68	107.50	164.75
ORF-T	YKR017C	0.0987253	0.72681	0.8178088	15.96	17.13	147.50	216.75
NUT	NUT1160	0.0987643	0.8447136	0.9005277	4.93	5.31	48.75	72.50
ORF-T	YLR022C	0.0987995	0.7232046	0.8149285	20.63	22.07	192.75	303.50
ORF-T	YJL132W	0.0990657	0.6967259	0.7942329	33.70	36.14	311.25	459.75
ORF-T	YBR148W	0.0994652	0.8367719	0.89452	5.86	6.32	56.25	78.00

Class	Transcript name	RRP6 KO_vs_WT log2_fold_change	RRP6 KO_vs_WT p-value	RRP6 KO_vs_WT FDR	Ave Norm Reads in WT	Ave Norm Reads in RRP6	Average RAW read counts in WT	Average RAW read counts in RRP6
ORF-T	YDR051C	0.0994786	0.688644	0.7887649	19.34	20.71	177.25	272.25
ORF-T	YER187W	0.0996655	0.7765658	0.8547316	10.43	11.20	94.25	140.50
SUT	SUT614	0.100079	0.7736753	0.8528358	3.45	3.68	32.25	50.50
SUT	SUT174	0.100223	0.7977357	0.8689202	2.13	2.27	19.50	30.75
ORF-T	YHR131C	0.1003233	0.7088265	0.8034046	94.14	101.00	884.75	1286.25
ORF-T	YIL001W	0.1003263	0.7346179	0.8236476	6.15	6.55	55.75	88.25
ORF-T	YOR274W	0.1007545	0.706936	0.8023345	21.18	22.75	196.50	291.75
ORF-T	YKL123W	0.1010951	0.731588	0.8217121	8.49	9.11	77.25	117.00
ORF-T	YNL260C	0.1018074	0.7166436	0.8092535	14.61	15.65	135.50	212.25
ORF-T	YOR334W	0.1024448	0.7056924	0.8014644	13.15	14.17	125.00	185.75
SUT	SUT014	0.1025759	0.6924976	0.7917399	36.96	39.72	350.00	529.50
NUT	NUT1037	0.1027947	0.7219621	0.8140742	8.90	9.59	83.50	125.75
NUT	NUT1395	0.1037091	0.7322883	0.8218764	570.73	613.23	5019.75	8003.00
ORF-T	YPL036W	0.1037786	0.6990854	0.7962925	14.34	15.40	132.25	204.75
NUT	NUT0186	0.1041022	0.7565771	0.8400271	480.49	516.45	4304.25	6580.75
SUT	SUT121	0.1049817	0.8200972	0.8842879	0.92	1.00	9.00	13.75
ORF-T	YPR010C-A	0.105276	0.7718528	0.8515716	12.58	13.54	109.75	161.00
ORF-T	YDL238C	0.1054136	0.7193398	0.8116619	24.73	26.58	219.00	341.25
sn/snoRNA	SNR39	0.1060691	0.7130658	0.8068397	43.95	47.27	380.25	602.00
SUT	SUT343	0.1063152	0.8309347	0.8910741	0.92	0.93	7.50	12.50
ORF-T	YHL048W	0.1068389	0.6950797	0.7933943	20.30	21.82	181.00	283.75
ORF-T	YOL095C	0.1071016	0.7139664	0.8074963	5.78	6.18	53.00	84.25
ORF-T	YOL020W	0.1072973	0.6896321	0.7894483	262.53	282.86	2432.75	3578.50
ORF-T	YLR360W	0.107855	0.6681703	0.7735143	31.92	34.35	282.50	446.75
ORF-T	YLR299W	0.1082783	0.6836619	0.7843646	48.29	52.10	438.75	645.00
ORF-T	YCR015C	0.1092142	0.6514357	0.7612791	27.12	29.28	253.25	383.75
NUT	NUT0318	0.1093655	0.687775	0.7881277	20.42	21.98	182.25	286.00
ORF-T	YIL107C	0.1095887	0.7141618	0.8076268	64.90	70.09	601.50	878.00
ORF-T	YGL091C	0.1099806	0.6345849	0.7490523	55.14	59.56	506.50	755.75

Class	Transcript name	RRP6 KO_vs_WT log2_fold _change	RRP6 KO_vs_WT p-value	RRP6 KO_vs_WT FDR	Ave Norm Reads in WT	Ave Norm Reads in RRP6	Average RAW read counts in WT	Average RAW read counts in RRP6
ORF-T	YIR034C	0.1100947	0.7598028	0.8422393	6.97	7.46	62.00	104.00
ORF-T	YML101C-A	0.1103109	0.7681257	0.8483877	2.38	2.58	22.00	34.00
ORF-T	YER144C	0.1106861	0.6488916	0.7597148	36.43	39.30	332.75	524.50
ORF-T	YDR119W-A	0.110837	0.7887479	0.8636768	16.31	17.59	139.25	219.00
ORF-T	YOR316C	0.1112357	0.6695506	0.7744219	124.30	134.31	1147.00	1706.25
ORF-T	YER169W	0.1118983	0.6697309	0.7744916	29.99	32.40	278.25	426.00
ORF-T	YIL121W	0.1121277	0.7319137	0.8217121	123.87	133.92	1115.00	1634.00
AST	AS_YJL037W	0.1126361	0.8509176	0.9041784	0.81	0.89	8.25	12.75
ORF-T	YGR216C	0.112978	0.6613447	0.7690915	32.94	35.59	301.25	478.75
SUT	SUT540	0.1132662	0.7562527	0.8398735	2.58	2.82	23.75	35.25
ORF-T	YCL001W-A	0.113363	0.7951071	0.8672824	1.10	1.17	9.75	15.50
ORF-T	YOL001W	0.1135604	0.6772405	0.7796887	11.72	12.60	101.50	165.50
ORF-T	YCR020W-B	0.1136714	0.7080926	0.8029416	26.96	29.16	245.75	380.00
ORF-T	YPL254W	0.1140847	0.6486526	0.7595231	70.42	76.29	682.50	1016.00
ORF-T	YMR201C	0.1149877	0.7248433	0.816319	5.05	5.45	45.50	72.00
ORF-T	YBR008C	0.1150629	0.6576319	0.7662114	28.24	30.55	259.25	412.25
ORF-T	YDR082W	0.1155709	0.6671498	0.7729354	20.73	22.51	194.50	293.00
NUT	NUT1501	0.1157604	0.6211909	0.7381793	35.86	38.84	326.75	508.50
ORF-T	YJL216C	0.1157883	0.7440429	0.8306132	5.39	5.88	50.50	74.75
ORF-T	YGR073C	0.1158813	0.74077	0.8280156	3.99	4.29	35.00	56.00
SRT	SRT345	0.1159126	0.8147957	0.8804543	1.02	1.13	9.75	14.50
ORF-T	YGR246C	0.1163957	0.6181908	0.7360757	41.79	45.32	394.00	603.25
ORF-T	YDR079C-A	0.116511	0.6682722	0.7735143	21.36	23.14	191.25	298.25
ORF-T	YLR030W	0.1165416	0.786426	0.8622323	1.95	2.09	17.75	28.75
ORF-T	YHR187W	0.1166997	0.6493007	0.7598408	34.26	37.20	332.75	501.25
ORF-T	YBR203W	0.1168336	0.6952035	0.7933943	74.16	80.44	684.25	1026.50
ORF-T	YKR028W	0.1169746	0.6319031	0.7470235	115.14	124.86	1058.00	1652.50
ORF-T	YOR153W	0.1170688	0.6741772	0.777932	592.34	642.38	5525.00	8903.00
ORF-T	YDL243C	0.1171253	0.7027988	0.7992583	11.56	12.58	111.25	165.00

Class	Transcript name	RRP6 KO_vs_WT log2_fold _change	RRP6 KO_vs_WT p-value	RRP6 KO_vs_WT FDR	Ave Norm Reads in WT	Ave Norm Reads in RRP6	Average RAW read counts in WT	Average RAW read counts in RRP6
ORF-T	YGL124C	0.1171857	0.6530575	0.7626441	26.34	28.58	247.75	380.25
ORF-T	YDL080C	0.1172855	0.608723	0.7288486	46.07	50.04	433.50	649.50
ORF-T	YOR010C	0.1173767	0.8177976	0.8825364	1.11	1.15	9.00	15.00
ORF-T	YIR025W	0.1179432	0.6730419	0.7770729	8.56	9.27	77.25	121.50
NUT	NUT0134	0.1179455	0.7886725	0.8636768	2.72	2.96	26.00	40.50
ORF-T	YHR075C	0.118043	0.6617516	0.7692338	12.57	13.66	114.50	176.50
ORF-T	YKL077W	0.1181547	0.6428074	0.7558384	94.49	102.56	858.50	1317.75
ORF-T	YLR099C	0.1185364	0.6902928	0.7901149	25.86	28.12	242.25	369.00
ORF-T	YLR107W	0.1188721	0.6018105	0.7244374	41.75	45.40	392.00	590.75
ORF-T	YNL023C	0.1190602	0.6344068	0.7489297	69.40	75.44	667.50	1001.25
ORF-T	YHR155W	0.1190864	0.5992793	0.7226899	52.40	56.88	478.00	756.00
ORF-T	YBR126W-B	0.119158	0.6601407	0.7682704	66.80	72.53	582.25	902.50
ORF-T	YJR097W	0.119186	0.805001	0.8726757	1.80	1.95	17.75	28.00
ORF-T	YNL258C	0.1192369	0.6141317	0.7330314	45.40	49.32	413.25	635.50
AST	AS_YGL136C	0.1193148	0.6846214	0.7850491	9.31	10.12	86.75	135.75
SUT	SUT061	0.1196939	0.7866459	0.8622323	2.76	3.01	26.50	41.25
ORF-T	YBR001C	0.119872	0.657768	0.7662815	81.90	89.04	744.25	1109.25
AST	AS_YBR007C	0.1200253	0.7945353	0.8672824	1.09	1.18	10.00	15.50
ORF-T	YNL256W	0.1203438	0.6336154	0.7484614	107.15	116.55	1035.00	1536.25
ORF-T	YMR239C	0.1205335	0.6816079	0.7830182	60.63	66.00	598.25	878.00
ORF-T	YKL166C	0.1206795	0.6328205	0.7477575	51.88	56.49	498.50	734.50
ORF-T	YPL140C	0.121053	0.6182627	0.7360757	31.50	34.35	299.50	441.50
ORF-T	YDR381C-A	0.1210729	0.6583357	0.7667657	16.79	18.26	150.00	228.75
ORF-T	YLR433C	0.1212349	0.6272936	0.7432025	23.66	25.74	217.25	334.25
ORF-T	YGL006W	0.1213284	0.6864292	0.7867642	250.15	272.11	2255.25	3413.00
NUT	NUT0841	0.1213586	0.5976906	0.7212916	36.94	40.20	338.50	523.50
AST	AS_YOL013C	0.1224222	0.8597977	0.9098751	0.90	0.99	9.50	14.75
ORF-T	YKL209C	0.1236812	0.6515279	0.7612987	381.25	415.38	3470.75	5326.50
SUT	SUT616	0.1236837	0.7253237	0.8165864	3.55	3.85	31.75	50.75

TABLE S1: Differential expression data for RRP6 RNA-Seq dataset Page 210

Class	Transcript name	RRP6 KO_vs_WT log2_fold _change	RRP6 KO_vs_WT p-value	RRP6 KO_vs_WT FDR	Ave Norm Reads in WT	Ave Norm Reads in RRP6	Average RAW read counts in WT	Average RAW read counts in RRP6
NUT	NUT0531	0.1243883	0.6504429	0.7604714	17.25	18.80	157.00	244.75
ORF-T	YBR147W	0.1244888	0.7697181	0.8497742	22.74	24.83	207.00	301.75
ORF-T	YIL085C	0.1245676	0.5998877	0.7230066	98.57	107.50	906.75	1376.25
ORF-T	YBR126W-A	0.1245685	0.6435348	0.7559884	46.61	50.79	405.00	634.50
ORF-T	YNL275W	0.1248633	0.5938713	0.7178852	41.00	44.81	389.25	576.50
ORF-T	YEL063C	0.1252673	0.5832846	0.7091174	63.13	68.82	570.25	903.00
ORF-T	YDL203C	0.1255421	0.5934107	0.7175533	65.12	71.12	623.00	940.00
ORF-T	YDR191W	0.1255558	0.6370496	0.7509947	14.98	16.35	139.50	216.50
sn/snoRNA ET	SNR191_RT_NOG2	0.1261498	0.6682402	0.7735143	141.56	154.56	1369.75	2022.00
NUT	NUT0604	0.1262833	0.7378278	0.8263278	1.88	2.05	17.25	27.00
ORF-T	YKL093W	0.1264502	0.7276191	0.818257	13.67	14.93	123.25	190.25
NUT	NUT0676	0.1267783	0.6670915	0.7729354	8.60	9.43	82.75	127.25
ORF-T	YKL061W	0.1268647	0.7063355	0.802014	3.27	3.54	28.25	45.00
ORF-T	YOL140W	0.1274038	0.6781297	0.7804451	55.21	60.36	521.75	784.75
SUT	SUT493	0.1275915	0.758069	0.8411497	3.08	3.36	29.25	47.00
ORF-T	YIL047C	0.1277785	0.63816	0.7516885	272.98	298.32	2581.75	3885.75
ORF-T	YMR285C	0.1282394	0.5959454	0.7197256	53.66	58.69	495.00	758.00
ORF-T	YDR244W	0.1283349	0.5985802	0.7221059	50.62	55.39	470.00	705.00
ORF-T	YJL058C	0.1284681	0.7119291	0.8058247	4.25	4.60	38.25	63.00
AST	AS_YIL153W	0.1290284	0.7570884	0.8403389	1.82	1.96	16.75	27.75
SUT	SUT657	0.1291105	0.7332023	0.822456	2.32	2.56	21.00	31.75
ORF-T	YKL050C	0.1294352	0.6590613	0.7674335	19.44	21.32	183.75	275.75
ORF-T	YLL054C	0.1294628	0.6495804	0.7598693	8.26	9.03	74.75	116.25
ORF-T	YBR033W	0.1299763	0.7641083	0.8454321	5.21	5.70	45.50	69.00
ORF-T	YML099C	0.1301303	0.596966	0.720568	38.93	42.57	364.00	584.50
ORF-T	YNL326C	0.1305643	0.645852	0.7573871	22.21	24.33	199.50	307.25
ORF-T	YPR013C	0.1310387	0.6763338	0.779234	6.59	7.14	57.75	96.50
ORF-T	YDR490C	0.1311553	0.6280408	0.7436658	43.25	47.38	402.50	627.75
ORF-T	YDR234W	0.1319965	0.6843469	0.7848937	223.53	245.01	2184.50	3265.75

Class	Transcript name	RRP6 KO_vs_WT log2_fold _change	RRP6 KO_vs_WT p-value	RRP6 KO_vs_WT FDR	Ave Norm Reads in WT	Ave Norm Reads in RRP6	Average RAW read counts in WT	Average RAW read counts in RRP6
ORF-T	YFR009W-A	0.1324591	0.6048476	0.726325	54.21	59.49	524.50	791.25
ORF-T	YOR316C-A	0.1325028	0.6276498	0.7434772	14.99	16.51	140.50	209.50
ORF-T	YDR362C	0.132647	0.6061297	0.7272213	27.33	29.99	254.25	392.00
NUT	NUT1127	0.1331674	0.6386444	0.7519956	8.44	9.23	75.50	118.75
ORF-T	YOR273C	0.133668	0.7961641	0.8678542	78.14	85.72	664.25	1024.75
ORF-T	YMR287C	0.1342206	0.5664892	0.6954562	30.68	33.73	287.75	439.25
ORF-T	YOR044W	0.1357228	0.6570138	0.7655798	14.25	15.66	127.50	198.25
ORF-T	YJR124C	0.1365956	0.6090337	0.7290291	73.22	80.55	695.75	1061.50
ORF-T	YOR102W	0.1367036	0.8076419	0.8748803	0.80	0.91	8.25	12.50
ORF-T	YNR070W	0.1368133	0.6253603	0.7416264	13.88	15.28	130.25	203.25
SRT	SRT284	0.1375486	0.672282	0.7764623	5.59	6.18	51.75	79.75
SUT	SUT276	0.1375536	0.7219215	0.8140742	2.80	3.05	24.75	40.00
ORF-T	YML132W	0.1375871	0.7587571	0.8416353	1.47	1.67	14.50	21.25
ORF-T	YGR081C	0.1377613	0.619487	0.7369243	30.97	34.06	292.00	466.50
NUT	NUT1058	0.1379899	0.7755115	0.8540103	1.43	1.59	14.25	22.25
ORF-T	YKL078W	0.1381699	0.6626986	0.7699796	31.38	34.59	312.75	471.50
ORF-T	YGR059W	0.1397787	0.7721354	0.8517902	1.55	1.67	13.25	22.25
AST	AS_YGL024W	0.1400618	0.7384841	0.8264203	0.93	1.05	9.00	14.00
ORF-T	YOL021C	0.1401753	0.5846165	0.7099316	213.90	235.77	2003.75	3072.50
NUT	NUT0074	0.141194	0.6994824	0.7965647	8.08	8.93	71.25	109.25
NUT	NUT1151	0.1418943	0.6606537	0.7687551	4.06	4.46	37.50	60.50
ORF-T	YMR127C	0.1419408	0.68377	0.7843646	13.16	14.54	122.50	191.50
ORF-T	YFL056C	0.1422539	0.5573725	0.6879491	31.19	34.42	285.50	450.50
ORF-T	YKR079C	0.1426134	0.5690038	0.6972699	86.85	95.96	831.25	1251.50
ORF-T	YKL208W	0.1426726	0.5791888	0.7054645	22.11	24.47	207.25	313.75
ORF-T	YMR247C	0.1427251	0.5601256	0.690493	143.22	158.17	1341.50	2065.25
ORF-T	YPL112C	0.1434912	0.5761453	0.7026616	21.41	23.65	195.00	308.75
ORF-T	YLR247C	0.1438421	0.5611852	0.6909636	99.44	109.91	920.25	1427.00
ORF-T	YDR361C	0.1441265	0.5943136	0.7183337	123.23	136.21	1167.75	1827.50

Class	Transcript name	RRP6 KO_vs_WT log2_fold _change	RRP6 KO_vs_WT p-value	RRP6 KO_vs_WT FDR	Ave Norm Reads in WT	Ave Norm Reads in RRP6	Average RAW read counts in WT	Average RAW read counts in RRP6
ORF-T	YHR118C	0.1442821	0.6037649	0.7257042	11.01	12.18	102.50	160.75
ORF-T	YPR085C	0.1446486	0.6172126	0.7351727	6.61	7.30	60.25	95.75
ORF-T	YBR274W	0.1448578	0.5310174	0.6677615	54.10	59.87	501.50	771.00
ORF-T	YGL166W	0.1457059	0.5838869	0.7094508	33.96	37.66	319.75	475.50
ORF-T	YPL047W	0.1465342	0.5615363	0.6911426	30.16	33.45	289.00	442.50
ORF-T	YHR044C	0.1466298	0.6763612	0.779234	4.04	4.45	36.50	59.50
SUT	SUT031	0.146934	0.6890454	0.7889558	8.02	8.90	70.75	108.75
ORF-T	YDL033C	0.1471148	0.544822	0.67735	29.30	32.43	270.50	432.75
SUT	SUT336	0.1472316	0.6064715	0.7274484	13.56	15.07	126.75	193.00
SUT	SUT223	0.1474857	0.6338616	0.7484614	12.30	13.63	112.25	176.00
SUT	SUT630	0.1474999	0.6797514	0.7814196	2.92	3.24	26.00	40.75
ORF-T	YGR250C	0.147631	0.6159105	0.7340553	126.03	139.59	1141.25	1797.75
ORF-T	YGR283C	0.1480129	0.6024764	0.7247425	22.90	25.42	224.25	348.25
NUT	NUT1321	0.1482442	0.6966461	0.7942329	8.83	9.75	74.75	122.25
ORF-T	YGR154C	0.1485827	0.6006429	0.7238149	15.54	17.21	139.75	221.75
SUT	SUT737	0.1502625	0.7632306	0.8447921	1.48	1.62	13.50	22.50
ORF-T	YER106W	0.1504138	0.7381725	0.8264203	1.43	1.59	13.50	21.50
ORF-T	YIL151C	0.1510449	0.4964168	0.6394612	47.58	52.86	439.25	685.00
NUT	NUT0856	0.1516268	0.7576659	0.8408175	0.94	1.08	9.00	13.75
ORF-T	YDL205C	0.1521191	0.5689321	0.6972668	99.54	110.61	926.75	1469.00
SUT	SUT537	0.1522257	0.7879063	0.8630509	1.44	1.55	11.75	20.25
ORF-T	YMR013W-A	0.1522735	0.6260911	0.7420772	1501.20	1668.29	13150.25	21219.00
ORF-T	YDR305C	0.1530053	0.5257722	0.6635653	24.15	26.86	221.75	349.75
AST	AS_YIL109C	0.1530118	0.7403542	0.8277326	1.85	2.08	17.25	27.00
SUT	SUT665	0.1534831	0.5604596	0.690493	10.84	12.04	100.25	162.00
ORF-T	YJL217W	0.154261	0.7803429	0.8575608	23.84	26.48	205.50	377.25
ORF-T	YPR086W	0.1543913	0.5211827	0.6593446	78.14	87.06	741.00	1118.25
ORF-T	YDR151C	0.1557448	0.5695753	0.6978855	24.63	27.38	216.00	351.25
sn/snoRNA	SNR62	0.1561166	0.6748086	0.7782149	157.80	175.82	1361.25	2229.75

Class	Transcript name	RRP6 KO_vs_WT log2_fold _change	RRP6 KO_vs_WT p-value	RRP6 KO_vs_WT FDR	Ave Norm Reads in WT	Ave Norm Reads in RRP6	Average RAW read counts in WT	Average RAW read counts in RRP6
ORF-T	YDL129W	0.156585	0.5592776	0.689727	33.09	36.95	323.75	496.25
ORF-T	YMR211W	0.1566772	0.5357082	0.6714253	28.92	32.27	275.25	434.50
NUT	NUT0793	0.1572065	0.6553991	0.7648472	5.81	6.52	54.50	84.00
NUT	NUT0478	0.1574151	0.7039778	0.8000577	2.44	2.77	23.75	35.50
ORF-T	YER171W	0.1580481	0.5024611	0.6447974	54.71	61.09	515.75	808.25
AST	AS_YLL037W	0.1583248	0.5550606	0.6856831	10.58	11.84	100.50	157.75
CUT	CUT567	0.1583534	0.6207822	0.7379426	119.71	133.58	1044.75	1663.50
ORF-T	YNL063W	0.1584952	0.5329374	0.6691363	16.01	17.86	146.50	233.25
sn/snoRNA	SNR19	0.1585975	0.5865207	0.7114729	6499.52	7254.75	57325.00	92733.25
NUT	NUT1342	0.1590926	0.5854055	0.7103766	6503.17	7261.32	57357.50	92816.50
SUT	SUT362	0.1596287	0.5932581	0.717488	7.10	7.93	63.50	101.50
ORF-T	YHR015W	0.1601699	0.7099877	0.8040786	5.66	6.34	53.50	84.75
ORF-T	YDR022C	0.1607091	0.6719791	0.7762347	3.54	3.94	32.00	52.25
ORF-T	YLR453C	0.1614573	0.574021	0.7014456	8.12	9.10	73.75	116.50
ORF-T	YLR363C	0.1619086	0.5727383	0.7005693	15.38	17.12	136.00	232.50
ORF-T	YGR156W	0.1619732	0.6496265	0.7598693	2.99	3.28	25.75	44.00
ORF-T	YKR060W	0.1626916	0.6266652	0.7426598	11.95	13.34	108.25	182.50
ORF-T	YDR484W	0.1630078	0.5677645	0.6962589	22.32	24.97	201.00	326.25
ORF-T	YCR066W	0.1632526	0.6146762	0.7332776	7.15	7.99	67.00	108.75
ORF-T	YKL124W	0.163327	0.53755	0.6722042	39.61	44.38	358.75	563.00
ORF-T	YDL206W	0.1639297	0.5233034	0.6616088	20.32	22.74	182.25	293.50
ORF-T	YKL076C	0.1642796	0.6459994	0.7574693	3.86	4.24	33.25	58.75
NUT	NUT0466	0.1652203	0.5518534	0.6831438	12.68	14.21	113.75	182.75
ORF-T	YNL277W	0.1653449	0.7919021	0.8653599	9.40	10.55	82.00	122.75
ORF-T	YHR003C	0.1654235	0.5118075	0.6523004	76.14	85.38	716.50	1165.25
ORF-T	YBR176W	0.1655752	0.5167101	0.6561522	22.52	25.23	202.75	326.75
ORF-T	YDL158C	0.1660151	0.6014114	0.7241941	8.63	9.74	80.50	122.00
NUT	NUT0796	0.1662907	0.5483383	0.6801453	20.19	22.72	190.50	293.50
ORF-T	YIR027C	0.1670452	0.7272822	0.8179694	3.91	4.40	37.00	58.25

Class	Transcript name	RRP6 KO_vs_WT log2_fold _change	RRP6 KO_vs_WT p-value	RRP6 KO_vs_WT FDR	Ave Norm Reads in WT	Ave Norm Reads in RRP6	Average RAW read counts in WT	Average RAW read counts in RRP6
ORF-T	YOL075C	0.1675598	0.4547177	0.6052182	50.57	56.81	464.50	741.75
ORF-T	YCL049C	0.1677422	0.571533	0.6995065	22.20	24.97	200.00	311.75
ORF-T	YMR057C	0.1681713	0.8011818	0.8701987	0.61	0.70	6.25	10.00
ORF-T	YER175C	0.1691546	0.5778015	0.7041149	12.52	14.00	111.25	190.00
ORF-T	YOL130W	0.1693508	0.5631735	0.692735	128.02	144.03	1205.75	1868.75
ORF-T	YFR009W	0.1697726	0.5201669	0.6586702	402.49	452.78	3799.50	6000.50
ORF-T	YMR015C	0.1698744	0.5171661	0.6563426	45.64	51.28	407.50	680.00
ORF-T	YDR143C	0.1700695	0.5165239	0.6559983	17.08	19.15	150.25	251.50
ORF-T	YEL048C	0.1706328	0.5621948	0.6916155	15.60	17.57	145.00	232.75
ORF-T	YGL159W	0.170732	0.4624151	0.6119415	31.36	35.32	290.50	464.50
ORF-T	YDR118W	0.1714618	0.4714905	0.6199061	30.41	34.33	287.00	445.25
AST	AS_YOR213C	0.1730207	0.6169459	0.7350286	3.45	3.94	33.00	51.00
SUT	SUT723	0.173348	0.6757206	0.778917	1.81	2.06	16.25	25.25
ORF-T	YPR198W	0.1734088	0.5043632	0.6462489	67.57	76.28	635.25	980.25
NUT	NUT1278	0.173505	0.7077719	0.8029416	1.18	1.35	10.75	16.75
AST	AS_YMR215W	0.1745256	0.7139565	0.8074963	1.86	2.13	18.75	29.25
ORF-T	YBR126C	0.1751175	0.6460724	0.7574693	247.78	279.74	2159.50	3486.50
ORF-T	YLL007C	0.1752252	0.6413797	0.7545565	9.24	10.39	79.75	132.00
CUT	CUT237	0.1758247	0.5202468	0.6586702	10.70	12.09	97.25	155.50
NUT	NUT0615	0.1760341	0.6145039	0.7332754	3.48	3.97	32.50	50.75
NUT	NUT0001	0.1762582	0.4738188	0.6212702	39.07	44.17	358.75	572.25
ORF-T	YDR030C	0.1774842	0.5771831	0.7035313	8.70	9.84	79.50	127.25
ORF-T	YKR061W	0.177898	0.553222	0.6842501	34.43	38.98	328.25	525.00
ORF-T	YDL231C	0.1789806	0.5032359	0.6455678	148.40	168.06	1395.75	2188.25
NUT	NUT0507	0.1790604	0.6056265	0.7268671	12.08	13.65	106.25	172.75
NUT	NUT0104	0.1798669	0.5543375	0.6850416	5.93	6.75	55.50	88.50
ORF-T	YNL119W	0.1800015	0.5038485	0.6459655	42.79	48.51	407.50	647.75
NUT	NUT0255	0.1800994	0.5705703	0.6986498	167.96	190.21	1417.75	2474.75
SUT	SUT732	0.1806956	0.5678746	0.6963092	5.37	6.12	50.00	78.00

Class	Transcript name	RRP6 KO_vs_WT log2_fold _change	RRP6 KO_vs_WT p-value	RRP6 KO_vs_WT FDR	Ave Norm Reads in WT	Ave Norm Reads in RRP6	Average RAW read counts in WT	Average RAW read counts in RRP6
ORF-T	YGR004W	0.1818957	0.5104514	0.6512666	52.26	59.35	488.00	755.50
AST	AS_YPL210C	0.1820526	0.7517004	0.8365325	0.71	0.86	7.50	11.25
ORF-T	YOR219C	0.1821267	0.4429631	0.5953606	53.61	60.84	493.75	793.00
SRT	SRT250	0.1830596	0.6742709	0.777932	0.94	1.08	8.50	13.50
SUT	SUT477	0.1830998	0.5462569	0.6786318	5.38	6.11	49.75	81.00
ORF-T	YGR075C	0.1831727	0.5372637	0.6720128	9.85	11.15	88.75	146.25
AST	AS_YNL118C	0.1842042	0.7751922	0.8538545	0.68	0.76	6.25	10.25
SRT	SRT263	0.1843469	0.700622	0.7974122	1.04	1.20	10.25	16.50
ORF-T	YDR020C	0.1846864	0.4934479	0.6371227	20.88	23.76	194.75	312.50
NUT	NUT1382	0.1847687	0.5180401	0.6572618	8.36	9.51	77.50	125.25
ORF-T	YHR036W	0.1849261	0.4279513	0.5829213	35.64	40.55	332.25	529.00
ORF-T	YOR149C	0.1850476	0.4731878	0.6208801	18.51	21.03	168.50	275.25
ORF-T	YJR009C	0.1856111	0.5853837	0.7103766	637.90	725.45	5454.75	8953.75
ORF-T	YAR029W	0.1862932	0.6471218	0.7582688	1.38	1.55	12.25	20.00
ORF-T	YLR092W	0.186392	0.6152537	0.7335325	9.96	11.32	87.75	141.75
SUT	SUT162	0.1864231	0.5647752	0.6938588	3.15	3.63	30.50	48.50
CUT	CUT359	0.1870493	0.6785148	0.780621	1.16	1.30	10.50	17.50
ORF-T	YBR293W	0.1873484	0.4301521	0.5845894	34.46	39.21	307.50	509.25
NUT	NUT0528	0.1875192	0.658275	0.7667657	2.07	2.34	19.00	32.00
SUT	SUT409	0.1880384	0.5298751	0.6669071	12.23	13.98	117.50	184.75
SRT	SRT441	0.1881022	0.6651752	0.7716594	3.10	3.58	28.50	42.00
ORF-T	YAL064W-B	0.1881053	0.5959782	0.7197256	3.59	4.13	33.25	52.25
ORF-T	YNR019W	0.1883301	0.4593282	0.6091679	51.20	58.37	469.50	749.25
NUT	NUT1073	0.1883549	0.4937565	0.6373387	16.18	18.42	146.00	239.00
AST	AS_YEL014C	0.1888088	0.5470324	0.6792605	4.85	5.54	45.50	73.75
NUT	NUT1520	0.1889933	0.6146529	0.7332776	2.96	3.39	27.00	43.50
ORF-T	YPR106W	0.1898407	0.6185098	0.7362208	3.25	3.62	27.25	48.50
ORF-T	YBR109W-A	0.1900163	0.6195656	0.7369308	4.44	5.09	40.75	64.50
ORF-T	YOL091W	0.1902843	0.6565282	0.7653471	5.61	6.42	52.50	84.00

Class	Transcript name	RRP6 KO_vs_WT log2_fold _change	RRP6 KO_vs_WT p-value	RRP6 KO_vs_WT FDR	Ave Norm Reads in WT	Ave Norm Reads in RRP6	Average RAW read counts in WT	Average RAW read counts in RRP6
SUT	SUT666	0.1903361	0.5740807	0.7014456	7.85	8.96	69.00	111.00
ORF-T	YIL165C	0.1904739	0.5012753	0.6436853	11.40	13.02	104.50	168.25
NUT	NUT0328	0.1904863	0.6470994	0.7582688	1.91	2.20	18.75	30.50
ORF-T	YOR298W	0.1909355	0.6819117	0.783278	3.04	3.46	28.75	49.00
ORF-T	YDL008W	0.1909453	0.4781582	0.624336	21.37	24.35	192.25	320.25
ORF-T	YMR073C	0.1909562	0.4423624	0.5949296	17.33	19.82	159.75	255.25
ORF-T	YCL058W-A	0.1910554	0.6548818	0.7644205	5.48	6.25	52.75	89.00
ORF-T	YPL214C	0.1911071	0.4215538	0.5768654	65.48	74.82	610.75	969.25
ORF-T	YGR029W	0.1913474	0.3912791	0.552072	44.58	50.92	408.50	657.50
ORF-T	YMR068W	0.191353	0.5078398	0.6493034	11.19	12.81	105.00	168.00
ORF-T	YDL048C	0.191613	0.705874	0.8015804	17.82	20.31	143.75	249.75
ORF-T	YBR063C	0.1920095	0.4897609	0.6339043	23.70	27.07	218.25	361.00
ORF-T	YLR164W	0.1921486	0.4902603	0.6343879	8.45	9.61	76.75	128.25
ORF-T	YMR061W	0.1938865	0.4129227	0.5699314	99.15	113.46	922.00	1462.75
ORF-T	YDR419W	0.1939552	0.4688952	0.6174378	27.60	31.65	260.00	408.25
AST	AS_YBR149W	0.1941612	0.7149001	0.8081898	2.47	2.80	23.50	40.75
ORF-T	YFR034W-A	0.1943851	0.5511503	0.6824799	5.79	6.63	52.25	83.75
SUT	SUT392	0.195466	0.6119288	0.7308646	2.18	2.48	19.25	32.00
NUT	NUT0703	0.1955216	0.5054216	0.6472786	7.32	8.37	65.50	107.50
ORF-T	YER150W	0.1959683	0.6566781	0.7653471	11.67	13.34	96.50	162.00
SUT	SUT235	0.1960404	0.4764903	0.6236926	16.57	19.04	155.50	242.00
ORF-T	YNL065W	0.196114	0.6652683	0.7716594	94.14	107.93	910.75	1292.50
ORF-T	YJL086C	0.196187	0.5821565	0.7083946	2.61	3.01	24.50	39.00
SUT	SUT021	0.1962255	0.5709478	0.6988878	2.61	2.96	24.00	40.25
ORF-T	YOL088C	0.1964816	0.4064268	0.5647524	62.79	71.97	572.50	924.25
ORF-T	YDR438W	0.1965645	0.435503	0.5893348	26.48	30.38	244.25	394.00
ORF-T	YDR206W	0.1967585	0.47224	0.6203033	153.33	175.80	1443.00	2276.25
ORF-T	YBR050C	0.1980379	0.66544	0.7717421	5.25	5.98	46.75	81.75
ORF-T	YPR021C	0.1981511	0.3984684	0.5591643	32.58	37.36	294.00	481.00

Class	Transcript name	RRP6 KO_vs_WT log2_fold _change	RRP6 KO_vs_WT p-value	RRP6 KO_vs_WT FDR	Ave Norm Reads in WT	Ave Norm Reads in RRP6	Average RAW read counts in WT	Average RAW read counts in RRP6
ORF-T	YHR130C	0.1982957	0.5519656	0.6831759	4.91	5.69	45.75	71.75
ORF-T	YPL198W	0.1986869	0.5286336	0.6657825	55.72	63.86	472.25	830.50
SUT	SUT176	0.198923	0.4739003	0.6212702	12.59	14.48	116.50	188.75
ORF-T	YHR002W	0.1998037	0.5997055	0.7229445	8.41	9.66	74.50	119.25
ORF-T	YCR060W	0.1998904	0.526184	0.6639189	3.49	4.05	32.75	52.25
ORF-T	YPL135W	0.2013302	0.4593527	0.6091679	45.73	52.62	428.25	691.50
NUT	NUT0237	0.2016874	0.4569655	0.60704	18.03	20.78	166.25	265.00
ORF-T	YNL003C	0.2019363	0.4053975	0.5640168	53.33	61.37	489.00	788.50
ORF-T	YHL026C	0.2020093	0.5788631	0.7052381	3.52	3.97	30.75	54.25
ORF-T	YAR003W	0.2036025	0.4345752	0.589013	77.14	88.86	711.25	1141.25
ORF-T	YIL014W	0.2046065	0.4133169	0.5700523	104.15	120.12	989.75	1534.75
NUT	NUT0459	0.204744	0.4475456	0.5993646	277.60	319.85	2403.50	4177.75
SUT	SUT751	0.2050591	0.5890396	0.7135843	2.15	2.52	20.00	31.50
ORF-T	YKL220C	0.2050798	0.4316277	0.586042	17.67	20.45	168.50	268.50
SRT	SRT412	0.205127	0.6563871	0.7653471	0.94	1.07	8.75	14.50
SRT	SRT123	0.2055831	0.5250651	0.6630048	3.15	3.68	30.50	49.25
ORF-T	YKR103W	0.2059428	0.461149	0.6106667	13.88	15.99	126.00	209.50
ORF-T	YER185W	0.2059887	0.6024099	0.7247425	3.97	4.63	38.25	59.75
sn/snoRNA	SNR51	0.2062047	0.5072197	0.6487574	252.99	291.86	2264.75	3745.00
CUT	CUT406	0.2062155	0.6225534	0.7389729	2.27	2.67	23.00	36.00
ORF-T	YNL141W	0.2063356	0.5116273	0.6521615	80.05	92.39	746.25	1196.75
ORF-T	YBR085C-A	0.207427	0.5085527	0.649803	92.36	106.68	829.50	1307.75
SUT	SUT025	0.2081244	0.5327847	0.6691356	3.18	3.68	29.50	48.25
ORF-T	YMR030W-A	0.2082264	0.6888186	0.7888158	1.25	1.42	11.00	19.25
ORF-T	YLR317W	0.2087102	0.680471	0.7818903	0.97	1.08	8.25	14.50
ORF-T	YKR046C	0.2089239	0.5122526	0.6524851	7.97	9.19	70.50	118.00
SUT	SUT484	0.2091343	0.6077132	0.7283313	1.96	2.22	17.50	30.75
ORF-T	YOR152C	0.2096855	0.5790303	0.7053566	3.75	4.27	32.00	56.00
ORF-T	YGL236C	0.209995	0.3909443	0.5519298	41.41	47.96	392.75	630.25

TABLE S1: Differential expression data for RRP6 RNA-Seq dataset Page 218

Class	Transcript name	RRP6 KO_vs_WT log2_fold _change	RRP6 KO_vs_WT p-value	RRP6 KO_vs_WT FDR	Ave Norm Reads in WT	Ave Norm Reads in RRP6	Average RAW read counts in WT	Average RAW read counts in RRP6
SUT	SUT070	0.2101485	0.6746955	0.7781802	1.29	1.44	10.75	19.00
SUT	SUT098	0.2101714	0.539589	0.6733344	6.95	7.95	58.00	103.50
NUT	NUT0608	0.2102086	0.6666125	0.7726575	31.46	36.39	269.75	436.75
NUT	NUT0492	0.210549	0.6094973	0.7290831	1.99	2.28	18.25	31.25
NUT	NUT0491	0.2105517	0.6094582	0.7290831	1.99	2.28	18.25	31.25
ORF-T	YDR183C-A	0.2109972	0.6254737	0.7416264	1.72	1.98	15.75	26.50
ORF-T	YJR136C	0.2113687	0.4178195	0.5743521	18.51	21.47	170.25	275.75
ORF-T	YGR122C-A	0.2121546	0.6608623	0.7688205	1.14	1.36	10.75	17.00
NUT	NUT1007	0.2123578	0.3859959	0.5470671	41.46	48.10	393.25	632.00
ORF-T	YMR034C	0.2124531	0.609893	0.7292495	8.87	10.33	83.75	128.75
ORF-T	YGR087C	0.212737	0.6655303	0.771758	1.45	1.69	14.25	23.75
ORF-T	YOL159C-A	0.2128904	0.4732333	0.6208801	7.57	8.76	67.00	111.00
SUT	SUT339	0.2129109	0.498618	0.6412529	16.44	19.02	152.50	264.25
NUT	NUT0698	0.212911	0.7065395	0.8020651	0.72	0.83	6.50	10.75
ORF-T	YLR165C	0.2129989	0.4179218	0.5743521	11.32	13.19	107.50	172.00
NUT	NUT1307	0.2134497	0.4944605	0.6377769	5.45	6.36	50.75	81.25
SUT	SUT193	0.2135157	0.5635869	0.6929054	5.02	5.86	47.50	76.25
ORF-T	YNL211C	0.2136515	0.5014753	0.6438601	7.35	8.56	68.00	110.00
ORF-T	YIL047C-A	0.2144254	0.4151254	0.571875	43.67	50.73	414.25	658.50
NUT	NUT1525	0.2150805	0.4402747	0.5933881	39.76	46.12	359.25	609.00
ORF-T	YBR114W	0.215082	0.4051015	0.5637653	95.19	110.61	913.75	1415.00
NUT	NUT1187	0.2155765	0.4737507	0.6212702	8.34	9.70	77.25	126.50
ORF-T	YMR009W	0.2156676	0.5508857	0.682281	3.72	4.27	32.75	56.75
ORF-T	YEL023C	0.2156757	0.4880007	0.6329259	6.58	7.59	59.00	101.50
ORF-T	YKL120W	0.21604	0.5604606	0.690493	68.85	80.01	629.75	974.50
SUT	SUT314	0.216154	0.5341148	0.6700689	3.32	3.91	31.25	50.00
SUT	SUT238	0.2162099	0.5370962	0.6718866	2.75	3.15	24.00	41.50
SUT	SUT290	0.2175502	0.6560339	0.7651453	0.95	1.04	7.75	14.00
SUT	SUT160	0.2181562	0.6609411	0.7688234	1.17	1.36	11.00	18.75

Class	Transcript name	RRP6 KO_vs_WT log2_fold_change	RRP6 KO_vs_WT p-value	RRP6 KO_vs_WT FDR	Ave Norm Reads in WT	Ave Norm Reads in RRP6	Average RAW read counts in WT	Average RAW read counts in RRP6
ORF-T	YGR036C	0.2189038	0.4108812	0.5682016	27.28	31.78	251.75	414.00
ORF-T	YBR196C-B	0.2198767	0.53284	0.6691356	2.65	3.13	25.50	41.25
AST	AS_ADH2	0.2204343	0.5110488	0.6515888	8.32	9.70	77.25	128.50
AST	AS_YML122C	0.2205457	0.6148329	0.7333777	1.68	1.94	15.75	27.00
CUT	CUT329	0.2206206	0.575695	0.7023966	2.35	2.79	21.75	34.25
SUT	SUT024	0.2228711	0.6700047	0.7746553	9.72	11.40	97.25	147.00
ORF-T	YLR348C	0.2229867	0.4687143	0.6174378	31.27	36.52	282.25	449.25
AST	AS_YHR180C-B	0.2233847	0.6373833	0.7513002	0.98	1.12	8.25	14.00
SUT	SUT523	0.2238079	0.6664878	0.7726018	1.56	1.87	14.50	22.25
ORF-T	YMR023C	0.2240824	0.4139623	0.5705845	27.58	32.26	253.50	408.75
SUT	SUT447	0.224638	0.6430725	0.7559153	3.09	3.60	29.75	52.00
SUT	SUT036	0.2246807	0.5845686	0.7099316	2.29	2.62	20.25	36.25
SUT	SUT717	0.2249035	0.4635496	0.6130405	5.49	6.38	50.25	87.25
ORF-T	YGL204C	0.2252585	0.5825554	0.7087089	4.71	5.46	40.75	72.50
ORF-T	YDR111C	0.2255865	0.4127929	0.5699314	74.79	87.51	691.00	1097.75
NUT	NUT0215	0.2260453	0.5525836	0.6836282	4.73	5.60	45.50	71.75
ORF-T	YJL200C	0.2260485	0.5285939	0.6657825	156.30	182.85	1532.50	2518.00
ORF-T	YNL112W	0.2260825	0.4878033	0.6327775	862.92	1009.37	8153.50	13099.75
ORF-T	YOL099C	0.2268345	0.5182258	0.6574125	2.54	2.96	23.00	39.00
ORF-T	YFL027C	0.2273314	0.3482672	0.5131506	39.41	46.18	371.50	607.50
ORF-T	YKL176C	0.2278224	0.4015883	0.5608572	238.44	279.30	2242.50	3570.25
ORF-T	YMR105C	0.2278877	0.7093705	0.8035601	163.20	191.11	1319.75	2242.50
ORF-T	YER041W	0.2278882	0.3619383	0.5259057	25.02	29.34	233.00	384.75
NUT	NUT0894	0.2279482	0.6504427	0.7604714	17.94	20.96	144.75	258.50
SUT	SUT296	0.2279622	0.5083736	0.6496565	3.04	3.57	27.50	45.25
SUT	SUT438	0.2280782	0.440443	0.5934368	5.57	6.48	51.00	88.25
CUT	CUT162	0.2282918	0.6097908	0.7292495	1.18	1.39	11.00	18.00
SRT	SRT24	0.2283866	0.6626921	0.7699796	9.72	11.44	97.25	147.50
SUT	SUT020	0.2295034	0.3766516	0.5384266	24.19	28.42	228.25	369.75

Class	Transcript name	RRP6 KO_vs_WT log2_fold _change	RRP6 KO_vs_WT p-value	RRP6 KO_vs_WT FDR	Ave Norm Reads in WT	Ave Norm Reads in RRP6	Average RAW read counts in WT	Average RAW read counts in RRP6
NUT	NUT0214	0.2297682	0.5016112	0.6439526	6.95	8.06	58.00	105.00
ORF-T	YIL086C	0.2299202	0.5002295	0.6425059	3.68	4.30	33.00	55.75
NUT	NUT1139	0.2300209	0.4791153	0.625181	24.54	28.84	227.25	361.25
NUT	NUT1217	0.2316124	0.5327922	0.6691356	2.07	2.45	19.25	32.00
SUT	SUT563	0.2322152	0.6557679	0.7650119	1.03	1.17	9.00	16.00
ORF-T	YDR333C	0.2339515	0.3330002	0.497183	84.80	99.79	803.00	1312.75
SRT	SRT437	0.2341631	0.7081079	0.8029416	0.97	1.18	9.75	15.00
SUT	SUT220	0.2347196	0.6623737	0.7698682	1.14	1.36	11.25	18.50
ORF-T	YCR028C	0.2348558	0.3781457	0.5397022	126.25	148.63	1174.25	1914.50
AST	AS_YBR224W	0.2359806	0.5745541	0.7018539	1.10	1.26	9.75	16.75
CUT	CUT602	0.2362123	0.6512028	0.7610952	6.72	7.92	57.00	93.50
ORF-T	YHL016C	0.2362154	0.5114141	0.6519721	20.22	23.84	187.75	311.75
ORF-T	YEL028W	0.2366389	0.4560097	0.6063293	4.68	5.46	41.50	72.25
AST	AS_YEL062W	0.2366857	0.7351421	0.8241438	0.84	0.98	8.00	14.50
SUT	SUT825	0.236696	0.5602848	0.690493	1.82	2.08	15.50	28.00
ORF-T	YML128C	0.2374303	0.5770728	0.7035313	213.65	251.87	1832.00	2973.00
NUT	NUT0519	0.2379587	0.3738699	0.5358305	10.40	12.24	95.25	163.00
ORF-T	YNR038W	0.2380081	0.4795162	0.6254707	30.63	36.05	277.25	506.00
ORF-T	YOR349W	0.2382375	0.5249997	0.6630048	4.85	5.69	41.50	71.50
SUT	SUT131	0.2385418	0.5342097	0.6700817	6.44	7.66	59.50	94.00
ORF-T	YNL214W	0.2389645	0.4985795	0.6412529	3.89	4.55	35.00	62.25
SUT	SUT605	0.2390628	0.5850814	0.7102397	2.99	3.58	27.50	42.00
NUT	NUT0092	0.2398599	0.3673048	0.5297989	16.25	19.20	148.00	249.50
SUT	SUT705	0.2399331	0.6432814	0.7559153	1.06	1.26	10.00	16.50
ORF-T	YDR275W	0.2400982	0.3886129	0.5496175	87.80	103.78	835.50	1349.00
ORF-T	YPL201C	0.2416453	0.5754977	0.7022409	2.39	2.86	24.00	39.00
ORF-T	YOR049C	0.2419024	0.5910516	0.71542	2.84	3.31	23.25	41.50
SUT	SUT648	0.2427031	0.6304867	0.7457219	1.09	1.24	9.25	16.75
SUT	SUT059	0.2430808	0.4640655	0.6134054	4.66	5.54	43.75	73.25

TABLE S1: Differential expression data for RRP6 RNA-Seq dataset Page 221

Class	Transcript name	RRP6 KO_vs_WT log2_fold _change	RRP6 KO_vs_WT p-value	RRP6 KO_vs_WT FDR	Ave Norm Reads in WT	Ave Norm Reads in RRP6	Average RAW read counts in WT	Average RAW read counts in RRP6
sn/snoRNA	SNR68	0.2435289	0.4987628	0.6413573	252.66	299.08	2153.75	3777.25
SUT	SUT628	0.2437693	0.4937474	0.6373387	2.30	2.68	20.25	35.25
SRT	SRT23	0.2442543	0.6384835	0.7518939	0.71	0.85	6.25	10.25
SRT	SRT325	0.2444372	0.7444274	0.8309504	0.52	0.62	5.50	9.50
CUT	CUT410	0.2444424	0.4659361	0.6151479	3.64	4.38	35.25	56.25
NUT	NUT1192	0.2448044	0.3559154	0.5199127	13.34	15.86	124.00	202.50
ORF-T	YIL173W	0.2451752	0.6598519	0.7681767	0.75	0.85	6.50	12.00
ORF-T	YBR045C	0.2453368	0.5963156	0.7199768	2.27	2.73	21.50	34.25
SRT	SRT87	0.2454264	0.6150002	0.7334037	1.16	1.42	10.75	16.75
ORF-T	YLR462W	0.2458084	0.6625152	0.7699439	1.55	1.84	13.75	23.50
SRT	SRT217	0.246124	0.5331665	0.6691938	1.28	1.57	12.50	20.50
AST	AS_YLR233C	0.2463126	0.5200989	0.6586702	2.88	3.40	25.75	44.75
SUT	SUT139	0.2470014	0.4848168	0.6302556	2.23	2.65	20.50	34.75
ORF-T	YDR323C	0.2483997	0.3302472	0.4948311	13.18	15.65	121.00	207.75
SUT	SUT229	0.248712	0.3870632	0.5479946	13.71	16.38	133.25	217.00
SUT	SUT008	0.2491271	0.5578389	0.6884405	1.51	1.79	13.25	22.50
NUT	NUT0115	0.249277	0.6220262	0.7387114	0.64	0.73	5.75	10.00
SUT	SUT554	0.2509549	0.5364019	0.6716013	2.31	2.74	20.00	33.75
ORF-T	YBR163W	0.2511551	0.3115282	0.476772	23.53	28.04	220.00	367.50
ORF-T	YGR225W	0.2511925	0.4761131	0.6233608	3.14	3.76	29.00	48.50
ORF-T	YMR017W	0.2519482	0.6135392	0.7325277	1.64	1.96	15.50	26.25
AST	AS_YNL276C	0.2534068	0.6700262	0.7746553	3.43	4.10	31.00	48.50
ORF-T	YJL214W	0.2539847	0.5659318	0.6948565	1.27	1.57	12.50	20.00
ORF-T	YGL016W	0.2549709	0.3393397	0.5038877	237.52	283.50	2236.25	3649.50
ORF-T	YLR237W	0.2552366	0.4184884	0.5743891	50.03	59.76	463.00	753.00
SUT	SUT542	0.2557723	0.5059181	0.6475032	6.87	8.22	64.75	107.50
CUT	CUT141	0.2559477	0.488922	0.6336157	1.88	2.23	17.00	29.50
ORF-T	YCL038C	0.2572879	0.3396876	0.50433	32.68	39.09	295.25	497.75
SUT	SUT452	0.2580634	0.378824	0.540263	23.35	27.91	209.00	360.50

Class	Transcript name	RRP6 KO_vs_WT log2_fold _change	RRP6 KO_vs_WT p-value	RRP6 KO_vs_WT FDR	Ave Norm Reads in WT	Ave Norm Reads in RRP6	Average RAW read counts in WT	Average RAW read counts in RRP6
CUT	CUT856	0.2589176	0.6567031	0.7653471	0.77	0.89	6.50	11.75
SUT	SUT577	0.2590175	0.5882471	0.7129405	1.26	1.54	11.50	18.50
SUT	SUT103	0.2590415	0.3092748	0.4751984	29.23	34.91	260.00	459.75
sn/snoRNA	SNR5	0.2592005	0.4813886	0.6274166	766.59	917.47	6695.75	11454.75
CUT	CUT557	0.2597812	0.5319776	0.6684945	2.00	2.38	18.00	31.25
ORF-T	YEL072W	0.2598291	0.4481894	0.5998861	3.04	3.57	26.25	47.50
SUT	SUT724	0.2601636	0.5198413	0.6586412	3.32	3.91	29.75	56.50
sn/snoRNA ET	SNR40-ET	0.2603625	0.4767802	0.6237998	28.81	34.49	250.50	430.25
ORF-T	YLR113W	0.2606999	0.3113158	0.4766638	151.68	181.75	1441.00	2463.75
SRT	SRT339	0.2609346	0.6821362	0.7832954	0.84	1.04	8.50	13.50
ORF-T	YML047W-A	0.261028	0.546602	0.6788933	1.88	2.23	17.00	30.00
NUT	NUT0738	0.261458	0.474403	0.6217675	776.77	931.10	6786.50	11633.25
ORF-T	YNR034W-A	0.2636041	0.7123588	0.8062206	11.89	14.24	93.75	168.50
ORF-T	YPR015C	0.2636172	0.514446	0.654368	1.71	2.05	15.50	26.25
ORF-T	YBL100W-C	0.2640702	0.6323356	0.7474472	1.39	1.67	12.00	20.00
NUT	NUT0202	0.2650983	0.4488495	0.6002119	4.75	5.77	46.00	74.75
ORF-T	YDR508C	0.2653521	0.2786051	0.4422944	176.59	212.26	1615.75	2759.25
NUT	NUT0664	0.2655245	0.7052688	0.8011638	12.06	14.47	95.25	171.75
ORF-T	YBR270C	0.2656686	0.3741854	0.5361304	9.59	11.54	89.50	154.25
SUT	SUT775	0.2657407	0.5759608	0.702636	1.41	1.70	13.00	22.00
ORF-T	YJL205C	0.2659155	0.4184513	0.5743891	3.31	4.00	30.25	50.75
ORF-T	YOL011W	0.2661274	0.2831086	0.446911	84.93	102.14	774.00	1316.25
AST	AS_YNL074C	0.266926	0.6481936	0.7591619	1.18	1.43	11.50	20.00
NUT	NUT1251	0.2673563	0.3473673	0.5121878	26.52	31.96	249.00	420.50
SUT	SUT675	0.2683061	0.5104732	0.6512666	2.21	2.65	20.25	35.25
NUT	NUT0010	0.2684245	0.5405251	0.6740021	1.98	2.34	16.50	29.50
SUT	SUT354	0.2686357	0.5970196	0.720568	0.76	0.94	7.00	11.50
ORF-T	YMR316W	0.2688094	0.3429542	0.5074363	96.42	116.26	903.50	1449.25
ORF-T	YPL025C	0.2689809	0.4971993	0.6402005	3.70	4.47	35.25	61.50

Class	Transcript name	RRP6 KO_vs_WT log2_fold _change	RRP6 KO_vs_WT p-value	RRP6 KO_vs_WT FDR	Ave Norm Reads in WT	Ave Norm Reads in RRP6	Average RAW read counts in WT	Average RAW read counts in RRP6
SUT	SUT500	0.2696976	0.3850348	0.5460887	8.09	9.71	71.50	127.00
ORF-T	YKL017C	0.2697513	0.3052108	0.4706637	28.28	34.05	258.75	455.50
ORF-T	YJL023C	0.2706083	0.3660278	0.5286486	7.91	9.53	70.25	122.00
ORF-T	YGL085W	0.2708068	0.3697987	0.5324172	14.33	17.38	137.00	220.50
NUT	NUT0227	0.2717085	0.6157746	0.7339801	103.02	124.38	859.00	1376.75
ORF-T	YAL062W	0.2719509	0.5750423	0.702025	30.66	37.07	290.00	460.50
ORF-T	YFL014W	0.2719972	0.6154371	0.7336645	103.02	124.40	859.00	1377.00
ORF-T	YPL263C	0.2721845	0.2851697	0.4486896	145.14	175.35	1372.50	2276.25
ORF-T	YCL048W-A	0.2725182	0.6299685	0.7454357	0.78	0.99	7.50	12.00
SUT	SUT096	0.2725473	0.4263452	0.5815319	4.86	5.93	47.00	76.75
SUT	SUT304	0.2727134	0.6134532	0.7325277	0.87	0.99	7.00	13.25
ORF-T	YGR122W	0.2728861	0.3267106	0.4912107	15.63	18.86	139.25	241.50
ORF-T	YIL108W	0.2734617	0.319555	0.4845481	101.59	122.88	974.00	1599.75
ORF-T	YOR121C	0.2735699	0.3646419	0.5279324	10.16	12.27	93.50	162.75
NUT	NUT0167	0.2741569	0.4020107	0.5608572	303.82	367.39	2688.50	4622.50
ORF-T	YOL141W	0.2748106	0.3360782	0.5004448	29.96	36.30	292.75	493.25
NUT	NUT1414	0.2750049	0.5615294	0.6911426	3.77	4.53	32.25	56.75
ORF-T	YOR108W	0.27513	0.4097486	0.5673356	109.62	132.63	1050.50	1878.25
ORF-T	YNL029C	0.2757394	0.3037678	0.4694551	42.01	50.99	399.25	639.25
ORF-T	YMR102C	0.2761016	0.3119162	0.4772209	72.04	87.28	663.75	1101.00
AST	AS_YOR267C	0.2790127	0.6888449	0.7888158	0.62	0.71	5.25	10.75
sn/snoRNA	SNR13	0.2793183	0.4182529	0.5743891	273.60	332.03	2405.50	4148.00
ORF-T	YNL261W	0.2793912	0.2644286	0.4271865	31.48	38.21	291.25	510.00
SRT	SRT237	0.2800968	0.6107149	0.7298891	0.59	0.74	5.75	9.75
AST	AS_YDL048C	0.2809919	0.5855543	0.7104716	1.03	1.25	9.75	17.25
ORF-T	YIL050W	0.2813722	0.3729955	0.5351859	7.19	8.66	63.25	115.75
sn/snoRNA ET	NR7-L, SNR7-S RT	0.2824664	0.3764592	0.5382411	29.77	36.17	254.00	452.25
NUT	NUT0130	0.2827817	0.3862292	0.5471671	5.13	6.27	48.00	82.00
ORF-T	YIL024C	0.2838203	0.3213085	0.485725	11.18	13.67	107.50	180.75

Class	Transcript name	RRP6 KO_vs_WT log2_fold _change	RRP6 KO_vs_WT p-value	RRP6 KO_vs_WT FDR	Ave Norm Reads in WT	Ave Norm Reads in RRP6	Average RAW read counts in WT	Average RAW read counts in RRP6
ORF-T	YDR007W	0.2840375	0.2943863	0.4590369	67.62	82.45	636.75	1020.50
AST	AS_YGR075C	0.2840905	0.3706731	0.5332954	4.97	6.07	46.25	79.50
SUT	SUT063	0.2840979	0.2800583	0.4437631	11.19	13.61	101.50	178.75
ORF-T	YOR107W	0.2842402	0.4348221	0.5891083	11.29	13.72	102.00	181.50
SUT	SUT698	0.2842635	0.6111562	0.7302878	2.02	2.48	19.00	31.75
NUT	NUT0863	0.2856428	0.4322392	0.5867932	3.70	4.53	34.25	59.75
SUT	SUT019	0.2857416	0.5573626	0.6879491	0.80	0.95	7.00	12.50
ORF-T	YLL059C	0.2858337	0.4209948	0.5762606	4.94	6.06	47.50	80.50
ORF-T	YMR025W	0.2863346	0.4106938	0.5681762	5.83	7.02	49.75	93.75
SUT	SUT841	0.2868834	0.3702583	0.5328507	4.00	4.91	37.50	64.75
SRT	SRT466	0.2877991	0.6074001	0.7281194	4.96	6.11	46.75	70.00
ORF-T	YJL059W	0.2879118	0.4370743	0.5907327	3.06	3.66	26.00	47.50
NUT	NUT1022	0.2880831	0.348714	0.5134175	5.55	6.70	49.50	90.75
ORF-T	YOR032C	0.2893548	0.5539371	0.6847146	3.57	4.33	30.50	54.25
NUT	NUT0744	0.2897122	0.3428636	0.5074019	18.96	23.23	176.50	296.75
ORF-T	YHR069C	0.2899036	0.2564632	0.4181365	69.04	84.48	665.75	1122.75
ORF-T	YMR040W	0.2899393	0.3804573	0.5417622	4.16	5.14	38.75	65.00
ORF-T	YDR467C	0.2918706	0.4276853	0.5827292	1.54	1.87	14.00	24.75
AST	AS_YDR409W	0.2927136	0.4179974	0.5743521	4.86	6.00	46.75	79.50
sn/snoRNA ET	SNR78-ET	0.2930039	0.4177274	0.5743521	112.88	138.31	978.75	1677.50
ORF-T	YJL151C	0.2932644	0.2277446	0.3846862	41.15	50.37	367.00	661.00
SUT	SUT490	0.2937216	0.4207144	0.5761896	4.70	5.81	45.00	76.50
AST	AS_YBR280C	0.2948718	0.4802311	0.6263127	1.23	1.51	11.50	20.50
ORF-T	YBR175W	0.2953437	0.2818035	0.4454604	38.22	46.92	366.00	643.75
SUT	SUT579	0.2956023	0.3283196	0.4927843	5.83	7.08	52.25	97.00
ORF-T	YER046W	0.2957284	0.2795739	0.4434136	11.22	13.84	106.50	180.00
ORF-T	YNL336W	0.2965605	0.3617497	0.525784	6.87	8.46	61.75	106.25
ORF-T	YKL038W	0.2965754	0.2591109	0.4212276	62.38	76.58	543.00	960.25
SUT	SUT168	0.2969329	0.5066995	0.6483388	1.22	1.52	11.50	19.75

Class	Transcript name	RRP6 KO_vs_WT log2_fold _change	RRP6 KO_vs_WT p-value	RRP6 KO_vs_WT FDR	Ave Norm Reads in WT	Ave Norm Reads in RRP6	Average RAW read counts in WT	Average RAW read counts in RRP6
ORF-T	YKL219W	0.2969715	0.2742699	0.437603	16.07	19.73	143.25	252.00
NUT	NUT1522	0.2971137	0.3049498	0.4706327	17.03	20.90	152.00	269.00
ORF-T	YER028C	0.2971685	0.5749623	0.7020123	2.12	2.61	20.25	36.25
ORF-T	YPR193C	0.2974738	0.375053	0.5369157	3.72	4.60	34.50	59.25
NUT	NUT0713	0.2978696	0.4126232	0.5699072	3.36	4.17	33.00	57.25
ORF-T	YDR228C	0.298488	0.2882657	0.4518687	12.92	15.82	116.00	211.25
NUT	NUT0210	0.2991762	0.4503285	0.601854	13.20	16.22	111.25	199.50
ORF-T	YCR021C	0.299639	0.63383	0.7484614	24.75	30.42	192.50	386.00
NUT	NUT0577	0.3011297	0.3434131	0.5078424	4.89	6.06	45.25	77.50
SUT	SUT048	0.3015056	0.4760022	0.6233283	1.49	1.84	13.50	23.75
SUT	SUT581	0.3015206	0.3541459	0.5186939	5.14	6.30	46.25	83.75
ORF-T	YPL258C	0.3015688	0.3710597	0.5336993	6.10	7.51	54.25	96.00
NUT	NUT1417	0.3015735	0.3548549	0.5193995	199.66	246.06	1736.25	3109.25
SUT	SUT647	0.3026566	0.5834537	0.7091174	4.97	6.18	46.75	71.00
ORF-T	YIL046W-A	0.3027312	0.5960361	0.7197256	1.23	1.54	12.25	21.00
ORF-T	YBR157C	0.3045957	0.256351	0.4180213	14.83	18.26	133.25	241.00
CUT	CUT003	0.3051304	0.4439546	0.5963216	7.73	9.60	71.00	119.00
ORF-T	YHR094C	0.305465	0.5244232	0.6626092	63.03	77.89	604.50	1104.25
SUT	SUT505	0.3055392	0.4381361	0.5916921	2.45	3.05	22.75	39.00
ORF-T	YDL171C	0.3055742	0.3169314	0.4823747	624.99	772.45	5792.25	9933.25
ORF-T	YDR387C	0.3056264	0.2071017	0.3594209	43.10	53.31	394.00	677.25
NUT	NUT0973	0.3057845	0.4242074	0.5793339	2.27	2.80	20.50	36.50
sn/snoRNA	SNR85	0.3066573	0.4103319	0.5678313	1320.53	1633.27	11471.25	20229.25
ORF-T	YDL175C	0.3067567	0.2733709	0.4366602	9.58	11.83	88.75	159.75
NUT	NUT0565	0.3079921	0.3794103	0.5407737	3.45	4.29	31.50	54.75
NUT	NUT0864	0.3089178	0.6186766	0.7362208	24.87	30.76	193.75	390.25
AST	AS_YGR216C	0.3090719	0.4605378	0.6101653	1.76	2.19	17.00	30.25
ORF-T	YHR096C	0.3093751	0.4164219	0.5730986	13.10	16.31	124.00	202.00
ORF-T	YCR007C	0.3093753	0.4129798	0.5699314	8.81	10.97	81.75	138.25

Class	Transcript name	RRP6 KO_vs_WT log2_fold _change	RRP6 KO_vs_WT p-value	RRP6 KO_vs_WT FDR	Ave Norm Reads in WT	Ave Norm Reads in RRP6	Average RAW read counts in WT	Average RAW read counts in RRP6
ORF-T	YDR046C	0.3095669	0.447532	0.5993646	178.46	221.16	1588.75	2752.00
SUT	SUT377	0.3104814	0.2124817	0.3661423	16.12	20.00	148.75	262.25
AST	AS_YPR123C	0.3108912	0.5307155	0.6675484	29.06	36.01	262.25	503.50
CUT	CUT091	0.3113636	0.4439856	0.5963216	3.62	4.45	32.00	60.00
ORF-T	YPR059C	0.3115941	0.5362002	0.6715989	1.46	1.87	15.00	24.75
ORF-T	YDL049C	0.3120747	0.2208413	0.3770041	15.47	19.16	138.75	252.00
SUT	SUT288	0.3130936	0.3713831	0.5340882	3.45	4.30	31.50	55.00
SUT	SUT761	0.3140275	0.3150221	0.4805147	6.87	8.58	65.25	112.75
ORF-T	YHL047C	0.3148677	0.2507023	0.4123025	41.95	52.27	393.25	665.25
SUT	SUT108	0.3157441	0.5170121	0.6563426	2.18	2.74	21.50	36.75
SUT	SUT685	0.3158095	0.5055703	0.6473777	1.02	1.31	10.25	17.50
ORF-T	YMR319C	0.3160705	0.2612285	0.4236455	76.94	95.72	702.75	1319.50
SRT	SRT40	0.3168293	0.5494698	0.6809455	0.66	0.85	6.50	11.25
NUT	NUT1228	0.3168711	0.3932242	0.554351	2.58	3.21	24.00	43.00
ORF-T	YIL164C	0.3175112	0.2251594	0.3817904	18.13	22.66	170.75	295.75
SUT	SUT107	0.3178166	0.3343468	0.4984554	5.49	6.85	50.50	88.75
ORF-T	YOL152W	0.3184627	0.5383487	0.672581	2.81	3.49	26.75	49.50
AST	AS_YIL140W	0.3196089	0.5122191	0.6524851	1.45	1.78	13.00	25.25
NUT	NUT0968	0.3200557	0.5424344	0.6754641	2.12	2.65	20.25	36.75
ORF-T	YPR048W	0.3212202	0.2354156	0.3942097	23.76	29.77	231.50	393.25
NUT	NUT0241	0.3220539	0.4426598	0.5951725	2.22	2.73	18.75	35.75
SUT	SUT032	0.3232264	0.305201	0.4706637	5.16	6.39	45.75	85.50
NUT	NUT0045	0.3235016	0.4974013	0.6402598	0.83	1.01	7.25	13.25
ORF-T	YBR255C-A	0.3236086	0.376275	0.5380541	3.76	4.67	32.50	59.25
NUT	NUT0017	0.3239447	0.4056229	0.5640999	1.84	2.32	16.75	29.50
ORF-T	YER137C	0.3248195	0.3715968	0.5341207	2.16	2.73	19.75	34.25
ORF-T	YPL041C	0.325077	0.3471649	0.512039	3.39	4.20	30.25	56.00
SUT	SUT624	0.3254076	0.442661	0.5951725	1.18	1.41	10.00	19.00
ORF-T	YIR005W	0.3259375	0.3159148	0.4814394	8.09	10.15	73.75	129.00

Class	Transcript name	RRP6 KO_vs_WT log2_fold _change	RRP6 KO_vs_WT p-value	RRP6 KO_vs_WT FDR	Ave Norm Reads in WT	Ave Norm Reads in RRP6	Average RAW read counts in WT	Average RAW read counts in RRP6
sn/snoRNA ET	SNR17B-ET	0.3260843	0.284515	0.4480147	33.85	42.37	298.00	567.00
AST	AS_YKL076C	0.3287725	0.4314545	0.5859646	2.21	2.79	20.25	36.25
NUT	NUT0522	0.3291319	0.4711676	0.6196211	1.29	1.54	11.00	22.25
ORF-T	YLR214W	0.3303182	0.5270369	0.6644251	98.67	124.01	881.25	1804.75
ORF-T	YGL224C	0.3304257	0.2269393	0.3835829	10.78	13.64	104.50	180.00
ORF-T	YJR119C	0.3308339	0.1815403	0.3295904	24.96	31.40	231.00	417.50
AST	AS_YDR069C	0.3315686	0.5348922	0.6707104	2.05	2.59	20.00	36.25
SUT	SUT320	0.3324546	0.4275442	0.5826944	1.70	2.16	16.25	29.00
CUT	CUT290	0.3327958	0.4828068	0.6286151	1.31	1.69	12.75	21.75
ORF-T	YPR124W	0.3329994	0.4687598	0.6174378	68.82	86.64	623.75	1209.75
ORF-T	YNL204C	0.3332058	0.3709221	0.5335776	3.43	4.35	33.25	58.75
NUT	NUT1327	0.333571	0.4606316	0.6101653	14.10	17.86	145.75	233.75
NUT	NUT0376	0.3341003	0.4840468	0.6295793	0.95	1.17	8.25	15.25
SUT	SUT089	0.334308	0.5159242	0.6556184	1.70	2.16	15.50	27.50
SUT	SUT041	0.3360135	0.4092776	0.5670908	3.46	4.35	33.00	61.50
NUT	NUT0893	0.3377327	0.1823354	0.3305178	15.50	19.54	139.00	256.75
SUT	SUT584	0.3379044	0.3595054	0.5232754	2.67	3.38	24.50	44.25
SUT	SUT710	0.3393254	0.3696548	0.532362	3.67	4.67	33.50	59.25
ORF-T	YOR222W	0.3397263	0.1707042	0.3153131	62.70	79.32	579.00	1068.00
SUT	SUT457	0.3398287	0.3333857	0.4973229	6.39	8.13	58.75	101.50
CUT	CUT829	0.3400819	0.5996569	0.7229445	0.56	0.68	4.75	9.00
ORF-T	YKR104W	0.3418876	0.3226367	0.4868709	3.29	4.18	30.25	55.25
SUT	SUT736	0.3419418	0.4208856	0.5762606	1.36	1.66	11.75	22.50
ORF-T	YPL189W	0.3425334	0.4007755	0.5605298	2.18	2.77	20.75	37.25
ORF-T	YOL052C-A	0.3433313	0.5123672	0.6524851	8.95	11.34	73.00	130.00
SUT	SUT756	0.3433448	0.3377979	0.5021899	3.09	3.94	28.00	49.75
ORF-T	YPR194C	0.3438356	0.2827075	0.4464875	22.21	28.14	195.00	364.00
ORF-T	YAL033W	0.3467709	0.1878426	0.3369272	27.37	34.91	264.50	452.75
SUT	SUT752	0.3472791	0.44451	0.5967038	1.55	1.99	14.50	25.75

Class	Transcript name	RRP6 KO_vs_WT log2_fold _change	RRP6 KO_vs_WT p-value	RRP6 KO_vs_WT FDR	Ave Norm Reads in WT	Ave Norm Reads in RRP6	Average RAW read counts in WT	Average RAW read counts in RRP6
ORF-T	YKL221W	0.3475652	0.3529532	0.5176236	1.91	2.47	18.25	32.00
ORF-T	YHR033W	0.3483786	0.1920747	0.3416016	35.96	45.84	341.25	608.50
ORF-T	YBR183W	0.3484371	0.4567391	0.606899	38.40	48.86	323.50	588.50
ORF-T	YNR050C	0.3489387	0.3529279	0.5176236	289.83	369.18	2819.00	5002.75
ORF-T	YIR031C	0.3505025	0.1705829	0.3151627	28.39	36.24	264.50	475.50
AST	AS_YMR167W	0.3524695	0.2418478	0.4015796	5.89	7.51	54.50	100.25
CUT	CUT495	0.353077	0.4129247	0.5699314	3.27	4.19	30.50	54.00
ORF-T	YMR081C	0.3537949	0.3770116	0.5385191	11.33	14.41	92.75	182.75
AST	AS_YOL164W-A	0.3540773	0.4667663	0.6158408	0.82	1.04	7.25	13.25
SUT	SUT297	0.3549591	0.4366375	0.5903006	4.07	5.23	38.75	69.00
SUT	SUT504	0.3550273	0.3261574	0.4907448	14.34	18.32	133.75	251.50
ORF-T	YER038C	0.355042	0.2413646	0.4010414	8.40	10.67	75.50	147.00
SUT	SUT204	0.3558397	0.2678631	0.4307734	9.60	12.24	88.00	170.25
NUT	NUT0387	0.3558429	0.2677178	0.4307092	9.60	12.24	88.00	170.25
ORF-T	YER071C	0.3563642	0.2043277	0.3559234	9.51	12.23	90.00	160.25
SUT	SUT342	0.3563725	0.3519277	0.5167208	2.82	3.64	27.00	49.00
ORF-T	YCR037C	0.3576625	0.152842	0.291905	123.36	158.12	1163.25	2057.25
ORF-T	YJL043W	0.3583574	0.4542417	0.6050092	0.93	1.20	8.50	15.50
ORF-T	YDR263C	0.3590386	0.2829751	0.4467702	7.23	9.25	64.50	122.25
CUT	CUT122	0.3591313	0.4748215	0.6220736	1.21	1.59	11.00	18.50
SUT	SUT194	0.3608223	0.4825971	0.6285043	0.71	0.92	6.25	11.25
ORF-T	YMR258C	0.3612173	0.1599584	0.3018377	89.48	114.99	823.50	1460.50
SUT	SUT440	0.3619215	0.310045	0.4757228	2.59	3.39	24.75	43.75
NUT	NUT1496	0.3627691	0.4179234	0.5743521	2.35	2.99	21.25	41.00
NUT	NUT0189	0.3628394	0.3258243	0.4904631	3.33	4.26	30.50	58.25
NUT	NUT0439	0.3632267	0.3649958	0.5280657	1.86	2.39	17.00	31.50
NUT	NUT1325	0.3633222	0.1872276	0.3363397	18.72	24.06	167.75	309.25
ORF-T	YKR106W	0.3635868	0.3069616	0.4727968	2.19	2.83	20.25	36.75
NUT	NUT1381	0.3638225	0.2412118	0.4008537	7.12	9.21	67.50	121.75

Class	Transcript name	RRP6 KO_vs_WT log2_fold _change	RRP6 KO_vs_WT p-value	RRP6 KO_vs_WT FDR	Ave Norm Reads in WT	Ave Norm Reads in RRP6	Average RAW read counts in WT	Average RAW read counts in RRP6
ORF-T	YDR195W	0.3640778	0.1437412	0.2803648	18.49	23.78	168.00	307.50
SUT	SUT405	0.3642424	0.4333864	0.5879549	0.72	0.93	6.75	12.50
NUT	NUT0498	0.3666532	0.4486908	0.6001845	2.02	2.63	19.50	35.25
CUT	CUT452	0.3673106	0.398658	0.559197	0.81	1.05	7.25	13.25
ORF-T	YOR028C	0.3673945	0.4369997	0.5907111	1.76	2.29	15.75	27.25
ORF-T	YBL016W	0.367999	0.1169156	0.2402643	34.67	44.69	316.25	599.25
SRT	SRT122	0.3680379	0.5834488	0.7091174	0.67	0.86	6.75	13.25
ORF-T	YOR300W	0.368084	0.3337388	0.4977557	2.51	3.24	23.00	42.50
ORF-T	YNR004W	0.3695506	0.3657748	0.5285144	3.16	4.13	30.25	54.00
ORF-T	YCR018C	0.3709268	0.1687993	0.312862	15.87	20.61	151.25	268.50
ORF-T	YDL239C	0.3710672	0.1708645	0.3154518	15.42	19.96	141.50	258.50
sn/snoRNA ET	SNR17A-ET	0.3711981	0.2408794	0.4005848	26.97	34.83	233.25	438.50
SUT	SUT109	0.3716402	0.3780768	0.5397022	2.40	3.06	20.25	39.75
SUT	SUT268	0.3716908	0.4072172	0.5653832	1.29	1.59	11.00	22.75
ORF-T	YOR034C-A	0.3720599	0.281321	0.4450644	3.14	4.09	29.25	53.00
ORF-T	YMR271C	0.3727554	0.1767361	0.3231626	18.46	23.88	165.50	307.25
ORF-T	SHU2	0.3742039	0.2487168	0.4096799	10.39	13.44	91.25	169.25
sn/snoRNA	SNR74	0.374301	0.2835182	0.4472361	439.29	569.44	3902.00	6930.25
SUT	SUT599	0.3744776	0.2938424	0.4584014	2.13	2.73	19.00	36.25
NUT	NUT0799	0.3746059	0.1168976	0.2402643	40.92	53.03	372.00	695.75
ORF-T	YJL116C	0.3766763	0.4790353	0.6251575	24.09	31.25	201.25	380.25
NUT	NUT1193	0.377032	0.2203079	0.3762846	17.76	23.10	172.25	319.00
SUT	SUT643	0.377034	0.2205374	0.3765491	17.76	23.10	172.25	319.00
ORF-T	YGR129W	0.378047	0.1661827	0.3097321	10.63	13.81	99.25	186.75
SRT	SRT340	0.3793451	0.3642482	0.527817	2.29	3.00	22.00	41.25
SUT	SUT522	0.3796487	0.4523749	0.6034857	1.83	2.41	18.00	31.75
SUT	SUT118	0.3803762	0.4285098	0.5829443	2.95	3.87	27.00	49.25
ORF-T	YML087C	0.3806273	0.2567703	0.4181881	6.51	8.48	58.00	105.25
SUT	SUT446	0.3806864	0.2843988	0.4480147	2.74	3.56	24.25	44.75

Class	Transcript name	RRP6 KO_vs_WT log2_fold _change	RRP6 KO_vs_WT p-value	RRP6 KO_vs_WT FDR	Ave Norm Reads in WT	Ave Norm Reads in RRP6	Average RAW read counts in WT	Average RAW read counts in RRP6
NUT	NUT0568	0.3809874	0.2304292	0.38844	3.90	5.09	36.50	68.25
SUT	SUT156	0.3822479	0.3912226	0.5520695	2.42	3.17	22.50	41.25
SUT	SUT773	0.3842268	0.267119	0.4300172	3.44	4.50	31.75	59.25
ORF-T	YOR120W	0.3844181	0.1316723	0.2630838	24.80	32.39	228.75	423.50
ORF-T	YKR040C	0.3851319	0.4603185	0.6101653	0.73	0.94	6.50	12.25
NUT	NUT1466	0.3855064	0.4180627	0.5743521	0.82	1.11	8.00	14.50
ORF-T	YPL125W	0.3861834	0.1168247	0.2402243	121.26	158.58	1161.00	2072.50
SUT	SUT511	0.3868805	0.2940385	0.4586364	1.91	2.56	18.00	32.00
ORF-T	YLL063C	0.3878823	0.1761448	0.3224516	7.42	9.75	68.75	125.25
NUT	NUT0643	0.3884236	0.4363075	0.5900127	1.40	1.86	13.25	23.50
NUT	NUT0444	0.3885701	0.4022143	0.5610637	1.49	1.93	13.00	24.50
NUT	NUT1517	0.388851	0.1846135	0.3327927	5.73	7.50	52.00	97.00
SUT	SUT822	0.3894258	0.3949658	0.5558934	2.20	2.86	20.00	39.00
CUT	CUT326	0.3904817	0.4946685	0.6379239	0.48	0.63	4.25	8.00
NUT	NUT0261	0.3913662	0.4235942	0.5789542	2.92	3.85	26.75	49.00
CUT	CUT537	0.3915795	0.4009582	0.5605521	4.12	5.40	35.75	66.50
ORF-T	YDL248W	0.3919951	0.2654289	0.4283729	3.53	4.62	31.25	59.25
SUT	SUT472	0.3920299	0.2264684	0.3831724	4.49	5.88	41.25	79.50
NUT	NUT0826	0.392984	0.1885012	0.3375681	5.45	7.14	49.00	92.50
ORF-T	YHL044W	0.393534	0.2028757	0.3542941	4.72	6.19	43.00	80.50
SUT	SUT147	0.3954682	0.2191922	0.3751548	6.12	8.05	56.75	106.00
ORF-T	YBR117C	0.3964798	0.2598523	0.4220799	5.46	7.24	52.75	95.50
ORF-T	YGL033W	0.3965028	0.4478096	0.5995369	1.09	1.44	10.25	19.25
SUT	SUT602	0.3965665	0.2138682	0.3678401	3.78	4.96	34.75	65.75
SUT	SUT254	0.3968977	0.2221162	0.3784118	3.53	4.62	32.00	61.75
ORF-T	YBR115C	0.3971711	0.1880562	0.3370637	164.61	216.78	1577.25	3020.25
SUT	SUT350	0.3973819	0.233466	0.3918561	48.70	64.09	425.00	828.00
ORF-T	YPL257W	0.3977316	0.2703361	0.4333285	2.46	3.18	21.00	41.00
ORF-T	YHR137W	0.3979683	0.4729947	0.6208801	34.91	45.94	313.25	706.50

Class	Transcript name	RRP6 KO_vs_WT log2_fold _change	RRP6 KO_vs_WT p-value	RRP6 KO_vs_WT FDR	Ave Norm Reads in WT	Ave Norm Reads in RRP6	Average RAW read counts in WT	Average RAW read counts in RRP6
NUT	NUT1086	0.3987656	0.1469684	0.2842891	52.07	68.73	488.75	876.50
ORF-T	YJL222W	0.3993352	0.4939123	0.6373387	0.48	0.64	4.50	8.50
SUT	SUT548	0.3995755	0.3558771	0.5199127	1.30	1.73	12.25	23.00
SRT	SRT424	0.3999011	0.3288188	0.4932038	2.46	3.32	24.25	43.00
SRT	SRT136	0.399942	0.4554929	0.6059337	0.79	1.06	7.00	12.75
NUT	NUT0663	0.4002679	0.2300316	0.3878345	5.45	7.21	49.50	92.00
SUT	SUT444	0.4023982	0.4538821	0.6046964	1.26	1.68	12.25	23.25
NUT	NUT0712	0.4025144	0.3369104	0.5014456	1.93	2.60	19.00	34.50
ORF-T	YPR045C	0.4034101	0.0997347	0.2150686	60.92	80.69	581.25	1039.75
NUT	NUT0433	0.403553	0.5302769	0.6672282	0.73	0.97	7.00	13.50
NUT	NUT0769	0.4043027	0.5634254	0.6929054	0.72	0.93	6.00	12.00
AST	AS_YPL028W	0.4052618	0.4348795	0.5891083	1.21	1.54	9.75	20.50
SUT	SUT287	0.4062079	0.4382056	0.5917067	0.80	1.03	7.00	13.75
NUT	NUT1121	0.4065857	0.2037232	0.3551157	3.39	4.49	30.75	58.75
ORF-T	YER060W	0.4078573	0.1288959	0.259128	25.23	33.56	241.50	430.50
NUT	NUT0359	0.4079339	0.2317153	0.3897009	279.99	371.44	2400.25	4694.25
SUT	SUT527	0.4083782	0.1929465	0.3424876	4.80	6.37	44.25	83.75
SUT	SUT216	0.4095699	0.2853894	0.4488252	7.01	9.34	66.25	122.50
ORF-T	YEL034C-A	0.4096495	0.32871	0.4931139	3.17	4.20	28.00	54.00
NUT	NUT0340	0.4111389	0.1928924	0.3424876	4.21	5.65	40.75	75.75
SUT	SUT573	0.4111714	0.2899647	0.4538277	1.90	2.49	16.50	32.25
AST	AS_YIL137C	0.4113396	0.5019445	0.6442955	0.87	1.16	8.75	17.25
SUT	SUT088	0.4114363	0.3888932	0.5496285	0.98	1.33	8.75	16.00
ORF-T	YER053C	0.4126086	0.3815349	0.5428146	25.03	33.28	209.25	413.00
ORF-T	YHR196W	0.4126267	0.2161943	0.3709551	100.43	133.73	988.75	1843.25
SUT	SUT074	0.4127742	0.3078147	0.4736045	3.58	4.84	36.25	65.25
SUT	SUT358	0.413864	0.2566708	0.4181881	8.64	11.51	77.75	148.25
NUT	NUT1031	0.4143557	0.3546889	0.5192629	1.11	1.43	10.00	20.50
NUT	NUT0153	0.4155122	0.1466364	0.2839795	6.22	8.31	56.50	106.75

Class	Transcript name	RRP6 KO_vs_WT log2_fold _change	RRP6 KO_vs_WT p-value	RRP6 KO_vs_WT FDR	Ave Norm Reads in WT	Ave Norm Reads in RRP6	Average RAW read counts in WT	Average RAW read counts in RRP6
ORF-T	YDL154W	0.4159285	0.1791678	0.3262407	8.86	11.83	81.50	155.00
ORF-T	YPL265W	0.4162301	0.191731	0.3411708	54.81	73.07	472.75	947.25
SUT	SUT726	0.4167792	0.3767154	0.5384266	1.32	1.71	11.25	23.25
SUT	SUT762	0.4179645	0.3762503	0.5380541	1.62	2.14	14.25	28.75
ORF-T	YMR013C	0.4181083	0.175615	0.3218065	34.61	46.31	321.50	589.50
SUT	SUT334	0.4185638	0.1588865	0.3001507	11.39	15.25	103.25	192.50
NUT	NUT0657	0.4186653	0.1542732	0.293865	11.71	15.69	106.75	198.00
AST	AS_YGR170W	0.4191823	0.3392147	0.5038877	1.55	2.08	14.75	28.75
SRT	SRT256	0.4196769	0.473847	0.6212702	0.52	0.72	5.00	9.25
ORF-T	YER062C	0.4197531	0.1287508	0.2589084	28.89	38.63	256.00	486.25
AST	AS_YOL047C	0.4198377	0.2860329	0.4492766	5.61	7.49	48.50	90.50
NUT	NUT0091	0.4204489	0.1871136	0.3362177	5.76	7.75	55.25	104.75
AST	AS_YNL216W	0.4210247	0.3541213	0.5186939	1.21	1.62	11.25	21.75
AST	AS_YDR048C	0.4219003	0.3039862	0.4697206	1.37	1.79	12.25	24.50
CUT	CUT635	0.423006	0.3815959	0.5428146	1.16	1.58	10.50	19.50
ORF-T	YKL053W	0.4233274	0.4716585	0.620024	0.82	1.08	6.75	13.25
NUT	NUT0051	0.4235219	0.4226974	0.5780418	1.04	1.36	9.50	20.00
AST	AS_YBR179C	0.4235343	0.4226184	0.5780122	1.04	1.36	9.50	20.00
SUT	SUT763	0.4239244	0.413749	0.5703684	0.71	0.95	6.75	13.25
NUT	NUT0300	0.4242803	0.1195588	0.2444689	11.13	14.92	101.25	194.75
ORF-T	YJL119C	0.4247015	0.2352971	0.3940767	4.41	5.97	41.25	76.25
AST	AS_YPL244C	0.4250751	0.33329	0.4973211	1.53	2.07	14.75	28.00
SUT	SUT357	0.4250891	0.278257	0.4420201	5.16	6.91	44.50	84.00
ORF-T	YNR071C	0.4257818	0.2707319	0.4338249	2.69	3.60	23.50	45.50
SUT	SUT827	0.4265134	0.4376191	0.591073	0.95	1.30	9.00	16.75
NUT	NUT1200	0.4277467	0.077937	0.1786174	45.35	61.07	425.25	792.75
SUT	SUT543	0.4289236	0.3152649	0.480667	1.36	1.83	13.00	25.75
SUT	SUT260	0.4296911	0.2477711	0.4085894	5.11	6.90	47.75	91.50
ORF-T	YLR286W-A	0.4298071	0.3377249	0.5021553	2.08	2.81	19.50	37.75

TABLE S1: Differential expression data for RRP6 RNA-Seq dataset Page 233

Class	Transcript name	RRP6 KO_vs_WT log2_fold _change	RRP6 KO_vs_WT p-value	RRP6 KO_vs_WT FDR	Ave Norm Reads in WT	Ave Norm Reads in RRP6	Average RAW read counts in WT	Average RAW read counts in RRP6
SUT	SUT015	0.4300645	0.3306299	0.4950212	1.54	2.07	14.25	28.25
ORF-T	YAL037W	0.4304021	0.2315509	0.3896145	4.61	6.23	41.75	78.00
AST	AS_YOL048C	0.4307974	0.2764892	0.4401212	1.60	2.14	14.75	29.25
ORF-T	YGR288W	0.4315877	0.1330384	0.2653176	32.94	44.49	302.75	557.50
CUT	CUT826	0.4316007	0.2259831	0.3826081	3.59	4.80	30.75	61.50
ORF-T	YAL061W	0.4317915	0.3861156	0.5471599	38.03	51.31	329.25	596.00
CUT	CUT292	0.432273	0.3370486	0.5014456	2.20	3.00	20.75	39.50
ORF-T	YBR076C-A	0.4328469	0.1694153	0.3135847	7.26	9.74	63.50	127.00
ORF-T	YOR092W	0.4335485	0.157517	0.2984035	10.84	14.59	92.75	180.75
SUT	SUT650	0.433668	0.0760309	0.175488	44.50	60.17	417.50	781.50
NUT	NUT1376	0.4340354	0.1914922	0.3408662	5.47	7.41	51.00	97.75
NUT	NUT0056	0.4340944	0.1657935	0.3094553	5.23	7.12	50.75	95.50
ORF-T	YBR190W	0.4343738	0.322472	0.4867975	1.47	1.95	12.75	25.25
ORF-T	YPL216W	0.4353757	0.1105026	0.2311872	31.63	42.79	291.75	559.50
NUT	NUT1150	0.4371077	0.38657	0.5475729	0.79	1.05	7.25	14.75
CUT	CUT634	0.4375888	0.3837537	0.5451152	0.71	0.97	6.75	13.00
NUT	NUT0765	0.4380443	0.1087927	0.2289848	31.67	42.92	292.00	561.25
SUT	SUT738	0.4388712	0.2956461	0.4602895	1.23	1.69	11.25	21.25
SUT	SUT301	0.4393068	0.2942959	0.4589669	2.44	3.35	23.75	44.75
NUT	NUT0356	0.4393879	0.0854583	0.19129	69.40	94.13	633.50	1199.25
ORF-T	YCR054C	0.4395804	0.1320641	0.2637621	14.10	19.09	130.75	260.75
ORF-T	YMR230W-A	0.4396887	0.2842166	0.4479542	2.32	3.16	21.00	40.00
ORF-T	YHR216W	0.4399619	0.0852055	0.1909785	69.38	94.13	633.25	1199.25
NUT	NUT0455	0.4400443	0.3333898	0.4973229	1.73	2.35	15.50	29.00
NUT	NUT0717	0.4412021	0.0781795	0.1789699	27.43	37.28	254.25	487.00
SUT	SUT445	0.4412528	0.1591786	0.3005912	6.64	8.95	59.00	120.50
ORF-T	YDR241W	0.4417469	0.1638417	0.3068339	8.31	11.31	76.25	143.75
AST	AS_YOR262W	0.4418727	0.3620216	0.5259382	0.88	1.17	8.00	16.00
sn/snoRNA ET	SNR87-ET	0.4420467	0.1651297	0.3084331	153.18	208.18	1430.75	2548.50

Class	Transcript name	RRP6 KO_vs_WT log2_fold _change	RRP6 KO_vs_WT p-value	RRP6 KO_vs_WT FDR	Ave Norm Reads in WT	Ave Norm Reads in RRP6	Average RAW read counts in WT	Average RAW read counts in RRP6
ORF-T	YKL051W	0.4425309	0.1816732	0.329673	12.59	17.04	107.00	217.00
SRT	SRT189	0.4428009	0.231881	0.3897175	5.11	6.97	47.75	92.25
ORF-T	YKL005C	0.4447853	0.0667959	0.1593117	23.06	31.40	209.75	402.50
AST	AS_YPR009W	0.4462242	0.2908641	0.4548811	5.47	7.47	51.25	98.75
ORF-T	YMR169C	0.4464099	0.2156954	0.3702251	14.29	19.44	124.50	243.00
ORF-T	YJR108W	0.4465376	0.1954195	0.3454934	1.83	2.50	17.00	33.00
NUT	NUT0362	0.4471428	0.106375	0.225686	23.40	31.95	220.50	418.75
SUT	SUT303	0.4472486	0.3080453	0.473772	5.25	7.17	48.25	92.50
ORF-T	YKR012C	0.44788	0.3998328	0.5600189	0.86	1.17	8.00	15.75
ORF-T	YPL185W	0.4481532	0.5390454	0.673026	0.64	0.84	5.25	11.00
AST	AS_YJR146W	0.4486245	0.0852531	0.1910428	13.72	18.77	127.50	244.00
ORF-T	YDR345C	0.4486576	0.3610486	0.5250669	209.47	285.87	1982.50	3904.50
NUT	NUT0204	0.4496026	0.2249582	0.3815775	3.00	4.07	27.25	55.25
CUT	CUT380	0.4509789	0.3945771	0.5554817	0.71	0.93	5.75	11.75
ORF-T	YER130C	0.4510545	0.0961171	0.2090138	33.55	45.90	312.25	593.25
NUT	NUT0278	0.4518879	0.2653078	0.4283414	1.97	2.71	18.75	36.00
NUT	NUT0223	0.4525753	0.450451	0.601859	3.36	4.62	35.25	68.50
SUT	SUT140	0.4526438	0.1037593	0.2215817	8.90	12.16	80.75	158.25
ORF-T	YOL123W	0.4537928	0.1429491	0.279252	147.63	202.23	1329.75	2512.25
SUT	SUT286	0.4540133	0.3320845	0.4963303	0.79	1.13	7.75	14.75
NUT	NUT0675	0.4541616	0.1654745	0.3090082	53.18	72.80	466.00	939.25
AST	AS_YKL071W	0.4553851	0.1153502	0.2385545	10.24	14.03	94.00	189.50
NUT	NUT0560	0.4582523	0.3702024	0.5328463	0.67	0.96	6.50	12.25
NUT	NUT1348	0.4585946	0.4216083	0.5768654	0.75	0.98	6.25	13.50
ORF-T	YER078W-A	0.4586382	0.4123762	0.569644	1.10	1.48	9.25	19.00
ORF-T	YEL059W	0.4591389	0.4555318	0.6059337	0.99	1.36	9.25	19.00
SUT	SUT531	0.4591808	0.1726905	0.3178917	2.73	3.69	24.00	49.00
NUT	NUT0502	0.4592116	0.2889964	0.4528734	1.43	1.95	12.75	25.50
NUT	NUT0612	0.4597947	0.2660649	0.428868	1.61	2.18	14.00	28.75

Class	Transcript name	RRP6 KO_vs_WT log2_fold _change	RRP6 KO_vs_WT p-value	RRP6 KO_vs_WT FDR	Ave Norm Reads in WT	Ave Norm Reads in RRP6	Average RAW read counts in WT	Average RAW read counts in RRP6
SUT	SUT716	0.4600955	0.2808395	0.4446099	1.46	1.97	12.50	25.75
SUT	SUT448	0.4612301	0.2180831	0.3736874	2.49	3.38	22.25	46.25
AST	AS_YMR020W	0.4618624	0.2987709	0.4635086	0.83	1.13	7.50	15.00
SRT	SRT285	0.4619076	0.3492687	0.5138801	2.73	3.79	25.25	46.00
ORF-T	YDR273W	0.4634273	0.221836	0.3780622	4.41	6.14	42.25	78.50
ORF-T	YOL128C	0.4642632	0.1319774	0.2636411	19.59	27.12	187.50	350.00
AST	AS_YER005W	0.4655149	0.3103878	0.4757228	1.13	1.51	9.75	20.50
ORF-T	YER045C	0.4657461	0.1440123	0.2805139	7.56	10.35	66.75	140.50
NUT	NUT0851	0.4667529	0.0664866	0.158837	19.36	26.71	175.75	353.75
SUT	SUT295	0.4676434	0.1123279	0.2336972	10.61	14.60	94.00	196.50
SUT	SUT461	0.4679715	0.150113	0.2881667	7.19	9.98	68.50	136.00
SUT	SUT637	0.4690597	0.3644294	0.527928	0.70	0.93	6.25	13.25
NUT	NUT0567	0.4690778	0.1114909	0.2326246	10.61	14.62	94.00	196.75
ORF-T	YDL179W	0.4692325	0.2009729	0.3520255	3.82	5.26	35.25	71.75
AST	AS_YLR095C	0.4693221	0.2072311	0.3595063	5.27	7.31	49.00	96.50
NUT	NUT0373	0.4704772	0.4589116	0.6088332	0.49	0.71	4.75	9.00
SUT	SUT789	0.4723221	0.328136	0.492766	0.99	1.35	9.00	18.50
NUT	NUT0272	0.4727536	0.3015273	0.4668511	1.54	2.18	14.50	27.25
SRT	SRT133	0.4732623	0.3469757	0.5119097	1.27	1.73	10.75	22.25
ORF-T	YHR101C	0.4745207	0.0726204	0.1692406	15.07	20.92	134.75	266.50
SUT	SUT247	0.4791554	0.181595	0.3295904	3.93	5.47	36.00	72.75
NUT	NUT0918	0.4807212	0.2864956	0.4497232	1.51	2.05	12.75	27.00
ORF-T	YGL209W	0.4810318	0.3130413	0.4782892	5.35	7.47	51.75	102.75
CUT	CUT525	0.4821191	0.3994361	0.5596656	0.79	1.08	7.00	14.75
AST	AS_YJR132W	0.4822022	0.379211	0.540566	0.70	1.00	7.00	14.00
ORF-T	YGR039W	0.4830066	0.1354747	0.2690285	4.21	5.92	39.75	78.50
NUT	NUT0162	0.4832719	0.396324	0.5570793	1.32	1.78	10.25	22.50
AST	AS_YDR331W	0.4835679	0.4267588	0.5819135	0.62	0.88	5.75	11.25
NUT	NUT0207	0.48462	0.0640345	0.1543317	28.77	40.34	268.00	510.50

Class	Transcript name	RRP6 KO_vs_WT log2_fold _change	RRP6 KO_vs_WT p-value	RRP6 KO_vs_WT FDR	Ave Norm Reads in WT	Ave Norm Reads in RRP6	Average RAW read counts in WT	Average RAW read counts in RRP6
CUT	CUT916	0.4888369	0.3304407	0.4949003	0.65	0.90	5.75	11.75
ORF-T	YOR318C	0.4889371	0.1390938	0.2736822	6.95	9.77	62.75	122.75
SUT	SUT740	0.4896009	0.3625477	0.5263362	0.85	1.24	8.75	16.50
SUT	SUT670	0.4899731	0.0484126	0.1249762	36.26	50.90	329.00	676.25
SUT	SUT482	0.490357	0.3123407	0.477534	1.10	1.51	9.25	19.25
ORF-T	YNR072W	0.4904428	0.3332376	0.4973211	1.02	1.42	9.50	19.50
SUT	SUT750	0.4904695	0.1001545	0.2157107	5.41	7.60	49.75	100.00
ORF-T	YMR013C-A	0.4907491	0.1629395	0.3056328	5.40	7.60	49.00	96.50
ORF-T	YLR101C	0.4914114	0.2637949	0.4267848	0.98	1.42	9.50	18.50
SUT	SUT807	0.4920736	0.1452844	0.282174	5.04	7.13	48.75	96.00
SUT	SUT289	0.492182	0.1981457	0.3488306	2.65	3.71	24.50	50.50
ORF-T	YEL035C	0.493353	0.2529715	0.4143195	1.54	2.17	13.75	27.25
SUT	SUT816	0.4943127	0.3411154	0.5057526	0.91	1.28	8.25	17.00
SUT	SUT479	0.4944111	0.1695748	0.3137025	5.75	8.17	55.25	104.25
ORF-T	YFR022W	0.4944937	0.0897835	0.1981574	8.56	12.01	76.25	159.75
ORF-T	YOL101C	0.4946077	0.2562882	0.4179865	74.66	105.24	694.00	1324.75
ORF-T	YGL098W	0.4947751	0.0286552	0.0826068	47.70	67.27	442.50	872.50
AST	AS_YDR036C	0.4950799	0.3359722	0.5003608	0.90	1.26	8.50	17.50
ORF-T	YGL194C-A	0.4959703	0.071961	0.1682564	15.71	22.17	142.25	283.25
AST	AS_YML036W	0.4963183	0.2252929	0.3819525	2.44	3.52	24.00	46.00
NUT	NUT0496	0.4964548	0.234277	0.3928903	2.39	3.33	21.50	45.75
NUT	NUT0943	0.4968645	0.3965146	0.5571981	0.89	1.31	9.75	18.25
ORF-T	YNL195C	0.4971097	0.279373	0.4432344	1.56	2.20	13.50	26.50
ORF-T	YKL047W	0.4973036	0.0291949	0.0839946	33.75	47.68	311.75	621.75
SRT	SRT553	0.4979065	0.3373318	0.5017189	0.70	1.00	6.25	12.50
NUT	NUT0582	0.4986927	0.2308975	0.3888716	5.71	8.09	52.75	104.75
SRT	SRT514	0.4987313	0.3872469	0.5481468	1.28	1.84	13.00	25.25
ORF-T	YJL052W	0.498894	0.2642677	0.4270634	163.06	230.36	1317.25	2777.25
ORF-T	YBR291C	0.4989433	0.2935232	0.4580451	36.82	52.09	359.50	677.75

Class	Transcript name	RRP6 KO_vs_WT log2_fold _change	RRP6 KO_vs_WT p-value	RRP6 KO_vs_WT FDR	Ave Norm Reads in WT	Ave Norm Reads in RRP6	Average RAW read counts in WT	Average RAW read counts in RRP6
SUT	SUT687	0.4989497	0.1771364	0.3236536	3.13	4.39	28.00	57.75
ORF-T	YHR022C	0.499231	0.1192521	0.243972	3.75	5.29	34.50	70.50
SRT	SRT244	0.5001592	0.2990855	0.4637113	0.78	1.13	7.50	15.00
ORF-T	YGL247W	0.5006201	0.0618657	0.1503987	11.64	16.53	109.00	215.75
AST	AS_YOL126C	0.5015587	0.3566811	0.5205923	0.89	1.25	8.50	18.00
NUT	NUT0787	0.5021244	0.077677	0.178075	10.83	15.41	103.50	202.00
NUT	NUT0229	0.5024073	0.3497337	0.5141733	0.61	0.90	6.00	11.75
ORF-T	YOR180C	0.5042457	0.1578204	0.2988097	2.51	3.57	23.25	46.50
SUT	SUT469	0.5049936	0.1377658	0.2719183	1.97	2.81	18.50	37.50
AST	AS_YLR122C	0.505026	0.3199218	0.4846877	0.65	0.94	6.50	13.25
NUT	NUT0600	0.5054928	0.3168968	0.4823747	1.31	1.89	12.75	25.50
SUT	SUT456	0.5055293	0.1564097	0.2968635	5.55	7.84	49.50	105.75
AST	AS_YIR018C-A	0.506414	0.1147974	0.2377526	3.56	5.06	33.25	68.00
ORF-T	YBR284W	0.5067778	0.1106094	0.2313596	8.74	12.45	81.75	164.00
ORF-T	YJL077C	0.5081391	0.4164683	0.5730986	0.67	0.93	5.75	12.25
CUT	CUT640	0.509203	0.4412556	0.5942336	0.74	1.00	6.00	14.00
ORF-T	YPL018W	0.5099751	0.0781642	0.1789699	18.62	26.55	170.75	339.50
NUT	NUT1179	0.5105227	0.0934752	0.2045582	5.20	7.39	46.25	94.50
NUT	NUT0683	0.5106885	0.0562732	0.1404032	26.03	37.01	235.75	507.75
SUT	SUT449	0.511505	0.321797	0.4861425	0.70	0.97	6.25	13.25
ORF-T	YHR071C-A	0.5117284	0.2583849	0.4202688	0.76	1.09	7.00	14.25
SUT	SUT321	0.5129418	0.1498127	0.2878642	1.83	2.62	16.50	33.25
ORF-T	YHR049C-A	0.5132104	0.0711701	0.1670706	14.42	20.62	132.00	261.75
CUT	CUT377	0.5139686	0.2509513	0.4123025	1.64	2.39	16.00	31.50
AST	AS_YPL196W	0.5140877	0.3480528	0.5129961	0.53	0.79	5.25	10.50
SUT	SUT533	0.5161614	0.3209502	0.4855906	0.67	0.94	5.75	12.00
ORF-T	YLR311C	0.5165411	0.3757578	0.5376957	1.03	1.51	9.50	18.00
ORF-T	YNL146C-A	0.5172712	0.1262517	0.2548896	4.47	6.36	39.00	80.25
ORF-T	YER066W	0.5182117	0.0488208	0.1256211	14.01	20.06	127.00	258.25

Class	Transcript name	RRP6 KO_vs_WT log2_fold _change	RRP6 KO_vs_WT p-value	RRP6 KO_vs_WT FDR	Ave Norm Reads in WT	Ave Norm Reads in RRP6	Average RAW read counts in WT	Average RAW read counts in RRP6
ORF-T	YLR053C	0.5182984	0.3201715	0.4849068	1.06	1.53	9.25	18.25
SUT	SUT570	0.5202529	0.1351284	0.2684995	3.75	5.44	35.75	71.50
NUT	NUT0394	0.5215744	0.0238007	0.0706317	31.79	45.71	299.00	594.50
SRT	SRT229	0.5216163	0.1419283	0.2783289	1.83	2.63	16.50	33.50
NUT	NUT0324	0.5233737	0.4284318	0.5829443	0.63	0.94	6.00	11.00
SUT	SUT151	0.5233849	0.4284652	0.5829443	0.63	0.94	6.00	11.00
SUT	SUT175	0.5235101	0.1983796	0.3491205	1.97	2.79	17.25	37.00
NUT	NUT0545	0.5235147	0.191279	0.3405469	2.60	3.68	21.75	45.75
SUT	SUT029	0.5237314	0.1805069	0.3282653	1.18	1.71	11.00	22.50
AST	AS_YPL268W	0.5237571	0.4531846	0.6040066	0.57	0.85	5.50	10.75
NUT	NUT0354	0.5238806	0.1520786	0.2907775	3.22	4.59	28.50	60.50
SUT	SUT702	0.5273349	0.1573047	0.2980572	1.89	2.74	17.75	36.25
ORF-T	YMR087W	0.5281131	0.0983788	0.2127496	8.49	12.25	77.25	157.25
SRT	SRT212	0.5297231	0.1423898	0.2784878	3.56	5.13	33.25	70.00
SUT	SUT711	0.5297452	0.0740914	0.1721258	22.78	32.95	219.00	444.75
ORF-T	YLL042C	0.5299732	0.1604185	0.3025927	3.43	5.03	33.00	63.50
AST	AS_YBR253W	0.5300605	0.2031085	0.3545346	1.43	2.01	12.50	27.00
CUT	CUT052	0.5309523	0.215606	0.3701717	1.87	2.77	18.50	35.50
NUT	NUT0485	0.5321778	0.1179895	0.2418796	4.14	5.98	38.00	79.25
SRT	SRT362	0.5322198	0.1599951	0.3018504	2.47	3.55	22.00	46.25
SUT	SUT429	0.5327086	0.2366355	0.3957924	0.84	1.23	7.75	16.00
SUT	SUT757	0.5329519	0.0612633	0.1492221	6.62	9.52	59.00	125.75
ORF-T	YGR256W	0.5332604	0.1035576	0.2212915	8.87	12.88	80.50	160.25
SUT	SUT054	0.533787	0.2155084	0.370155	0.80	1.19	7.50	15.25
NUT	NUT0587	0.5343907	0.241622	0.4012708	1.19	1.64	9.75	22.00
NUT	NUT0108	0.5345629	0.3179095	0.4830921	0.58	0.84	5.25	11.00
SUT	SUT090	0.5354385	0.1005974	0.2164309	8.64	12.56	80.75	164.50
ORF-T	YDR249C	0.5362498	0.0528341	0.1334282	19.36	28.15	183.50	370.50
SUT	SUT636	0.5373045	0.269779	0.4327795	0.66	0.99	6.00	12.00

TABLE S1: Differential expression data for RRP6 RNA-Seq dataset Page 239

Class	Transcript name	RRP6 KO_vs_WT log2_fold _change	RRP6 KO_vs_WT p-value	RRP6 KO_vs_WT FDR	Ave Norm Reads in WT	Ave Norm Reads in RRP6	Average RAW read counts in WT	Average RAW read counts in RRP6
NUT	NUT0446	0.538496	0.0845005	0.1897356	7.88	11.40	72.25	157.25
NUT	NUT0948	0.5392826	0.1296471	0.2601714	2.75	3.99	25.75	54.50
CUT	CUT886	0.5395467	0.2001022	0.3510491	1.60	2.33	14.50	30.00
SUT	SUT078	0.5396207	0.1633546	0.3061653	4.88	7.14	46.25	93.50
ORF-T	YHR180W-A	0.540267	0.208291	0.3606467	1.46	2.10	12.50	26.25
SUT	SUT230	0.54059	0.1628494	0.3055969	3.70	5.32	33.25	74.75
SUT	SUT046	0.5409719	0.3304053	0.4949003	0.99	1.44	9.00	19.25
CUT	CUT423	0.5413826	0.2376885	0.39729	1.32	1.95	12.00	24.50
SUT	SUT498	0.5424629	0.1310891	0.2622303	2.69	3.92	25.25	53.25
SUT	SUT536	0.5436306	0.0898494	0.1981902	7.57	11.02	68.00	143.00
SUT	SUT155	0.5437926	0.2604086	0.4226634	0.86	1.19	7.25	16.25
NUT	NUT1284	0.5460388	0.2031045	0.3545346	1.49	2.13	12.75	28.00
NUT	NUT1503	0.5470892	0.3408002	0.5053867	1.12	1.59	9.50	21.75
NUT	NUT0724	0.5482916	0.2203602	0.3763102	14.35	21.04	131.50	255.00
AST	AS_YOR260W	0.5485851	0.2428017	0.4024347	1.33	1.95	12.25	25.50
SUT	SUT495	0.5505458	0.1218905	0.2484123	3.33	4.90	31.00	63.50
SUT	SUT460	0.5519177	0.2214386	0.377743	0.97	1.48	9.75	19.25
AST	AS_YGR208W	0.5521941	0.1879666	0.3370298	1.34	1.96	11.75	24.25
SUT	SUT539	0.5543913	0.123067	0.2501023	4.44	6.46	39.25	88.50
ORF-T	YDR379C-A	0.5553207	0.1688453	0.3128696	3.73	5.43	33.25	75.50
NUT	NUT1437	0.5553687	0.1108005	0.2316153	13.25	19.52	126.75	262.75
ORF-T	YJL016W	0.5559686	0.0416014	0.111187	34.99	51.45	312.75	637.00
ORF-T	YIR030C	0.5564077	0.1365821	0.2707276	2.45	3.59	22.50	48.25
AST	AS_YBR195C	0.5566346	0.2978828	0.4625924	1.04	1.53	9.75	21.25
ORF-T	YMR316C-A	0.5575776	0.2116588	0.3650363	0.95	1.40	8.75	18.75
ORF-T	YNL024C	0.5586719	0.0719906	0.1682564	27.90	41.23	271.75	526.50
NUT	NUT1095	0.5589596	0.0759205	0.1753109	4.72	6.94	43.00	92.00
NUT	NUT1405	0.559067	0.3282911	0.4927843	0.64	0.88	5.00	11.50
ORF-T	YPL264C	0.5598222	0.0827331	0.1869747	4.54	6.66	41.25	87.75

TABLE S1: Differential expression data for RRP6 RNA-Seq dataset Page 240

Class	Transcript name	RRP6 KO_vs_WT log2_fold _change	RRP6 KO_vs_WT p-value	RRP6 KO_vs_WT FDR	Ave Norm Reads in WT	Ave Norm Reads in RRP6	Average RAW read counts in WT	Average RAW read counts in RRP6
SUT	SUT116	0.5599917	0.2773013	0.4412666	1.67	2.49	17.00	36.25
SUT	SUT166	0.5608021	0.0767121	0.1766533	5.30	7.80	49.25	107.25
SRT	SRT78	0.5609585	0.3007983	0.465937	0.74	1.10	6.75	14.50
SUT	SUT181	0.5612499	0.1158459	0.2390403	2.29	3.33	20.75	45.50
ORF-T	YBR297W	0.5616283	0.045606	0.1194542	68.16	100.66	646.25	1350.00
AST	AS_YNL321W	0.5626877	0.2053634	0.3572338	2.04	3.06	20.25	41.50
SRT	SRT81	0.5633308	0.4016828	0.5608572	0.78	1.19	7.50	14.50
ORF-T	YGL165C	0.5670184	0.1485662	0.2862873	1.43	2.14	13.00	27.00
ORF-T	YER148W-A	0.5678817	0.2268816	0.3835829	0.77	1.11	6.75	14.75
ORF-T	YKL132C	0.5684889	0.0221099	0.0666139	19.31	28.71	180.25	373.25
sn/snoRNA ET	SNR86-ET	0.5685577	0.1438784	0.2804462	20.10	29.80	175.50	372.00
SUT	SUT143	0.5698715	0.3659999	0.5286486	1.73	2.58	16.50	33.50
ORF-T	YOR134W	0.5699295	0.1951693	0.3451573	14.52	21.61	133.00	261.50
SRT	SRT570	0.570101	0.2696261	0.4327168	0.61	0.84	5.00	11.25
CUT	CUT451	0.5701056	0.1579975	0.2989206	2.63	3.93	24.75	51.75
NUT	NUT0072	0.5701961	0.1469292	0.2842891	1.22	1.83	11.50	24.25
NUT	NUT0929	0.5707416	0.0762919	0.1759267	3.75	5.52	33.25	72.25
CUT	CUT917	0.5710652	0.2255154	0.3822655	1.55	2.29	13.50	29.25
NUT	NUT1208	0.5720935	0.1993372	0.3501336	2.52	3.82	25.00	49.75
NUT	NUT0337	0.5721902	0.1059748	0.2249789	6.50	9.70	63.25	135.25
NUT	NUT1063	0.5735104	0.1455242	0.2824223	2.62	3.90	23.25	49.25
CUT	CUT093	0.5736474	0.1022175	0.2191711	7.53	11.23	70.75	151.75
NUT	NUT1088	0.5740332	0.076032	0.175488	4.11	6.05	36.25	80.75
ORF-T	YGR176W	0.5744741	0.3129666	0.4782475	0.63	0.88	5.25	12.50
ORF-T	YOL114C	0.5747948	0.1048305	0.2231341	2.49	3.67	22.00	48.25
ORF-T	YIL094C	0.5757682	0.0565616	0.1409479	131.31	195.75	1247.00	2651.00
SUT	SUT075	0.5773956	0.0427158	0.1133842	9.63	14.33	85.75	186.25
SUT	SUT262	0.5776209	0.2238257	0.3803604	1.07	1.62	10.00	20.50
ORF-T	YCL064C	0.5786987	0.1763206	0.3226891	36.99	55.20	339.50	791.25

TABLE S1: Differential expression data for RRP6 RNA-Seq dataset Page 241

Class	Transcript name	RRP6 KO_vs_WT log2_fold _change	RRP6 KO_vs_WT p-value	RRP6 KO_vs_WT FDR	Ave Norm Reads in WT	Ave Norm Reads in RRP6	Average RAW read counts in WT	Average RAW read counts in RRP6
ORF-T	YMR232W	0.5792438	0.1114887	0.2326246	1.61	2.44	15.50	33.00
NUT	NUT1120	0.5794441	0.200726	0.3517763	0.75	1.13	6.75	14.25
ORF-T	YGR295C	0.5800219	0.0200282	0.0615558	67.39	100.76	611.50	1281.00
SUT	SUT191	0.5804006	0.2152302	0.3697413	1.03	1.53	9.50	20.50
AST	AS_YJR069C	0.5807273	0.2443695	0.4043686	0.97	1.48	9.00	18.50
SUT	SUT250	0.5839961	0.1781917	0.3251104	1.75	2.57	15.50	35.25
ORF-T	YPR121W	0.5853579	0.0309641	0.0879879	34.11	51.26	318.75	661.75
NUT	NUT0931	0.5862062	0.0961319	0.2090138	2.64	3.97	24.75	53.50
SUT	SUT189	0.5875778	0.0358965	0.0990946	17.63	26.49	160.25	341.50
sn/snoRNA ET	SNR64-ET	0.5889272	0.0286464	0.0826052	25.22	37.89	221.25	477.25
ORF-T	YPL081W	0.5890903	0.0698796	0.1648726	105.81	159.12	962.50	2204.75
CUT	CUT477	0.5907536	0.2863624	0.4496541	1.22	1.90	12.25	24.25
NUT	NUT0784	0.5909405	0.0690384	0.1633467	105.83	159.36	962.75	2208.25
ORF-T	YCR105W	0.5912965	0.2102117	0.3632239	0.64	0.98	6.00	12.75
NUT	NUT0917	0.5935126	0.0593787	0.1457581	6.73	10.16	61.50	129.50
AST	AS_YPL091W	0.5951089	0.4186757	0.5744898	0.61	0.88	5.00	12.25
SUT	SUT267	0.5954481	0.1111027	0.2321026	2.88	4.38	26.50	55.50
SUT	SUT298	0.5959971	0.2192	0.3751548	2.61	3.98	23.50	48.50
SUT	SUT084	0.5964058	0.1306113	0.2614909	2.74	4.10	23.75	52.75
ORF-T	YLR137W	0.5971089	0.0307965	0.0876771	18.22	27.67	177.25	368.50
ORF-T	YDR173C	0.5976982	0.0568067	0.1413736	6.73	10.19	61.50	130.00
SRT	SRT458	0.5981012	0.0630934	0.152684	4.37	6.68	41.75	87.00
CUT	CUT895	0.5986329	0.1565533	0.2969682	0.97	1.49	9.00	19.25
NUT	NUT0060	0.5989481	0.1518917	0.2904753	0.90	1.35	8.50	18.75
NUT	NUT0849	0.6003892	0.2075814	0.3599124	0.93	1.45	9.00	18.75
NUT	NUT0382	0.6015185	0.1123758	0.2336972	1.60	2.43	14.25	30.25
ORF-T	YNL222W	0.6016203	0.031891	0.0902578	8.27	12.51	75.00	165.25
ORF-T	YFL053W	0.6018856	0.1558464	0.2961845	2.73	4.19	25.25	51.25
SUT	SUT093	0.6030763	0.0261606	0.0765759	35.50	53.99	341.00	729.50

Class	Transcript name	RRP6 KO_vs_WT log2_fold _change	RRP6 KO_vs_WT p-value	RRP6 KO_vs_WT FDR	Ave Norm Reads in WT	Ave Norm Reads in RRP6	Average RAW read counts in WT	Average RAW read counts in RRP6
SUT	SUT551	0.6035324	0.0309048	0.087886	13.16	20.08	124.75	260.75
SUT	SUT474	0.6042252	0.1367016	0.2708248	1.25	1.91	11.25	24.00
CUT	CUT291	0.6046311	0.1483252	0.2860964	4.26	6.52	40.25	82.50
SRT	SRT415	0.6051815	0.0306695	0.0873895	13.16	20.10	124.75	261.00
NUT	NUT0884	0.6059358	0.0539407	0.1357808	7.75	11.84	73.75	160.25
AST	AS_YLR064W	0.605983	0.1883763	0.3374643	1.58	2.38	14.00	32.00
ORF-T	YDR185C	0.6060097	0.0338652	0.0945189	11.23	17.07	100.25	218.25
NUT	NUT1003	0.6061211	0.2046114	0.3562945	3.66	5.61	36.75	79.25
ORF-T	YKL125W	0.6068664	0.0370396	0.1015795	82.74	126.13	806.50	1673.00
AST	AS_YKR096W	0.6071205	0.3891034	0.5498485	0.53	0.83	5.00	10.50
ORF-T	YHR193C-A	0.6084802	0.2240877	0.3806773	0.88	1.34	8.00	17.50
NUT	NUT1524	0.6085723	0.2739145	0.4372695	0.70	1.04	6.25	14.50
NUT	NUT0357	0.6093795	0.0725545	0.1691651	4.60	6.98	42.00	95.50
SUT	SUT499	0.6101453	0.1181085	0.2420744	3.71	5.72	35.50	74.00
NUT	NUT0791	0.6106518	0.0261155	0.0764881	75.83	115.95	729.00	1458.50
sn/snoRNA ET	SNR72-ET	0.611098	0.1738983	0.3194154	1.16	1.75	10.25	23.00
ORF-T	YPL024W	0.6111084	0.0256284	0.0752802	74.61	114.12	717.25	1434.00
NUT	NUT1391	0.6113887	0.0725187	0.1691207	2.82	4.27	25.25	57.00
SUT	SUT553	0.6116317	0.1884207	0.3374839	0.80	1.22	7.25	15.75
SUT	SUT587	0.6116921	0.047007	0.1220753	7.74	11.89	72.25	152.25
SUT	SUT374	0.6136896	0.0773805	0.1775522	4.41	6.72	40.75	93.00
NUT	NUT0993	0.6149031	0.033168	0.0930105	6.02	9.23	55.00	120.00
NUT	NUT1074	0.6170849	0.1298115	0.2603976	3.93	6.03	36.00	76.50
ORF-T	YNL042W	0.6181515	0.0680728	0.1616609	3.43	5.23	30.00	65.50
SUT	SUT769	0.6183801	0.0780719	0.1788453	3.63	5.56	32.75	72.50
NUT	NUT0569	0.6193422	0.2776799	0.4415903	0.78	1.18	7.25	17.00
NUT	NUT1518	0.6195355	0.1516033	0.2900887	1.95	2.97	17.25	38.50
AST	AS_YMR318C	0.62042	0.0919755	0.2019283	1.48	2.27	13.25	28.75
ORF-T	YFL052W	0.6206111	0.0947776	0.206772	34.44	53.03	323.75	652.00

Class	Transcript name	RRP6 KO_vs_WT log2_fold _change	RRP6 KO_vs_WT p-value	RRP6 KO_vs_WT FDR	Ave Norm Reads in WT	Ave Norm Reads in RRP6	Average RAW read counts in WT	Average RAW read counts in RRP6
AST	AS_YHR205W	0.6206126	0.1896668	0.3388463	1.43	2.24	13.50	28.50
SUT	SUT640	0.6213566	0.103796	0.2216132	1.91	2.83	15.75	36.75
SRT	SRT119	0.6213976	0.2966292	0.4613926	0.79	1.24	7.75	16.75
SUT	SUT157	0.6232929	0.1068755	0.2264145	4.86	7.52	47.75	104.75
ORF-T	YLR431C	0.6239656	0.060497	0.1478914	14.66	22.65	137.75	291.25
SUT	SUT796	0.6246974	0.0673497	0.1603069	4.26	6.49	37.50	88.75
NUT	NUT0801	0.6258447	0.1217301	0.2481857	2.87	4.44	25.75	56.75
SUT	SUT177	0.6261634	0.0638795	0.1540687	4.53	6.96	41.50	95.00
NUT	NUT0553	0.6273663	0.1897065	0.3388463	0.75	1.16	7.00	15.50
CUT	CUT035	0.6274752	0.1120157	0.2335263	1.75	2.77	17.50	37.75
ORF-T	YCL058C	0.6280528	0.1717297	0.3165854	0.88	1.35	7.75	17.00
SUT	SUT395	0.6297076	0.1298834	0.2604901	2.13	3.30	20.00	46.00
ORF-T	YOR161C	0.6304266	0.0837227	0.1884928	76.62	118.61	666.25	1411.25
ORF-T	YOR366W	0.6363236	0.0990604	0.2139484	1.36	2.17	13.25	28.75
NUT	NUT1220	0.6368765	0.0855421	0.1913925	2.35	3.64	21.00	47.00
AST	AS_YHR163W	0.6374012	0.2035739	0.3550397	0.71	1.10	6.75	15.50
AST	AS_YKL174C	0.6375903	0.1470683	0.2842891	1.03	1.59	9.00	20.00
NUT	NUT0168	0.6390597	0.0237494	0.0705557	10.22	15.87	90.75	207.75
SUT	SUT795	0.6412273	0.0258299	0.0757616	13.06	20.35	117.75	265.00
NUT	NUT0244	0.6415771	0.0171251	0.054322	24.81	38.77	231.75	503.00
ORF-T	YKL090W	0.6421583	0.0082049	0.0292879	32.29	50.55	308.25	645.25
NUT	NUT0479	0.6424822	0.0425708	0.1131482	3.78	5.90	34.00	75.25
SUT	SUT307	0.6427993	0.1096696	0.2300276	4.84	7.53	44.00	101.50
ORF-T	YER060W-A	0.6434761	0.0897661	0.1981574	3.11	4.85	29.25	67.25
ORF-T	YJR010W	0.6437247	0.008122	0.0290332	37.54	58.64	338.50	752.75
SUT	SUT700	0.644935	0.2074054	0.359731	0.71	1.10	6.25	14.25
CUT	CUT529	0.6475686	0.1670725	0.3107838	1.00	1.56	9.50	21.75
SUT	SUT145	0.6476047	0.0516153	0.1310394	6.97	10.99	65.25	140.00
NUT	NUT0388	0.6485528	0.033184	0.0930295	8.93	14.07	83.75	180.25

Class	Transcript name	RRP6 KO_vs_WT log2_fold _change	RRP6 KO_vs_WT p-value	RRP6 KO_vs_WT FDR	Ave Norm Reads in WT	Ave Norm Reads in RRP6	Average RAW read counts in WT	Average RAW read counts in RRP6
SUT	SUT300	0.6487474	0.0630516	0.152656	5.12	8.01	47.50	109.25
ORF-T	YER097W	0.6488125	0.0694171	0.164032	3.01	4.68	26.75	61.25
ORF-T	YLR343W	0.6506552	0.1248065	0.2528177	7.91	12.47	74.50	159.50
SUT	SUT111	0.6508527	0.016159	0.0518444	24.49	38.52	229.00	499.75
SUT	SUT557	0.6511088	0.1150394	0.2380352	2.25	3.48	20.00	47.00
AST	AS_YHR022C	0.6525007	0.1745429	0.3203659	0.64	0.98	5.75	13.25
ORF-T	YMR122w-a_RT	0.6528147	0.0999503	0.215363	5.16	8.10	47.50	109.00
SUT	SUT712	0.6535064	0.1125731	0.2340592	2.61	4.11	25.00	58.00
AST	AS_YHR016C	0.6539286	0.217514	0.3728389	0.66	1.06	6.50	14.75
ORF-T	YLR312C	0.6557339	0.1007243	0.2164758	17.68	27.88	157.75	332.50
SUT	SUT812	0.6569676	0.146683	0.2840153	2.00	3.14	18.25	41.75
ORF-T	YMR105W-A	0.6575184	0.2827527	0.4464889	0.70	1.05	5.50	13.25
SUT	SUT271	0.6581794	0.1748367	0.320718	0.85	1.36	8.00	18.00
SUT	SUT656	0.6588307	0.3167561	0.4822843	0.94	1.49	8.75	19.25
NUT	NUT1196	0.6592779	0.0618238	0.1503694	5.05	7.93	44.50	102.00
SUT	SUT818	0.6594839	0.1154254	0.2386121	1.03	1.67	9.75	21.50
CUT	CUT852	0.6605945	0.1248294	0.2528177	1.14	1.75	10.00	23.75
NUT	NUT1093	0.6620541	0.0324271	0.0913802	6.11	9.63	54.75	126.50
SUT	SUT245	0.6630957	0.0360009	0.099325	3.75	5.93	33.75	75.50
NUT	NUT0572	0.6636448	0.1136521	0.2357679	1.38	2.18	12.75	29.50
SUT	SUT347	0.6637472	0.1354165	0.2689659	1.38	2.20	13.00	29.25
AST	AS_YOR387C	0.6638777	0.1068474	0.2264024	1.97	3.14	18.50	41.00
ORF-T	YPL003W	0.6640298	0.0160155	0.0514661	50.24	79.69	462.00	978.50
SUT	SUT035	0.6642785	0.3246514	0.4891922	0.62	0.97	5.25	12.50
ORF-T	YPR156C	0.6647468	0.0132355	0.0439494	34.88	55.18	307.50	734.25
SUT	SUT766	0.6655401	0.0304488	0.0868342	4.24	6.76	39.25	87.50
AST	AS_YJL194W	0.6656396	0.1978172	0.3483739	0.77	1.22	7.25	16.75
AST	AS_YHR119W	0.6659834	0.2246773	0.3814	0.82	1.29	7.50	18.00
NUT	NUT1164	0.666384	0.0826349	0.1868363	1.54	2.36	13.25	32.25

TABLE S1: Differential expression data for RRP6 RNA-Seq dataset Page 245

Class	Transcript name	RRP6 KO_vs_WT log2_fold _change	RRP6 KO_vs_WT p-value	RRP6 KO_vs_WT FDR	Ave Norm Reads in WT	Ave Norm Reads in RRP6	Average RAW read counts in WT	Average RAW read counts in RRP6
ORF-T	YOL150C	0.6671248	0.1340827	0.2669853	1.55	2.48	14.50	32.75
SUT	SUT550	0.6672183	0.0790904	0.1804816	2.78	4.47	26.00	56.50
NUT	NUT1481	0.6678807	0.1555755	0.2957812	0.71	1.10	6.25	14.50
NUT	NUT0121	0.668911	0.3089347	0.4747482	1.01	1.62	9.25	21.00
SRT	SRT334	0.6689676	0.2441878	0.4042006	0.46	0.67	3.75	9.00
NUT	NUT0372	0.6690287	0.13682	0.2709876	1.20	1.91	11.25	26.00
SUT	SUT590	0.6693721	0.0528973	0.1335211	4.07	6.44	36.75	87.00
CUT	CUT551	0.6695734	0.051265	0.1304457	7.92	12.73	78.75	163.25
SUT	SUT211	0.6699452	0.2608911	0.4232345	1.84	2.97	17.75	36.00
CUT	CUT589	0.6708604	0.2106439	0.3635969	0.46	0.80	4.75	10.25
CUT	CUT665	0.6710266	0.0926985	0.2032112	4.11	6.63	40.50	86.50
NUT	NUT0173	0.6711339	0.0711895	0.1670706	5.88	9.40	55.25	123.50
AST	AS_YML035C	0.6729257	0.1821335	0.3302111	1.02	1.61	9.50	23.50
ORF-T	YOL118C	0.6732363	0.2316473	0.3897009	0.76	1.29	7.75	15.75
ORF-T	YLR411W	0.6742969	0.11735	0.2410096	1.36	2.14	12.25	28.75
ORF-T	YGR146C-A	0.6744827	0.0489306	0.1258714	4.82	7.67	42.75	96.25
ORF-T	YDL182W	0.6745682	0.0574257	0.1422379	10.49	16.67	93.25	231.50
NUT	NUT0195	0.6747684	0.1163223	0.2396312	3.37	5.36	29.00	67.25
NUT	NUT1081	0.6756469	0.0393946	0.1068177	4.05	6.54	38.75	87.00
ORF-T	YGL122C	0.6761779	0.0108318	0.0372155	51.08	81.46	441.00	1071.50
SUT	SUT370	0.6773991	0.0328846	0.0924987	3.94	6.23	35.00	84.00
SUT	SUT661	0.6774493	0.0456606	0.1195147	2.84	4.48	25.25	60.25
SRT	SRT104	0.6778769	0.2224044	0.378583	0.82	1.30	7.00	15.75
SRT	SRT206	0.6779404	0.1671359	0.3108229	2.60	4.14	24.50	61.00
ORF-T	YNL203C	0.679213	0.0924809	0.2028666	3.29	5.25	30.50	73.25
NUT	NUT1091	0.6801787	0.2957139	0.460324	0.74	1.13	6.00	15.50
NUT	NUT1409	0.6807605	0.0103191	0.0356242	11.67	18.71	106.75	248.00
ORF-T	YOR062C	0.6809226	0.0144849	0.0472705	11.90	19.15	111.25	245.75
SRT	SRT281	0.6811944	0.1748487	0.320718	1.26	1.99	11.25	26.75

Class	Transcript name	RRP6 KO_vs_WT log2_fold _change	RRP6 KO_vs_WT p-value	RRP6 KO_vs_WT FDR	Ave Norm Reads in WT	Ave Norm Reads in RRP6	Average RAW read counts in WT	Average RAW read counts in RRP6
SRT	SRT278	0.6815365	0.0319513	0.0903378	3.94	6.25	35.00	84.25
SUT	SUT023	0.6821972	0.0910486	0.2003784	2.74	4.38	24.50	56.75
SUT	SUT684	0.6854209	0.0907568	0.1998671	1.32	2.13	12.00	27.75
SUT	SUT381	0.6872008	0.1786389	0.3256907	1.35	2.18	12.50	29.50
SRT	SRT28	0.6872261	0.1959122	0.3458513	0.89	1.40	7.50	17.50
SUT	SUT266	0.6875045	0.1150053	0.2380352	1.83	2.95	16.25	36.25
ORF-T	YLR122C	0.6920712	0.0303542	0.0866135	3.90	6.39	37.75	83.75
SUT	SUT165	0.6927278	0.0400597	0.1081046	2.78	4.47	25.50	60.75
NUT	NUT1490	0.692746	0.0410344	0.1101687	2.57	4.11	23.00	54.25
SUT	SUT655	0.6928841	0.0344329	0.0958116	3.57	5.77	32.00	73.25
SRT	SRT546	0.6929339	0.1935601	0.343154	0.78	1.30	7.25	15.75
sn/snoRNA ET	SNR74-ET	0.693151	0.0402201	0.1084148	45.42	73.43	393.75	902.75
ORF-T	YOR192C	0.6947817	0.0078868	0.0283798	14.24	23.04	129.00	302.75
CUT	CUT463	0.6950666	0.0753695	0.174423	1.47	2.38	13.50	31.75
AST	AS_YPR114W	0.6955784	0.272164	0.435234	0.73	1.19	6.50	14.75
ORF-T	YDR106W	0.6959126	0.0441938	0.1164705	2.87	4.70	26.75	60.25
SUT	SUT846	0.6965555	0.0783883	0.1792833	1.96	3.15	17.50	41.75
NUT	NUT0219	0.6974969	0.047771	0.1237408	56.35	91.46	524.75	1124.75
ORF-T	YMR306C-A	0.6988578	0.0942382	0.2059597	1.19	1.90	10.25	24.00
NUT	NUT0196	0.6996093	0.0715567	0.1676466	1.68	2.75	16.00	37.50
ORF-T	YLR254C	0.7010306	0.0186838	0.0582868	6.38	10.40	59.75	139.75
SRT	SRT274	0.7029885	0.1543583	0.2939659	0.59	1.05	6.00	13.00
AST	AS_YPL256C	0.7044381	0.188976	0.3380582	0.86	1.41	8.00	18.75
SUT	SUT508	0.7086169	0.0565665	0.1409479	9.56	15.62	83.25	194.00
NUT	NUT0063	0.7091746	0.0494327	0.1267159	2.64	4.37	25.25	56.75
SUT	SUT077	0.7100755	0.0702656	0.1655124	1.23	2.01	11.00	25.50
ORF-T	YJL038C	0.7111197	0.1303383	0.2612466	2.04	3.34	19.00	45.50
sn/snoRNA	SNR14	0.7116394	0.0352164	0.0975877	393.39	644.21	3432.50	8071.00
SUT	SUT564	0.7117504	0.0914222	0.2010253	3.37	5.52	30.75	70.25

TABLE S1: Differential expression data for RRP6 RNA-Seq dataset Page 247

Class	Transcript name	RRP6 KO_vs_WT log2_fold _change	RRP6 KO_vs_WT p-value	RRP6 KO_vs_WT FDR	Ave Norm Reads in WT	Ave Norm Reads in RRP6	Average RAW read counts in WT	Average RAW read counts in RRP6
ORF-T	YOR390W	0.7124111	0.0967139	0.2101071	1.91	3.12	17.25	40.50
NUT	NUT0759	0.7132168	0.072039	0.1682726	2.13	3.42	18.25	45.00
SUT	SUT390	0.7132258	0.0719882	0.1682564	2.13	3.42	18.25	45.00
NUT	NUT0623	0.7132282	0.0035491	0.0144253	26.86	43.97	244.00	591.25
SUT	SUT372	0.7140395	0.0711964	0.1670706	1.33	2.11	11.75	29.25
ORF-T	YGR035C	0.7141002	0.0528026	0.133382	2.99	4.89	26.75	63.25
ORF-T	YKR015C	0.7147335	0.1515831	0.2900887	1.16	1.96	11.25	24.50
SRT	SRT305	0.7147457	0.2151047	0.36962	0.81	1.36	7.75	17.50
ORF-T	YJL047C-A	0.7153265	0.0572261	0.1419602	209.10	343.17	1717.00	4528.75
SUT	SUT512	0.7155749	0.044296	0.1165965	2.20	3.60	20.00	47.50
SUT	SUT653	0.7158277	0.1491861	0.2870757	0.90	1.44	8.00	20.25
ORF-T	YPL272C	0.7159698	0.0404281	0.1088589	3.75	6.09	33.75	86.00
ORF-T	YIL089W	0.7180753	0.0172793	0.0547594	4.73	7.82	44.25	102.00
SUT	SUT728	0.7182575	0.046085	0.1203633	3.84	6.26	33.75	83.25
SUT	SUT373	0.7185683	0.1588908	0.3001507	1.22	1.99	11.00	26.75
sn/snoRNA ET	SNR50-ET	0.7192501	0.0278493	0.0806062	21.90	36.03	193.00	451.75
CUT	CUT139	0.7202477	0.1550584	0.2950206	0.57	0.92	5.00	11.75
SUT	SUT355	0.7211186	0.0132782	0.0440741	9.44	15.51	86.25	213.50
ORF-T	YER076W-A	0.7221756	0.1055238	0.2241158	0.93	1.53	8.75	20.75
SRT	SRT578	0.722448	0.1558868	0.2962056	0.57	0.99	5.75	13.25
CUT	CUT546	0.7231154	0.0772691	0.1774496	3.48	5.75	32.25	76.50
NUT	NUT1378	0.7232267	0.1079431	0.2277045	0.75	1.31	7.25	16.25
NUT	NUT0126	0.7232657	0.0312205	0.0885831	2.67	4.37	24.00	58.25
CUT	CUT465	0.7233666	0.0342531	0.095364	3.94	6.57	37.00	83.50
CUT	CUT490	0.7244868	0.2311221	0.3891527	0.38	0.55	3.00	7.75
ORF-T	YKR034W	0.7245989	0.1875524	0.3365863	0.64	1.07	6.25	14.75
CUT	CUT511	0.7250688	0.1444235	0.2810997	0.82	1.38	7.75	18.25
NUT	NUT1239	0.7257674	0.0458735	0.1199346	2.45	4.08	22.75	53.50
NUT	NUT0763	0.7258189	0.0856703	0.191625	1.95	3.21	18.00	44.75

TABLE S1: Differential expression data for RRP6 RNA-Seq dataset Page 248

Class	Transcript name	RRP6 KO_vs_WT log2_fold_change	RRP6 KO_vs_WT p-value	RRP6 KO_vs_WT FDR	Ave Norm Reads in WT	Ave Norm Reads in RRP6	Average RAW read counts in WT	Average RAW read counts in RRP6
AST	AS_YEL017W	0.7262581	0.0439892	0.1161223	1.87	3.16	18.00	41.50
SUT	SUT759	0.7264655	0.037228	0.1019296	2.13	3.55	19.75	45.75
CUT	CUT707	0.7268366	0.0435728	0.1152644	1.87	3.07	17.00	40.75
CUT	CUT070	0.7269871	0.1572721	0.2980515	0.56	0.97	5.50	12.75
ORF-T	YMR320W	0.7271092	0.0123485	0.0414523	11.24	18.60	104.00	250.00
CUT	CUT282	0.7273228	0.129442	0.2599851	1.75	2.85	14.75	37.25
SUT	SUT483	0.7277536	0.0787512	0.1798294	1.95	3.18	17.00	41.75
SUT	SUT546	0.7282466	0.1550946	0.2950338	2.37	3.96	22.75	52.50
NUT	NUT0603	0.7289347	0.0860689	0.1921023	1.13	1.84	10.00	24.50
CUT	CUT690	0.7296075	0.1711297	0.3157101	0.98	1.64	9.25	21.50
ORF-T	YLR123C	0.7311085	0.0363714	0.1000707	3.14	5.30	30.75	70.00
NUT	NUT1267	0.7332418	0.0472804	0.1226589	2.38	4.05	23.50	52.50
AST	AS_YDL137W	0.7333005	0.1031748	0.2206778	1.21	2.02	11.50	27.50
AST	AS_YML125C	0.7340857	0.1484354	0.2861993	1.92	3.18	18.25	46.75
AST	AS_YFL051C	0.7349957	0.0497971	0.127515	6.02	10.07	55.25	123.25
ORF-T	YPL192C	0.7366431	0.1247239	0.2528177	1.22	2.06	11.25	25.25
NUT	NUT1296	0.7381143	0.0363853	0.1000707	3.98	6.58	35.00	87.75
SUT	SUT571	0.7394322	0.0349666	0.0969772	9.22	15.44	85.00	193.75
AST	AS_YPL084W	0.7403705	0.0727607	0.1695257	1.01	1.70	9.50	22.75
ORF-T	YNL251C	0.7404594	0.0206022	0.0629935	19.77	32.94	170.00	431.00
SUT	SUT092	0.7406988	0.0568251	0.1413736	1.62	2.73	15.50	37.25
NUT	NUT0080	0.741092	0.0626941	0.1520825	1.52	2.59	14.75	34.25
NUT	NUT0564	0.7414348	0.0109914	0.0376487	5.85	9.81	54.25	127.25
ORF-T	YMR141C	0.7418278	0.2105559	0.363538	0.96	1.58	8.75	22.75
SUT	SUT249	0.7442169	0.1401207	0.2752732	0.66	1.19	6.75	14.75
NUT	NUT0820	0.7444083	0.01619	0.0518971	8.73	14.55	77.00	190.25
SUT	SUT414	0.7445946	0.0418914	0.111814	2.06	3.38	17.75	43.75
NUT	NUT0158	0.7448167	0.0263216	0.0769354	4.71	7.86	42.50	104.50
NUT	NUT1051	0.7452106	0.0203877	0.0624323	9.71	16.35	90.25	207.50

TABLE S1: Differential expression data for RRP6 RNA-Seq dataset Page 249

Class	Transcript name	RRP6 KO_vs_WT log2_fold _change	RRP6 KO_vs_WT p-value	RRP6 KO_vs_WT FDR	Ave Norm Reads in WT	Ave Norm Reads in RRP6	Average RAW read counts in WT	Average RAW read counts in RRP6
NUT	NUT1429	0.7473443	0.0163012	0.0521846	13.97	23.34	118.00	296.00
AST	AS_YBR173C	0.7476536	0.0890368	0.1970259	2.08	3.47	19.50	49.25
ORF-T	YBR056C-B	0.7477615	0.1908157	0.3400822	0.46	0.77	4.25	10.25
NUT	NUT0310	0.7482284	0.0249706	0.0736482	7.45	12.58	69.75	161.00
ORF-T	YLR205C	0.7483188	0.0246858	0.0729362	10.85	18.33	102.50	229.75
NUT	NUT0965	0.748941	0.0273365	0.0794642	404.69	680.09	3538.00	8514.00
SUT	SUT348	0.7493379	0.0683026	0.1619355	1.22	2.05	11.00	26.25
NUT	NUT0555	0.749385	0.0478007	0.1237675	2.36	3.92	20.25	49.75
NUT	NUT1516	0.7502016	0.0400355	0.1080905	4.30	7.22	38.25	92.25
NUT	NUT0380	0.7503367	0.0898774	0.1981902	4.71	7.98	45.00	99.25
SUT	SUT556	0.7505831	0.0702186	0.1654466	1.01	1.74	10.00	23.75
ORF-T	YBR099C	0.7507413	0.1096007	0.2299677	1.24	2.16	12.25	26.75
ORF-T	YBR032W	0.7529082	0.3103541	0.4757228	0.50	0.89	5.50	12.25
NUT	NUT1261	0.7547113	0.0371954	0.1018681	4.91	8.25	42.75	104.75
SUT	SUT701	0.7547118	0.0371888	0.1018681	4.91	8.25	42.75	104.75
CUT	CUT131	0.7552099	0.1641025	0.3071857	1.42	2.47	14.75	33.25
NUT	NUT0821	0.7555181	0.0137018	0.0451828	8.73	14.66	77.00	191.75
NUT	NUT0105	0.7556568	0.1287144	0.2589084	0.71	1.19	6.75	16.75
AST	AS_YPL023C	0.7572768	0.1951069	0.3451075	0.85	1.47	8.25	19.00
ORF-T	YPR160C-A	0.7601775	0.0980816	0.2122435	0.95	1.59	8.25	20.25
NUT	NUT1383	0.7605375	0.0540934	0.1360973	1.47	2.53	14.25	34.25
SUT	SUT186	0.7607311	0.0773238	0.1774946	1.06	1.78	9.50	23.25
SUT	SUT330	0.7615162	0.0073095	0.026716	11.47	19.51	109.50	260.25
AST	AS_YDR460W	0.7641549	0.181246	0.3292532	0.58	0.94	4.75	12.00
ORF-T	YKL006C-A	0.7643161	0.0074092	0.0270313	36.36	61.86	339.25	766.75
NUT	NUT1242	0.7660528	0.0225794	0.0677248	5.31	9.04	48.75	118.50
ORF-T	YIR035C	0.7664857	0.0119979	0.0404772	63.89	108.74	593.50	1348.50
NUT	NUT0418	0.7671013	0.0998202	0.2151746	0.85	1.46	7.75	18.50
SUT	SUT328	0.7674186	0.0061668	0.0232039	14.35	24.39	129.25	321.50

Class	Transcript name	RRP6 KO_vs_WT log2_fold _change	RRP6 KO_vs_WT p-value	RRP6 KO_vs_WT FDR	Ave Norm Reads in WT	Ave Norm Reads in RRP6	Average RAW read counts in WT	Average RAW read counts in RRP6
sn/snoRNA ET	SNR19-ET	0.7677343	0.0093571	0.032797	164.64	280.34	1493.25	3539.50
NUT	NUT1294	0.7681969	0.0303055	0.0865235	1.87	3.13	16.50	41.50
NUT	NUT0961	0.7683617	0.0498925	0.1277269	6.09	10.35	56.25	143.25
NUT	NUT0024	0.7687307	0.0452521	0.1187102	2.19	3.77	21.00	50.75
SUT	SUT369	0.7691502	0.0161543	0.0518444	7.78	13.37	75.25	174.25
NUT	NUT1161	0.7694821	0.0126141	0.0422592	15.33	26.24	145.00	333.00
NUT	NUT0649	0.770609	0.0058037	0.022068	14.28	24.33	128.75	320.50
ORF-T	YPL148C	0.773218	0.0111689	0.0382045	9.28	15.84	82.75	204.75
ORF-T	YDL021W	0.7735662	0.024896	0.0734498	5.26	8.94	46.25	114.00
NUT	NUT0058	0.7740608	0.0925433	0.2029593	0.72	1.21	6.25	15.50
AST	AS_YKL197C	0.7753454	0.1221595	0.2487593	0.61	1.01	5.25	13.00
CUT	CUT534	0.7761225	0.1837876	0.3321947	0.55	0.90	4.50	11.50
NUT	NUT0213	0.7763728	0.1054118	0.2240195	0.61	1.03	5.50	13.75
ORF-T	YPL245W	0.7766126	0.0112926	0.0385885	114.49	196.26	1117.50	2592.25
NUT	NUT1092	0.7767118	0.0574368	0.1422379	1.27	2.20	12.25	30.50
NUT	NUT0785	0.7775636	0.0186872	0.0582868	4.65	7.85	39.75	104.50
CUT	CUT480	0.778896	0.096836	0.2101888	1.55	2.64	14.25	36.00
CUT	CUT564	0.7792917	0.085294	0.191092	0.73	1.24	6.50	16.00
sn/snoRNA ET	SNR77-ET	0.780155	0.0229225	0.0685703	12.23	20.94	103.25	258.75
ORF-T	YGR177C	0.7807833	0.0395657	0.1072241	54.65	93.88	510.25	1305.25
ORF-T	YKL065W-A	0.7820319	0.1073266	0.2270361	1.07	1.87	10.00	23.50
AST	AS_YDR505C	0.7821963	0.088759	0.1966268	1.46	2.50	13.25	33.00
CUT	CUT415	0.7824043	0.1419524	0.2783289	0.89	1.51	7.50	18.75
NUT	NUT0962	0.7833511	0.0515401	0.1309145	1.74	2.95	15.00	37.75
SUT	SUT213	0.7853456	0.0831957	0.1877112	1.52	2.65	14.25	34.25
NUT	NUT0870	0.785494	0.0060989	0.0229911	11.19	19.29	103.75	257.25
SUT	SUT721	0.7861464	0.1159086	0.239094	1.46	2.53	13.00	30.75
SUT	SUT814	0.788158	0.0357002	0.0987657	2.65	4.57	23.50	58.50
NUT	NUT0578	0.7895808	0.0643561	0.1548847	2.61	4.56	25.25	60.50

TABLE S1: Differential expression data for RRP6 RNA-Seq dataset Page 251

Class	Transcript name	RRP6 KO_vs_WT log2_fold _change	RRP6 KO_vs_WT p-value	RRP6 KO_vs_WT FDR	Ave Norm Reads in WT	Ave Norm Reads in RRP6	Average RAW read counts in WT	Average RAW read counts in RRP6
sn/snoRNA ET	SNR57-ET	0.7900529	0.0131515	0.0437417	1763.94	3050.11	15727.50	38167.25
sn/snoRNA ET	SNR39B-ET	0.7920572	0.0864512	0.192785	7.36	12.64	58.75	162.00
SUT	SUT066	0.7930776	0.0300067	0.0857675	3.68	6.30	32.50	85.25
NUT	NUT0453	0.7934889	0.0233868	0.0696288	27.44	47.65	268.25	648.00
NUT	NUT1479	0.7937513	0.0592998	0.145671	2.39	4.12	21.50	55.00
SRT	SRT404	0.7945365	0.1991031	0.3498816	1.59	2.78	14.25	34.75
AST	AS_YBL042C	0.7946806	0.1126991	0.2342148	2.94	5.08	27.75	74.25
SUT	SUT541	0.7956658	0.027567	0.0800628	2.43	4.16	21.00	54.25
NUT	NUT0366	0.7961494	0.014721	0.0478705	3.52	6.10	31.50	77.75
NUT	NUT0345	0.7962976	0.0180271	0.0566771	3.56	6.13	31.75	82.00
NUT	NUT0316	0.7969639	0.039802	0.1076666	6.37	11.15	60.00	135.50
SUT	SUT612	0.7977748	0.2586467	0.4206084	0.48	0.89	5.50	12.25
NUT	NUT1420	0.7991063	0.1107809	0.2316153	3.45	5.98	32.25	88.50
SUT	SUT772	0.7996885	0.0187277	0.0583772	3.32	5.86	31.75	75.25
NUT	NUT1407	0.7997078	0.0187942	0.0585198	3.32	5.86	31.75	75.25
ORF-T	YGR144W	0.800721	0.0493568	0.1266449	1.89	3.35	19.00	46.75
SUT	SUT480	0.801007	0.0190173	0.0590789	3.83	6.71	35.75	88.50
AST	AS_GAP1	0.802332	0.0613874	0.149452	15.12	26.45	143.50	321.00
ORF-T	YNR074C	0.8065496	0.011459	0.0390381	7.88	13.81	72.00	176.25
sn/snoRNA ET	SNR47-ET	0.8072093	0.0226255	0.0678429	85.98	150.41	742.25	1876.50
NUT	NUT0710	0.8086816	0.0388034	0.1055758	1.88	3.26	16.50	42.25
SUT	SUT639	0.809217	0.1069331	0.2264889	0.78	1.32	6.75	17.50
SUT	SUT207	0.8100687	0.0458531	0.1199346	2.12	3.70	19.75	51.75
NUT	NUT0292	0.8106001	0.0649031	0.1558665	1.81	3.15	16.00	42.25
NUT	NUT0358	0.8110447	0.0208245	0.0634423	10.00	17.60	92.00	219.75
SUT	SUT528	0.8115068	0.0042589	0.0169014	5.26	9.19	47.00	118.50
CUT	CUT086	0.8121165	0.039898	0.1078329	1.41	2.44	12.75	32.75
NUT	NUT0099	0.8135734	0.0279789	0.0809581	1.38	2.46	13.00	32.50
NUT	NUT0735	0.817374	0.1425592	0.2787604	0.61	1.07	5.50	14.25

TABLE S1: Differential expression data for RRP6 RNA-Seq dataset Page 252

Class	Transcript name	RRP6 KO_vs_WT log2_fold _change	RRP6 KO_vs_WT p-value	RRP6 KO_vs_WT FDR	Ave Norm Reads in WT	Ave Norm Reads in RRP6	Average RAW read counts in WT	Average RAW read counts in RRP6
ORF-T	YLR171W	0.8184443	0.0975566	0.2112887	0.59	0.96	5.00	13.50
ORF-T	YOR050C	0.8198693	0.2486312	0.4096059	0.85	1.48	7.75	21.25
SUT	SUT403	0.8201161	0.0478304	0.1237675	2.57	4.52	22.75	58.25
ORF-T	YJL035C	0.8210271	0.0067832	0.0251569	14.74	26.17	140.75	329.25
CUT	CUT229	0.8212152	0.0368507	0.1011437	4.80	8.54	46.25	115.25
NUT	NUT0510	0.8240887	0.0296882	0.085011	2.16	3.79	19.00	49.50
SUT	SUT148	0.8255863	0.0402177	0.1084148	1.47	2.55	12.75	33.25
ORF-T	YPL223C	0.8257863	0.0264307	0.0771649	3.04	5.45	28.50	68.50
sn/snoRNA	SNR6	0.8270361	0.0220853	0.0665608	94.41	167.41	800.00	2115.25
AST	AS_YHR037W	0.8275554	0.1830771	0.3312668	0.54	0.98	5.50	14.00
NUT	NUT1180	0.8276333	0.0063466	0.0237561	14.84	26.47	141.75	333.50
AST	AS_YOL060C	0.827754	0.2215219	0.3777775	1.25	2.29	13.75	31.50
ORF-T	YPR145C-A	0.8280078	0.0699744	0.1650578	1.82	3.19	15.50	41.50
NUT	NUT1117	0.8281765	0.0084079	0.0299172	3.45	6.09	30.50	77.75
CUT	CUT376	0.8293843	0.1464118	0.283599	1.05	1.87	10.00	26.50
SUT	SUT173	0.8332252	0.1086456	0.2287229	1.48	2.61	13.50	36.50
AST	AS_YMR210W	0.8339773	0.0539133	0.1357458	1.45	2.51	12.50	33.75
SUT	SUT595	0.835372	0.0077175	0.0279069	3.45	6.11	30.50	78.25
NUT	NUT0040	0.8365757	0.0469126	0.1218931	1.36	2.49	13.00	31.75
SUT	SUT402	0.8369839	0.0533446	0.1344146	1.06	1.95	10.25	25.75
NUT	NUT0471	0.8402788	0.1174121	0.2410756	0.79	1.49	8.25	20.00
SRT	SRT384	0.8409438	0.1447523	0.2814659	0.55	0.94	4.50	12.00
NUT	NUT0835	0.8412304	0.0197209	0.0607965	2.88	5.33	29.75	68.50
AST	AS_YHL032C	0.8437539	0.1860603	0.3348023	0.87	1.61	9.25	23.25
NUT	NUT0315	0.844213	0.0188925	0.0587638	2.89	5.22	27.25	70.00
NUT	NUT0867	0.844729	0.0242498	0.0717952	2.42	4.30	21.50	58.00
AST	AS_YBL058W	0.8456001	0.0633865	0.153136	0.82	1.50	7.75	19.75
SUT	SUT356	0.8458631	0.0217893	0.0658644	2.23	3.95	19.50	52.75
SUT	SUT611	0.8461678	0.0110431	0.0378001	3.48	6.33	33.25	84.00

Class	Transcript name	RRP6 KO_vs_WT log2_fold_change	RRP6 KO_vs_WT p-value	RRP6 KO_vs_WT FDR	Ave Norm Reads in WT	Ave Norm Reads in RRP6	Average RAW read counts in WT	Average RAW read counts in RRP6
AST	AS_YDR110W	0.8467397	0.1685424	0.3125954	0.53	1.00	5.50	13.50
NUT	NUT1318	0.8473306	0.0502785	0.1283891	1.45	2.53	12.50	34.00
NUT	NUT0766	0.8476624	0.0827671	0.1870096	0.63	1.10	5.50	14.75
NUT	NUT0209	0.8488271	0.0187967	0.0585198	2.78	5.00	25.00	65.25
NUT	NUT1153	0.8491853	0.044284	0.1165954	2.31	4.17	21.25	54.25
NUT	NUT1046	0.8502678	0.0120495	0.0406244	3.85	6.95	35.50	90.50
SUT	SUT797	0.850995	0.0343545	0.0956199	5.57	10.00	48.00	125.00
CUT	CUT004	0.8533058	0.0170582	0.0541437	4.30	7.79	39.75	102.75
NUT	NUT0831	0.8533231	0.0161465	0.0518375	2.60	4.68	24.00	63.25
NUT	NUT1075	0.8551713	0.0188168	0.0585644	2.24	4.09	20.25	50.25
SUT	SUT625	0.8552371	0.0225326	0.0676047	2.26	4.08	20.25	51.75
SUT	SUT017	0.8553794	0.031229	0.0885831	4.03	7.33	38.25	96.25
ORF-T	YPL279C	0.8555066	0.0278422	0.0806062	1.06	1.90	9.75	25.75
SUT	SUT559	0.8556495	0.085098	0.1909072	1.41	2.56	12.75	33.00
ORF-T	YDL183C	0.8584674	0.0590561	0.1453205	2.72	4.97	26.25	65.25
SRT	SRT313	0.8589643	0.194829	0.3447356	0.54	1.02	5.50	13.50
SUT	SUT281	0.8596207	0.0281125	0.0812515	3.47	6.25	31.75	86.25
NUT	NUT0547	0.8596234	0.0280894	0.0812331	3.47	6.25	31.75	86.25
NUT	NUT1053	0.8602094	0.0196493	0.0606315	2.37	4.35	22.00	55.50
NUT	NUT0885	0.8604361	0.0168046	0.0535412	3.41	6.29	33.25	80.75
AST	AS_YDR340W	0.8611301	0.0187736	0.0585021	4.10	7.39	36.25	97.50
CUT	CUT220	0.8617804	0.0630726	0.1526701	0.66	1.16	5.75	15.25
ORF-T	YDR194W-A	0.862288	0.0776474	0.178075	1.06	1.86	9.00	25.25
ORF-T	YMR018W	0.8632912	0.0466386	0.1215255	3.51	6.46	33.50	80.00
SUT	SUT130	0.8636861	0.0334777	0.0936187	4.58	8.38	43.75	114.00
CUT	CUT395	0.864787	0.0173463	0.0549199	1.41	2.54	12.50	32.75
NUT	NUT0184	0.8670265	0.0441911	0.1164705	1.53	2.83	15.00	38.75
NUT	NUT0827	0.8670824	0.0082033	0.0292879	5.96	10.90	57.00	149.25
CUT	CUT554	0.8679101	0.0261824	0.0766004	3.82	7.03	36.50	93.50

Class	Transcript name	RRP6 KO_vs_WT log2_fold _change	RRP6 KO_vs_WT p-value	RRP6 KO_vs_WT FDR	Ave Norm Reads in WT	Ave Norm Reads in RRP6	Average RAW read counts in WT	Average RAW read counts in RRP6
CUT	CUT445	0.8691153	0.0132362	0.0439494	3.03	5.70	31.00	73.75
NUT	NUT0699	0.8710494	0.0021606	0.0093467	7.68	13.98	67.75	180.25
NUT	NUT0541	0.8723918	0.0812747	0.1842567	0.91	1.70	9.00	23.75
ORF-T	YCR020C	0.8727338	0.0481136	0.1243088	2.68	4.82	23.25	68.75
ORF-T	YCR098C	0.8735439	0.0029751	0.0123666	6.11	11.25	57.00	145.75
NUT	NUT0711	0.8749587	0.0666299	0.159047	0.86	1.56	8.00	21.75
NUT	NUT0982	0.875444	0.0045723	0.0179545	9.14	16.74	81.75	210.00
NUT	NUT0746	0.8776931	0.0161749	0.0518791	1.38	2.51	12.25	32.25
ORF-T	YPR150W	0.8784631	0.0770914	0.1772649	1.53	2.81	13.25	35.00
NUT	NUT0264	0.8811148	0.0866311	0.1930581	0.69	1.17	5.50	16.00
NUT	NUT1144	0.8812106	0.2471222	0.4077193	0.48	0.95	5.75	13.75
AST	AS_YKR050W	0.8813065	0.1229541	0.2500136	0.55	1.03	5.25	13.75
NUT	NUT0586	0.8815431	0.0770688	0.1772649	0.82	1.50	7.50	20.75
sn/snoRNA	SNR57	0.8816044	0.0065804	0.0245129	1584.47	2919.27	14132.50	36524.25
SRT	SRT333	0.8821639	0.0986967	0.2133	1.08	2.00	10.00	26.25
SUT	SUT231	0.8825681	0.0666663	0.159078	0.78	1.36	6.50	18.00
NUT	NUT1000	0.8833995	0.0049841	0.0193903	3.53	6.41	30.75	84.25
NUT	NUT0501	0.8835758	0.0069264	0.0255657	8.22	15.14	74.25	199.25
SUT	SUT253	0.8835768	0.0069173	0.0255477	8.22	15.14	74.25	199.25
SUT	SUT341	0.8836677	0.2008689	0.3519045	0.58	1.05	5.00	13.75
NUT	NUT1156	0.8852372	0.0722964	0.1686805	0.93	1.70	8.50	23.25
SUT	SUT202	0.8877473	0.0155558	0.0501806	2.18	4.03	20.00	53.75
NUT	NUT0751	0.8881211	0.0126937	0.0424978	2.30	4.33	21.75	54.50
AST	AS_YMR072W	0.8897373	0.0089626	0.0315678	2.85	5.25	25.75	69.75
SUT	SUT016	0.8903375	0.0024447	0.0103888	16.81	31.21	156.75	409.00
SUT	SUT735	0.8907413	0.0595634	0.1461404	1.04	2.00	9.75	23.50
SUT	SUT753	0.8913438	0.0078886	0.0283798	6.49	12.03	57.50	153.75
NUT	NUT0808	0.8916899	0.0661428	0.1582407	1.62	3.00	14.00	37.50
SUT	SUT622	0.8923277	0.0237747	0.070596	2.10	3.89	19.25	51.25

Class	Transcript name	RRP6 KO_vs_WT log2_fold _change	RRP6 KO_vs_WT p-value	RRP6 KO_vs_WT FDR	Ave Norm Reads in WT	Ave Norm Reads in RRP6	Average RAW read counts in WT	Average RAW read counts in RRP6
SUT	SUT319	0.8925908	0.060448	0.1478433	1.70	3.22	16.00	39.25
ORF-T	YDL094C	0.8928091	0.0519188	0.1315451	0.66	1.17	5.75	15.75
NUT	NUT0031	0.893154	0.0023798	0.0101688	16.46	30.61	153.50	401.50
SUT	SUT768	0.8938986	0.0022274	0.0095957	9.07	16.86	83.50	223.00
SUT	SUT055	0.8941026	0.0008801	0.0041982	10.19	19.01	95.25	244.25
NUT	NUT1358	0.8945458	0.0676505	0.1608175	0.58	1.07	5.25	14.00
ORF-T	YDR380W	0.8948565	0.1962368	0.3462217	25.24	46.88	224.75	747.75
SRT	SRT173	0.8966883	0.1252257	0.2534677	0.51	0.97	5.25	14.50
SRT	SRT495	0.8974342	0.000637	0.0031226	14.68	27.28	131.00	359.75
ORF-T	YGR131W	0.9000789	0.0044535	0.0175634	4.61	8.52	40.00	109.00
NUT	NUT1168	0.9032355	0.0019933	0.0087296	6.81	12.72	62.75	171.25
SUT	SUT828	0.903725	0.0062681	0.0235146	3.18	5.91	29.25	81.50
CUT	CUT622	0.9039736	0.0413342	0.1106634	0.77	1.43	7.25	20.00
SUT	SUT799	0.9040593	0.0033074	0.0135742	4.67	8.88	45.75	116.25
ORF-T	YMR182C	0.9057574	0.0783358	0.1792056	0.68	1.21	5.75	16.25
NUT	NUT0563	0.9062898	0.2012572	0.3523398	0.91	1.71	8.50	22.75
ORF-T	YJL088W	0.9072008	0.1839554	0.3322597	0.54	0.96	4.50	13.50
NUT	NUT1388	0.9075545	0.0992662	0.2142553	0.64	1.17	5.50	15.00
SUT	SUT086	0.9102123	0.0283029	0.081755	3.74	6.98	31.75	85.75
CUT	CUT723	0.9116822	0.1362823	0.2702194	0.34	0.71	3.75	9.75
CUT	CUT281	0.9123552	0.0324183	0.0913802	10.60	19.94	97.75	267.75
NUT	NUT0386	0.9131166	0.0034948	0.0142447	4.02	7.52	35.75	97.75
ORF-T	YDR259C	0.9137477	0.0564736	0.1408208	0.93	1.76	8.75	23.50
ORF-T	YBR012C	0.9140046	0.0401008	0.1081799	3.93	7.45	36.75	95.25
NUT	NUT0722	0.9140238	0.00322	0.0132532	4.38	8.19	39.00	109.00
SUT	SUT062	0.9146214	0.0271735	0.0790359	1.47	2.77	13.00	34.50
SRT	SRT8	0.9151982	0.0526044	0.1330148	4.74	8.98	42.75	105.75
NUT	NUT0330	0.9162786	0.026376	0.0770496	1.18	2.14	10.00	28.75
NUT	NUT0976	0.9167357	0.0001336	0.0007939	20.33	38.42	188.50	503.25

Class	Transcript name	RRP6 KO_vs_WT log2_fold _change	RRP6 KO_vs_WT p-value	RRP6 KO_vs_WT FDR	Ave Norm Reads in WT	Ave Norm Reads in RRP6	Average RAW read counts in WT	Average RAW read counts in RRP6
SUT	SUT793	0.9178211	0.0232115	0.0692274	3.81	7.25	35.25	91.00
SUT	SUT158	0.9195015	0.0151561	0.0491428	3.38	6.46	32.25	83.50
AST	AS_YBR054W	0.9204994	0.089338	0.1975191	1.08	2.05	10.00	27.00
ORF-T	YGR050C	0.9206569	0.0052654	0.0202969	5.81	11.03	53.75	144.00
NUT	NUT0860	0.9207087	0.0198095	0.0609952	2.95	5.65	27.50	71.25
NUT	NUT0039	0.9207801	0.0353935	0.0979978	2.44	4.63	22.50	60.50
ORF-T	YGR269W	0.9210482	0.0093282	0.0327072	2.32	4.35	20.25	55.00
ORF-T	YFL055W	0.9217566	0.0006817	0.0033207	17.68	33.50	163.75	444.50
sn/snoRNA ET	SNR79-ET	0.9231348	0.0265433	0.0774486	2.46	4.68	21.75	56.75
NUT	NUT0625	0.9243357	0.0210114	0.0639731	1.50	2.79	13.00	36.75
ORF-T	YPR157W	0.9244431	0.0073454	0.026818	13.62	25.86	126.75	335.75
SUT	SUT091	0.9262921	0.0097347	0.0338966	5.86	11.18	55.00	146.50
CUT	CUT087	0.9264834	0.0353382	0.0978983	1.17	2.25	11.25	30.75
NUT	NUT1289	0.9271416	0.0146654	0.0477512	1.70	3.21	15.00	41.50
NUT	NUT0218	0.9285369	0.0004196	0.0021623	23.15	44.08	213.50	581.00
NUT	NUT0760	0.9290802	0.0094196	0.0329588	1.63	3.16	15.50	41.25
SUT	SUT800	0.9292281	0.0076323	0.0276981	4.43	8.40	40.25	112.50
NUT	NUT0671	0.9298387	0.0328297	0.0924218	1.02	1.95	9.50	26.00
ORF-T	YDR523C	0.9331376	0.0181307	0.0568502	4.37	8.42	42.25	112.50
AST	AS_YML123C	0.9338692	0.0284104	0.0820185	9.74	18.60	89.75	249.00
AST	AS_YDL123W	0.9340323	0.0208182	0.0634423	1.82	3.42	15.75	44.00
NUT	NUT0882	0.9347802	0.00061	0.0030036	8.80	16.88	82.25	220.50
NUT	NUT1475	0.9364646	0.0023301	0.0099814	9.88	18.90	88.25	243.50
SUT	SUT434	0.9388357	0.008616	0.030507	4.53	8.67	40.50	108.25
SUT	SUT679	0.9389508	0.0028487	0.011885	7.21	13.82	65.50	178.25
SUT	SUT517	0.9389753	0.0856967	0.191625	0.80	1.52	7.25	20.50
ORF-T	YDR396W	0.9397819	0.1038661	0.2217159	0.53	1.00	4.75	13.25
SUT	SUT764	0.9398429	0.0190869	0.0592096	3.27	6.21	29.25	88.25
CUT	CUT402	0.941374	0.0083725	0.0298017	1.61	3.14	15.25	41.00

Class	Transcript name	RRP6 KO_vs_WT log2_fold _change	RRP6 KO_vs_WT p-value	RRP6 KO_vs_WT FDR	Ave Norm Reads in WT	Ave Norm Reads in RRP6	Average RAW read counts in WT	Average RAW read counts in RRP6
SRT	SRT15	0.9430942	0.0047991	0.0187284	5.03	9.68	47.75	134.25
CUT	CUT582	0.9434861	0.0235703	0.0700719	1.77	3.47	17.00	44.00
NUT	NUT0090	0.9449818	0.0483128	0.1247595	0.62	1.20	5.75	15.75
ORF-T	YOR053W	0.9453938	0.0801592	0.1824251	0.87	1.64	7.25	20.75
SUT	SUT228	0.9479008	0.0007424	0.0035925	17.74	34.26	161.50	436.00
NUT	NUT0898	0.9483141	0.0337698	0.0942787	1.86	3.60	17.50	48.50
NUT	NUT0794	0.9498647	0.0949433	0.2070073	0.66	1.28	6.00	16.25
ORF-T	YHR092C	0.9518775	0.1579217	0.2988893	203.15	392.94	1692.25	4850.75
SRT	SRT379	0.95496	0.0119259	0.0402884	1.28	2.45	11.25	31.25
NUT	NUT1471	0.9566285	0.0306686	0.0873895	1.15	2.24	10.75	30.25
NUT	NUT0876	0.9568231	0.041194	0.11042	1.03	2.01	9.75	27.75
NUT	NUT0621	0.957447	0.0165129	0.0527783	1.50	3.01	14.50	37.50
ORF-T	YLR307C-A	0.9606051	0.1588019	0.3001507	1.26	2.48	12.50	34.00
NUT	NUT1326	0.9608915	0.0009104	0.0043283	7.05	13.82	67.00	179.75
SUT	SUT489	0.9618625	0.0061621	0.0231948	2.86	5.52	25.50	72.00
NUT	NUT0423	0.9626686	0.0021813	0.00942	3.43	6.67	31.25	88.25
NUT	NUT0050	0.9627347	0.0278094	0.0805603	2.50	4.85	23.25	68.25
NUT	NUT1253	0.9630671	0.0058558	0.0222074	3.01	5.82	26.75	78.25
SUT	SUT588	0.9633701	0.0134188	0.0444239	2.21	4.30	20.25	57.25
NUT	NUT1393	0.9634141	0.0127562	0.042654	1.94	3.81	17.75	48.00
NUT	NUT0704	0.9636527	0.0622798	0.1511504	0.90	1.83	9.00	23.00
NUT	NUT1021	0.9642167	0.0049666	0.0193372	2.61	5.03	22.75	65.50
NUT	NUT0616	0.9643226	0.0396375	0.1073855	1.70	3.38	16.00	41.75
SUT	SUT598	0.9654278	0.0468599	0.1218503	0.69	1.38	6.50	17.75
SRT	SRT438	0.9654364	0.0468897	0.1218648	0.69	1.38	6.50	17.75
SUT	SUT323	0.9668995	0.0109085	0.037428	1.90	3.73	17.50	48.75
SRT	SRT596	0.9674107	0.1645724	0.3077506	0.37	0.75	3.75	10.50
NUT	NUT0171	0.9679618	0.0074646	0.0271647	2.00	3.96	18.50	49.75
ORF-T	YNR075W	0.9687611	0.0012455	0.0057142	10.23	19.99	92.25	264.50

Class	Transcript name	RRP6 KO_vs_WT log2_fold _change	RRP6 KO_vs_WT p-value	RRP6 KO_vs_WT FDR	Ave Norm Reads in WT	Ave Norm Reads in RRP6	Average RAW read counts in WT	Average RAW read counts in RRP6
NUT	NUT1418	0.9696268	0.0202125	0.0620089	1.13	2.23	10.75	30.25
AST	AS_YBR011C	0.9699209	0.0675394	0.160629	0.89	1.80	9.00	24.25
sn/snoRNA ET	SNR75-ET	0.9704215	0.0045264	0.0177952	20.89	40.93	183.75	499.50
NUT	NUT1215	0.9753182	0.0106019	0.0365251	1.93	3.74	17.00	49.50
NUT	NUT1297	0.9769807	0.0165346	0.0528311	1.05	1.98	9.00	26.50
NUT	NUT1137	0.9775355	0.0068061	0.0251956	3.48	6.80	31.50	92.25
NUT	NUT0938	0.9792879	0.051345	0.1305833	0.85	1.73	8.75	24.25
CUT	CUT573	0.97987	0.0627855	0.152231	0.58	1.13	5.25	15.25
ORF-T	YNL276C	0.9801982	0.0149928	0.0486601	3.30	6.43	29.00	89.00
AST	AS_YDR490C	0.9806253	0.0042476	0.0168631	2.02	3.95	18.50	53.75
NUT	NUT0939	0.9809166	0.0045185	0.0177776	2.86	5.60	25.50	72.75
CUT	CUT089	0.9811894	0.0205322	0.0627986	8.26	16.35	76.50	209.50
AST	AS_YDR076W	0.9814167	0.0036558	0.0148113	3.24	6.37	29.25	85.25
SUT	SUT187	0.983868	0.0005992	0.0029573	12.13	24.10	116.50	320.25
AST	AS_YDR079C-A	0.9841108	0.0493272	0.1266158	0.52	1.04	4.75	13.50
CUT	CUT123	0.9847735	0.0596271	0.1462071	0.51	0.99	4.50	13.00
NUT	NUT0933	0.9850942	0.0024419	0.0103888	5.05	10.05	48.25	135.00
CUT	CUT792	0.9872384	0.0039463	0.0158543	3.52	7.08	34.00	92.50
NUT	NUT0673	0.9874996	0.0067004	0.0248774	1.77	3.52	16.25	45.75
SUT	SUT777	0.9881672	0.088945	0.1969091	3.35	6.68	32.75	89.75
AST	AS_YNL128W	0.9887279	0.0583706	0.1441596	0.50	1.01	4.75	13.50
NUT	NUT1281	0.9906721	0.0138507	0.0455476	5.66	11.26	54.25	160.25
SRT	SRT66	0.9915489	0.0467718	0.1217782	0.71	1.39	6.50	19.75
NUT	NUT1165	0.9918579	0.0485699	0.1252311	0.56	1.07	4.75	13.75
NUT	NUT0191	0.9927179	0.087928	0.1952579	0.74	1.42	6.25	19.00
SUT	SUT001	0.9932673	0.110005	0.2303151	0.70	1.39	6.25	17.25
SUT	SUT239	0.994294	0.0076417	0.027709	2.25	4.49	20.50	58.25
NUT	NUT0369	0.9968126	0.0005491	0.0027426	11.53	23.13	111.00	308.00
NUT	NUT0854	0.9970593	0.0215813	0.0653394	9.96	19.94	96.00	259.50

Class	Transcript name	RRP6 KO_vs_WT log2_fold _change	RRP6 KO_vs_WT p-value	RRP6 KO_vs_WT FDR	Ave Norm Reads in WT	Ave Norm Reads in RRP6	Average RAW read counts in WT	Average RAW read counts in RRP6
SUT	SUT327	0.9984606	0.0018257	0.0080817	2.94	5.94	27.50	75.25
SUT	SUT713	0.9987555	0.0130291	0.0434619	5.66	11.33	54.25	161.00
SRT	SRT509	0.9987556	0.0130362	0.0434714	5.66	11.33	54.25	161.00
NUT	NUT0231	0.9992594	0.0188732	0.0587218	1.11	2.22	10.00	28.25
NUT	NUT1224	0.9994388	0.0576679	0.1427051	0.62	1.21	5.25	15.25
SUT	SUT578	0.9998863	0.0059749	0.0226168	3.99	8.05	38.50	109.00
sn/snoRNA ET	snr6-ET	0.9998974	0.0060737	0.0229131	45.67	91.24	387.50	1156.50
NUT	NUT1243	0.9999047	0.0142978	0.0467846	0.96	1.96	8.75	24.00
ORF-T	YNL334C	1.0006219	0.0756757	0.174999	0.50	0.99	4.50	13.00
SUT	SUT441	1.0016461	0.0180028	0.0566252	1.33	2.75	12.75	33.25
NUT	NUT0680	1.0018624	0.0647648	0.1556453	0.53	1.11	5.50	15.50
NUT	NUT0906	1.0035502	0.0029853	0.0124036	3.24	6.47	29.25	86.75
NUT	NUT0201	1.0037278	0.003014	0.0124971	3.33	6.82	33.25	90.50
ORF-T	YGL183C	1.0037327	0.0141276	0.0463144	1.39	2.85	13.25	35.50
CUT	CUT228	1.0042857	0.0358086	0.0989773	0.65	1.28	5.75	16.75
AST	AS_YFR003C	1.0044714	0.0337574	0.0942787	0.61	1.24	5.75	16.75
CUT	CUT096	1.0044791	0.0487715	0.125551	1.12	2.27	10.25	29.00
SUT	SUT248	1.0045357	0.015289	0.0494941	0.98	1.94	8.50	24.50
ORF-T	YPR092W	1.0048963	0.0531624	0.1340225	0.58	1.08	4.75	14.75
AST	AS_YER109C	1.005961	0.0446235	0.117336	1.32	2.69	13.00	37.50
AST	AS_YJL020C	1.0066616	0.0568354	0.1413736	0.49	1.00	4.50	12.75
NUT	NUT0409	1.008899	0.0196767	0.0606973	1.64	3.34	15.25	42.50
NUT	NUT1218	1.008982	0.0136487	0.045052	1.88	3.84	18.00	50.75
CUT	CUT124	1.0101616	0.0150563	0.0488508	1.84	3.75	17.50	50.25
NUT	NUT0761	1.0111274	0.0071438	0.0261955	2.33	4.81	23.50	65.75
CUT	CUT403	1.0113785	0.0067958	0.0251758	2.43	5.02	24.50	68.50
NUT	NUT0511	1.0118293	0.0005967	0.0029474	58.78	118.65	548.25	1471.25
NUT	NUT1260	1.0120599	0.0028075	0.0117473	2.67	5.42	25.00	71.25
AST	AS_YDR057W	1.0132213	0.1675134	0.3113168	0.34	0.76	4.00	10.25

TABLE S1: Differential expression data for RRP6 RNA-Seq dataset Page 260

Class	Transcript name	RRP6 KO_vs_WT log2_fold _change	RRP6 KO_vs_WT p-value	RRP6 KO_vs_WT FDR	Ave Norm Reads in WT	Ave Norm Reads in RRP6	Average RAW read counts in WT	Average RAW read counts in RRP6
SUT	SUT391	1.0132445	0.0078385	0.0282804	3.58	7.31	34.25	93.25
SUT	SUT734	1.0143443	0.0068872	0.0254585	6.71	13.56	61.75	179.50
NUT	NUT1384	1.0148593	0.0081737	0.0292074	3.53	7.06	31.50	99.00
NUT	NUT0792	1.0152127	0.0046755	0.0182957	2.39	4.78	21.00	61.75
AST	AS_YOL094C	1.0159578	0.0294706	0.0845706	0.89	1.88	9.00	25.00
NUT	NUT1084	1.0166854	0.0134452	0.0444967	1.90	3.93	18.50	51.75
SRT	SRT79	1.0168531	0.086068	0.1921023	0.59	1.21	5.75	16.75
NUT	NUT0688	1.0192485	0.0784299	0.1792988	0.50	0.97	4.25	13.00
SUT	SUT544	1.0198457	0.0010034	0.0047028	8.89	18.02	80.25	229.00
NUT	NUT1344	1.0203871	0.058829	0.145009	0.50	1.07	4.75	12.75
SUT	SUT119	1.0206684	0.0012085	0.0055522	7.12	14.59	69.75	191.25
SUT	SUT069	1.0222901	0.001816	0.008046	4.57	9.22	40.50	122.25
NUT	NUT0399	1.0232851	0.0325228	0.0916089	0.59	1.13	5.00	15.25
SUT	SUT302	1.0236872	0.0015724	0.0070817	4.10	8.30	37.50	112.00
ORF-T	YGL179C	1.0248589	0.0021162	0.0091704	24.77	50.47	228.50	625.25
NUT	NUT1018	1.0253114	0.0021229	0.0091957	24.82	50.59	229.00	626.75
NUT	NUT0262	1.0253188	0.0011422	0.0052836	7.12	14.64	69.75	191.75
NUT	NUT0157	1.0262505	0.0142989	0.0467846	8.34	17.03	77.25	219.00
NUT	NUT0165	1.027029	0.0095208	0.0332323	1.16	2.45	11.25	31.25
SRT	SRT363	1.0284555	0.0094657	0.0330757	2.37	4.86	22.00	63.25
ORF-T	YBR250W	1.0288898	0.0064934	0.0242516	1.62	3.39	15.75	44.50
SUT	SUT844	1.0295469	0.0241411	0.0715154	1.00	2.10	9.75	28.00
NUT	NUT0088	1.0300457	0.0015192	0.0068802	4.01	8.23	38.00	111.75
ORF-T	YCR013C	1.0303479	0.058695	0.1447494	4.07	8.36	39.50	112.50
CUT	CUT918	1.032983	0.0825871	0.1867826	0.66	1.36	6.25	18.00
CUT	CUT105	1.0337104	0.0140672	0.0461612	1.45	3.01	14.00	41.00
NUT	NUT0804	1.0342357	0.0296275	0.0849243	0.84	1.70	7.75	23.25
SUT	SUT730	1.0367167	0.0508723	0.1295774	0.70	1.36	5.75	18.00
NUT	NUT0583	1.0374914	0.0024595	0.0104431	5.09	10.41	45.50	138.25

Class	Transcript name	RRP6 KO_vs_WT log2_fold _change	RRP6 KO_vs_WT p-value	RRP6 KO_vs_WT FDR	Ave Norm Reads in WT	Ave Norm Reads in RRP6	Average RAW read counts in WT	Average RAW read counts in RRP6
NUT	NUT1361	1.0384304	0.0075256	0.0273646	1.42	2.96	13.25	37.75
SUT	SUT510	1.0397096	0.0088533	0.0312267	3.23	6.67	30.25	88.75
NUT	NUT0701	1.0427676	0.0574009	0.1422189	0.58	1.20	5.25	15.75
CUT	CUT018	1.0438877	0.0427832	0.1134734	0.56	1.21	5.25	14.75
AST	AS_YDR233C	1.0448844	0.0322214	0.0909091	0.74	1.54	7.00	20.75
NUT	NUT0078	1.0449476	0.0269909	0.0785729	1.21	2.45	10.75	33.75
AST	AS_YER019C-A	1.0454062	0.0142411	0.0466258	1.07	2.14	9.25	28.50
SUT	SUT739	1.0462877	0.0722904	0.1686805	0.40	0.83	3.75	11.00
CUT	CUT521	1.0476267	0.0132359	0.0439494	1.18	2.40	10.75	33.25
AST	AS_YFL067W	1.0491678	0.0043085	0.0170649	2.06	4.39	20.50	56.25
SUT	SUT082	1.0502118	0.0421621	0.1123284	0.59	1.17	5.00	15.25
NUT	NUT0178	1.0513515	0.0045015	0.0177247	1.59	3.30	14.50	43.25
CUT	CUT336	1.05255	0.0112433	0.0384459	0.76	1.61	7.25	21.25
SUT	SUT468	1.0526158	0.0045211	0.0177811	2.20	4.50	19.50	60.50
sn/snoRNA ET	SNR66-ET	1.0527897	0.0003022	0.0016116	17.19	35.52	147.25	459.50
ORF-T	YBL048W	1.053124	0.0106056	0.0365256	2.41	4.94	20.75	64.25
NUT	NUT0053	1.0537233	0.0031134	0.0128514	4.40	9.12	40.00	121.00
SUT	SUT607	1.0541182	0.005023	0.0195267	2.95	6.10	27.25	83.00
NUT	NUT0781	1.05527	0.005698	0.0217481	3.22	6.65	28.25	85.50
NUT	NUT0470	1.0560368	0.0002519	0.0013799	8.36	17.32	74.00	224.75
NUT	NUT1504	1.0571624	0.0974059	0.2110078	0.62	1.32	6.25	18.50
SUT	SUT455	1.05806	0.0124739	0.0418173	2.41	5.09	22.75	64.25
ORF-T	YOR344C	1.059237	0.0055781	0.021331	7.09	14.68	60.50	192.75
NUT	NUT0427	1.0601208	0.039908	0.1078329	0.66	1.36	6.00	18.50
ORF-T	YNL301C	1.060323	4.82E-05	0.0003228	133.01	277.39	1217.00	3628.00
NUT	NUT0271	1.0607384	0.0398712	0.10782	1.24	2.60	11.25	34.50
SUT	SUT272	1.0630225	0.0080894	0.0289474	1.56	3.20	13.50	41.00
SRT	SRT132	1.0632698	0.0091204	0.032079	1.27	2.66	11.50	33.50
NUT	NUT0925	1.0652586	0.0009154	0.0043503	4.10	8.60	38.00	115.00

Class	Transcript name	RRP6 KO_vs_WT log2_fold _change	RRP6 KO_vs_WT p-value	RRP6 KO_vs_WT FDR	Ave Norm Reads in WT	Ave Norm Reads in RRP6	Average RAW read counts in WT	Average RAW read counts in RRP6
AST	AS_YHR014W	1.0677055	0.0049349	0.0192284	2.93	6.19	28.00	83.75
NUT	NUT1299	1.0681157	0.0070766	0.0259964	1.28	2.64	11.50	35.25
ORF-T	YIL134W	1.0683789	1.49E-05	0.000114	21.68	45.47	195.75	575.50
NUT	NUT0934	1.0687447	0.0053204	0.0204775	1.09	2.24	9.50	28.75
sn/snoRNA	SNR75	1.0690327	0.0047221	0.0184564	67.17	140.92	585.25	1675.25
AST	AS_YDR453C	1.0696629	0.0296592	0.0849911	0.68	1.52	6.75	18.50
ORF-T	YGL158W	1.0698075	0.0280919	0.0812331	31.08	65.29	279.75	744.50
CUT	CUT823	1.0707613	0.0162127	0.0519506	1.06	2.25	9.75	28.25
NUT	NUT1377	1.0709409	0.0018763	0.0082804	3.27	6.93	31.00	92.25
SUT	SUT488	1.0728738	0.0146835	0.0477793	1.22	2.51	10.25	31.25
SRT	SRT368	1.0756581	0.0189687	0.0589492	1.60	3.41	14.75	41.25
SRT	SRT89	1.0766342	0.0369483	0.1013841	0.48	1.04	4.75	14.50
AST	AS_YGL064C	1.0785487	0.0026484	0.0111604	1.98	4.19	18.25	54.75
NUT	NUT0964	1.0790052	0.0064729	0.0241929	1.45	2.97	12.50	40.00
NUT	NUT0525	1.0819042	0.0069914	0.0257491	1.56	3.25	13.50	41.50
NUT	NUT0650	1.0819588	0.012686	0.0424861	1.41	2.94	12.25	38.50
SUT	SUT473	1.0821461	0.0102099	0.0353076	1.63	3.40	14.00	44.00
NUT	NUT0907	1.0835901	0.0098683	0.0342438	2.01	4.21	17.25	54.00
SUT	SUT494	1.0842149	0.0155911	0.0502624	2.24	4.80	22.00	65.75
NUT	NUT0602	1.0865994	0.028287	0.0817323	0.53	1.09	4.50	13.75
CUT	CUT866	1.0886423	0.0020378	0.0088956	4.19	8.79	35.50	112.75
CUT	CUT473	1.089115	0.0028429	0.0118659	3.86	8.14	33.25	105.25
SUT	SUT240	1.0891762	0.0001713	0.0009828	8.04	17.03	71.00	221.00
AST	AS_YBR038W	1.0892612	0.0641864	0.1545501	0.65	1.34	5.50	17.50
ORF-T	YFL062W	1.0906758	0.0300425	0.0858453	0.82	1.82	8.00	22.75
NUT	NUT0123	1.0920244	0.0121117	0.040793	1.34	2.85	12.00	36.25
SUT	SUT257	1.0924591	0.0129631	0.0432562	2.23	4.82	22.00	66.50
ORF-T	YOR277C	1.0942324	0.0003237	0.0017155	28.13	60.06	250.50	754.75
CUT	CUT203	1.0949899	0.0276655	0.0802817	2.01	4.27	18.25	58.25

TABLE S1: Differential expression data for RRP6 RNA-Seq dataset Page 263

Class	Transcript name	RRP6 KO_vs_WT log2_fold _change	RRP6 KO_vs_WT p-value	RRP6 KO_vs_WT FDR	Ave Norm Reads in WT	Ave Norm Reads in RRP6	Average RAW read counts in WT	Average RAW read counts in RRP6
CUT	CUT763	1.0951192	0.0086213	0.030515	0.79	1.70	7.25	21.75
CUT	CUT908	1.0957831	0.0864981	0.1928044	0.59	1.29	6.00	18.00
CUT	CUT488	1.0970937	0.006937	0.0255957	1.32	2.90	12.75	37.00
NUT	NUT0855	1.09848	0.0070676	0.0259728	2.05	4.30	17.25	55.00
AST	AS_YNL295W	1.0986114	0.0170358	0.0541237	0.78	1.71	7.50	23.00
CUT	CUT284	1.0988181	0.0216323	0.0654487	1.59	3.45	15.25	46.25
ORF-T	YNL097W-A	1.0989736	0.0683659	0.1620215	0.31	0.72	3.00	8.75
NUT	NUT0236	1.1010031	0.0676304	0.1608075	0.43	0.83	3.50	12.00
NUT	NUT0748	1.101238	0.0033382	0.0136781	3.12	6.81	30.25	86.25
NUT	NUT0118	1.1012425	0.0002371	0.0013103	10.60	22.79	97.25	292.25
ORF-T	YOR186W	1.1033379	0.0223271	0.0671578	0.55	1.24	5.25	15.50
CUT	CUT636	1.1034058	0.0108051	0.0371364	0.99	2.16	9.25	28.00
SUT	SUT585	1.1037248	0.0182821	0.0572005	1.08	2.33	10.25	32.75
AST	AS_YMR276W	1.1059606	0.013735	0.0452677	1.31	2.76	11.50	37.50
NUT	NUT0006	1.1064126	0.0670386	0.1597771	0.62	1.32	5.50	18.00
ORF-T	YFL010W-A	1.1083385	0.0590642	0.1453205	0.40	0.92	4.00	12.00
CUT	CUT523	1.1088827	0.0589372	0.1451692	0.45	1.07	5.00	14.25
CUT	CUT302	1.1095203	0.0836192	0.1883207	0.54	1.19	5.00	14.75
SUT	SUT575	1.1111999	0.0028149	0.0117682	4.45	9.68	42.50	129.25
NUT	NUT0579	1.1119228	0.0654154	0.1567979	0.63	1.39	6.00	19.00
NUT	NUT0833	1.1119664	0.0064326	0.0240514	1.35	2.99	12.75	36.75
NUT	NUT0995	1.1127877	2.40E-05	0.0001735	9.37	20.20	83.75	262.75
ORF-T	YNL057W	1.1134393	0.0499878	0.1278651	1.04	2.32	10.25	28.25
NUT	NUT0267	1.1135927	0.0048589	0.0189546	1.46	3.06	12.50	40.75
NUT	NUT1225	1.1152806	0.0271301	0.0789325	1.08	2.43	11.50	34.50
SRT	SRT523	1.11534	0.0006361	0.0031199	2.58	5.57	23.25	71.50
SRT	SRT309	1.115485	0.042953	0.1138638	0.81	1.82	7.75	21.50
SUT	SUT743	1.1179741	8.83E-05	0.0005471	17.37	37.71	162.50	519.50
SUT	SUT205	1.1193825	0.0005586	0.0027832	4.29	9.35	39.25	119.50

Class	Transcript name	RRP6 KO_vs_WT log2_fold _change	RRP6 KO_vs_WT p-value	RRP6 KO_vs_WT FDR	Ave Norm Reads in WT	Ave Norm Reads in RRP6	Average RAW read counts in WT	Average RAW read counts in RRP6
AST	AS_YBR216C	1.1194113	0.0226895	0.0679943	0.54	1.13	4.75	15.25
sn/snoRNA ET	SNR73-ET	1.1209287	0.0019492	0.008557	40.89	88.89	351.50	1092.75
CUT	CUT298	1.1214204	0.0147753	0.0480008	1.08	2.36	9.75	30.75
AST	AS_YMR296C	1.122431	0.0193305	0.0598677	3.66	8.02	35.25	109.00
NUT	NUT0588	1.1228451	0.0153492	0.0496252	0.78	1.67	6.75	20.75
CUT	CUT777	1.1229749	0.0005874	0.0029092	2.56	5.55	23.00	71.25
AST	AS_YDR381C-A	1.1234358	0.0219381	0.0662149	0.70	1.51	6.50	21.25
NUT	NUT0260	1.1235108	0.0030022	0.0124635	1.61	3.55	15.25	47.25
SUT	SUT782	1.1240194	0.0141684	0.0464329	1.27	2.71	10.75	33.50
SUT	SUT623	1.124224	0.0127001	0.0425052	1.02	2.28	9.50	28.25
NUT	NUT0847	1.1245882	0.0444396	0.1169439	0.56	1.19	5.00	16.25
ORF-T	YMR182W-A	1.1246081	0.0011409	0.0052796	3.04	6.69	28.75	89.25
ORF-T	YLR235C	1.1250334	0.0335516	0.0937993	0.56	1.23	5.25	16.75
SRT	SRT569	1.1253266	0.0229699	0.0686308	0.51	1.09	4.50	14.00
AST	AS_YLR353W	1.1272731	0.0337637	0.0942787	0.70	1.49	6.25	20.50
NUT	NUT1309	1.1277202	0.0005532	0.0027588	2.56	5.57	23.00	71.50
SUT	SUT424	1.1278281	0.0403977	0.1088062	0.72	1.49	5.75	19.25
NUT	NUT0145	1.1279305	0.0257075	0.0754465	0.81	1.74	7.25	23.50
NUT	NUT0002	1.1279318	0.0099137	0.0343777	1.16	2.53	10.50	33.25
sn/snoRNA ET	SNR67-ET*	1.1286849	0.0052505	0.0202469	65.69	143.64	577.50	1776.50
NUT	NUT0726	1.1290137	0.0211842	0.0644214	0.90	1.97	8.25	26.25
SUT	SUT255	1.1296663	1.82E-05	0.0001364	15.68	34.38	145.75	443.00
NUT	NUT1227	1.1305173	0.0051	0.0197726	1.92	4.25	18.00	55.50
NUT	NUT1336	1.131014	7.48E-05	0.0004742	17.52	38.36	163.75	528.25
NUT	NUT1406	1.1319521	0.0053698	0.0206284	1.30	2.94	12.75	38.75
SUT	SUT534	1.1321928	0.0027225	0.0114202	2.96	6.57	28.00	83.50
NUT	NUT0188	1.1325658	0.0195364	0.0603386	0.76	1.70	7.25	22.25
NUT	NUT0910	1.1354939	0.0079668	0.02861	1.77	3.90	16.25	50.75
NUT	NUT0154	1.1371632	0.0072314	0.0264785	0.98	2.13	8.50	27.00

Class	Transcript name	RRP6 KO_vs_WT log2_fold _change	RRP6 KO_vs_WT p-value	RRP6 KO_vs_WT FDR	Ave Norm Reads in WT	Ave Norm Reads in RRP6	Average RAW read counts in WT	Average RAW read counts in RRP6
SUT	SUT261	1.1385259	0.0001111	0.0006727	4.42	9.76	41.25	130.00
SUT	SUT627	1.1395662	0.0004425	0.0022614	4.54	10.06	43.00	136.50
NUT	NUT1279	1.1398905	0.0004103	0.0021209	8.01	17.82	79.00	237.25
SUT	SUT416	1.1402828	0.0212261	0.0645294	0.54	1.10	4.50	15.25
NUT	NUT0451	1.1405457	4.64E-06	4.14E-05	17.74	39.16	163.00	509.75
CUT	CUT299	1.1409018	0.036776	0.1009663	0.56	1.20	4.75	15.50
NUT	NUT0875	1.1410026	0.001583	0.0071104	4.00	8.75	34.50	112.75
AST	AS_YMR226C	1.1416708	0.0084383	0.0300043	0.93	2.02	8.25	26.75
SUT	SUT073	1.1419309	0.0020342	0.008884	1.72	3.88	16.50	50.00
NUT	NUT0064	1.1432213	0.0024785	0.0105102	3.93	8.67	35.25	112.00
CUT	CUT755	1.1440816	0.0230027	0.0687084	0.47	0.98	4.00	13.00
NUT	NUT0581	1.1442819	0.0122113	0.0411148	1.08	2.39	9.75	31.25
ORF-T	YHR157W	1.1443505	0.0006079	0.0029963	3.80	8.40	34.25	104.75
NUT	NUT0515	1.1449515	1.42E-05	0.0001094	20.91	46.41	203.25	621.25
CUT	CUT132	1.1465631	0.0071704	0.0262741	1.17	2.65	11.00	32.50
NUT	NUT0065	1.1485723	0.0006443	0.0031524	2.19	4.93	20.75	64.75
NUT	NUT0452	1.1486648	0.0077876	0.0281267	1.12	2.45	9.75	31.00
ORF-T	YLR297W	1.1495985	0.0044173	0.017475	7.09	15.71	64.50	209.25
NUT	NUT1426	1.1499312	0.0102831	0.035512	1.49	3.27	13.00	40.25
NUT	NUT0143	1.1500318	0.0072904	0.0266559	1.75	3.99	17.25	52.25
NUT	NUT1043	1.1509733	0.0086386	0.0305549	0.90	1.98	8.00	25.75
NUT	NUT0495	1.151375	0.0094853	0.0331311	1.30	2.87	11.75	38.75
SRT	SRT336	1.1519024	0.0392442	0.1065533	0.56	1.22	5.00	16.50
CUT	CUT202	1.1539497	0.0168284	0.0536	1.68	3.84	17.00	49.50
ORF-T	YER181C	1.1542236	0.0115031	0.0391617	1.10	2.38	9.25	30.50
NUT	NUT1005	1.1552176	0.0007779	0.0037534	4.03	9.02	37.25	115.00
ORF-T	YIL012W	1.156135	0.010096	0.0349375	0.89	2.03	8.50	26.50
NUT	NUT0403	1.1565606	0.0019175	0.0084513	2.27	5.09	21.00	67.00
NUT	NUT0815	1.1576983	0.0036839	0.0149072	1.19	2.57	10.25	34.25

Class	Transcript name	RRP6 KO_vs_WT log2_fold _change	RRP6 KO_vs_WT p-value	RRP6 KO_vs_WT FDR	Ave Norm Reads in WT	Ave Norm Reads in RRP6	Average RAW read counts in WT	Average RAW read counts in RRP6
NUT	NUT0193	1.1603057	0.003516	0.0143196	3.27	7.31	30.25	99.00
AST	AS_YJL030W	1.1605872	0.0753328	0.1743931	0.34	0.78	3.25	10.50
NUT	NUT1372	1.1611483	0.0051861	0.0200447	1.22	2.81	11.75	36.00
AST	AS_YAL021C	1.1614284	0.0241345	0.0715154	1.61	3.56	14.25	50.75
SUT	SUT122	1.1633072	0.0022276	0.0095957	3.11	6.89	26.75	88.00
SUT	SUT057	1.1636302	0.0005085	0.0025718	3.00	6.68	27.00	87.75
NUT	NUT0888	1.1639849	0.0039165	0.0157533	1.03	2.28	9.25	30.25
AST	AS_YLR412W	1.1643035	0.0022031	0.0095064	2.34	5.27	22.00	70.50
AST	AS_YJL045W	1.1647695	0.0029714	0.0123563	1.62	3.63	14.50	46.00
ORF-T	YJR020W	1.1652074	0.0049715	0.0193489	1.63	3.75	16.25	49.75
SUT	SUT396	1.1662626	0.0225122	0.0675634	0.73	1.64	6.50	20.50
SRT	SRT297	1.1662732	0.0231763	0.0691654	0.73	1.64	6.50	20.50
CUT	CUT431	1.1671109	0.0094327	0.0329818	1.13	2.56	10.25	32.50
NUT	NUT1034	1.1671579	7.11E-05	0.000453	10.00	22.47	91.00	285.25
AST	AS_YMR009W	1.1676179	0.005055	0.0196282	0.79	1.78	7.25	23.00
SUT	SUT236	1.1694322	0.0287296	0.0827976	1.44	3.19	12.25	39.75
NUT	NUT0128	1.1700967	6.60E-05	0.0004254	9.50	21.52	90.50	276.75
ORF-T	YBR040W	1.171895	0.0603527	0.1476818	0.53	1.17	4.50	14.50
CUT	CUT066	1.173791	0.0070011	0.0257757	1.11	2.48	9.75	31.50
CUT	CUT732	1.174154	0.0220003	0.0663629	0.81	1.89	7.75	23.75
SUT	SUT388	1.1753989	0.0002497	0.0013692	8.10	18.26	73.50	241.75
ORF-T	YKL030W	1.1763083	0.0158646	0.0510136	0.48	1.16	4.75	14.75
AST	AS_YDR310C	1.1776147	0.0083577	0.0297596	1.15	2.53	10.00	34.75
NUT	NUT0775	1.1780659	0.0153966	0.0497612	1.65	3.73	15.75	54.25
AST	AS_YDR034C-A	1.178927	0.0025921	0.0109505	1.99	4.44	17.25	56.25
NUT	NUT1385	1.1811853	0.0312895	0.088705	0.66	1.55	6.50	20.75
SUT	SUT221	1.1815513	2.98E-06	2.83E-05	20.84	47.40	197.75	624.25
sn/snoRNA ET	SNR33-ET	1.1821705	2.36E-05	0.0001711	8.12	18.38	73.75	243.50
SUT	SUT767	1.1824439	0.0314625	0.0891453	0.85	1.93	7.75	24.75

TABLE S1: Differential expression data for RRP6 RNA-Seq dataset Page 267

Class	Transcript name	RRP6 KO_vs_WT log2_fold _change	RRP6 KO_vs_WT p-value	RRP6 KO_vs_WT FDR	Ave Norm Reads in WT	Ave Norm Reads in RRP6	Average RAW read counts in WT	Average RAW read counts in RRP6
SUT	SUT552	1.1825716	0.0167262	0.0533419	1.12	2.59	10.75	35.50
CUT	CUT191	1.1827946	0.0080717	0.0289149	1.00	2.24	9.00	30.50
NUT	NUT0465	1.1828011	0.0207237	0.0632118	1.54	3.44	13.00	43.00
SRT	SRT315	1.1843424	0.0311443	0.0884176	0.72	1.55	5.75	20.00
SRT	SRT32	1.1847216	0.0364068	0.1000707	0.41	0.94	3.75	12.00
NUT	NUT0762	1.1850897	0.032983	0.0926723	0.60	1.34	5.25	18.00
CUT	CUT784	1.1855456	0.0009609	0.0045362	3.96	8.94	35.25	118.50
ORF-T	YER038W-A	1.1866776	0.0844532	0.1897303	0.37	0.79	3.00	10.50
CUT	CUT464	1.1869583	0.0041827	0.0166286	1.16	2.69	11.00	35.50
CUT	CUT088	1.1869592	0.0120649	0.0406627	1.01	2.31	9.25	30.00
NUT	NUT0141	1.1869898	0.0135351	0.0447356	1.69	3.90	16.25	50.25
SRT	SRT124	1.1872603	0.0427791	0.1134734	0.36	0.77	3.00	10.25
ORF-T	YGR069W	1.1875841	0.0176479	0.0557175	0.40	0.94	3.75	12.00
NUT	NUT1235	1.1878801	0.0001052	0.0006394	9.65	22.04	89.00	281.75
NUT	NUT0840	1.1879107	0.0069594	0.0256593	0.88	1.95	7.75	26.25
NUT	NUT1349	1.188228	0.0296817	0.085011	0.69	1.53	6.25	22.00
CUT	CUT664	1.1894261	0.0228691	0.0684309	0.76	1.74	7.00	22.75
NUT	NUT0935	1.189739	0.0072751	0.0266093	1.30	2.90	11.00	36.75
CUT	CUT014	1.1906733	0.0191067	0.0592471	0.90	2.01	8.00	28.00
AST	AS_YGR201C	1.190845	0.0027043	0.0113626	2.10	4.80	19.25	64.00
NUT	NUT1205	1.1916134	0.0116002	0.0394259	1.10	2.48	9.75	32.25
NUT	NUT0437	1.1918132	2.41E-06	2.36E-05	20.87	47.82	198.00	629.25
SUT	SUT451	1.1921606	0.0030851	0.0127448	1.39	3.11	12.00	39.75
NUT	NUT0923	1.1935015	0.0011233	0.0052057	2.16	4.86	18.75	64.50
sn/snoRNA ET	SNR76-ET	1.1938006	0.0009317	0.0044254	17.79	40.69	155.25	488.50
sn/snoRNA ET	SNR14-ET	1.1965005	0.0015735	0.0070836	20.07	46.06	182.00	567.25
SUT	SUT668	1.1968134	0.0386404	0.1051971	0.61	1.32	4.75	16.50
CUT	CUT651	1.1970527	0.007102	0.0260614	0.78	1.69	6.50	22.50
ORF-T	YLR120W-A	1.1987842	0.0035468	0.0144218	0.81	1.90	7.75	25.00

TABLE S1: Differential expression data for RRP6 RNA-Seq dataset Page 268

Class	Transcript name	RRP6 KO_vs_WT log2_fold_change	RRP6 KO_vs_WT p-value	RRP6 KO_vs_WT FDR	Ave Norm Reads in WT	Ave Norm Reads in RRP6	Average RAW read counts in WT	Average RAW read counts in RRP6
NUT	NUT0139	1.1993055	7.93E-05	0.0004983	6.42	14.69	57.00	189.25
AST	AS_YKR072C	1.2000373	0.0836275	0.1883207	0.43	0.94	3.50	12.25
NUT	NUT0656	1.2013542	0.010913	0.037431	0.88	2.05	8.00	25.25
ORF-T	YDR223W	1.2022685	0.0034273	0.014015	5.33	12.33	50.00	156.00
ORF-T	YOR237W	1.2031796	0.0048654	0.0189725	4.99	11.60	49.00	151.50
AST	AS_YDL138W	1.2064686	3.22E-05	0.0002259	5.19	12.06	48.50	155.25
CUT	CUT778	1.2071323	0.0218061	0.0658954	0.62	1.42	5.50	18.75
NUT	NUT1519	1.2071516	0.03927	0.106578	0.64	1.47	5.50	18.25
SUT	SUT154	1.2091584	0.0156771	0.0505073	1.04	2.32	8.75	30.75
NUT	NUT0347	1.2103049	6.12E-05	0.0003984	5.31	12.30	49.00	161.25
NUT	NUT0647	1.2112458	4.22E-05	0.0002857	5.49	12.83	52.25	164.75
SUT	SUT558	1.212589	0.0010671	0.0049747	3.21	7.41	28.50	95.75
CUT	CUT330	1.2126358	0.018122	0.0568405	0.53	1.17	4.50	15.50
ORF-T	YDR124W	1.2131697	4.42E-05	0.0002987	8.59	20.06	82.00	258.75
NUT	NUT0114	1.2132947	0.0022041	0.0095065	1.08	2.54	10.25	33.25
SRT	SRT566	1.2133102	0.0418912	0.111814	0.80	1.87	7.25	23.75
ORF-T	YLR162W-A	1.2134589	0.009771	0.0339994	1.54	3.48	13.25	48.75
NUT	NUT1038	1.2139918	0.0040799	0.0162936	1.18	2.77	11.25	37.75
SUT	SUT634	1.2141486	0.0018377	0.0081261	2.45	5.64	21.50	72.75
SUT	SUT632	1.2144458	0.000863	0.0041203	12.35	28.73	113.75	358.75
AST	AS_YGL234W	1.2147256	0.0297077	0.0850334	0.62	1.46	6.00	19.75
NUT	NUT1483	1.2166236	0.0009464	0.0044784	3.71	8.65	33.75	110.25
SUT	SUT633	1.2179744	0.0011594	0.0053532	1.98	4.67	18.50	59.25
NUT	NUT0381	1.2209457	0.005383	0.0206712	1.14	2.71	11.00	36.25
SRT	SRT252	1.2211139	0.0152153	0.0493118	0.49	1.11	4.25	14.75
SUT	SUT030	1.2214281	0.0295906	0.084891	0.45	1.02	4.00	14.00
NUT	NUT1280	1.2231881	0.0036092	0.0146461	1.27	2.88	11.00	39.00
NUT	NUT1049	1.2233886	0.0231959	0.0692033	0.40	0.97	4.00	13.50
NUT	NUT0858	1.2237303	0.0593626	0.1457542	0.63	1.45	5.75	20.25

TABLE S1: Differential expression data for RRP6 RNA-Seq dataset Page 269

Class	Transcript name	RRP6 KO_vs_WT log2_fold_change	RRP6 KO_vs_WT p-value	RRP6 KO_vs_WT FDR	Ave Norm Reads in WT	Ave Norm Reads in RRP6	Average RAW read counts in WT	Average RAW read counts in RRP6
SUT	SUT693	1.2239291	5.97E-05	0.0003905	3.29	7.74	30.50	99.75
NUT	NUT1390	1.2256357	0.003205	0.013197	1.95	4.56	17.50	58.50
NUT	NUT0462	1.2265464	0.0116319	0.0394936	0.54	1.23	4.75	16.25
NUT	NUT0889	1.2270644	0.0119182	0.0402758	0.71	1.61	6.25	22.25
NUT	NUT1229	1.2279637	0.0435361	0.1151976	0.69	1.64	6.75	23.00
CUT	CUT863	1.2280407	0.0103828	0.0357948	1.06	2.50	10.00	33.50
NUT	NUT1067	1.2285164	0.0007993	0.0038475	1.86	4.27	16.25	57.50
NUT	NUT1008	1.229577	0.027805	0.0805603	0.62	1.47	6.00	20.00
NUT	NUT0175	1.2335745	0.0131874	0.0438307	0.91	2.19	8.50	27.25
AST	AS_YDL008W	1.2339706	0.0106791	0.0367537	1.56	3.60	13.50	49.25
SUT	SUT137	1.2341471	0.001172	0.0054015	2.35	5.59	22.25	74.50
SRT	SRT508	1.2342202	0.01944	0.060102	0.68	1.56	6.00	21.50
CUT	CUT084	1.2343704	0.0006647	0.0032444	2.50	6.02	24.75	78.75
NUT	NUT0691	1.2355783	0.0297358	0.0850896	0.41	0.92	3.50	12.25
NUT	NUT0637	1.2363927	0.0050045	0.0194621	0.89	2.03	7.50	25.75
NUT	NUT1108	1.2367983	0.0178931	0.0563505	0.65	1.56	6.25	21.50
CUT	CUT506	1.2371822	0.0190382	0.0591256	0.74	1.68	6.50	24.00
NUT	NUT1386	1.2374653	4.63E-05	0.0003117	5.98	14.15	55.00	182.50
SUT	SUT671	1.2384034	0.0021357	0.009243	2.48	5.85	21.75	72.50
NUT	NUT0503	1.2388774	3.33E-06	3.11E-05	13.88	32.88	130.00	423.75
CUT	CUT904	1.2395456	0.022969	0.0686308	1.86	4.44	17.00	52.75
NUT	NUT1328	1.2396961	0.0001921	0.0010831	4.19	9.96	38.75	125.75
NUT	NUT1283	1.239913	0.0004634	0.0023567	2.26	5.36	20.75	68.00
SUT	SUT815	1.240088	0.0002861	0.0015388	6.02	14.33	57.00	183.25
NUT	NUT0371	1.240096	0.0002873	0.0015437	5.14	12.22	48.25	157.00
SUT	SUT002	1.2414956	0.0095101	0.0332064	8.36	19.77	75.75	242.00
CUT	CUT875	1.2419549	0.0015511	0.0070075	7.51	17.85	68.75	213.00
SRT	SRT575	1.2419553	0.0015517	0.0070075	7.51	17.85	68.75	213.00
NUT	NUT1484	1.2420606	0.0002321	0.0012868	6.31	15.02	59.25	192.25

TABLE S1: Differential expression data for RRP6 RNA-Seq dataset Page 270

Class	Transcript name	RRP6 KO_vs_WT log2_fold_change	RRP6 KO_vs_WT p-value	RRP6 KO_vs_WT FDR	Ave Norm Reads in WT	Ave Norm Reads in RRP6	Average RAW read counts in WT	Average RAW read counts in RRP6
SUT	SUT703	1.2423878	6.16E-05	0.0003998	4.98	11.74	44.75	154.75
CUT	CUT126	1.2456299	0.0219012	0.0661232	0.56	1.24	4.50	16.50
SUT	SUT010	1.2467263	0.002669	0.0112328	2.20	5.15	19.00	67.50
ORF-T	YMR118C	1.249581	0.0080263	0.0288134	1.04	2.53	10.00	32.75
SUT	SUT464	1.2498332	0.0080819	0.0289383	1.68	3.92	14.50	53.75
NUT	NUT1440	1.2520525	0.0182485	0.0571306	0.47	1.17	4.75	16.00
NUT	NUT0355	1.2542596	0.0071219	0.0261247	1.95	4.66	17.75	59.75
SUT	SUT203	1.2543795	0.0057944	0.0220456	3.25	7.68	27.50	96.50
AST	AS_YKL157W	1.2549967	0.0005503	0.0027471	3.83	9.17	35.75	123.00
NUT	NUT1040	1.2561796	0.0022754	0.0097803	1.31	3.18	12.75	44.00
NUT	NUT1211	1.2572561	0.0005465	0.0027349	3.75	9.00	35.00	121.00
NUT	NUT0384	1.2581443	0.0005968	0.0029474	2.77	6.63	25.50	88.25
NUT	NUT1096	1.2585502	0.0017344	0.0077253	1.24	3.02	12.00	40.75
NUT	NUT0346	1.2587335	0.0002352	0.001301	4.25	10.24	39.50	129.75
NUT	NUT1379	1.2600471	0.0037484	0.0151559	1.61	3.85	14.25	48.50
SUT	SUT199	1.2607146	0.0102641	0.0354584	0.57	1.39	5.25	17.50
CUT	CUT206	1.2610127	0.0022938	0.0098512	1.05	2.52	9.75	34.00
NUT	NUT1167	1.2618543	2.97E-05	0.0002097	5.37	12.88	48.50	162.75
CUT	CUT873	1.2621105	0.0056192	0.0214717	1.02	2.47	9.25	30.25
CUT	CUT672	1.2625189	0.0005044	0.0025521	1.86	4.57	18.00	59.75
NUT	NUT1195	1.2639757	0.0072457	0.0265115	1.65	4.00	15.25	50.25
ORF-T	YLR169W	1.2641637	0.0153192	0.0495657	1.04	2.54	10.25	35.00
CUT	CUT100	1.2662686	0.0149887	0.0486601	0.72	1.79	6.75	22.00
SRT	SRT140	1.266974	0.0054143	0.0207833	3.22	7.68	27.25	96.50
NUT	NUT1014	1.2680922	0.0001951	0.0010985	2.65	6.48	25.25	82.50
NUT	NUT1264	1.2689067	4.04E-05	0.0002756	5.05	12.11	45.25	159.50
ORF-T	YPR192W	1.270017	0.0113788	0.0388303	1.92	4.64	16.75	53.25
NUT	NUT1293	1.2704097	0.0041496	0.0165199	0.68	1.73	6.75	22.25
SUT	SUT237	1.2704186	0.001225	0.0056251	3.86	9.35	37.00	129.00

TABLE S1: Differential expression data for RRP6 RNA-Seq dataset Page 271

Class	Transcript name	RRP6 KO_vs_WT log2_fold _change	RRP6 KO_vs_WT p-value	RRP6 KO_vs_WT FDR	Ave Norm Reads in WT	Ave Norm Reads in RRP6	Average RAW read counts in WT	Average RAW read counts in RRP6
SUT	SUT215	1.2730214	0.0068207	0.0252402	0.79	1.98	7.50	23.75
SRT	SRT407	1.2751486	0.0235704	0.0700719	0.45	1.08	4.00	13.75
ORF-T	YCL026C-A	1.275414	0.0491969	0.1263953	0.63	1.51	5.75	21.00
NUT	NUT0256	1.2762976	0.0001501	0.0008815	5.22	12.74	49.50	163.75
SUT	SUT325	1.2771419	1.01E-05	8.11E-05	6.10	14.78	54.75	188.75
AST	AS_YDR395W	1.2783282	0.0456044	0.1194542	0.94	2.25	8.25	30.50
NUT	NUT1275	1.2794585	0.0021141	0.0091656	0.85	2.06	7.75	26.50
NUT	NUT0107	1.2812969	0.0050772	0.0197067	1.56	3.79	14.75	52.75
NUT	NUT0509	1.2822864	3.41E-05	0.0002374	3.67	9.02	35.25	120.00
NUT	NUT0004	1.284166	0.0060275	0.0227817	8.53	20.77	77.50	256.75
SUT	SUT709	1.2842436	0.0001659	0.0009588	7.26	17.79	68.50	224.50
CUT	CUT590	1.2843867	0.0011372	0.0052654	1.09	2.65	10.00	35.25
NUT	NUT1370	1.2846399	3.64E-05	0.0002511	8.24	20.14	78.25	271.50
SUT	SUT809	1.2851351	0.0074531	0.0271424	1.09	2.70	10.50	35.25
NUT	NUT1159	1.2857754	0.0003858	0.0020065	1.84	4.58	17.75	60.00
CUT	CUT405	1.2863493	0.0143902	0.0470224	0.59	1.40	5.00	17.25
NUT	NUT0005	1.2867242	6.37E-06	5.45E-05	7.30	17.96	70.25	237.75
NUT	NUT1216	1.2872947	0.0091011	0.0320221	3.29	8.02	29.50	108.75
CUT	CUT502	1.2876568	0.0293058	0.0842416	0.44	1.06	3.75	13.00
SUT	SUT005	1.2877782	0.0032589	0.013397	0.96	2.21	7.75	28.75
NUT	NUT0120	1.2884128	1.21E-06	1.29E-05	9.85	24.10	90.75	315.50
NUT	NUT1012	1.2891293	0.0011173	0.00519	1.59	3.85	14.25	51.50
NUT	NUT0131	1.2901977	0.0115879	0.0394085	0.73	1.82	6.75	23.00
CUT	CUT069	1.2901991	0.0115912	0.0394085	0.73	1.82	6.75	23.00
NUT	NUT1276	1.2904219	7.61E-05	0.0004814	4.43	10.85	40.75	143.50
CUT	CUT516	1.2909296	0.0006283	0.0030845	1.71	4.12	15.00	54.00
NUT	NUT1170	1.2914648	0.002857	0.011915	0.69	1.72	6.50	22.75
CUT	CUT389	1.2914659	0.0012661	0.0057979	1.41	3.41	12.50	44.75
NUT	NUT1322	1.2923861	0.0004515	0.0023018	1.56	3.84	14.25	49.75

Class	Transcript name	RRP6 KO_vs_WT log2_fold _change	RRP6 KO_vs_WT p-value	RRP6 KO_vs_WT FDR	Ave Norm Reads in WT	Ave Norm Reads in RRP6	Average RAW read counts in WT	Average RAW read counts in RRP6
AST	AS_YDR079W	1.2938099	0.0016643	0.0074459	1.03	2.61	10.00	33.50
ORF-T	YDR222W	1.2941199	0.0004156	0.0021447	11.36	27.88	106.25	375.75
NUT	NUT0845	1.2941364	0.0035347	0.0143858	1.23	3.00	11.00	38.75
NUT	NUT1109	1.2954532	0.005086	0.0197283	1.09	2.61	9.25	33.75
CUT	CUT806	1.2976953	0.0004812	0.0024408	2.11	5.19	19.50	69.75
NUT	NUT0280	1.2981349	0.001506	0.0068286	0.75	1.94	7.50	25.75
NUT	NUT1158	1.2982964	0.0227392	0.0680827	0.76	1.85	7.00	27.00
NUT	NUT0520	1.300159	0.0023284	0.0099787	1.05	2.59	9.50	33.50
NUT	NUT0767	1.3006361	0.0051228	0.0198534	0.73	1.75	6.25	22.25
NUT	NUT1019	1.3010635	0.0009366	0.0044445	1.09	2.67	10.00	35.75
NUT	NUT1145	1.3021028	0.0001196	0.0007174	5.14	12.71	46.75	161.25
CUT	CUT743	1.3025424	0.0194419	0.060102	0.62	1.60	6.00	19.75
NUT	NUT0469	1.3031068	7.94E-05	0.0004983	5.10	12.60	46.25	161.25
SUT	SUT386	1.3032513	0.0024233	0.0103195	4.19	10.35	38.00	134.00
NUT	NUT1330	1.3032865	0.0065277	0.0243525	0.58	1.45	5.50	19.75
SUT	SUT251	1.3033373	1.92E-05	0.0001423	4.60	11.50	44.75	152.25
NUT	NUT1133	1.3038931	1.27E-05	9.91E-05	5.13	12.79	48.50	164.50
NUT	NUT1404	1.3040453	0.0030534	0.012642	1.11	2.75	10.25	36.50
NUT	NUT0591	1.3040728	0.0114651	0.0390457	1.14	2.76	9.50	34.75
NUT	NUT0839	1.3045163	0.0066108	0.0246042	1.24	3.05	11.25	39.50
SUT	SUT784	1.3073301	0.0061016	0.0229929	0.85	2.15	8.00	27.00
NUT	NUT0610	1.3080869	0.0023888	0.0101985	0.72	1.76	6.50	23.50
NUT	NUT0764	1.3081292	0.0078552	0.0283203	0.44	1.09	4.00	14.25
AST	AS_YJL056C	1.3082765	0.0070821	0.0260072	0.69	1.85	7.25	23.25
SUT	SUT167	1.3106435	0.0001717	0.0009838	3.74	9.36	34.75	117.50
SUT	SUT293	1.3114718	0.0051297	0.0198648	0.83	2.06	7.50	26.50
NUT	NUT0549	1.3121011	0.0181978	0.0570426	0.72	1.77	6.50	23.75
ORF-T	YJL191W	1.3124904	2.56E-06	2.48E-05	67.43	167.48	614.50	2226.50
SUT	SUT433	1.3130139	0.0009746	0.0045901	2.15	5.47	21.50	71.75

Class	Transcript name	RRP6 KO_vs_WT log2_fold _change	RRP6 KO_vs_WT p-value	RRP6 KO_vs_WT FDR	Ave Norm Reads in WT	Ave Norm Reads in RRP6	Average RAW read counts in WT	Average RAW read counts in RRP6
NUT	NUT1442	1.3137753	0.0016848	0.007531	0.95	2.37	8.75	31.25
NUT	NUT0812	1.3181149	0.0057919	0.0220456	0.73	1.79	6.50	23.50
NUT	NUT0329	1.3181528	0.0065513	0.0244188	1.18	2.87	10.00	37.50
AST	AS_YJL047C	1.3203905	0.0062208	0.0233894	0.55	1.35	5.00	18.25
NUT	NUT0379	1.3204598	2.43E-06	2.38E-05	67.88	169.53	619.00	2254.00
ORF-T	YJL027C	1.3206671	0.0136209	0.0449897	0.75	1.88	7.00	25.50
sn/snoRNA ET	SNR39-ET	1.3212759	6.13E-05	0.0003989	7.34	18.30	64.25	229.25
SUT	SUT114	1.3217378	8.99E-05	0.0005555	5.06	12.73	47.75	163.50
SUT	SUT018	1.3220734	5.17E-05	0.0003435	3.11	7.76	28.50	102.75
AST	AS_YLR091W	1.322277	4.10E-05	0.0002793	2.78	7.05	26.25	90.25
NUT	NUT0166	1.3224106	0.0020663	0.0090122	0.88	2.21	8.00	28.25
CUT	CUT097	1.3224175	0.0020756	0.0090413	0.88	2.21	8.00	28.25
ORF-T	YGR138C	1.3230184	0.0002706	0.0014677	13.80	34.46	121.75	428.25
CUT	CUT859	1.3236263	0.014597	0.0475749	0.43	1.07	4.00	15.00
NUT	NUT1290	1.3239129	0.0058612	0.0222197	0.59	1.51	5.50	19.25
NUT	NUT0494	1.3245992	0.0309033	0.087886	0.41	1.12	4.50	15.50
AST	AS_YBR069C	1.3249152	7.74E-06	6.46E-05	7.69	19.36	72.75	256.75
CUT	CUT697	1.3249607	0.0037515	0.0151596	0.99	2.46	8.75	32.25
NUT	NUT0096	1.3250734	2.31E-05	0.0001683	3.58	9.04	33.25	114.75
NUT	NUT1505	1.3256352	3.50E-05	0.0002423	2.51	6.41	24.00	83.00
NUT	NUT1110	1.3273835	0.0020753	0.0090413	2.78	7.05	26.00	87.75
NUT	NUT1122	1.3284632	0.0070861	0.0260124	1.53	3.86	14.50	52.75
NUT	NUT0441	1.3289105	0.0494348	0.1267159	0.37	0.88	3.00	11.00
NUT	NUT0041	1.3307245	4.17E-05	0.0002833	3.11	7.81	28.50	103.25
SUT	SUT803	1.3320259	0.0054658	0.0209572	1.14	2.70	9.00	37.00
SUT	SUT580	1.3335069	0.0004192	0.0021613	5.33	13.45	47.75	170.50
NUT	NUT0642	1.3335638	2.93E-06	2.79E-05	6.82	17.17	61.25	219.50
NUT	NUT0473	1.3337689	0.0402939	0.1085845	0.50	1.24	4.25	15.75
NUT	NUT1100	1.3354644	0.000439	0.002246	6.27	15.85	56.25	200.25

Class	Transcript name	RRP6 KO_vs_WT log2_fold _change	RRP6 KO_vs_WT p-value	RRP6 KO_vs_WT FDR	Ave Norm Reads in WT	Ave Norm Reads in RRP6	Average RAW read counts in WT	Average RAW read counts in RRP6
CUT	CUT767	1.3357034	0.000109	0.0006615	2.59	6.57	23.75	85.25
NUT	NUT0474	1.337661	0.0021648	0.009361	1.73	4.42	16.00	56.00
CUT	CUT050	1.3385352	0.0009615	0.0045373	1.74	4.51	17.25	59.25
CUT	CUT285	1.3391444	0.0053598	0.0205975	0.70	1.77	6.25	22.25
NUT	NUT1188	1.3407605	0.0020876	0.0090809	0.93	2.37	8.50	30.50
SUT	SUT778	1.3422737	0.0051552	0.0199484	0.54	1.42	5.25	18.75
SRT	SRT73	1.3423443	0.0076332	0.0276981	0.72	1.83	6.75	24.25
SUT	SUT591	1.3427422	0.0020569	0.0089754	2.68	6.87	25.00	85.00
NUT	NUT1413	1.3431994	0.0062478	0.0234561	1.02	2.44	8.00	31.75
NUT	NUT0194	1.3434363	0.0002777	0.0014998	1.94	4.97	18.25	64.25
NUT	NUT1523	1.3439567	8.76E-05	0.0005436	3.54	9.09	34.00	120.50
NUT	NUT1366	1.3443269	7.98E-06	6.63E-05	4.15	10.57	38.25	136.75
CUT	CUT128	1.3446984	0.0011942	0.0054988	0.77	1.91	6.75	24.75
AST	AS_YHR194W	1.3466261	8.17E-05	0.0005093	2.75	7.07	25.75	89.75
NUT	NUT0601	1.347868	0.0046423	0.0181869	0.84	2.25	8.25	27.25
SUT	SUT566	1.3483375	0.0004316	0.0022138	4.34	11.09	39.50	136.75
CUT	CUT884	1.3491045	0.0039951	0.0160122	0.97	2.43	8.50	32.00
NUT	NUT1141	1.3491238	0.005641	0.0215389	0.82	2.14	7.75	27.75
NUT	NUT0030	1.3497277	0.0005428	0.0027191	2.02	5.16	19.00	71.25
SUT	SUT209	1.3508011	0.0058505	0.0221995	1.10	2.90	11.25	40.50
AST	AS_YNR021W	1.3517965	0.0108869	0.0373794	0.48	1.19	4.25	16.25
NUT	NUT0169	1.3544821	1.55E-06	1.60E-05	7.11	18.21	65.00	233.25
CUT	CUT837	1.3552147	0.0072115	0.026415	0.75	1.80	6.00	23.25
NUT	NUT0375	1.3553197	0.0007251	0.0035188	0.95	2.55	9.25	31.75
NUT	NUT1143	1.3579272	0.0013931	0.0063424	1.85	4.80	17.50	63.25
SRT	SRT248	1.3589137	0.0001768	0.0010082	3.31	8.56	30.75	108.75
NUT	NUT0958	1.3590159	5.93E-06	5.12E-05	5.93	15.26	54.50	193.50
NUT	NUT1346	1.359155	6.68E-05	0.0004297	2.97	7.70	28.25	101.25
AST	AS_YNL193W	1.3593098	7.38E-05	0.0004684	2.97	7.70	28.25	101.25

TABLE S1: Differential expression data for RRP6 RNA-Seq dataset Page 275

Class	Transcript name	RRP6 KO_vs_WT log2_fold _change	RRP6 KO_vs_WT p-value	RRP6 KO_vs_WT FDR	Ave Norm Reads in WT	Ave Norm Reads in RRP6	Average RAW read counts in WT	Average RAW read counts in RRP6
SUT	SUT335	1.3600421	0.0001772	0.0010094	3.41	8.81	31.50	112.00
NUT	NUT0689	1.3601934	0.0065527	0.0244188	0.87	2.23	7.75	27.75
NUT	NUT0946	1.3613349	0.0004595	0.0023393	1.43	3.80	14.25	50.50
NUT	NUT0508	1.3620388	0.0009054	0.0043084	1.60	4.11	14.75	54.50
NUT	NUT0483	1.3637971	0.0004248	0.0021843	3.50	8.96	31.50	121.50
SUT	SUT380	1.3639022	0.0019665	0.0086256	1.06	2.71	9.50	35.75
SRT	SRT511	1.36448	0.0011545	0.0053372	1.47	3.80	13.25	46.75
CUT	CUT500	1.3646977	0.0017982	0.0079804	0.89	2.27	8.00	30.50
NUT	NUT1016	1.365909	0.0001262	0.0007541	2.02	5.33	19.75	68.50
NUT	NUT0293	1.3662421	2.44E-05	0.0001763	6.26	16.02	55.25	215.75
NUT	NUT0250	1.3666122	0.0133286	0.0441689	0.45	1.15	4.25	16.50
SRT	SRT215	1.3670322	0.0062525	0.0234648	1.10	2.77	9.25	35.00
SUT	SUT520	1.3675529	3.96E-05	0.0002703	3.16	8.11	28.75	110.75
NUT	NUT0412	1.3675804	0.000241	0.0013289	1.84	4.84	17.75	65.50
CUT	CUT316	1.3675939	0.0005212	0.0026214	1.03	2.80	10.25	35.50
ORF-T	YPL017C	1.368034	0.0019502	0.0085576	11.14	28.82	101.50	343.00
NUT	NUT1250	1.3681848	8.44E-06	6.98E-05	3.18	8.29	29.75	107.00
NUT	NUT0932	1.3691893	1.29E-05	0.0001008	3.63	9.34	32.25	119.00
SUT	SUT076	1.3704551	3.36E-06	3.13E-05	6.03	15.63	55.00	198.25
NUT	NUT0199	1.3707592	0.0023405	0.0100134	0.87	2.23	7.75	28.75
NUT	NUT1262	1.3715438	2.00E-05	0.0001478	4.66	12.04	42.00	157.75
AST	AS_YPL018W	1.3715807	0.0035365	0.0143858	3.43	8.93	31.25	106.00
ORF-T	YHR182C-A	1.3721281	0.0288116	0.0830103	0.65	1.64	5.75	23.50
NUT	NUT1182	1.3745855	0.0002991	0.0015976	2.68	6.91	23.75	89.00
AST	AS_YNL072W	1.3753726	9.44E-06	7.68E-05	3.66	9.55	33.75	122.25
AST	AS_YAL039C	1.3755468	9.14E-06	7.47E-05	5.48	14.37	53.50	192.25
NUT	NUT0018	1.3763024	0.0006873	0.0033447	2.64	6.79	23.00	88.50
CUT	CUT241	1.3764763	0.0029946	0.0124373	1.18	3.06	10.50	38.75
SUT	SUT169	1.3768978	2.25E-05	0.0001648	3.86	10.16	37.50	134.25

Class	Transcript name	RRP6 KO_vs_WT log2_fold_change	RRP6 KO_vs_WT p-value	RRP6 KO_vs_WT FDR	Ave Norm Reads in WT	Ave Norm Reads in RRP6	Average RAW read counts in WT	Average RAW read counts in RRP6
SUT	SUT476	1.3784487	0.0035956	0.0146024	1.52	3.88	13.00	50.75
AST	AS_YJR021C	1.3787851	0.0001269	0.0007573	1.97	5.19	18.75	70.25
CUT	CUT836	1.3791242	0.0005483	0.0027398	1.26	3.14	10.50	40.75
CUT	CUT421	1.3799481	0.0025079	0.010617	2.10	5.59	20.75	71.25
SRT	SRT335	1.3829906	0.0014657	0.0066486	1.12	2.94	10.25	38.00
SUT	SUT003	1.3840944	9.18E-06	7.49E-05	5.35	14.13	52.25	188.50
NUT	NUT1045	1.3846532	0.0179311	0.056435	0.40	1.00	3.50	13.75
AST	AS_YLR256W	1.3861595	3.21E-08	5.42E-07	19.28	50.33	173.50	670.75
NUT	NUT1345	1.3869982	8.45E-05	0.0005256	3.47	9.21	34.00	122.50
SRT	SRT360	1.3884356	0.0011174	0.00519	1.24	3.27	11.50	41.75
SUT	SUT470	1.3884481	0.0011226	0.005205	1.24	3.27	11.50	41.75
AST	AS_YDR476C	1.3895399	5.45E-06	4.77E-05	4.32	11.30	38.75	144.25
NUT	NUT0512	1.3903197	0.0027344	0.0114652	0.56	1.51	5.25	19.25
AST	AS_YHR139C	1.3910026	0.0001016	0.0006196	2.37	6.33	23.25	84.25
CUT	CUT113	1.3921397	0.0005579	0.0027808	1.06	2.68	9.00	35.50
CUT	CUT387	1.3933718	0.0244477	0.0722794	0.39	1.05	3.75	14.00
SUT	SUT095	1.3941273	0.01612	0.0517687	0.34	0.92	3.25	12.25
ORF-T	YGR018C	1.3942954	0.0025405	0.0107505	0.90	2.27	7.50	29.25
CUT	CUT459	1.3965441	0.0100951	0.0349375	0.51	1.30	4.50	18.25
AST	AS_YHR050W	1.3969756	0.0010036	0.0047028	0.99	2.68	9.75	36.25
ORF-T	YPR054W	1.398059	0.0002615	0.0014255	3.39	8.91	31.50	125.25
NUT	NUT1356	1.3991306	0.0002058	0.0011526	1.65	4.25	14.25	56.25
NUT	NUT0967	1.400112	8.17E-05	0.0005093	3.96	10.45	36.75	141.00
CUT	CUT746	1.4015944	1.83E-05	0.0001371	2.63	6.95	24.00	92.00
ORF-T	YOL097W-A	1.4018531	7.49E-05	0.0004743	3.69	9.87	35.00	125.50
SUT	SUT833	1.4029186	1.88E-05	0.0001404	2.28	6.14	21.75	78.75
NUT	NUT0779	1.4034757	7.23E-05	0.0004597	2.71	7.01	23.00	93.50
CUT	CUT497	1.4044732	0.001716	0.0076532	1.04	2.85	10.25	37.25
NUT	NUT0401	1.4056814	0.0010444	0.0048804	1.56	4.05	13.25	51.00

TABLE S1: Differential expression data for RRP6 RNA-Seq dataset Page 277

Class	Transcript name	RRP6 KO_vs_WT log2_fold _change	RRP6 KO_vs_WT p-value	RRP6 KO_vs_WT FDR	Ave Norm Reads in WT	Ave Norm Reads in RRP6	Average RAW read counts in WT	Average RAW read counts in RRP6
NUT	NUT0097	1.407146	0.009377	0.0328325	0.47	1.15	3.75	15.00
SRT	SRT55	1.4080433	7.08E-05	0.0004518	3.56	9.41	31.75	122.25
SUT	SUT486	1.4094883	0.0085414	0.0303175	0.49	1.34	4.75	18.25
NUT	NUT0598	1.410016	0.0089149	0.0314217	0.42	1.10	3.75	14.50
CUT	CUT062	1.412169	0.0011556	0.0053383	0.84	2.17	7.25	28.25
NUT	NUT0635	1.4141919	0.0002776	0.0014998	2.02	5.28	17.00	65.50
ORF-T	YLR279W	1.4154606	0.0152306	0.0493209	0.54	1.32	4.25	18.75
NUT	NUT0302	1.4160604	0.0017721	0.0078754	0.67	1.80	6.25	23.75
AST	AS_YPR066W	1.4186016	1.62E-05	0.0001225	2.28	6.21	21.75	79.50
sn/snoRNA ET	SNR58-ET	1.4216109	1.57E-05	0.0001195	9.56	25.52	83.00	322.75
CUT	CUT773	1.4221083	0.0008484	0.0040585	0.93	2.53	8.75	33.75
NUT	NUT0920	1.4230116	0.0005778	0.0028686	1.54	4.22	15.25	57.75
CUT	CUT883	1.4236833	0.003887	0.0156722	0.57	1.55	5.25	19.75
CUT	CUT297	1.4272779	0.0021806	0.00942	0.93	2.51	8.75	34.00
ORF-T	YIL100C-A	1.4321027	0.0169496	0.053901	0.37	1.00	3.25	12.50
NUT	NUT0246	1.4346736	0.0017584	0.0078216	1.14	3.02	10.00	41.25
NUT	NUT0020	1.4351349	0.0002763	0.0014947	2.14	5.76	19.75	80.75
sn/snoRNA ET	SNR68-ET-FLX1	1.4354363	4.02E-09	8.88E-08	27.60	74.63	249.00	945.25
AST	AS_YDR261C	1.4355918	4.36E-05	0.0002944	3.35	9.03	29.75	117.25
NUT	NUT1422	1.4356896	0.0007548	0.0036489	0.68	1.90	6.50	24.75
AST	AS_YGR178C	1.4361854	1.05E-05	8.37E-05	4.90	13.13	43.25	177.50
NUT	NUT0037	1.436632	1.49E-05	0.000114	12.63	34.26	120.75	466.25
NUT	NUT1090	1.4366562	0.0096038	0.033487	1.04	2.95	11.25	41.25
NUT	NUT0716	1.4383344	0.0026744	0.0112465	1.04	2.75	9.25	39.50
NUT	NUT1457	1.4392454	0.0009941	0.0046678	1.80	4.70	14.75	65.75
NUT	NUT1316	1.4398901	0.0036366	0.0147453	0.60	1.69	6.00	23.00
AST	AS_YLR099C	1.4409692	7.76E-06	6.47E-05	3.05	8.37	29.25	111.50
AST	AS_YEL028W	1.4425241	5.82E-05	0.0003826	2.24	6.17	21.00	78.25
NUT	NUT0530	1.4428669	0.0055196	0.0211393	0.42	1.17	4.00	15.75

Class	Transcript name	RRP6 KO_vs_WT log2_fold _change	RRP6 KO_vs_WT p-value	RRP6 KO_vs_WT FDR	Ave Norm Reads in WT	Ave Norm Reads in RRP6	Average RAW read counts in WT	Average RAW read counts in RRP6
AST	AS_YFR015C	1.4434767	0.00099	0.0046541	1.05	2.91	10.25	40.00
AST	AS_YHR204W	1.4438973	0.0001809	0.001028	2.69	7.47	26.50	97.75
AST	AS_YLR281C	1.4443203	0.0038418	0.0155025	0.74	1.95	6.25	25.50
NUT	NUT1506	1.445216	0.0016537	0.0074076	0.56	1.55	5.25	20.50
NUT	NUT0331	1.445738	0.0002678	0.0014541	1.39	3.67	11.75	48.00
NUT	NUT0773	1.4462981	0.0066175	0.0246057	1.00	2.75	9.50	36.75
NUT	NUT0901	1.446406	1.55E-05	0.0001181	3.26	8.82	29.50	119.50
NUT	NUT1369	1.4469421	0.0087579	0.0309442	0.34	0.94	3.25	13.00
AST	AS_YLR329W	1.4478858	8.69E-06	7.15E-05	2.60	7.09	23.75	93.75
ORF-T	YMR175W	1.4479595	0.0064877	0.0242392	0.87	2.48	8.50	29.25
NUT	NUT1111	1.4519945	0.0049631	0.019331	1.02	2.87	10.00	37.50
AST	AS_YLR124W	1.4525018	0.0001342	0.0007965	1.07	2.96	10.00	39.00
NUT	NUT0052	1.453046	0.0002439	0.0013442	1.93	5.34	18.25	70.75
NUT	NUT0795	1.453083	0.0036161	0.0146682	0.59	1.52	5.00	21.50
NUT	NUT0028	1.4531715	0.0010961	0.0051008	0.97	2.70	9.00	34.00
SUT	SUT618	1.4536642	0.0001598	0.000928	2.30	6.30	20.50	79.50
NUT	NUT0043	1.4543229	0.0042921	0.0170131	0.69	1.84	6.00	24.50
NUT	NUT1052	1.4556924	4.81E-05	0.0003228	3.37	9.27	30.25	116.25
NUT	NUT1495	1.4557111	0.0003411	0.0017981	1.16	3.11	10.25	41.50
NUT	NUT0758	1.455993	0.0034501	0.0140912	0.91	2.39	7.50	31.75
CUT	CUT328	1.4566219	0.0006657	0.0032479	0.89	2.49	8.25	31.50
NUT	NUT0234	1.4570295	0.0005404	0.0027083	1.10	3.09	10.75	42.50
SRT	SRT268	1.4579128	0.0035013	0.0142654	0.56	1.50	5.00	20.50
CUT	CUT556	1.4583225	0.0067487	0.0250381	0.38	1.12	3.75	14.25
SRT	SRT516	1.4596625	0.0029651	0.0123349	0.89	2.38	7.50	30.50
AST	AS_YCR024C	1.4610417	0.0001461	0.0008595	1.43	4.02	13.75	53.75
NUT	NUT0836	1.4619324	0.002963	0.0123313	0.45	1.26	4.25	16.75
NUT	NUT0950	1.4622088	0.0016869	0.0075367	0.96	2.65	8.75	35.00
CUT	CUT400	1.4627616	0.0046162	0.0181057	0.90	2.35	7.25	31.00

TABLE S1: Differential expression data for RRP6 RNA-Seq dataset Page 279

Class	Transcript name	RRP6 KO_vs_WT log2_fold _change	RRP6 KO_vs_WT p-value	RRP6 KO_vs_WT FDR	Ave Norm Reads in WT	Ave Norm Reads in RRP6	Average RAW read counts in WT	Average RAW read counts in RRP6
NUT	NUT0411	1.4630786	6.69E-06	5.67E-05	6.30	17.51	61.50	236.00
CUT	CUT526	1.4638018	0.0174538	0.0551911	0.38	1.02	3.50	14.75
CUT	CUT002	1.4638609	0.0041775	0.0166179	0.57	1.55	5.00	19.75
NUT	NUT0081	1.464191	0.0013794	0.0062828	0.82	2.25	7.25	28.75
CUT	CUT489	1.4648571	0.0216432	0.065462	0.37	1.03	3.50	14.00
CUT	CUT246	1.4652862	0.0002304	0.0012785	2.83	7.77	25.75	104.75
SUT	SUT597	1.4659599	0.0001442	0.0008501	2.52	7.08	24.50	92.50
CUT	CUT025	1.4669902	1.24E-05	9.75E-05	11.36	31.49	109.75	428.00
NUT	NUT0221	1.4673569	0.0023284	0.0099787	0.59	1.58	5.00	20.00
SRT	SRT434	1.467902	0.0081843	0.0292351	0.45	1.20	3.75	15.00
AST	AS_YBL017C	1.4679888	0.0170322	0.0541237	0.34	0.87	2.75	11.50
NUT	NUT0190	1.4693685	0.002254	0.0097052	0.55	1.50	4.75	18.50
NUT	NUT0756	1.4700032	0.0002982	0.0015954	1.91	5.35	18.50	74.00
NUT	NUT0079	1.4708153	0.0001423	0.00084	1.35	3.83	13.00	51.25
NUT	NUT1236	1.4724534	0.0030046	0.0124686	0.64	1.89	6.75	26.25
NUT	NUT0180	1.4726464	0.0141167	0.0462934	0.37	0.96	3.00	12.75
CUT	CUT461	1.4763006	0.0003088	0.0016405	1.84	5.12	16.50	64.50
AST	AS_YJR048W	1.4776909	1.92E-05	0.0001426	3.28	9.10	29.50	118.75
NUT	NUT1064	1.4807299	0.0005245	0.0026355	0.70	1.93	6.25	24.75
ORF-T	YCR025C	1.4811618	0.001569	0.0070696	1.02	2.87	9.25	35.75
NUT	NUT0365	1.4813482	0.0074765	0.0271982	0.53	1.47	5.00	21.25
AST	AS_YNL034W	1.4845752	2.44E-05	0.0001762	1.48	4.14	13.50	54.50
NUT	NUT1065	1.4852401	1.91E-06	1.92E-05	4.59	12.76	41.25	172.50
NUT	NUT0125	1.485587	0.0002409	0.0013289	1.55	4.30	14.00	56.75
SUT	SUT232	1.4869052	1.09E-06	1.17E-05	7.73	21.74	71.50	282.75
CUT	CUT839	1.4894326	0.001166	0.0053762	1.16	3.17	10.00	43.00
CUT	CUT156	1.4909629	0.0068445	0.0253097	0.51	1.50	5.00	19.25
sn/snoRNA ET	SNR48-ET	1.4914385	2.02E-05	0.0001494	17.61	49.48	154.50	623.00
NUT	NUT0319	1.4918015	0.0017295	0.0077069	0.97	2.79	9.25	36.00

TABLE S1: Differential expression data for RRP6 RNA-Seq dataset Page 280

Class	Transcript name	RRP6 KO_vs_WT log2_fold_change	RRP6 KO_vs_WT p-value	RRP6 KO_vs_WT FDR	Ave Norm Reads in WT	Ave Norm Reads in RRP6	Average RAW read counts in WT	Average RAW read counts in RRP6
NUT	NUT0472	1.4924247	0.0078513	0.0283164	0.65	1.87	6.50	26.75
AST	AS_YKR004C	1.4940732	5.94E-05	0.000389	2.94	8.35	27.25	104.75
ORF-T	YLR366W	1.4959337	0.0006681	0.0032577	1.15	3.28	10.50	40.50
NUT	NUT0445	1.49733	0.008084	0.0289383	0.66	1.87	6.00	24.25
CUT	CUT314	1.4977677	0.0001399	0.0008275	2.90	8.15	25.50	104.25
NUT	NUT0003	1.498707	0.0005147	0.0025949	1.00	2.77	9.00	39.25
CUT	CUT001	1.4991357	0.0004749	0.0024126	1.00	2.77	9.00	39.25
CUT	CUT368	1.499353	2.59E-05	0.000186	3.06	8.57	26.75	111.75
AST	AS_YDR205W	1.5000293	0.0002885	0.0015483	1.42	4.11	14.00	56.25
AST	AS_YNR047W	1.5018973	0.0028356	0.0118413	1.00	2.84	9.50	39.25
AST	AS_YLR352W	1.504202	1.04E-05	8.32E-05	4.78	13.64	45.50	187.00
NUT	NUT1174	1.5046074	0.0001211	0.0007263	1.03	2.82	8.75	36.25
NUT	NUT0872	1.5046546	0.0004199	0.0021625	1.32	3.77	12.50	50.25
NUT	NUT1477	1.5053856	0.0017043	0.0076043	0.66	1.89	6.25	26.25
AST	AS_YMR180C	1.5057032	0.0001522	0.0008916	1.24	3.66	12.25	46.75
ORF-T	YER037W	1.5058059	2.38E-08	4.18E-07	19.24	54.73	180.25	705.75
SRT	SRT356	1.5058511	0.0087016	0.0307561	0.39	1.14	3.75	15.25
AST	AS_YLR070C	1.5062389	1.08E-07	1.55E-06	8.05	23.07	77.25	302.75
CUT	CUT693	1.5067369	0.0011905	0.0054844	0.74	2.12	6.75	27.00
SUT	SUT256	1.5070733	1.04E-07	1.50E-06	8.14	23.31	78.00	306.00
AST	AS_YLR341W	1.5076695	1.77E-07	2.37E-06	5.11	14.65	48.50	189.00
SRT	SRT306	1.5079442	0.0003932	0.0020418	1.00	2.84	9.25	39.00
NUT	NUT0504	1.508776	9.47E-08	1.38E-06	8.12	23.28	77.75	305.50
CUT	CUT599	1.5099964	0.0050402	0.0195858	0.65	1.89	6.00	23.75
SUT	SUT179	1.5121752	0.0001677	0.0009669	1.71	5.03	17.25	67.50
CUT	CUT741	1.5122086	0.0010458	0.0048845	0.88	2.50	8.00	33.50
SUT	SUT044	1.5140155	4.48E-07	5.35E-06	5.08	14.61	47.50	186.75
CUT	CUT357	1.5144942	0.0001099	0.0006654	1.71	4.92	15.75	62.50
ORF-T	YBR072C-A	1.5147671	0.0004419	0.0022599	1.70	4.81	14.75	60.75

TABLE S1: Differential expression data for RRP6 RNA-Seq dataset Page 281

Class	Transcript name	RRP6 KO_vs_WT log2_fold _change	RRP6 KO_vs_WT p-value	RRP6 KO_vs_WT FDR	Ave Norm Reads in WT	Ave Norm Reads in RRP6	Average RAW read counts in WT	Average RAW read counts in RRP6
NUT	NUT0183	1.51527	0.0003843	0.0019995	2.61	7.45	24.25	104.00
NUT	NUT0883	1.5163427	0.0035363	0.0143858	0.81	2.31	7.00	27.75
NUT	NUT0286	1.5168189	8.13E-05	0.0005085	2.71	7.62	23.75	107.00
AST	AS_YBL091C-A	1.5182156	0.0001525	0.0008931	1.17	3.21	9.75	42.00
CUT	CUT264	1.5182864	0.005431	0.0208395	0.52	1.51	4.75	19.00
NUT	NUT1219	1.5183882	0.0008813	0.0042017	0.65	1.94	6.25	24.75
AST	AS_YGR139W	1.5189117	0.0333858	0.0934394	0.35	0.98	3.00	12.25
ORF-T	YML007C-A	1.5195905	0.0046578	0.0182333	0.50	1.42	4.50	18.75
NUT	NUT0684	1.5218525	0.0004341	0.002223	0.61	1.79	5.75	23.75
AST	AS_YIL033C	1.5225547	1.08E-05	8.60E-05	6.03	17.45	58.25	236.25
CUT	CUT048	1.5231257	0.0102426	0.0353964	1.09	3.09	9.25	40.75
NUT	NUT0928	1.5239564	0.0117438	0.0397798	0.42	1.26	4.00	15.00
SRT	SRT604	1.5240157	0.0007033	0.0034178	0.75	2.15	6.75	28.00
AST	AS_YLR427W	1.5243627	0.008034	0.028831	0.49	1.41	4.50	19.50
NUT	NUT1157	1.5248272	0.0001007	0.0006162	2.09	6.02	18.75	76.00
SUT	SUT127	1.5261107	0.0001573	0.0009152	1.14	3.35	11.00	45.25
AST	AS_YMR104C	1.5263624	0.0061338	0.0231054	0.46	1.25	3.75	15.50
ORF-T	YDL086C-A	1.5274549	0.0003351	0.00177	1.76	5.21	17.75	71.00
CUT	CUT320	1.5279196	0.0030271	0.0125462	0.41	1.18	3.75	15.75
AST	AS_YER131W	1.5282323	0.0183217	0.0573063	0.48	1.38	4.50	19.25
AST	AS_YOR104W	1.5286413	0.001165	0.0053748	0.47	1.39	4.50	18.50
NUT	NUT0631	1.5296647	0.000383	0.001995	0.89	2.56	8.00	33.25
ORF-T	YER088W-B	1.52969	9.37E-08	1.37E-06	4.97	14.32	44.75	183.50
NUT	NUT1234	1.5298697	0.0002567	0.0014029	1.65	4.68	14.00	58.50
NUT	NUT1515	1.5308393	0.0045991	0.0180458	1.59	4.61	15.00	65.50
SUT	SUT836	1.5343954	0.0187274	0.0583772	1.37	3.98	13.00	57.25
sn/snoRNA ET	SNR128-ET	1.536526	8.80E-06	7.24E-05	28.27	81.80	233.50	1023.50
NUT	NUT0370	1.537211	3.61E-06	3.33E-05	7.41	21.66	71.75	291.75
AST	AS_YGL101W	1.5382925	0.0002243	0.0012467	0.92	2.64	8.25	35.25

Class	Transcript name	RRP6 KO_vs_WT log2_fold _change	RRP6 KO_vs_WT p-value	RRP6 KO_vs_WT FDR	Ave Norm Reads in WT	Ave Norm Reads in RRP6	Average RAW read counts in WT	Average RAW read counts in RRP6
NUT	NUT1272	1.5385668	6.04E-06	5.20E-05	4.94	14.42	46.75	197.50
NUT	NUT1114	1.5396123	3.40E-05	0.0002368	1.44	4.22	13.50	56.75
NUT	NUT0073	1.5404381	0.003665	0.0148425	0.58	1.58	4.75	21.50
NUT	NUT1363	1.5406908	1.54E-06	1.60E-05	5.65	16.42	50.50	201.00
SUT	SUT120	1.5410912	0.0023349	0.009998	0.40	1.23	4.00	16.50
SUT	SUT282	1.5410914	0.0001009	0.0006171	1.29	3.79	12.00	48.75
SUT	SUT299	1.5414682	0.0003077	0.0016365	1.37	3.99	12.50	53.75
NUT	NUT1425	1.5418067	5.75E-05	0.0003793	2.76	7.99	25.25	109.50
NUT	NUT1266	1.5418815	3.69E-07	4.50E-06	3.38	9.81	30.75	131.50
SUT	SUT644	1.5423268	8.03E-07	8.99E-06	4.79	13.91	43.00	181.00
NUT	NUT1221	1.5428137	0.0087939	0.0310467	0.35	1.13	3.75	14.50
NUT	NUT0011	1.5439567	3.54E-05	0.000245	1.60	4.49	13.25	58.50
SRT	SRT413	1.5441107	0.0007398	0.0035851	0.62	1.78	5.50	23.25
CUT	CUT601	1.544111	0.0007398	0.0035851	0.62	1.78	5.50	23.25
NUT	NUT0101	1.5446556	3.20E-07	3.96E-06	4.93	14.49	46.25	185.25
NUT	NUT1175	1.5454249	7.69E-05	0.0004862	1.28	3.90	12.75	50.25
NUT	NUT1270	1.5464622	8.01E-08	1.19E-06	5.68	16.72	54.00	216.25
SUT	SUT278	1.5470125	0.0018709	0.0082637	0.95	2.81	8.75	34.75
NUT	NUT0523	1.5480833	6.53E-06	5.56E-05	6.74	19.68	60.75	259.75
AST	AS_YLR265C	1.5490429	0.0018926	0.008345	0.61	1.66	5.00	22.50
AST	AS_YFR007W	1.5492884	4.13E-07	4.97E-06	3.59	10.50	32.50	137.00
NUT	NUT1319	1.5493329	0.0055089	0.0211141	0.62	1.91	6.50	26.75
CUT	CUT121	1.5493797	0.0018216	0.0080674	0.53	1.50	4.50	19.00
AST	AS_YKR058W	1.549965	0.0034317	0.0140273	0.57	1.81	6.25	25.25
SUT	SUT270	1.5502144	6.38E-06	5.45E-05	6.74	19.71	60.75	260.25
NUT	NUT0741	1.551348	0.0005482	0.0027398	1.10	3.27	10.25	42.25
NUT	NUT0705	1.5514058	2.98E-05	0.0002103	2.21	6.50	20.25	84.50
NUT	NUT0619	1.5517039	8.07E-05	0.0005059	1.20	3.55	11.00	45.50
NUT	NUT0590	1.5519477	0.0009739	0.004589	1.78	5.09	14.50	64.25

Class	Transcript name	RRP6 KO_vs_WT log2_fold _change	RRP6 KO_vs_WT p-value	RRP6 KO_vs_WT FDR	Ave Norm Reads in WT	Ave Norm Reads in RRP6	Average RAW read counts in WT	Average RAW read counts in RRP6
NUT	NUT0463	1.5519789	3.87E-05	0.0002648	2.74	8.11	25.50	101.50
NUT	NUT1017	1.5534878	0.0081039	0.028989	0.71	2.03	6.25	28.75
CUT	CUT267	1.553739	0.0019333	0.0085059	0.57	1.58	4.75	21.25
CUT	CUT209	1.5565043	0.0053581	0.0205975	0.39	1.02	3.00	13.50
SRT	SRT450	1.5575179	0.0058413	0.0221935	0.42	1.24	3.75	15.75
AST	AS_YER038C	1.55833	4.54E-06	4.05E-05	5.09	15.09	48.25	192.25
CUT	CUT749	1.5591934	0.0015226	0.0068912	0.55	1.57	4.75	20.50
sn/snoRNA ET	snR53-ET	1.5593893	0.0002587	0.0014108	2.11	6.27	19.25	78.25
NUT	NUT0142	1.5605195	1.06E-06	1.15E-05	3.43	10.05	30.50	134.00
CUT	CUT675	1.5615698	0.0052172	0.0201343	0.44	1.33	4.00	16.75
AST	AS_YBR292C	1.5616757	0.0017951	0.0079744	0.68	1.95	5.75	24.50
NUT	NUT1474	1.5619206	0.005183	0.0200447	0.68	1.94	5.75	25.25
CUT	CUT600	1.5620253	0.0001231	0.0007371	1.90	5.48	15.75	68.25
CUT	CUT057	1.5626258	0.0039027	0.0157168	0.34	1.04	3.25	13.75
SUT	SUT707	1.5652238	5.73E-08	8.94E-07	5.61	16.72	53.25	216.25
CUT	CUT460	1.5664907	1.56E-05	0.0001187	3.09	9.03	27.00	120.25
SRT	SRT227	1.5668818	0.0002004	0.0011254	0.77	2.33	7.25	30.25
NUT	NUT1190	1.56836	9.12E-05	0.0005634	1.01	3.06	9.50	39.50
AST	AS_YKR062W	1.5684647	2.17E-05	0.0001592	2.75	8.27	26.75	112.00
SUT	SUT224	1.5687176	0.0050866	0.0197283	2.36	7.08	22.00	83.00
AST	AS_YDL025C	1.5693426	0.0001895	0.0010698	1.55	4.68	15.25	64.25
NUT	NUT1245	1.5698081	0.0005212	0.0026214	0.83	2.43	7.25	30.50
AST	AS_YLR149C	1.5713769	1.29E-05	0.0001008	3.30	9.95	32.00	129.75
NUT	NUT1071	1.5715206	0.0018918	0.008345	0.39	1.19	3.75	16.00
SRT	SRT586	1.5716721	0.0015405	0.0069631	0.53	1.53	4.50	19.25
CUT	CUT418	1.5718487	0.0027131	0.01139	0.50	1.48	4.50	19.50
NUT	NUT0797	1.5725157	6.96E-07	7.91E-06	3.73	11.14	35.00	150.00
NUT	NUT1194	1.5751893	1.67E-07	2.26E-06	5.83	17.31	52.25	226.25
NUT	NUT0150	1.5757977	0.0041732	0.0166072	0.35	1.11	3.50	14.75

TABLE S1: Differential expression data for RRP6 RNA-Seq dataset Page 284

Class	Transcript name	RRP6 KO_vs_WT log2_fold _change	RRP6 KO_vs_WT p-value	RRP6 KO_vs_WT FDR	Ave Norm Reads in WT	Ave Norm Reads in RRP6	Average RAW read counts in WT	Average RAW read counts in RRP6
SUT	SUT233	1.576434	5.48E-05	0.0003627	1.14	3.48	11.00	46.25
NUT	NUT1463	1.5765927	0.0004333	0.0022204	2.54	7.55	23.00	101.00
AST	AS_YKL128C	1.5766092	6.30E-07	7.24E-06	3.07	9.15	27.75	119.75
CUT	CUT163	1.5777126	0.0010617	0.0049541	0.54	1.53	4.50	19.50
SUT	SUT146	1.5781919	0.0001053	0.0006394	1.07	3.08	9.00	40.00
NUT	NUT0321	1.5786798	0.0015826	0.0071104	0.74	2.23	7.00	30.50
NUT	NUT0666	1.5791292	0.0115262	0.0392139	0.42	1.29	4.00	16.50
AST	AS_YJL019W	1.5793972	6.50E-05	0.0004194	2.17	6.43	19.00	83.25
AST	AS_YBR060C	1.5796131	0.0001891	0.001068	1.21	3.74	11.75	46.75
CUT	CUT751	1.5798312	1.31E-05	0.0001022	1.71	5.21	16.50	70.25
NUT	NUT1271	1.5804391	0.0018313	0.0081028	0.42	1.26	3.75	15.75
CUT	CUT305	1.5817695	0.0040779	0.0162922	0.58	1.80	5.50	21.75
NUT	NUT0573	1.5839177	2.11E-05	0.0001551	1.95	5.89	18.25	78.25
NUT	NUT0276	1.5845852	2.62E-05	0.0001881	4.55	13.69	41.75	173.25
NUT	NUT1282	1.5867375	0.0012822	0.0058691	0.54	1.63	5.00	21.75
SUT	SUT431	1.586913	0.0008541	0.0040817	0.44	1.32	4.00	17.50
SRT	SRT280	1.5869424	0.0009943	0.0046678	0.88	2.74	8.50	33.75
NUT	NUT0516	1.5875036	1.08E-05	8.60E-05	3.34	10.19	32.50	133.00
NUT	NUT0419	1.5878567	0.0007418	0.0035912	0.92	2.80	8.50	35.00
NUT	NUT1498	1.591008	2.57E-07	3.27E-06	5.94	17.98	56.25	243.75
AST	AS_YDR085C	1.5916618	0.0001298	0.0007733	1.40	4.16	12.50	54.75
NUT	NUT1056	1.5922247	0.0052764	0.0203314	0.80	2.37	7.00	33.75
AST	AS_YJR096W	1.5922351	5.48E-07	6.38E-06	3.97	11.89	35.50	156.00
AST	AS_YEL060C	1.5924554	0.0001819	0.0010325	2.42	7.29	22.50	101.50
NUT	NUT0413	1.5929355	0.0070456	0.0259111	0.36	1.06	3.25	14.75
CUT	CUT353	1.593593	0.001001	0.0046948	0.49	1.43	4.25	19.00
NUT	NUT0669	1.5936072	0.0009994	0.0046896	0.49	1.43	4.25	19.00
NUT	NUT0991	1.5941883	0.016282	0.0521562	0.40	1.23	4.00	18.00
NUT	NUT1011	1.5942766	4.95E-07	5.85E-06	3.13	9.59	30.00	123.50

Class	Transcript name	RRP6 KO_vs_WT log2_fold _change	RRP6 KO_vs_WT p-value	RRP6 KO_vs_WT FDR	Ave Norm Reads in WT	Ave Norm Reads in RRP6	Average RAW read counts in WT	Average RAW read counts in RRP6
NUT	NUT0361	1.5949131	0.0001661	0.0009592	2.88	8.88	29.00	118.50
CUT	CUT352	1.5953925	0.0062994	0.0236147	0.44	1.38	4.25	17.50
AST	AS_YJR053W	1.5959763	8.45E-05	0.0005256	0.97	2.97	9.00	38.50
AST	AS_YKL096W	1.596836	0.0024066	0.0102614	0.49	1.59	5.00	20.25
CUT	CUT064	1.5970479	2.95E-05	0.0002082	1.53	4.73	14.50	60.25
NUT	NUT1335	1.5990647	4.41E-05	0.0002982	0.88	2.55	7.50	33.75
SRT	SRT249	1.5996973	0.0011549	0.0053372	0.66	2.07	6.25	26.00
SUT	SUT264	1.5997257	0.0021049	0.0091372	0.51	1.55	4.75	20.75
NUT	NUT0518	1.599726	0.0021063	0.0091394	0.51	1.55	4.75	20.75
AST	AS_YJR019C	1.6010248	1.24E-06	1.31E-05	4.70	14.38	45.25	192.25
AST	AS_YNL126W	1.6031667	0.0067943	0.0251758	0.28	0.97	3.00	12.25
NUT	NUT0589	1.6036678	4.34E-05	0.0002935	2.32	7.09	21.50	95.25
NUT	NUT0083	1.6036812	0.0098208	0.0341377	0.95	2.83	8.00	37.50
AST	AS_YGL198W	1.6041768	6.02E-06	5.18E-05	1.78	5.49	16.75	70.25
NUT	NUT1438	1.604416	5.22E-05	0.0003465	2.02	6.17	18.50	80.50
AST	AS_YHR087W	1.6047676	0.0001	0.0006125	1.05	3.11	9.00	40.50
NUT	NUT1448	1.6063025	0.0002086	0.0011666	1.07	3.17	9.25	42.00
CUT	CUT094	1.6065887	0.003081	0.0127385	0.37	1.15	3.50	15.50
SUT	SUT225	1.6076981	0.0018381	0.0081261	3.45	10.56	31.25	125.75
NUT	NUT0447	1.6080425	1.20E-07	1.70E-06	8.77	26.63	78.50	348.75
NUT	NUT1423	1.6087013	1.66E-05	0.0001253	3.17	9.59	28.25	129.25
CUT	CUT043	1.6108306	5.02E-05	0.0003353	2.58	7.84	22.25	96.75
NUT	NUT0879	1.6110395	0.0001733	0.0009915	3.39	10.43	31.25	129.25
CUT	CUT670	1.6123705	0.0051349	0.0198773	0.29	0.93	2.75	11.75
AST	AS_YJR140C	1.6138722	0.003642	0.0147611	0.39	1.21	3.75	16.75
CUT	CUT216	1.6139126	0.0021691	0.0093754	0.63	1.92	5.50	23.50
AST	AS_YPR030W	1.6150095	1.35E-07	1.88E-06	5.65	17.38	53.25	235.50
SUT	SUT641	1.6151547	4.57E-06	4.08E-05	3.28	10.02	29.50	130.50
CUT	CUT194	1.6152233	0.0017525	0.0077989	0.38	1.10	3.25	14.75

Class	Transcript name	RRP6 KO_vs_WT log2_fold _change	RRP6 KO_vs_WT p-value	RRP6 KO_vs_WT FDR	Ave Norm Reads in WT	Ave Norm Reads in RRP6	Average RAW read counts in WT	Average RAW read counts in RRP6
CUT	CUT313	1.6156894	0.0158354	0.0509685	0.28	0.85	2.50	11.25
CUT	CUT801	1.6166765	6.90E-05	0.0004418	0.82	2.41	7.00	32.00
SUT	SUT420	1.617427	3.75E-07	4.58E-06	3.35	10.32	31.25	138.50
CUT	CUT898	1.6176791	0.0007806	0.0037628	0.91	2.93	9.00	35.75
NUT	NUT0843	1.6193854	0.0020943	0.0090952	0.72	2.21	6.50	29.00
AST	AS_YER089C	1.6198014	2.15E-08	3.83E-07	4.59	14.09	41.50	180.50
ORF-T	YNL228W	1.6199196	0.0004505	0.002298	0.56	1.66	4.75	21.00
SRT	SRT393	1.6201486	0.0107664	0.0370161	0.30	0.90	2.75	12.50
NUT	NUT1492	1.6205633	0.004484	0.0176627	0.35	1.16	3.50	14.50
NUT	NUT0986	1.6207671	1.83E-08	3.32E-07	5.65	17.31	50.75	226.50
NUT	NUT0019	1.6225822	0.0003209	0.0017013	0.64	2.02	6.00	25.75
ORF-T	YPR087W	1.6240082	0.0090319	0.0318007	0.31	0.94	2.75	12.50
SUT	SUT831	1.6244027	0.0001646	0.0009518	1.52	4.66	14.00	64.75
CUT	CUT756	1.6246795	0.0009437	0.004468	0.54	1.67	5.00	22.25
AST	AS_YHR029C	1.6256025	5.87E-05	0.0003853	1.18	3.57	10.25	46.75
AST	AS_YDL185W	1.6256833	0.0189094	0.0587983	0.55	1.70	5.00	22.25
NUT	NUT0617	1.6260921	0.0003985	0.0020648	0.71	2.03	5.75	27.25
NUT	NUT0402	1.6272042	0.0003963	0.0020565	0.88	2.73	8.00	35.00
SUT	SUT188	1.6273964	1.53E-06	1.59E-05	6.70	20.85	65.00	281.50
CUT	CUT734	1.627871	0.0019447	0.0085412	0.45	1.37	4.00	18.00
NUT	NUT1329	1.6291919	2.29E-05	0.000167	1.88	5.80	17.00	76.50
NUT	NUT1514	1.63305	2.90E-06	2.77E-05	3.46	10.63	30.50	143.25
CUT	CUT705	1.6339149	7.96E-05	0.0004996	1.62	5.05	15.00	67.00
SRT	SRT512	1.6340405	0.0009403	0.0044581	0.60	1.90	5.75	25.75
NUT	NUT1042	1.6343154	0.0004284	0.0021986	0.93	2.89	8.50	37.50
CUT	CUT775	1.6355932	5.05E-06	4.46E-05	1.47	4.45	12.75	59.25
NUT	NUT0032	1.6362512	0.0062194	0.0233894	0.36	1.10	3.25	15.00
AST	AS_YJL060W	1.6369085	0.0001119	0.0006765	0.83	2.64	8.00	35.75
CUT	CUT876	1.636981	0.0068929	0.02547	0.48	1.40	4.00	20.00

TABLE S1: Differential expression data for RRP6 RNA-Seq dataset Page 287

Class	Transcript name	RRP6 KO_vs_WT log2_fold _change	RRP6 KO_vs_WT p-value	RRP6 KO_vs_WT FDR	Ave Norm Reads in WT	Ave Norm Reads in RRP6	Average RAW read counts in WT	Average RAW read counts in RRP6
CUT	CUT783	1.6378878	0.0014071	0.0064001	0.50	1.58	4.75	21.50
AST	AS_YLR135W	1.6381079	4.17E-06	3.78E-05	1.69	5.18	15.00	69.75
SUT	SUT129	1.6382766	6.48E-05	0.0004179	2.47	7.61	21.50	98.00
AST	AS_YOR367W	1.6393724	0.0024432	0.0103888	0.34	1.10	3.25	14.25
NUT	NUT1387	1.6415485	3.86E-06	3.53E-05	4.41	13.95	42.75	177.00
CUT	CUT072	1.6419683	8.08E-05	0.000506	1.35	4.24	12.50	54.75
CUT	CUT102	1.6421907	0.0034587	0.0141203	0.85	2.74	8.25	33.50
AST	AS_YOL104C	1.6431168	0.0011331	0.0052486	0.41	1.33	4.00	17.75
CUT	CUT134	1.6433259	0.0004536	0.0023101	0.60	1.90	5.50	24.50
NUT	NUT0336	1.6434433	0.0001726	0.0009882	1.29	4.05	12.00	53.25
NUT	NUT0834	1.6438699	5.16E-06	4.54E-05	2.17	6.81	20.00	87.75
NUT	NUT0021	1.6445963	0.0004225	0.002175	1.54	4.65	12.50	61.75
AST	AS_YDR106W	1.6469078	0.000601	0.0029637	0.78	2.55	7.75	33.00
AST	AS_YKL045W	1.6485558	5.06E-05	0.0003376	1.92	5.97	17.00	78.25
SRT	SRT533	1.649345	0.0124228	0.0416738	0.59	1.89	6.00	27.75
AST	AS_YPL019C	1.649862	0.0001366	0.0008093	1.00	3.13	9.25	43.25
NUT	NUT1214	1.6500403	5.23E-05	0.0003466	2.13	6.77	20.00	85.25
AST	AS_YNL078W	1.6507195	1.06E-06	1.15E-05	4.72	14.79	42.00	180.25
CUT	CUT345	1.6510206	0.0004269	0.0021928	0.70	2.25	6.50	28.00
AST	AS_YEL027W	1.651028	0.0077184	0.0279069	0.31	1.00	3.00	14.00
SUT	SUT744	1.6510639	4.31E-06	3.89E-05	2.25	6.96	19.50	91.50
NUT	NUT1324	1.6539735	0.0032406	0.0133273	0.41	1.29	3.75	17.25
SRT	SRT44	1.6560142	1.06E-05	8.50E-05	1.51	4.86	14.25	62.00
CUT	CUT793	1.6564319	4.44E-05	0.0002995	1.15	3.60	10.25	46.75
CUT	CUT151	1.6566279	9.46E-06	7.68E-05	4.18	13.24	38.50	167.00
NUT	NUT0566	1.6568672	0.0003967	0.0020575	0.81	2.56	7.25	32.25
CUT	CUT479	1.6578745	2.51E-07	3.20E-06	2.50	7.93	23.00	101.25
NUT	NUT1340	1.6586854	0.011376	0.0388303	0.64	2.10	6.75	30.75
CUT	CUT334	1.6587366	0.0041094	0.0163791	0.29	1.00	3.00	13.25

TABLE S1: Differential expression data for RRP6 RNA-Seq dataset Page 288

Class	Transcript name	RRP6 KO_vs_WT log2_fold _change	RRP6 KO_vs_WT p-value	RRP6 KO_vs_WT FDR	Ave Norm Reads in WT	Ave Norm Reads in RRP6	Average RAW read counts in WT	Average RAW read counts in RRP6
NUT	NUT0752	1.6592074	9.45E-06	7.68E-05	1.41	4.45	12.75	57.00
SUT	SUT466	1.6592971	5.80E-05	0.0003815	1.33	4.14	11.50	52.00
NUT	NUT0140	1.6596181	0.0001609	0.0009336	11.32	35.85	107.00	458.75
NUT	NUT1241	1.6601527	0.0047613	0.0185954	0.34	1.10	3.25	15.00
CUT	CUT106	1.66053	0.0005715	0.0028416	1.56	4.92	13.75	60.25
AST	AS_YJL160C	1.6605921	3.73E-05	0.000257	1.23	3.88	11.25	51.00
AST	AS_YLR127C	1.6606333	0.0004333	0.0022204	0.56	1.82	5.25	23.25
AST	AS_YGL230C	1.6630276	0.0003721	0.0019464	1.17	3.77	11.00	47.75
NUT	NUT0632	1.6631839	5.52E-06	4.82E-05	2.17	6.78	19.25	90.75
SUT	SUT708	1.6637313	1.34E-05	0.0001036	2.15	6.76	18.75	85.00
NUT	NUT0055	1.6638615	2.70E-05	0.0001925	1.85	5.91	17.25	77.25
AST	AS_YDL054C	1.6639524	0.0001707	0.0009795	0.70	2.20	6.25	28.25
NUT	NUT0025	1.6650672	0.0024869	0.0105415	0.64	1.98	5.50	27.00
NUT	NUT1076	1.6656235	1.23E-05	9.61E-05	4.86	15.44	44.00	189.50
NUT	NUT1306	1.665867	3.19E-06	3.00E-05	1.47	4.54	12.75	60.50
CUT	CUT366	1.6662309	0.0038955	0.0156941	0.36	1.21	3.50	15.50
AST	AS_YER175W-A	1.6668307	0.0001449	0.0008536	0.75	2.39	7.00	32.75
AST	AS_YIL120W	1.6672643	5.48E-06	4.79E-05	3.10	9.70	26.50	130.25
CUT	CUT341	1.6676712	0.0001012	0.000618	0.90	2.84	8.00	36.50
CUT	CUT561	1.6696192	1.57E-06	1.62E-05	2.40	7.78	23.00	99.00
SUT	SUT808	1.6702565	0.001403	0.0063846	0.58	1.81	5.00	23.50
CUT	CUT747	1.6714822	0.0003786	0.0019749	0.50	1.65	4.75	21.25
AST	AS_YGL117W	1.6730714	0.0001178	0.0007082	1.77	5.48	14.75	74.00
AST	AS_YPL221W	1.6732369	0.0008012	0.0038546	1.64	5.18	14.50	70.25
NUT	NUT0899	1.6734211	2.50E-05	0.0001799	1.65	5.19	14.25	65.25
AST	AS_YCL025C	1.6743699	0.0037523	0.0151596	0.62	1.86	5.00	25.75
CUT	CUT821	1.6756614	0.0001186	0.0007124	1.11	3.47	9.50	44.00
CUT	CUT750	1.6769135	0.0017996	0.0079804	0.37	1.17	3.25	14.50
SRT	SRT17	1.6774108	0.0128336	0.0428948	0.45	1.53	4.75	21.25

Class	Transcript name	RRP6 KO_vs_WT log2_fold _change	RRP6 KO_vs_WT p-value	RRP6 KO_vs_WT FDR	Ave Norm Reads in WT	Ave Norm Reads in RRP6	Average RAW read counts in WT	Average RAW read counts in RRP6
CUT	CUT899	1.6780903	7.10E-07	8.06E-06	4.21	13.52	38.50	171.50
SUT	SUT065	1.6789223	0.0001634	0.0009472	10.79	34.62	102.25	441.00
NUT	NUT1198	1.6790039	0.0001676	0.0009669	1.09	3.49	9.75	44.00
SUT	SUT746	1.6827575	2.56E-06	2.49E-05	2.46	8.06	24.25	107.25
AST	AS_YHR132C	1.6835304	0.0006346	0.0031139	0.73	2.43	7.00	30.25
CUT	CUT462	1.6851434	0.0025539	0.0107983	0.58	1.94	5.50	24.00
NUT	NUT0678	1.6855237	1.65E-06	1.69E-05	1.77	5.67	16.00	74.25
AST	AS_YDR533C	1.6855991	8.17E-05	0.0005093	0.55	1.76	5.00	23.25
SRT	SRT137	1.6876486	0.0007514	0.0036339	0.41	1.33	3.75	17.25
NUT	NUT0089	1.6887037	0.0032251	0.013269	0.28	0.97	2.75	12.25
SUT	SUT246	1.6887119	4.48E-06	4.03E-05	3.36	10.88	31.00	141.25
NUT	NUT0159	1.6887613	0.0002906	0.001557	0.64	2.15	6.50	30.00
CUT	CUT360	1.6889385	0.0002563	0.0014016	1.18	3.75	10.25	48.00
NUT	NUT0106	1.6893638	1.14E-06	1.22E-05	2.53	8.16	23.25	109.00
AST	AS_YPL104W	1.6898868	0.0009681	0.0045661	0.48	1.68	5.00	22.00
SUT	SUT780	1.6903552	0.0009518	0.0044999	0.39	1.24	3.50	16.50
AST	AS_YHR110W	1.6913705	0.0005751	0.0028567	0.58	1.94	5.50	24.00
AST	AS_YLR328W	1.6922074	0.0005451	0.0027294	0.64	2.01	5.75	28.50
sn/snoRNA ET	SNR83-ET	1.6924576	1.65E-07	2.24E-06	9.76	31.49	86.00	402.75
NUT	NUT1310	1.6937323	2.51E-06	2.44E-05	1.22	4.01	11.50	52.75
NUT	NUT0385	1.6946101	9.74E-08	1.42E-06	6.31	20.34	55.50	260.25
CUT	CUT199	1.6952855	1.54E-05	0.0001177	1.25	4.06	11.50	53.50
SUT	SUT549	1.696971	0.0003466	0.0018251	0.68	2.15	5.75	26.50
sn/snoRNA ET	snR161-ET	1.6969885	8.09E-08	1.21E-06	6.88	22.32	62.00	279.75
AST	AS_YOL147C	1.6972949	1.15E-06	1.23E-05	1.82	5.88	16.50	77.00
AST	AS_YGR062C	1.6986188	7.90E-06	6.57E-05	3.86	12.60	35.25	158.50
NUT	NUT1341	1.698983	1.04E-09	2.67E-08	6.74	21.84	61.00	287.75
SUT	SUT786	1.7009759	1.30E-05	0.0001017	1.15	3.63	10.00	49.00
CUT	CUT647	1.7027938	5.00E-05	0.0003341	0.93	3.02	8.25	37.50

Class	Transcript name	RRP6 KO_vs_WT log2_fold _change	RRP6 KO_vs_WT p-value	RRP6 KO_vs_WT FDR	Ave Norm Reads in WT	Ave Norm Reads in RRP6	Average RAW read counts in WT	Average RAW read counts in RRP6
SUT	SUT779	1.7032491	7.21E-06	6.08E-05	2.81	9.07	25.00	122.25
AST	AS_YKR060W	1.7043121	0.0001635	0.0009473	0.94	3.08	8.75	40.25
NUT	NUT0484	1.7052162	3.25E-06	3.04E-05	3.36	11.00	31.00	142.75
AST	AS_YPL111W	1.7053443	1.38E-05	0.0001067	0.96	3.18	9.00	41.25
CUT	CUT109	1.7057796	0.0001637	0.0009473	0.63	1.96	5.25	25.00
AST	AS_YMR283C	1.7060799	4.14E-05	0.000281	0.88	2.93	8.25	38.00
SUT	SUT792	1.7069138	2.26E-06	2.23E-05	2.05	6.72	18.75	87.75
SUT	SUT722	1.7071787	3.84E-05	0.0002633	1.36	4.49	12.75	58.50
NUT	NUT0291	1.7075621	0.0020022	0.0087554	0.39	1.18	3.25	16.25
AST	AS_YBR222C	1.7116598	2.33E-05	0.0001695	1.74	5.72	16.00	75.00
AST	AS_YPL059W	1.7118822	6.32E-06	5.42E-05	2.51	8.28	23.25	105.00
SUT	SUT783	1.7122805	7.86E-05	0.0004956	2.36	7.68	20.50	95.75
AST	AS_YKL121W	1.7125026	2.26E-05	0.0001653	1.92	6.37	18.00	80.25
ORF-T	YML066C	1.7129224	0.0004244	0.0021836	0.50	1.68	4.75	22.25
AST	AS_YDR277C	1.7130662	0.0001789	0.0010189	6.33	20.82	59.25	267.00
CUT	CUT633	1.7131442	0.0003478	0.0018296	0.83	2.71	7.50	35.50
NUT	NUT0299	1.7146787	2.90E-05	0.0002049	1.36	4.40	12.00	59.00
AST	AS_YGL128C	1.7153833	6.80E-05	0.0004365	0.68	2.25	6.25	29.25
SUT	SUT349	1.7155529	3.22E-06	3.02E-05	1.88	6.19	17.25	80.75
AST	AS_YHR176W	1.7161671	5.28E-06	4.63E-05	1.16	3.83	10.75	51.50
SRT	SRT234	1.7165825	0.0002579	0.0014075	0.53	1.68	4.50	21.25
AST	AS_YNL210W	1.7169509	3.94E-06	3.60E-05	2.02	6.79	19.75	90.50
AST	AS_YLR186W	1.7193503	0.0007407	0.0035873	0.48	1.66	4.75	22.25
AST	AS_YDL078C	1.7196389	1.41E-06	1.48E-05	2.34	7.90	23.25	104.75
CUT	CUT902	1.7213685	2.02E-05	0.0001494	1.27	4.21	11.75	55.75
CUT	CUT779	1.7224613	1.85E-05	0.0001378	0.75	2.57	7.25	33.25
NUT	NUT1240	1.7226469	0.0006097	0.0030036	0.36	1.23	3.50	16.75
AST	AS_YJR095W	1.7227094	6.13E-05	0.000399	0.96	3.13	8.50	41.50
NUT	NUT1027	1.7239634	4.75E-05	0.0003192	2.02	6.49	16.75	88.25

Class	Transcript name	RRP6 KO_vs_WT log2_fold_change	RRP6 KO_vs_WT p-value	RRP6 KO_vs_WT FDR	Ave Norm Reads in WT	Ave Norm Reads in RRP6	Average RAW read counts in WT	Average RAW read counts in RRP6
AST	AS_YPL040C	1.724229	0.0008028	0.0038604	0.50	1.82	5.25	23.25
AST	AS_YDL107W	1.7249527	0.0005867	0.0029087	0.33	1.15	3.25	15.25
NUT	NUT0203	1.7259603	0.0001527	0.0008931	0.60	2.10	6.00	27.50
AST	AS_YLR390W	1.7268761	2.64E-05	0.0001885	1.18	3.94	11.00	51.25
NUT	NUT0148	1.7271731	0.0003278	0.001735	0.61	2.07	5.75	26.50
CUT	newCUT2	1.7271881	3.29E-05	0.0002298	2.85	9.47	25.75	120.25
CUT	CUT501	1.7298061	0.0003339	0.0017648	0.64	2.23	6.25	27.50
CUT	CUT833	1.7301214	0.0008112	0.0038987	0.60	2.05	5.75	27.25
NUT	NUT0251	1.7305765	9.50E-05	0.0005853	0.66	2.19	6.00	28.50
CUT	CUT058	1.7309503	0.0039121	0.0157421	0.54	1.82	5.00	22.50
NUT	NUT0112	1.7309503	0.0039121	0.0157421	0.54	1.82	5.00	22.50
AST	AS_YOL005C	1.731005	1.04E-05	8.33E-05	2.04	6.70	18.00	87.00
NUT	NUT1136	1.7311168	4.07E-06	3.70E-05	2.98	9.75	25.50	130.50
AST	AS_YPR052C	1.7315059	2.34E-05	0.0001699	1.76	5.80	16.00	78.25
CUT	CUT205	1.7323518	5.10E-05	0.0003395	1.83	6.09	16.50	77.50
NUT	NUT0771	1.7323537	4.33E-05	0.0002932	0.75	2.57	7.25	34.50
AST	AS_YGR263C	1.7323846	1.17E-05	9.24E-05	1.83	6.11	17.00	81.50
ORF-T	YNL235C	1.7324792	0.0014201	0.0064536	1.10	3.57	9.25	46.75
AST	AS_YER079W	1.7333561	8.91E-05	0.0005517	1.70	5.69	16.00	76.50
NUT	NUT1365	1.7335978	0.0008209	0.0039402	0.55	1.83	5.00	24.00
NUT	NUT0211	1.7347324	5.53E-07	6.42E-06	2.03	6.84	19.25	89.50
NUT	NUT0842	1.7351497	4.09E-05	0.0002783	2.88	9.62	26.00	122.50
SRT	SRT603	1.7353726	6.37E-06	5.45E-05	1.77	5.98	16.75	79.00
SUT	SUT720	1.7354975	1.57E-09	3.92E-08	15.23	50.76	139.00	650.25
NUT	NUT0249	1.7368091	0.0001335	0.0007939	0.83	2.88	8.25	39.00
NUT	NUT0575	1.7372895	2.36E-05	0.0001711	2.57	8.73	25.25	118.25
NUT	NUT0208	1.7376542	0.0002119	0.0011818	1.03	3.45	9.50	46.50
AST	AS_YOR236W	1.737863	2.63E-05	0.0001883	1.73	5.73	15.25	74.25
NUT	NUT0311	1.7379924	1.16E-06	1.23E-05	1.80	6.06	16.75	78.25

Class	Transcript name	RRP6 KO_vs_WT log2_fold _change	RRP6 KO_vs_WT p-value	RRP6 KO_vs_WT FDR	Ave Norm Reads in WT	Ave Norm Reads in RRP6	Average RAW read counts in WT	Average RAW read counts in RRP6
NUT	NUT0177	1.7388834	4.03E-05	0.0002751	0.97	3.35	9.50	43.50
CUT	CUT420	1.7393689	0.0001838	0.0010421	0.83	2.99	8.75	38.25
AST	AS_YBR286W	1.740354	0.0001529	0.0008939	0.78	2.45	6.25	32.00
CUT	CUT146	1.7406243	7.58E-06	6.36E-05	5.59	18.63	49.00	236.00
NUT	NUT1105	1.7411489	9.59E-05	0.0005903	0.96	3.26	9.00	41.75
NUT	NUT0084	1.7450374	0.0092629	0.0325345	0.31	1.10	3.00	13.25
ORF-T	YGR240C-A	1.7473492	0.0009331	0.00443	0.42	1.53	4.25	19.00
AST	AS_YIL048W	1.7480246	4.60E-05	0.0003095	2.02	6.91	19.50	88.25
ORF-T	YCR081C-A	1.7482285	0.0002491	0.0013669	0.98	3.16	8.00	42.00
AST	AS_YHL002W	1.7515699	0.00045	0.0022976	1.07	3.62	9.75	47.25
SRT	SRT36	1.7517775	0.0003272	0.0017327	0.47	1.65	4.50	21.25
NUT	NUT0780	1.7523965	1.36E-07	1.89E-06	2.40	8.24	23.00	105.50
NUT	NUT0660	1.754477	0.0001094	0.0006634	2.16	7.39	21.25	99.00
AST	AS_YHL031C	1.754884	0.0001173	0.0007063	0.88	3.00	8.00	37.00
AST	AS_YNR058W	1.7556609	0.0002467	0.0013556	1.00	3.59	10.50	45.75
NUT	NUT1083	1.7569849	0.0001839	0.0010421	0.93	3.11	8.25	40.75
AST	AS_YGR049W	1.7590521	8.54E-06	7.04E-05	1.86	6.32	17.00	83.50
NUT	NUT0534	1.7603271	6.17E-05	0.0004002	0.87	2.85	7.50	37.75
NUT	NUT0094	1.7603401	0.0001808	0.001028	0.75	2.49	6.50	32.50
AST	AS_YML028W	1.761399	8.10E-06	6.72E-05	4.80	16.29	43.50	209.25
SUT	SUT252	1.7615774	6.61E-06	5.61E-05	1.70	5.80	15.75	74.75
NUT	NUT0212	1.7616867	0.0004503	0.002298	0.53	1.79	4.75	23.00
SRT	SRT129	1.7638098	0.000174	0.0009947	0.45	1.50	4.00	19.75
AST	AS_YNL239W	1.7639372	1.04E-09	2.67E-08	5.72	19.37	51.25	255.50
NUT	NUT0152	1.76457	1.35E-06	1.42E-05	2.73	9.16	24.00	123.00
CUT	CUT923	1.7647435	0.0002115	0.0011802	0.82	2.72	7.00	34.75
NUT	NUT0367	1.7649428	0.000524	0.0026341	0.46	1.51	4.00	20.25
AST	AS_YGL221C	1.7666694	8.15E-05	0.000509	0.79	2.68	7.25	36.25
AST	AS_YHR174W	1.7671018	0.0003715	0.0019442	1.08	3.70	10.00	48.75

Class	Transcript name	RRP6 KO_vs_WT log2_fold_change	RRP6 KO_vs_WT p-value	RRP6 KO_vs_WT FDR	Ave Norm Reads in WT	Ave Norm Reads in RRP6	Average RAW read counts in WT	Average RAW read counts in RRP6
ORF-T	YHR021W-A	1.7671216	0.0004096	0.0021181	0.50	1.55	4.00	20.75
NUT	NUT1402	1.7674013	4.89E-06	4.34E-05	1.70	5.77	15.50	76.50
NUT	NUT1142	1.7676624	0.0003034	0.0016168	0.50	1.81	5.00	23.25
NUT	NUT0438	1.7682786	4.19E-08	6.83E-07	6.00	20.50	54.75	253.50
NUT	NUT1493	1.7702353	1.19E-05	9.42E-05	1.29	4.45	12.00	58.75
CUT	CUT355	1.7715569	2.45E-07	3.13E-06	2.01	6.88	18.25	88.50
SUT	SUT834	1.7722742	2.02E-06	2.02E-05	2.72	9.19	23.75	123.75
SRT	SRT518	1.7729676	0.0060545	0.0228578	0.21	0.81	2.25	10.75
NUT	NUT0133	1.7751595	5.09E-07	5.99E-06	3.13	10.76	29.00	138.25
NUT	NUT0326	1.7761524	1.90E-07	2.51E-06	6.89	23.67	64.25	313.00
SUT	SUT153	1.7761543	1.91E-07	2.53E-06	6.89	23.67	64.25	313.00
AST	AS_YGR280C	1.778095	0.0066122	0.0246042	0.35	1.13	3.00	16.25
CUT	newCUT1	1.7793243	1.76E-06	1.79E-05	2.70	9.26	24.00	116.25
sn/snoRNA ET	SNR69-ET	1.779371	2.11E-08	3.77E-07	16.34	55.81	137.25	702.25
NUT	NUT1060	1.7799371	1.30E-05	0.0001011	1.45	5.03	13.75	68.50
CUT	CUT453	1.7799428	3.42E-05	0.000238	2.67	9.22	24.25	117.00
ORF-T	YPL114W	1.7802781	9.81E-06	7.93E-05	1.49	4.98	12.75	66.25
NUT	NUT0570	1.78208	0.0001526	0.0008931	0.94	3.30	9.00	44.25
ORF-T	YER158W-A	1.7823108	0.0010196	0.0047711	0.32	1.14	3.00	14.25
AST	AS_YIL083C	1.7826983	8.84E-06	7.26E-05	1.82	6.25	16.50	80.00
NUT	NUT0694	1.7832412	0.0023403	0.0100134	0.33	1.21	3.25	15.50
CUT	CUT430	1.7837098	0.001519	0.0068802	0.36	1.27	3.25	15.25
AST	AS_YGR237C	1.7850202	0.001261	0.005779	0.83	2.83	7.50	39.00
NUT	NUT0054	1.7857677	0.0026326	0.011103	0.31	1.07	2.75	13.25
CUT	CUT031	1.78577	0.0026339	0.0111038	0.31	1.07	2.75	13.25
CUT	CUT695	1.7869766	0.0001087	0.0006598	0.62	2.18	5.75	28.25
NUT	NUT0685	1.7869815	3.34E-06	3.11E-05	2.52	8.70	23.00	113.25
NUT	NUT0070	1.7879535	0.0012614	0.005779	0.64	2.09	5.25	29.25
NUT	NUT0192	1.788277	0.000364	0.0019078	0.41	1.37	3.50	17.25

TABLE S1: Differential expression data for RRP6 RNA-Seq dataset Page 294

Class	Transcript name	RRP6 KO_vs_WT log2_fold _change	RRP6 KO_vs_WT p-value	RRP6 KO_vs_WT FDR	Ave Norm Reads in WT	Ave Norm Reads in RRP6	Average RAW read counts in WT	Average RAW read counts in RRP6
NUT	NUT1301	1.7885489	2.85E-06	2.72E-05	1.59	5.51	14.75	74.00
NUT	NUT1373	1.7886077	0.0002994	0.0015984	3.23	11.32	32.50	147.00
AST	AS_YPL119C	1.7896159	0.0001582	0.0009192	0.56	1.91	5.00	25.50
CUT	CUT914	1.7903917	3.14E-05	0.0002207	0.76	2.64	7.00	34.75
ORF-T	YGL199C	1.7912766	3.51E-05	0.000243	1.17	4.04	10.50	51.00
NUT	NUT1439	1.7920772	0.0002808	0.0015151	0.49	1.82	5.00	24.00
CUT	CUT549	1.7925816	5.98E-05	0.0003906	8.55	29.66	77.00	367.00
AST	AS_YNL076W	1.7935811	0.0001046	0.000636	1.24	4.29	11.50	60.50
NUT	NUT0308	1.793773	4.64E-08	7.38E-07	3.97	13.68	35.25	174.75
AST	AS_YLR266C	1.7943693	1.81E-05	0.0001359	1.03	3.58	9.50	48.50
CUT	CUT700	1.7951257	1.37E-05	0.0001062	1.73	5.95	15.00	75.50
AST	AS_YBR146W	1.795404	0.0005314	0.0026687	0.40	1.36	3.50	17.75
NUT	NUT1226	1.7960633	0.0003774	0.0019707	0.73	2.56	6.75	32.00
NUT	NUT1183	1.7964591	1.08E-06	1.16E-05	1.99	6.93	18.50	94.75
NUT	NUT1412	1.7968406	3.49E-07	4.28E-06	1.73	5.96	15.50	78.00
AST	AS_YDR281C	1.7970043	0.0004827	0.0024473	0.65	2.38	6.50	31.75
NUT	NUT0111	1.7970296	0.0002673	0.0014525	0.45	1.66	4.50	22.00
NUT	NUT1364	1.7984787	0.0001011	0.0006172	1.24	4.30	11.50	60.75
sn/snoRNA ET	SNR84-ET	1.7992747	5.09E-07	5.98E-06	6.89	23.90	59.25	293.25
NUT	NUT0432	1.7996966	0.0016683	0.0074605	0.28	1.03	2.75	13.50
NUT	NUT0035	1.8008737	8.54E-05	0.00053	0.96	3.35	8.75	43.50
CUT	CUT607	1.8016728	4.91E-05	0.0003287	0.87	2.97	7.50	38.25
NUT	NUT0558	1.8025571	3.22E-08	5.42E-07	7.46	25.90	64.75	325.50
NUT	NUT0719	1.803213	0.0008242	0.0039539	0.53	1.96	5.50	28.25
SRT	SRT163	1.8032665	7.37E-05	0.0004678	0.58	2.07	5.50	28.00
CUT	CUT346	1.803618	4.16E-05	0.0002822	0.66	2.24	5.75	29.50
NUT	NUT0363	1.8038463	4.32E-08	6.99E-07	3.71	12.93	33.50	166.50
SUT	SUT110	1.8048479	9.72E-05	0.0005963	1.26	4.50	12.00	57.00
AST	AS_YGR284C	1.8050879	9.73E-06	7.87E-05	1.04	3.78	10.25	48.75

TABLE S1: Differential expression data for RRP6 RNA-Seq dataset Page 295

Class	Transcript name	RRP6 KO_vs_WT log2_fold _change	RRP6 KO_vs_WT p-value	RRP6 KO_vs_WT FDR	Ave Norm Reads in WT	Ave Norm Reads in RRP6	Average RAW read counts in WT	Average RAW read counts in RRP6
AST	AS_YIL124W	1.8063005	2.49E-06	2.43E-05	1.23	4.40	11.75	56.50
CUT	CUT581	1.8063942	1.55E-05	0.0001181	0.80	2.68	6.75	34.75
AST	AS_YGR011W	1.8077116	2.06E-05	0.0001519	0.86	2.94	7.50	38.00
NUT	NUT0618	1.8083957	4.66E-06	4.15E-05	0.99	3.42	8.75	44.00
SRT	SRT219	1.8087189	6.12E-06	5.26E-05	2.19	7.66	19.75	96.00
SUT	SUT312	1.808978	7.00E-06	5.91E-05	2.19	7.66	19.75	95.75
NUT	NUT1023	1.809947	7.66E-05	0.0004845	0.90	3.07	7.75	40.75
CUT	CUT251	1.8105751	3.74E-06	3.44E-05	1.92	6.76	17.25	84.50
ORF-T	YJL153C	1.8106032	0.0657102	0.1574296	0.69	2.43	6.25	28.00
SRT	SRT440	1.8110315	0.0300746	0.0859128	0.20	0.77	2.25	11.00
NUT	NUT1447	1.8118647	2.11E-07	2.74E-06	2.28	8.03	20.75	105.25
NUT	NUT1430	1.8134195	0.0001605	0.0009318	0.46	1.81	5.00	24.25
CUT	CUT824	1.8141213	0.00017	0.0009774	0.41	1.52	4.00	20.00
NUT	NUT0886	1.8141699	3.07E-05	0.0002163	1.36	4.83	13.00	68.25
AST	AS_YBR077C	1.8143202	0.0003565	0.0018711	0.60	2.13	5.50	27.25
NUT	NUT0561	1.8157008	0.000169	0.0009733	0.89	3.14	8.00	38.25
NUT	NUT0941	1.817246	0.0005964	0.0029474	0.38	1.35	3.50	17.75
AST	AS_YLL035W	1.81847	0.0007399	0.0035851	0.89	3.20	8.50	42.75
AST	AS_YHR183W	1.8189257	5.96E-05	0.00039	1.99	7.07	19.00	99.75
CUT	CUT924	1.8194027	0.0041017	0.0163549	0.20	0.77	2.00	10.00
AST	AS_YPR183W	1.8226882	6.46E-05	0.0004173	1.07	3.77	9.50	48.25
AST	AS_YDL036C	1.8228229	0.0011651	0.0053748	0.53	1.89	5.00	26.50
SRT	SRT200	1.8233438	1.82E-05	0.0001365	1.44	5.10	13.25	69.25
AST	AS_YMR021C	1.8236875	3.97E-06	3.62E-05	1.22	4.28	11.00	57.50
NUT	NUT1238	1.8238496	5.98E-06	5.15E-05	1.19	4.18	10.50	52.25
AST	AS_YIL142W	1.8254006	0.0002637	0.0014349	0.77	2.75	7.00	35.25
NUT	NUT0877	1.8258942	3.84E-05	0.0002633	4.00	14.31	39.25	190.00
NUT	NUT0442	1.8259927	0.0001407	0.0008314	0.50	1.81	4.75	24.25
AST	AS_YJL012C	1.8265787	0.0006855	0.0033377	0.43	1.46	3.75	20.25

Class	Transcript name	RRP6 KO_vs_WT log2_fold_change	RRP6 KO_vs_WT p-value	RRP6 KO_vs_WT FDR	Ave Norm Reads in WT	Ave Norm Reads in RRP6	Average RAW read counts in WT	Average RAW read counts in RRP6
AST	AS_YGR039W	1.8294966	5.97E-05	0.0003905	0.87	3.02	7.50	38.75
AST	AS_YFR051C	1.829824	0.0005742	0.0028536	0.81	2.91	7.50	39.50
NUT	NUT0662	1.8299964	3.26E-06	3.05E-05	1.58	5.71	14.75	71.25
SRT	SRT364	1.8315135	0.0010793	0.0050246	0.40	1.46	3.75	19.50
NUT	NUT0390	1.8325726	5.73E-10	1.60E-08	4.26	15.27	39.75	197.75
NUT	NUT1050	1.8326777	4.18E-07	5.03E-06	7.68	27.28	68.25	345.25
AST	AS_YJR033C	1.8337127	5.75E-06	4.97E-05	1.68	6.08	16.25	80.75
SUT	SUT006	1.8339043	3.08E-05	0.0002163	0.75	2.61	6.50	34.25
NUT	NUT0015	1.8339171	3.07E-05	0.000216	0.75	2.61	6.50	34.25
NUT	NUT1132	1.8349516	0.000247	0.0013565	0.77	2.77	7.00	35.50
NUT	NUT1082	1.8363311	3.08E-06	2.91E-05	2.22	7.89	19.75	101.50
NUT	NUT1119	1.8364397	1.91E-05	0.0001418	0.79	2.86	7.50	39.00
NUT	NUT1368	1.8371916	7.58E-10	2.02E-08	8.24	29.56	77.25	370.00
NUT	NUT1273	1.8375131	4.37E-08	7.02E-07	3.74	13.34	33.75	172.75
CUT	CUT611	1.8386556	0.0005622	0.0027993	0.41	1.39	3.50	19.00
NUT	NUT0551	1.8387461	1.08E-07	1.55E-06	2.09	7.51	19.25	96.75
AST	AS_YER088C-A	1.8405262	7.03E-05	0.0004495	0.58	2.05	5.25	27.50
SUT	SUT011	1.8406491	8.97E-05	0.0005546	0.53	1.95	5.00	24.75
NUT	NUT1079	1.8419982	0.0009795	0.0046089	0.40	1.38	3.50	19.50
ORF-T	YLL037W	1.8430839	0.0030085	0.0124797	0.25	0.96	2.50	12.50
AST	AS_YLR410W	1.8442628	1.21E-08	2.35E-07	5.05	18.03	45.25	237.25
NUT	NUT0449	1.8453199	4.50E-05	0.0003034	0.58	2.12	5.50	29.00
NUT	NUT1277	1.8462248	1.18E-08	2.31E-07	5.05	18.06	45.25	237.50
NUT	NUT1333	1.8476058	0.0002041	0.001144	0.76	2.81	7.25	35.00
AST	AS_YNL236W	1.8484065	9.31E-05	0.0005736	1.90	6.87	17.50	90.00
AST	AS_YIL043C	1.8496149	0.0003363	0.0017748	0.43	1.49	3.75	20.00
CUT	CUT548	1.8497465	0.0005135	0.0025914	0.78	2.90	7.50	36.50
AST	AS_YOR124C	1.8500309	0.0006217	0.0030553	0.52	1.94	5.25	28.00
CUT	CUT311	1.8506197	1.47E-06	1.53E-05	1.55	5.54	14.00	73.00

Class	Transcript name	RRP6 KO_vs_WT log2_fold _change	RRP6 KO_vs_WT p-value	RRP6 KO_vs_WT FDR	Ave Norm Reads in WT	Ave Norm Reads in RRP6	Average RAW read counts in WT	Average RAW read counts in RRP6
NUT	NUT0415	1.8514578	5.57E-06	4.84E-05	1.68	6.16	16.25	82.00
NUT	NUT0482	1.8518807	0.0001913	0.0010793	0.51	1.87	4.75	25.00
AST	AS_YOR229W	1.8522435	0.0001473	0.0008661	0.72	2.51	6.25	35.25
CUT	CUT017	1.8529404	5.31E-07	6.20E-06	2.22	7.89	19.25	104.50
SRT	SRT542	1.8535695	6.71E-05	0.0004313	0.51	1.87	4.75	24.75
AST	AS_YBR029C	1.8540396	0.0005012	0.0025372	1.14	3.98	9.25	53.25
NUT	NUT0027	1.8542612	7.91E-08	1.18E-06	3.04	10.86	26.50	142.75
AST	AS_YPR117W	1.8545232	4.03E-06	3.67E-05	1.36	5.03	13.25	67.75
sn/snoRNA ET	SNR80-ET	1.8553287	1.16E-08	2.28E-07	17.07	61.72	152.50	782.00
AST	AS_YLR047C	1.8553525	0.0005632	0.0028018	1.57	5.88	16.75	80.25
AST	AS_YHR036W	1.856852	7.74E-05	0.0004887	0.74	2.62	6.50	35.00
NUT	NUT0416	1.8573135	6.50E-06	5.54E-05	1.26	4.59	11.50	57.75
CUT	CUT481	1.8583411	5.87E-05	0.0003856	0.99	3.57	8.75	46.00
NUT	NUT0307	1.8585985	8.88E-07	9.82E-06	2.05	7.48	19.00	99.75
CUT	CUT861	1.8587492	1.45E-05	0.0001112	1.50	5.37	13.00	70.00
SUT	SUT285	1.8606372	1.47E-08	2.78E-07	6.87	24.82	59.75	312.50
AST	AS_YJL016W	1.8617949	4.73E-07	5.61E-06	1.87	6.84	17.50	93.25
AST	AS_YLR109W	1.8626876	9.02E-06	7.40E-05	0.66	2.39	6.00	31.50
CUT	CUT815	1.8635077	6.06E-05	0.0003951	1.14	4.16	10.75	58.75
SUT	SUT749	1.8648702	4.65E-10	1.32E-08	7.89	28.87	74.00	361.50
NUT	NUT1432	1.8652491	2.76E-06	2.65E-05	1.00	3.54	8.75	47.75
AST	AS_YDR352W	1.8654592	8.30E-07	9.25E-06	1.65	5.96	14.50	74.50
NUT	NUT1302	1.8654787	0.0001933	0.0010887	0.35	1.37	3.50	18.00
CUT	CUT759	1.8662807	1.84E-05	0.0001376	0.84	2.97	7.25	39.25
NUT	NUT0702	1.8662906	0.0004766	0.0024201	0.45	1.48	3.50	19.25
NUT	NUT0247	1.8663519	0.0008408	0.0040297	0.29	1.08	2.75	14.50
NUT	NUT1102	1.8674147	4.37E-09	9.54E-08	3.58	12.94	31.50	167.50
AST	AS_YLR131C	1.8684199	0.0001553	0.0009049	1.27	4.68	12.00	60.75
NUT	NUT0513	1.8685032	0.0001619	0.0009385	1.27	4.68	12.00	60.75

Class	Transcript name	RRP6 KO_vs_WT log2_fold _change	RRP6 KO_vs_WT p-value	RRP6 KO_vs_WT FDR	Ave Norm Reads in WT	Ave Norm Reads in RRP6	Average RAW read counts in WT	Average RAW read counts in RRP6
NUT	NUT0392	1.8693578	2.20E-07	2.86E-06	2.31	8.42	20.75	108.50
CUT	CUT167	1.8693674	8.52E-07	9.46E-06	2.03	7.45	18.75	99.50
AST	AS_YJL044C	1.869387	0.0001129	0.0006815	0.80	3.02	7.75	39.75
SRT	SRT116	1.8719978	9.04E-05	0.0005584	0.66	2.39	5.75	29.50
NUT	NUT1468	1.8729742	3.38E-05	0.0002356	0.63	2.37	6.00	31.00
SRT	SRT549	1.8736138	5.68E-05	0.0003745	1.14	4.18	10.75	59.25
SRT	SRT595	1.8743916	0.0020081	0.0087773	0.35	1.21	3.00	16.75
AST	AS_YPL267W	1.8777029	0.0002471	0.0013565	0.66	2.32	5.50	31.25
NUT	NUT0285	1.8783253	5.52E-05	0.000365	0.99	3.55	8.50	47.50
AST	AS_YDR074W	1.8788762	0.0006413	0.0031392	0.70	2.70	7.25	36.75
sn/snoRNA	SNR42-A	1.8788886	2.73E-08	4.71E-07	47.47	174.43	403.75	2177.50
SUT	SUT576	1.8812215	0.0003051	0.0016246	0.83	3.11	7.75	41.00
NUT	NUT1389	1.8821983	9.30E-07	1.02E-05	0.95	3.53	8.75	45.50
AST	AS_YPL063W	1.8828345	0.0001159	0.0006982	1.32	4.96	12.50	62.50
NUT	NUT0674	1.8839886	1.26E-07	1.78E-06	2.24	8.28	20.50	109.00
AST	AS_YGL174W	1.8854708	5.98E-05	0.0003906	0.56	2.08	5.25	28.00
NUT	NUT0543	1.8865957	0.0026125	0.0110274	0.41	1.53	3.75	19.00
AST	AS_YOR202W	1.8881617	4.90E-06	4.34E-05	0.89	3.18	7.75	43.00
AST	AS_YPL171C	1.8896774	1.55E-05	0.0001181	0.56	2.11	5.25	28.25
CUT	CUT742	1.8903645	5.15E-08	8.12E-07	2.31	8.53	20.75	111.50
SRT	SRT342	1.8906518	1.30E-05	0.0001011	0.80	3.04	7.50	38.50
SUT	SUT829	1.8915503	2.25E-10	6.93E-09	6.84	25.55	65.00	330.00
AST	AS_YGR268C	1.8938942	1.48E-08	2.79E-07	3.38	12.52	30.25	160.50
NUT	NUT1185	1.8940817	0.0013271	0.0060609	0.51	1.97	5.25	29.25
ORF-T	YJL015C	1.8944421	9.84E-07	1.08E-05	1.43	5.34	13.25	72.25
AST	AS_YKL165C	1.894583	0.0009084	0.0043211	0.27	1.03	2.50	13.00
CUT	CUT706	1.8950184	0.0008828	0.0042049	0.41	1.55	3.75	19.50
CUT	CUT592	1.8951762	7.04E-05	0.0004497	0.68	2.44	5.75	32.25
CUT	CUT605	1.8955207	5.92E-05	0.0003884	4.52	16.81	40.75	208.00

TABLE S1: Differential expression data for RRP6 RNA-Seq dataset Page 299

Class	Transcript name	RRP6 KO_vs_WT log2_fold _change	RRP6 KO_vs_WT p-value	RRP6 KO_vs_WT FDR	Ave Norm Reads in WT	Ave Norm Reads in RRP6	Average RAW read counts in WT	Average RAW read counts in RRP6
NUT	NUT0956	1.895627	1.21E-05	9.51E-05	0.68	2.55	6.25	33.50
AST	AS_YFR034C	1.8960244	1.85E-05	0.0001378	1.07	4.12	10.50	52.50
CUT	CUT184	1.8984001	0.0003738	0.0019539	0.68	2.53	6.25	33.75
SUT	SUT586	1.8991853	3.72E-05	0.0002562	0.77	2.86	7.00	37.75
NUT	NUT1357	1.899264	0.000305	0.0016246	0.35	1.40	3.50	18.00
NUT	NUT0243	1.8999013	3.26E-05	0.0002284	1.26	4.81	12.00	61.00
NUT	NUT0263	1.9007777	0.0008825	0.0042049	0.40	1.43	3.50	20.00
NUT	NUT0697	1.9014286	1.76E-06	1.79E-05	2.02	7.46	17.75	96.50
AST	AS_YKL069W	1.9032289	0.0003784	0.0019749	0.33	1.32	3.25	16.75
AST	AS_YML029W	1.9034956	1.17E-11	5.22E-10	18.97	71.06	174.75	916.00
SRT	SRT91	1.9040334	0.0025535	0.0107983	0.26	0.99	2.50	13.75
NUT	NUT1291	1.904852	1.65E-11	6.98E-10	19.04	71.38	175.25	920.75
NUT	NUT0029	1.9053842	1.62E-05	0.0001225	0.86	3.30	8.25	44.00
CUT	CUT019	1.9053885	1.62E-05	0.0001226	0.86	3.30	8.25	44.00
AST	AS_YDR117C	1.9070776	9.11E-06	7.46E-05	1.15	4.36	10.75	58.50
NUT	NUT0778	1.907819	5.22E-05	0.0003465	0.67	2.44	5.75	31.50
NUT	NUT1044	1.9082185	0.0030626	0.0126727	0.53	2.13	5.75	30.00
NUT	NUT1312	1.9089617	1.82E-06	1.84E-05	1.96	7.46	18.75	97.25
AST	AS_YGL027C	1.9091113	0.000979	0.0046089	0.38	1.35	3.25	18.75
AST	AS_YNR018W	1.9097318	0.000173	0.0009902	2.51	9.62	25.75	125.00
NUT	NUT1148	1.9098848	2.47E-05	0.0001782	0.60	2.17	5.25	30.00
CUT	CUT652	1.9100628	0.0009475	0.0044814	0.66	2.55	6.25	32.75
AST	AS_YML114C	1.9124588	0.0001329	0.0007904	0.78	2.89	6.75	35.25
NUT	NUT0977	1.9132791	7.94E-08	1.19E-06	3.31	12.58	31.50	167.25
AST	AS_YLR330W	1.9138305	0.0001276	0.000761	0.55	2.15	5.25	27.00
ORF-T	YHL030W-A	1.9144937	0.0002704	0.0014674	0.40	1.50	3.50	18.50
NUT	NUT0296	1.9150317	1.83E-05	0.0001373	1.60	5.98	14.00	77.25
NUT	NUT0816	1.9156809	6.84E-05	0.0004387	0.68	2.56	6.00	32.00
CUT	CUT133	1.9177127	0.0023024	0.0098838	0.25	0.91	2.25	13.25

TABLE S1: Differential expression data for RRP6 RNA-Seq dataset Page 300

Class	Transcript name	RRP6 KO_vs_WT log2_fold _change	RRP6 KO_vs_WT p-value	RRP6 KO_vs_WT FDR	Ave Norm Reads in WT	Ave Norm Reads in RRP6	Average RAW read counts in WT	Average RAW read counts in RRP6
CUT	CUT071	1.9184207	1.67E-07	2.25E-06	1.88	7.14	17.25	91.00
AST	AS_YDR323C	1.9187905	0.000251	0.0013754	0.49	1.87	4.50	23.75
AST	AS_YOL084W	1.9196919	4.92E-05	0.000329	0.60	2.15	5.00	28.25
AST	AS_YDL040C	1.9208972	0.0063577	0.0237887	0.28	1.05	2.50	14.00
NUT	NUT0989	1.9215861	4.03E-06	3.67E-05	1.20	4.58	11.00	58.75
AST	AS_YIL133C	1.9218071	0.005542	0.0212055	0.24	1.00	2.50	12.75
NUT	NUT1419	1.9225385	2.24E-06	2.21E-05	1.21	4.54	10.75	59.50
NUT	NUT1513	1.9229077	1.78E-06	1.80E-05	1.27	4.90	12.25	65.75
SUT	SUT383	1.9239731	9.30E-05	0.0005734	0.88	3.18	7.25	42.75
AST	AS_YDL124W	1.9240472	1.27E-05	9.91E-05	1.21	4.64	11.50	65.75
NUT	NUT0268	1.9244376	1.32E-05	0.0001024	0.72	2.87	7.25	39.00
CUT	CUT279	1.9249636	2.30E-05	0.000168	0.52	1.89	4.50	25.25
NUT	NUT1500	1.9255883	9.99E-11	3.47E-09	7.64	29.16	71.75	378.50
CUT	CUT435	1.9272274	7.93E-05	0.0004983	0.63	2.36	5.50	29.50
AST	AS_YPR200C	1.9272328	7.89E-05	0.0004972	0.63	2.36	5.50	29.50
AST	AS_YNL023C	1.9276661	1.95E-06	1.95E-05	1.44	5.57	13.50	69.25
CUT	CUT108	1.9279314	3.98E-06	3.64E-05	1.60	6.01	13.75	73.50
AST	AS_YBR260C	1.9279321	7.08E-05	0.0004517	0.55	2.08	5.00	28.00
CUT	CUT145	1.9285513	0.0033269	0.0136431	0.60	2.29	5.25	27.75
AST	AS_YKL193C	1.9286193	0.0002855	0.0015369	0.43	1.66	4.00	22.25
AST	AS_YNL053W	1.9288524	1.33E-10	4.40E-09	7.45	28.54	70.25	357.00
CUT	CUT021	1.9291167	2.31E-05	0.0001685	1.35	5.10	12.00	69.00
AST	AS_YDL100C	1.9305108	0.0001795	0.0010214	0.40	1.58	3.75	19.75
CUT	CUT148	1.9307985	5.00E-07	5.90E-06	2.84	10.74	25.00	140.75
CUT	CUT189	1.9326194	1.98E-07	2.60E-06	1.81	6.83	16.00	88.25
AST	AS_YLR398C	1.9339195	0.0002534	0.0013875	0.37	1.37	3.25	18.25
CUT	CUT877	1.935139	2.61E-05	0.0001868	0.65	2.45	5.75	32.00
NUT	NUT0467	1.9364959	3.76E-06	3.46E-05	1.92	7.29	16.75	95.50
NUT	NUT0500	1.9385081	0.0001537	0.0008975	1.79	7.09	19.00	97.50

Class	Transcript name	RRP6 KO_vs_WT log2_fold _change	RRP6 KO_vs_WT p-value	RRP6 KO_vs_WT FDR	Ave Norm Reads in WT	Ave Norm Reads in RRP6	Average RAW read counts in WT	Average RAW read counts in RRP6
AST	AS_YOR295W	1.9398089	2.75E-05	0.0001953	0.52	2.00	4.75	26.00
AST	AS_YGR019W	1.9408175	3.50E-06	3.25E-05	5.00	19.17	45.00	242.50
AST	AS_YBR062C	1.9423831	1.90E-05	0.0001412	0.80	3.11	7.50	41.50
AST	AS_YHR098C	1.9439934	7.91E-05	0.0004977	1.22	4.68	10.75	60.25
AST	AS_YJR106W	1.9440645	0.0002659	0.0014462	0.43	1.77	4.50	25.00
NUT	NUT0077	1.9442601	1.01E-05	8.15E-05	0.94	3.74	9.00	46.50
AST	AS_YDR246W	1.9470193	2.07E-06	2.07E-05	0.75	2.73	6.25	35.75
SUT	SUT583	1.9477491	2.39E-08	4.19E-07	2.19	8.34	19.25	109.00
NUT	NUT0832	1.949448	0.0003486	0.0018325	0.34	1.23	2.75	15.25
NUT	NUT0909	1.9494803	3.27E-05	0.0002291	1.41	5.64	14.00	67.50
AST	AS_YOR329C	1.9495889	3.71E-05	0.0002557	0.99	3.71	8.50	49.75
AST	AS_YJL137C	1.9500216	1.68E-07	2.27E-06	2.11	8.11	18.75	104.50
AST	AS_YDL197C	1.9506252	3.78E-05	0.0002593	0.63	2.39	5.50	31.25
AST	AS_YCR097W	1.9514549	5.78E-05	0.0003804	0.82	3.38	8.50	43.50
AST	AS_YPR036W	1.9519331	9.74E-11	3.40E-09	7.19	27.96	68.00	363.50
CUT	CUT204	1.9524442	2.38E-07	3.05E-06	2.02	7.78	18.00	100.00
CUT	CUT661	1.9547807	1.13E-06	1.21E-05	2.47	9.38	20.75	125.50
CUT	CUT686	1.9564772	8.18E-10	2.18E-08	5.53	21.32	48.75	281.50
AST	AS_YKL198C	1.9570834	5.84E-08	9.08E-07	6.66	25.96	60.75	314.50
CUT	CUT524	1.9573871	7.10E-07	8.06E-06	1.58	6.08	13.75	75.75
NUT	NUT0235	1.9582182	2.62E-07	3.32E-06	1.40	5.48	13.00	71.00
CUT	CUT286	1.9584644	0.0083084	0.0295945	0.19	0.67	1.50	9.00
NUT	NUT1128	1.9619927	0.0119181	0.0402758	0.30	1.20	3.00	17.50
SUT	SUT182	1.96367	9.08E-07	1.00E-05	0.81	3.12	7.25	40.75
NUT	NUT1106	1.9640793	1.74E-06	1.77E-05	1.16	4.45	10.25	58.75
SUT	SUT371	1.9646443	0.0002622	0.0014286	0.91	3.46	7.50	43.25
AST	AS_YER028C	1.9651341	6.84E-08	1.04E-06	1.98	7.60	17.00	94.75
AST	AS_YGR015C	1.9656709	1.36E-05	0.0001053	0.67	2.64	6.25	35.75
NUT	NUT0269	1.9656717	1.36E-05	0.0001053	0.67	2.64	6.25	35.75

Class	Transcript name	RRP6 KO_vs_WT log2_fold_change	RRP6 KO_vs_WT p-value	RRP6 KO_vs_WT FDR	Ave Norm Reads in WT	Ave Norm Reads in RRP6	Average RAW read counts in WT	Average RAW read counts in RRP6
SUT	SUT222	1.9675484	1.23E-08	2.38E-07	4.19	16.46	38.25	203.50
AST	AS_YJL089W	1.9692296	3.50E-05	0.0002423	0.57	2.23	5.25	30.00
NUT	NUT0341	1.9696638	0.0001266	0.0007561	0.33	1.21	2.75	15.75
ORF-T	YOR186C-A	1.9725859	2.26E-08	3.97E-07	2.83	10.95	24.50	139.25
SRT	SRT431	1.9727974	1.15E-11	5.17E-10	5.45	21.48	50.25	280.25
NUT	NUT1338	1.972837	0.0031894	0.013138	0.22	1.00	2.50	13.25
AST	AS_YNL230C	1.9737466	3.10E-07	3.87E-06	1.42	5.45	12.25	71.50
CUT	CUT038	1.9744519	0.0002568	0.0014029	0.30	1.16	2.75	15.75
AST	AS_YNL205C	1.974613	0.0001539	0.0008983	0.42	1.71	4.00	21.25
NUT	NUT1458	1.9759366	0.0070257	0.0258472	0.24	0.90	2.00	11.75
NUT	NUT1268	1.9775481	0.0001395	0.0008253	1.20	4.81	11.25	60.50
AST	AS_YGR088W	1.9795372	2.26E-06	2.23E-05	1.05	4.21	10.00	57.75
NUT	NUT0774	1.9801935	3.82E-07	4.65E-06	1.27	4.88	11.00	65.50
AST	AS_YPL231W	1.9804414	1.79E-05	0.0001346	0.87	3.44	7.75	43.25
AST	AS_YHR070W	1.9810115	9.69E-12	4.46E-10	5.38	21.29	49.50	278.00
SUT	SUT159	1.981303	0.000771	0.0037219	0.39	1.49	3.50	21.50
CUT	CUT771	1.9824638	1.45E-06	1.52E-05	0.80	3.23	7.50	41.25
CUT	CUT275	1.9839467	2.48E-05	0.0001785	0.68	2.86	6.75	35.25
AST	AS_YER044C-A	1.9855147	2.69E-06	2.59E-05	0.79	3.08	7.00	40.75
NUT	NUT1026	1.9857184	6.72E-06	5.69E-05	1.13	4.44	10.00	58.50
AST	AS_YNR051C	1.9873378	0.0024596	0.0104431	0.27	1.08	2.50	14.50
AST	AS_YIL084C	1.9887861	1.26E-07	1.78E-06	1.01	4.02	9.25	52.50
NUT	NUT0539	1.9889088	2.93E-06	2.79E-05	1.97	7.80	17.75	101.25
NUT	NUT1048	1.9905922	3.93E-09	8.73E-08	2.78	11.11	25.75	145.50
NUT	NUT0294	1.9907823	6.16E-05	0.0003998	0.53	2.08	4.75	27.75
NUT	NUT0630	1.991258	2.42E-05	0.0001753	0.52	2.16	5.00	27.50
AST	AS_YMR291W	1.9917972	0.0001809	0.001028	0.73	2.99	7.25	41.25
CUT	CUT469	1.9921333	0.000177	0.0010087	0.43	1.73	4.00	23.00
AST	AS_YGR292W	1.994223	3.58E-06	3.31E-05	0.94	3.75	8.75	50.25

Class	Transcript name	RRP6 KO_vs_WT log2_fold _change	RRP6 KO_vs_WT p-value	RRP6 KO_vs_WT FDR	Ave Norm Reads in WT	Ave Norm Reads in RRP6	Average RAW read counts in WT	Average RAW read counts in RRP6
NUT	NUT1397	1.9957771	1.98E-05	0.0001464	1.87	7.47	17.00	98.25
SRT	SRT577	1.9975268	0.007692	0.0278415	0.24	0.92	2.00	12.00
AST	AS_YDL144C	2.0003382	1.66E-07	2.24E-06	1.17	4.67	10.75	62.75
CUT	CUT356	2.0008498	0.0046226	0.0181239	1.57	6.31	13.75	77.75
NUT	NUT0916	2.0019133	0.0016242	0.0072859	0.26	1.10	2.50	14.00
NUT	NUT0636	2.0023886	1.63E-07	2.22E-06	1.35	5.30	11.75	69.50
AST	AS_YCR042C	2.0030969	3.29E-07	4.07E-06	2.57	10.13	21.75	127.25
NUT	NUT1055	2.0032266	2.98E-07	3.72E-06	2.15	8.57	19.00	110.25
NUT	NUT0679	2.0041885	2.25E-05	0.0001648	0.80	3.26	7.50	43.00
SUT	SUT037	2.0043093	2.84E-07	3.56E-06	2.83	11.31	25.50	151.00
AST	AS_YPR034W	2.0047561	0.0015618	0.0070465	0.27	1.01	2.25	14.00
AST	AS_YDR119W	2.0049761	8.50E-06	7.02E-05	0.94	3.70	8.25	50.00
AST	AS_YMR274C	2.005039	1.20E-06	1.28E-05	0.79	3.21	7.25	41.00
AST	AS_YDR169C-A	2.0051048	9.13E-06	7.47E-05	0.57	2.47	5.75	31.00
AST	AS_YIL049W	2.0064035	3.20E-06	3.00E-05	0.59	2.48	5.75	32.25
AST	AS_YCR059C	2.0074106	2.82E-07	3.55E-06	2.83	11.33	25.50	151.25
AST	AS_YJL153C	2.0079928	1.39E-10	4.61E-09	3.39	13.67	31.25	177.25
AST	AS_YHR051W	2.0087911	0.0002141	0.0011937	0.61	2.50	5.75	33.75
CUT	CUT065	2.0088605	2.80E-07	3.53E-06	1.65	6.50	14.25	85.75
NUT	NUT0819	2.0094518	8.50E-07	9.46E-06	2.13	8.68	20.25	112.00
CUT	CUT370	2.0097485	0.0008115	0.0038987	0.30	1.06	2.25	13.75
SUT	SUT506	2.0105681	8.68E-08	1.28E-06	6.81	27.52	62.25	348.50
AST	AS_YMR037C	2.0116168	0.0001871	0.0010582	1.08	4.56	11.50	62.25
NUT	NUT1054	2.0119411	1.44E-06	1.51E-05	0.76	3.26	7.75	43.25
AST	AS_YDR063W	2.0121007	0.0002448	0.0013473	0.61	2.50	5.75	33.25
NUT	NUT1509	2.0122961	7.26E-06	6.11E-05	0.65	2.82	6.75	37.50
NUT	NUT0750	2.0124181	2.26E-07	2.92E-06	1.22	4.95	11.25	64.25
ORF-T	YBR054W	2.0126424	5.06E-08	7.99E-07	62.71	252.81	537.75	3329.25
NUT	NUT1482	2.0127656	1.08E-06	1.16E-05	1.15	4.61	10.25	58.00

Class	Transcript name	RRP6 KO_vs_WT log2_fold _change	RRP6 KO_vs_WT p-value	RRP6 KO_vs_WT FDR	Ave Norm Reads in WT	Ave Norm Reads in RRP6	Average RAW read counts in WT	Average RAW read counts in RRP6
AST	AS_YLR137W	2.0128397	5.01E-06	4.43E-05	0.59	2.43	5.50	31.50
AST	AS_YLR195C	2.0142776	2.96E-07	3.71E-06	4.47	18.13	41.75	244.00
NUT	NUT0529	2.0150034	0.000511	0.0025815	0.35	1.49	3.50	19.75
CUT	CUT476	2.0154419	4.81E-05	0.0003228	1.10	4.53	10.25	55.50
AST	AS_YPR115W	2.0155652	2.33E-05	0.0001695	0.66	2.69	6.25	36.50
AST	AS_YLR216C	2.0164316	5.98E-06	5.15E-05	1.12	4.62	10.75	59.75
SUT	SUT079	2.0168313	1.65E-06	1.69E-05	1.50	6.03	13.50	79.50
AST	AS_YCR081W	2.0170413	1.65E-07	2.24E-06	2.25	9.09	20.50	120.75
NUT	NUT0085	2.0195714	3.16E-07	3.93E-06	2.76	11.16	25.00	149.00
CUT	CUT309	2.0199009	1.82E-07	2.42E-06	1.37	5.62	13.00	75.25
SRT	SRT71	2.0202006	0.0001977	0.0011114	0.88	3.65	8.50	45.00
ORF-T	YPL165C	2.0206982	2.63E-05	0.0001881	1.03	4.29	10.25	59.00
AST	AS_YDR126W	2.0216123	2.17E-06	2.15E-05	1.25	4.96	11.00	68.00
CUT	CUT197	2.0223992	2.02E-10	6.35E-09	2.98	12.17	27.25	155.75
NUT	NUT1009	2.0227348	0.0001683	0.0009701	0.35	1.37	3.00	17.75
NUT	NUT0963	2.0233887	6.84E-08	1.04E-06	7.43	30.31	68.50	385.50
NUT	NUT1233	2.0240491	1.59E-05	0.0001209	0.44	2.03	4.75	26.00
NUT	NUT1085	2.0250425	2.99E-06	2.84E-05	0.94	3.83	8.75	51.50
AST	AS_YDR092W	2.0264232	2.82E-06	2.70E-05	5.74	23.50	54.25	314.75
NUT	NUT0344	2.0283214	7.42E-14	5.94E-12	9.10	36.93	80.50	481.50
AST	AS_YER044C	2.0288198	0.0001676	0.0009669	0.30	1.26	2.75	15.75
AST	AS_YER157W	2.030005	0.0002155	0.0012009	0.26	1.11	2.50	14.75
AST	AS_YER091C	2.0310966	0.0019259	0.0084842	1.03	4.29	10.50	63.25
CUT	CUT047	2.0311949	0.0004354	0.0022289	0.27	1.20	2.75	15.75
CUT	CUT183	2.0313782	1.31E-07	1.84E-06	1.84	7.59	17.25	99.50
AST	AS_YKL182W	2.0319347	6.26E-07	7.20E-06	1.98	8.02	17.50	105.75
AST	AS_YPL233W	2.0321074	0.0003146	0.0016697	0.36	1.36	3.00	19.25
AST	AS_YOR186W	2.0322042	2.87E-09	6.59E-08	3.37	13.60	29.00	173.25
AST	AS_YIL032C	2.0323453	2.91E-05	0.0002059	0.70	3.03	7.25	40.00

TABLE S1: Differential expression data for RRP6 RNA-Seq dataset Page 305

Class	Transcript name	RRP6 KO_vs_WT log2_fold _change	RRP6 KO_vs_WT p-value	RRP6 KO_vs_WT FDR	Ave Norm Reads in WT	Ave Norm Reads in RRP6	Average RAW read counts in WT	Average RAW read counts in RRP6
NUT	NUT0170	2.032624	5.31E-07	6.20E-06	0.82	3.33	7.50	44.75
AST	AS_YOR371C	2.0342934	1.24E-07	1.75E-06	1.11	4.52	10.00	58.50
NUT	NUT0524	2.0344566	2.35E-07	3.03E-06	4.43	18.19	41.25	245.00
CUT	CUT310	2.0350737	5.93E-07	6.87E-06	0.82	3.39	7.50	43.50
AST	AS_YCR082W	2.0356165	0.0002076	0.0011618	0.29	1.32	3.00	17.00
CUT	CUT383	2.0363949	3.50E-08	5.82E-07	1.86	7.50	16.25	97.75
CUT	CUT662	2.0395803	0.0020908	0.009084	0.78	3.14	6.75	43.25
NUT	NUT0868	2.0396402	3.24E-09	7.35E-08	2.55	10.51	23.50	139.25
NUT	NUT0132	2.0401665	4.98E-07	5.88E-06	2.04	8.39	18.50	107.75
AST	AS_YDR144C	2.0419519	4.65E-07	5.54E-06	2.01	8.28	18.25	106.50
AST	AS_YJL164C	2.0421694	7.78E-11	2.80E-09	2.98	12.28	27.00	158.75
NUT	NUT0611	2.0428439	1.75E-07	2.35E-06	1.34	5.59	12.75	75.00
CUT	CUT805	2.0434837	0.007425	0.0270694	0.14	0.60	1.25	7.00
AST	AS_YDR234W	2.0437776	1.71E-07	2.30E-06	0.87	3.63	8.25	49.25
AST	AS_YLL036C	2.045162	3.65E-06	3.36E-05	1.10	4.51	10.00	61.25
NUT	NUT1103	2.0461234	6.67E-14	5.51E-12	8.95	36.76	79.00	479.50
AST	AS_YNL045W	2.0462156	1.14E-05	9.06E-05	3.63	15.14	36.00	207.75
CUT	CUT868	2.0462767	0.0090575	0.0318798	0.18	0.70	1.50	9.00
NUT	NUT1173	2.0469883	6.06E-11	2.26E-09	6.91	28.43	61.25	371.50
CUT	CUT656	2.0487133	6.53E-06	5.56E-05	0.86	3.51	7.75	48.25
NUT	NUT1191	2.0493455	2.28E-06	2.24E-05	1.54	6.31	13.75	86.50
CUT	CUT649	2.0500323	9.56E-05	0.0005884	0.36	1.52	3.25	18.50
CUT	CUT630	2.0511197	1.64E-06	1.69E-05	0.88	3.71	8.25	48.75
SUT	SUT164	2.051352	7.14E-14	5.76E-12	8.78	36.20	77.50	472.25
NUT	NUT0339	2.0516743	7.54E-06	6.33E-05	1.11	4.61	10.50	64.00
CUT	CUT493	2.0518894	0.0002208	0.001229	0.35	1.42	3.00	17.75
NUT	NUT0861	2.0519464	1.59E-06	1.64E-05	2.42	9.98	21.25	128.50
AST	AS_YMR202W	2.0540106	7.14E-06	6.02E-05	1.10	4.56	10.00	63.50
NUT	NUT0912	2.0541902	1.52E-06	1.58E-05	1.21	4.95	10.75	67.75

Class	Transcript name	RRP6 KO_vs_WT log2_fold _change	RRP6 KO_vs_WT p-value	RRP6 KO_vs_WT FDR	Ave Norm Reads in WT	Ave Norm Reads in RRP6	Average RAW read counts in WT	Average RAW read counts in RRP6
CUT	CUT028	2.0542196	0.0019354	0.0085099	0.20	0.80	1.75	11.00
CUT	CUT039	2.0549	2.49E-05	0.0001794	0.80	3.29	7.00	42.50
SRT	SRT49	2.0557113	6.39E-06	5.46E-05	1.13	4.66	10.00	60.75
CUT	CUT006	2.0576459	0.0016325	0.00732	0.21	0.92	2.00	11.75
AST	AS_YIL045W	2.05802	0.0001914	0.0010795	0.26	1.20	2.75	16.25
CUT	CUT398	2.0581639	5.20E-05	0.0003455	0.38	1.61	3.50	20.50
NUT	NUT0655	2.0590571	5.14E-06	4.53E-05	0.85	3.48	7.50	46.25
NUT	NUT0926	2.0610832	0.0006212	0.0030542	0.63	2.74	6.25	35.00
AST	AS_YJL083W	2.061192	6.64E-11	2.43E-09	6.66	27.66	59.00	362.00
sn/snoRNA ET	SNR38*RT	2.0613722	1.77E-09	4.33E-08	31.20	130.02	266.50	1579.75
SRT	SRT540	2.0637406	2.89E-05	0.0002049	0.59	2.58	6.00	35.75
AST	AS_YML006C	2.0639792	0.0008659	0.0041323	0.24	1.18	2.75	15.50
AST	AS_YGR040W	2.0647785	1.02E-06	1.11E-05	1.08	4.46	9.50	57.25
NUT	NUT0552	2.0666509	9.66E-05	0.0005937	0.35	1.40	3.00	18.50
AST	AS_YDL179W	2.069287	3.33E-05	0.0002322	1.04	4.47	9.75	55.00
NUT	NUT0690	2.0693996	0.0021307	0.0092254	0.29	1.19	2.50	15.25
NUT	NUT1206	2.0694856	2.89E-10	8.68E-09	3.29	13.97	31.25	188.50
AST	AS_YEL044W	2.0708031	2.07E-05	0.0001521	0.67	2.61	5.25	32.75
NUT	NUT1265	2.0712233	1.21E-08	2.34E-07	1.73	7.46	16.75	98.25
CUT	CUT897	2.0713959	6.52E-07	7.47E-06	1.01	4.23	9.00	53.25
NUT	NUT0198	2.0726265	2.84E-06	2.71E-05	0.70	2.96	6.50	40.50
AST	AS_YLR294C	2.0730997	1.14E-05	9.00E-05	1.02	4.16	8.50	53.25
SUT	SUT105	2.0731435	4.01E-08	6.58E-07	4.20	17.79	39.25	226.25
AST	AS_YKL085W	2.0733636	0.0001149	0.0006933	0.37	1.50	3.25	21.00
NUT	NUT0506	2.073402	0.0003164	0.0016786	0.67	2.77	6.00	40.25
NUT	NUT0626	2.0737769	7.84E-06	6.53E-05	0.88	3.71	8.00	49.00
NUT	NUT1172	2.0762819	1.67E-05	0.0001263	0.54	2.28	5.00	30.50
CUT	CUT797	2.0762885	0.0026207	0.0110573	0.28	1.25	2.75	16.25
NUT	NUT1147	2.0765319	6.63E-06	5.63E-05	0.91	4.05	9.25	51.25

Class	Transcript name	RRP6 KO_vs_WT log2_fold _change	RRP6 KO_vs_WT p-value	RRP6 KO_vs_WT FDR	Ave Norm Reads in WT	Ave Norm Reads in RRP6	Average RAW read counts in WT	Average RAW read counts in RRP6
CUT	CUT363	2.0770186	0.0030539	0.012642	0.27	1.10	2.25	13.75
CUT	CUT030	2.0776564	4.06E-05	0.0002769	0.82	3.51	7.75	45.75
NUT	NUT0230	2.0793076	0.0023943	0.0102175	0.27	1.16	2.50	15.25
NUT	NUT0228	2.0803132	3.05E-08	5.18E-07	4.27	18.17	40.00	230.75
AST	AS_YBR248C	2.0809071	2.74E-05	0.0001948	0.42	1.76	3.75	22.75
NUT	NUT0745	2.0809908	6.70E-05	0.0004306	2.66	11.31	24.25	144.50
ORF-T	YHR069C-A	2.0860133	4.39E-07	5.27E-06	1.07	4.62	10.00	59.50
NUT	NUT1246	2.0863622	1.93E-05	0.0001434	0.48	2.08	4.50	27.25
CUT	CUT909	2.0866257	0.0001265	0.0007557	0.30	1.29	2.75	16.75
NUT	NUT1512	2.0868119	9.43E-07	1.04E-05	0.89	3.78	8.25	50.25
CUT	CUT322	2.0884065	8.84E-08	1.30E-06	1.26	5.26	11.00	69.00
CUT	CUT276	2.0888844	2.14E-06	2.13E-05	0.79	3.39	7.25	43.25
CUT	CUT754	2.089473	3.45E-07	4.24E-06	1.94	8.43	18.75	107.75
AST	AS_YKR076W	2.0902498	5.70E-06	4.94E-05	0.76	3.23	6.75	40.25
SRT	SRT476	2.091313	0.0003513	0.0018458	0.27	1.25	2.75	16.25
CUT	CUT808	2.091409	1.04E-06	1.13E-05	1.19	5.18	11.25	63.25
CUT	CUT503	2.0914507	1.59E-06	1.64E-05	6.04	25.86	57.00	346.00
AST	AS_YHR202W	2.0915342	0.000175	0.0009991	0.45	1.93	4.25	27.00
NUT	NUT1433	2.0918444	3.11E-06	2.94E-05	1.56	6.85	15.50	88.25
NUT	NUT0146	2.0932553	7.43E-07	8.38E-06	1.15	5.13	11.75	67.75
NUT	NUT1177	2.0943481	0.0002041	0.001144	0.83	3.62	7.75	44.50
NUT	NUT0014	2.0943623	9.28E-10	2.44E-08	1.91	8.22	18.00	111.75
NUT	NUT0905	2.0944872	8.57E-06	7.06E-05	0.84	3.62	7.75	48.25
NUT	NUT1222	2.0945075	9.91E-05	0.0006077	0.37	1.53	3.25	21.25
CUT	CUT265	2.0947541	1.48E-07	2.05E-06	3.41	14.59	31.25	194.75
NUT	NUT1223	2.0954986	6.13E-07	7.08E-06	1.41	6.09	13.00	78.75
SUT	SUT100	2.0960349	1.76E-05	0.0001329	1.72	7.46	16.25	94.75
CUT	CUT687	2.0966958	0.0006477	0.0031661	0.34	1.36	2.75	17.50
AST	AS_YBL035C	2.097069	9.56E-10	2.50E-08	1.86	8.02	17.50	109.00

Class	Transcript name	RRP6 KO_vs_WT log2_fold _change	RRP6 KO_vs_WT p-value	RRP6 KO_vs_WT FDR	Ave Norm Reads in WT	Ave Norm Reads in RRP6	Average RAW read counts in WT	Average RAW read counts in RRP6
CUT	CUT424	2.0973079	5.46E-06	4.77E-05	0.57	2.58	5.50	31.50
CUT	CUT268	2.100859	1.13E-06	1.21E-05	0.79	3.32	7.00	44.75
AST	AS_YOR152C	2.1011809	2.07E-07	2.72E-06	1.47	6.55	14.75	86.00
AST	AS_YKR075C	2.1024084	4.88E-06	4.34E-05	1.83	7.95	17.00	98.75
CUT	CUT135	2.1026708	0.0001175	0.0007069	0.46	2.02	4.25	26.00
CUT	CUT842	2.1060999	8.53E-06	7.04E-05	0.58	2.42	5.00	31.25
SUT	SUT442	2.1073221	1.15E-05	9.10E-05	0.75	3.21	6.75	43.00
AST	AS_YDL244W	2.108884	9.01E-06	7.39E-05	0.65	2.87	6.25	37.25
CUT	CUT470	2.1114936	0.0005531	0.0027588	0.33	1.48	3.25	20.50
NUT	NUT0873	2.1117478	0.0006131	0.0030173	0.33	1.48	3.25	20.50
NUT	NUT1298	2.1139447	3.32E-05	0.0002318	0.46	2.10	4.50	27.25
CUT	CUT417	2.1150599	5.53E-06	4.82E-05	0.82	3.47	7.00	45.25
AST	AS_YAL060W	2.1154067	6.87E-05	0.0004406	0.60	2.56	5.50	37.25
NUT	NUT0908	2.1163024	9.70E-07	1.06E-05	6.15	26.75	57.75	357.25
NUT	NUT0725	2.1163498	6.21E-09	1.30E-07	1.96	8.41	17.25	109.25
NUT	NUT1004	2.116361	1.72E-12	9.38E-11	5.32	23.10	48.75	307.50
AST	AS_YLR085C	2.1168785	0.0002444	0.0013461	0.62	2.62	5.50	38.00
CUT	CUT024	2.1170114	8.13E-06	6.73E-05	0.73	3.21	6.75	41.50
NUT	NUT0822	2.1171642	0.0002984	0.0015954	0.32	1.31	2.75	18.50
AST	AS_YOR138C	2.1183373	7.10E-09	1.45E-07	1.95	8.33	17.00	108.00
SRT	SRT203	2.1191637	7.76E-05	0.00049	0.43	1.86	3.75	22.50
ORF-T	YAR075W	2.1204269	9.24E-06	7.53E-05	0.91	4.16	9.25	52.00
SRT	SRT565	2.1209403	0.0005965	0.0029474	0.21	0.94	2.00	12.75
AST	AS_YNL240C	2.1217952	8.69E-07	9.63E-06	0.93	4.05	8.25	49.50
NUT	NUT0182	2.1224121	6.78E-06	5.74E-05	0.42	1.90	4.00	24.50
CUT	CUT494	2.1225543	1.20E-07	1.71E-06	1.04	4.55	9.75	61.75
CUT	CUT175	2.1235387	3.30E-08	5.54E-07	1.77	7.75	16.25	102.50
CUT	CUT407	2.1268203	1.38E-05	0.0001066	0.41	1.71	3.50	22.25
NUT	NUT0768	2.1268253	1.38E-05	0.0001065	0.41	1.71	3.50	22.25

Class	Transcript name	RRP6 KO_vs_WT log2_fold _change	RRP6 KO_vs_WT p-value	RRP6 KO_vs_WT FDR	Ave Norm Reads in WT	Ave Norm Reads in RRP6	Average RAW read counts in WT	Average RAW read counts in RRP6
CUT	CUT610	2.1278863	1.51E-05	0.0001152	0.81	3.53	7.25	48.25
CUT	CUT708	2.1278984	1.20E-05	9.48E-05	0.52	2.33	5.00	32.00
NUT	NUT0911	2.1290026	6.12E-07	7.08E-06	1.22	5.28	10.75	70.00
AST	AS_YGL047W	2.1338271	0.0001816	0.0010309	0.35	1.57	3.25	20.75
NUT	NUT0728	2.1339727	1.53E-09	3.85E-08	2.71	11.92	24.75	154.75
CUT	CUT606	2.1345569	6.27E-08	9.67E-07	2.48	11.13	24.25	139.25
AST	AS_YDR043C	2.13472	3.79E-08	6.25E-07	1.55	6.79	14.00	90.00
CUT	CUT198	2.1357305	0.0005096	0.0025758	0.30	1.31	2.75	17.25
AST	AS_YHR086W	2.1358835	2.03E-07	2.66E-06	0.70	3.15	6.50	39.50
NUT	NUT0197	2.1362653	4.46E-06	4.00E-05	0.53	2.37	5.00	31.75
CUT	CUT325	2.1369627	7.43E-05	0.0004714	0.95	4.21	8.50	51.25
CUT	CUT169	2.137204	0.0003566	0.0018711	0.36	1.50	3.00	19.50
AST	AS_YIL132C	2.138527	3.56E-05	0.0002457	0.33	1.62	3.50	21.00
NUT	NUT1355	2.1395965	1.50E-10	4.88E-09	3.22	14.06	28.50	185.00
NUT	NUT0252	2.1396882	6.15E-05	0.0003998	0.46	2.07	4.25	26.75
AST	AS_YDR506C	2.1397635	1.09E-07	1.56E-06	1.50	6.56	13.50	86.50
AST	AS_YHR167W	2.140011	0.000789	0.0037995	0.19	0.83	1.75	11.50
CUT	CUT855	2.1409177	2.14E-06	2.13E-05	1.53	7.01	15.50	90.50
SRT	SRT446	2.141168	0.0005866	0.0029087	0.32	1.59	3.50	19.75
CUT	CUT666	2.1411799	0.000587	0.0029087	0.32	1.59	3.50	19.75
AST	AS_YGR134W	2.143851	1.60E-06	1.65E-05	0.63	2.72	5.50	35.75
AST	AS_YGR010W	2.1463221	4.53E-09	9.78E-08	1.81	8.08	16.75	106.00
AST	AS_YKR077W	2.1466004	0.0002341	0.0012954	0.28	1.29	2.75	18.00
AST	AS_YGL254W	2.1466117	4.15E-11	1.61E-09	3.82	17.00	35.25	225.50
NUT	NUT1472	2.1473172	1.31E-05	0.000102	0.99	4.49	9.75	62.00
CUT	CUT098	2.1490672	7.31E-07	8.25E-06	0.59	2.63	5.50	35.75
SRT	SRT275	2.1502155	0.0001506	0.0008838	0.26	1.20	2.50	16.00
CUT	CUT378	2.1502159	0.0001507	0.0008838	0.26	1.20	2.50	16.00
CUT	CUT186	2.1545641	3.76E-05	0.0002583	0.31	1.54	3.25	19.75

Class	Transcript name	RRP6 KO_vs_WT log2_fold _change	RRP6 KO_vs_WT p-value	RRP6 KO_vs_WT FDR	Ave Norm Reads in WT	Ave Norm Reads in RRP6	Average RAW read counts in WT	Average RAW read counts in RRP6
NUT	NUT0499	2.1550776	6.24E-09	1.30E-07	1.97	8.86	18.50	119.25
NUT	NUT0174	2.1551928	7.63E-08	1.15E-06	1.50	6.63	13.50	87.50
AST	AS_YIR003W	2.1553577	4.50E-06	4.04E-05	0.79	3.71	8.00	47.00
AST	AS_YDR093W	2.1555513	9.71E-06	7.86E-05	1.16	5.32	11.25	63.50
CUT	CUT304	2.1556881	1.81E-07	2.41E-06	1.43	6.33	13.00	88.25
CUT	CUT535	2.1557895	6.36E-05	0.0004121	0.33	1.48	3.00	19.00
NUT	NUT1186	2.1570809	1.46E-07	2.02E-06	0.83	3.60	7.25	48.00
SRT	SRT100	2.1573444	0.002282	0.0098047	0.22	0.98	2.00	12.75
NUT	NUT1323	2.1575424	6.74E-05	0.0004328	0.50	2.23	4.50	30.25
CUT	CUT728	2.1593859	3.30E-05	0.0002309	0.41	1.86	3.75	23.25
AST	AS_YNL127W	2.1597029	1.46E-05	0.0001123	0.55	2.56	5.50	35.50
NUT	NUT1337	2.1598663	7.43E-08	1.12E-06	0.89	4.16	8.75	54.00
CUT	CUT296	2.1615739	0.0012968	0.0059334	0.24	1.09	2.25	14.75
ORF-T	YML012C-A	2.1626562	1.78E-05	0.000134	0.68	2.99	6.00	40.00
CUT	CUT054	2.1654157	4.12E-05	0.0002801	0.33	1.57	3.25	20.75
CUT	CUT390	2.1665533	2.98E-06	2.83E-05	0.54	2.47	5.00	32.00
CUT	CUT385	2.167354	3.27E-09	7.39E-08	1.85	8.44	17.50	110.25
ORF-T	YEL030C-A	2.1679057	0.0003003	0.0016025	0.21	1.05	2.25	14.75
AST	AS_YNR035C	2.1679138	0.0028314	0.0118323	0.14	0.61	1.25	8.50
ORF-T	YDR136C	2.1685037	0.0001224	0.0007332	0.33	1.56	3.25	21.00
AST	AS_YKL025C	2.1714863	5.07E-05	0.0003382	1.08	4.81	9.25	57.25
CUT	CUT454	2.1717031	6.46E-05	0.0004172	0.35	1.52	3.00	19.50
AST	AS_YMR222C	2.1720142	4.37E-08	7.02E-07	1.15	5.15	10.50	68.50
AST	AS_YNL209W	2.1745898	0.0002332	0.0012918	0.62	2.88	5.75	34.50
AST	AS_YBR265W	2.1766057	5.53E-06	4.82E-05	1.48	6.61	13.00	90.25
AST	AS_YIL123W	2.1779081	1.03E-07	1.49E-06	1.79	8.20	16.75	105.50
NUT	NUT1499	2.1787512	0.0002748	0.0014872	0.36	1.56	3.00	20.00
AST	AS_YPL158C	2.1790089	1.70E-08	3.12E-07	1.08	4.79	9.50	64.00
CUT	CUT519	2.1791178	0.0004009	0.0020764	0.54	2.58	5.50	33.25

Class	Transcript name	RRP6 KO_vs_WT log2_fold_change	RRP6 KO_vs_WT p-value	RRP6 KO_vs_WT FDR	Ave Norm Reads in WT	Ave Norm Reads in RRP6	Average RAW read counts in WT	Average RAW read counts in RRP6
AST	AS_YOR034C-A	2.1791492	0.0012025	0.0055319	0.19	0.87	1.75	11.50
SUT	SUT064	2.1806596	0.000638	0.0031246	0.40	1.73	3.25	21.75
AST	AS_YJL097W	2.1814813	0.0002788	0.0015051	0.31	1.29	2.50	17.50
AST	AS_YGR202C	2.184002	5.67E-05	0.0003745	0.33	1.52	3.00	19.25
AST	AS_YNL117W	2.1848058	1.28E-10	4.28E-09	2.96	13.25	25.75	174.50
AST	AS_YOL052C	2.1860633	1.05E-06	1.15E-05	3.05	13.98	28.00	169.75
CUT	CUT020	2.1861283	1.04E-05	8.35E-05	0.67	3.17	6.75	44.25
CUT	CUT118	2.1863803	3.90E-05	0.0002667	0.48	2.15	4.25	28.75
AST	AS_YDR284C	2.1874139	1.49E-08	2.80E-07	1.70	7.70	15.25	103.00
AST	AS_YDR486C	2.1896455	4.02E-07	4.87E-06	0.59	2.70	5.50	36.75
AST	AS_YBR016W	2.1899914	7.61E-05	0.0004814	0.26	1.15	2.25	14.75
CUT	CUT575	2.1913838	4.52E-06	4.05E-05	0.64	3.00	6.00	38.00
AST	AS_YJR059W	2.1916528	1.38E-07	1.92E-06	1.24	5.67	11.50	77.25
AST	AS_YOR298C-A	2.1921379	0.000398	0.0020637	0.29	1.30	2.50	16.25
CUT	CUT422	2.1929572	5.07E-05	0.0003379	0.32	1.43	2.75	18.00
ORF-T	YKL097C	2.1933289	3.61E-08	6.01E-07	0.81	3.59	7.00	47.25
AST	AS_YLR245C	2.1935562	1.23E-08	2.37E-07	0.92	4.37	9.00	56.25
CUT	CUT827	2.1941078	1.88E-06	1.89E-05	1.48	6.71	13.00	88.75
CUT	CUT583	2.1943577	3.47E-12	1.78E-10	4.28	19.63	39.25	262.00
CUT	CUT027	2.194538	7.16E-08	1.09E-06	1.24	5.65	11.25	77.00
AST	AS_YKR007W	2.1954937	5.71E-06	4.94E-05	0.37	1.85	3.75	23.50
AST	AS_YFR025C	2.1955559	3.48E-08	5.80E-07	1.16	5.38	10.75	69.50
AST	AS_YBR116C	2.1957893	3.55E-06	3.29E-05	0.63	2.80	5.50	37.50
NUT	NUT0789	2.1966426	5.08E-07	5.98E-06	0.79	3.75	7.50	47.50
CUT	CUT442	2.1968276	1.35E-06	1.41E-05	0.70	3.38	7.00	43.75
NUT	NUT1317	2.2001738	2.22E-08	3.93E-07	1.40	6.46	12.75	81.25
NUT	NUT0087	2.201036	1.82E-06	1.84E-05	0.96	4.44	9.00	62.25
AST	AS_YNL278W	2.2010537	1.32E-07	1.85E-06	0.80	3.82	7.75	49.50
sn/snoRNA ET	SNR82-ET	2.2029972	1.00E-14	1.02E-12	8.42	38.75	76.25	504.75

Class	Transcript name	RRP6 KO_vs_WT log2_fold _change	RRP6 KO_vs_WT p-value	RRP6 KO_vs_WT FDR	Ave Norm Reads in WT	Ave Norm Reads in RRP6	Average RAW read counts in WT	Average RAW read counts in RRP6
NUT	NUT1465	2.2041753	2.73E-06	2.62E-05	0.71	3.23	6.50	44.50
NUT	NUT0800	2.2045237	1.98E-05	0.0001464	0.53	2.50	5.00	32.75
AST	AS_YPL208W	2.2048723	4.51E-06	4.04E-05	0.69	3.12	6.25	43.25
CUT	CUT214	2.2057069	0.0009884	0.0046489	0.21	1.03	2.00	12.50
AST	AS_YDR094W	2.2073477	0.0005386	0.0027009	0.42	2.09	4.25	24.25
SRT	SRT125	2.2076649	0.0028117	0.0117597	0.36	1.75	3.75	23.00
CUT	CUT702	2.20839	2.49E-07	3.17E-06	0.98	4.38	8.25	56.25
AST	AS_YOR284W	2.209587	2.41E-06	2.36E-05	0.73	3.28	6.25	43.75
NUT	NUT0396	2.2098902	1.20E-05	9.42E-05	0.35	1.66	3.25	21.50
AST	AS_YJR047C	2.2108175	1.19E-06	1.27E-05	0.75	3.47	6.75	43.75
CUT	CUT391	2.21121	0.0002894	0.0015524	0.46	2.13	4.00	26.00
NUT	NUT1134	2.2129564	6.71E-08	1.03E-06	1.76	8.27	16.50	106.25
AST	AS_YGL067W	2.213021	0.0001114	0.0006742	0.29	1.54	3.25	21.00
CUT	CUT885	2.2130599	9.74E-06	7.88E-05	0.58	2.56	5.00	35.75
CUT	CUT344	2.2155283	9.70E-06	7.86E-05	0.62	2.96	5.75	36.00
AST	AS_YPL181W	2.2158933	0.0001176	0.0007074	0.22	1.02	2.00	13.50
AST	AS_YJR115W	2.2162121	5.42E-07	6.31E-06	0.60	2.90	5.75	37.25
SUT	SUT277	2.2179821	6.42E-09	1.33E-07	0.97	4.69	9.50	60.25
AST	AS_YBR148W	2.2201092	4.45E-07	5.33E-06	0.46	2.08	4.00	26.75
AST	AS_YOR327C	2.2208388	9.87E-06	7.96E-05	0.55	2.59	5.00	33.25
NUT	NUT1094	2.2214989	2.15E-09	5.13E-08	1.69	7.74	14.75	101.00
AST	AS_YDR213W	2.222872	3.52E-07	4.32E-06	1.71	7.67	13.50	96.50
AST	AS_YOL097C	2.2229115	1.26E-06	1.33E-05	0.72	3.36	6.50	43.00
NUT	NUT1115	2.223462	1.03E-08	2.04E-07	2.02	9.34	18.00	124.25
CUT	CUT321	2.2247916	1.02E-05	8.16E-05	0.71	3.50	7.00	41.50
ORF-T	YAR073W	2.2263204	6.33E-08	9.75E-07	2.29	10.95	22.75	142.75
AST	AS_YGL255W	2.2287431	8.25E-08	1.22E-06	1.04	4.85	9.50	66.00
NUT	NUT1189	2.2300873	1.17E-07	1.67E-06	1.66	7.58	14.00	98.00
NUT	NUT1015	2.2309222	2.31E-09	5.49E-08	1.94	8.80	16.00	116.50

Class	Transcript name	RRP6 KO_vs_WT log2_fold _change	RRP6 KO_vs_WT p-value	RRP6 KO_vs_WT FDR	Ave Norm Reads in WT	Ave Norm Reads in RRP6	Average RAW read counts in WT	Average RAW read counts in RRP6
NUT	NUT0992	2.23093	3.28E-06	3.06E-05	0.64	3.09	6.00	39.00
AST	AS_YOL135C	2.231783	5.54E-06	4.82E-05	0.53	2.55	5.00	33.50
CUT	CUT724	2.2320104	7.90E-08	1.18E-06	1.17	5.50	10.75	72.25
NUT	NUT1452	2.2326828	5.25E-06	4.61E-05	0.44	2.14	4.25	29.00
NUT	NUT0639	2.2329785	3.71E-06	3.41E-05	1.11	5.20	9.75	64.50
NUT	NUT0448	2.2330421	4.08E-05	0.0002782	0.30	1.34	2.50	17.00
AST	AS_YCL055W	2.2354247	2.74E-09	6.31E-08	2.78	13.01	25.00	176.75
AST	AS_YGR003W	2.2370354	0.0005122	0.0025865	0.19	1.00	2.00	13.25
AST	AS_YPR040W	2.2377134	4.14E-08	6.78E-07	2.83	13.47	27.25	182.25
CUT	CUT434	2.2392801	0.0002731	0.0014808	0.20	1.03	2.00	13.25
CUT	CUT270	2.2398772	1.57E-07	2.15E-06	1.42	6.73	13.00	87.50
AST	AS_YAL028W	2.2411339	0.0002015	0.0011306	0.28	1.29	2.50	18.25
CUT	CUT657	2.2416607	1.69E-09	4.15E-08	1.12	5.18	9.75	67.50
NUT	NUT0790	2.2424708	1.12E-11	5.03E-10	3.25	15.37	29.75	201.25
CUT	CUT178	2.2435699	5.05E-06	4.46E-05	0.80	3.84	7.50	50.25
NUT	NUT0287	2.2449549	1.49E-07	2.05E-06	0.69	3.29	6.25	42.25
NUT	NUT0922	2.2449768	1.72E-07	2.30E-06	1.77	8.04	14.00	101.75
AST	AS_YMR191W	2.2454442	1.78E-07	2.38E-06	1.24	6.06	12.25	79.25
NUT	NUT1392	2.2467846	3.34E-06	3.11E-05	0.51	2.45	4.75	33.50
AST	AS_YBR233W	2.2468383	1.85E-06	1.87E-05	0.57	2.71	5.25	36.50
CUT	CUT200	2.2472165	5.62E-09	1.19E-07	1.41	6.58	12.25	83.50
AST	AS_YER053C	2.2494682	4.71E-05	0.0003169	0.26	1.27	2.50	17.25
AST	AS_YHL018W	2.2544637	0.0001846	0.001045	0.55	2.50	4.50	34.75
NUT	NUT0217	2.2559961	5.57E-06	4.84E-05	1.42	6.92	13.50	87.25
NUT	NUT1285	2.2571666	4.92E-07	5.82E-06	0.79	3.86	7.50	51.50
AST	AS_YIL041W	2.2583088	5.90E-05	0.0003873	0.55	2.63	5.00	35.50
AST	AS_YPR061C	2.2586234	3.42E-05	0.000238	0.48	2.34	4.50	30.50
AST	AS_YDR530C	2.2607332	4.19E-06	3.79E-05	0.43	2.27	4.50	29.75
AST	AS_YJL077W-B	2.2610984	0.000517	0.0026054	0.22	1.05	2.00	13.75

Class	Transcript name	RRP6 KO_vs_WT log2_fold _change	RRP6 KO_vs_WT p-value	RRP6 KO_vs_WT FDR	Ave Norm Reads in WT	Ave Norm Reads in RRP6	Average RAW read counts in WT	Average RAW read counts in RRP6
CUT	CUT499	2.2626676	9.19E-06	7.50E-05	0.55	2.72	5.25	35.75
NUT	NUT1097	2.2637379	0.0001405	0.0008306	0.22	1.16	2.25	15.25
CUT	CUT076	2.2639593	7.84E-08	1.18E-06	1.24	5.84	10.75	78.75
SUT	SUT197	2.2642973	0.0012478	0.0057221	0.24	1.16	2.25	16.25
CUT	CUT768	2.264928	0.0001194	0.0007167	0.40	1.91	3.50	24.25
AST	AS_YDL234C	2.2653146	4.90E-06	4.34E-05	0.81	3.82	7.25	53.50
AST	AS_YMR163C	2.2662558	7.17E-09	1.46E-07	1.68	8.13	15.75	113.00
CUT	CUT890	2.2670706	8.10E-05	0.0005067	0.22	1.05	2.00	14.00
AST	AS_YHR178W	2.2681284	1.27E-07	1.78E-06	1.39	6.64	12.50	87.25
AST	AS_YBR128C	2.2688368	3.05E-08	5.18E-07	1.30	6.30	12.00	86.25
SUT	SUT729	2.2702043	1.04E-06	1.13E-05	0.67	3.25	6.25	44.25
AST	AS_YPL041C	2.2706943	2.29E-05	0.0001671	0.32	1.50	2.75	19.00
NUT	NUT0542	2.27315	0.0001434	0.0008463	0.37	1.83	3.50	25.00
SUT	SUT142	2.274553	2.85E-07	3.58E-06	0.60	2.84	5.25	36.75
CUT	CUT642	2.2750823	0.0004264	0.0021917	0.38	1.81	3.50	25.75
NUT	NUT1511	2.2778225	5.73E-06	4.96E-05	0.46	2.36	4.50	30.50
ORF-T	YLR255C	2.2778863	3.49E-07	4.28E-06	1.02	5.11	10.25	71.00
NUT	NUT0059	2.2786299	4.59E-06	4.10E-05	0.42	2.02	3.75	26.00
NUT	NUT0739	2.2808453	3.87E-07	4.71E-06	0.57	2.81	5.25	36.50
NUT	NUT0044	2.2810513	2.25E-08	3.95E-07	1.30	6.35	12.00	87.00
NUT	NUT0595	2.2817494	7.14E-07	8.08E-06	1.19	5.81	10.75	74.50
AST	AS_YKR019C	2.2823421	6.59E-08	1.01E-06	0.86	4.12	7.50	52.50
AST	AS_YPR182W	2.2836913	3.77E-10	1.10E-08	1.93	9.20	16.50	119.50
AST	AS_YJR013W	2.2838583	1.20E-07	1.70E-06	0.60	2.81	5.25	38.00
AST	AS_YNL071W	2.2843115	3.80E-06	3.48E-05	1.57	7.81	15.75	109.50
CUT	CUT811	2.2850734	1.50E-08	2.81E-07	1.75	8.23	14.25	107.75
AST	AS_YOR336W	2.2867934	1.59E-06	1.64E-05	0.63	3.07	5.75	40.75
AST	AS_YCL043C	2.2892853	2.15E-06	2.14E-05	0.63	3.05	5.75	42.50
CUT	CUT638	2.2917813	0.0001039	0.000632	0.61	2.85	5.00	39.25

TABLE S1: Differential expression data for RRP6 RNA-Seq dataset Page 315

Class	Transcript name	RRP6 KO_vs_WT log2_fold_change	RRP6 KO_vs_WT p-value	RRP6 KO_vs_WT FDR	Ave Norm Reads in WT	Ave Norm Reads in RRP6	Average RAW read counts in WT	Average RAW read counts in RRP6
NUT	NUT1089	2.2918009	0.0001036	0.0006306	0.61	2.85	5.00	39.25
NUT	NUT0871	2.2932219	2.75E-11	1.10E-09	2.48	12.13	22.25	155.25
AST	AS_YHL022C	2.2945487	2.33E-07	3.00E-06	0.58	2.92	5.50	38.25
AST	AS_YJL093C	2.2950961	5.78E-06	4.99E-05	0.33	1.63	3.00	21.25
AST	AS_YPL083C	2.2953985	9.91E-07	1.09E-05	0.39	2.09	4.00	27.50
NUT	NUT0352	2.2962035	1.54E-10	5.00E-09	2.13	10.55	20.00	141.25
CUT	CUT196	2.2977048	7.17E-07	8.11E-06	0.61	3.01	5.50	38.00
NUT	NUT0033	2.2981043	8.06E-09	1.62E-07	1.04	5.26	10.00	67.75
NUT	NUT0009	2.2982043	1.71E-10	5.48E-09	3.47	17.42	34.50	225.50
AST	AS_YPL031C	2.2983966	4.28E-12	2.18E-10	3.06	15.04	28.00	197.25
AST	AS_YGL196W	2.2992696	1.42E-08	2.71E-07	1.34	6.23	10.75	82.25
AST	AS_YOR052C	2.2998704	9.86E-06	7.96E-05	0.35	1.73	3.25	23.75
AST	AS_YGL044C	2.3006662	0.0011968	0.0055083	0.20	0.96	1.75	12.50
sn/snoRNA ET	SNR189-ET	2.3014176	1.29E-12	7.21E-11	7.26	35.61	63.00	448.50
CUT	CUT217	2.3022092	0.0004809	0.0024404	0.38	1.86	3.25	22.25
AST	AS_YKL183W	2.3049653	1.14E-12	6.49E-11	2.56	12.67	23.50	165.50
AST	AS_YNL042W-B	2.3052642	2.78E-05	0.0001974	0.29	1.40	2.50	18.00
AST	AS_YOR299W	2.3077787	2.42E-06	2.37E-05	0.60	2.96	5.50	39.50
ORF-T	YJR018W	2.3082735	7.50E-06	6.30E-05	0.47	2.23	4.00	29.75
NUT	NUT0117	2.3094266	4.53E-09	9.78E-08	2.19	10.66	19.00	140.50
AST	AS_YDR314C	2.3123132	9.68E-05	0.0005945	0.25	1.33	2.50	17.75
NUT	NUT1396	2.3124379	0.0001347	0.0007989	0.63	3.10	5.50	36.75
NUT	NUT0949	2.3124489	2.66E-06	2.56E-05	0.41	2.14	4.00	27.00
AST	AS_YOR328W	2.3132107	1.77E-07	2.37E-06	1.03	5.22	9.75	63.75
AST	AS_YEL066W	2.3148333	1.07E-05	8.50E-05	0.42	2.11	3.75	25.50
CUT	CUT249	2.3151174	0.0006919	0.0033656	0.31	1.61	3.00	22.00
NUT	NUT0026	2.3159382	2.54E-08	4.44E-07	0.95	4.55	8.00	58.25
NUT	NUT0487	2.3165444	0.0002378	0.0013132	0.38	1.92	3.50	26.00
NUT	NUT0585	2.3166315	0.0001988	0.0011167	0.24	1.13	2.00	14.50

TABLE S1: Differential expression data for RRP6 RNA-Seq dataset Page 316

Class	Transcript name	RRP6 KO_vs_WT log2_fold _change	RRP6 KO_vs_WT p-value	RRP6 KO_vs_WT FDR	Ave Norm Reads in WT	Ave Norm Reads in RRP6	Average RAW read counts in WT	Average RAW read counts in RRP6
sn/snoRNA	SNR87-A	2.3173628	1.76E-09	4.31E-08	4.52	22.79	43.50	280.00
CUT	CUT584	2.3174799	3.22E-05	0.0002257	0.34	1.66	3.00	22.00
NUT	NUT0848	2.3176686	1.96E-07	2.59E-06	0.42	2.08	3.75	26.75
CUT	CUT085	2.3187319	1.31E-05	0.0001017	0.42	2.15	4.00	28.00
AST	AS_YDR315C	2.3190051	1.67E-06	1.71E-05	0.67	3.51	6.75	45.50
AST	AS_YOL064C	2.3208213	0.0009577	0.0045235	0.19	0.86	1.50	11.50
ORF-T	YDR327W	2.3222886	0.0001316	0.0007833	0.22	1.11	2.00	14.50
AST	AS_YGR193C	2.3228068	1.85E-06	1.86E-05	0.92	4.52	8.00	58.50
NUT	NUT1451	2.3237725	5.32E-07	6.20E-06	0.67	3.32	6.00	43.50
AST	AS_YGR091W	2.3245889	1.25E-07	1.76E-06	0.96	4.69	8.25	61.75
AST	AS_YHR008C	2.3254122	4.94E-10	1.39E-08	3.60	18.06	33.00	240.50
NUT	NUT1087	2.325758	3.24E-06	3.04E-05	0.45	2.09	3.75	29.25
NUT	NUT0124	2.3272678	3.68E-06	3.40E-05	0.37	1.82	3.25	23.25
SUT	SUT412	2.3286333	8.77E-12	4.13E-10	2.72	13.72	25.25	179.25
AST	AS_YOR091W	2.3292859	1.08E-07	1.55E-06	0.72	3.53	6.25	45.75
CUT	CUT160	2.3296848	2.26E-06	2.23E-05	0.72	3.57	6.25	45.50
AST	AS_YDR361C	2.3306224	7.90E-05	0.0004975	0.26	1.15	2.00	15.00
sn/snoRNA ET	SNR8-ET	2.3308206	4.31E-10	1.23E-08	46.97	236.13	403.50	2908.25
NUT	NUT0599	2.3313642	1.38E-09	3.50E-08	1.68	8.53	15.75	118.25
CUT	CUT888	2.3321163	1.53E-05	0.0001168	0.36	1.91	3.50	24.00
NUT	NUT0343	2.3322287	2.21E-06	2.19E-05	0.95	4.78	8.50	63.25
AST	AS_YGL022W	2.3332259	0.0003394	0.0017898	0.35	1.93	3.75	27.25
CUT	CUT174	2.333721	0.0003356	0.001772	0.20	1.00	1.75	12.75
NUT	NUT0740	2.3341223	0.0001864	0.0010551	0.40	2.03	3.50	24.75
NUT	NUT0116	2.3352655	2.10E-09	5.04E-08	0.86	4.33	7.75	56.00
AST	AS_YDR030C	2.335268	2.09E-09	5.03E-08	0.86	4.33	7.75	56.00
AST	AS_YGR031W	2.3359067	2.83E-07	3.56E-06	0.53	2.89	5.50	38.25
NUT	NUT1449	2.3365159	2.08E-06	2.07E-05	0.60	3.02	5.50	40.25
NUT	NUT0776	2.3395406	4.68E-07	5.56E-06	1.00	5.10	9.25	67.50

Class	Transcript name	RRP6 KO_vs_WT log2_fold_change	RRP6 KO_vs_WT p-value	RRP6 KO_vs_WT FDR	Ave Norm Reads in WT	Ave Norm Reads in RRP6	Average RAW read counts in WT	Average RAW read counts in RRP6
ORF-T	YOR293C-A	2.3435413	7.10E-05	0.0004525	0.24	1.27	2.25	16.50
NUT	NUT1061	2.3447604	5.46E-08	8.54E-07	0.98	4.87	8.50	64.00
CUT	CUT580	2.3457092	0.0003835	0.0019967	0.22	1.11	2.00	15.75
SRT	SRT442	2.3461757	2.45E-05	0.0001769	0.37	1.86	3.25	23.00
NUT	NUT0410	2.3472656	6.35E-08	9.76E-07	0.85	4.17	7.25	55.00
CUT	CUT067	2.3483702	3.19E-06	3.00E-05	0.33	1.70	3.00	21.75
ORF-T	YFR035C	2.3489582	1.43E-06	1.50E-05	0.34	1.69	3.00	22.25
CUT	CUT643	2.3490283	2.99E-06	2.84E-05	0.33	1.77	3.25	23.50
NUT	NUT0303	2.350533	6.99E-08	1.06E-06	0.60	2.99	5.25	39.00
NUT	NUT1099	2.3505626	6.39E-09	1.32E-07	0.86	4.36	7.75	57.50
NUT	NUT0461	2.350688	2.08E-07	2.72E-06	1.31	6.60	11.25	80.50
AST	AS_YGL222C	2.3523673	2.65E-05	0.0001895	0.31	1.55	2.75	20.75
CUT	CUT444	2.3527111	0.0001034	0.0006298	0.21	1.02	1.75	12.75
NUT	NUT0481	2.3529618	0.0003666	0.0019206	0.14	0.72	1.25	9.25
CUT	CUT562	2.3531151	2.85E-08	4.90E-07	0.77	3.99	7.25	53.25
AST	AS_YNL142W	2.3535523	0.0001693	0.0009745	0.23	1.24	2.25	16.25
NUT	NUT1199	2.3536233	1.55E-09	3.88E-08	2.30	11.96	22.00	152.75
NUT	NUT0016	2.3544044	6.55E-09	1.35E-07	2.48	12.58	21.50	160.00
AST	AS_YBR053C	2.3549652	1.39E-07	1.93E-06	0.81	3.97	6.75	51.00
SUT	SUT053	2.355362	2.92E-09	6.68E-08	2.12	10.61	18.25	140.00
CUT	CUT595	2.3561717	0.0003128	0.001661	0.30	1.46	2.50	19.25
CUT	CUT324	2.35671	0.0003312	0.0017513	0.23	1.14	2.00	15.25
NUT	NUT0320	2.3582827	9.22E-08	1.35E-06	0.58	3.05	5.50	40.25
CUT	CUT835	2.3593635	9.80E-08	1.42E-06	0.50	2.82	5.25	37.25
CUT	CUT922	2.3613474	9.67E-11	3.40E-09	1.99	10.02	17.00	130.50
CUT	CUT774	2.3614053	1.35E-05	0.0001048	0.28	1.41	2.50	18.75
AST	AS_YBR223C	2.361883	0.0009548	0.0045118	0.30	1.57	2.75	19.50
NUT	NUT0996	2.362981	7.95E-12	3.80E-10	4.84	25.18	46.75	325.25
CUT	CUT211	2.3642294	3.22E-05	0.0002257	0.35	1.86	3.25	23.00

Class	Transcript name	RRP6 KO_vs_WT log2_fold _change	RRP6 KO_vs_WT p-value	RRP6 KO_vs_WT FDR	Ave Norm Reads in WT	Ave Norm Reads in RRP6	Average RAW read counts in WT	Average RAW read counts in RRP6
CUT	CUT092	2.3667784	0.0002882	0.0015477	0.19	0.89	1.50	11.25
AST	AS_YDR424C	2.3668491	0.0002854	0.0015369	0.19	0.89	1.50	11.25
AST	AS_YLR024C	2.36697	2.32E-10	7.12E-09	1.44	7.77	14.50	98.75
NUT	NUT0488	2.3669777	2.32E-10	7.12E-09	1.44	7.77	14.50	98.75
AST	AS_YDR326C	2.3672956	1.96E-06	1.97E-05	0.48	2.54	4.50	32.25
CUT	CUT432	2.36841	1.91E-09	4.63E-08	1.12	5.83	10.25	74.00
AST	AS_YMR012W	2.3699757	0.0008526	0.0040769	0.36	1.93	3.50	23.75
AST	AS_YDR167W	2.3708697	0.0014324	0.0065033	0.18	0.89	1.50	11.50
CUT	CUT668	2.3709241	7.86E-06	6.54E-05	0.35	1.94	3.50	24.75
CUT	CUT315	2.3714729	4.34E-08	7.01E-07	0.56	3.03	5.50	41.00
AST	AS_YDL204W	2.3758454	2.12E-05	0.0001556	0.35	1.72	3.00	23.50
CUT	CUT079	2.3765304	0.0001579	0.0009184	0.19	0.99	1.75	13.25
NUT	NUT1181	2.3775911	1.20E-08	2.34E-07	2.06	10.80	19.50	143.25
AST	AS_YLR074C	2.377657	7.96E-07	8.94E-06	0.69	3.48	6.00	47.50
CUT	CUT696	2.3790613	1.69E-05	0.0001277	0.31	1.68	3.00	22.50
CUT	CUT615	2.3803839	6.60E-10	1.79E-08	1.80	9.07	15.00	123.75
AST	AS_YMR092C	2.3813232	0.0002297	0.0012753	0.22	1.04	1.75	13.50
CUT	CUT645	2.3827738	6.23E-09	1.30E-07	0.80	4.16	7.25	54.50
AST	AS_YJL081C	2.386667	0.0001436	0.0008466	0.26	1.31	2.25	18.25
ORF-T	YJL026C-A	2.3868691	3.92E-07	4.76E-06	0.77	4.17	7.50	55.50
CUT	CUT250	2.389903	3.90E-10	1.14E-08	1.38	7.62	14.00	96.50
AST	AS_YNL175C	2.3934684	3.02E-06	2.87E-05	0.44	2.12	3.50	27.50
AST	AS_YNL290W	2.3936809	6.42E-05	0.0004152	0.22	1.04	1.75	13.50
CUT	CUT467	2.3938361	7.35E-10	1.97E-08	1.36	7.36	13.25	96.75
CUT	CUT159	2.3958018	1.08E-05	8.58E-05	0.33	1.63	2.75	21.25
NUT	NUT1353	2.396193	5.07E-06	4.47E-05	0.50	2.70	4.75	35.75
NUT	NUT1352	2.397281	4.01E-06	3.66E-05	0.42	2.29	4.00	29.00
CUT	CUT871	2.3993594	5.86E-05	0.0003853	0.47	2.56	4.50	33.25
NUT	NUT0718	2.3998811	1.93E-07	2.55E-06	0.39	2.05	3.50	26.25

TABLE S1: Differential expression data for RRP6 RNA-Seq dataset Page 319

Class	Transcript name	RRP6 KO_vs_WT log2_fold _change	RRP6 KO_vs_WT p-value	RRP6 KO_vs_WT FDR	Ave Norm Reads in WT	Ave Norm Reads in RRP6	Average RAW read counts in WT	Average RAW read counts in RRP6
CUT	CUT619	2.4003643	8.01E-07	8.99E-06	0.44	2.21	3.75	29.00
AST	AS_YBR219C	2.4010589	6.70E-06	5.68E-05	0.33	1.86	3.25	24.00
AST	AS_YFR021W	2.401077	3.46E-12	1.78E-10	1.81	9.76	17.50	130.25
CUT	CUT825	2.4017123	0.0001745	0.0009967	0.54	2.85	4.75	33.75
AST	AS_YER012W	2.4033525	7.12E-06	6.00E-05	0.53	2.82	4.75	36.00
CUT	CUT785	2.4055485	6.08E-09	1.28E-07	1.15	6.00	10.00	75.25
AST	AS_YJR156C	2.4073768	5.31E-05	0.0003521	0.24	1.32	2.25	16.25
NUT	NUT0665	2.4073773	1.35E-06	1.42E-05	0.36	1.80	3.00	23.50
CUT	CUT820	2.4077147	2.42E-09	5.72E-08	1.13	5.94	10.00	75.75
NUT	NUT0147	2.4091761	1.70E-09	4.18E-08	1.15	6.25	11.00	81.50
CUT	CUT572	2.4099103	5.26E-07	6.16E-06	0.71	3.96	7.00	51.00
AST	AS_YCR014C	2.4122951	0.0002747	0.0014872	0.18	0.93	1.50	11.25
CUT	CUT578	2.413152	3.74E-13	2.48E-11	2.01	10.90	19.25	145.00
NUT	NUT0360	2.4144267	9.95E-07	1.09E-05	0.34	2.10	3.75	27.25
CUT	CUT210	2.4153341	9.16E-07	1.01E-05	0.46	2.40	4.00	30.50
NUT	NUT0706	2.4154222	2.26E-06	2.23E-05	0.38	2.23	4.00	28.50
CUT	CUT382	2.4168693	3.29E-07	4.07E-06	0.36	1.92	3.25	24.75
AST	AS_YMR199W	2.4178096	5.18E-06	4.55E-05	0.43	2.58	4.75	34.00
NUT	NUT1332	2.4199352	2.36E-05	0.0001711	0.25	1.21	2.00	15.75
AST	AS_YBR299W	2.4209443	4.91E-07	5.82E-06	0.72	3.75	6.25	51.25
NUT	NUT0430	2.4241828	4.19E-05	0.0002839	0.24	1.34	2.25	16.50
NUT	NUT0997	2.4279854	3.10E-06	2.93E-05	0.70	3.71	6.25	51.75
AST	AS_YFL042C	2.4297707	0.0005386	0.0027009	0.14	0.76	1.25	9.75
AST	AS_YMR198W	2.4298567	9.11E-10	2.40E-08	1.08	5.83	9.75	73.50
AST	AS_YLR099W-A	2.4312893	2.80E-06	2.68E-05	0.38	2.14	3.75	29.25
NUT	NUT0645	2.4318464	1.11E-06	1.20E-05	0.51	2.52	4.00	32.25
AST	AS_YDR493W	2.4318865	2.82E-08	4.86E-07	0.53	2.84	4.75	36.75
NUT	NUT1375	2.4337256	9.35E-08	1.37E-06	0.68	3.78	6.50	49.50
CUT	CUT781	2.4338498	7.63E-08	1.15E-06	0.98	5.40	9.25	70.00

Class	Transcript name	RRP6 KO_vs_WT log2_fold _change	RRP6 KO_vs_WT p-value	RRP6 KO_vs_WT FDR	Ave Norm Reads in WT	Ave Norm Reads in RRP6	Average RAW read counts in WT	Average RAW read counts in RRP6
NUT	NUT1204	2.4340715	7.10E-10	1.91E-08	2.27	12.24	20.25	156.75
CUT	CUT814	2.4341526	1.65E-08	3.05E-07	0.90	5.00	8.50	60.25
NUT	NUT0866	2.4355034	1.33E-10	4.40E-09	1.47	8.22	14.50	108.25
CUT	CUT770	2.4392483	5.02E-05	0.0003354	0.19	1.05	1.75	13.50
NUT	NUT0480	2.4409717	7.95E-07	8.94E-06	1.52	8.28	13.75	108.00
AST	AS_YLL050C	2.4409727	8.00E-07	8.99E-06	1.52	8.28	13.75	108.00
NUT	NUT0985	2.4424734	3.95E-10	1.14E-08	1.35	7.17	11.75	95.75
CUT	CUT080	2.4426073	1.65E-06	1.69E-05	0.50	2.95	5.25	38.50
SRT	SRT178	2.4460558	7.57E-07	8.53E-06	1.52	8.30	13.75	108.50
CUT	CUT735	2.4460581	0.0008427	0.0040367	0.15	0.90	1.50	11.25
AST	AS_YPL052W	2.447269	1.89E-07	2.50E-06	0.52	2.96	5.00	37.50
CUT	CUT704	2.4475744	6.02E-12	2.94E-10	1.68	9.17	15.25	122.25
AST	AS_YBR072W	2.4487877	6.25E-07	7.20E-06	0.37	2.21	3.75	27.75
AST	AS_YMR188C	2.4496047	1.91E-05	0.0001418	0.29	1.54	2.50	20.00
AST	AS_YBR070C	2.4504634	1.68E-09	4.13E-08	0.74	4.15	7.00	53.75
NUT	NUT0806	2.4505468	5.12E-05	0.0003408	0.61	3.21	5.00	41.75
NUT	NUT0205	2.4522058	1.63E-08	3.02E-07	0.68	3.73	6.25	51.00
CUT	CUT816	2.4529935	2.42E-10	7.37E-09	1.35	7.26	11.75	95.50
NUT	NUT1367	2.4534738	2.82E-13	1.95E-11	2.16	11.94	20.25	160.00
NUT	NUT0069	2.4534817	7.26E-06	6.11E-05	0.32	1.68	2.75	22.00
CUT	CUT428	2.454464	0.000266	0.0014462	0.27	1.42	2.25	18.50
NUT	NUT1450	2.4549407	6.24E-08	9.65E-07	0.68	3.79	6.25	46.50
NUT	NUT1070	2.4558598	4.64E-08	7.38E-07	1.03	5.57	9.00	73.00
AST	AS_YNL254C	2.4578906	1.58E-07	2.16E-06	0.82	4.40	7.25	59.50
CUT	CUT007	2.4583769	1.22E-09	3.11E-08	2.03	11.05	17.75	142.00
NUT	NUT0273	2.4655376	8.15E-05	0.000509	0.20	1.07	1.75	14.00
AST	AS_YHR035W	2.4670343	7.43E-10	1.99E-08	0.95	5.13	8.25	66.25
NUT	NUT1207	2.4672192	2.33E-15	2.61E-13	3.03	16.74	27.75	219.00
CUT	CUT441	2.4683483	6.01E-07	6.95E-06	0.69	3.67	5.75	48.75

TABLE S1: Differential expression data for RRP6 RNA-Seq dataset Page 321

Class	Transcript name	RRP6 KO_vs_WT log2_fold_change	RRP6 KO_vs_WT p-value	RRP6 KO_vs_WT FDR	Ave Norm Reads in WT	Ave Norm Reads in RRP6	Average RAW read counts in WT	Average RAW read counts in RRP6
CUT	CUT337	2.4689193	8.97E-09	1.79E-07	0.82	4.50	7.25	56.75
AST	AS_YCR068W	2.4690044	7.49E-11	2.72E-09	1.44	8.18	14.00	107.75
AST	AS_YNL102W	2.4695002	0.0002869	0.0015422	0.17	1.05	1.75	13.50
NUT	NUT0226	2.4707886	1.32E-06	1.39E-05	0.27	1.74	3.00	23.25
AST	AS_YEL010W	2.471479	8.60E-05	0.0005336	0.22	1.22	2.00	16.00
CUT	CUT694	2.4724851	5.47E-08	8.54E-07	1.03	5.57	8.75	71.00
CUT	CUT701	2.4725593	2.76E-10	8.32E-09	2.14	11.90	19.25	152.75
AST	AS_YGR241C	2.4730997	2.11E-05	0.0001551	0.49	2.90	5.00	38.00
AST	AS_YNR046W	2.4738595	2.80E-05	0.0001987	0.35	1.90	3.00	24.75
NUT	NUT0944	2.4741224	4.50E-09	9.75E-08	0.62	3.40	5.50	43.75
CUT	CUT579	2.4755163	3.03E-06	2.87E-05	0.68	3.71	6.00	51.75
AST	AS_YDR335W	2.4809032	0.0001751	0.0009995	0.17	1.07	1.75	13.75
CUT	CUT802	2.4819726	1.02E-05	8.16E-05	0.20	1.22	2.00	15.75
ORF-T	YJL067W	2.4852081	4.81E-05	0.0003228	0.30	1.50	2.25	18.75
CUT	CUT190	2.4876646	6.82E-07	7.77E-06	0.46	2.81	4.75	34.50
AST	AS_YLR453C	2.4881773	3.62E-11	1.42E-09	1.23	6.99	11.50	91.75
NUT	NUT0971	2.4885899	0.0001707	0.0009795	0.14	0.78	1.25	10.25
AST	AS_YGR277C	2.4918059	9.88E-06	7.96E-05	0.33	1.85	3.00	25.00
NUT	NUT0904	2.4932205	1.49E-06	1.55E-05	0.47	2.56	4.00	33.25
NUT	NUT1047	2.4934724	1.35E-08	2.58E-07	0.85	4.74	7.50	61.00
CUT	CUT498	2.4935984	1.34E-06	1.41E-05	0.47	2.56	4.00	33.25
CUT	CUT748	2.4949102	8.93E-08	1.31E-06	1.35	7.78	12.75	98.50
sn/snoRNA ET	SNR32-ET	2.4958473	9.49E-20	2.13E-17	20.41	115.08	184.50	1463.00
ORF-T	YGL182C	2.4960249	1.11E-05	8.82E-05	0.28	1.56	2.50	20.50
CUT	CUT411	2.4972135	3.67E-08	6.07E-07	0.50	2.80	4.50	37.00
AST	AS_YJR122W	2.4975263	1.30E-09	3.31E-08	1.88	10.85	18.25	138.50
CUT	CUT182	2.4978019	1.86E-10	5.93E-09	1.41	8.08	13.25	108.50
AST	AS_YLR148W	2.4982762	2.86E-08	4.91E-07	0.59	3.41	5.50	43.50
NUT	NUT0714	2.4986708	5.68E-12	2.79E-10	1.81	10.40	17.25	136.00

Class	Transcript name	RRP6 KO_vs_WT log2_fold _change	RRP6 KO_vs_WT p-value	RRP6 KO_vs_WT FDR	Ave Norm Reads in WT	Ave Norm Reads in RRP6	Average RAW read counts in WT	Average RAW read counts in RRP6
AST	AS_YFR029W	2.4988832	4.37E-07	5.24E-06	0.68	3.91	6.50	53.75
NUT	NUT1098	2.4989272	1.21E-10	4.07E-09	1.12	6.09	9.50	79.00
NUT	NUT1101	2.4996721	3.64E-08	6.04E-07	0.54	3.05	5.00	42.00
CUT	CUT843	2.5000425	6.88E-05	0.0004407	0.25	1.29	2.00	16.75
AST	AS_YHR122W	2.5015642	1.12E-06	1.21E-05	0.29	1.69	2.75	22.50
NUT	NUT0857	2.5023219	3.94E-07	4.78E-06	0.74	4.11	6.50	56.00
SRT	SRT300	2.5050452	3.41E-08	5.71E-07	0.50	2.81	4.50	37.25
SRT	SRT361	2.5071418	4.74E-06	4.22E-05	0.41	2.38	3.75	28.75
AST	AS_YOR123C	2.5072926	1.01E-07	1.46E-06	0.36	2.05	3.25	26.25
NUT	NUT1408	2.5076333	0.0002111	0.0011791	0.21	1.35	2.25	18.25
CUT	CUT301	2.5077678	2.19E-07	2.85E-06	0.85	4.79	7.50	61.75
AST	AS_YDR207C	2.5088914	8.02E-06	6.65E-05	0.27	1.65	2.75	22.50
AST	AS_YDR118W	2.5107869	3.44E-05	0.000239	0.27	1.47	2.25	18.50
NUT	NUT0557	2.5134401	2.30E-11	9.26E-10	1.23	7.11	11.50	93.25
AST	AS_YKL080W	2.515845	6.76E-09	1.39E-07	0.88	5.21	8.50	67.00
AST	AS_YOR177C	2.5199604	3.67E-05	0.0002529	0.22	1.28	2.00	16.25
CUT	CUT010	2.5205978	3.52E-06	3.26E-05	0.49	2.65	4.00	34.50
CUT	CUT101	2.5226307	1.55E-05	0.0001181	0.21	1.27	2.00	16.50
AST	AS_YNR045W	2.5230799	3.96E-08	6.52E-07	0.62	3.71	6.00	48.50
NUT	NUT0803	2.5239172	1.64E-09	4.07E-08	0.93	5.29	8.25	66.75
CUT	CUT617	2.5276068	4.57E-05	0.0003077	0.20	1.15	1.75	14.25
AST	AS_YNL054W	2.52818	2.41E-10	7.36E-09	1.27	7.48	12.25	102.00
CUT	CUT654	2.5287374	8.06E-11	2.89E-09	1.48	8.62	13.75	108.50
ORF-T	YLR437C-A	2.534007	2.96E-05	0.000209	0.24	1.31	2.00	17.00
CUT	CUT790	2.5355253	2.88E-08	4.93E-07	0.42	2.39	3.75	31.75
NUT	NUT0957	2.5358598	1.28E-07	1.79E-06	0.90	5.59	9.75	76.75
CUT	CUT111	2.5359569	8.34E-05	0.0005195	0.19	1.11	1.75	15.00
AST	AS_YHR039C-A	2.5370477	0.000234	0.0012954	0.19	1.10	1.75	15.25
NUT	NUT1350	2.5374106	5.39E-09	1.15E-07	1.23	7.17	11.25	91.50

Class	Transcript name	RRP6 KO_vs_WT log2_fold_change	RRP6 KO_vs_WT p-value	RRP6 KO_vs_WT FDR	Ave Norm Reads in WT	Ave Norm Reads in RRP6	Average RAW read counts in WT	Average RAW read counts in RRP6
CUT	CUT585	2.5378023	7.04E-10	1.90E-08	1.79	10.58	17.25	135.25
CUT	CUT907	2.542124	1.52E-07	2.08E-06	0.75	4.17	6.25	55.75
CUT	CUT819	2.5445559	2.41E-07	3.09E-06	0.38	2.37	3.75	30.75
AST	AS_YOL138C	2.5496716	4.34E-06	3.91E-05	0.56	3.14	4.75	44.00
NUT	NUT1347	2.550997	1.22E-06	1.29E-05	0.54	3.19	5.00	42.75
AST	AS_YGR006W	2.5510488	1.76E-08	3.22E-07	0.77	4.45	6.75	57.50
CUT	CUT150	2.5511942	1.90E-11	7.76E-10	3.83	22.59	35.25	283.50
CUT	CUT807	2.5542815	0.00029	0.0015544	0.12	0.92	1.50	12.00
SRT	SRT48	2.5561957	8.14E-06	6.73E-05	0.20	1.29	2.00	16.75
NUT	NUT0538	2.5563659	2.77E-06	2.66E-05	0.29	1.64	2.50	21.50
AST	AS_YGR244C	2.5566275	9.10E-06	7.45E-05	0.22	1.41	2.25	18.75
NUT	NUT0334	2.5568024	0.0001964	0.0011052	0.19	1.11	1.75	15.50
CUT	CUT860	2.5612944	1.66E-05	0.0001253	0.21	1.16	1.75	15.25
CUT	CUT587	2.5619101	6.18E-09	1.29E-07	1.25	7.02	10.00	94.00
CUT	CUT319	2.5619163	1.86E-07	2.47E-06	0.62	3.58	5.50	49.00
AST	AS_YJR017C	2.5621854	1.54E-08	2.87E-07	0.62	3.61	5.50	47.25
CUT	CUT033	2.5625721	1.96E-05	0.0001456	0.26	1.61	2.50	21.00
NUT	NUT0571	2.5628068	3.84E-09	8.55E-08	0.71	4.34	7.00	60.75
NUT	NUT0974	2.5644075	0.0004868	0.0024669	0.13	0.80	1.25	11.00
CUT	CUT650	2.564698	9.87E-06	7.96E-05	0.23	1.33	2.00	17.25
CUT	CUT201	2.565674	2.42E-07	3.09E-06	0.33	2.08	3.25	27.25
CUT	CUT107	2.5667621	1.30E-11	5.68E-10	1.17	7.06	11.00	92.50
AST	AS_YMR183C	2.5685453	4.26E-06	3.85E-05	0.41	2.42	3.75	33.75
AST	AS_YLR380W	2.5701945	2.38E-06	2.33E-05	0.29	1.75	2.75	24.25
CUT	CUT646	2.5729017	8.48E-08	1.25E-06	0.35	2.12	3.25	27.75
AST	AS_YBR073W	2.5737717	8.84E-06	7.26E-05	0.27	1.63	2.50	21.75
NUT	NUT0966	2.5746043	1.99E-07	2.62E-06	0.63	3.81	5.75	48.75
CUT	CUT550	2.5750924	3.11E-07	3.87E-06	0.58	3.48	5.25	44.50
CUT	CUT870	2.5753245	7.12E-05	0.0004532	0.21	1.19	1.75	15.00

Class	Transcript name	RRP6 KO_vs_WT log2_fold_change	RRP6 KO_vs_WT p-value	RRP6 KO_vs_WT FDR	Ave Norm Reads in WT	Ave Norm Reads in RRP6	Average RAW read counts in WT	Average RAW read counts in RRP6
CUT	CUT236	2.575978	4.91E-08	7.77E-07	0.44	2.52	3.75	32.75
sn/snoRNA ET	SNR81-ET	2.5760001	6.08E-10	1.69E-08	1.68	9.91	14.50	123.25
sn/snoRNA ET	SNR60-ET	2.5761808	6.00E-13	3.81E-11	28.26	168.37	250.50	2242.75
CUT	CUT468	2.5764222	7.39E-13	4.51E-11	1.78	10.67	16.25	135.75
NUT	NUT0420	2.5769319	0.0001018	0.0006204	0.13	0.95	1.50	12.75
NUT	NUT0333	2.576961	2.27E-12	1.20E-10	2.21	13.28	20.50	175.75
NUT	NUT0730	2.5776676	8.08E-05	0.0005059	0.20	1.18	1.75	15.00
AST	AS_YFR023W	2.5780797	0.0002746	0.0014872	0.17	1.12	1.75	14.75
CUT	CUT140	2.5795392	1.39E-06	1.46E-05	0.29	1.66	2.50	22.25
AST	AS_YLR299W	2.5817379	6.23E-10	1.71E-08	1.05	6.30	9.50	83.75
AST	AS_YGL030W	2.5826725	0.0001532	0.0008953	0.20	1.18	1.75	15.25
AST	AS_YOR280C	2.5826914	8.19E-08	1.22E-06	0.40	2.35	3.50	30.75
NUT	NUT1104	2.5827541	1.16E-10	3.96E-09	1.38	8.38	13.00	111.00
ORF-T	YDR042C	2.5830034	7.92E-13	4.76E-11	4.76	28.68	44.50	371.25
sn/snoRNA ET	SNR34-ET*	2.5831482	3.94E-10	1.14E-08	1.36	8.16	12.50	109.75
AST	AS_YPL127C	2.5839493	2.99E-08	5.10E-07	0.75	4.48	6.75	59.00
CUT	CUT786	2.5873108	4.71E-07	5.60E-06	0.64	3.81	5.75	53.50
NUT	NUT0533	2.5885163	1.96E-11	7.98E-10	1.75	10.62	16.25	135.50
CUT	CUT294	2.5898272	4.18E-08	6.82E-07	0.56	3.12	4.50	40.25
NUT	NUT0395	2.5903568	1.02E-05	8.20E-05	0.39	2.43	3.75	32.25
SUT	SUT467	2.5905467	4.57E-08	7.32E-07	1.14	6.78	10.00	91.00
AST	AS_YNL288W	2.5930339	2.69E-06	2.59E-05	0.62	3.93	6.25	50.25
AST	AS_YGL216W	2.5939169	7.73E-06	6.46E-05	0.22	1.34	2.00	17.25
AST	AS_YJR141W	2.5940102	2.63E-08	4.58E-07	0.46	2.56	3.75	34.25
AST	AS_YKL185W	2.5940465	5.47E-15	5.80E-13	2.14	13.04	20.00	176.25
NUT	NUT1288	2.5945307	7.78E-08	1.17E-06	0.33	2.13	3.25	27.50
AST	AS_YDR177W	2.595672	0.0001369	0.0008105	0.19	1.04	1.50	13.50
AST	AS_YOR035C	2.5957289	9.92E-08	1.44E-06	0.32	2.10	3.25	27.75
NUT	NUT0378	2.5957551	2.31E-09	5.49E-08	0.85	5.19	8.00	68.50

TABLE S1: Differential expression data for RRP6 RNA-Seq dataset Page 325

Class	Transcript name	RRP6 KO_vs_WT log2_fold_change	RRP6 KO_vs_WT p-value	RRP6 KO_vs_WT FDR	Ave Norm Reads in WT	Ave Norm Reads in RRP6	Average RAW read counts in WT	Average RAW read counts in RRP6
AST	AS_YHR083W	2.5959246	1.96E-08	3.53E-07	0.47	2.80	4.25	38.25
CUT	CUT016	2.5966418	1.55E-07	2.12E-06	0.56	3.24	4.75	42.25
CUT	CUT508	2.5966611	1.85E-05	0.0001378	0.26	1.86	3.00	22.50
NUT	NUT0913	2.5966688	1.85E-05	0.0001378	0.26	1.86	3.00	22.50
AST	AS_YMR184W	2.6009903	5.41E-08	8.47E-07	1.17	7.16	10.75	88.25
AST	AS_YOL131W	2.6012225	1.48E-07	2.05E-06	0.61	3.80	5.75	49.25
AST	AS_YGL153W	2.6012527	1.19E-06	1.27E-05	0.30	1.83	2.75	24.50
NUT	NUT1249	2.6019083	7.89E-13	4.76E-11	1.72	10.52	16.00	136.75
CUT	CUT518	2.6032121	5.42E-06	4.75E-05	0.27	1.50	2.25	21.25
CUT	CUT226	2.6038139	4.83E-15	5.23E-13	4.01	24.62	37.75	311.25
NUT	NUT1311	2.6055386	4.18E-08	6.82E-07	1.19	7.32	11.00	90.25
AST	AS_YIL159W	2.6068441	2.67E-05	0.0001909	0.46	2.86	4.50	41.50
NUT	NUT1473	2.6082969	1.47E-05	0.0001128	0.25	1.39	2.00	17.75
CUT	CUT780	2.6083576	1.30E-07	1.83E-06	1.08	6.63	9.75	81.75
AST	AS_YPR147C	2.6086216	1.22E-05	9.55E-05	0.49	2.85	4.00	36.75
AST	AS_RPL15B	2.6091269	5.73E-09	1.21E-07	1.43	8.85	13.25	106.75
AST	AS_YHR172W	2.6094209	4.96E-14	4.20E-12	4.36	26.63	39.75	334.25
NUT	NUT0322	2.609751	7.79E-09	1.57E-07	0.61	3.50	5.00	45.50
CUT	CUT698	2.6107927	1.57E-08	2.92E-07	0.45	2.85	4.25	36.50
NUT	NUT0837	2.611047	3.97E-07	4.81E-06	0.67	4.09	6.00	53.25
SRT	SRT14	2.6126093	1.33E-07	1.86E-06	0.56	3.28	4.75	42.75
AST	AS_YOR350C	2.6128145	3.65E-07	4.47E-06	0.40	2.62	4.00	34.50
CUT	CUT507	2.6133236	1.15E-07	1.64E-06	0.59	3.54	5.25	48.00
NUT	NUT0593	2.6137787	4.41E-09	9.59E-08	1.43	8.88	13.25	107.25
CUT	CUT680	2.6151171	0.0001839	0.0010421	0.32	1.96	2.75	23.75
NUT	NUT1485	2.6154109	2.88E-08	4.93E-07	0.55	3.50	5.25	43.75
NUT	NUT0620	2.6161425	2.23E-10	6.89E-09	0.87	5.34	8.00	70.50
CUT	CUT458	2.6161755	4.19E-10	1.20E-08	0.69	4.21	6.25	55.75
AST	AS_YLR063W	2.61645	8.94E-13	5.24E-11	1.68	10.43	15.75	135.50

TABLE S1: Differential expression data for RRP6 RNA-Seq dataset Page 326

Class	Transcript name	RRP6 KO_vs_WT log2_fold _change	RRP6 KO_vs_WT p-value	RRP6 KO_vs_WT FDR	Ave Norm Reads in WT	Ave Norm Reads in RRP6	Average RAW read counts in WT	Average RAW read counts in RRP6
SUT	SUT382	2.6174738	3.46E-05	0.00024	0.29	1.87	2.75	22.75
CUT	CUT510	2.6179556	6.56E-07	7.51E-06	0.43	2.47	3.50	32.50
AST	AS_YHR067W	2.6185839	9.73E-11	3.40E-09	0.97	5.73	8.25	74.25
sn/snoRNA ET	SNR65-ET	2.6187483	9.67E-09	1.92E-07	3.79	23.37	34.25	283.25
CUT	CUT037	2.6198204	1.23E-05	9.61E-05	0.17	1.02	1.50	13.50
CUT	CUT475	2.6202654	3.90E-13	2.57E-11	3.74	22.72	31.75	275.75
ORF-T	YGR031C-A	2.6206595	8.12E-08	1.21E-06	0.32	2.26	3.50	29.50
AST	AS_YOL011W	2.6213902	1.17E-05	9.22E-05	0.24	1.39	2.00	18.00
NUT	NUT1118	2.6223772	5.38E-11	2.06E-09	0.93	5.67	8.25	74.75
CUT	CUT620	2.6227673	2.14E-05	0.0001568	0.37	2.15	3.00	28.00
CUT	CUT769	2.6235139	6.97E-05	0.0004455	0.17	1.15	1.75	15.50
CUT	CUT260	2.6237395	5.62E-14	4.69E-12	2.02	12.67	19.50	171.75
AST	AS_YJL149W	2.62477	1.04E-11	4.76E-10	1.59	9.84	14.75	132.25
CUT	CUT867	2.62778	7.67E-06	6.43E-05	0.25	1.53	2.25	20.25
CUT	CUT375	2.6297515	4.18E-10	1.20E-08	0.85	5.29	7.75	69.75
CUT	CUT439	2.6301966	1.58E-05	0.0001199	0.33	2.02	3.00	29.75
ORF-T	YOR072W-A	2.6309757	2.93E-16	3.94E-14	2.99	18.62	27.75	240.00
CUT	CUT541	2.6322994	6.51E-09	1.34E-07	0.60	3.84	5.75	49.50
AST	AS_YLR136C	2.6327567	1.62E-14	1.57E-12	2.53	15.88	24.25	215.75
NUT	NUT1254	2.6339015	1.21E-08	2.35E-07	0.54	3.41	5.00	43.50
NUT	NUT0930	2.6344135	5.78E-10	1.61E-08	0.84	5.17	7.50	67.75
NUT	NUT1113	2.6346722	2.48E-14	2.36E-12	4.32	26.80	39.00	336.25
AST	AS_YLL046C	2.6347913	0.0001643	0.0009507	0.12	0.71	1.00	9.00
CUT	CUT342	2.6354103	1.18E-08	2.32E-07	0.58	3.60	5.25	47.50
CUT	CUT911	2.6367157	5.26E-07	6.16E-06	0.30	2.10	3.25	28.50
NUT	NUT0042	2.6373139	9.76E-10	2.54E-08	0.85	5.36	8.00	68.75
NUT	NUT1025	2.6385076	7.11E-05	0.000453	0.16	1.05	1.50	13.25
NUT	NUT0954	2.6392411	1.33E-09	3.37E-08	0.67	4.35	6.50	56.25
CUT	CUT613	2.6400798	2.55E-09	5.99E-08	0.69	4.31	6.25	54.50

Class	Transcript name	RRP6 KO_vs_WT log2_fold _change	RRP6 KO_vs_WT p-value	RRP6 KO_vs_WT FDR	Ave Norm Reads in WT	Ave Norm Reads in RRP6	Average RAW read counts in WT	Average RAW read counts in RRP6
CUT	CUT545	2.6421922	4.23E-09	9.29E-08	0.56	3.68	5.50	47.25
CUT	CUT689	2.6446163	5.54E-05	0.000366	0.31	1.96	2.75	24.00
CUT	CUT753	2.6456649	9.32E-12	4.33E-10	1.08	6.89	10.25	92.25
AST	AS_YNL024C	2.6467145	2.24E-08	3.95E-07	0.84	5.61	8.75	72.00
NUT	NUT0200	2.6476943	1.72E-08	3.16E-07	0.57	3.60	5.25	47.50
CUT	CUT571	2.6514117	1.52E-07	2.08E-06	0.54	3.27	4.50	40.25
AST	AS_YNR008W	2.6561943	6.13E-07	7.08E-06	0.42	2.37	3.25	32.25
CUT	CUT447	2.6583889	2.07E-08	3.71E-07	0.49	3.31	5.00	44.00
NUT	NUT0281	2.6591184	5.19E-07	6.09E-06	0.39	2.40	3.50	34.00
NUT	NUT0606	2.6613343	2.44E-06	2.38E-05	0.29	1.77	2.50	23.75
AST	AS_YLR404W	2.6621882	9.53E-11	3.40E-09	0.94	5.99	8.75	81.25
AST	AS_YDR113C	2.6623915	5.52E-06	4.82E-05	0.18	1.22	1.75	16.00
NUT	NUT0592	2.6644007	2.59E-06	2.51E-05	0.22	1.54	2.25	19.50
CUT	CUT692	2.6646734	1.07E-06	1.15E-05	0.25	1.43	2.00	18.75
SRT	SRT425	2.6652539	6.41E-05	0.000415	0.16	1.05	1.50	14.25
CUT	CUT340	2.6652765	3.13E-10	9.33E-09	0.77	4.88	7.00	62.75
AST	AS_YKL099C	2.6666914	4.41E-06	3.96E-05	0.28	1.79	2.50	22.25
CUT	CUT300	2.6680452	7.71E-06	6.45E-05	0.20	1.38	2.00	17.50
NUT	NUT1488	2.6700438	6.16E-05	0.0004	0.20	1.49	2.25	18.75
NUT	NUT0940	2.6705414	1.12E-10	3.85E-09	0.72	4.48	6.25	57.25
AST	AS_YCR006C	2.6737236	1.68E-06	1.72E-05	0.26	1.62	2.25	20.50
AST	AS_YBR039W	2.675099	3.35E-05	0.0002335	0.25	1.60	2.25	21.00
NUT	NUT0301	2.6772854	6.41E-06	5.46E-05	0.37	2.52	3.75	32.50
CUT	CUT862	2.6835224	1.42E-05	0.0001096	0.15	1.05	1.50	14.00
AST	AS_YGR135W	2.6855091	7.42E-06	6.23E-05	0.43	2.73	3.75	35.50
CUT	CUT854	2.6863559	4.44E-11	1.71E-09	1.02	6.38	8.75	80.50
NUT	NUT0127	2.6888729	4.65E-06	4.14E-05	0.18	1.25	1.75	16.25
AST	AS_YPL039W	2.6903196	0.0001303	0.0007761	0.13	1.16	1.75	14.25
NUT	NUT0398	2.6948249	9.50E-08	1.38E-06	0.52	3.06	4.00	39.25

Class	Transcript name	RRP6 KO_vs_WT log2_fold _change	RRP6 KO_vs_WT p-value	RRP6 KO_vs_WT FDR	Ave Norm Reads in WT	Ave Norm Reads in RRP6	Average RAW read counts in WT	Average RAW read counts in RRP6
CUT	CUT912	2.6979951	2.72E-05	0.0001938	0.19	1.25	1.75	16.25
NUT	NUT0489	2.6998687	1.68E-11	7.07E-10	2.97	19.36	27.50	252.75
NUT	NUT0454	2.7008222	4.05E-06	3.68E-05	0.28	1.83	2.50	22.75
AST	AS_YHR092C	2.7008635	1.73E-06	1.77E-05	0.21	1.27	1.75	16.75
SUT	SUT817	2.7009392	3.45E-08	5.77E-07	0.45	3.15	4.50	39.25
AST	AS_YHR097C	2.7028376	0.0001242	0.000743	0.16	1.09	1.50	13.75
AST	AS_YDR517W	2.7031377	2.37E-06	2.32E-05	0.34	2.20	3.00	28.25
CUT	CUT364	2.704197	6.96E-05	0.000445	0.74	4.85	6.50	59.25
AST	AS_YCR063W	2.7042339	3.58E-09	8.05E-08	0.60	3.82	5.25	50.25
AST	AS_YHR104W	2.706913	3.09E-07	3.86E-06	0.47	3.25	4.75	43.50
NUT	NUT0076	2.7111338	4.38E-06	3.94E-05	0.22	1.47	2.00	18.50
AST	AS_YLR365W	2.7115795	3.58E-10	1.05E-08	1.15	7.58	10.50	94.25
CUT	CUT577	2.7119758	2.21E-08	3.92E-07	1.41	9.44	13.50	119.00
AST	AS_YJL026W	2.7124715	5.91E-10	1.64E-08	1.31	8.71	12.50	114.75
NUT	NUT1230	2.7137532	2.63E-08	4.58E-07	0.55	3.41	4.50	43.75
CUT	CUT906	2.7138736	2.81E-05	0.0001989	0.21	1.30	1.75	17.00
AST	AS_YJL062W-A	2.715657	7.84E-06	6.53E-05	0.21	1.28	1.75	17.25
CUT	CUT231	2.7160803	4.51E-10	1.29E-08	0.87	5.49	7.25	71.50
AST	AS_YBR127C	2.7185363	3.45E-07	4.24E-06	0.27	1.64	2.25	22.25
CUT	CUT644	2.7186765	9.73E-11	3.40E-09	0.79	5.02	6.75	64.50
NUT	NUT1401	2.7221727	6.13E-10	1.69E-08	0.48	3.20	4.50	43.75
NUT	NUT0878	2.7268775	5.29E-17	7.85E-15	5.81	38.40	52.25	470.25
AST	AS_YNL161W	2.7272085	2.56E-09	5.99E-08	0.82	5.47	7.50	70.50
AST	AS_YHL028W	2.7272186	2.60E-09	6.08E-08	0.48	3.36	4.75	43.75
SUT	SUT279	2.7293934	2.24E-12	1.19E-10	1.49	9.89	13.50	126.00
CUT	CUT547	2.7303432	1.62E-07	2.20E-06	0.82	5.56	7.75	69.50
AST	AS_YLR027C	2.7314776	3.87E-11	1.51E-09	2.67	17.80	24.75	232.50
CUT	CUT394	2.7327234	0.0001696	0.0009753	0.42	2.82	3.75	35.00
AST	AS_YDR336W	2.732976	2.58E-09	6.03E-08	0.62	3.95	5.25	51.75

Class	Transcript name	RRP6 KO_vs_WT log2_fold _change	RRP6 KO_vs_WT p-value	RRP6 KO_vs_WT FDR	Ave Norm Reads in WT	Ave Norm Reads in RRP6	Average RAW read counts in WT	Average RAW read counts in RRP6
AST	AS_YJL206C	2.7342509	6.26E-10	1.72E-08	0.74	5.11	7.25	67.50
CUT	CUT655	2.7357622	1.81E-12	9.79E-11	2.74	18.12	24.25	227.00
AST	AS_YMR152W	2.7380457	6.04E-05	0.0003941	0.16	1.11	1.50	14.50
NUT	NUT0644	2.7382018	1.08E-08	2.14E-07	0.40	2.76	3.75	35.00
NUT	NUT0869	2.7393032	7.21E-09	1.47E-07	0.74	4.96	6.75	65.00
NUT	NUT0709	2.7395026	2.70E-09	6.25E-08	0.49	3.30	4.50	43.50
NUT	NUT0544	2.7396584	3.06E-08	5.18E-07	0.44	2.79	3.75	37.25
AST	AS_YPR069C	2.7400007	0.0002454	0.0013502	0.10	0.75	1.00	9.50
NUT	NUT1062	2.7411944	1.84E-09	4.47E-08	0.73	4.64	6.00	57.75
AST	AS_YEL051W	2.7420465	9.96E-10	2.58E-08	0.57	3.85	5.25	49.50
NUT	NUT0757	2.7427899	1.05E-10	3.64E-09	0.88	5.91	8.00	73.75
AST	AS_YOR040W	2.7430874	9.99E-13	5.75E-11	51.39	343.99	456.75	4287.75
NUT	NUT1489	2.7452852	9.75E-13	5.65E-11	1.61	10.61	14.00	135.75
AST	AS_YGL060W	2.7456327	1.12E-06	1.20E-05	0.23	1.74	2.50	24.50
NUT	NUT0998	2.7504876	6.46E-05	0.0004172	0.17	1.11	1.50	15.75
NUT	NUT0314	2.7517592	3.15E-07	3.92E-06	0.33	2.50	3.50	30.75
CUT	CUT259	2.7518585	5.65E-06	4.90E-05	0.35	2.16	2.75	27.50
CUT	CUT776	2.7583167	1.49E-05	0.000114	0.17	1.13	1.50	15.00
AST	AS_YHR031C	2.7603312	1.63E-13	1.17E-11	1.86	12.69	17.25	168.25
CUT	CUT812	2.7620586	0.000115	0.0006935	0.15	0.97	1.25	12.50
NUT	NUT1470	2.7626665	1.58E-11	6.77E-10	0.70	4.82	6.50	61.75
CUT	CUT269	2.7629342	6.63E-05	0.0004271	0.14	0.97	1.25	12.00
ORF-T	YGR121W-A	2.7649597	3.96E-09	8.76E-08	0.35	2.43	3.25	31.50
AST	AS_YER071C	2.7670307	4.97E-06	4.39E-05	0.19	1.31	1.75	17.50
sn/snoRNA ET	SNR5-ET*	2.7684409	2.69E-10	8.17E-09	2.86	19.55	26.00	244.00
CUT	CUT594	2.7721372	1.19E-05	9.38E-05	0.36	2.36	3.00	30.75
sn/snoRNA ET	SNR49-ET	2.7732187	1.28E-12	7.20E-11	4.78	32.56	42.25	413.00
AST	AS_YHL019C	2.7735633	1.04E-09	2.67E-08	0.55	3.50	4.50	45.50
AST	AS_YNL035C	2.7745283	6.01E-11	2.26E-09	0.59	3.97	5.25	52.50

TABLE S1: Differential expression data for RRP6 RNA-Seq dataset Page 330

Class	Transcript name	RRP6 KO_vs_WT log2_fold _change	RRP6 KO_vs_WT p-value	RRP6 KO_vs_WT FDR	Ave Norm Reads in WT	Ave Norm Reads in RRP6	Average RAW read counts in WT	Average RAW read counts in RRP6
NUT	NUT0187	2.7768016	5.75E-08	8.94E-07	0.38	2.86	4.00	37.50
CUT	CUT869	2.784065	8.62E-07	9.57E-06	0.39	2.55	3.25	32.50
CUT	CUT401	2.7845023	3.43E-06	3.18E-05	0.27	1.92	2.50	24.00
SRT	SRT183	2.7864391	2.66E-06	2.56E-05	0.14	1.10	1.50	15.00
CUT	CUT288	2.7870671	3.75E-06	3.44E-05	0.20	1.48	2.00	20.75
sn/snoRNA ET	snR61-ET	2.7874309	1.12E-13	8.35E-12	13.06	90.22	117.50	1094.75
CUT	CUT733	2.791822	7.13E-13	4.41E-11	1.33	9.27	12.25	120.00
CUT	CUT609	2.7974507	1.70E-07	2.29E-06	0.29	1.97	2.50	24.50
NUT	NUT1112	2.8004116	1.34E-08	2.57E-07	0.48	3.28	4.25	43.75
CUT	CUT207	2.8008253	2.74E-05	0.0001948	0.25	1.86	2.50	24.50
AST	AS_YNL162W	2.8018272	1.21E-05	9.48E-05	0.16	1.32	1.75	16.50
AST	AS_YMR132C	2.8046186	1.26E-08	2.43E-07	0.66	4.52	5.75	58.50
CUT	CUT865	2.8062226	6.62E-07	7.56E-06	0.29	1.95	2.50	26.50
CUT	CUT559	2.8064622	6.12E-10	1.69E-08	0.68	4.79	6.25	62.50
AST	AS_YOR144C	2.8065381	3.16E-06	2.98E-05	0.28	1.92	2.50	27.25
CUT	CUT409	2.8068293	1.87E-08	3.38E-07	0.29	2.11	2.75	27.75
CUT	CUT164	2.8076723	3.85E-05	0.0002635	0.24	1.70	2.25	22.75
ORF-T	YHL019W-A	2.8103648	1.45E-08	2.73E-07	0.46	3.01	3.75	38.75
CUT	CUT515	2.8114934	1.77E-08	3.23E-07	0.95	6.23	7.25	78.00
AST	AS_YML037C	2.8129544	9.41E-07	1.03E-05	0.25	1.88	2.50	26.00
AST	AS_YML013W	2.8131137	4.51E-13	2.94E-11	1.45	10.07	12.75	129.50
NUT	NUT1292	2.8131138	4.51E-13	2.94E-11	1.45	10.07	12.75	129.50
CUT	CUT112	2.8135894	5.98E-05	0.0003905	0.16	1.15	1.50	16.25
NUT	NUT1462	2.8151391	3.56E-25	1.20E-22	10.42	73.32	94.75	945.50
CUT	CUT278	2.8162402	4.99E-12	2.48E-10	0.72	5.27	7.00	68.75
CUT	CUT170	2.8172781	7.13E-07	8.08E-06	0.27	2.11	2.75	25.75
AST	AS_YEL030W	2.8173426	2.72E-09	6.28E-08	0.51	3.85	5.25	53.25
NUT	NUT0282	2.8176647	8.24E-08	1.22E-06	0.62	4.59	6.25	58.75
ORF-T	YPL250W-A	2.8176889	0.0001122	0.0006778	0.17	1.03	1.25	13.25

Class	Transcript name	RRP6 KO_vs_WT log2_fold _change	RRP6 KO_vs_WT p-value	RRP6 KO_vs_WT FDR	Ave Norm Reads in WT	Ave Norm Reads in RRP6	Average RAW read counts in WT	Average RAW read counts in RRP6
NUT	NUT0914	2.8177559	3.22E-08	5.42E-07	0.46	3.03	3.75	40.25
CUT	CUT044	2.8197511	3.27E-10	9.65E-09	1.58	11.17	14.25	139.25
CUT	CUT358	2.8217368	1.29E-06	1.36E-05	0.39	2.83	3.75	38.25
CUT	CUT772	2.8221271	1.43E-08	2.73E-07	0.66	4.79	6.25	60.50
ORF-T	YJL048C	2.8231969	2.13E-18	3.97E-16	19.80	140.08	179.50	1866.75
CUT	CUT730	2.8243316	3.16E-06	2.98E-05	0.21	1.57	2.00	20.00
AST	AS_YLR295C	2.8244644	1.36E-09	3.45E-08	0.73	5.34	7.00	69.25
NUT	NUT0576	2.8259074	8.72E-10	2.31E-08	0.88	5.97	7.25	79.50
NUT	NUT1035	2.8267453	3.17E-11	1.26E-09	0.64	4.67	6.00	58.75
AST	AS_YNL191W	2.8267519	2.33E-07	3.00E-06	0.37	2.70	3.50	36.00
sn/snoRNA ET	SNR9-ET	2.8277811	1.17E-13	8.67E-12	37.99	269.66	340.00	3379.00
AST	AS_YPR093C	2.8307683	1.87E-09	4.53E-08	0.70	4.73	5.75	59.25
SRT	SRT532	2.8311197	2.60E-06	2.51E-05	0.38	2.86	3.75	36.75
AST	AS_YMR032W	2.8317746	6.41E-06	5.46E-05	0.20	1.40	1.75	17.75
CUT	CUT586	2.8319109	6.89E-08	1.05E-06	0.39	2.73	3.50	36.75
AST	AS_YBR047W	2.8327216	3.46E-05	0.0002401	0.15	1.01	1.25	13.00
SUT	SUT427	2.8327275	1.11E-05	8.80E-05	0.18	1.37	1.75	17.75
AST	AS_YPL232W	2.8333487	4.52E-08	7.26E-07	0.41	3.07	4.00	40.25
AST	AS_YBR261C	2.8344669	1.32E-05	0.0001025	0.13	0.97	1.25	13.00
AST	AS_YGR087C	2.8352931	4.27E-08	6.92E-07	0.60	4.50	6.00	57.50
sn/snoRNA ET	SNR71-ET	2.8443664	1.21E-19	2.66E-17	3.91	28.10	35.75	353.75
AST	AS_YGL081W	2.8451456	6.63E-11	2.43E-09	0.61	4.51	5.75	56.75
CUT	CUT892	2.846209	2.21E-05	0.000162	0.18	1.21	1.50	15.75
AST	AS_YLL039C	2.8478682	4.33E-05	0.0002929	0.16	1.02	1.25	13.75
AST	AS_YBR122C	2.8501838	6.82E-07	7.77E-06	0.36	2.70	3.50	35.00
AST	AS_YCR046C	2.8505044	2.52E-06	2.45E-05	0.39	2.68	3.25	35.00
AST	AS_YDR416W	2.8506567	3.37E-11	1.33E-09	0.59	4.09	5.00	51.75
CUT	CUT880	2.8511525	3.34E-08	5.59E-07	0.41	3.12	4.00	40.50
CUT	CUT026	2.8517954	1.84E-09	4.47E-08	0.49	3.68	4.75	47.25

Class	Transcript name	RRP6 KO_vs_WT log2_fold _change	RRP6 KO_vs_WT p-value	RRP6 KO_vs_WT FDR	Ave Norm Reads in WT	Ave Norm Reads in RRP6	Average RAW read counts in WT	Average RAW read counts in RRP6
NUT	NUT0708	2.8522216	5.60E-11	2.12E-09	0.80	5.65	7.00	74.75
AST	AS_YGR084C	2.85413	8.76E-08	1.29E-06	0.32	2.33	3.00	32.75
AST	AS_YBL098W	2.8543356	7.43E-08	1.12E-06	0.23	1.75	2.25	23.75
AST	AS_YCL029C	2.855151	3.79E-06	3.48E-05	0.21	1.43	1.75	18.75
NUT	NUT0022	2.8555581	1.42E-13	1.05E-11	3.61	26.06	32.25	339.00
AST	AS_YER130C	2.8572283	1.20E-06	1.28E-05	0.26	1.79	2.25	25.50
NUT	NUT1461	2.8575297	4.32E-09	9.48E-08	0.32	2.35	3.00	32.25
NUT	NUT0164	2.862163	6.18E-07	7.12E-06	0.51	3.79	4.75	48.75
CUT	CUT598	2.8633146	6.44E-11	2.38E-09	0.61	4.57	5.75	57.50
AST	AS_YPL038W	2.8638728	1.29E-11	5.66E-10	0.85	6.11	7.50	78.00
CUT	CUT653	2.8694522	8.48E-05	0.0005267	0.09	0.81	1.00	10.25
NUT	NUT1146	2.8714369	6.43E-13	4.03E-11	0.93	6.60	8.00	86.25
NUT	NUT0456	2.8761154	3.62E-11	1.42E-09	0.99	7.02	8.25	91.25
AST	AS_YNL138W-A	2.8787434	2.17E-06	2.15E-05	0.30	2.11	2.50	27.25
AST	AS_YIL003W	2.8793896	8.74E-13	5.18E-11	0.89	6.26	7.50	81.25
CUT	CUT913	2.8852092	4.17E-06	3.78E-05	0.17	1.25	1.50	15.75
SRT	SRT599	2.8852105	4.18E-06	3.78E-05	0.17	1.25	1.50	15.75
AST	AS_YDR225W	2.8867852	5.19E-07	6.09E-06	0.22	1.45	1.75	19.75
CUT	CUT143	2.8876922	2.51E-13	1.75E-11	3.35	24.69	30.00	329.50
AST	AS_YKL026C	2.8909036	1.06E-06	1.15E-05	0.17	1.23	1.50	16.25
AST	AS_YJL198W	2.8931669	1.62E-11	6.90E-10	0.74	5.39	6.50	70.00
AST	AS_YDL182W	2.8933261	1.85E-17	2.87E-15	4.86	35.86	42.50	437.75
AST	AS_YOR354C	2.8944842	2.24E-08	3.95E-07	0.75	5.45	6.50	71.00
AST	AS_YER169W	2.8956471	2.90E-10	8.68E-09	0.82	5.93	7.00	78.75
AST	AS_YPL077C	2.8957998	4.47E-07	5.34E-06	0.23	1.66	2.00	22.25
CUT	CUT504	2.8985694	2.16E-06	2.15E-05	0.21	1.48	1.75	18.75
CUT	CUT114	2.9035464	1.46E-08	2.76E-07	0.28	2.22	2.75	29.75
AST	AS_YGR093W	2.9054128	1.65E-09	4.09E-08	0.55	3.89	4.50	48.00
CUT	CUT560	2.9085325	2.24E-07	2.90E-06	0.24	2.02	2.50	25.75

Class	Transcript name	RRP6 KO_vs_WT log2_fold_change	RRP6 KO_vs_WT p-value	RRP6 KO_vs_WT FDR	Ave Norm Reads in WT	Ave Norm Reads in RRP6	Average RAW read counts in WT	Average RAW read counts in RRP6
CUT	CUT721	2.9092338	3.10E-06	2.93E-05	0.17	1.28	1.50	16.00
AST	AS_YJR050W	2.9108555	1.44E-08	2.73E-07	0.59	4.31	5.00	55.25
AST	AS_YDR279W	2.9115263	3.11E-07	3.87E-06	0.27	1.87	2.25	26.25
NUT	NUT0747	2.9137486	1.79E-08	3.26E-07	0.75	5.53	6.50	71.75
AST	AS_YEL047C	2.9151578	1.49E-08	2.80E-07	0.33	2.76	3.50	36.25
CUT	CUT685	2.920161	7.65E-11	2.76E-09	0.75	5.64	6.75	72.50
AST	AS_YPR116W	2.9214339	1.24E-06	1.31E-05	0.20	1.49	1.75	19.00
CUT	CUT487	2.9227884	1.20E-11	5.29E-10	0.77	5.72	6.75	75.75
SRT	SRT500	2.9234527	5.07E-10	1.42E-08	0.58	4.55	5.50	57.75
AST	AS_YGL189C	2.9240253	4.23E-11	1.63E-09	1.08	8.06	9.50	108.75
SUT	SUT418	2.9259036	4.32E-06	3.90E-05	0.19	1.47	1.75	19.00
CUT	CUT149	2.9262885	4.52E-10	1.29E-08	0.73	5.64	6.75	72.00
NUT	NUT1152	2.9263907	3.67E-10	1.07E-08	0.40	3.25	4.00	43.00
CUT	CUT354	2.9288945	4.47E-07	5.34E-06	0.29	2.12	2.50	28.25
sn/snoRNA ET	SNR46-ET	2.936229	1.43E-17	2.32E-15	31.69	242.46	281.00	3004.75
CUT	CUT379	2.9368796	4.47E-14	3.89E-12	0.97	7.71	9.50	100.50
AST	AS_YOL033W	2.9393492	9.68E-11	3.40E-09	0.40	3.11	3.75	42.25
CUT	CUT614	2.9412629	7.45E-09	1.51E-07	0.33	2.41	2.75	30.25
CUT	CUT361	2.9422316	2.92E-07	3.66E-06	0.20	1.49	1.75	19.75
CUT	CUT396	2.9446114	1.75E-08	3.21E-07	0.54	4.11	4.75	53.25
CUT	CUT791	2.9463388	1.92E-10	6.08E-09	0.69	5.44	6.50	68.00
AST	AS_YPL180W	2.9480908	7.04E-10	1.90E-08	0.45	3.58	4.25	45.50
CUT	CUT022	2.9490916	9.79E-10	2.55E-08	0.37	2.96	3.50	38.00
sn/snoRNA ET	SNR63-ET	2.9568794	6.68E-16	8.32E-14	6.49	50.29	58.50	673.75
AST	AS_YMR039C	2.9587325	1.20E-10	4.06E-09	0.77	5.65	6.25	75.00
AST	AS_YER070W	2.9635708	4.76E-13	3.08E-11	1.18	9.21	10.75	122.00
NUT	NUT0216	2.9643426	1.92E-10	6.08E-09	0.40	3.36	4.00	43.75
NUT	NUT0850	2.9659282	7.49E-11	2.72E-09	1.85	14.37	16.25	181.25
CUT	CUT765	2.9665697	1.81E-06	1.83E-05	0.25	2.12	2.50	26.75

Class	Transcript name	RRP6 KO_vs_WT log2_fold_change	RRP6 KO_vs_WT p-value	RRP6 KO_vs_WT FDR	Ave Norm Reads in WT	Ave Norm Reads in RRP6	Average RAW read counts in WT	Average RAW read counts in RRP6
CUT	CUT248	2.9670167	3.01E-08	5.14E-07	0.30	2.22	2.50	28.50
CUT	CUT834	2.9677264	0.0001152	0.0006943	0.14	1.09	1.25	15.00
CUT	CUT905	2.9690695	9.06E-13	5.28E-11	0.81	6.03	6.75	78.50
CUT	CUT011	2.9697408	2.26E-15	2.58E-13	2.28	17.70	20.00	230.25
NUT	NUT0254	2.9699089	9.27E-06	7.55E-05	0.15	1.08	1.25	15.00
CUT	CUT456	2.9708748	7.21E-11	2.63E-09	1.85	14.42	16.25	181.75
NUT	NUT0374	2.9709005	6.73E-07	7.68E-06	0.15	1.28	1.50	17.00
AST	AS_YJL066C	2.9713268	6.69E-09	1.37E-07	0.42	2.97	3.25	38.00
NUT	NUT0274	2.9716231	2.16E-10	6.71E-09	0.70	5.60	6.50	71.50
CUT	CUT116	2.9726496	4.71E-08	7.49E-07	0.30	2.33	2.75	33.00
AST	AS_YHR005C-A	2.9727925	1.57E-10	5.06E-09	0.68	5.55	6.50	70.00
AST	AS_YGL075C	2.9734321	5.26E-09	1.12E-07	0.29	2.19	2.50	29.00
AST	AS_YJR073C	2.9735193	6.35E-10	1.74E-08	0.50	4.12	5.00	55.75
CUT	CUT637	2.9838656	1.01E-09	2.61E-08	0.33	2.63	3.00	33.75
CUT	CUT042	2.9881181	2.42E-06	2.36E-05	0.21	1.55	1.75	22.50
NUT	NUT0892	2.9889023	1.68E-12	9.18E-11	0.88	6.85	7.75	91.00
AST	AS_YKL059C	2.9897501	4.22E-12	2.16E-10	0.90	6.88	7.50	89.25
CUT	CUT612	2.9932149	2.38E-09	5.64E-08	0.38	2.92	3.25	38.00
CUT	CUT012	2.995654	1.67E-09	4.12E-08	1.05	8.53	10.00	111.50
AST	AS_YER035W	2.9961334	1.58E-11	6.77E-10	0.71	5.81	6.75	73.75
AST	AS_YBR133C	2.9997974	9.32E-12	4.33E-10	1.98	16.06	18.50	195.00
CUT	CUT536	3.0014024	1.27E-10	4.24E-09	0.35	2.69	3.00	34.50
NUT	NUT0185	3.0035243	3.25E-06	3.04E-05	0.17	1.36	1.50	17.50
CUT	CUT295	3.0043393	2.16E-08	3.84E-07	0.39	2.98	3.25	40.50
AST	AS_YGL037C	3.0062566	3.02E-13	2.06E-11	3.96	31.77	35.50	426.50
AST	AS_YBR035C	3.0080173	5.33E-15	5.71E-13	2.89	23.24	26.25	303.00
CUT	CUT522	3.0081477	4.05E-10	1.17E-08	0.50	3.87	4.25	50.75
CUT	CUT049	3.0091657	4.92E-08	7.77E-07	0.35	2.58	2.75	33.25
AST	AS_YGR002C	3.0120927	1.78E-06	1.80E-05	0.19	1.69	2.00	23.25

Class	Transcript name	RRP6 KO_vs_WT log2_fold _change	RRP6 KO_vs_WT p-value	RRP6 KO_vs_WT FDR	Ave Norm Reads in WT	Ave Norm Reads in RRP6	Average RAW read counts in WT	Average RAW read counts in RRP6
AST	AS_YML070W	3.013733	1.09E-05	8.68E-05	0.18	1.53	1.75	20.75
AST	AS_YER083C	3.0138661	9.46E-06	7.68E-05	0.16	1.33	1.50	18.50
NUT	NUT0646	3.0145815	9.91E-06	7.97E-05	0.16	1.36	1.50	17.00
CUT	CUT800	3.0150532	3.54E-07	4.34E-06	0.29	2.29	2.50	28.50
NUT	NUT0783	3.016289	1.06E-07	1.53E-06	0.21	1.77	2.00	23.75
AST	AS_YGR092W	3.0170311	2.67E-08	4.62E-07	0.27	2.24	2.50	28.75
ORF-T	YCL007C	3.0184098	1.09E-11	4.94E-10	0.69	5.49	6.00	69.50
NUT	NUT1526	3.0238331	9.08E-10	2.40E-08	0.50	4.19	4.75	54.00
AST	AS_YLR334C	3.025569	2.20E-10	6.82E-09	0.47	4.11	4.75	53.25
ORF-T	YPR170C	3.0265501	1.56E-07	2.14E-06	0.21	1.62	1.75	20.50
AST	AS_YJL167W	3.0279321	1.32E-07	1.85E-06	0.23	1.85	2.00	23.00
AST	AS_YGL210W	3.0296358	5.66E-09	1.19E-07	0.41	3.36	3.75	45.00
NUT	NUT1155	3.0302898	1.24E-12	7.01E-11	0.74	5.92	6.50	77.00
NUT	NUT0969	3.031759	1.06E-11	4.81E-10	0.76	6.39	7.25	81.50
sn/snoRNA ET	SNR13-ET	3.0348226	5.77E-15	6.06E-13	9.37	76.75	82.75	953.75
CUT	CUT901	3.0406053	8.02E-12	3.82E-10	0.68	5.48	6.00	70.25
NUT	NUT0936	3.0408864	5.15E-09	1.10E-07	0.25	2.20	2.50	29.25
CUT	CUT813	3.0419274	2.07E-07	2.72E-06	0.20	1.61	1.75	20.75
AST	AS_YGR059W	3.0446918	8.73E-09	1.75E-07	0.33	2.57	2.75	32.25
ORF-T	YPL222C-A	3.0464308	1.93E-23	5.41E-21	5.68	46.81	51.00	602.75
AST	AS_YNR017W	3.0468059	1.52E-14	1.49E-12	0.69	5.97	6.75	78.25
AST	AS_YPR079W	3.0469739	2.33E-05	0.0001697	0.12	0.94	1.00	12.00
CUT	CUT343	3.0500094	9.60E-09	1.91E-07	0.36	2.99	3.25	38.75
NUT	NUT0259	3.0508526	1.49E-09	3.76E-08	0.29	2.31	2.50	30.75
CUT	CUT626	3.0514565	1.44E-08	2.73E-07	1.12	9.39	10.25	115.50
SRT	SRT465	3.0537031	2.20E-05	0.0001612	0.13	1.11	1.25	15.75
NUT	NUT1255	3.0609353	1.03E-13	7.73E-12	1.09	8.96	9.50	115.00
CUT	CUT399	3.0634446	1.72E-11	7.19E-10	0.65	5.51	6.00	68.50
AST	AS_YLR173W	3.0638297	9.89E-14	7.55E-12	1.09	8.98	9.50	115.25

TABLE S1: Differential expression data for RRP6 RNA-Seq dataset Page 336

Class	Transcript name	RRP6 KO_vs_WT log2_fold _change	RRP6 KO_vs_WT p-value	RRP6 KO_vs_WT FDR	Ave Norm Reads in WT	Ave Norm Reads in RRP6	Average RAW read counts in WT	Average RAW read counts in RRP6
CUT	CUT699	3.063936	4.54E-12	2.29E-10	0.89	7.52	8.25	95.75
NUT	NUT0338	3.0642254	8.95E-05	0.000554	0.10	0.73	0.75	9.50
NUT	NUT1359	3.066305	3.21E-08	5.42E-07	0.23	1.87	2.00	24.50
SUT	SUT535	3.0683714	6.46E-12	3.11E-10	1.11	9.33	10.00	120.00
AST	AS_YDR425W	3.06933	6.04E-17	8.71E-15	1.49	12.58	13.75	164.50
AST	AS_YMR016C	3.070167	6.34E-11	2.35E-09	0.44	3.94	4.50	55.00
NUT	NUT1057	3.0702289	7.96E-09	1.61E-07	0.33	2.62	2.75	32.75
NUT	NUT1010	3.0720928	6.53E-13	4.06E-11	1.30	10.84	11.50	140.50
AST	AS_YNL315C	3.0743186	2.11E-07	2.74E-06	0.21	1.83	2.00	24.25
AST	AS_YER080W	3.0779746	1.62E-10	5.21E-09	0.38	3.24	3.50	42.75
NUT	NUT0144	3.0813698	4.95E-06	4.39E-05	0.17	1.45	1.50	17.50
NUT	NUT0813	3.0815069	6.13E-11	2.28E-09	0.55	4.68	5.00	59.50
CUT	CUT689*	3.0828209	4.69E-18	8.30E-16	16.82	142.49	153.25	1904.25
AST	AS_YPR135W	3.0866234	3.17E-10	9.39E-09	0.36	3.05	3.25	39.75
CUT	CUT736	3.0943854	8.62E-09	1.73E-07	0.27	2.53	2.75	32.25
AST	AS_YBR196C-B	3.0972354	2.30E-06	2.26E-05	0.19	1.81	2.00	23.25
CUT	CUT830	3.0983813	4.63E-08	7.38E-07	0.28	2.37	2.50	31.25
AST	AS_YNL088W	3.099707	1.68E-08	3.10E-07	0.22	1.89	2.00	24.75
CUT	CUT832	3.1027919	7.43E-09	1.51E-07	0.25	2.12	2.25	29.25
NUT	NUT0596	3.1089808	2.26E-13	1.58E-11	0.86	7.28	7.50	95.75
CUT	CUT761	3.1096958	6.26E-08	9.67E-07	0.15	1.62	1.75	20.75
CUT	CUT252	3.1103089	2.94E-14	2.67E-12	1.06	9.19	9.75	120.00
AST	AS_YLR270W	3.1107281	4.35E-08	7.02E-07	0.19	1.83	2.00	25.25
CUT	CUT059	3.1121673	4.41E-09	9.59E-08	0.28	2.41	2.50	30.75
CUT	CUT667	3.1162262	1.32E-12	7.34E-11	0.73	5.97	6.00	77.00
CUT	CUT455	3.1183348	2.66E-09	6.18E-08	0.23	1.96	2.00	25.00
AST	AS_YMR138W	3.120019	2.09E-07	2.74E-06	0.17	1.46	1.50	19.25
SRT	SRT29	3.1215746	2.07E-12	1.11E-10	1.01	8.75	9.00	109.00
CUT	CUT051	3.1219446	9.93E-05	0.0006083	0.10	0.74	0.75	10.00

TABLE S1: Differential expression data for RRP6 RNA-Seq dataset Page 337

Class	Transcript name	RRP6 KO_vs_WT log2_fold_change	RRP6 KO_vs_WT p-value	RRP6 KO_vs_WT FDR	Ave Norm Reads in WT	Ave Norm Reads in RRP6	Average RAW read counts in WT	Average RAW read counts in RRP6
CUT	CUT261	3.125852	1.67E-11	7.03E-10	0.66	5.85	6.25	78.75
AST	AS_YOL078W	3.1305877	4.13E-07	4.97E-06	0.27	2.25	2.25	28.25
NUT	NUT0874	3.130847	6.93E-10	1.88E-08	0.36	3.14	3.25	41.25
NUT	NUT1308	3.1334015	4.13E-07	4.97E-06	0.17	1.47	1.50	19.50
CUT	CUT737	3.1362634	1.03E-13	7.73E-12	0.91	7.88	8.00	100.50
ORF-T	YCR040W	3.13747	2.81E-11	1.12E-09	0.91	7.81	7.75	97.00
NUT	NUT0417	3.1377708	4.80E-12	2.40E-10	0.64	5.84	6.25	78.50
AST	AS_YPR164W	3.1379115	8.81E-13	5.19E-11	0.75	6.56	6.75	85.25
AST	AS_YPL057C	3.1388062	3.05E-08	5.18E-07	0.24	2.17	2.25	29.00
sn/snoRNA ET	SNR62-ET	3.1414188	2.49E-23	6.79E-21	6.68	58.37	57.25	734.00
NUT	NUT1460	3.1417406	2.01E-14	1.93E-12	1.09	9.70	10.00	127.50
AST	AS_YBR030W	3.1493893	4.92E-09	1.06E-07	0.40	3.51	3.50	45.00
NUT	NUT1184	3.149827	2.98E-10	8.89E-09	0.37	3.37	3.50	43.50
AST	AS_YLR094C	3.1502458	8.14E-07	9.10E-06	0.20	1.93	2.00	24.50
NUT	NUT1521	3.1508613	5.01E-13	3.22E-11	0.75	6.61	6.75	86.00
CUT	CUT804	3.1534333	1.37E-05	0.0001056	0.12	1.01	1.00	13.00
CUT	CUT925	3.1571271	6.49E-10	1.77E-08	0.45	4.14	4.25	53.25
AST	AS_YPL191C	3.160946	4.64E-08	7.38E-07	0.17	1.49	1.50	19.50
CUT	CUT593	3.161864	1.82E-05	0.0001366	0.11	1.02	1.00	12.75
SUT	SUT465	3.1630138	1.58E-10	5.11E-09	1.12	9.99	10.00	124.50
AST	AS_YNL173C	3.1631079	3.75E-05	0.000258	0.13	1.24	1.25	15.50
AST	AS_YDR156W	3.163772	1.51E-08	2.82E-07	0.28	2.30	2.25	30.25
SRT	SRT529	3.1642956	1.11E-05	8.80E-05	0.20	1.90	2.00	25.50
CUT	CUT788	3.1642973	1.11E-05	8.80E-05	0.20	1.90	2.00	25.50
NUT	NUT1149	3.1674366	1.80E-11	7.48E-10	0.47	4.10	4.00	52.00
CUT	CUT233	3.1703041	7.19E-13	4.42E-11	0.50	4.48	4.50	58.75
AST	AS_YEL071W	3.1703981	4.70E-10	1.33E-08	0.29	2.54	2.50	31.50
NUT	NUT1339	3.172192	2.58E-07	3.27E-06	0.20	1.76	1.75	23.50
CUT	CUT449	3.1743637	2.19E-12	1.17E-10	0.76	6.77	6.75	90.25

Class	Transcript name	RRP6 KO_vs_WT log2_fold _change	RRP6 KO_vs_WT p-value	RRP6 KO_vs_WT FDR	Ave Norm Reads in WT	Ave Norm Reads in RRP6	Average RAW read counts in WT	Average RAW read counts in RRP6
CUT	CUT273	3.1783646	3.78E-08	6.24E-07	0.27	2.53	2.50	31.25
ORF-T	YCR041W	3.1800225	1.86E-11	7.65E-10	0.91	8.04	7.75	99.75
CUT	CUT181	3.1824697	5.80E-16	7.44E-14	0.99	9.14	9.25	114.75
ORF-T	YOR041C	3.182694	1.27E-07	1.78E-06	0.22	1.81	1.75	23.50
NUT	NUT0952	3.1839192	1.51E-17	2.42E-15	1.67	15.01	14.75	190.00
NUT	NUT0896	3.1881067	1.32E-11	5.75E-10	1.17	10.61	10.50	132.75
NUT	NUT0881	3.1888914	1.60E-06	1.65E-05	0.13	1.03	1.00	13.75
AST	AS_YLR174W	3.1889249	1.91E-06	1.92E-05	0.09	1.00	1.00	13.25
ORF-T	YML094C-A	3.1923433	6.57E-07	7.51E-06	0.14	1.29	1.25	16.25
CUT	CUT040	3.1927057	9.30E-12	4.33E-10	0.43	3.81	3.75	49.50
CUT	CUT563	3.1946263	9.58E-09	1.91E-07	0.23	2.04	2.00	27.00
NUT	NUT0915	3.2001384	3.06E-05	0.0002158	0.12	1.04	1.00	14.25
CUT	CUT919	3.2020987	8.76E-12	4.13E-10	0.67	6.14	6.00	79.75
SRT	SRT526	3.2040828	2.46E-05	0.0001771	0.13	1.28	1.25	16.50
AST	AS_YOR025W	3.2066421	1.01E-06	1.11E-05	0.13	1.31	1.25	16.00
CUT	CUT840	3.2097891	6.25E-15	6.50E-13	3.47	31.46	28.25	407.75
ORF-T	YOR225W	3.2107449	3.94E-07	4.78E-06	0.20	1.83	1.75	23.25
AST	AS_YHR168W	3.2133439	6.24E-09	1.30E-07	0.26	2.33	2.25	30.75
AST	AS_YNL010W	3.2154995	8.47E-07	9.43E-06	0.18	1.57	1.50	21.50
CUT	CUT474	3.2162786	3.80E-08	6.27E-07	0.78	7.57	8.00	100.25
ORF-T	YCR064C	3.2183879	5.48E-11	2.09E-09	0.58	5.26	5.00	69.50
NUT	NUT1256	3.2185503	1.24E-06	1.31E-05	0.09	1.02	1.00	13.50
NUT	NUT0068	3.2234341	5.14E-12	2.54E-10	0.43	3.89	3.75	50.75
AST	AS_YLR263W	3.2238509	1.46E-14	1.45E-12	0.68	6.38	6.25	81.75
NUT	NUT0312	3.2254533	3.82E-05	0.0002623	0.08	0.80	0.75	10.25
SRT	SRT247	3.225731	9.59E-10	2.50E-08	0.30	2.86	2.75	36.75
AST	AS_YMR146C	3.2287634	1.66E-13	1.18E-11	0.72	6.61	6.25	87.00
AST	AS_YDR070C	3.237311	1.39E-05	0.0001072	0.09	0.82	0.75	10.25
CUT	CUT528	3.2374371	6.14E-09	1.29E-07	0.21	2.05	2.00	27.25

TABLE S1: Differential expression data for RRP6 RNA-Seq dataset Page 339

Class	Transcript name	RRP6 KO_vs_WT log2_fold_change	RRP6 KO_vs_WT p-value	RRP6 KO_vs_WT FDR	Ave Norm Reads in WT	Ave Norm Reads in RRP6	Average RAW read counts in WT	Average RAW read counts in RRP6
NUT	NUT1263	3.2380985	1.43E-14	1.43E-12	0.68	6.45	6.25	82.75
ORF-T	YLR364C-A	3.2386717	2.75E-10	8.32E-09	0.38	3.65	3.50	45.00
AST	AS_YLR366W	3.2417644	1.41E-10	4.65E-09	0.38	3.66	3.50	45.25
NUT	NUT0428	3.245853	1.85E-11	7.65E-10	0.34	3.37	3.25	43.00
CUT	CUT891	3.2463223	4.71E-09	1.01E-07	0.19	1.84	1.75	24.00
CUT	CUT818	3.2546425	6.44E-15	6.62E-13	0.50	4.91	4.75	64.50
CUT	CUT671	3.2572758	9.08E-11	3.25E-09	0.47	4.15	3.75	52.75
AST	AS_YOR332W	3.2577084	7.25E-05	0.0004606	0.12	1.09	1.00	14.75
SRT	SRT144	3.2587455	5.17E-08	8.13E-07	0.15	1.58	1.50	20.50
AST	AS_YPR113W	3.2658234	1.34E-08	2.57E-07	0.20	2.09	2.00	27.25
AST	AS_YOR358W	3.266746	1.80E-07	2.40E-06	0.12	1.30	1.25	17.50
AST	AS_YJR039W	3.2731398	1.70E-06	1.73E-05	0.11	1.08	1.00	14.00
NUT	NUT1508	3.2771955	6.24E-06	5.36E-05	0.12	1.11	1.00	14.00
AST	AS_YML079W	3.2783893	1.19E-08	2.32E-07	0.15	1.81	1.75	23.25
CUT	CUT921	3.2788778	8.22E-14	6.49E-12	0.78	7.23	6.50	91.25
AST	AS_YPL222W	3.2816775	1.46E-27	6.39E-25	6.79	65.88	61.00	850.50
ORF-T	YGL152C	3.2821663	2.10E-07	2.74E-06	0.10	1.31	1.25	17.00
NUT	NUT1305	3.2823103	4.24E-07	5.10E-06	0.17	1.42	1.25	17.50
AST	AS_YDL057W	3.2840007	5.65E-11	2.13E-09	0.32	3.17	3.00	43.00
CUT	CUT676	3.2870533	1.94E-08	3.50E-07	0.31	2.79	2.50	36.75
AST	AS_YIL093C	3.2877421	9.94E-16	1.21E-13	0.97	9.42	8.75	118.50
AST	AS_YHR086W-A	3.2921985	4.57E-08	7.32E-07	0.15	1.59	1.50	21.50
NUT	NUT0406	3.2954761	2.70E-09	6.25E-08	0.33	3.28	3.00	41.50
AST	AS_YER135C	3.2976696	4.59E-11	1.76E-09	0.51	4.97	4.50	62.00
sn/snoRNA ET	SNR59-ET	3.2983688	4.78E-12	2.40E-10	0.62	6.05	5.50	77.00
CUT	CUT308	3.3026771	1.26E-10	4.22E-09	0.74	7.20	6.50	91.50
NUT	NUT0457	3.3027895	2.49E-17	3.80E-15	0.94	9.26	8.50	121.50
AST	AS_YER093C-A	3.3046148	3.96E-09	8.76E-08	0.17	1.87	1.75	25.00
AST	AS_YPR168W	3.3062207	4.64E-14	4.00E-12	0.78	7.37	6.50	93.00

Class	Transcript name	RRP6 KO_vs_WT log2_fold _change	RRP6 KO_vs_WT p-value	RRP6 KO_vs_WT FDR	Ave Norm Reads in WT	Ave Norm Reads in RRP6	Average RAW read counts in WT	Average RAW read counts in RRP6
AST	AS_YDR320C	3.3088441	1.44E-13	1.05E-11	0.54	5.60	5.25	73.00
AST	AS_YPL250C	3.3147741	2.11E-06	2.10E-05	0.19	1.73	1.50	22.25
CUT	CUT659	3.3149716	7.74E-06	6.46E-05	0.12	1.11	1.00	15.75
AST	AS_YKL055C	3.3164817	8.49E-17	1.21E-14	0.91	9.05	8.25	118.50
CUT	CUT303	3.3194499	8.44E-13	5.04E-11	0.59	5.85	5.25	77.00
AST	AS_YJR006W	3.3227263	1.44E-10	4.74E-09	0.31	3.26	3.00	42.00
AST	AS_YOR051C	3.3246551	5.45E-13	3.48E-11	0.55	5.33	4.75	70.25
AST	AS_YIL042C	3.3262084	2.78E-07	3.51E-06	0.23	2.45	2.25	32.25
NUT	NUT1129	3.3290308	1.87E-06	1.88E-05	0.16	1.63	1.50	23.50
CUT	CUT041	3.3304263	1.07E-06	1.15E-05	0.12	1.14	1.00	15.00
AST	AS_YHR105W	3.3358695	8.24E-14	6.49E-12	0.41	4.16	3.75	54.25
NUT	NUT1033	3.3417331	9.25E-14	7.18E-12	0.63	6.47	5.75	81.25
SRT	SRT513	3.3425465	8.13E-06	6.73E-05	0.09	1.09	1.00	15.25
AST	AS_YPR174C	3.3464751	1.42E-11	6.11E-10	0.41	4.21	3.75	53.25
CUT	CUT188	3.3484223	2.27E-15	2.58E-13	0.82	8.39	7.50	105.75
AST	AS_YNL247W	3.3530921	6.16E-08	9.54E-07	0.18	1.73	1.50	23.00
AST	AS_YMR194W	3.3531697	3.46E-14	3.09E-12	1.26	12.93	11.50	163.75
NUT	NUT1315	3.3548023	3.14E-14	2.83E-12	1.26	12.95	11.50	164.00
CUT	CUT669	3.3589429	1.75E-11	7.28E-10	0.33	3.20	2.75	41.00
SUT	SUT503	3.374772	1.32E-14	1.33E-12	0.91	9.03	7.50	115.00
AST	AS_YLR389C	3.3807305	5.23E-19	1.08E-16	1.55	16.21	14.25	216.75
AST	AS_YGL032C	3.3820753	9.26E-07	1.02E-05	0.15	1.67	1.50	22.50
AST	AS_YDL202W	3.3836378	3.18E-10	9.39E-09	0.27	2.87	2.50	37.25
CUT	CUT482	3.3842299	4.54E-09	9.78E-08	0.24	2.58	2.25	34.75
ORF-T	YDR426C	3.3874851	1.06E-15	1.27E-13	0.96	10.12	8.75	129.50
CUT	CUT691	3.3899609	1.45E-07	2.01E-06	0.20	2.04	1.75	25.75
NUT	NUT0266	3.3955086	1.61E-15	1.86E-13	0.82	8.79	7.75	115.75
CUT	CUT472	3.3964671	1.72E-10	5.50E-09	0.26	2.88	2.50	37.50
NUT	NUT0707	3.4035407	3.47E-13	2.33E-11	0.40	4.57	4.00	57.50

Class	Transcript name	RRP6 KO_vs_WT log2_fold _change	RRP6 KO_vs_WT p-value	RRP6 KO_vs_WT FDR	Ave Norm Reads in WT	Ave Norm Reads in RRP6	Average RAW read counts in WT	Average RAW read counts in RRP6
AST	AS_YMR246W	3.4099854	2.80E-12	1.46E-10	0.39	4.29	3.75	57.00
AST	AS_YML128C	3.413621	1.63E-16	2.22E-14	0.53	5.81	5.00	74.25
AST	AS_YKR095W-A	3.4155799	3.49E-06	3.24E-05	0.07	0.88	0.75	12.00
CUT	CUT104	3.4211498	6.61E-08	1.01E-06	0.12	1.48	1.25	19.00
NUT	NUT0368	3.4279053	2.21E-07	2.87E-06	0.23	2.62	2.25	34.75
ORF-T	YKL183C-A	3.4316873	2.81E-07	3.54E-06	0.08	1.16	1.00	15.75
CUT	CUT351	3.4349993	4.23E-08	6.88E-07	0.14	1.53	1.25	19.75
AST	AS_YNL329C	3.4391679	1.92E-09	4.63E-08	0.22	2.61	2.25	35.00
CUT	CUT466	3.4429244	4.46E-14	3.89E-12	0.71	7.88	6.75	105.25
NUT	NUT0942	3.4462508	8.62E-18	1.50E-15	1.08	11.76	9.75	152.00
AST	AS_YNR054C	3.4508846	2.13E-08	3.81E-07	0.14	1.54	1.25	20.00
AST	AS_YJL218W	3.4512911	6.06E-11	2.26E-09	0.24	2.67	2.25	36.00
CUT	CUT881	3.4529436	1.56E-06	1.62E-05	0.10	0.93	0.75	12.50
CUT	CUT280	3.4548051	5.16E-17	7.77E-15	0.53	5.97	5.00	77.00
NUT	NUT1478	3.4576285	3.81E-06	3.49E-05	0.06	0.90	0.75	11.75
CUT	CUT173	3.4588836	2.71E-11	1.09E-09	0.37	4.21	3.50	53.25
AST	AS_YJL184W	3.4604148	2.61E-06	2.53E-05	0.10	0.95	0.75	12.25
SRT	SRT231	3.4620058	4.23E-08	6.88E-07	0.15	1.78	1.50	23.75
CUT	CUT213	3.4625064	2.45E-09	5.78E-08	0.27	3.05	2.50	38.75
CUT	CUT034	3.4659086	2.60E-08	4.53E-07	0.14	1.55	1.25	20.25
AST	AS_YLR355C	3.4706853	6.35E-06	5.44E-05	0.08	1.19	1.00	15.50
CUT	CUT762	3.4720573	2.94E-15	3.22E-13	0.54	6.45	5.50	84.50
NUT	NUT1398	3.4728609	1.08E-07	1.55E-06	0.20	2.19	1.75	27.50
AST	AS_YLR323C	3.473603	1.68E-06	1.72E-05	0.14	1.55	1.25	21.00
CUT	CUT486	3.4840133	3.67E-07	4.48E-06	0.11	1.28	1.00	15.50
CUT	CUT446	3.4929422	1.50E-10	4.88E-09	0.25	2.85	2.25	36.00
CUT	CUT568	3.4956811	1.39E-11	6.03E-10	0.47	4.95	3.75	61.75
AST	AS_YER133W	3.500316	1.19E-11	5.25E-10	0.47	4.96	3.75	62.00
NUT	NUT0460	3.5083015	1.90E-11	7.76E-10	0.81	9.38	7.75	134.75

Class	Transcript name	RRP6 KO_vs_WT log2_fold _change	RRP6 KO_vs_WT p-value	RRP6 KO_vs_WT FDR	Ave Norm Reads in WT	Ave Norm Reads in RRP6	Average RAW read counts in WT	Average RAW read counts in RRP6
CUT	CUT674	3.5130127	3.41E-12	1.77E-10	1.06	11.86	9.00	146.75
CUT	CUT142	3.5283837	4.40E-12	2.23E-10	0.71	8.26	6.50	102.00
NUT	NUT1286	3.5299351	1.73E-08	3.17E-07	0.18	1.95	1.50	25.25
AST	AS_YDL166C	3.5316276	5.97E-06	5.15E-05	0.10	0.99	0.75	13.25
CUT	CUT597	3.5378247	9.59E-14	7.38E-12	0.53	6.16	4.75	77.00
AST	AS_YHR060W	3.5390338	3.46E-06	3.21E-05	0.08	1.00	0.75	12.50
AST	AS_YKL029C	3.5429332	9.99E-12	4.58E-10	0.78	9.29	7.50	133.50
AST	AS_YBR055C	3.5442293	1.15E-10	3.92E-09	0.25	2.87	2.25	38.75
NUT	NUT0559	3.5442453	9.71E-18	1.66E-15	0.53	6.36	5.00	81.75
SRT	SRT348	3.5551242	3.14E-09	7.14E-08	0.23	2.63	2.00	35.25
NUT	NUT0095	3.5573626	5.18E-06	4.55E-05	0.10	1.01	0.75	13.50
AST	AS_YDR471W	3.5601116	1.60E-05	0.0001214	0.09	1.27	1.00	17.75
AST	AS_YGL172W	3.5664724	3.14E-05	0.0002204	0.06	0.93	0.75	13.00
sn/snoRNA ET	SNR31-ET	3.5794347	1.21E-18	2.30E-16	1.12	13.50	10.25	175.25
AST	AS_YIR032C	3.5974302	6.89E-06	5.83E-05	0.07	0.99	0.75	13.25
AST	AS_YOL117W	3.6021683	6.90E-08	1.05E-06	0.12	1.37	1.00	18.00
CUT	CUT809	3.6051902	1.08E-07	1.55E-06	0.15	1.74	1.25	22.50
AST	AS_YHR058C	3.6056633	1.73E-05	0.0001306	0.07	0.71	0.50	9.25
AST	AS_YNL204C	3.6095494	9.64E-11	3.40E-09	0.20	2.93	2.25	36.75
CUT	CUT008	3.6202438	9.95E-07	1.09E-05	0.10	1.40	1.00	16.50
AST	AS_YLR185W	3.6239502	2.61E-09	6.09E-08	0.21	2.15	1.50	28.00
NUT	NUT0288	3.6419209	1.19E-07	1.69E-06	0.21	2.76	2.00	34.25
CUT	CUT684	3.6456724	5.26E-08	8.26E-07	0.09	1.36	1.00	18.25
AST	AS_YBR106W	3.6485861	4.51E-06	4.04E-05	0.07	1.04	0.75	14.00
AST	AS_YAL024C	3.649123	5.62E-09	1.19E-07	0.16	2.08	1.50	26.75
AST	AS_YDR527W	3.6585133	5.59E-19	1.13E-16	1.31	16.53	11.75	205.25
CUT	CUT095	3.6611852	2.98E-09	6.80E-08	0.26	3.47	2.50	44.75
CUT	CUT532	3.6616914	7.05E-14	5.74E-12	0.30	3.59	2.50	45.25
NUT	NUT0970	3.6623877	8.27E-07	9.23E-06	0.12	1.44	1.00	19.50

Class	Transcript name	RRP6 KO_vs_WT log2_fold _change	RRP6 KO_vs_WT p-value	RRP6 KO_vs_WT FDR	Ave Norm Reads in WT	Ave Norm Reads in RRP6	Average RAW read counts in WT	Average RAW read counts in RRP6
AST	AS_YHR156C	3.677708	3.60E-19	7.72E-17	6.49	83.18	59.25	1026.00
AST	AS_YGR030C	3.6787387	6.12E-29	3.25E-26	8.64	110.98	79.75	1392.00
AST	AS_YDR127W	3.6809289	2.82E-15	3.13E-13	0.61	8.01	5.75	106.50
AST	AS_YDR398W	3.6849506	1.06E-09	2.70E-08	0.10	1.74	1.25	22.50
CUT	CUT817	3.6890011	3.70E-09	8.30E-08	0.15	2.08	1.50	29.50
CUT	CUT176	3.6929592	1.62E-07	2.21E-06	0.08	1.08	0.75	14.50
AST	AS_YML109W	3.6946964	1.11E-10	3.83E-09	0.23	3.15	2.25	42.00
AST	AS_YBR231C	3.6992521	3.71E-09	8.30E-08	0.14	1.83	1.25	23.75
AST	AS_YML094W	3.7205686	1.02E-08	2.02E-07	0.14	1.85	1.25	23.75
CUT	CUT915	3.742646	3.12E-09	7.12E-08	0.13	1.84	1.25	23.75
AST	AS_YDR410C	3.7646669	4.04E-09	8.91E-08	0.09	1.46	1.00	20.25
NUT	NUT1399	3.7689703	1.28E-15	1.50E-13	0.34	4.82	3.25	63.00
AST	AS_YOR048C	3.7854444	3.72E-09	8.30E-08	0.09	1.51	1.00	19.75
NUT	NUT0641	3.7856745	1.18E-11	5.22E-10	0.19	2.97	2.00	37.25
NUT	NUT0960	3.7879332	8.26E-08	1.22E-06	0.10	1.18	0.75	15.75
SRT	SRT534	3.7890241	6.46E-07	7.41E-06	0.08	1.18	0.75	15.25
CUT	CUT350	3.7928091	4.10E-16	5.36E-14	0.82	11.22	7.25	144.75
AST	AS_YER041W	3.8240602	3.40E-07	4.19E-06	0.12	1.62	1.00	22.00
CUT	CUT419	3.8305296	1.80E-08	3.26E-07	0.12	1.62	1.00	21.25
CUT	CUT542	3.8353739	2.63E-14	2.46E-12	0.38	5.24	3.25	65.50
CUT	CUT745	3.8356608	1.96E-12	1.06E-10	0.28	3.99	2.50	49.50
CUT	CUT060	3.8420562	6.87E-09	1.41E-07	0.12	1.65	1.00	20.75
CUT	CUT374	3.8459069	8.89E-14	6.95E-12	0.28	4.33	2.75	54.00
CUT	CUT711	3.8518792	2.55E-14	2.40E-12	0.23	3.24	2.00	41.50
CUT	CUT437	3.8610423	2.47E-07	3.16E-06	0.22	3.21	2.00	40.75
AST	AS_YGR180C	3.8636164	4.84E-08	7.68E-07	0.07	1.21	0.75	16.00
CUT	CUT789	3.8639482	1.06E-17	1.75E-15	0.34	5.18	3.25	67.50
CUT	CUT081	3.8673523	6.04E-13	3.81E-11	0.21	3.24	2.00	42.00
AST	AS_YOL121C	3.8679605	5.52E-09	1.17E-07	0.13	1.66	1.00	22.50

Class	Transcript name	RRP6 KO_vs_WT log2_fold _change	RRP6 KO_vs_WT p-value	RRP6 KO_vs_WT FDR	Ave Norm Reads in WT	Ave Norm Reads in RRP6	Average RAW read counts in WT	Average RAW read counts in RRP6
sn/snoRNA ET	SNR42-ET	3.8714915	1.82E-31	1.31E-28	8.85	129.35	79.25	1613.00
CUT	CUT372	3.8764558	9.41E-12	4.35E-10	0.14	2.06	1.25	27.00
CUT	CUT900	3.8880314	2.70E-07	3.42E-06	0.07	1.24	0.75	15.75
NUT	NUT0149	3.9050109	1.58E-07	2.15E-06	0.08	1.26	0.75	16.75
sn/snoRNA ET	SNR43-ET	3.9100231	1.38E-20	3.32E-18	1.43	21.29	12.50	259.75
CUT	CUT327	3.9116032	1.94E-10	6.10E-09	0.14	2.46	1.50	30.25
CUT	CUT799	3.9117445	1.09E-06	1.17E-05	0.06	0.88	0.50	11.25
AST	AS_YHR141C	3.9347475	1.32E-08	2.54E-07	0.30	4.62	2.75	60.25
SRT	SRT171	3.9376122	2.11E-10	6.59E-09	0.30	4.57	2.75	65.50
AST	AS_YCL005W-A	3.9387933	1.30E-20	3.20E-18	1.22	18.56	10.75	226.50
CUT	CUT147	3.938828	4.18E-26	1.56E-23	2.38	36.88	22.25	465.25
CUT	CUT168	3.9483677	1.10E-15	1.30E-13	0.49	7.15	4.00	89.75
CUT	CUT138	3.9620208	1.54E-16	2.13E-14	0.91	14.46	8.75	196.50
NUT	NUT0844	3.9675998	1.64E-09	4.07E-08	0.13	2.14	1.25	30.00
CUT	CUT782	3.9712403	2.07E-13	1.46E-11	0.22	3.48	2.00	46.25
CUT	CUT436	3.975178	1.19E-08	2.32E-07	0.12	1.80	1.00	23.50
CUT	CUT848	3.9785932	2.73E-14	2.53E-12	0.33	4.87	2.75	66.75
AST	AS_YMR128W	3.9842205	2.12E-08	3.79E-07	0.08	1.33	0.75	17.75
SRT	SRT321	3.993505	1.12E-08	2.21E-07	0.12	1.83	1.00	23.75
NUT	NUT0309	3.9962828	5.83E-16	7.44E-14	0.49	7.40	4.00	92.75
AST	AS_YIR014W	3.9996582	7.62E-12	3.66E-10	0.17	2.71	1.50	34.00
AST	AS_YGR273C	4.000416	8.38E-16	1.03E-13	0.46	6.92	3.75	87.00
AST	AS_YOL096C	4.001745	3.14E-08	5.32E-07	0.12	1.80	1.00	24.25
CUT	CUT703	4.0039235	2.77E-12	1.45E-10	0.17	3.03	1.75	42.50
AST	AS_YKL028W	4.0066495	6.40E-12	3.10E-10	0.17	2.30	1.25	29.75
AST	AS_YOR132W	4.0082899	1.45E-16	2.03E-14	0.45	6.82	3.75	93.25
CUT	CUT566	4.0226867	5.40E-17	7.89E-15	0.28	4.52	2.50	58.50
NUT	NUT0651	4.0252778	2.03E-08	3.65E-07	0.08	1.39	0.75	17.50
AST	AS_YLR219W	4.0286431	1.96E-09	4.72E-08	0.11	1.84	1.00	23.50

Class	Transcript name	RRP6 KO_vs_WT log2_fold _change	RRP6 KO_vs_WT p-value	RRP6 KO_vs_WT FDR	Ave Norm Reads in WT	Ave Norm Reads in RRP6	Average RAW read counts in WT	Average RAW read counts in RRP6
NUT	NUT1313	4.0357534	5.55E-14	4.66E-12	0.22	3.64	2.00	48.50
NUT	NUT1259	4.0407413	1.60E-09	3.99E-08	0.11	1.85	1.00	23.75
AST	AS_YER132C	4.0500533	9.74E-29	4.68E-26	1.17	20.13	11.50	262.25
AST	AS_YNL264C	4.0619028	1.45E-12	7.97E-11	0.16	2.77	1.50	36.50
NUT	NUT0817	4.0860463	3.21E-09	7.30E-08	0.12	1.95	1.00	25.25
CUT	CUT082	4.0894141	1.66E-06	1.69E-05	0.05	0.97	0.50	12.75
AST	AS_YGL181W	4.1147457	2.12E-06	2.11E-05	0.04	0.97	0.50	12.50
AST	AS_YMR117C	4.1163037	2.33E-07	3.00E-06	0.04	1.00	0.50	12.25
CUT	CUT725	4.1179804	2.64E-08	4.58E-07	0.07	1.44	0.75	19.25
CUT	CUT874	4.1210303	1.64E-07	2.23E-06	0.06	1.00	0.50	13.25
sn/snoRNA ET	SNR36-ET	4.1210915	8.90E-29	4.49E-26	2.93	50.58	25.50	633.50
NUT	NUT0981	4.1221634	6.39E-19	1.26E-16	0.31	5.34	2.75	69.75
CUT	CUT144	4.1262548	1.84E-17	2.87E-15	0.42	7.25	3.75	94.50
AST	AS_YKR061W	4.1310256	8.70E-31	5.48E-28	4.72	82.66	42.50	1051.50
NUT	NUT0556	4.1476292	7.93E-13	4.76E-11	0.14	2.48	1.25	32.00
AST	AS_YBL033C	4.1576922	3.91E-14	3.46E-12	0.20	3.86	2.00	52.00
NUT	NUT0629	4.1678781	2.85E-13	1.95E-11	0.16	2.99	1.50	39.00
sn/snoRNA ET	SNR11-ET	4.1684981	2.24E-34	2.59E-31	31.88	572.64	280.50	7307.25
AST	AS_YLR447C	4.2054305	4.17E-11	1.62E-09	0.12	2.10	1.00	27.00
CUT	CUT307	4.2155436	8.16E-19	1.58E-16	0.57	10.36	5.00	136.75
CUT	CUT744	4.2410272	1.46E-13	1.06E-11	0.15	3.13	1.50	38.00
CUT	CUT879	4.2465922	2.88E-27	1.21E-24	0.81	15.61	7.50	201.50
sn/snoRNA ET	SNR45-ET	4.2501574	1.39E-28	6.38E-26	7.23	136.68	62.00	1751.75
AST	AS_YJR072C	4.2772246	4.78E-10	1.35E-08	0.10	1.67	0.75	22.00
AST	AS_YER128W	4.2795531	2.66E-18	4.89E-16	0.24	4.84	2.25	62.75
CUT	CUT180	4.3275637	9.77E-25	3.08E-22	0.69	13.53	6.00	171.75
AST	AS_YMR194C-A	4.3286765	8.68E-23	2.25E-20	0.81	16.12	7.25	209.50
CUT	CUT720	4.3534758	3.42E-09	7.72E-08	0.06	1.18	0.50	15.50
sn/snoRNA ET	SNR35-ET	4.3698894	1.82E-31	1.31E-28	2.34	48.38	21.00	626.75

Class	Transcript name	RRP6 KO_vs_WT log2_fold _change	RRP6 KO_vs_WT p-value	RRP6 KO_vs_WT FDR	Ave Norm Reads in WT	Ave Norm Reads in RRP6	Average RAW read counts in WT	Average RAW read counts in RRP6
SRT	SRT31	4.3978214	5.38E-08	8.44E-07	0.05	1.18	0.50	16.25
AST	AS_YOR222W	4.4022235	3.39E-32	3.11E-29	2.25	47.61	20.25	617.00
AST	AS_YGL087C	4.4068127	1.57E-26	6.35E-24	5.36	113.82	48.75	1494.50
CUT	CUT335	4.4097981	1.46E-10	4.77E-09	0.07	1.75	0.75	23.75
CUT	CUT543	4.4304991	6.44E-20	1.48E-17	0.33	8.71	4.00	115.50
AST	AS_YMR194C-B	4.4558162	2.57E-34	2.59E-31	25.01	548.09	219.75	6993.75
CUT	CUT492	4.4560199	2.88E-18	5.18E-16	0.27	5.60	2.25	72.00
CUT	CUT306	4.4610987	2.44E-34	2.59E-31	24.99	549.64	219.50	7016.25
CUT	CUT596	4.4770409	1.70E-08	3.12E-07	0.05	1.27	0.50	16.25
CUT	CUT882	4.4802634	1.93E-20	4.53E-18	0.36	8.05	3.25	105.25
AST	AS_YGL093W	4.4957564	1.95E-08	3.52E-07	0.05	1.29	0.50	16.50
AST	AS_YNL110C	4.5363238	2.23E-11	9.03E-10	0.07	1.91	0.75	26.00
AST	AS_YBL072C	4.5833323	3.60E-13	2.40E-11	0.16	4.03	1.50	51.50
NUT	NUT1032	4.6221519	5.08E-09	1.09E-07	0.05	1.40	0.50	18.25
AST	AS_YOR276W	4.6282455	1.70E-22	4.28E-20	0.32	7.62	2.75	100.25
AST	AS_YLR028C	4.6643178	9.79E-24	2.82E-21	0.76	19.15	6.75	262.75
CUT	CUT232	4.6812369	5.18E-19	1.08E-16	0.17	4.30	1.50	55.50
CUT	CUT317	4.6914142	1.06E-12	6.07E-11	0.08	2.20	0.75	28.75
AST	AS_YOR184W	4.7325409	3.56E-40	1.20E-36	1.33	35.25	12.00	446.75
AST	AS_YDL075W	4.7440001	2.17E-08	3.86E-07	0.06	1.57	0.50	20.00
AST	AS_YOR042W	4.7605677	4.24E-32	3.57E-29	1.78	46.74	14.75	591.75
CUT	CUT831	4.8370969	1.03E-17	1.74E-15	0.13	3.98	1.25	51.50
AST	AS_YPL038W-A	4.8521146	5.88E-07	6.82E-06	0.03	0.88	0.25	11.50
CUT	CUT858	4.9694902	1.68E-29	9.39E-27	0.35	10.47	3.00	138.00
CUT	CUT822	4.9841175	7.67E-08	1.15E-06	0.03	0.96	0.25	12.50
sn/snoRNA ET	SNR3-ET	4.9891805	1.47E-35	2.96E-32	20.58	652.51	178.25	8646.50
AST	AS_YDR158W	4.9965493	6.27E-12	3.06E-10	0.06	2.62	0.75	33.00
sn/snoRNA ET	SNR30-ET	5.0150519	3.04E-24	9.00E-22	0.59	18.95	5.25	259.75
CUT	CUT365	5.0269517	3.42E-16	4.54E-14	0.14	4.57	1.25	58.00

Class	Transcript name	RRP6 KO_vs_WT log2_fold _change	RRP6 KO_vs_WT p-value	RRP6 KO_vs_WT FDR	Ave Norm Reads in WT	Ave Norm Reads in RRP6	Average RAW read counts in WT	Average RAW read counts in RRP6
AST	AS_YDL156W	5.037313	1.62E-08	3.00E-07	0.03	0.98	0.25	13.25
CUT	CUT565	5.0615018	5.90E-08	9.16E-07	0.03	1.01	0.25	13.50
AST	AS_YGL108C	5.0729912	2.73E-08	4.72E-07	0.03	1.00	0.25	13.75
CUT	CUT221	5.0737358	1.22E-40	6.17E-37	11.80	396.17	102.50	5205.25
CUT	CUT853	5.0849587	2.22E-37	5.61E-34	1.05	35.22	9.25	441.00
CUT	CUT137	5.1271354	5.66E-26	2.04E-23	0.89	31.62	8.50	433.25
CUT	CUT433	5.1272036	9.88E-31	5.86E-28	1.55	53.08	13.25	692.25
CUT	CUT539	5.1501298	3.64E-26	1.41E-23	0.32	11.75	3.00	145.00
sn/snoRNA ET	SNR37-ET	5.263105	1.22E-25	4.23E-23	0.38	14.76	3.50	204.00
CUT	CUT681	5.3522458	7.93E-25	2.58E-22	0.31	13.31	3.00	184.50
CUT	CUT223	5.4212495	5.67E-23	1.51E-20	0.47	20.12	4.25	281.50
CUT	CUT117	5.4576182	5.61E-31	3.77E-28	0.26	11.04	2.25	143.50
CUT	CUT920	5.5272295	5.33E-10	1.49E-08	0.03	1.42	0.25	17.75
CUT	CUT222	5.7733861	5.57E-35	9.36E-32	5.56	301.17	46.75	4054.50
CUT	CUT841	5.8432598	1.84E-34	2.59E-31	0.47	24.89	3.75	317.00
AST	AS_YBR103W	6.0651712	3.10E-13	2.10E-11	0.03	2.01	0.25	27.75
AST	AS_YJL104W	6.4475838	1.37E-12	7.57E-11	0.03	2.62	0.25	36.75
CUT	CUT274	6.4736526	6.26E-16	7.89E-14	0.02	2.66	0.25	34.25
AST	AS_YEL020W-A	8.2613666	3.48E-09	7.84E-08	0.00	0.88	0.00	11.75
CUT	CUT397	8.2663213	1.19E-08	2.32E-07	0.00	0.88	0.00	12.00
ORF-T	YEL020C-B	8.3245317	2.21E-09	5.27E-08	0.00	0.92	0.00	12.25
