# Supplementary material for: The Exosome Component Rrp6 Is Required for RNA Polymerase II Termination at Specific Targets of the Nrd1-Nab3 Pathway
Source: PLoS Genet. 2015 Feb 13;11(2):e1004999. doi: 10.1371/journal.pgen.1004999 (PMC4378619; doi:10.1371/journal.pgen.1004999)
Supplement: S2 Table — Table includes GO identification number (“Best GOs”), number of hits from GOStat analysis of our down-regulated protein coding gene list matching the GO ID (“Count”), total number of genes assigned the GO ID (“Total”), p-value for the GO term, and the descriptor for the GO ID. Information calculated using GOstat, as described in the methods. (PDF) [file pgen.1004999.s007.pdf]

Supplemental Table 2: Top 30 GO-terms enriched in the down-regulated protein coding gene dataset

Best GOs (Max: 30)	Count 927	Total 6476	P-Value	Ontology
GO:0005830	115	175	6.43E-82	Cytosolic Ribosome
GO:0044445	120	196	6.71E-77	Cytosolic Part (Cytosol Component)
GO:0003735	122	230	9.32E-62	Structural Constituent of Ribosome
GO:0033279	123	240	8.96E-59	Ribosome
GO:0005842	68	97	1.37E-52	Cytosolic Large Ribosomal Subunit
GO:0005840	139	353	2.12E-40	Ribosome
GO:0015934	76	142	3.29E-38	Large Ribosomal Subunit
GO:0005843	45	64	2.49E-34	Cytosolic Small Ribosomal Subunit
GO:0043228	259	1032	1.65E-24	Non-Membrane-Bound Organelle
GO:0043232	259	1032	1.65E-24	Intracellular Non-Membrane-Bound Organelle
GO:0030529	177	622	1.38E-23	Ribonucleoprotein Complex
GO:0015935	47	98	7.85E-19	Small Ribosomal Subunit
GO:0009277	46	114	5.68E-13	Fungal-Type Cell Wall
GO:0005618	46	114	5.68E-13	Cell Wall
GO:0030312	46	114	5.68E-13	External Encapsulating Structure
GO:0005829	145	623	4.07E-09	Cytosol
GO:0044422	420	2309	5.66E-09	Organelle Part
GO:0044446	420	2309	5.66E-09	Intracellular Organelle Part
GO:0043229	666	4023	6.50E-09	Intracellular Organelle
GO:0043226	666	4024	6.63E-09	Organelle
GO:0044249	209	1009	3.93E-08	Cellular Biosynthetic Process
GO:0032991	322	1705	4.54E-08	Macromolecular Complex
GO:0044464	846	5490	3.67E-07	Cell Part
GO:0006412	145	687	9.57E-06	Translation
GO:0005737	602	3699	2.15E-05	Cytoplasm
GO:0009058	236	1251	3.37E-05	Biosynthetic Process
GO:0044444	458	2717	6.65E-05	Cytoplasmic Part
GO:0009987	729	4653	6.65E-05	Cellular Process
GO:0005199	12	19	0.000102	Structural Constituent of Cell Wall
GO:0030150	13	22	0.000104	Protein Import into Mitochondrial Matrix
