# Supplementary material for: The Exosome Component Rrp6 Is Required for RNA Polymerase II Termination at Specific Targets of the Nrd1-Nab3 Pathway
Source: PLoS Genet. 2015 Feb 13;11(2):e1004999. doi: 10.1371/journal.pgen.1004999 (PMC4378619; doi:10.1371/journal.pgen.1004999)
Supplement: S3 Table — Table includes differential expression data expressed in log2 rrp6Δ/WT ratio (i.e. fold change) as well as p-values and false discovery rate (FDR), all calculated from four replicates by the EdgeR program as discussed in the methods. Table also includes standard gene names (or acronyms) for clarity. Significantly downregulated transcripts are in red-shaded cells whereas upregulated sn/snoRNAs are in green-shaded cells. (PDF) [file pgen.1004999.s008.pdf]

Table S3: Differential expression information for the ribosomal protein coding transcripts Page 1

Transcript name	Standard gene name	RRP6 _KO_ vs WT log2 FC	RRP6 _KO vs WT p-value	RRP6 _KO vs_WT FDR
YLR367W	RPS22B	-1.992743	1.27E-06	1.34E-05
YGL189C	RPS26A	-1.234646	4.08E-04	2.11E-03
YDR012W	RPL4B	-1.224361	2.50E-05	1.80E-04
YOL040C	RPS15	-1.187806	1.48E-04	0.000871
YOL039W	RPP2A	-1.18084	2.86E-04	1.54E-03
YKL180W	RPL17A	-1.178588	2.47E-04	1.36E-03
YLR185W	RPL37A	-1.138591	7.94E-05	0.000498
YDR418W	RPL12B	-1.112196	1.63E-05	0.000123
YBL092W	RPL32	-1.103358	0.0002222	0.001236
YHL033C	RPL8A	-1.096525	2.78E-05	0.000197
YER131W	RPS26B	-1.075197	1.52E-04	0.00089
YCR031C	RPS14A	-1.067605	0.0002575	0.001406
YOL127W	RPL25	-1.061722	0.0006462	0.00316
YMR242C	RPL20A	-1.035553	0.0010762	0.005013
YML073C	RPL6A	-1.032985	0.000519	0.002613
YPL249C-A	RPL36B	-0.983926	0.0006543	0.003197
YPL079W	RPL21B	-0.964466	0.0001339	0.000795
YFL034C-A	RPL22B	-0.955394	0.0012336	0.005662
YDR025W	RPS11A	-0.949811	0.0016071	0.007215
YIL052C	RPL34B	-0.948519	0.0009384	0.004451
YOL120C	RPL18A	-0.943612	0.0004191	0.002161
YLL045C	RPL8B	-0.943453	0.000723	0.00351
YNL096C	RPS7B	-0.938209	0.0005994	0.002957
YNL302C	RPS19B	-0.93574	0.0013151	0.006009
YER056C-A	RPL34A	-0.930519	0.0009908	0.004656
YER102W	RPS8B	-0.928462	6.59E-04	3.22E-03
YKL006W	RPL14A	-0.92515	1.58E-03	7.09E-03
YNL178W	RPS3	-0.901668	0.0025041	0.010605
YBL087C	RPL23A	-0.892906	0.002011	0.008786
YGL031C	RPL24A	-0.88995	0.0016174	0.007259
YLR048W	RPS0B	-0.885436	0.0023533	0.010064
YIL133C	RPL16A	-0.885136	0.0004631	0.002356
YPR043W	RPL43A	-0.884017	0.002664	0.011217
YOL121C	RPS19A	-0.883042	0.0010284	0.004808
YER074W	RPS24A	-0.87574	0.0058453	0.022194
YBR189W	RPS9B	-0.869864	0.0024119	0.010275
YDR500C	RPL37B	-0.866716	0.0017226	0.007679
YFR031C-A	RPL2A	-0.851989	0.005231	0.02018
YPL131W	RPL5	-0.843837	0.0045064	0.017737
YOR182C	RPS30B	-0.835264	0.0078704	0.028345
YDL083C	RPS16B	-0.834289	0.0044297	0.017518
YBL072C	RPS8A	-0.831343	0.0019911	0.008726
YLR333C	RPS25B	-0.819168	0.0034659	0.014144
YBR031W	RPL4A	-0.808244	0.0048695	0.018981
YOR312C	RPL20B	-0.806282	0.006965	0.025671
YLR061W	RPL22A	-0.803565	0.0086104	0.030498
YDL061C	RPS29B	-0.798657	0.0044449	0.01755
YLR448W	RPL6B	-0.798618	0.0047792	0.018658
YDR064W	RPS13	-0.791903	0.0052173	0.020134
YNL069C	RPL16B	-0.789254	0.0137802	0.045367
YIL069C	RPS24B	-0.787525	0.0060321	0.02279
YJL177W	RPL17B	-0.786743	0.0076389	0.027709
YBL027W	RPL19B	-0.786163	0.0135939	0.044915
YER117W	RPL23B	-0.778262	0.0095956	0.03347
YGR027C	RPS25A	-0.774556	0.0114579	0.039038

Table S3: Differential expression information for the ribosomal protein coding transcripts Page 2

Transcript name	Standard gene name	RRP6 _KO_ vs WT log2 FC	RRP6 _KO vs WT p-value	RRP6 _KO vs_WT FDR
YGL030W	RPL30	-0.772552	0.0080671	0.028908
YDL136W	RPL35B	-0.770191	0.0040663	0.016259
YLR340W	RPP0	-0.77013	0.0110787	0.037909
YOR096W	RPS7A	-0.764428	0.0204919	0.062694
YLR388W	RPS29A	-0.761878	0.0042301	0.016807
YLR325C	RPL38	-0.759297	0.0085087	0.030212
YDL075W	RPL31A	-0.755111	0.0065852	0.024522
YFR032C-A	RPL29	-0.754373	0.0444649	0.11698
YLR264W	RPS28B	-0.753698	0.0357407	0.098824
YDL130W	RPP1B	-0.751444	0.0019945	0.00873
YGR214W	RPS0A	-0.742393	0.010866	0.037321
YOR293W	RPS10A	-0.74227	0.014457	0.04721
YGR085C	RPL11B	-0.74147	0.0085608	0.030354
YLR029C	RPL15A	-0.731306	0.0227316	0.068083
YOR234C	RPL33B	-0.728371	0.0232336	0.069254
YDR450W	RPS18A	-0.727014	0.0146413	0.047688
YJR123W	RPS5	-0.725396	0.0133131	0.044146
YPL143W	RPL33A	-0.724666	0.0117044	0.0397
YMR142C	RPL13B	-0.724028	0.0118811	0.040184
YGL103W	RPL28	-0.721152	0.0154361	0.049858
YIL018W	RPL2B	-0.719007	0.0215832	0.065339
YHR203C	RPS4B	-0.717286	0.009648	0.03363
YKR094C	RPL40B	-0.713796	0.0177864	0.056102
YOR063W	RPL3	-0.711676	1.17E-02	3.96E-02
YGL147C	RPL9A	-0.708731	1.98E-02	6.10E-02
YNL162W	RPL42A	-0.701371	0.0140184	0.046031
YGL123W	RPS2	-0.699459	0.015224	0.049315
YIL148W	RPL40A	-0.69744	0.0256972	0.075447
YDL081C	RPP1A	-0.692989	0.0138284	0.0455
YPL090C	RPS6A	-0.692649	0.0128554	0.042953
YLR167W	RPS31	-0.692286	0.017123	0.054322
YMR194W	RPL36A	-0.690894	0.0145699	0.047502
YML024W	RPS17A	-0.685614	0.028975	0.083433
YLR441C	RPS1A	-0.682298	0.01404	0.046087
YHR141C	RPL42B	-0.675091	0.0171414	0.054357
YEL054C	RPL12A	-0.672709	0.0089589	0.031566
YML063W	RPS1B	-0.666632	0.0194106	0.060042
YLR406C	RPL31B	-0.666172	0.0144954	0.047289
YBR181C	RPS6B	-0.664573	0.0167511	0.053404
YDL191W	RPL35A	-0.662184	0.0153436	0.049623
YJL190C	RPS22A	-0.661559	0.0261498	0.076566
YHL015W	RPS20	-0.658424	0.019602	0.060523
YLR287C-A	RPS30A	-0.657464	0.017669	0.055767
YBR048W	RPS11B	-0.65009	0.0199817	0.061432
YDR471W	RPL27B	-0.644312	0.0258483	0.075794
YKR057W	RPS21A	-0.642271	0.0276957	0.080346
YGR148C	RPL24B	-0.642236	0.0182273	0.057082
YHL001W	RPL14B	-0.639806	0.0232776	0.069365
YDL082W	RPL13A	-0.634606	0.0175214	0.055387
YDR447C	RPS17B	-0.626113	0.029302	0.084242
YOR369C	RPS12	-0.624058	0.0206402	0.063052
YBR191W	RPL21A	-0.622635	0.0416636	0.111294
YGR034W	RPL26B	-0.61042	0.0178033	0.056138
YPR102C	RPL11A	-0.608627	0.0229386	0.068598
YLR344W	RPL26A	-0.603486	0.0192758	0.059735

Table S3: Differential expression information for the ribosomal protein coding transcripts Page 3

Transcript name	Standard gene name	RRP6 _KO_ vs WT log2 FC	RRP6 _KO vs WT p-value	RRP6 _KO vs_WT FDR
YHR010W	RPL27A	-0.601308	0.0493884	0.126694
YMR143W	RPS16A	-0.599466	0.0346025	0.096177
YDR382W	RPP2B	-0.589555	0.0261842	0.0766
YPR132W	RPS23B	-0.583809	0.0406487	0.109395
YOR167C	RPS28A	-0.576323	0.0495282	0.126891
YHR021C	RPS27B	-0.560382	0.0628014	0.152233
YGR118W	RPS23A	-0.559219	0.042936	0.113849
YML026C	RPS18B	-0.557373	0.059988	0.146896
YGL135W	RPL1B	-0.55551	0.0363786	0.100071
YJL136C	RPS21B	-0.549716	0.0446804	0.117424
YJR094W-A	RPL43B	-0.536115	0.0754008	0.17443
YJR145C	RPS4A	-0.533175	0.0835771	0.188317
YLR075W	RPL10	-0.512194	0.0608524	0.148472
YJL189W	RPL39	-0.505149	0.0831211	0.18768
YMR230W	RPS10B	-0.503579	0.0968439	0.210189
YPL220W	RPL1A	-0.468115	0.0691459	0.163534
YKL156W	RPS27A	-0.41425	0.1248119	0.252818
YGL076C	RPL7A	-0.39988	0.1361771	0.270211
YHR062C	RPP1	-0.376622	0.1612654	0.303452
YNL067W	RPL9B	-0.371443	0.1648438	0.308062
YDL133C-A	RPL41B	-0.319501	0.2893062	0.453218
YBR084C-A	RPL19A	-0.317091	0.2983566	0.46308
YDL184C	RPL41A	-0.307528	0.3224259	0.486798
YPL198W	RPL7B	0.1986869	0.5286336	0.665783
YPL081W	RPS9A	0.5890903	0.0698796	0.164873
YNL301C	RPL18B	1.060323	4.82E-05	0.000323
YJL191W	RPS14B	1.3124904	2.56E-06	2.49E-05
