# Supplementary material for: The Exosome Component Rrp6 Is Required for RNA Polymerase II Termination at Specific Targets of the Nrd1-Nab3 Pathway
Source: PLoS Genet. 2015 Feb 13;11(2):e1004999. doi: 10.1371/journal.pgen.1004999 (PMC4378619; doi:10.1371/journal.pgen.1004999)
Supplement: S4 Table — Table includes differential expression data expressed in log2 rrp6Δ/WT ratio (i.e. fold change), as well as p-values and false discovery rate (FDR), all calculated from four replicates by the EdgeR program as discussed in the methods. Significantly downregulated sn/snoRNAs are in red-shaded cells whereas upregulated sn/snoRNAs are in green-shaded cells. (PDF) [file pgen.1004999.s009.pdf]

Table S4: Differential expression data for sn / snoRNA transcripts Page 1

Transcript class	Standard gene name	RRP6 _KO_ vs WT log2 FC	RRP6 _KO vs WT p-value	RRP6 _KO vs_WT FDR
sn/snoRNA	SNR81	-2.86038652	4.85E-14	4.14E-12
sn/snoRNA	SNR44	-2.23089965	2.77E-07	3.50E-06
sn/snoRNA	SNR35	-1.92814627	2.05E-10	6.41E-09
sn/snoRNA	SNR56	-1.5238076	6.39E-05	0.000413774
sn/snoRNA	SNR86	-1.4623751	8.21E-07	9.17E-06
sn/snoRNA	SNR65	-1.43094174	9.68E-06	7.85E-05
sn/snoRNA	SNR78	-1.36595297	5.82E-04	0.002888894
sn/snoRNA	SNR64	-1.36489882	2.01E-05	0.000148358
sn/snoRNA	SNR84	-1.23607228	0.000125125	0.000748372
sn/snoRNA	SNR33	-1.21593305	9.83E-05	0.000603253
sn/snoRNA	SNR66	-1.19594955	9.14E-05	0.000563868
sn/snoRNA	SNR59	-1.10752129	0.014237604	0.046625789
sn/snoRNA	SNR189	-1.04874335	0.001930926	0.008499109
sn/snoRNA	SNR191	-0.89679544	0.020416846	0.062502573
sn/snoRNA	snR61	-0.84975548	0.013504106	0.044647673
sn/snoRNA	SNR34	-0.83318708	0.014336742	0.046878903
sn/snoRNA	SNR71	-0.76595061	0.015401095	0.04976118
sn/snoRNA	SNR87	-0.67537383	0.016687617	0.053235664
sn/snoRNA	SNR46	-0.66127403	0.02844551	0.082096313
sn/snoRNA	SNR50	-0.62821069	0.029626572	0.084924341
sn/snoRNA	SNR31	-0.71372803	0.037316016	0.102087548
sn/snoRNA	SNR14	0.711639383	0.035216361	0.09758774
sn/snoRNA	SNR6	0.82703613	0.022085331	0.066560823
sn/snoRNA	SNR57	0.881604448	0.006580374	0.024512926
sn/snoRNA	SNR75	1.069032702	0.004722081	0.01845638
sn/snoRNA	SNR42-A	1.878888595	2.73E-08	4.71E-07
sn/snoRNA	SNR87-A	2.31736285	1.76E-09	4.31E-08
sn/snoRNA	SNR39B	-0.90902784	0.039039925	0.106084238
sn/snoRNA	SNR40	-0.59158803	0.052996397	0.133670752
sn/snoRNA	SNR36	-0.63511102	0.058982769	0.145245968
sn/snoRNA	SNR80	-0.69362677	0.0740974	0.17212582
sn/snoRNA	SNR128	-0.57419583	0.118859877	0.243317703
sn/snoRNA	SNR42	-0.54936932	0.06806735	0.161660948
sn/snoRNA	SNR52	-0.53752662	0.120245375	0.245605501
sn/snoRNA	SNR8	-0.5323453	0.142159353	0.278487816
sn/snoRNA	SNR79	-0.52774415	0.149737192	0.287773936
sn/snoRNA	SNR18	-0.51938153	0.085166497	0.190933516
sn/snoRNA	SNR47	-0.51725443	0.143937233	0.280475953
sn/snoRNA	SNR83	-0.50571291	0.095794227	0.208546845
sn/snoRNA	SNR60	-0.48715501	0.218627515	0.374429324
sn/snoRNA	SNR9	-0.45297193	0.198883562	0.349702151
sn/snoRNA	SNR77	-0.44424895	0.149459373	0.287404332
sn/snoRNA	SNR161	-0.42355302	0.179671466	0.326981965
sn/snoRNA	SNR43	-0.4164562	0.226726278	0.383525692
sn/snoRNA	SNR32	-0.3838458	0.2510801	0.412302527
sn/snoRNA	SNR55	-0.37429956	0.296846966	0.461555507
sn/snoRNA	SNR38	-0.35540613	0.340398658	0.504939698
sn/snoRNA	SNR190	-0.32484309	0.361747053	0.525783972
sn/snoRNA	SNR49	-0.31873561	0.313881222	0.479209424
sn/snoRNA	SNR54	-0.29666824	0.338839999	0.50344225
sn/snoRNA	SNR3	-0.26996702	0.452510653	0.603581053
sn/snoRNA	SNR70	-0.26471889	0.394224359	0.555372791
sn/snoRNA	SNR63	-0.26143756	0.403192231	0.562039045
sn/snoRNA	SNR53	-0.25869215	0.477901804	0.624237615
sn/snoRNA	SNR30	-0.24430417	0.434322121	0.588828215

Transcript class	Standard gene name	RRP6 _KO_ vs WT log2 FC	RRP6 _KO vs WT p-value	RRP6 _KO vs_WT FDR
sn/snoRNA	SNR76	-0.22956588	0.418323974	0.574389084
sn/snoRNA	SNR24	-0.21919324	0.503883819	0.645965476
sn/snoRNA	SNR37	-0.19816656	0.533192777	0.669193761
sn/snoRNA	SNR4	-0.12520244	0.739787893	0.827326293
sn/snoRNA	SNR41	-0.12260904	0.665292218	0.771659389
sn/snoRNA	SNR48	-0.09075816	0.808692987	0.875736692
sn/snoRNA	SNR45	-0.08311355	0.795439103	0.867438943
sn/snoRNA	SNR69	-0.07913845	0.782649698	0.859253082
sn/snoRNA	SNR67	-0.05616709	0.856106882	0.907683924
sn/snoRNA	SNR72	-0.05584022	0.859360009	0.909698334
sn/snoRNA	SNR11	-0.0514268	0.89548336	0.933960656
sn/snoRNA	SNR7	-0.0381921	0.903347021	0.93988355
sn/snoRNA	SNR82	0.020484443	0.948940617	0.970148067
sn/snoRNA	SNR17A	0.055254753	0.862752868	0.911471967
sn/snoRNA	SNR17B	0.072352447	0.81444406	0.880168674
sn/snoRNA	SNR58	0.092225721	0.760947806	0.843045229
sn/snoRNA	SNR39	0.106069082	0.71306577	0.806839679
sn/snoRNA	SNR62	0.15611664	0.674808634	0.778214861
sn/snoRNA	SNR19	0.158597522	0.586520686	0.711472945
sn/snoRNA	SNR51	0.206204667	0.507219697	0.64875741
sn/snoRNA	SNR68	0.243528938	0.498762767	0.641357274
sn/snoRNA	SNR5	0.259200468	0.481388595	0.627416647
sn/snoRNA	SNR13	0.279318313	0.418252867	0.574389084
sn/snoRNA	SNR85	0.306657293	4.10E-01	5.68E-01
sn/snoRNA	SNR74	0.374300957	2.84E-01	4.47E-01
