# Supplementary material for: The Exosome Component Rrp6 Is Required for RNA Polymerase II Termination at Specific Targets of the Nrd1-Nab3 Pathway
Source: PLoS Genet. 2015 Feb 13;11(2):e1004999. doi: 10.1371/journal.pgen.1004999 (PMC4378619; doi:10.1371/journal.pgen.1004999)
Supplement: S5 Table — Oligonucleotide sequences for each strand specific probe used for end labeling and northern blot analysis. (PDF) [file pgen.1004999.s010.pdf]

Table S5: Sequences for DNA oligonucleotide probes used for northern blot

Name	Sequence	Reference
snR13 Probe	GCC AAA CAG CAA CTC GAG CCA AAT GCA CTC	This study
snR3 Probe	GCT CGA TCT TCG TAC TGT CTA ATG CGG TGG	This study
snR11 Probe	CTA TCA ACC GCG AGC ACG ACA GTG	This study
FMP40 Probe	GTA CCC AAC TTC TGG GGA CAA ACA ACG GG	This study
YPL222C-A Probe	CCC GTT GTT TGT CCC CAG AAG TTG GGT AC	This study
